# Aggregation of DNA oligomers with a neutral polymer facilitates DNA solubilization in organic solvents for DNA-encoded chemistry

**DOI:** 10.1039/d5sc06782k

**Published:** 2025-10-28

**Authors:** Johannes Bingold, Erik Mafenbayer, Wibke Langenkamp, Lisa Liang, Chun Zhang, Malte Mildner, Julia Isabel Bahner, Mohamed Akmal Marzouk, Bettina Böttcher, Ann-Christin Pöppler, Ralf Weberskirch, Andreas Brunschweiger

**Affiliations:** a Institute of Pharmacy and Food Chemistry, Julius-Maximilians-Universität Würzburg Am Hubland 97074 Würzburg Germany andreas.brunschweiger@uni-wuerzburg.de; b Department of Chemistry and Chemical Biology, Polymer Hybrid Systems, TU Dortmund University Otto-Hahn Straße 6 44227 Dortmund Germany; c Institute of Organic Chemistry, Julius-Maximilians-Universität Würzburg Am Hubland 97074 Würzburg Germany; d Rudolf-Virchow-Center, Julius-Maximilians-Universität Würzburg Josef-Schneider-Straße 2 97080 Würzburg Germany; e Biocenter, Julius-Maximilians-Universität Würzburg Am Hubland 97074 Würzburg Germany

## Abstract

Chemical diversification of DNA-conjugated substrates is key in DNA-encoded library (DEL) synthesis and other nucleic acid-based technologies. One major challenge to the translation of synthesis methods to DNA-tagged substrates is the lack of solubility of the highly charged DNA oligomer in most organic solvents. A neutral acrylate block copolymer, devoid of any canonical nucleic acid-binding structure, tightly interacted with DNA oligonucleotides in their ammonium form, and solubilized them in nonpolar solvents such as dichloromethane, chloroform and toluene. The ternary DNA–copolymer–ammonium salt interactions led to the formation of aggregates in organic solvents whose size correlated with DNA oligomer length. This method for DNA solubilization was successfully applied to diversify DNA-tagged starting materials by three isocyanide multicomponent reactions (IMCR) with broad scope and excellent yields. The copolymer does not require tailored DNA conjugates and solubilized DNA oligomers of up to 80 nucleotides length. It will likely broaden the toolbox of DEL-compatible synthesis methods well beyond IMCR chemistry and it has application potential in other nucleic acid-based technologies that require a broadened solvent scope for nucleic acid conjugate synthesis.

## Abstract 2

A neutral block copolymer interacted with DNA oligomers and solubilized them in nonpolar solvents. This approach allowed for reacting DNA-tagged substrates by isocyanide multicomponent reactions with broad scope and excellent yields.

## Graphical abstract

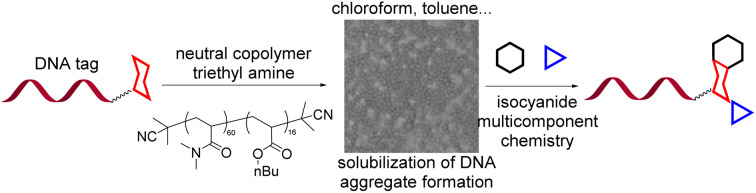

## Introduction

**Fig. 1:**
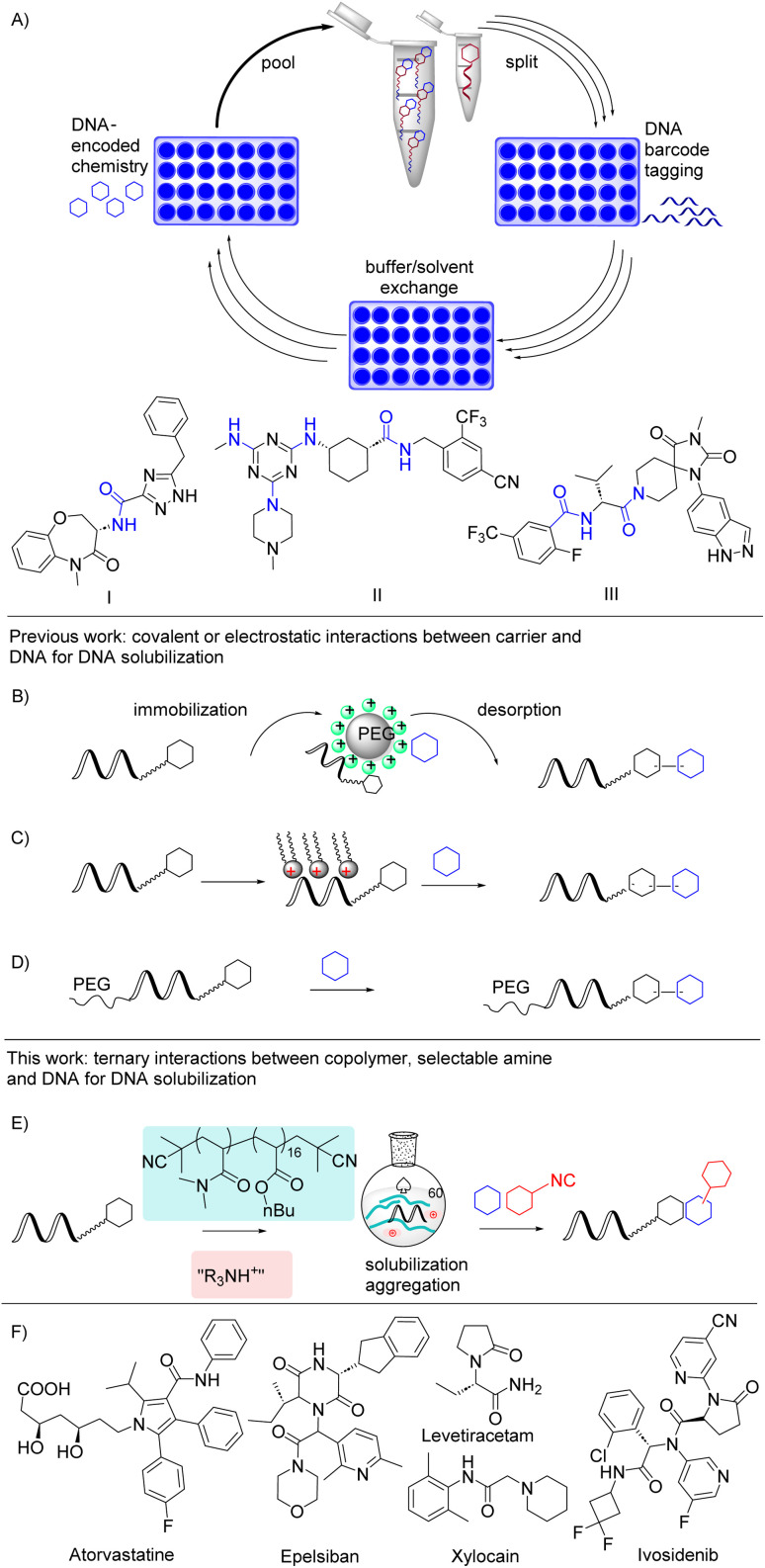
Strategies for DNA-encoded chemistry in organic solvents. (A) DNA-encoded split-pool synthesis and published clinical candidates that originated from DEL screens. (B) Immobilization of DNA tags on ion exchange resins. (C) Solubilization of DNA tags in polar organic solvents as tight ion pairs with cationic surfactants. (D) Encoded chemistry on a PEG polymer–DNA conjugate. (E) This work: solubilization of DNA tags as ammonium salts in organic solvents with an amphiphilic block copolymer and application to three isocyanide multicomponent reactions (IMCR). (F) Approved drugs synthesized by IMCRs.

DNA-encoded library (DEL) technologies are a widely used small molecule screening platform.^1–4^ Commonly, DELs are synthesized by solution-phase split-pool combinatorial chemistry that gives efficient access to vast compound libraries (Fig. 1A). The technology has delivered three published clinical candidates whose chemotypes illustrate the building block logic of encoded compound design and the validated coupling chemistry methodology for DELs (I–III, Fig. 1A).^1–3^ DEL synthesis reactions need to be compatible with the delicate barcode tag, with the combinatorial synthesis process, and they are usually performed in homogeneous solution, *i.e.* they require water or aqueous co-solvents to dissolve the polyanionic DNA barcode tags. However, water and aqueous co-solvents may not dissolve hydrophobic substrates,^5^ they may damage water-sensitive catalysts, and they may cause hydrolysis reactions that compete with the intended reaction and lead to side products, as has previously been shown in DEL screenings.^6^ Furthermore, reactions under aqueous reaction conditions may damage the genetic tag itself by hydrolytic damage pathways.^7^ The poor solubility of DNA oligonucleotides in most organic solvents is a major obstacle to the development of methods for the diversification of oligonucleotide-tagged starting materials by a toolbox of synthesis methods that goes beyond “a few good reactions”, which are mainly carbonyl coupling reactions, click reactions, and the Suzuki reaction.^8–10^ Approaches to convince DNA to take the step from its natural habitat water and find a temporary home in dry organic solvents could be a key step towards broadening the toolbox of “good” DEL reactions.

In the early times of DNA-encoded chemistry, DNA-templated reactions have been shown in a 95:5 mixture of acetonitrile and water.^11^ Pehr Harbury pioneered reversible immobilization of DNA tags by Coulomb interactions on ion-exchange solid phase. This approach was subsequently adopted and refined by several DEL research groups (Fig. 1B).^12–15^ It requires an immobilization and a high-salt desorption step, and the reaction conditions need to be compatible with the solid support. In the solution phase, cationic lipids like didodecyldimethylammonium bromide have been shown to form tight ion pairs with oligonucleotides (6mer, 14mer and 22mer) and to solubilize these ion pairs in polar organic solvents such as DMF, THF and DMSO for polymer conjugation reactions. This approach facilitated a photoredox reaction on DEL barcodes (Fig. 1C).^5,16^

Polymers and copolymers are a further class of structures that have been shown to solubilize DNA in organic solvents (Fig. 1D). For instance, block copolymers composed of a polyethylene glycol block and a cationic block formed nanoparticles with calf thymus DNA in polar organic solvents and benzene.^17^ Long, 10 kDa PEG polymers were covalently attached to DNA oligomers of up to 21 nucleotides length, including G-quadruplex DNA. These DNA PEG–polymer conjugates were soluble in dichloroethane and polar solvents,^18^ and they allowed for investigating DNA structures in organic solvents. DNA PEG-conjugates were shown to allow for amide couplings on DNA barcodes in polar organic solvents, but required a tailored DNA barcode-PEG conjugation strategy.^19^ Alternatively, DELs may be synthesized *via* covalently coupled barcodes on solid phases.^20–24^ These solid phase approaches may need tailored substrates, and they require a cleavage step if the libraries are not screened on the solid phase.^25^ This research work shows the ongoing interest of DEL and nucleic acid chemists in approaches to expand the solvent scope for reactions on nucleic acids. The very few approaches encourage investigating new avenues to “DNA in dichloromethane” for applications in nucleic acid-based technologies. Here, we deliver a conceptually new approach to solubilization of DNA oligomers in non-aqueous solvents for *e.g.* DNA-encoded chemistry that exploits ternary interactions between a DNA, a neutral block copolymer and selectable nitrogenous cations.

We have previously investigated poly(*N*,*N*-dimethylacrylamide)–poly(*n*-butyl acrylate) block copolymers (Fig. 1E) as micellar nanoreactors for DNA-encoded chemistry in aqueous solvents.^26^ These copolymers formed micellar structures in water and localized a sulfonic acid moiety either in the core or in the corona of the micelles. The copolymer micelles promoted Povarov and Groebke–Blackburn–Bienaymé reactions with DNA-tagged aldehydes. Investigations in this micellar reaction system revealed that the DNA oligomers tightly interacted with the copolymer, and indeed more than 99.9% of the DNA were associated with the copolymer micelles under low-salt conditions.^27^

The observation that DNA oligomers tightly interacted with the acrylate copolymer led us to hypothesize that the copolymer–DNA complexes might allow for exchanging the bulk solvent from an aqueous environment to a pure organic solvent without precipitation of the DNA oligomer. In this manuscript, we present our findings that the block copolymer shown in Fig. 1E, which is devoid of any canonical DNA-binding structures such as positive charges or intercalators, solubilized DNA oligomers of up to 80 nucleotides length as salts of organic amines in non-aqueous solvents. In organic solvents, the DNA oligomers tightly interacted with the copolymer, forming aggregates that correlated with DNA oligomer length. To investigate the potential of this DNA solubilization method for DNA-encoded chemistry, we selected hydrolysis-sensitive imine multicomponent reactions (MCRs) with slow reaction kinetics (Fig. 1E), *i.e.* reactions that do not show desirable properties for DEL synthesis.^1,2,8,9^ These MCRs are underdeveloped in the DEL context, but workhorse reactions in drug research, because they give access to a wide range of structurally diverse scaffolds from simple, readily available starting materials (Fig. 1F).^28–31^ Diverse DNA-tagged aldehydes were diversified in the copolymer–DNA interaction system in an operationally simple manner by Ugi- (U-4CR) and Ugi-azide (UA-4CR) isocyanide multicomponent reactions (IMCRs). To probe the compatibility of the copolymer system with an acid catalyst we investigated the Groebke–Blackburn–Bienaymé reaction (GBB-3CR). All reactions proceeded with high, in most cases even quantitative conversions of the DNA-tagged starting materials. DNA-ligation and sequencing experiments showed that the reaction conditions were DNA-compatible, and successful reactions with mixtures of substrates suggested DEL-compatibility.

## Results and discussion

### DNA solubilization in organic solvents and DNA copolymer interactions

**Fig. 2:**
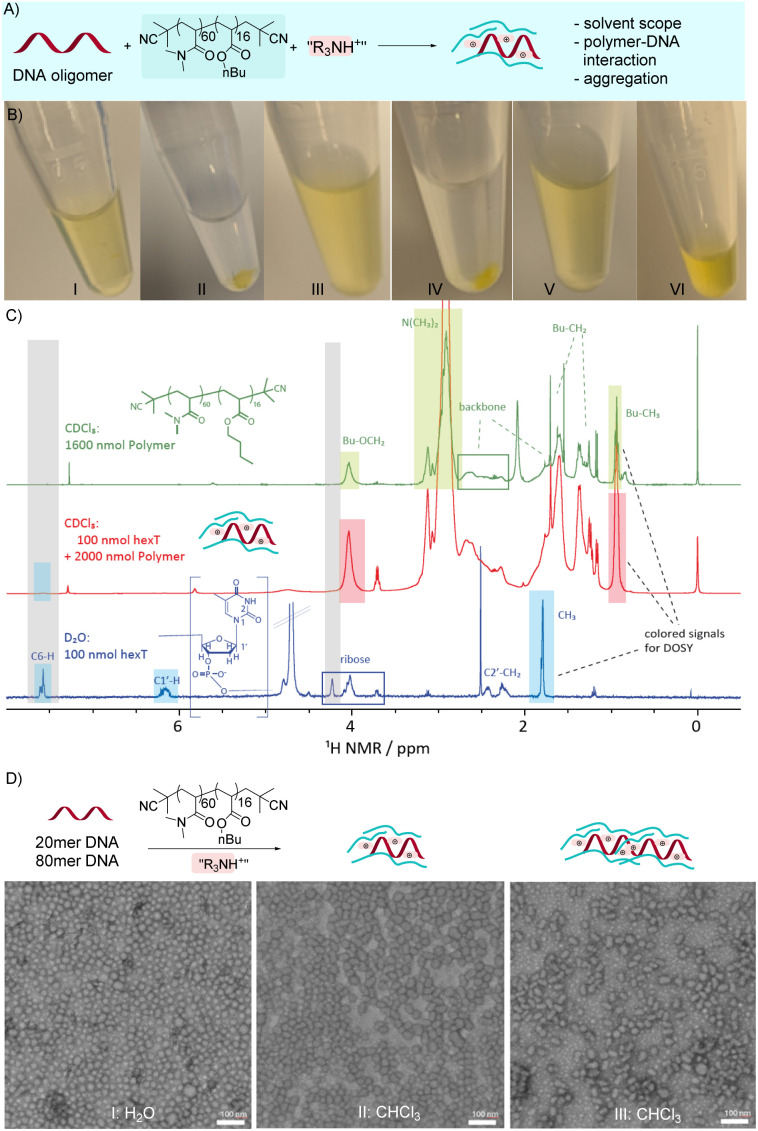
DNA solubilization experiments and structural characterization of DNA–copolymer aggregates in organic solvents. (A) DNA oligomers were solubilized by the copolymer in organic solvents. (B) Photographical pictures of FAM-labelled 20mer DNA oligomer solubilized by the copolymer in various organic solvents: (I) DNA + copolymer + Et_3_NH^+^ in toluene; (II) DNA in DCM w/o copolymer; (III) DNA + copolymer + Et_3_NH^+^ as counterion in DCM; (IV) DNA in CHCl_3_ w/o copolymer; (V) DNA + copolymer + Et_3_NH^+^ as counterion in CHCl_3_; (VI) DNA + copolymer + NH_4_^+^ as counterion in DCM. (C) ^1^H NMR spectra of a hexathymidine (hexT) DNA oligomer in D_2_O (lower panel), a mixture of hexT and the copolymer (middle panel), and the copolymer alone (upper panel) in CDCl_3_. The spectra were scaled for better comparability. Signal assignment is indicated and well separated signals for the DOSY analysis, are highlighted by colored boxes. (D) Structural characterization of DNA–copolymer mixtures by transmission electron microscopy (TEM). (I) TEM image of 20mer DNA (5 nmol in 300 μL) and 30 equiv. copolymer in water; (II) TEM image of 20mer DNA (5 nmol in 300 μL) and 30 equiv. copolymer in chloroform; (III) TEM image of 80mer DNA (5 nmol in 300 μL) and 30 equiv. polymer in chloroform.

We tested our hypothesis that the copolymer could solubilize DNA in organic solvents with a fluorescence-labelled 10mer DNA oligonucleotide in the sodium form that was dissolved together with 30 equivalents of the copolymer in neat water (Fig. 2A). However, after evaporation of water the addition of various organic solvents to the residue yielded only a DNA pellet in every case (Fig. S1). We then probed the impact of the counterion, as the DNA–copolymer interaction was strictly ion strength-dependent in our previous DNA copolymer interaction studies.^27^ The solubilization experiments were repeated with nitrogenous salts of the DNA, including salts of triethylamine, DIPEA (Hünig base), piperidine, pyridine, ammonia, and imidazole. Again, various organic solvents were added to the residual DNA–copolymer mixture to probe DNA solubilization (Fig. 2B, S2, S5 and Table S1). To our delight, non-polar aprotic solvents dichloromethane, chloroform and toluene readily dissolved the organic salts of the DNA oligomer complexed with the copolymer. Curiously, polar solvents acetonitrile, THF, MeOH and DMF failed to dissolve the DNA–copolymer complexes, a behaviour that distinguished our solubilizing principle from cationic lipids and the PEG polymer conjugates.^5^ If polar solvents were needed for a given DEL reaction, these solvents could be added conveniently to a chloroform or dichloromethane stock solution of a DNA tag (Table S1 and Fig. S3). For instance, methanol could be added in ratios of up to 9:1 to a dichloromethane stock solution of a DNA tag (Fig. S3). This could be due to almost all the polar amide interacting with the DNA and the tertiary amine and only the non-polar *tert*-butyl ester interacting with the organic solvent. As a result, the DNA–copolymer mixture was only soluble in non-polar aprotic solvents. The copolymer (30 eq.) readily solubilized 6mer to 80mer single-stranded DNA oligomers in dichloromethane and chloroform (Table S1 and Fig. S2–S5). The DNA tags were conveniently removed from the organic phase by adding an aqueous sodium chloride solution and subsequent extraction of the DNA into the aqueous phase (Fig. S6). Alternatively, the organic solvent was evaporated, and the DNA was precipitated by addition of an ethanolic sodium acetate solution (EtOH/water 3:1) (Fig. S6). The DNA could be isolated by HPLC ion-pair chromatography after evaporation of the organic solvent, too, as the small amounts of the copolymer did not disturb the chromatographic system.

The solubilization of DNA in chloroform by the copolymer was investigated by dynamic light scattering (DLS), NMR and transmission electron microscopy (TEM) analysis. DNA oligomers of different length were solubilized in chloroform or toluene with the copolymer, and indeed particle formation could be observed by DLS measurements in all experiments where copolymer and DNA were present (Fig. S13–S23). It should be noted at this point that the block copolymer alone did not form aggregates in the organic solvents chloroform, DCM, and toluene. Copolymer aggregation was solely found to be induced by the presence of DNA oligomers.

Curiously, the size of the particles was strictly depending on the length of the DNA oligomers, and we observed an approximate correlation between DNA oligomer length and particle size (Fig. S13–S23). For example, a 10mer DNA formed particles with an average diameter of 14 nm, a 20mer DNA formed particles with an average diameter of 35 nm, and a 40mer DNA formed particles with an average diameter of 74 nm. Even the counter ions showed a small, albeit measurable impact on particle size (Fig. S14–S16). Furthermore, we observed a measurable impact of amine substrates on the particle size for the U-4C, UA-4C, and GBB-3C reactions (reaction development *vide infra*) which hinted at a close DNA–copolymer–amine substrate interaction (Fig. S17).

The interaction of DNA and copolymer was investigated by ^1^H and ^1^H DOSY NMR (Fig. 2C, S24, S25 and Table S2). We selected a short hexathymidine (hexa-T) DNA for these experiments, because the signals of a nucleobase homomer can be readily detected even at the typically low DNA concentrations. The ^1^H NMR spectra of the pure polymer in CDCl_3_ (upper spectrum) and pure hexa-T in D_2_O (lower spectrum) were used for signal assignment and identification of relevant signal areas. The spectrum of the same concentration of hexT (TEA salt) incubated with 20 equivalents of the polymer in CDCl_3_ showed only weak and broadened signals for the hexT DNA (*e.g.* blue boxes around 7.6 ppm). This indicates that hexa-T was part of a larger aggregate, resulting in a shorter *T*_2_ relaxation and thus broader lines. Triethylamine signals could not be observed in the spectra due to overlap with the polymer signals. We then performed DOSY experiments. The signals highlighted by coloured boxes were chosen for the further analysis as they show little signal overlap. Fitting of the respective DOSY signal decay curves yielded values of 2 × 10^−10^ m^2^ s^−1^ for the pure DNA oligomer in deuterated water and 1.8 × 10^−10^ m^2^ s^−1^ for the pure polymer in deuterated chloroform. For the two mixtures with 50 and 100 nmol hexa-T and 20 eq. of polymer, the diffusion of the polymer was slightly lower (∼1.4 × 10^−10^ m^2^ s^−1^). Despite the low signal intensity, for the mixture with 100 nmol hexT, a diffusion coefficient for hexa-T itself could also be estimated. The diffusion coefficient was 2.9 × 10^−11^ m^2^ s^−1^ and thus much lower than the diffusion coefficient of hexa-T in water at the same concentration. Therefore, we concluded hexT to be part of larger aggregates (Table S2). Due to the 20-fold excess of copolymer, the diffusion value of the copolymer was faster than hexa-T, which was consistent with a population of polymer chains in solution and a population of polymer chains that formed aggregates with the hexa-T DNA. Because DLS and NMR analyses supported the formation of larger structures by the DNA–copolymer interaction, we used transmission electron microscopy (TEM) to gain insight into the shape of these structures. TEM pictures readily confirmed that the copolymer and both a 20mer and 80mer DNA aggregated in chloroform (Fig. 2D). The particles induced by the 80mer DNA appeared to be elongated aggregates compared to the more round-shaped structures formed by the 20mer DNA and copolymer, confirming the observation of the impact of DNA oligomer length on particle size. The particle sizes visible in the TEM pictures agreed with the DLS measurements (Fig. 2D).

### Towards DNA-encoded multicomponent reactions in solution phase

Dissolving DNA tags in organic solvents promises to facilitate translation of reactions to DEL synthesis, considering the impact of solvents on starting material solubility and reaction kinetics as well as avoiding water-mediated undesired reactions.^32,33^ As first reactions to be investigated with the copolymer–DNA interaction system, we selected Ugi (U-4CR), Ugi-azide (UA-4CR), and Groebke–Blackburn–Bienaymé (GBB-3CR) isocyanide reactions (IMCRs). These reactions are widely used in the design of screening libraries, in the synthesis of drug candidates, and drugs (Fig. 1F).^28,34,35^ Important for DEL design, the family of these multicomponent reactions tap into an abundant source of mono- and bifunctional starting materials and gives efficient access to scaffold diversity.^36,37^ Isocyanide multicomponent reactions do not lend themselves as first choice for solution-phase DEL synthesis as they show slow reaction kinetics and depend on a hydrolysis-sensitive imine formation step. We have previously shown that IMCRs can be translated to controlled pore glass (CPG) solid phase-coupled DNA tags for a productive DEL design.^38–40^ However, in this approach the reaction can only be used in the first step of a DEL synthesis and several functional groups are excluded from library synthesis because of a cleavage step that requires high concentrations of ammonia. A micellar reaction system enabled the Groebke–Blackburn–Bienaymé reaction (GBB-3CR) in aqueous solvents, but reaction conversion rates were very low, probably because polar azole starting materials did not enter the micellar reaction site.^26^ The interest in multicomponent reactions as a versatile scaffold-generating tool for solution phase DEL design is documented by to date two publications in the field.^41,42^ The solution phase GBB-3R was shown with a focused substrate scope, and the lactam-yielding U-4C-3CR gave highly variable reaction yields, ranging from 10–90%. Despite the potential of isocyanide MCR chemistry for library design, no more efforts to bring this versatile chemistry to DEL have been published to date. The UA-4CR even is a new addition to the toolbox of solution phase DEL synthesis methods.

We are focused on the development of a barcoding strategy that uses a chemically stabilized tag (csDNA), consisting of pyrimidine nucleobases and 7-deazaadenine, because this barcode tolerated acidic conditions for compound synthesis and protective group removal.^21^ Therefore, we used a short 10-mer pyrimidine (TC) DNA as a readily available surrogate of a chemically stabilized DNA barcode tag to optimize reaction conditions for the three IMCRs and to investigate the reaction scope. With optimized conditions in hand, the IMCRs were tested on barcode oligomers and with mixtures of both csDNA-tagged and native DNA-tagged substrates to show the compatibility of the reactions with genetic tags and pooled substrates. Finally, the DNA tags were post reaction ligated, PCR-amplified and sequenced to show compatibility with a DNA barcoding process.

### Ugi four-component reaction

We initiated translation of the U-4CR to the DNA-tagged synthesis with a TC DNA-aldehyde conjugate 1a, 4-ethylaniline 2a, cyclohexylisocyanide 3a, and hexanoic acid 4a. An electron-rich aldehyde was chosen for reaction optimization because substrates of this kind react rather sluggishly, *i.e.* optimized reaction conditions should then be translatable to a broad scope of aldehyde starting materials. Initially, the reaction conditions were taken from previous work on solid-phase encoded Ugi reactions and tested in the solution phase system (Table 1).^38^

**Table 1:** Reaction conditions for the Ugi-four-component (U-4CR) reaction

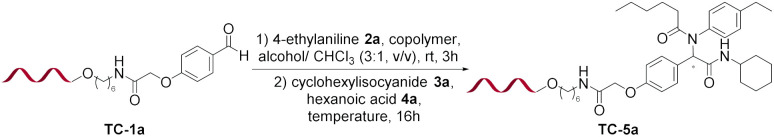					
No.	Solvent^a^	Eq. (2a, 3a, 4a)	*T* (°C)	Cat.	Conv. (%)
1	MeOH/CHCl_3_	250	50 °C	—	>95%
2	MeOH/CHCl_3_	500	50 °C	—	>95%
3	MeOH/CHCl_3_	1000	50 °C	—	>95%
4	MeOH/CHCl_3_	2000	50 °C	—	>95%
5	MeOH/CHCl_3_	2000	37 °C	—	>95%
6	MeOH/CHCl_3_	2000	25 °C	—	>95%
7	MeOH/CHCl_3_	2000	25 °C	ZnCl_2_	>95%
8	MeOH/CHCl_3_	2000	25 °C	MgCl_2_	>95%
9	MeOH/CHCl_3_	2000	25 °C	FeCl_2_	>95%
10	EtOH/CHCl_3_	2000	50 °C	—	>95%
11	iPrOH/CHCl_3_	2000	50 °C	—	>95%
12^b^	CF_3_CH_2_OH/CHCl_3_	3000	60 °C	—	>95%

a Alcohol/CHCl_3_ (3:1, vol/vol).

b All reactants were added simultaneously, and the reaction was run for 48 h.

**Fig. 3:**
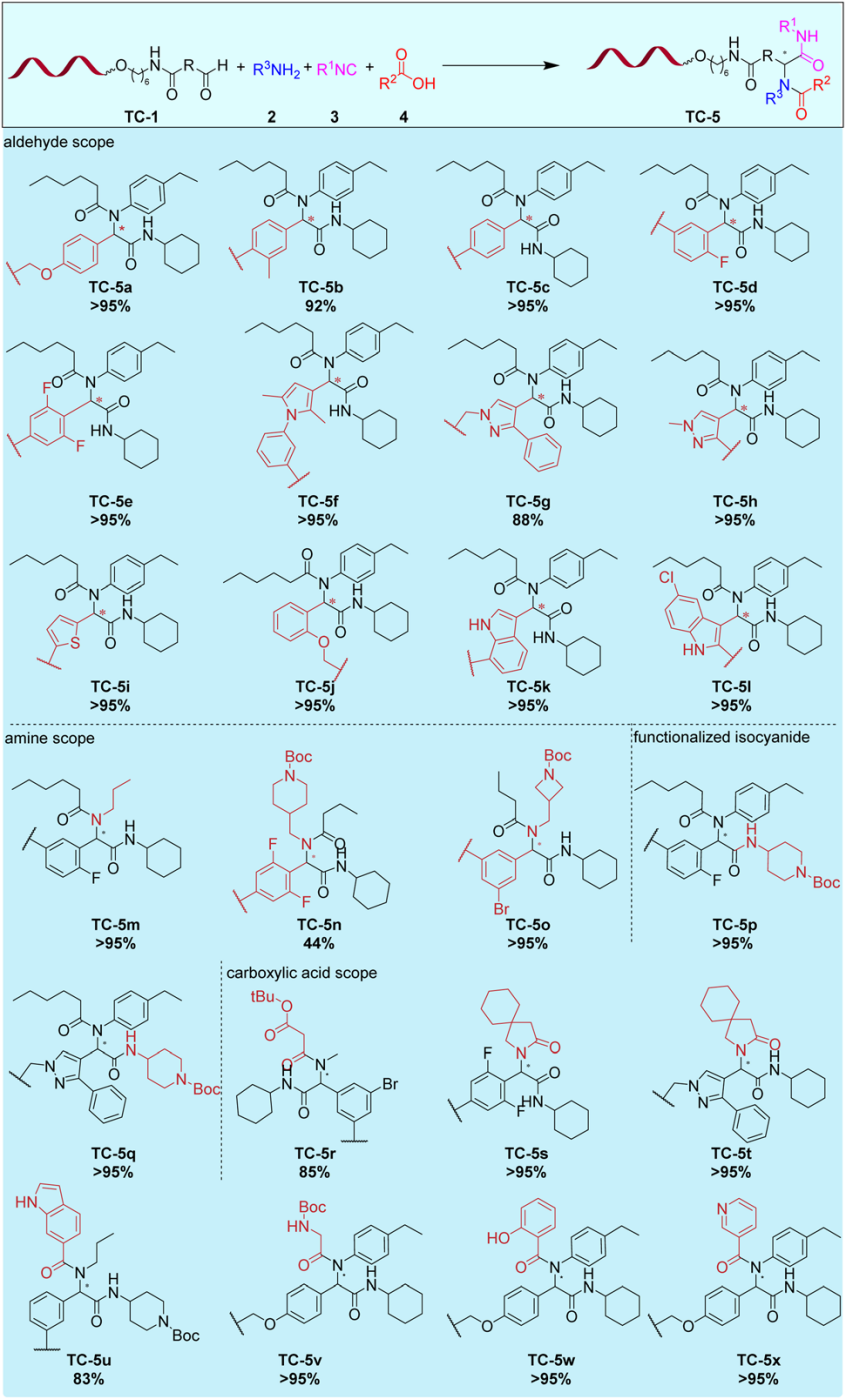
Scope of the Ugi four-component reaction on 10mer TC-DNA. Reaction conditions: TC-1 (3 nmol) and copolymer (90 nmol) and primary amine 2 (6 μmol) in MeOH/CHCl_3_ (50 μL; 3:1, v/v) for 3 h at rt. Addition of isocyanide 3 (6 μmol) and carboxylic acid 4 (6 μmol). The reaction was run for 16 h at 50 °C.

The DNA-tagged aldehyde 1a was condensed with the aniline 2a for imine formation, and then both the isocyanide 3a and the carboxylic acid 4a were added to the preformed imine. Already in the first experiments we obtained the target product with full conversion, and without side product formation. The U-4CR reaction was highly robust to several conditions. These included variation of starting material equivalents, reaction temperature, solvents, and addition of a few Lewis acid catalysts to the reaction (Table 1). In all experiments full consumption of the DNA-tagged aldehyde and clean conversion to the target Ugi dipeptide could be observed. Conveniently, the starting materials could be added simultaneously, *i.e.* without the separate imine formation step (Table 1, entry 12). This finding encouraged investigations into a broad substrate scope, including sterically hindered and electron-rich substrates (Fig. 3, extended scope in Table S4). First, several aldehydes were coupled to the TC-DNA (TC-1a–TC-1l). The scope of arylaldehydes was focused on sterically hindered *ortho*-substituted substrates (TC-1b-1f and TC-1i and TC-1j) and included substrates with low reactivity such as electron-rich indol-3-carbaldehydes (TC-1k and TC-1l).

All these DNA-conjugates were successfully reacted with 4-ethylaniline 2a, cyclohexylisocyanide 3a, and hexanoic acid 4a, giving the target Ugi dipeptides with quantitative consumption of the DNA-tagged aldehydes. In the next step, we varied simultaneously and with the same success both the aldehyde and the amine component, including aliphatic bifunctional amines (TC-5m–TC-5o). Then, 4-isocyano-*N*-Boc-piperidine 3b was validated as a bifunctional starting material which would allow for a further synthesis cycle, by *e.g.* carbonyl chemistry, starting from TC-5p-5q. The product TC-5r contained a *tert*-butyl protected dicarboxylic acid, which could be diversified by reverse amide synthesis. Finally, DNA-tagged aldehyde TC-1a was reacted with gabapentin and isocyanide 3a yielding the target lactams TC-5s and TC-5t by Ugi four-center three-component reaction (U-4C-3CR). Furthermore, diverse carboxylic acids, including a Boc-protected amino acid (TC-5v) were productive substrates for the U-4C reaction and gave the desired products with excellent to quantitative conversion rates (83% → 95%) (TC-5u–TC-5y, see SI part for an extended substrate scope). Taken together, the U4-CR was in the copolymer reaction system a very robust reaction regarding the reaction conditions and the substrate scope which numbered more than 50 examples and included aldehyde starting materials with low reactivity, starting materials with low solubility in aqueous solvents (*e.g.* Fmoc-protected building blocks), and diverse starting materials with protected functional groups for further library synthesis.

### Ugi azide four-component reaction

**Fig. 4:**
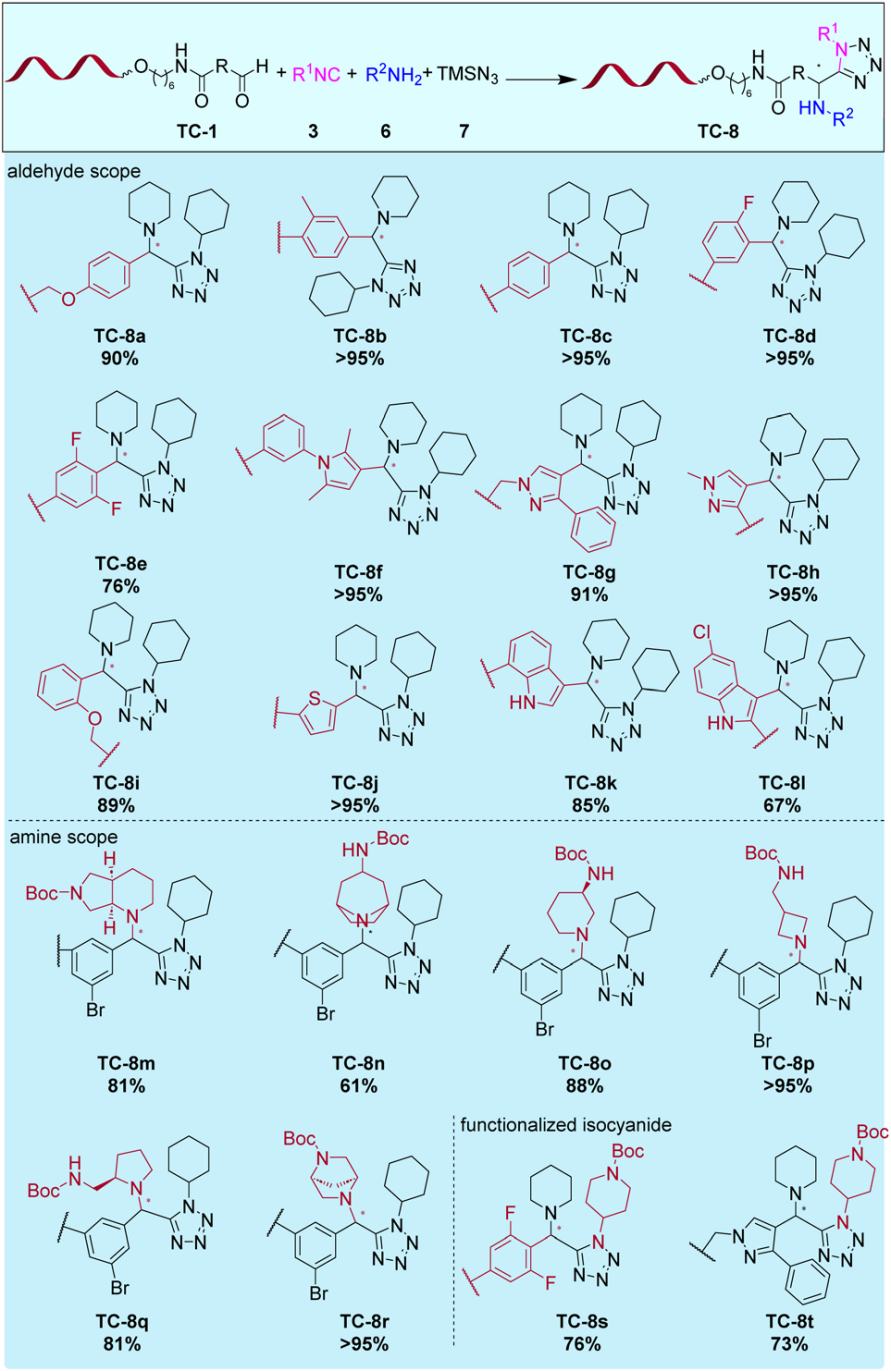
Scope of the Ugi-azide four-component reaction on 10mer TC-DNA. Reaction conditions: TC-1 (3 nmol) and copolymer (90 nmol) and secondary amine 6 (6 μmol) in MeOH/CHCl_3_ (50 μL; 3:1, v/v) for 3 h at rt. Addition of isocyanide 3 (6 μmol) and TMSN_3_7 (6 μmol). The reaction was run for 16 h at 50 °C.

Conditions for the solution phase UA-4CR were tested with the very same set of conditions that we used in the U-4CR development (Table 1). DNA-tagged aldehyde TC-1a, was reacted with isocyanide 3a, piperidine 6a and TMS-N_3_7 (Table 2). The UA-4CR was generally much more sensitive to reaction conditions than the U-4CR. Reaction conversions could be improved to nearly quantitative consumption of the DNA-tagged starting material by increasing the concentration of the starting materials at slightly elevated temperatures (Table 2, entries 1–6). Lower temperatures, in combination with Lewis acid catalysts, or less polar solvent mixtures all led to lower product conversions (Table 2, entries 7–11). Furthermore, we tested more forcing reaction conditions (60 °C, 48 h, entry 12) and added all reactants simultaneously. This condition led to a much lower conversion, and we concluded that the separate imine formation step was important for the reaction. With optimized reaction conditions in hand, the scope of the reaction was investigated regarding all variable components (Fig. 4, and extended scope in Table S7). The scope of aldehydes included the same aldehydes as for the U-4CR.

**Table 2:** Optimization of the Ugi-azide four-component (UA-4CR) reaction

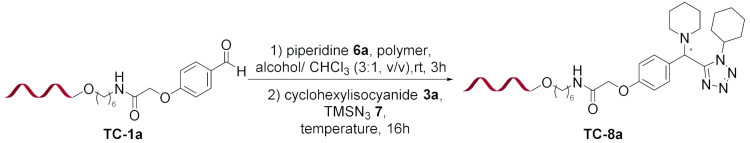					
No.	Solvent^a^	Eq. (3a, 6a, 7)	*T* (°C)	Cat.	% conv.
1	MeOH/CHCl_3_	250	50 °C	—	61%
2	MeOH/CHCl_3_	500	50 °C	—	70%
3	MeOH/CHCl_3_	1000	50 °C	—	67%
4	MeOH/CHCl_3_	2000	50 °C	—	90%
5	MeOH/CHCl_3_	2000	37 °C	—	87%
6	MeOH/CHCl_3_	2000	25 °C	—	67%
7	MeOH/CHCl_3_	2000	25 °C	ZnCl_2_	51%
8	MeOH/CHCl_3_	2000	25 °C	MgCl_2_	38%
9	MeOH/CHCl_3_	2000	25 °C	FeCl_2_	n.d.
10	EtOH/CHCl_3_	2000	50 °C	—	67%
11	iPrOH/CHCl_3_	2000	50 °C	—	38%
12^b^	CF_3_CH_2_OH/CHCl_3_	3000	60 °C	—	25%

a Alcohol/CHCl_3_ (3:1, vol/vol).

b All reactants were added simultaneously, and the reaction was run for 48 h.

All these DNA-tagged conjugates were successfully reacted with cyclohexyl isocyanide 3a, piperidine 6a and TMS-N_3_7 with conversion rates of 70% → 95%. Then, we varied the secondary amine component and DNA-aldehyde TC-1g was reacted with amines 6b–6g, isocyanide 3a and TMS-N_3_7 (Fig. 3 and Table S7). The set of amines also included several bifunctional amines that were mono-Boc-protected. These would allow for a further DEL-synthesis cycle by *e.g.* carbonyl chemistry. We observed conversion rates of >95% in most cases. Only a few building block combinations gave lower conversions which were still synthetically useful for DEL synthesis (*e.g.*TC-8n, 58% conversion).

### Groebke–Blackburn–Bienaymé three-component reaction

The GBB-3CR presented a similar picture as the U-4CR at the reaction development stage (Table 3). Under all conditions tested, which included variation of starting material concentrations (entries 1–4), temperature (entries 5 and 6), and solvents (entries 7 and 8), we obtained the target heterocycle with high (80%) to almost quantitative conversions.

**Table 3:** Reaction conditions for the Groebke–Blackburn–Bienaymé (GBB-3CR) reaction

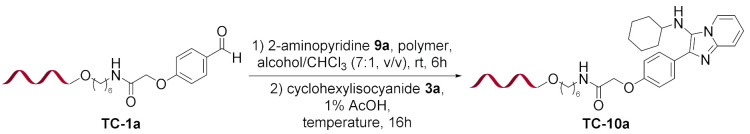					
No.	Solvent^a^	Eq. (3a, 9a)	*T* (°C)	Cat.^b^	% conv.
1	MeOH/CHCl_3_	250	25 °C	AcOH	81%
2	MeOH/CHCl_3_	500	25 °C	AcOH	89%
3	MeOH/CHCl_3_	1000	25 °C	AcOH	>95%
4	MeOH/CHCl_3_	2000	25 °C	AcOH	>95%
5	MeOH/CHCl_3_	2000	15 °C	AcOH	95%
6	MeOH/CHCl_3_	2000	5 °C	AcOH	>95%
7	EtOH/CHCl_3_	2000	25 °C	AcOH	>95%
8	iPrOH/CHCl_3_	2000	25 °C	AcOH	95%

a Alcohol/CHCl_3_ (7:1, vol/vol).

b 1% of acetic acid.

**Fig. 5:**
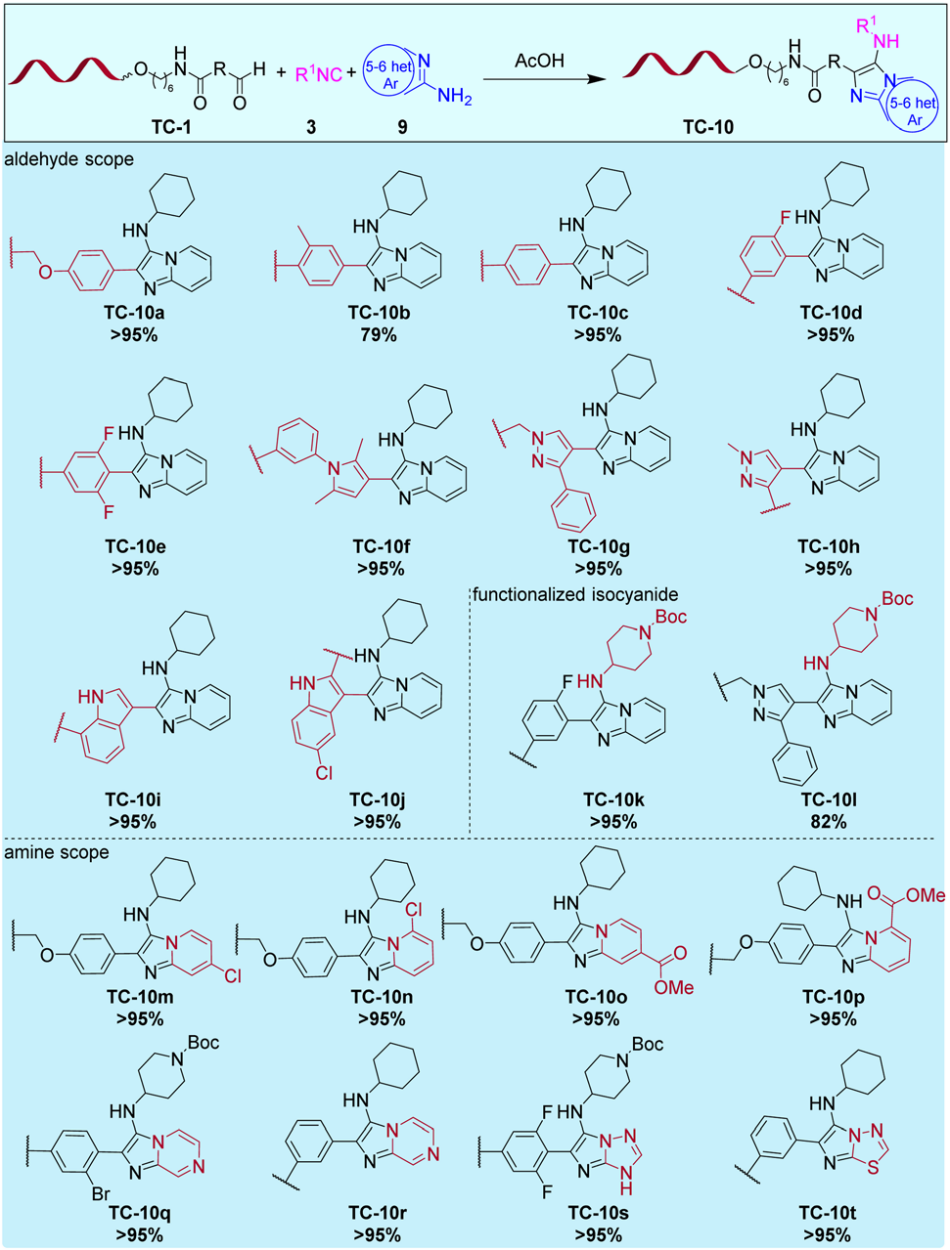
Scope of the Groebke–Blackburn–Bienaymé three-component reaction on 10mer TC-DNA. Reaction conditions: TC-1 (3 nmol) and copolymer (90 nmol) and heteroaromatic amine 9 (6 μmol) in MeOH/CHCl_3_ (80 μL; 7:1, v/v) for 6 h at rt. Addition of isocyanide 3 (6 μmol) and acetic acid (0.8 μL; *c* = 1 vol%). The reaction was run for 16 h at 25 °C.

The substrate scope of the GBB-3R was first tested with 10 diverse DNA-tagged aldehydes (TC1a–c, e, f, h–j, m, n), cyclohexylisocyanide 3a, and 2-aminopyridine 9a (Fig. 5). Again, we placed emphasis on *ortho*-substituted aldehydes to probe steric hindrance. Electron-rich indol-3-carbaldehydes that react sluggishly but are attractive structures from a compound library screening perspective were tested as well. We observed quantitative conversions to the target heterocycles TC-10a–j with all these substrate combinations, even with the more challenging aldehydes. The isocyanide component was exchanged to a Boc-protected piperidine, giving the option of a further scaffold diversification step (TC-10k and TC-10l). These two compounds were obtained quantitatively, too. As the last step, we also varied the heteroaromatic amine (azole component) 9. Here, building blocks 9b–9e with hydrolysis-sensitive functional groups were tested, which were inaccessible in the previously published solid-phase approach, because of the cleavage step with methylamine.^38^ These building blocks contained either a chloro-substituent, which could be substituted by a nucleophile (9b, 9c), or a methyl ester (9d, 9e) in *ortho*- or *para*-position. As expected, the heterocycles TC-10m–TC-10p were obtained with quantitative conversions in the non-aqueous reaction medium without side-reactions, and the functional groups remained intact. These compounds showed that the solution phase GBB-3R tolerated inclusion of different diversifiable functional groups for a future library design. Finally, we diversified the core heterocyclic scaffold and synthesized imidazopyrazines, TC-10q and TC-10r with quantitative conversion, too. To synthesize further core scaffolds, we tested five-membered aminoazoles 4*H*-1,2,4-triazol-3-amine, and 1,3,4-thiadiazol-2-amine which afforded the annulated 5 + 5 ring systems TC-10s and TC-10t with quantitative conversion.

In the copolymer reaction system, the GBB-3CR showed excellent robustness to different reaction conditions. A diverse scope of starting materials, including 10 different DNA-tagged aldehydes, functionalized building blocks such as a Boc-protected isocyanide substrate and halide- as well as ester-substituted aminopyridines was productive and furnished the desired heterocyclic products in straightforward manner and with almost quantitative conversions in all cases.

### DNA scope of IMCRs, reactions with DNA-tagged mixtures and DNA-compatibility of reaction conditions

The three IMCRs were optimized with a TC oligomer that served as a surrogate of a chemically stabilized barcode.^21^ For an encoded library synthesis scenario these model oligonucleotides have to be replaced by DNA barcode-tagged substrates.

**Fig. 6:**
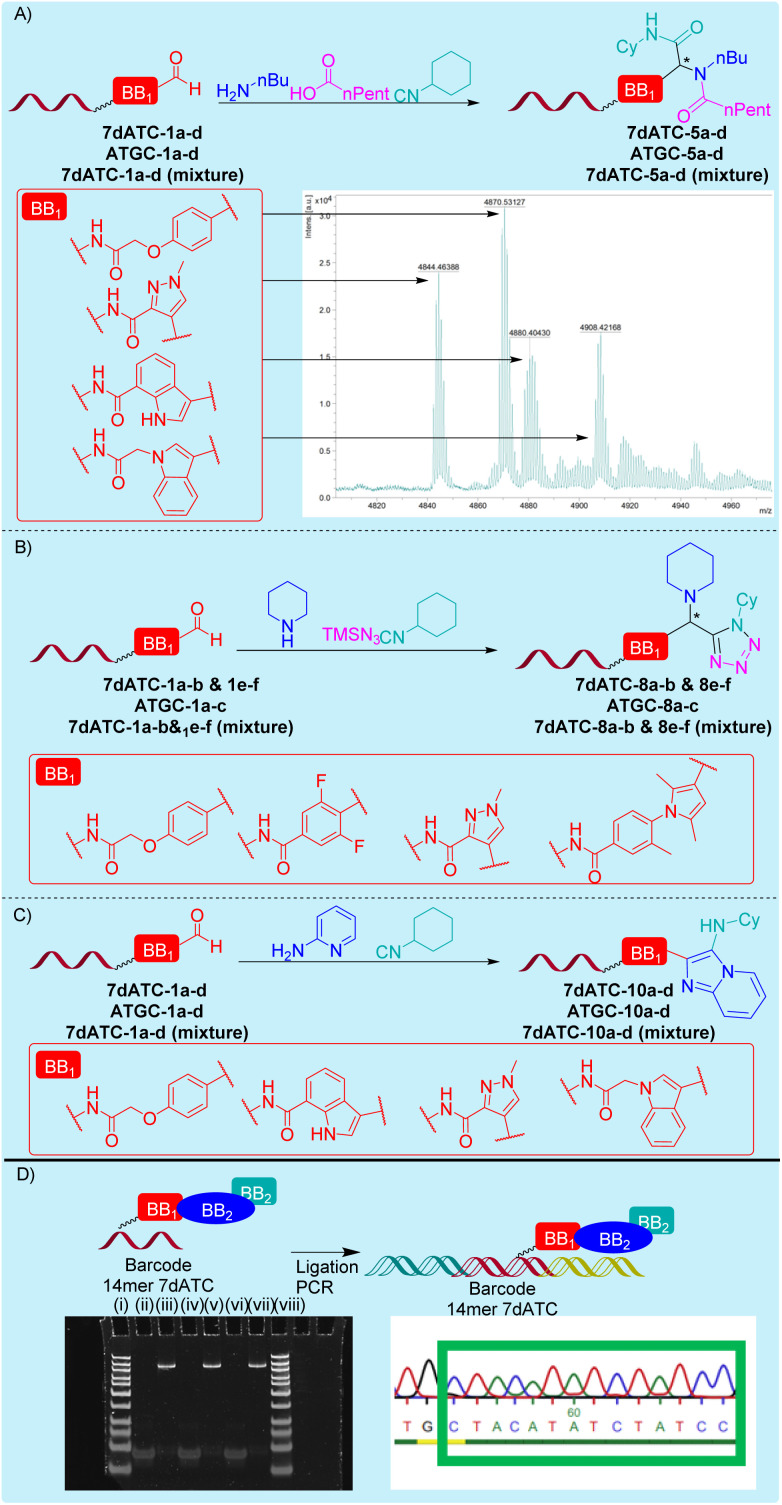
DNA-scope of multicomponent reactions, reactions with DNA-tagged mixtures and DNA compatibility assessment. DNA sequences: ATGC: 5′-(C6)aminolinker-CTACGTATGTGACC; 7dATC: 5′-CTACATAXCTATCC, X denotes a 5-triazolylthymine residue as linker to the barcoded compound. (A–C) Synthesis of DNA barcode-tagged product mixtures by Groebke–Blackburn–Bienaymé (A), Ugi-4-component (B), and Ugi-azide (C) reactions with mixtures of DNA-tagged aldehydes. As barcode we used a chemically stabilized 14mer DNA 7dATC. (D) DNA damage assessment after the three IMCRs on 7dATC-barcode by ligation, barcode amplification by PCR and Sanger sequencing of the amplicon. Analysis of ligated products by gel electrophoresis: (i) DNA ladder; (ii) 7dATC-5a; (iii) product of barcode ligation and barcode PCR of 7dATC-5a [U-4CR; 178 bp]; (iv) 7dATC-8a; (v) product of barcode ligation and barcode PCR of 7dATC-8a [UA-4CR, 178 bp]; (vi) 7dATC-10c; (vii) product of barcode ligation and barcode PCR of 7dATC-10c [GBB-3CR, 178 bp]; (viii) DNA ladder.

We selected two DNA barcodes. These were a chemically stabilized 14mer 7deATC barcode that consisted of pyrimidine nucleosides and 2′-deoxy-7-deazaadenosine (denoted as 7dATC), and an ATGC sequence that contained all four native nucleosides (denoted as ATGC). Each four carbaldehyde building blocks were coupled to both sequences giving 7dATC-1–4 and ATGC-1–4. These conjugates were reacted under the same conditions of the GBB-3CR, U4C-R, and UA-4CR as previously used for the TC oligomer (Fig. 6A and Tables S10–S24). In the case of the GBB-3CR, the reaction outcome of the TC-coupled starting materials could be reproduced with the barcode sequences, *i.e.* the DNA-tagged aldehydes were fully converted to the desired imidazopyridine (Tables S10–13). The U-4CR (Tables S15–S18), and UA-4CR (Tables S20–23) gave synthetically useful 60–70% conversion to the target compounds under the conditions that gave full conversion to the corresponding pyrimidine DNA conjugates TC-5 and TC-8. For the U-4CR, we extended the reaction time to 48 h, increased the temperature to 60 °C and substituted methanol by trifluoroethanol. Gratifyingly, these conditions allowed us to add all reactants as one single mixture to the DNA-tagged aldehyde, *i.e.* we could omit the separate imine formation step, and the reaction conversions for the U-4CR to were improved to 85%. In all cases, the DNA oligomers stayed intact, *i.e.* we did not observe formation of 8-oxopurines or abasic sites, which would be clearly visible by mass-spectrometric analysis.

To investigate the application potential of the U-4CR, UA-4CR and GBB-3CR for DNA-encoded library synthesis, the three reactions were performed with DNA-tagged mixtures of each four aldehyde starting materials as described in Fig. 6B–D. To simulate DEL synthesis, the starting materials were pooled, and the reaction was carried out according to the optimized conditions. These mixture experiments delivered the expected products, as the calculated masses of all products were confirmed by MALDI-TOF-MS and the reaction conversions were in the range of 80–90% for the GBB-3CR (Table S14) and the U-4CR (Table S19), and approximately 80% for the UA-4CR (Table S24).

For further proof of the compatibility of IMCR chemistry with the DNA-barcoding process, we ligated primer sequences to exemplary 7dATC-tagged GBB-3CR-, U-4CR-, and UA-4CR-products, amplified the DNA barcode by PCR, performed qPCR analysis (Fig. S67–S75) and sequenced the amplicon by Sanger sequencing (Fig. 6D, S76 and S77). The sequencing result showed that the barcode was intact. Thus, the reaction conditions of the IMCRs did not lead to nucleobase deamination, a lesion that can be detected better by sequencing than by mass spectrometric analysis. The successful DNA barcode ligation, amplification and sequencing results in combination with the high-yielding IMCR chemistry gave experimental evidence for the utility of the copolymer reaction system for DNA-encoded libraries that are designed by IMCR chemistry.

## Conclusions

In this work, we present a conceptually novel approach to DNA solubilization in pure organic solvents that exploited the ternary interaction of a poly(*N*,*N*-dimethylacrylamide)–poly(*n*-butyl acrylate) copolymer devoid of any canonical DNA-binding substructures, selectable ammonium salts and DNA oligomers. DNA oligomers of up to 80 nucleotides length were solubilized by this interaction in pure dichloromethane, chloroform, toluene, and in several non-aqueous co-solvents. In these solvents the DNA oligomers interacted tightly with the copolymer/amine combination and induced the formation of DNA–copolymer aggregates whose size correlated with DNA sequence length. As an application case, the copolymer system was used to facilitate the DNA-compatible translation of three isocyanide multicomponent reactions to DNA-tagged substrates in an operationally simple manner. The reactant scope included polar and nonpolar substrates, substrates with low reactivity and substrates with functionalities for combinatorial library synthesis. The target compounds were in nearly all cases furnished with excellent yields, meeting the stringent demands of DNA-encoded library synthesis. Importantly, the copolymer system did not interfere with downstream DEL operations such as enzymatic DNA tag ligation and barcode amplification.

The DNA solubilization method that we call CECOS “copolymer-mediated encoded chemistry in organic solvents” encourages further research in the design of ternary systems consisting of a nucleic acid oligomer, a counterion and a (co)polymer to broaden the solvent scope for nucleic acid solubilization. Currently, we assume based on the NMR and TEM analyses that the aggregates are composed of a core-like structure that contains the polar polyacrylamide part of the polymer, the DNA oligomer, and the amine, while a shell-like structure surrounding the core is formed by the hydrophobic poly-butyl ester. This hypothesis of the aggregate structure might explain the exclusive solubility of the aggregates in non-polar solvents. Investigations in the hydrophilic–lipophilic balance of the copolymer will be done to improve our understanding of the aggregates and possibly extent the solvent scope. CECOS offers visible potential for reaction development and application in DEL synthesis. As the copolymer system does not require tailored DNA barcodes or substrates and tolerated 6 nt- to 80 nt-long DNA oligomers, it is likely compatible with different DNA-barcoding strategies. The copolymer system may be used in further applications that require nucleic acids to be dissolved in organic solvents.

## Author contributions

A. B., R. W., A.-C. P. and B. B. supervised the research, analyzed the data, and wrote the manuscript with the help of all co-authors. J. B. performed solubilization experiments and analyzed DNA-polymer aggregates by DLS, J. B., E. M., L. L. and M. A. M. performed MCR chemistry with DNA-tagged starting materials, C. Z. performed DNA tagging experiments, W. L. synthesized the polymer, M. M. analysed DNA polymer aggregates by NMR, J. I. B. analyzed DNA polymer aggregates by TEM.

## Conflicts of interest

There are no conflicts to declare.

## Supplementary Material

SC-OLF-D5SC06782K-s001

## Data Availability

The data supporting this article have been included as part of the supplementary information (SI). Supplementary information: synthesis and characterization of the copolymer, DNA solubilization experiments, characterization of DNA–copolymer aggregates, reactions with DNA-tagged starting materials, characterization of DNA-conjugates, and DNA ligation, amplification and sequencing experiments. See DOI: https://doi.org/10.1039/d5sc06782k.
