# Supplementary material for: Aggregation of DNA oligomers with a neutral polymer facilitates DNA solubilization in organic solvents for DNA-encoded chemistry
Source: Chem Sci. 2025 Oct 28;16(46):21781–90. doi: 10.1039/d5sc06782k (PMC12560349; doi:10.1039/d5sc06782k)
Supplement: SC-016-D5SC06782K-s001 [file SC-016-D5SC06782K-s001.pdf]

Supplementary Information (SI) for Chemical Science.
This journal is © The Royal Society of Chemistry 2025

Supporting information

Aggregation of DNA oligomers with a neutral polymer facilitates DNA solubilization in organic solvents for DNA-encoded chemistry

Johannes Bingold,[a] Erik Mafenbayer,[a] Wibke Langenkamp,[b] Lisa Liang,[a] Chun Zhang,[a] Malte Mildner,[c] Julia Isabel Bahner,[d,e] Mohamed Akmal Marzouk,[a] Bettina Böttcher,[d,e] Ann-Christin Pöppler,[c] Ralf Weberskirch,[b] Andreas Brunschweiger*[a]

* Corresponding author

[a] Julius-Maximilians-Universität Würzburg, Institute of Pharmacy and Food Chemistry, Am Hubland, 97074 Würzburg, Germany
E-mail: andreas.brunschweiger@uni-wuerzburg.de

[b] Department of Chemistry and Chemical Biology, Polymer Hybrid Systems, TU Dortmund University, Otto-Hahn Straße 6, 44227 Dortmund, Germany

[c] Julius-Maximilians-Universität Würzburg, Institute of Organic Chemistry, Am Hubland, 97074 Würzburg, Germany

[d,e] Julius-Maximilians-Universität Würzburg, Rudolf-Virchow-Center, Josef-Schneider-Straße 2, 97080 Würzburg, Germany; Julius-Maximilians-Universität Würzburg, Biocenter, Am Hubland, 97074 Würzburg, Germany

Table of contents

Materials and methods 4

Copolymer synthesis 8

Representative synthesis procedures on DNA-tagged starting materials 10

Solubilizing DNA in organic solvents 15

DNA extration/ precipitation from organic solvents 19

UV/Vis spectra of DNA solubilized in organic solvents 20

DLS measurements 22

DOSY-NMR 28

Negatively stained Transmission Electron Microscopy analysis of particles formed by DNA oligomers and the copolymer 31

Amide Coupling of Carboxylic Acid to CPG-Bound Oligonucleotide (10mer TC) 34

Ugi four-component reaction 42

- HPLC traces of representative examples of crude U-4CR products 109

Ugi-azide four-component reaction 126

- HPLC traces of representative examples of crude UA-4CR products 157

Groebke-Blackburn-Bienaymé three-component reaction 171

- HPLC traces of representative examples of crude GBB-3CR products 202

IMCRs on different DNA sequences 218

- Amide Coupling of Carboxylic Acid to CPG-Bound Oligonucleotide (14mer 7dATC) 218
- Amide Coupling of Carboxylic Acid to CPG-Bound Oligonucleotide (14mer ATGC) 219
- Ugi four-component reaction with 14mer 7dATC-coupled aldehydes 220
- Ugi four-component reaction with 14mer ATGC-coupled aldehydes 226
- Ugi four-component reaction with a mixture of 14mer 7dATC-coupled aldehydes 232
- Ugi-azide four-component reaction with 14mer 7dATC-coupled aldehydes 235
- Ugi-azide four-component reaction with 14mer ATGC-coupled aldehydes 241
- Ugi-azide four-component reaction with a mixture of 14mer 7dATC-coupled aldehydes 246

Groebke-Blackburn-Bienaymé three-component reaction with 14mer 7dATC-coupled aldehydes 248

Groebke-Blackburn-Bienaymé three-component reaction with 14mer ATGC-coupled aldehydes 255

Groebke-Blackburn-Bienaymé three-component reaction with a mixture of 14mer 7dATC-coupled aldehydes 261

Quality assessment of DNA barcodes after multicomponent reactions by qPCR, PCR and Sanger sequencing........ 263

Materials and methods

Reagents. Unless otherwise noted, chemicals were purchased from Sigma-Aldrich (Taufkirchen, Germany), Thermo Fisher Scientific (Karlsruhe, Germany), and VWR (Langenfeld, Germany). All DNA oligomers used for solubilization experiments were synthesized by Sigma-Aldrich Co. LLC (Munich, Germany). The 10mer TC, the ATC and ATGC oligonucleotides attached to controlled pore glass solid phase (CPG, 1000 Å) were synthesized by Ella Biotech GmbH (Fürstenfeldbruck, Germany). Controlled pore glass solid phase was filtered on a synthesis column plugged onto a vacuum manifold (Vac-Man®, Promega).

Analytical RP-HPLC. Oligonucleotide-small molecule conjugates were analyzed by ion-pair reverse-phase high-performance liquid chromatography (RP-HPLC, Agilent 1260 Infinity II) using a C_{18} stationary phase (Phenomenex, Gemini; 5 µm, C18, 110 Å, 100*4.6 mm). A gradient from 100 mM aqueous triethylammonium acetate (pH = 8.0, eluent A) to MeOH (eluent B) was used at a flow rate of 1.2 mL/min.

Method. Linear gradient of 20% to 80% B within 8 min, then 80% to 100% B within 0,5 min, followed by 100% B for 2 min, then 80% to 20% B within 2 min, followed by 20% B for 2 min. HPLC chromatograms were recorded at 260 and 280 nm wavelengths.

Semi-preparative RP-HPLC. Oligonucleotide-small molecule conjugates were purified by semi-preparative RP-HPLC (Agilent 1260 Infinity II) using a C_{18} stationary phase (Phenomenex, Gemini; 5 µm, C18, 110 Å, 100*10 mm). A gradient from 100 mM aqueous triethylammonium acetate (pH = 8.0, eluent A) to MeOH (eluent B) was used at a flow rate of 4 mL/min.

Method. Linear gradient of 20% to 80% B within 8 min, then 80% to 100% B within 0.5 min, followed by 100% B for 2 min, then 80% to 20% B within 2 min, followed by 20% B for 2min. HPLC chromatograms were recorded at 260 and 280 nm wavelengths. Fractions containing the desired products were concentrated by SpeedVac.

Oligonucleotide concentrations. Oligonucleotide concentrations were determined by UV spectroscopy using a spectrophotometer (NanoPhotometer N120, Implen, Germany) and Qubit™ Flex Fluorometer (Thermo Fisher Scientific™).

MALDI-TOF. Oligonucleotides were analyzed by MALDI-MS (Bruker UltrafleXtreme) using 3-Hydroxypropionic acid (3-HPA) matrix (Dichrom).

LC-MS. Oligonucleotides were analyzed by LC-MS (Agilent 1260 Infinity II) using a C_{18} stationary phase (Phenomenex, Gemini; 3 µm, C18, 110 Å, 100*2 mm). A gradient from an aqueous mixture

of 100mM Hexafluoroisopropanol (HFIP) and 15mM triethylammonium acetate buffer (eluent A) and MeOH (eluent B) was used at a flow rate of 0.5 mL/min.

Method. Linear gradient of 10% to 50% B within 3 min, then 50% to 95% B within 6 min, followed by 95% B for 1 min, then 95% to 10% B within 5 min. HPLC chromatograms were recorded at 260 and 280 nm wavelengths. MS spectra were simultaneously recorded.

DMT-cleavage: The DMT-protective group of an aminolinker-modified DNA strand bound to CPG solid support was removed by addition of 200 µL 3% trichloroacetic acid in CH_2Cl_2 for 1 min. Orange coloring of the solution indicated successful removal of protecting group. The deprotection was repeated 4-5 times until no further coloring of the solution was observed. CPG-bound deprotected DNA was washed three times with each 200 µL of 1% TEA in ACN, DMF, MeOH, ACN and CH_2Cl_2 and then dried in vacuo.

DOSY-NMR

^{1}H NMR spectra and ^{1}H Diffusion Ordered spectroscopy (^{1}H DOSY) data were recorded on a Bruker Avance Neo 400 MHz spectrometer, equipped with a BBFO 5 mm probe containing a z-axis gradient coil. The chemical shifts were referenced to the respective residual proton solvent signal.

DOSY spectra were recorded using a “ledbpg2s” sequence at a temperature of 298 K. The diffusion gradients were linearly incremented in 32 steps from 2% to 98% with a maximum gradient strength of 50 G/cm. All experiments were conducted with an eddy current delay of 5 ms and a recycle delay of 3.2 ms. A diffusion time of 50 ms and a pulse duration of 3.33 ms were used to reach a signal attenuation of 1% for all samples. Only the hexa-T signal for the 100 nmol sample did not fully decay. Diffusion coefficients were obtained by mono- or biexponential fitting of the signal intensity of selected signals using Origin Pro 2020 software (OriginLab® Northampton, Massachusetts, USA).

Dynamic light scattering (DLS) measurements

DNA oligomers were dissolved as described above in 200µL of either toluene or chloroform. DLS measurements of the ternary DNA-amine-copolymer complexes were performed on a Zetasizer (Zetasizer Ultra, Malvern Panalytical, UK) at 25°C.

UV/Vis spectra

DNA oligomers were dissolved as described above in 200µL of either dichloromethane or chloroform. UV/Vis spectra of solubilized DNAs were measured on a spectrophotometer (JASCO V-730, JASCO, Japan) at room temperature.

Negatively stained transmission electron microscopy (TEM)

Samples were directly applied to glow-discharged, carbon-coated copper grids, and negatively stained with 2% uranyl acetate. The grids were imaged using a Tecnai T12 transmission electron microscope (Thermo Fisher Scientific) operated at 120 kV, equipped with a tungsten filament and an Eagle CCD camera. Images were acquired under low-dose conditions at a magnification of 52000× (pixel size: 4.402 Å/pixel), with a total electron exposure of 30 $e^{-}/Å^{2}$ and a targeted underfocus of 1 µm.

FAM-labeled DNA sequences

FAM-labeled DNA oligomers used for solubilization experiments were synthesized by Sigma-Aldrich Co. LLC (Munich, Germany).

10mer ATGC

5'-[6-FAM]-CTACATGTGC-3'

20mer ATGC

5'-[6-FAM]-CTACATATCCGAGCTGGAGT-3'

40mer ATGC

5'-[6-FAM]-CTACATATCCTGCATCCTGTCCTAGGTGCTGCCATAACCA-3'

60mer ATGC

5'-[6-FAM]-CTACATATCCGAGCTGGAGTCTACATATCCTGCATCCTGTCCTAGGTGCTGCCATAAC C-3'

80mer ATGC

5'-[6-FAM]-CTACATATCCTGCATCCTGTCCTAGGTGCTGCCATAACCACTACATATCCTGCATCCTGTC CTAGGTGCTGCCATAACCA- 3'

DNA sequences for IMCR chemistry

10mer TC, 14mer ATGC and 14mer chemically stabilized ATC were synthesized by Ella Biotech (Germany) and delivered coupled to CPG beads with 5'-DMT-on.

10mer TC

5'-Amino-C6-TTCCTCTCCT-3'

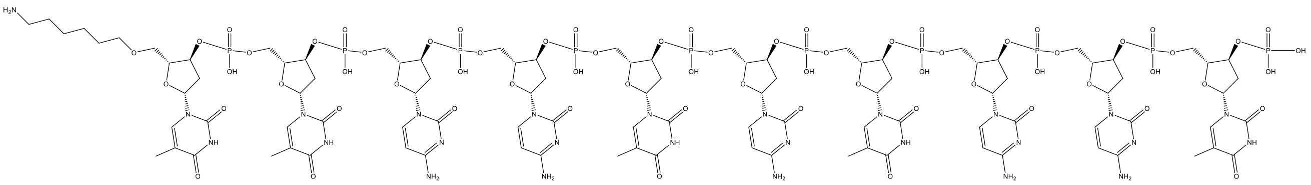

14mer ATGC

5'-Amino-C6-CTACGTATGTGACC-3'

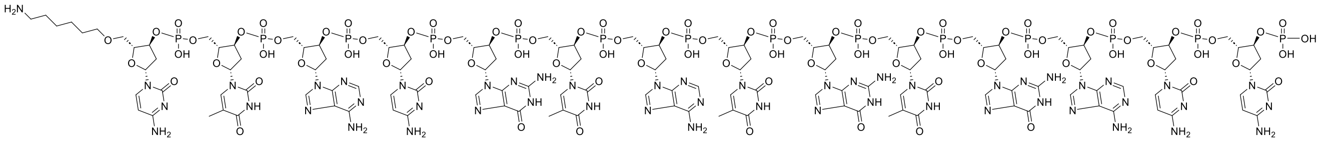

5'-Amino-C6-GTCTTGCCGAATTC-3'

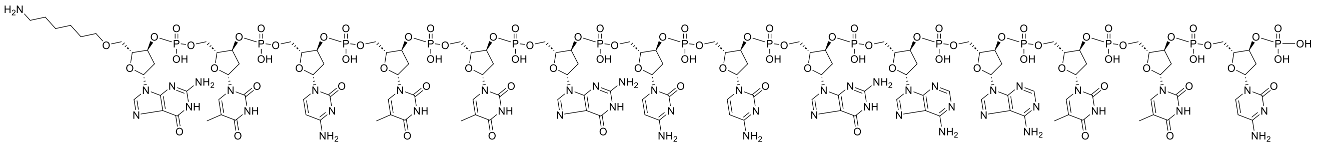

14mer chemically stabilized ATC

5'-Amino-C6-CTACATA**X**CTATCC-3'(**X** = 5-Ethylnyl-dU-CEP, A = 7-deaza dA)

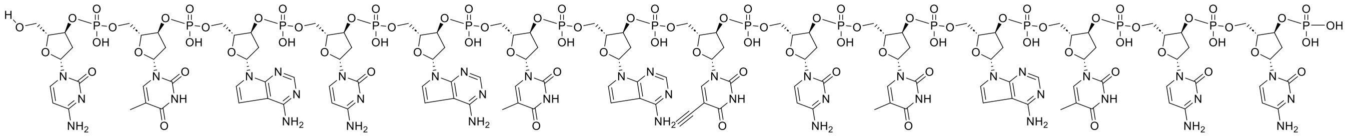

Copolymer synthesis

Synthesis of the hydrophilic polymer block

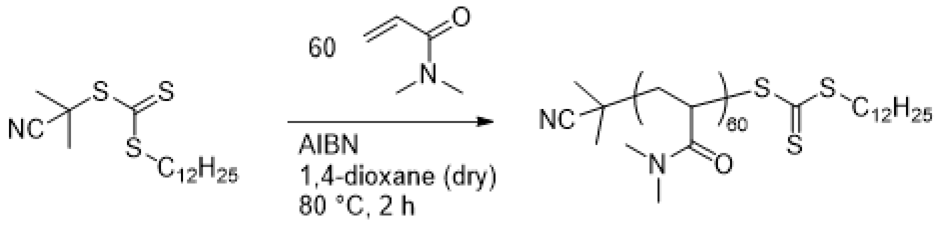

*N,*N-dimethylacrylamide (60 eq.), 2-cynano-2-propyldodecyltrithiocarbonate (1 eq.), and 2,2'-azobis(isobutyronitrile) (0.05 eq.) were dissolved in 12 mL of dry 1,4-dioxane in a Schlenk tube. The mixture was perfused with argon for 30 min and polymerised at 80 °C for 2h. The solvent was then removed under reduced pressure and the residue was taken up in 8 mL dichloromethane. The polymer was precipitated in about 800 mL of cold diethyl ether and then obtained after filtration. The polymer was dried under high vacuum and then isolated as a yellow solid.

Production of the hydrophobic part

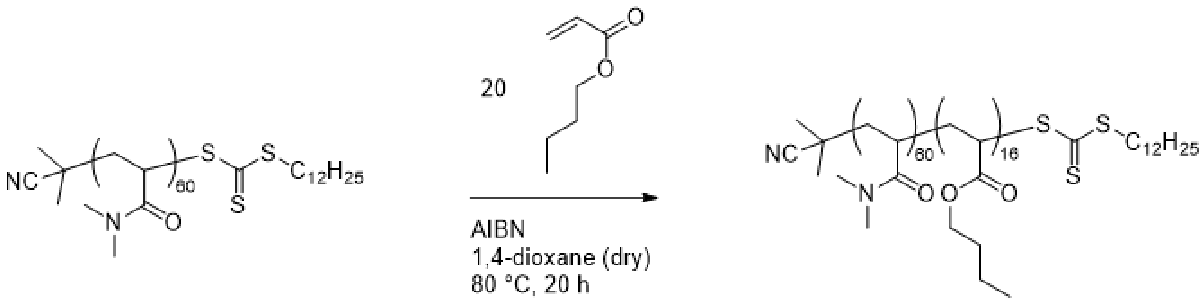

PDMA (1 eq.), n-butyl *acrylate* (20 eq.) and 2,2'-azobis(isobutyronitrile) (0.05 eq.) are dissolved in dry 1,4-dioxane in a Schlenk tube. The solution is stirred until the polymer is completely dissolved and then perfused with argon for 30 min. Polymerisation was carried out at 80 °C for 20 hours. The solvent was removed under reduced pressure and the residue was taken up in 5 mL dichloromethane. The polymer was precipitated in 600 mL of cold diethyl ether and pentane (v/v = 1:1) and obtained after solvent removal. After final drying under high vacuum, the polymer is obtained as a yellowish solid.

End group splitting

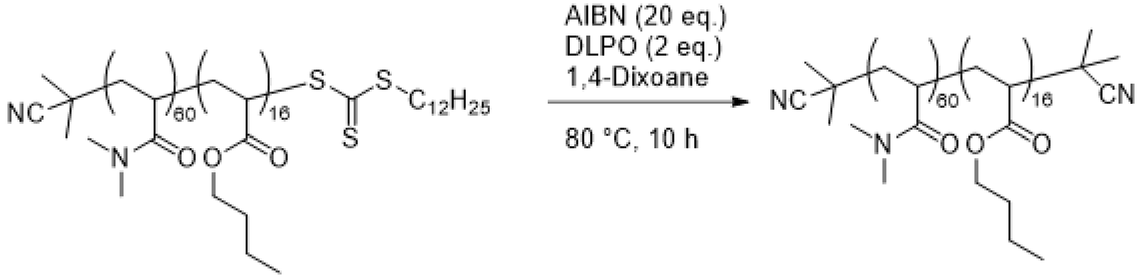

The block copolymer (1 eq.), 2,2'-azobis(isobutyronitrile) (20 eq.) and dodecanoyl peroxide (2 eq.) are dissolved in dry dioxane in a heated Schlenk tube. The solution is degassed with argon and stirred at 80 °C for 10 hours. The reaction is then terminated in liquid nitrogen and the solvent is removed under reduced pressure. The residue is taken up in a little dichloromethane and precipitated in a cold mixture of diethyl ether and pentane (v/v = 1:1). The resulting white solid is dried under high vacuum. The complete cleavage of the RAFT group was confirmed for all polymers via UVVis.

**Block copolymer**	**Composition**	$M_{(n, NMR)}$ [a] **[g/mol]**	$d_{(h, H2O)}$ [b] **[nm]**	$d_{(h,MeOH)}$ [b] **[nm]**	$d_{(h,CH3Cl)}$ [b] **[nm]**	$d_{(h,toluene)}$ [b] **[nm]**
**WL-25c**	DMA_{60}-$BuA_{(1)\ (6)}$	8134	44± 0.4	5± 0.5	-	-
**WL-52c**	DMA_{60}-BuA_{17}	8263	29 2±	2 ± 0,3	0.14± 0.05	6 ± 3
**WL-100c**	$DMA_{56\text{-}}BuA_{19}$	8122	35± 0.9	28± 4	20 1±	18 ± 3

a) Determined from the ^{1}H-NMR spectrum in $CDCl_3$; b) determined via DLS measurements of a 1mM polymer solution in the respective solvent listed.

Representative synthesis procedures on DNA-tagged starting materials

General procedure 1, amide coupling reaction on CPG beads

The CPG-bound oligonucleotides (ca. 90 nmol, 3 mg solid phase material), the carboxylic acid and HATU were dried *in vacuo* for 15 min. Stock solutions of all reactants in dry DMF were prepared before the reaction was started. To the solution of the acid (100 equiv.) in 170 µL dry DMF were added HATU (100 equiv.) dissolved in 40 µL dry DMF and DIPEA (250 equiv.). The mixture was shaken for 10 min at 37°C and added to the CPG-bound oligos. The amide coupling reaction was shaken at 37°C for 1 h. Then, the CPG solid phase was filtered over a filter column, washed with each 3x 200 µL of DMF, MeOH, ACN and CH_2Cl_2 and dried in vacuo. The amide coupling was repeated twice.

General procedure 2, capping after amide coupling reactions

Unreacted amines were capped with acetic acid anhydride (three times 30 s reaction time, 200 µL of a 1:1 mixture of THF/methylimidazole, 9:1, vol/vol, and THF/pyridine/acetic acid anhydride, 8:1:1, vol/vol). The capped CPG-bound oligonucleotide conjugate was washed three times with each 200 µL of DMF, MeOH, ACN and CH_2Cl_2 and dried *in vacuo*.

General procedure 3, Ugi four-component reaction

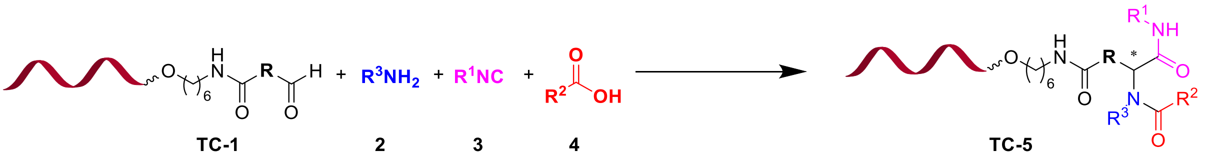

A DNA-aldehyde conjugate **TC-1** (3 nmol, 1 equiv.) was solubilized with the help of the copolymer (90 nmol, 30 equiv.) in 50 µL of a mixture of $CHCl_3$/MeOH=1:3, v/v. Then, an amine **2** (6 µmol, 2000 equiv.) was added to the oligonucleotide-copolymer mixture. If the amine was liquid, it was added directly to the DNA. Solid amines (6 µmol, 2000 equiv.) were dissolved in MeOH (10 µL) and added to the DNA. In this case, the DNA **TC-1** was dissolved in 40 of a mixture of $CHCl_3$/MeOH=1:2.2, v/v, so that after addition of the amine a final volume of 50 µL of a mixture of $CHCl_3$/MeOH=1:3, v/v resulted. The mixture was shaken at ambient temperature for 3 h to effect imine formation. Afterwards, the acid **4** (6 µmol, 2000 equiv.) and isocyanide **3** (6 µmol, 2000 equiv.) were added. Liquid reagents were added directly by pipetting, and solid reagents were dissolved each in 5 µL of MeOH and added to the reaction, which brought the final volume to 60 µL of a $CHCl_3$/MeOH=1:4, v/v mixture. The mixture was shaken for 16 h at 50°C. Then the solvent was evaporated to dryness. The product **TC-5** was purified by RP-HPLC and analysed by analytical HPLC and MALDI-MS.

General procedure 4, Ugi-azide four-component reaction

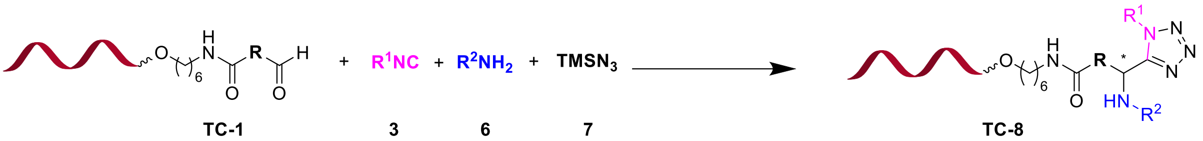

The DNA-aldehyde conjugate **TC-1** (3 nmol, 1 equiv.) was solubilized with the help of the copolymer (90 nmol, 30 equiv.) in 50 µL of a mixture of $CHCl_3$/MeOH=1:3, v/v. Then, a secondary amine **6** (6 µmol, 2000 equiv.) was added to the oligonucleotide-copolymer mixture. If the amine was liquid, it was added directly to the DNA. Solid amines (6 µmol, 2000 equiv.) were dissolved in MeOH (10 µL) and added to the DNA. In this case, the DNA **TC-1** was dissolved in 40 of a mixture of $CHCl_3$/MeOH=1:2.2, v/v, so that after addition of the amine a final volume of 50 µL of a mixture of $CHCl_3$/MeOH=1:3, v/v resulted. The mixture was shaken at ambient temperature for 3 h to effect imine formation. Afterwards, isocyanide **3** (6 µmol, 2000 equiv.) and $TMSN_3$ **7** (6 µmol, 2000 equiv.) were added directly by pipetting. Solid isocyanides were dissolved in 5 µL of MeOH and added to the reaction, which brought the final volume to 55 µL of a $CHCl_3$/MeOH=1:3.5, v/v mixture. The mixture was shaken for 16 h at 50°C and the solvent was evaporated *in vacuo*. The product **TC-8** was first purified by RP-HPLC and later analysed by analytical HPLC and MALDI-MS.

General procedure 5, Groebke-Blackburn-Bienaymé three-component reaction

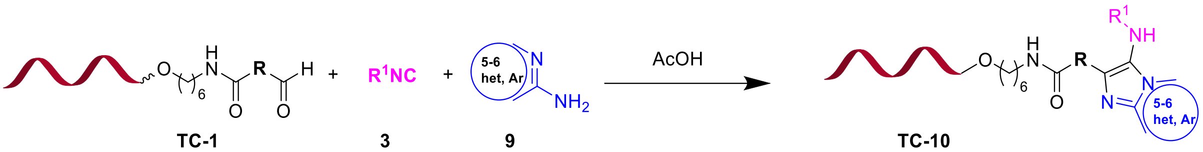

The DNA-aldehyde conjugate **TC-1** (3 nmol, 1 equiv.) was solubilized with the help of the copolymer (90 nmol, 30 equiv.) in 70 µL of a mixture of $CHCl_3$/MeOH=1:6, v/v. Then, the hetarylamine **9** (6 µmol, 2000 equiv.) was dissolved in 10 µL MeOH and added to the DNA to give a final volume of 80 µL (MeOH/$CHCl_3$=7:1, v/v). The reaction was shaken at ambient temperature for 6 h to effect imine formation. Afterwards, isocyanide **3** (6 µmol, 2000 equiv.) and acetic acid (0.8 µL, final c = ca. 1%) were added directly by pipetting. Solid isocyanides were dissolved in 5 µL of MeOH and added to the reaction, which brought the final volume to 85 µL of a $CHCl_3$/MeOH=1:7.5, v/v mixture. The mixture was shaken for 16 h at ambient temperature and the solvent removed *in vacuo*. The product **TC-10** was first purified by RP-HPLC and later analysed by analytical HPLC and MALDI-MS.

General procedure 6, DNA solubilization in organic solvents

5 nmol of fluorescence-labeled DNA (10mer, 20mer, 40mer, 60mer or 80mer) was dissolved together with the copolymer (30 equiv., 150 nmol, 150 µL, from an aqueous 1 mM copolymer stock solution) in a total volume of 170 µL of deionized water, and the solvent was evaporated *in vacuo*. For solubilization experiments, the residue was then either directly used (DNA as Na^+ form) or dissolved in 1 mL of a 100 mM aqueous solution of an amine (e.g. ammonia, triethylamine, DIPEA, piperidine, pyridine or imidazole, 100 µmol amine). These solutions were evaporated to dryness for solubilization experiments.

Alternatively, ion-pair chromatography-purified DNA oligomers are isolated as triethylammonium salts. These can be directly dissolved in an aqueous stock solution of the copolymer, and the solvent can be evaporated to dryness for solubilization experiments.

Afterwards, the residue was dissolved in 40 – 200 µL of an organic solvent (DCM, chloroform, toluene), as required by the intended following experiment. To these solvents other solvents could be added to obtain co-solvent mixtures for desired reactions (e.g. MeCN/ DCM, DMF/ DCM etc.). Alternatively, DNA oligomers could be directly solubilized in solvent mixtures. Solubility of the DNA was measured/shown by UV/Vis measurement or simply by taking a photographical picture of dye-labelled DNA.

General procedure 7, DNA extration/ precipitation from organic solvents

DNA could be separated from the polymer either by extraction with NaCl solution, and precipitation or purification by preparative HPLC. For extraction, an aqueous NaCl solution was added, and the two-phase system was shaken and then centrifugated to separate the organic and aqueous phases.

For DNA precipitation, 50µL H_2O, 5 µL 3M NaOAc & 150 µL EtOH were added to the solution and then the mixture was transferred to a -80°C freezer and left overnight. Afterwards, the mixture was centrifugated and the supernatant removed. The DNA pellet was washed with 70% EtOH to remove any surplus salts.

Solubilizing DNA in organic solvents

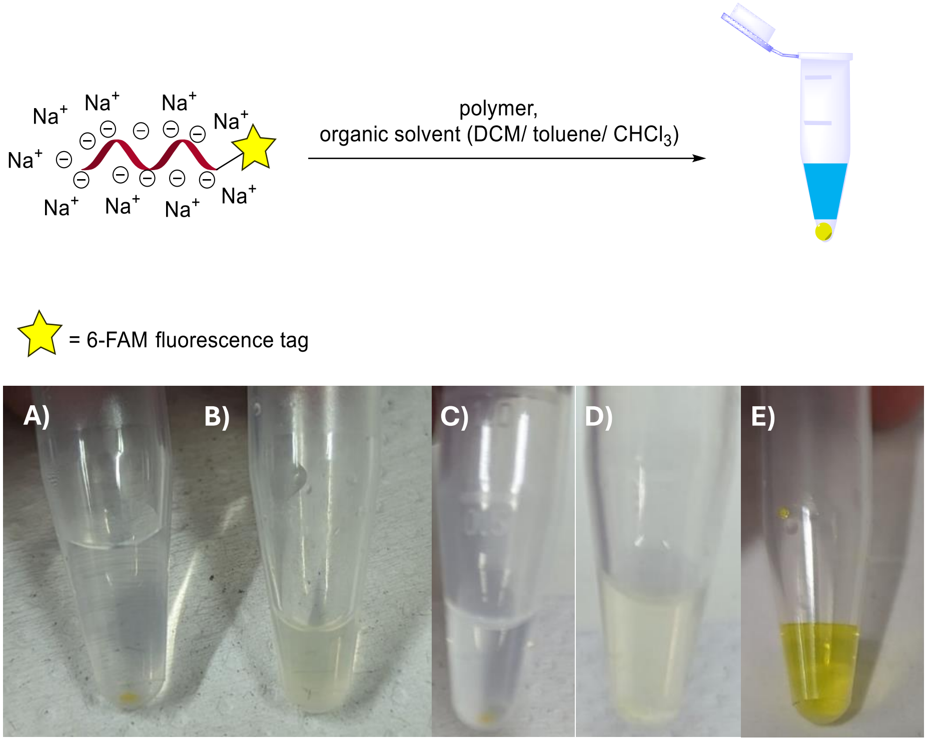

Figure S1. Experiments to dissolve a FAM-labeled 10mer DNA with Na^+-counterion in organic solvents. **A)** DNA in MeCN w/o copolymer; **B)** DNA + copolymer in MeCN (turbid mixture, after centrifugation a pellet was formed); **C)** DNA in DCM w/o copolymer; **D)** DNA + copolymer in DCM (turbid mixture, after centrifugation a pellet was formed); **E)** Control: fluorescence-labeled DNA in H_2O.

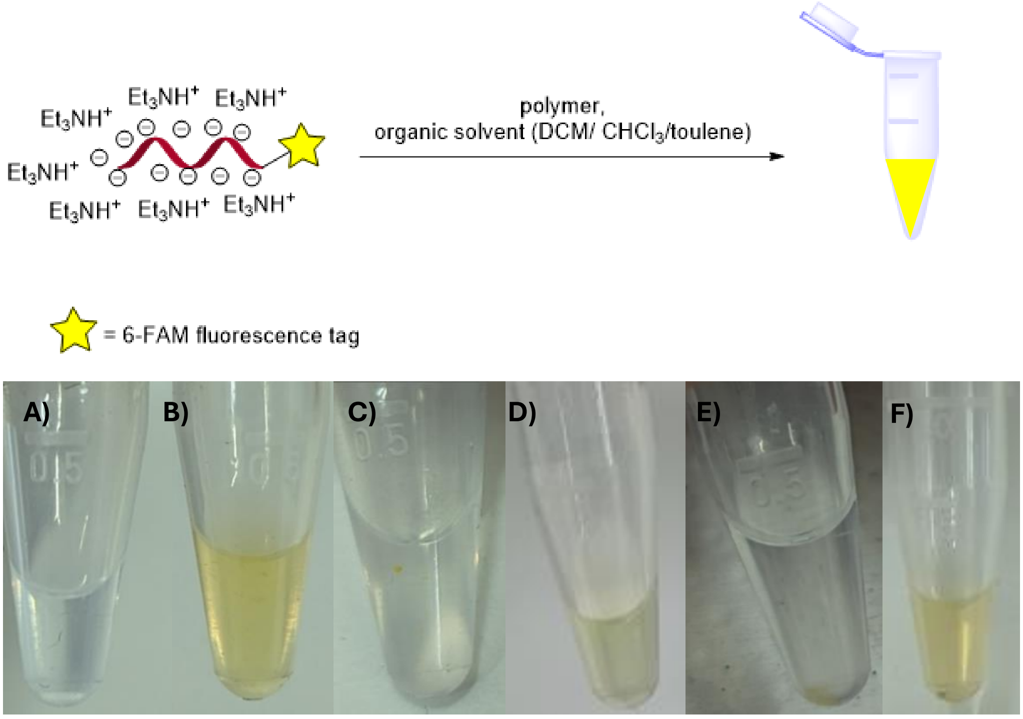

Figure S2. Solubilization of FAM-labeled DNA with Et_3NH^+-counterion in organic solvents. **A)** 10mer DNA in DCM w/o copolymer; **B)** 10mer DNA + copolymer in DCM; **C)** 10mer DNA in $CHCl_3$ w/o copolymer; **D)** 10mer DNA + copolymer in $CHCl_3$; **E)** 10mer DNA in toluene w/o copolymer; **F)** 10mer DNA + copolymer in toluene (the DNA was HPLC-purified for this experiment and used as triethyl ammonium salt).

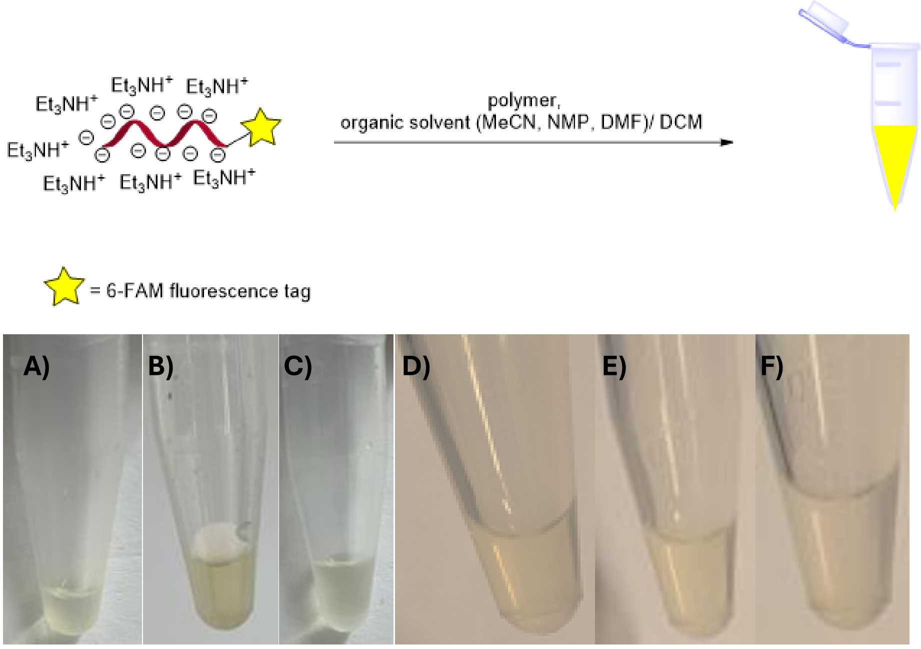

Figure S3. Solubilization of FAM-labeled 10mer DNA (Et_3NH^+ form) in co-solvents of DCM or chloroform. **A)** DNA + copolymer in MeCN/DCM = 9:1; **B)** DNA + copolymer in DMF/DCM = 1:1; **C)** DNA + copolymer in NMP/DCM = 2:1; **D)** DNA + copolymer in $Et_2O/CHCl_3$ = 2:1; **E)** DNA + copolymer in MTBE/DCM = 2:1; **F)** DNA + copolymer in $EtOH/CHCl_3$ = 2:1. All ratios given as vol/vol.

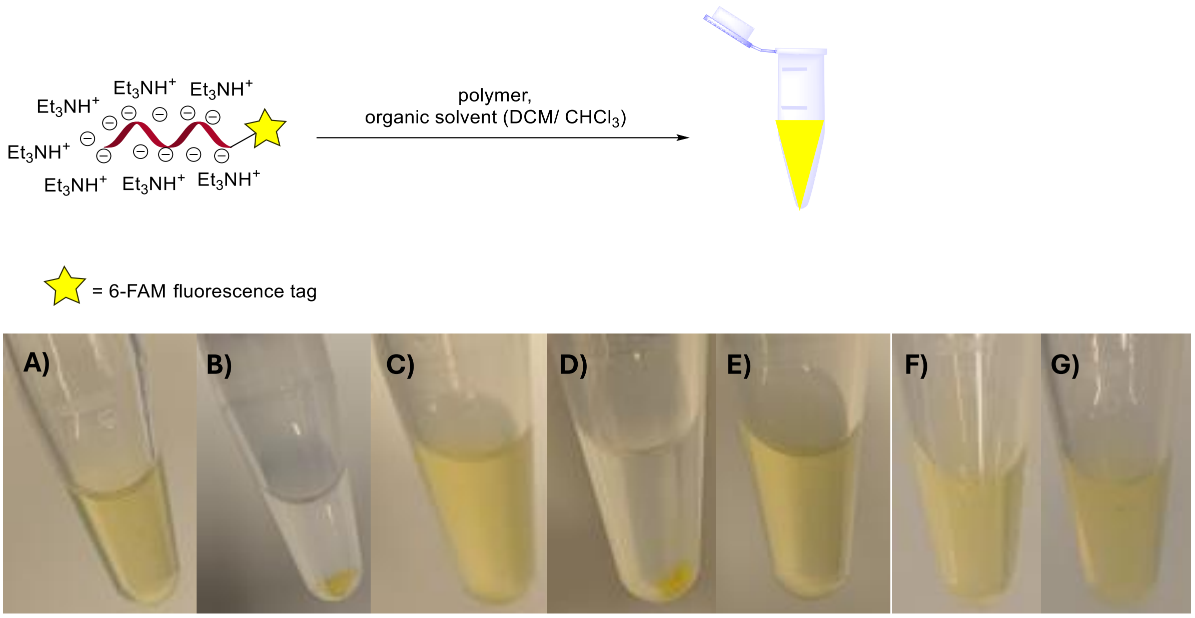

Figure S4. Solubilization of FAM-labeled 20-80mer DNA (Et_3NH^+ form) in organic solvents. **A)** 20 mer DNA + copolymer in toluene; **B)** 20mer DNA in DCM w/o copolymer; **C)** 20mer DNA + copolymer in DCM; **D)** 20mer DNA in $CHCl_3$ w/o copolymer; **E)** 20mer DNA + copolymer in $CHCl_3$; **F)** 60mer DNA + copolymer in $CHCl_3$; **G)** 80mer DNA + copolymer in $CHCl_3$.

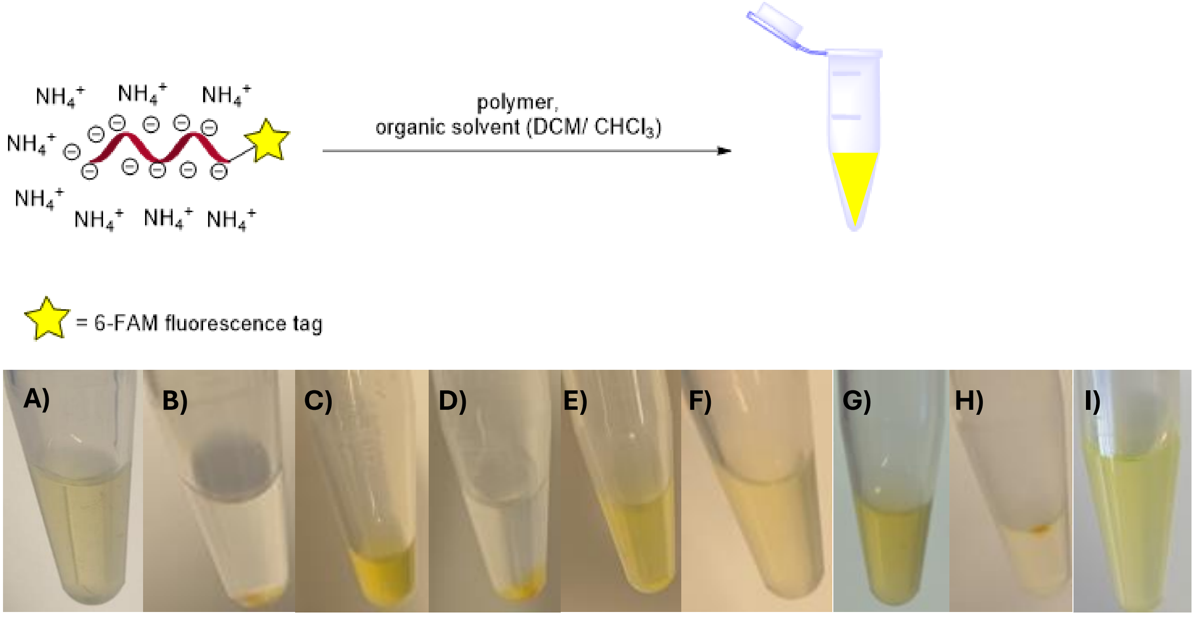

Figure S5. Solubilization of FAM-labeled DNA with different counter ions in organic solvents. **A)** 20mer DNA + copolymer + DIPEA in $CHCl_3$; **B)** 20mer DNA in DCM w/o copolymer; **C)** 20mer DNA + copolymer + NH_4^+ in DCM; **D)** 20mer DNA in $CHCl_3$ w/o copolymer; **E)** 20mer DNA + copolymer + NH_4^+ in $CHCl_3$; **F)** 20mer DNA + copolymer + pyridine in $CHCl_3$; **G)** 20mer DNA + copolymer + piperidine in $CHCl_3$; **H)** 40mer DNA + copolymer + piperidine in DCM; **I)** 20mer DNA + copolymer + imidazole in $CHCl_3$.

Table S1. Summary of DNA solubilization experiments.

Solvent	Counterion	DNA solubility
DCM, toluene	Na^+	No
DCM, $CHCl_3$	Et_3NH^+	10mer, 20mer, 40mer, 60mer, 80mer solubilized
toluene	Et_3NH^+	Residual Na^+ ions were not tolerated, stringent ion exchange was mandatory; 10mer, 20mer, 40mer solubilized
EtOH	Et_3NH^+	with DCM or $CHCl_3$ as co-solvent, 10mer, 20mer solubilized (EtOH/ $CHCl_3$ = 2:1, v/v)
MeOH	Et_3NH^+	with DCM or $CHCl_3$ as co-solvent, 10mer, 20mer solubilized (MeOH/ $CHCl_3$ = 9:1, v/v)
NMP	Et_3NH^+	with DCM or $CHCl_3$ as co-solvent, 10mer, 20mer solubilized (NMP/ $CHCl_3$ = 2:1, v/v)
DMF	Et_3NH^+	with DCM or $CHCl_3$ as co-solvent, 10mer, 20mer solubilized (DMF/ $CHCl_3$ = 1:1, v/v)
MeCN	Et_3NH^+	with DCM or $CHCl_3$ as co-solvent, 10mer, 20mer solubilized (MeCN/ $CHCl_3$ = 9:1, v/v)
Et_2O	Et_3NH^+	with DCM or $CHCl_3$ as co-solvent, 10mer, 20mer solubilized (Et_2O/ $CHCl_3$ = 2:1, v/v)
MTBE	Et_3NH^+	with DCM or $CHCl_3$ as co-solvent, 10mer, 20mer solubilized (MTBE/ $CHCl_3$ = 2:1, v/v)
DCM, $CHCl_3$	NH_4^+	10mer, 20mer, 40mer solubilized
DCM, $CHCl_3$	DIPEA	10mer, 20mer, 40mer solubilized
DCM, $CHCl_3$	pyridine	10mer, 20mer, 40mer solubilized
DCM, $CHCl_3$	imidazole	10mer, 20mer, 40mer solubilized
DCM, $CHCl_3$	piperidine	10mer, 20mer solubilized, the 40mer was not solubilized

n (DNA) = 5 nmol

DNA extration/ precipitation from organic solvents

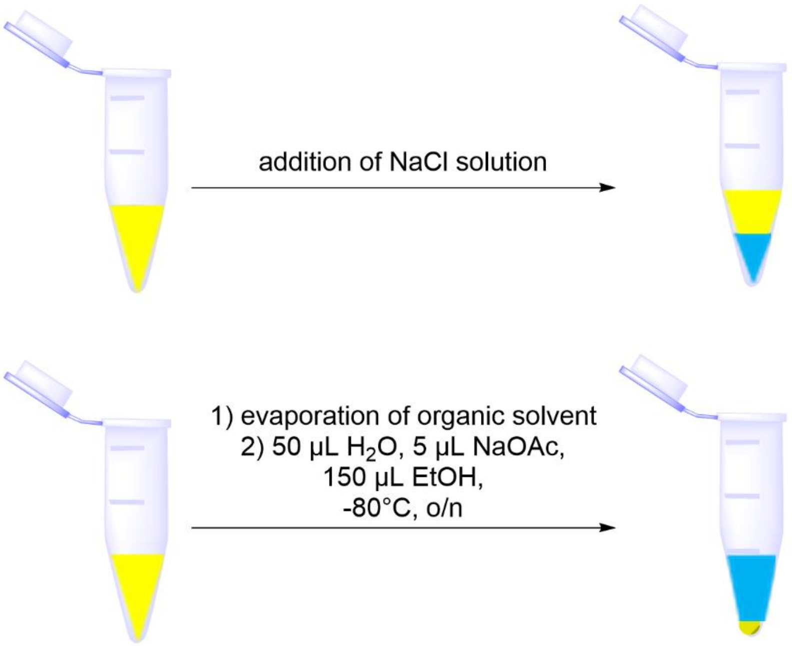

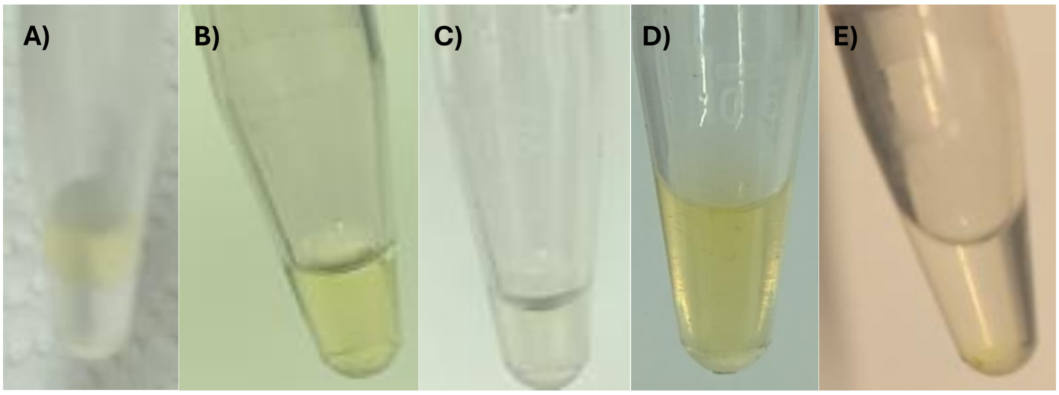

Figure S6. 20mer FAM-labelled DNA extraction with aq. NaCl solution and subsequent DNA precipitation from organic solvents. **A-C)** DNA extraction from DCM with saturated NaCl solution (**A)** both phases (organic & aqueous) in one tube; **B)** aqueous phase; **C)** organic phase); **D)** DNA + copolymer + Et_3NH^+ in DCM; **E)** DNA precipitation after DCM evaporation with 50μL H_2O, 5 μL 3M aq. NaOAc & 150 μL EtOH (overnight at -80°C).

UV/Vis spectra of DNA solubilized in organic solvents

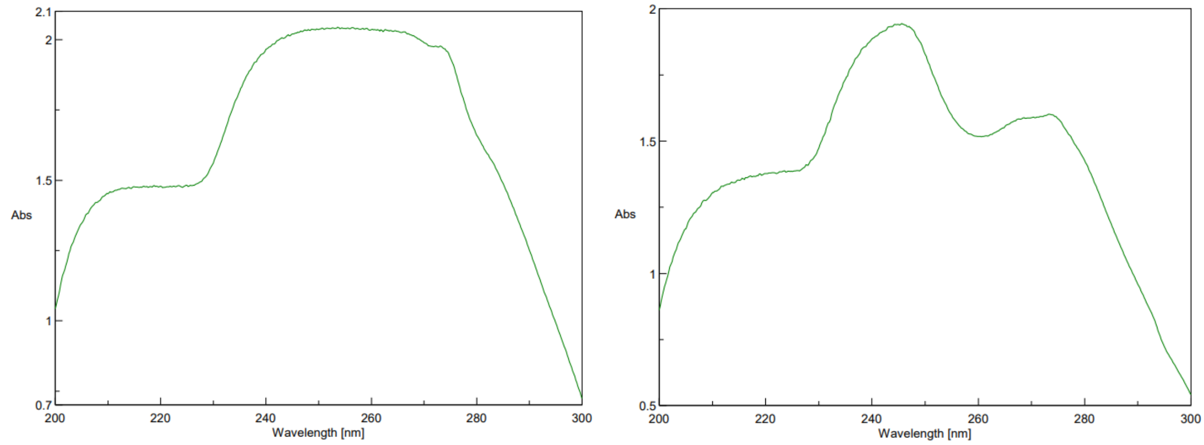

Figure S7. UV/Vis spectra of 10mer DNA + copolymer in $CHCl_3$ (left-hand spectrum) and 10mer DNA (FAM-labeled) + copolymer in $CHCl_3$ (right-hand spectrum).

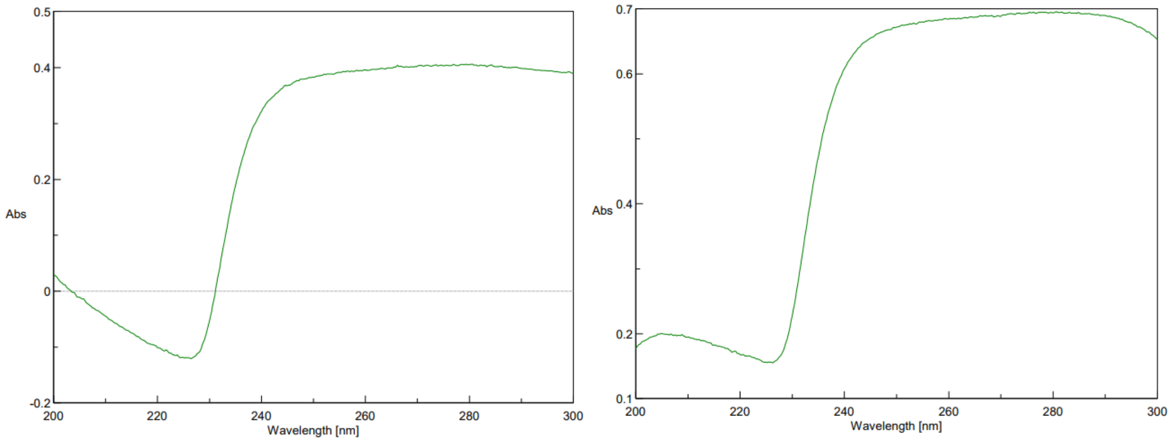

Figure S8. UV/Vis spectra of 10mer DNA + copolymer in DCM (left-hand spectrum) and 10mer DNA (FAM-labeled) + copolymer in DCM (right-hand spectrum).

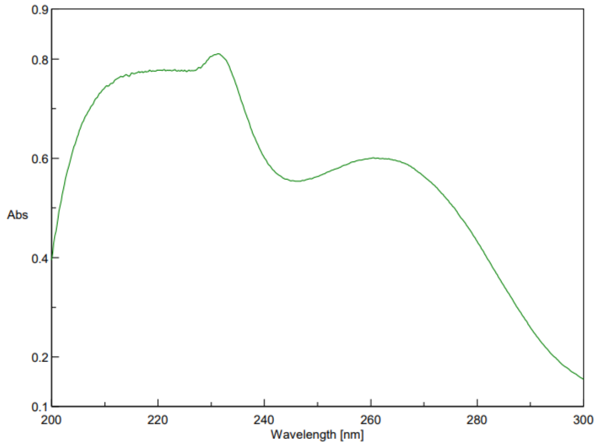

Figure S9. UV/Vis spectrum of 10mer DNA (FAM-labeled) in H_2O.

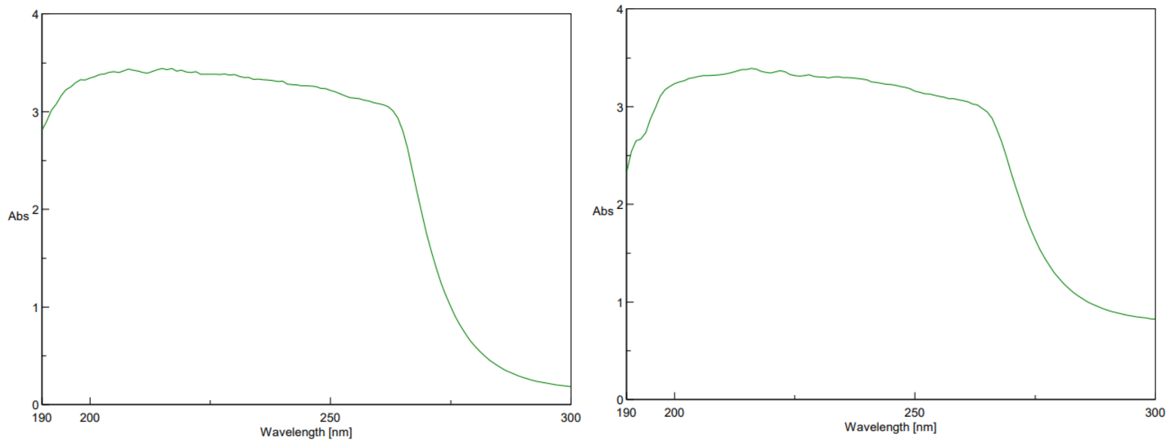

Figure S10. UV/Vis spectra of 20mer DNA (FAM-labeled) + copolymer + Et_3NH^+ in $CHCl_3$ (left-hand spectrum) and 20mer DNA (FAM-labeled) + copolymer + NH_3 in $CHCl_3$ (right-hand spectrum).

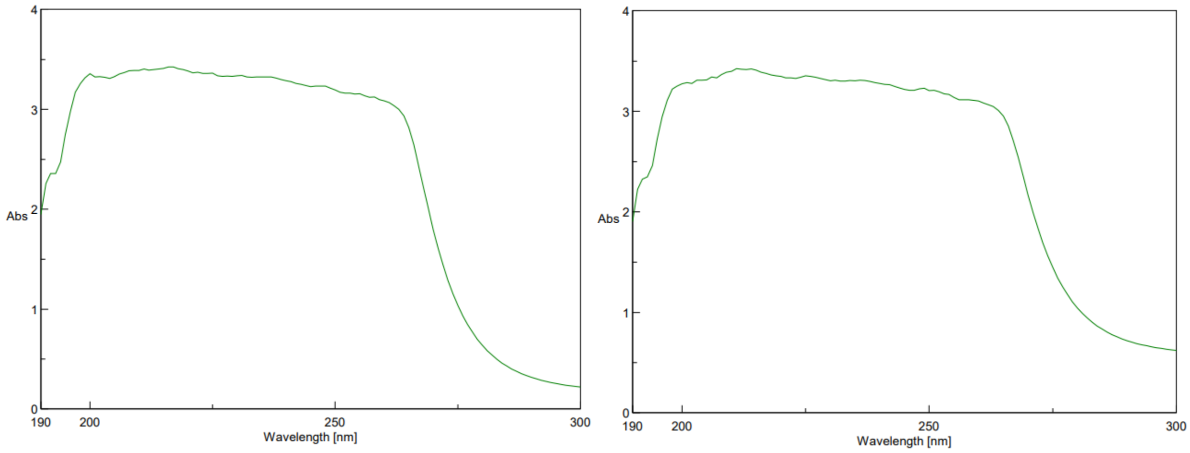

Figure S11. UV/Vis spectra of 20mer DNA (FAM-labeled) + copolymer + DIPEA in $CHCl_3$ (left-hand spectrum) and 20mer DNA (FAM-labeled) + copolymer + pyridine in $CHCl_3$ (right-hand spectrum).

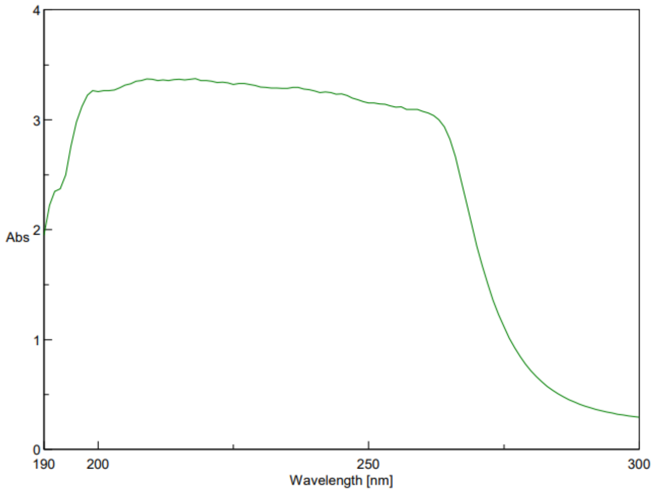

Figure S12. UV/Vis trace of 20mer DNA (FAM-labeled) + copolymer + piperidine in $CHCl_3$.

DLS measurements

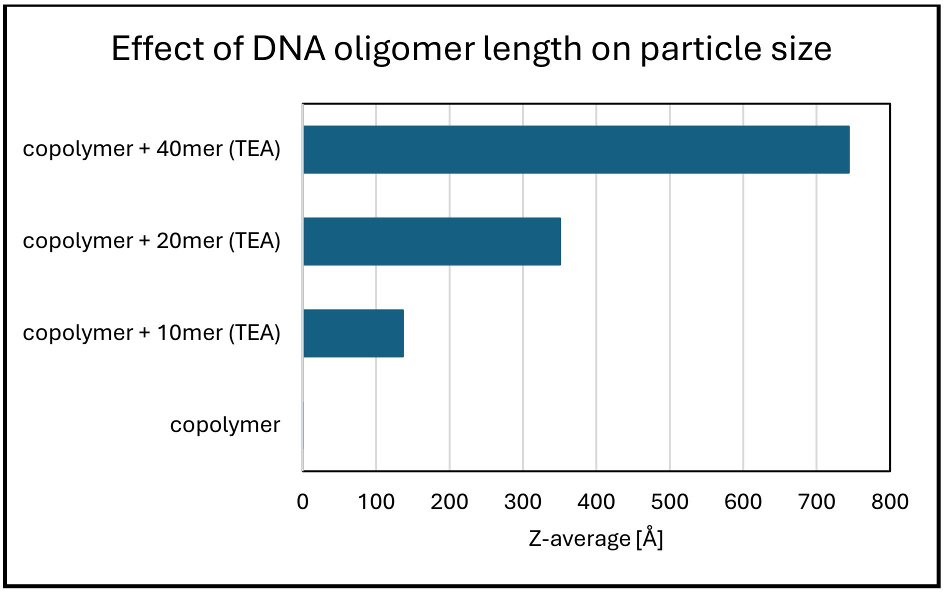

Figure S13. DLS measurement of particles formed by DNA-copolymer interaction in $CHCl_3$. Effect of DNA sequence length on particle size.

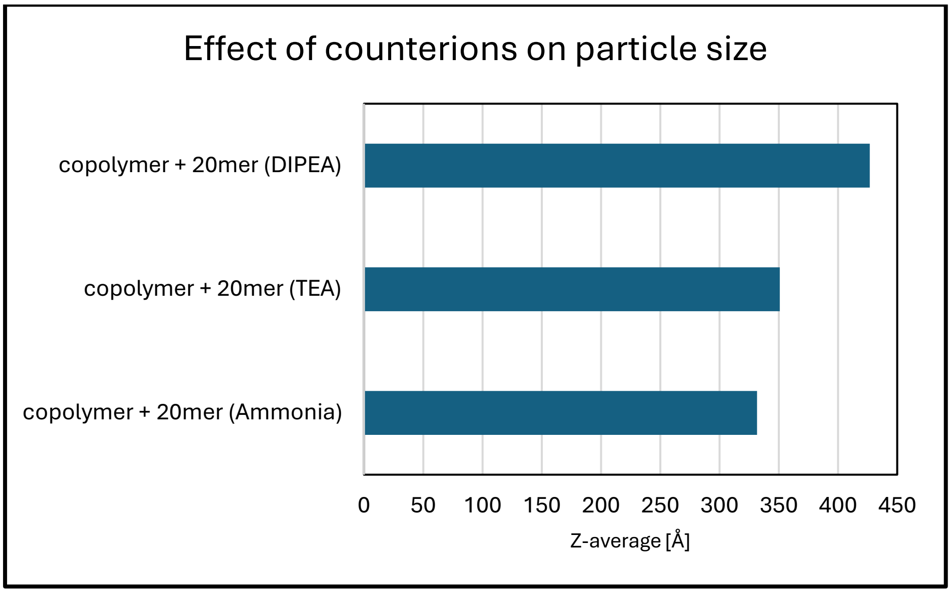

Figure S14. DLS measurement of particles formed by DNA-copolymer interaction in $CHCl_3$. Effect of DNA counterions on particle size.

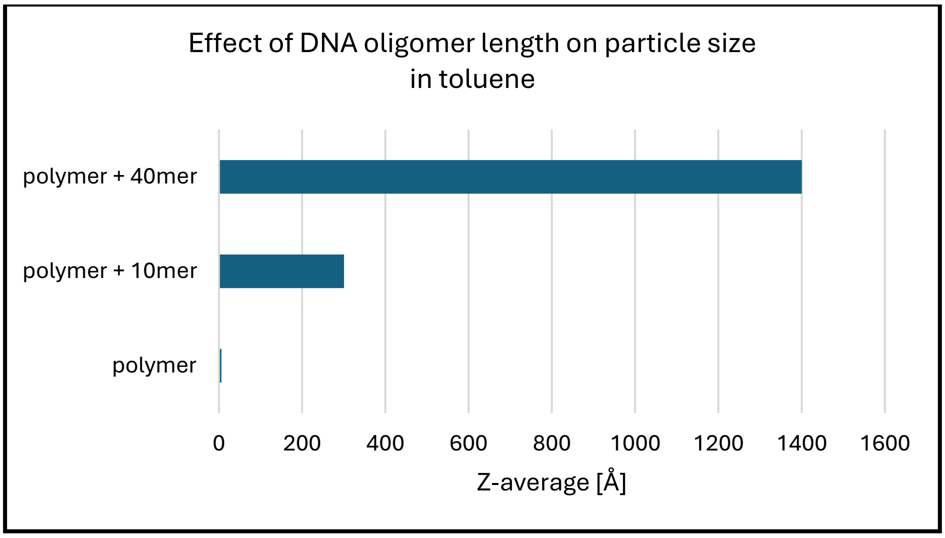

Figure S15. DLS measurement of particles formed by DNA-copolymer interaction in toluene. Effect of DNA sequence length on particle size.

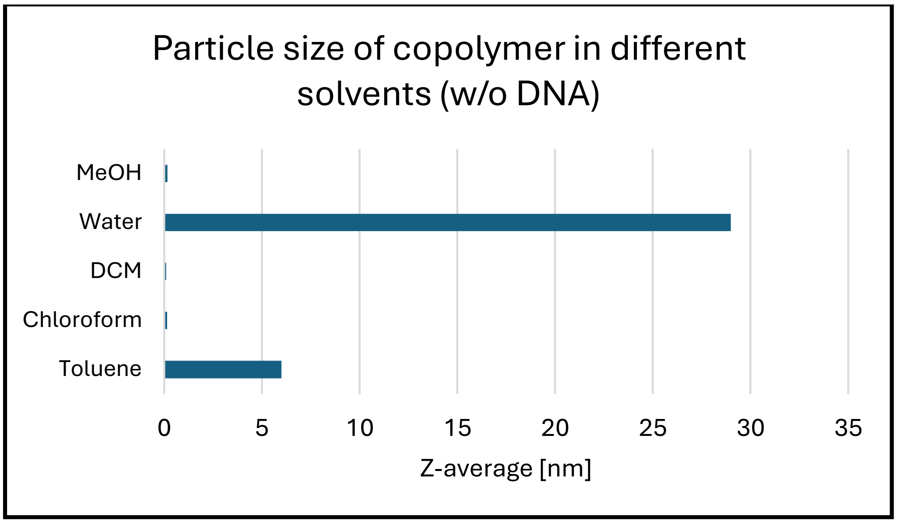

Figure S16. DLS measurement of copolymer particle sizes in different solvents.

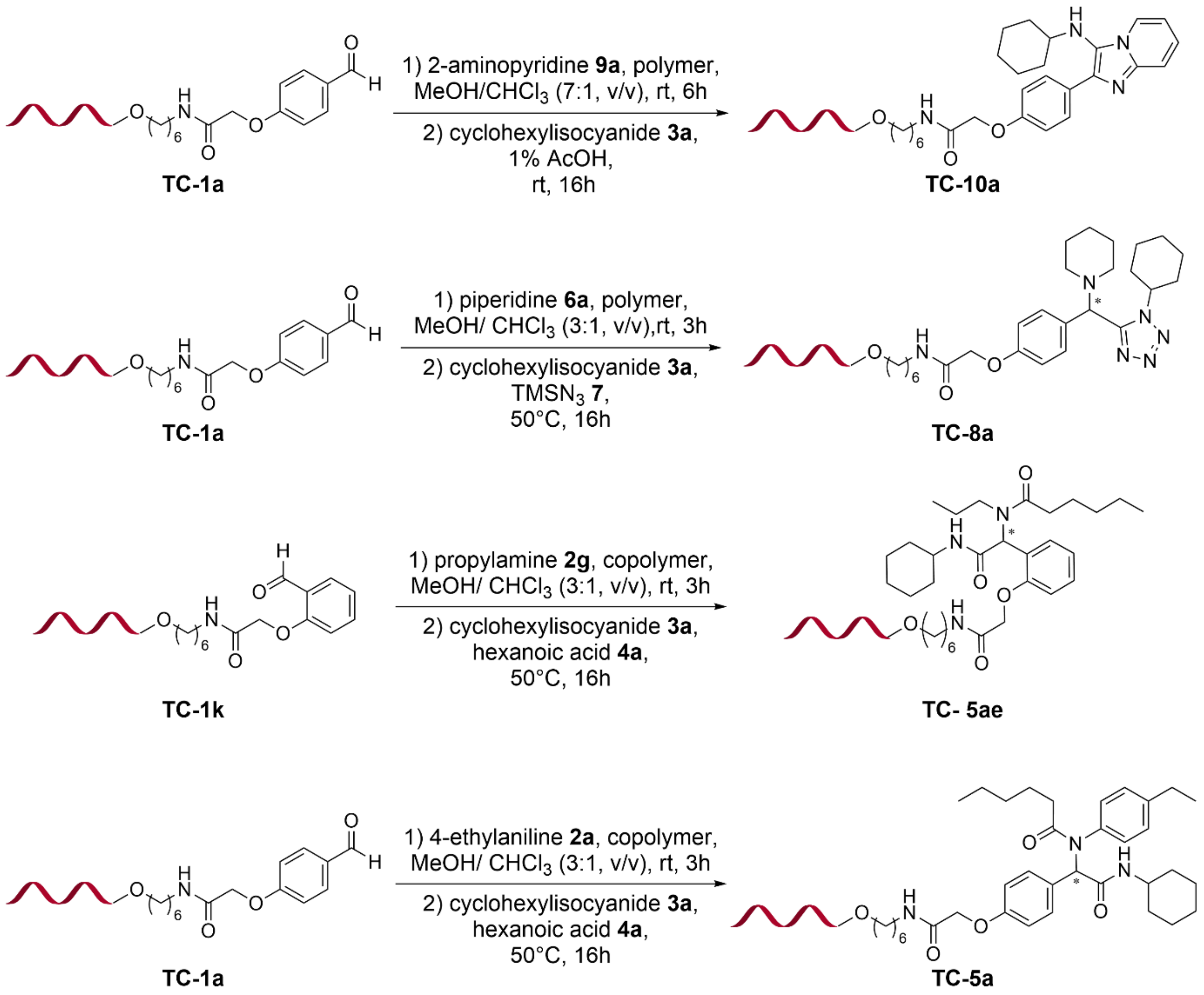

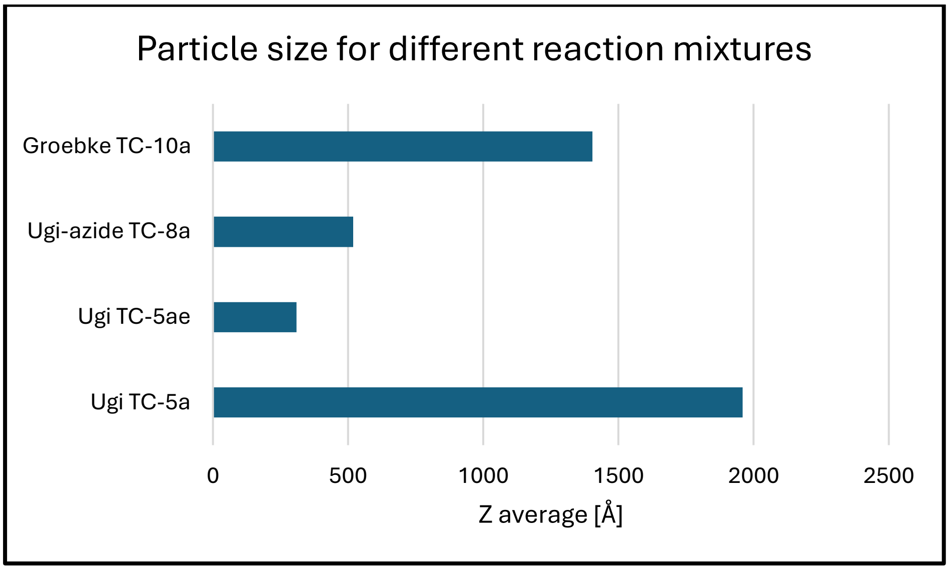

Figure S17. DLS measurement of particles formed by DNA-copolymer interaction in toluene. Effect of different reaction mixtures on particle size. Reaction conditions were the same as in the standard procedure.

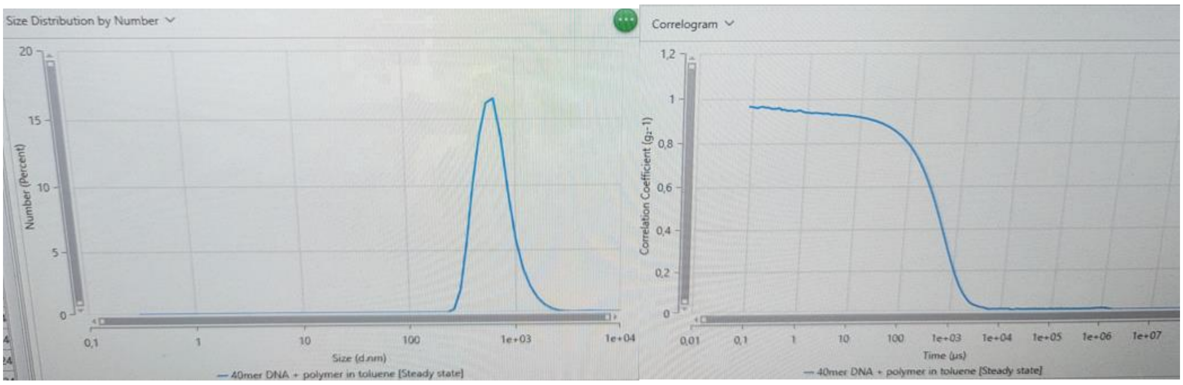

Figure S18. Size distribution by number (left-hand screenshot) and correlogram (right-hand screenshot) for DLS measurement of particles formed by 40mer DNA & copolymer complex in toluene.

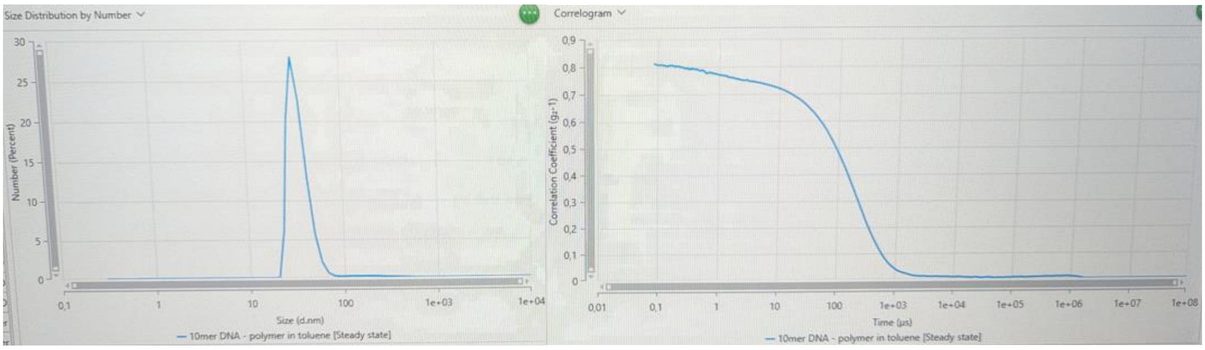

Figure S19. Size distribution by number (left-hand screenshot) and correlogram (right-hand screenshot) for DLS measurement of particles formed by 10mer DNA & copolymer complex in toluene.

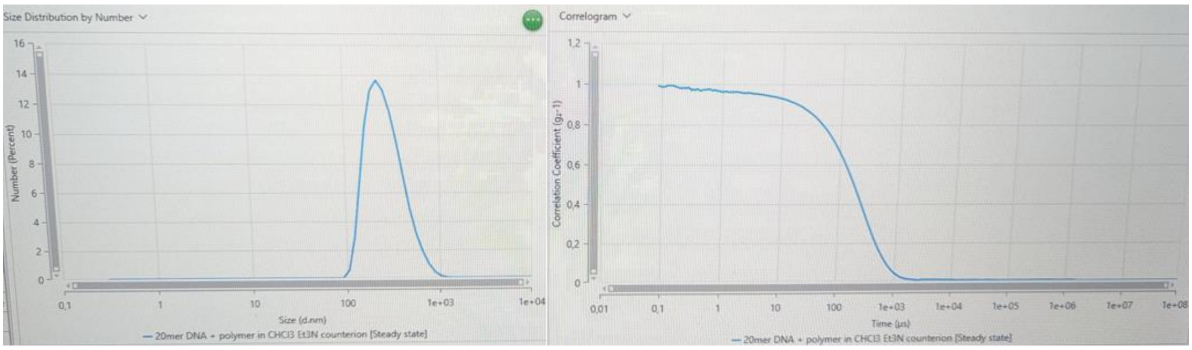

Figure S20. Size distribution by number (left-hand screenshot) and correlogram (right-hand screenshot) for DLS measurement of particles formed by 20mer DNA & copolymer in $CHCl_3$.

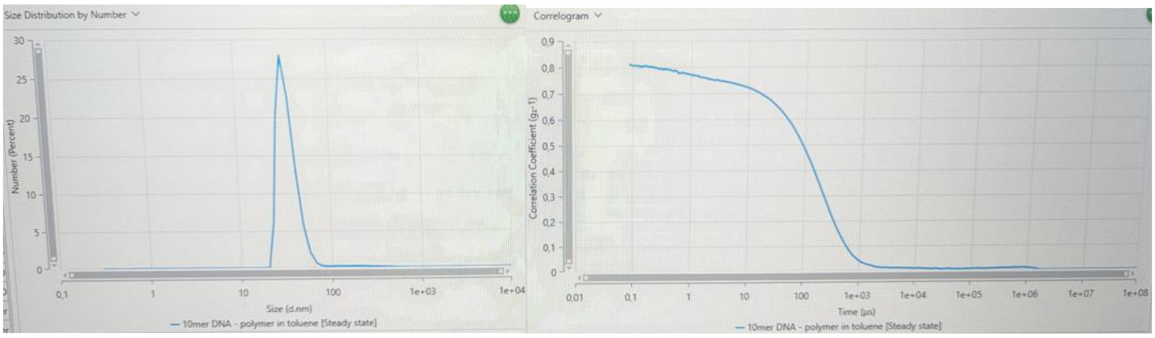

Figure S21. Size distribution by number (left-hand screenshot) and correlogram (right-hand screenshot) for DLS measurement of particles formed by the copolymer in water.

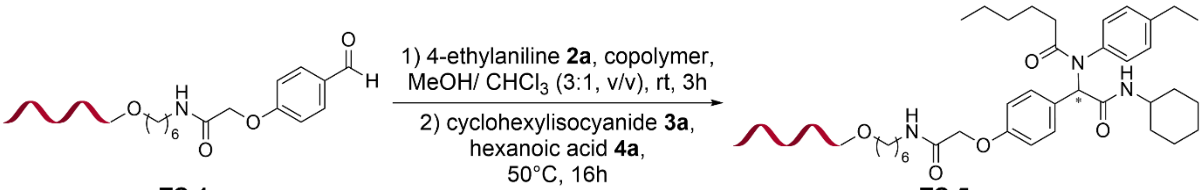

TC-1a **TC-5a**

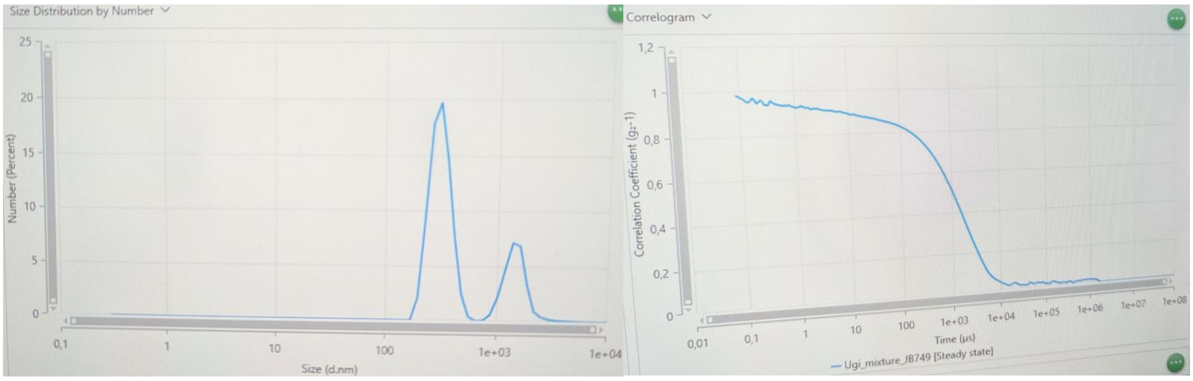

Figure S22. Size distribution by number (left-hand screenshot) and correlogram (right-hand screenshot) for DLS measurement of DNA & copolymer in the Ugi reaction mixture **TC-5a**.

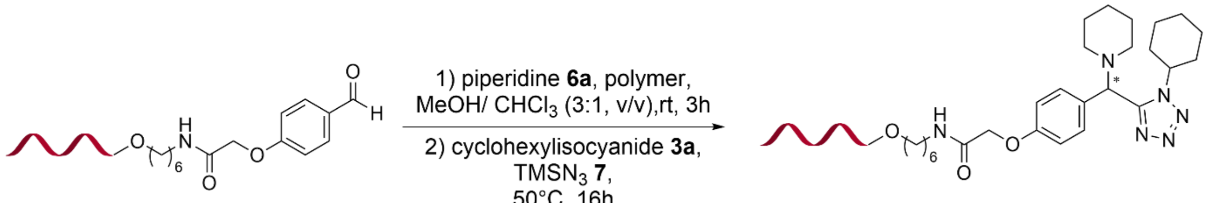

TC-1a **TC-8a**

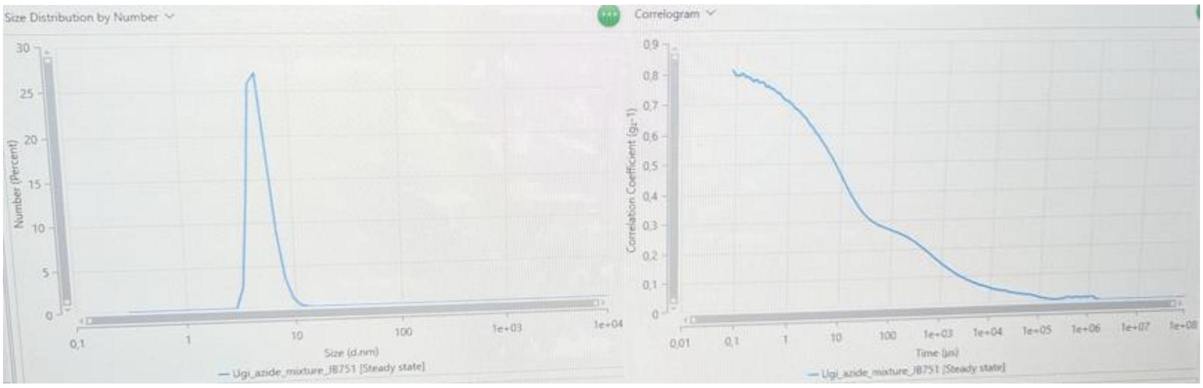

Figure S23. Size distribution by number (left-hand screenshot) and correlogram (right-hand screenshot) for DLS measurement of DNA & copolymer in the Ugi-azide reaction mixture **TC-8a**.

DOSY-NMR

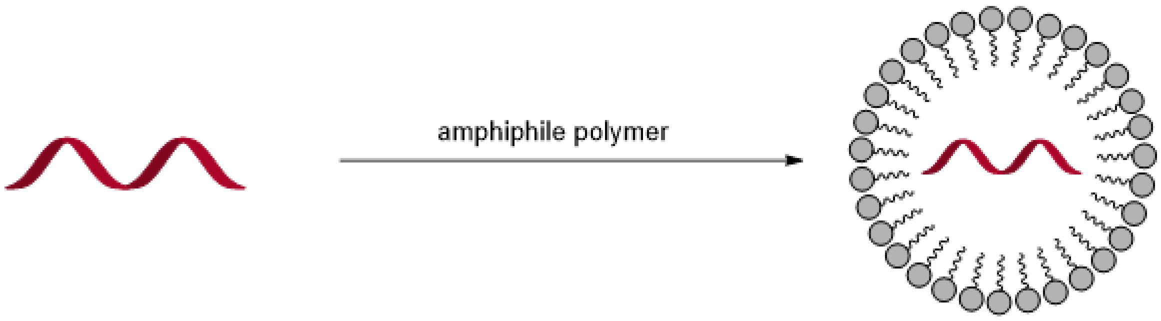

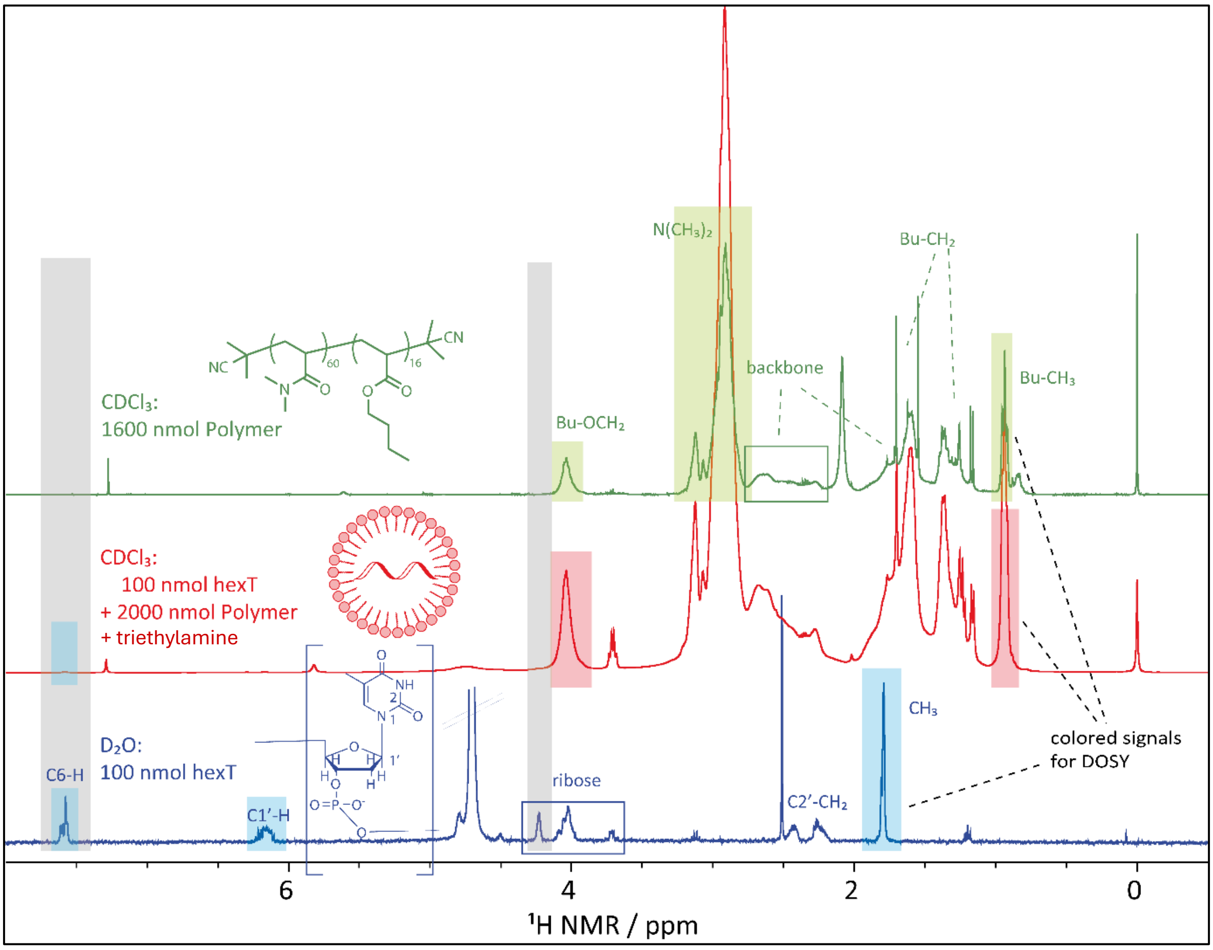

Figure S24. ^{1}H NMR spectra of a hexathymidine (hexa-T) DNA oligomer in D_2O (lower panel), a mixture of hexa-T and the copolymer (middle panel), and the copolymer alone (upper panel) in $CDCl_3$. The spectra were scaled for better comparability. Signal assignment is indicated and well separated signals for the DOSY analysis, are highlighted by colored boxes.

Table S2. Diffusion coefficients extracted from fitting of different signal decay curves in DOSY experiments of hexa-T in D_2O as well as the copolymer and a mixture of hexa-T and the copolymer in $CDCl_3$. D results from a monoexponential fit, D1 and D2 are the two components from a biexponential fit. As the values for the mixture with 50 nmol do not differ roughly one order of magnitude, the monoexponential fit was used in the discussion even though the biexponential fit describes the data better.

Sample	signal (ppm)	D [m^2/s]	D1	D2
hexa-T	1.90 - 1.70	2.07E-10		
	6.29 - 6.02	2.28E-10		
50 nmol	7.67 - 7.50	2.08E-10		
hexa-T				
100 nmol	7.67 - 7.50	1.98E-10		
polymer	1.00 - 0.91	1.76E-10		
	3.28 - 2.77	1.81E-10		
	4.21 - 3.84	1.75E-10		
mixture	1.07 - 0.75	1.49E-10	1.32E-10	6.40E-10
50 nmol	4.24 - 3.87	1.39E-10	1.39E-10	1.39E-10
mixture	1.07 – 0.75	1.25E-10		
100 nmol	7.67 - 7.50	2.85E-11	Not fully decayed	

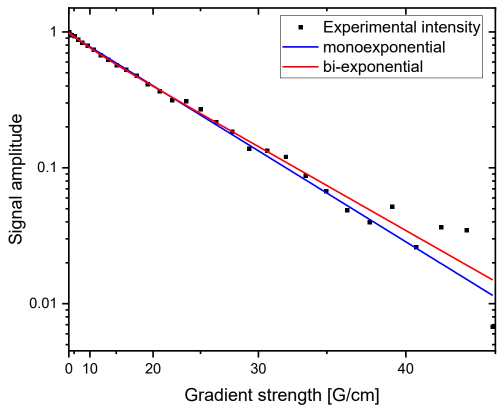

hexa-T – signal at 1.90 ppm

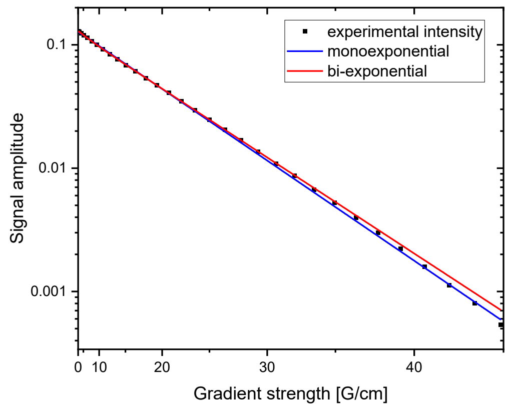

copolymer – signal at 1.00 ppm

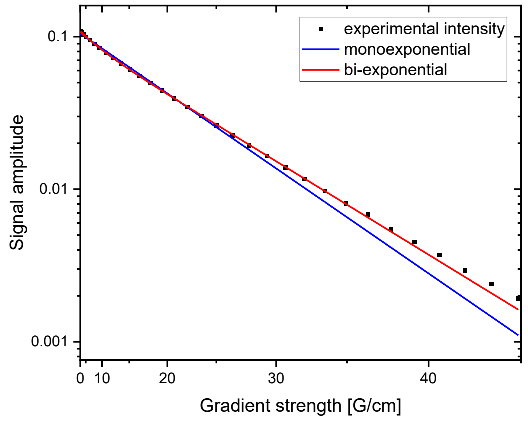

mixture – signal at 1.07 ppm

Figure S25. Exemplary DOSY signal decay curves for selected signals. Diffusion coefficients were obtained by either mono- (blue) or biexponential fitting (red) of the respective signals.

Negatively stained Transmission Electron Microscopy analysis of particles formed by DNA oligomers and the copolymer

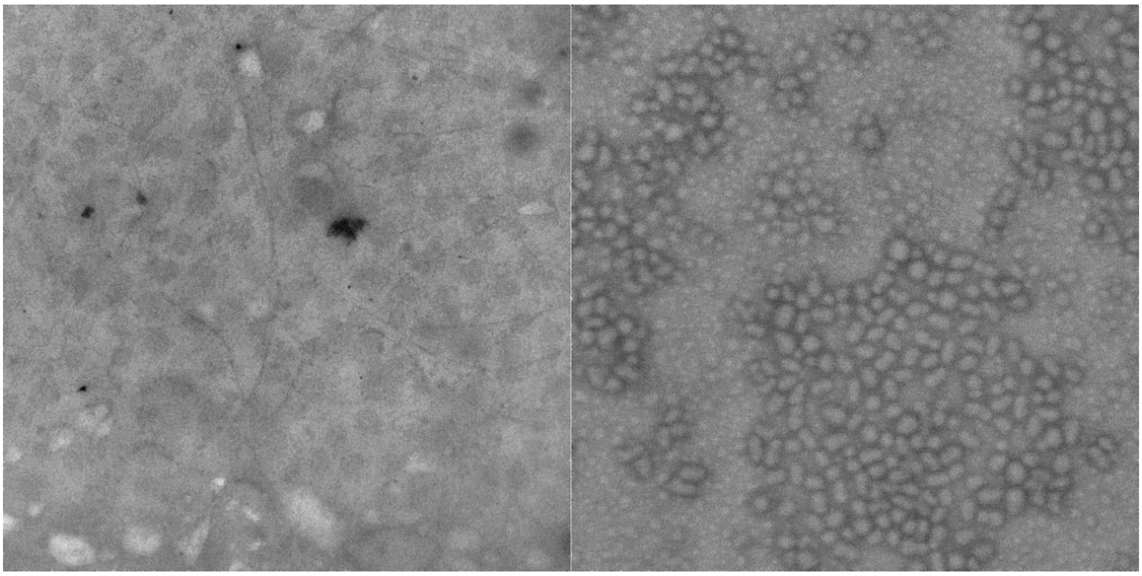

Figure S26. TEM image of 20mer DNA (Et_3NH^+ form) (c = 10 nmol per 300µL) and 30 equiv. polymer in chloroform (left-hand picture: not enlarged; right-hand picture: enlarged).

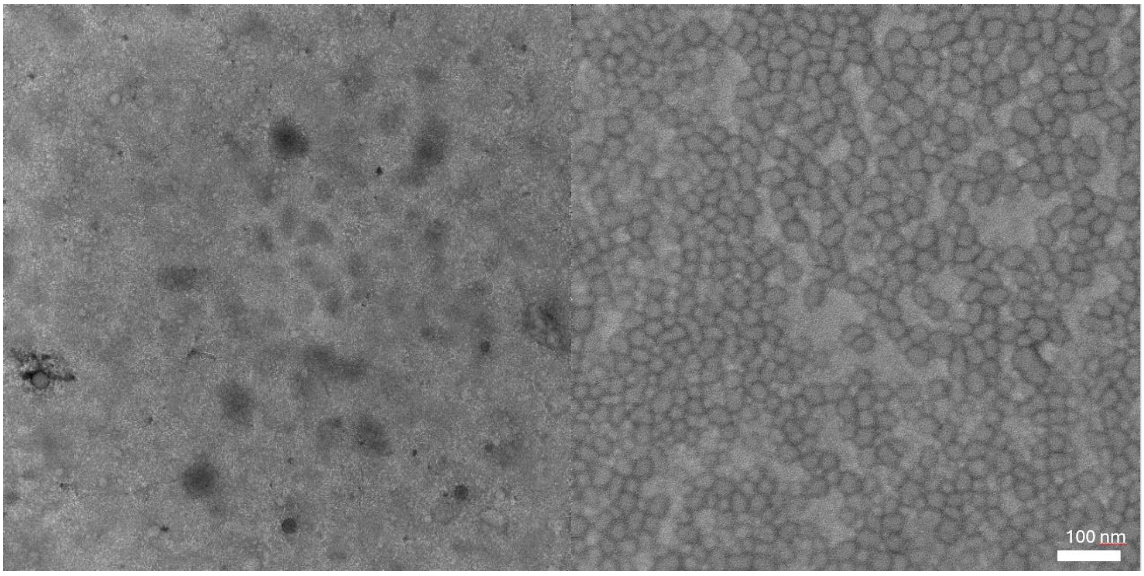

Figure S27. TEM image of 20mer DNA (Et_3NH^+ form) (c = 5 nmol per 300µL) and 30 equiv. polymer in chloroform (left-hand picture: not enlarged; right-hand picture: enlarged).

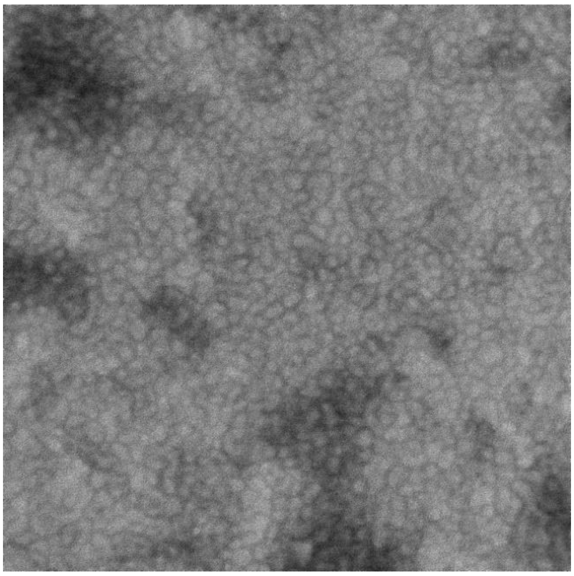

Figure S28. TEM image of 80mer DNA (Et_3NH^+ form) (c = 10 nmol per 300µL) and 30 equiv. polymer in chloroform (enlarged).

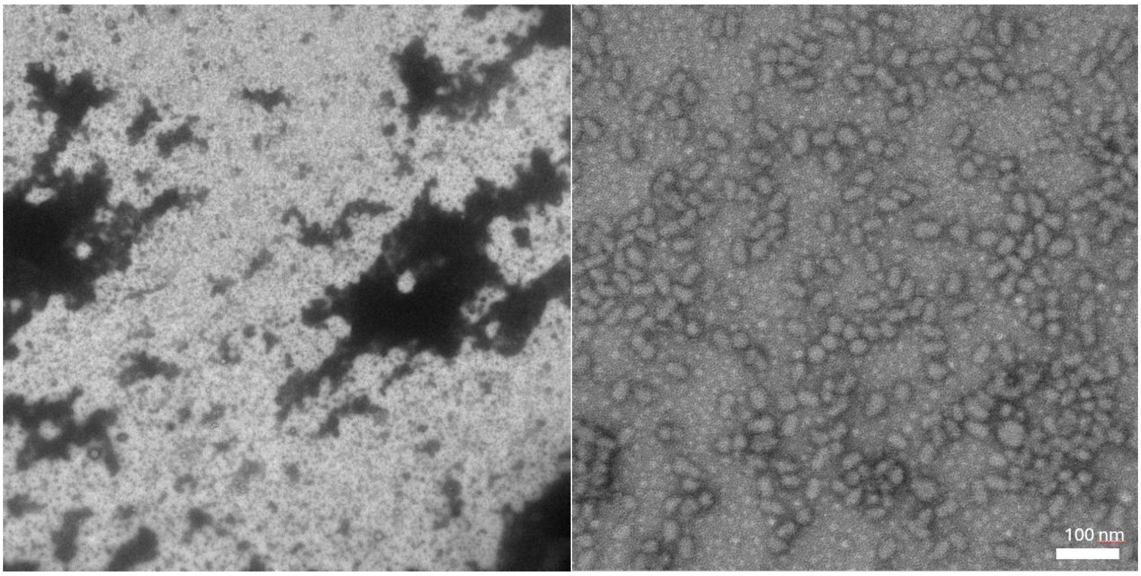

Figure S29. TEM image of 80mer DNA (Et_3NH^+ form) (c = 5 nmol per 300µL) and 30 equiv. polymer in chloroform (left-hand picture: not enlarged; right-hand picture: enlarged).

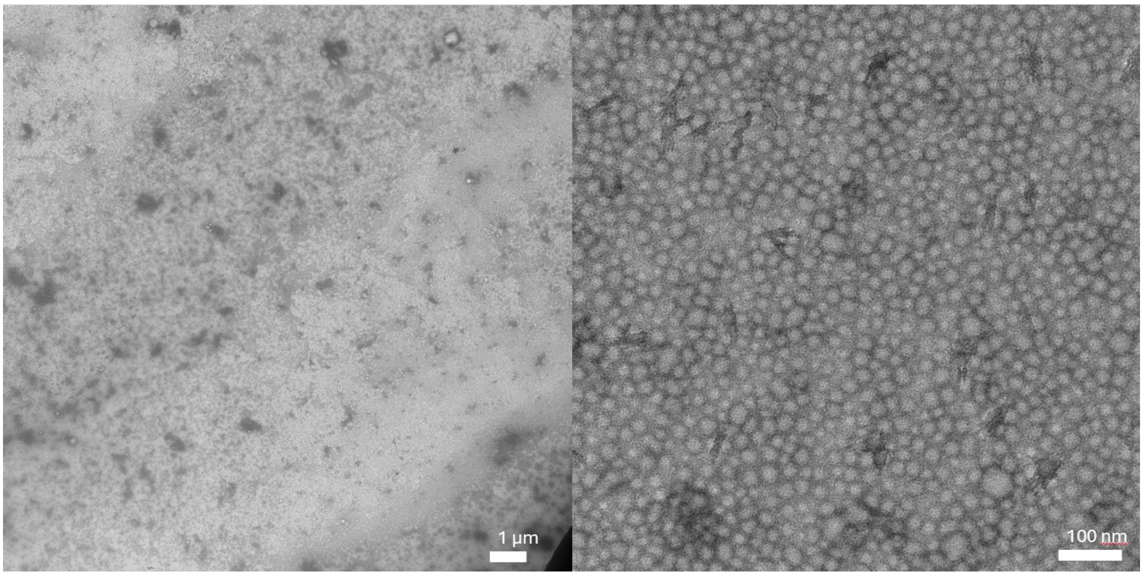

Figure S30. TEM image of 20mer DNA (Et_3NH^+ form) (c = 5 nmol per 300µL) and 30 equiv. polymer in water (left-hand picture: not enlarged; right-hand picture: enlarged).

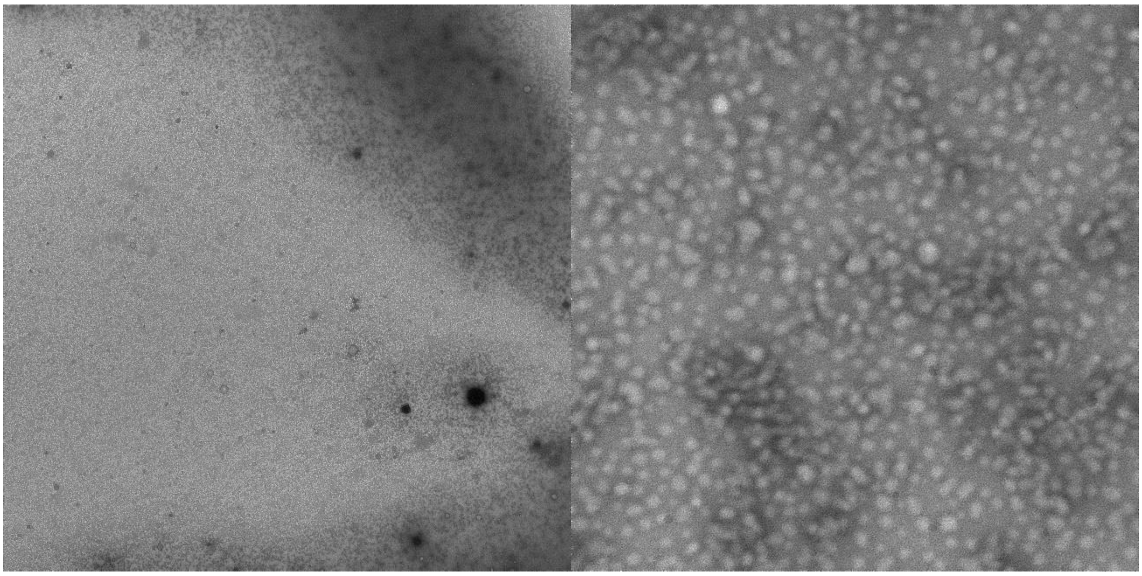

Figure S31. TEM image of 20mer DNA (Et_3NH^+ form) (c = 1 nmol per 300µL) and 30 equiv. polymer in water (left-hand picture: not enlarged; right-hand picture: enlarged).

Amide Coupling of Carboxylic Acid to CPG-Bound Oligonucleotide (10mer TC)

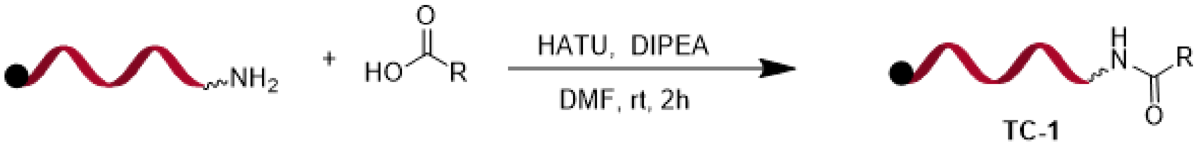

	Aldehyde
**TC-1a**	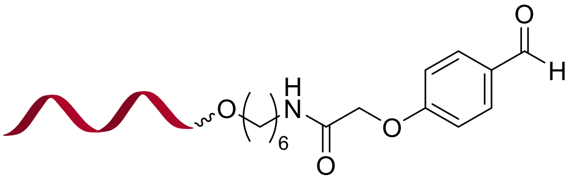
**TC-1b**	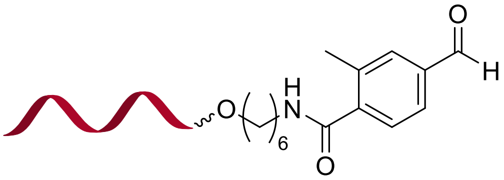
**TC-1c**	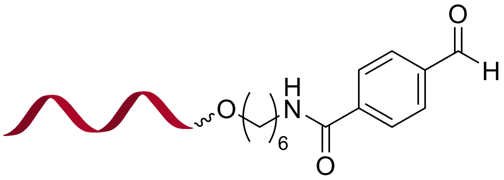
**TC-1d**	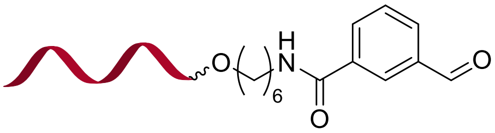
**TC-1e**	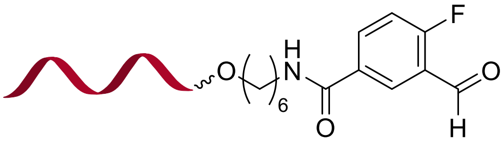
**TC-1f**	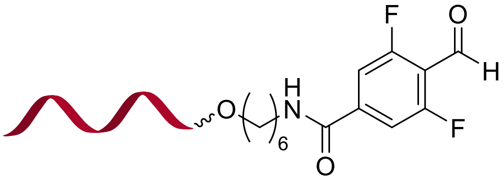
**TC-1g**	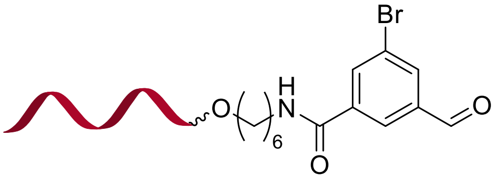
**TC-1h**	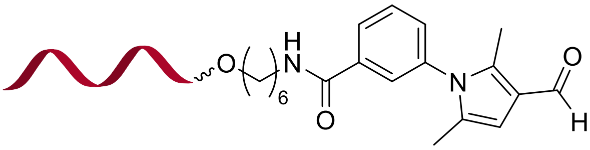

TC-1i

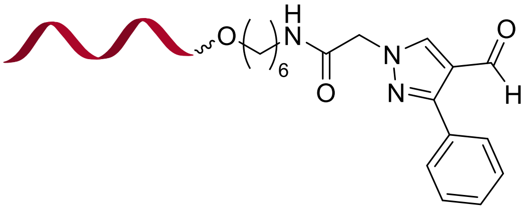

TC-1j

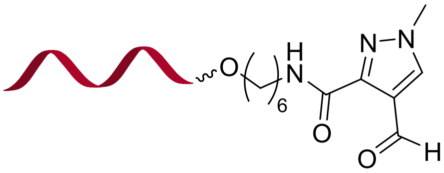

TC-1k

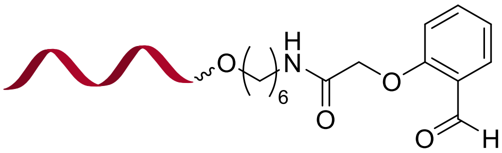

TC-1l

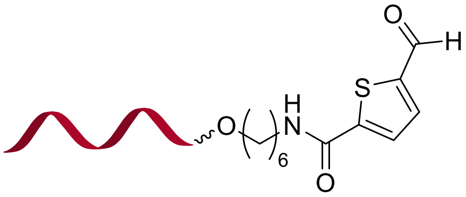

TC-1m

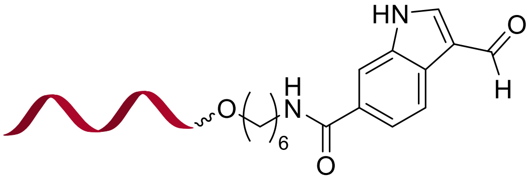

TC-1n

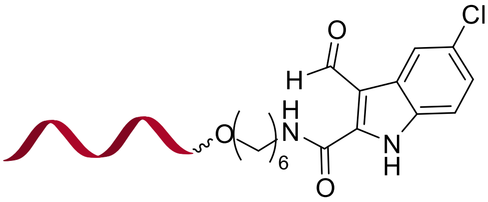

Representative examples of CPG- bound aldehyde connjugates (10mer TC)

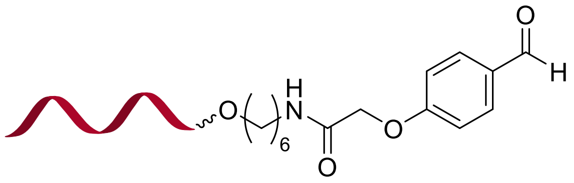

TC-1a

MS calc. 3246.0; found 3246.3

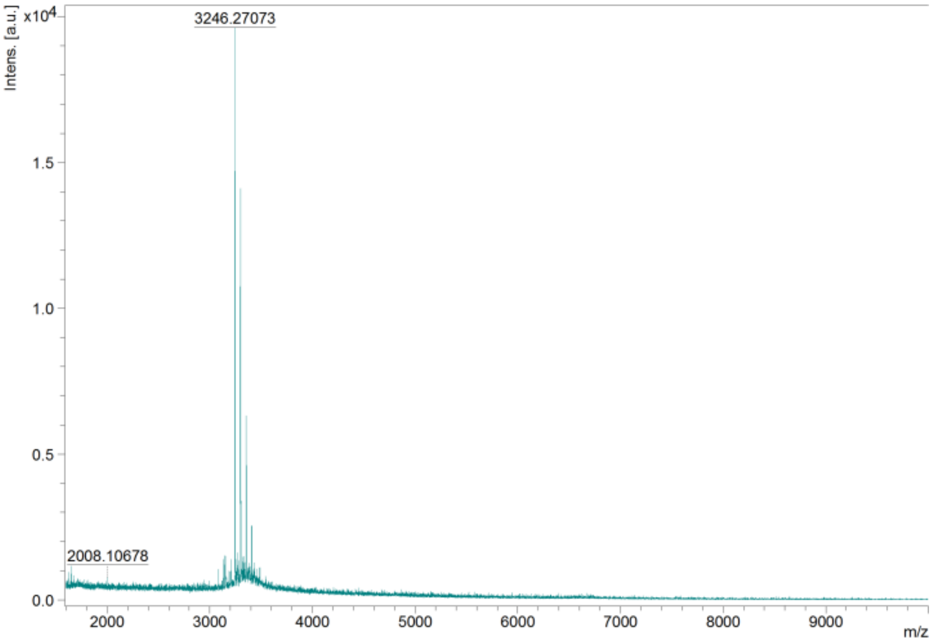

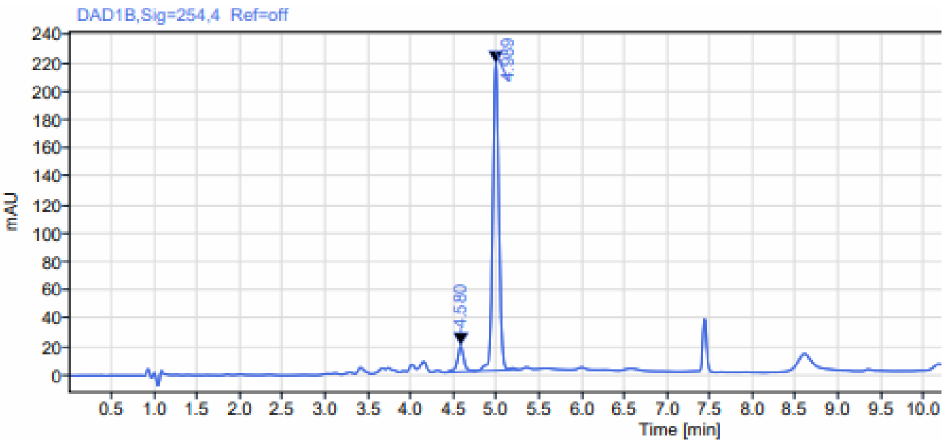

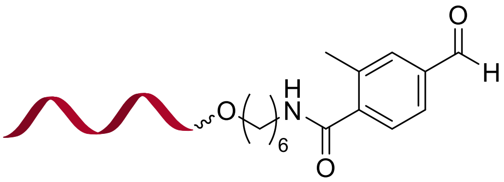

TC-1b

MS calc. 3230.0; found 3230.9

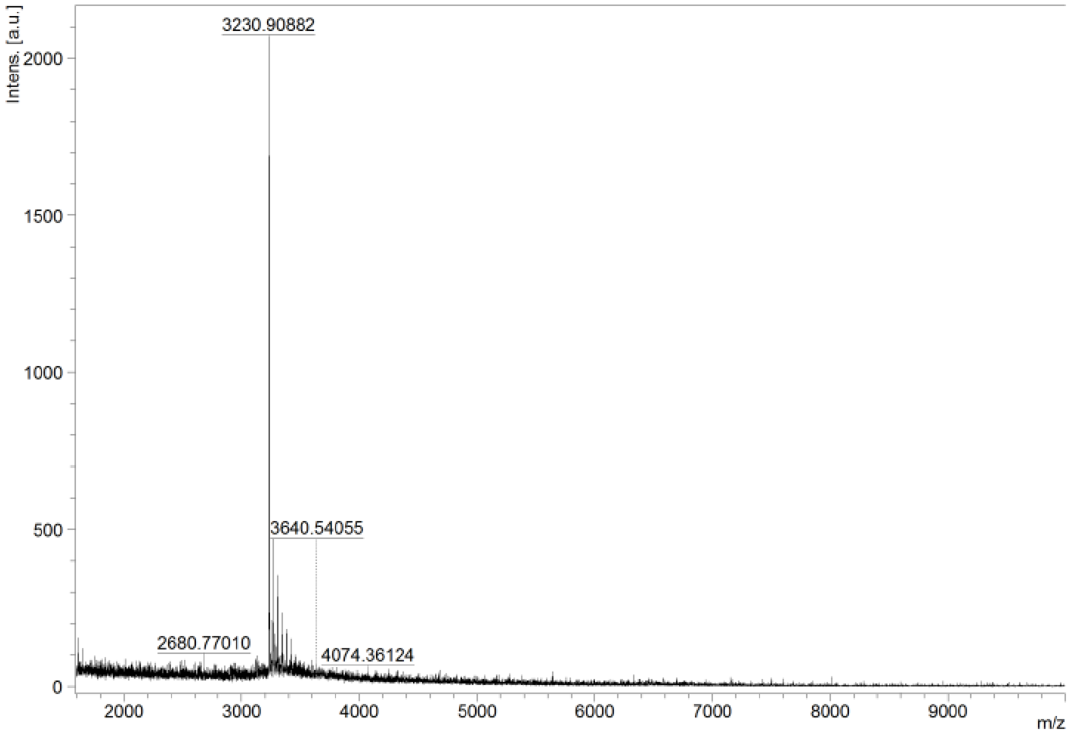

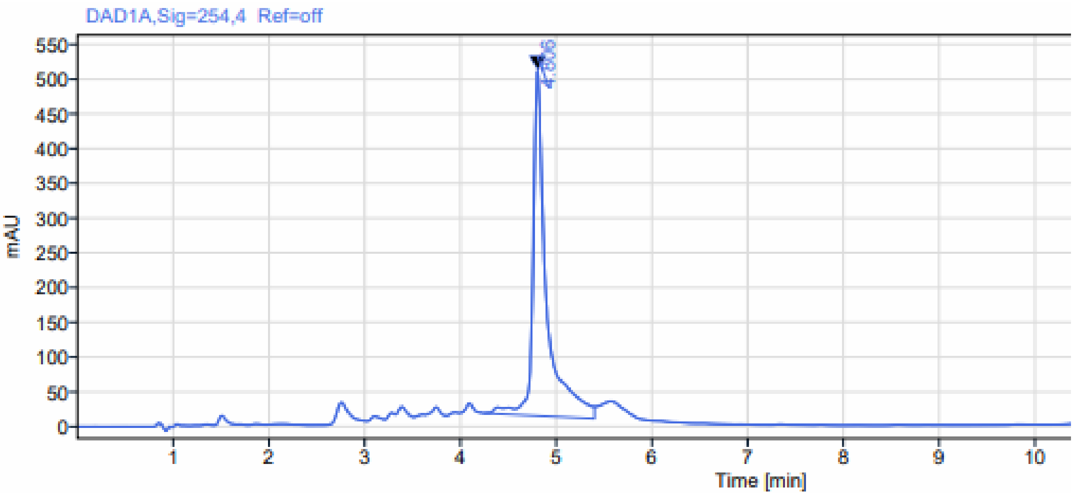

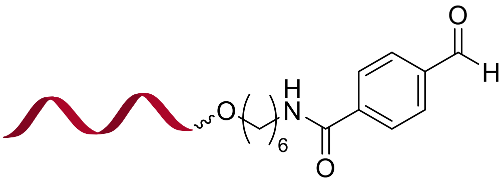

TC-1c

MS calc. 3216.0; found 3216.1

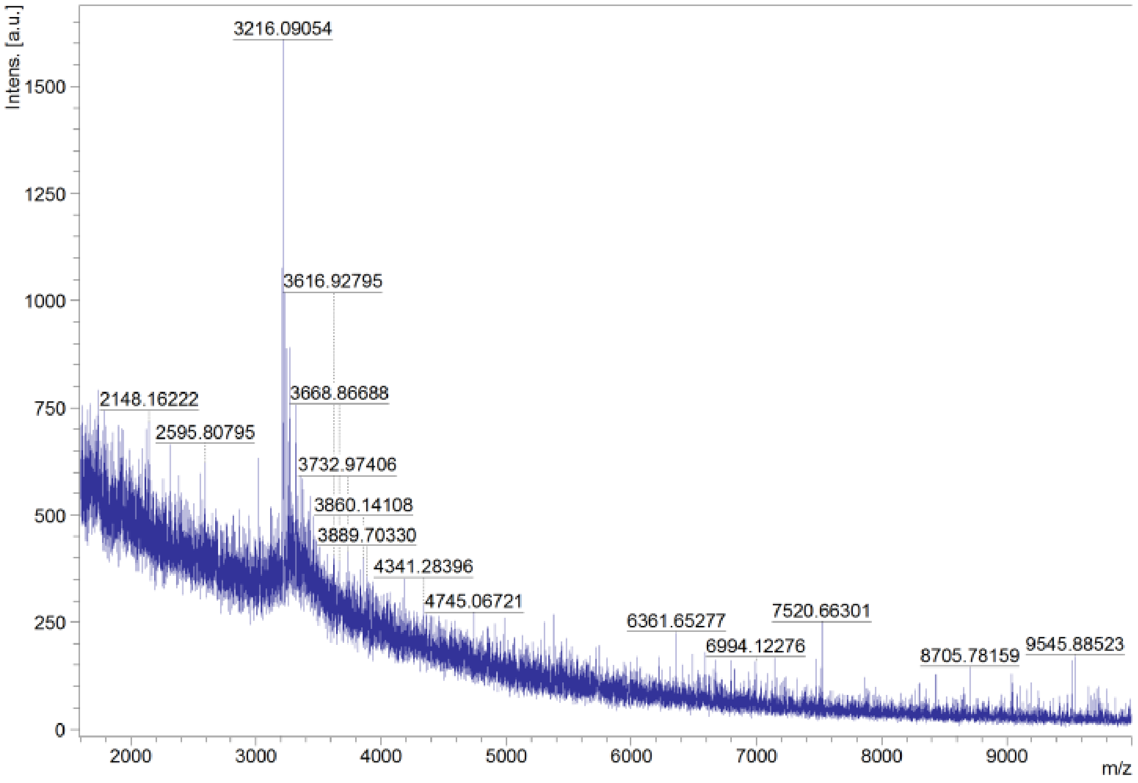

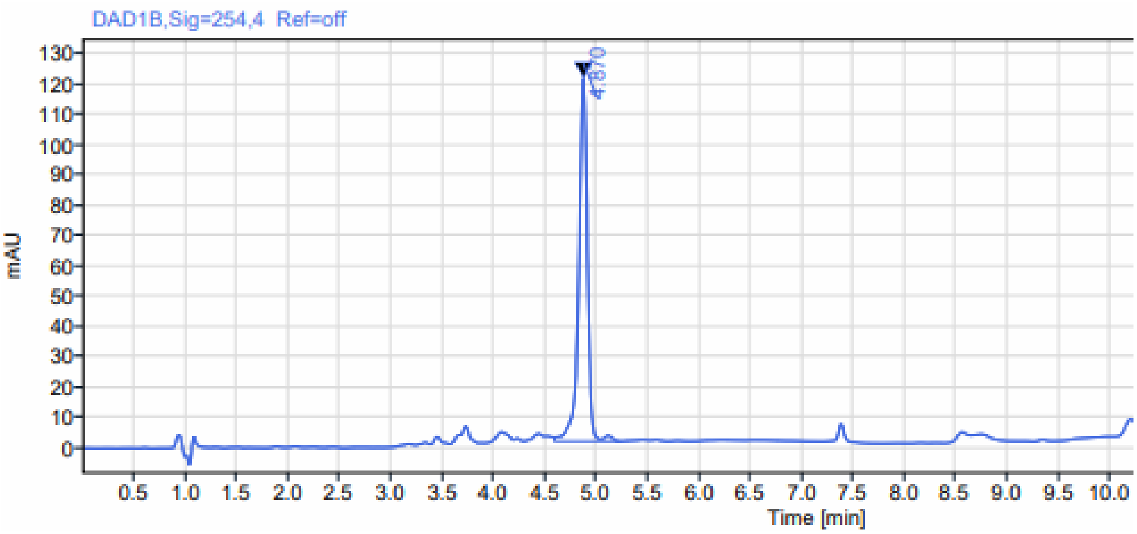

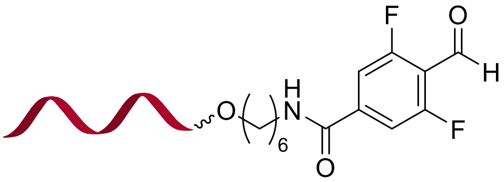

TC-1f

MS calc. 3252.0; found 3252.8

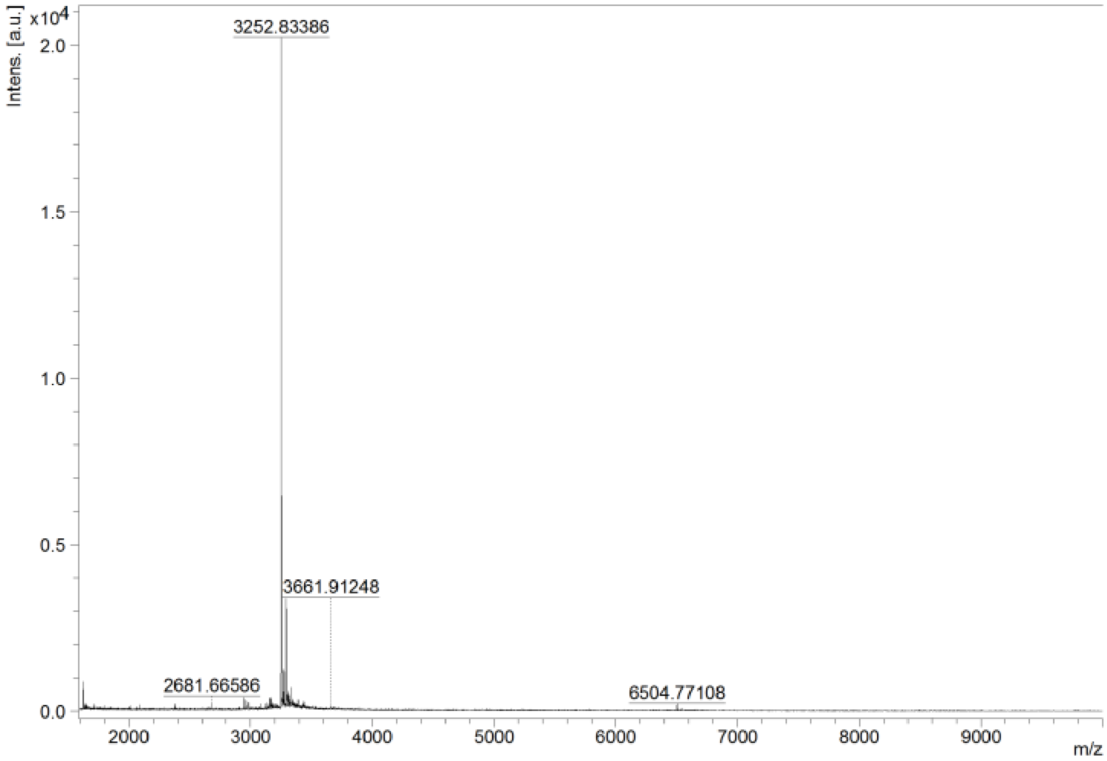

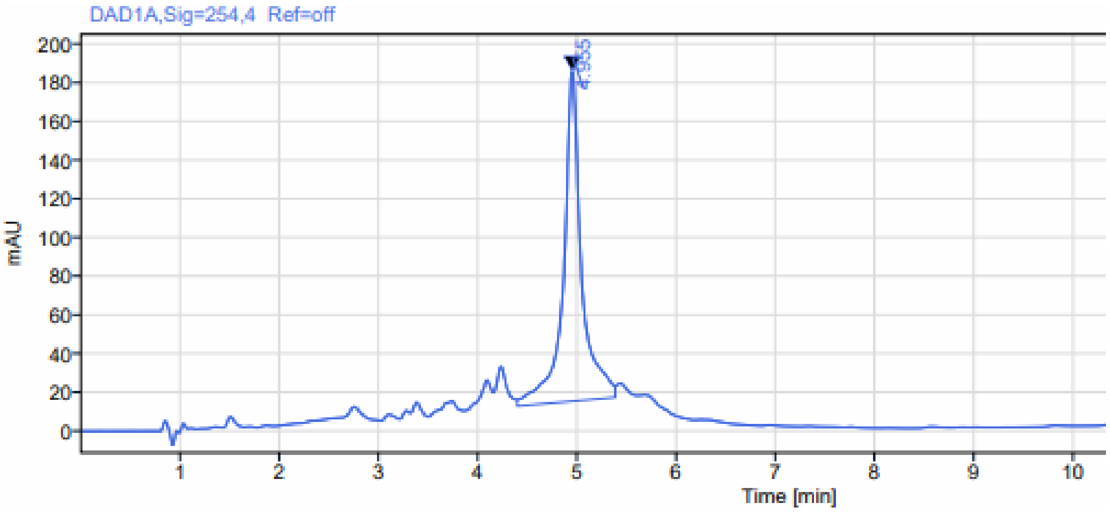

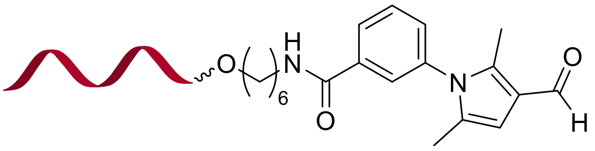

TC-1h

MS calc. 3306.0; found 3223.0 [M+NH_3]

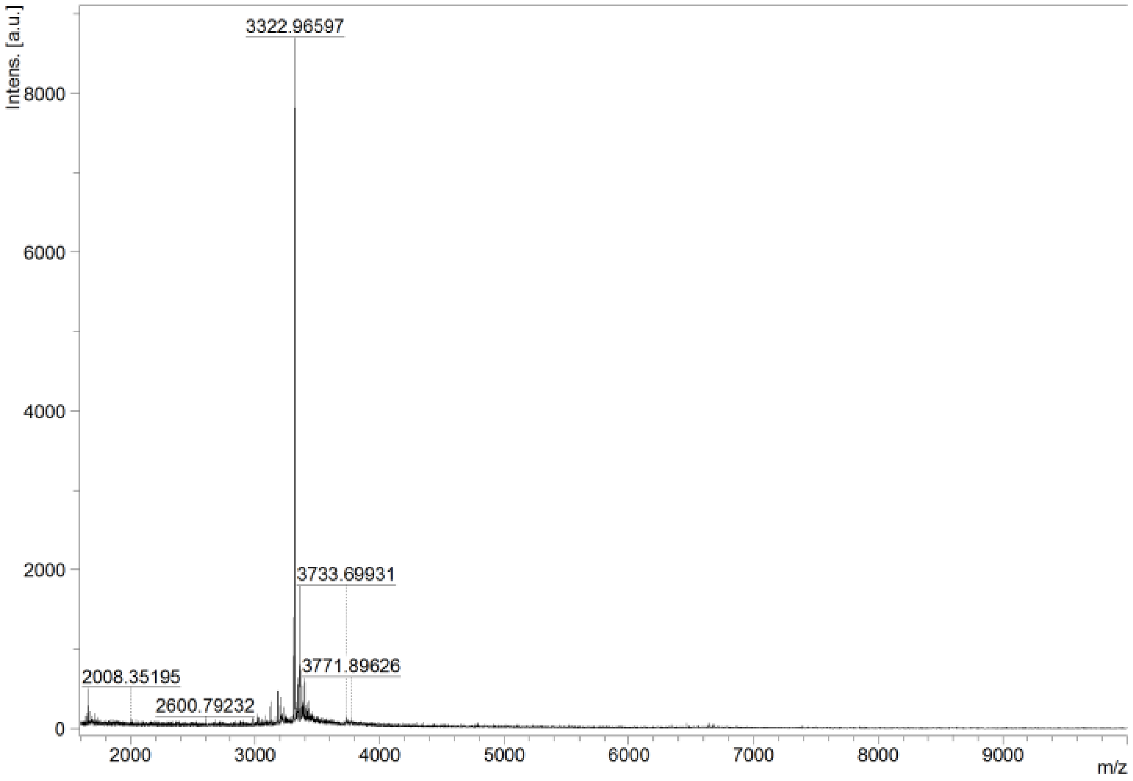

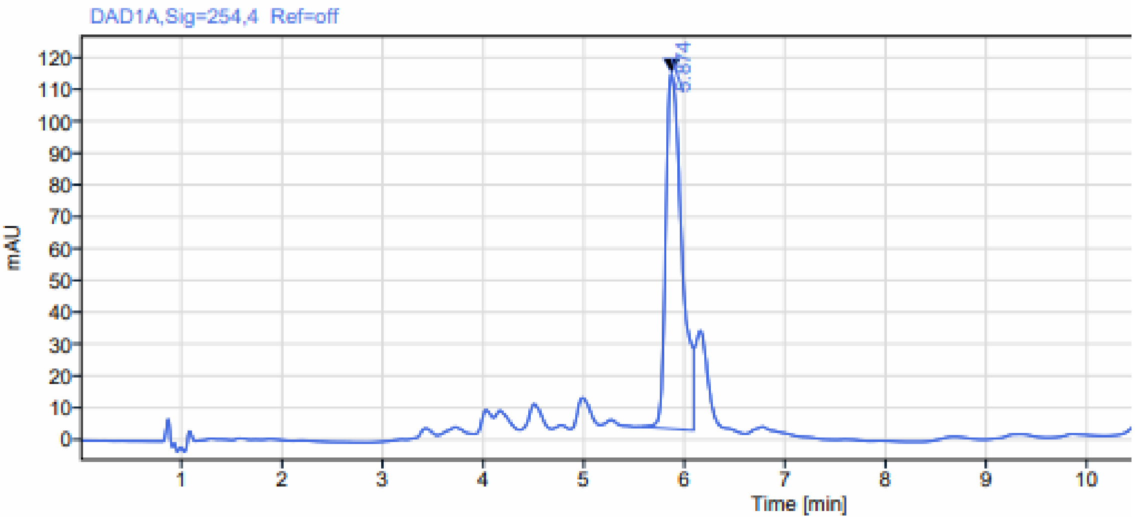

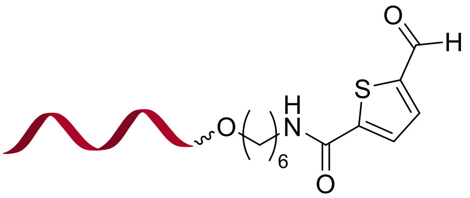

TC-1j

MS calc. 3222.0; found 3222.9

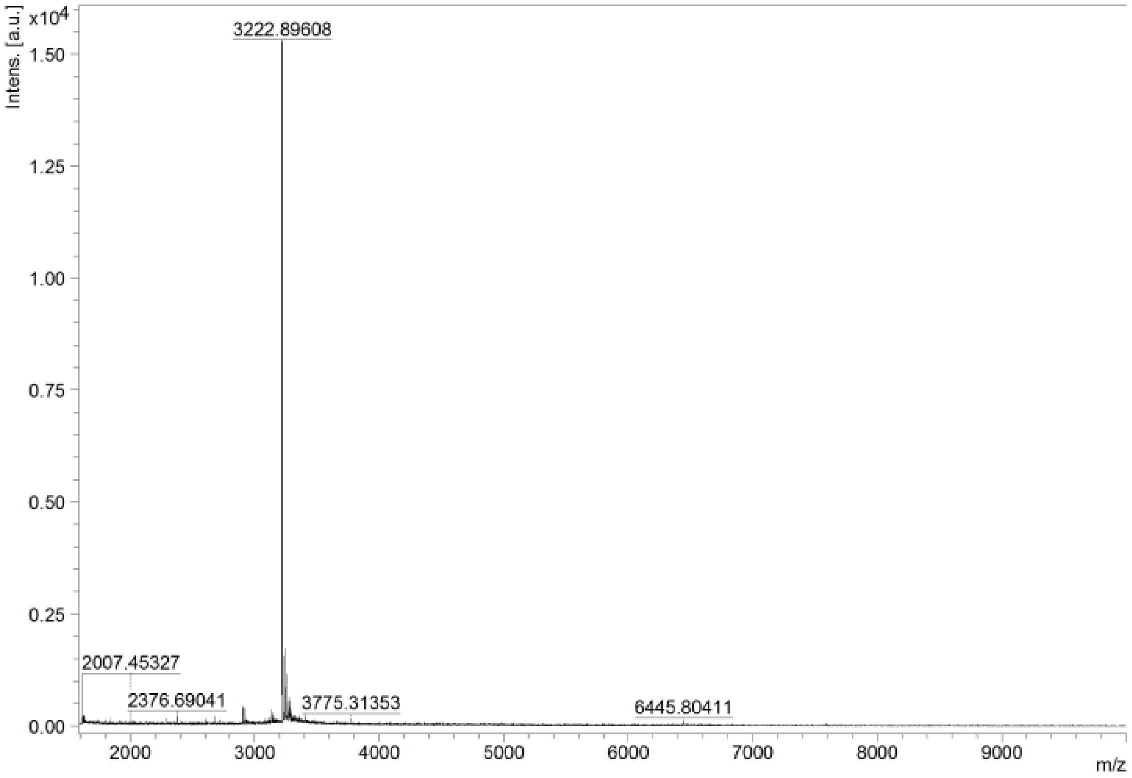

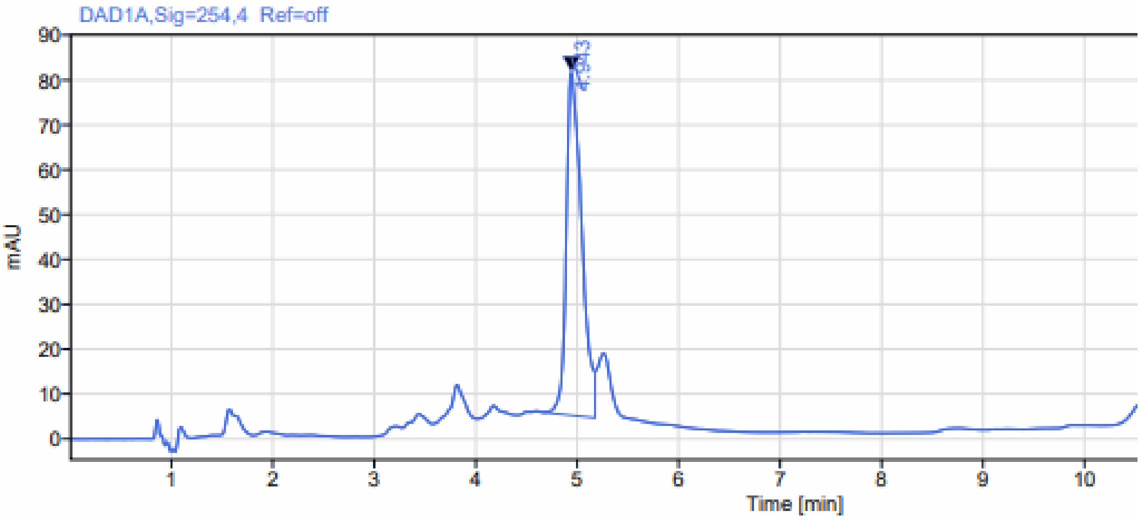

Ugi four-component reaction

Graphic presentation of the procedure

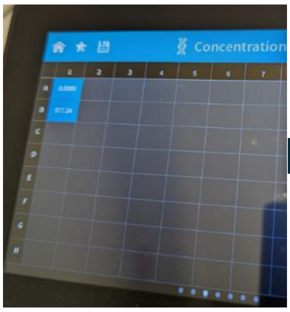

Nanodrop → measuring DNA concentration

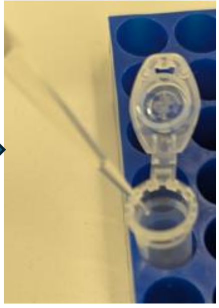

Pipetting 3 nmol DNA

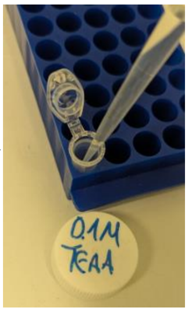

Addition of TEA as counterion

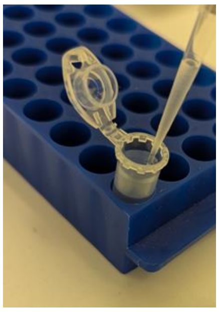

Addition of copolymer

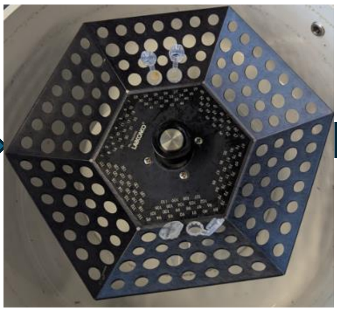

Solvent evaporation

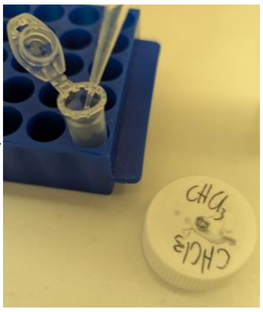

Addition of $CHCl_3$

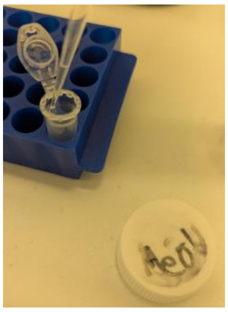

Addition of MeOH

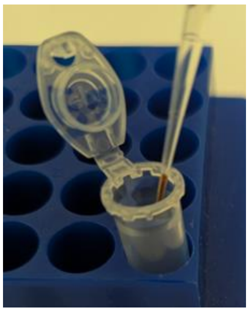

Addition of primary amine

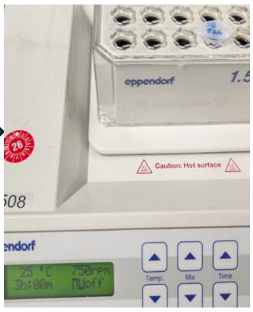

Imine formation

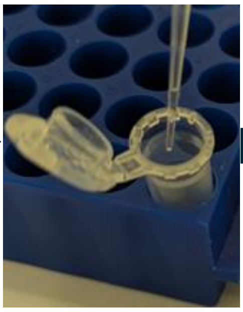

Addition of carboxylic acid

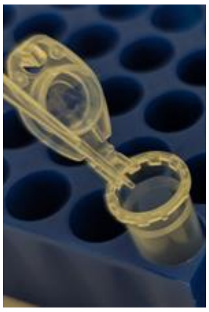

Addition of isocyanide

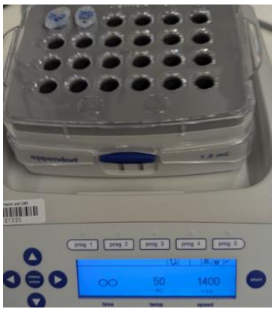

Shaking at 50°C for 16h (Table 1, entry 4)

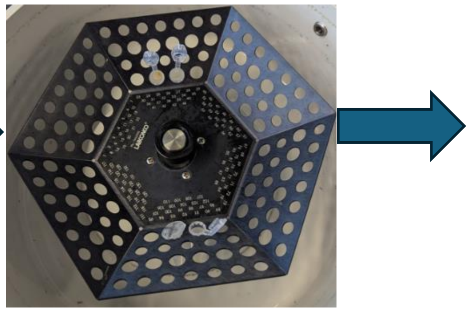

Solvent evaporation

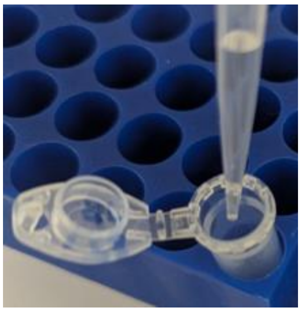

Addition of water for analysis & purification

Optimization of Ugi four-component reaction

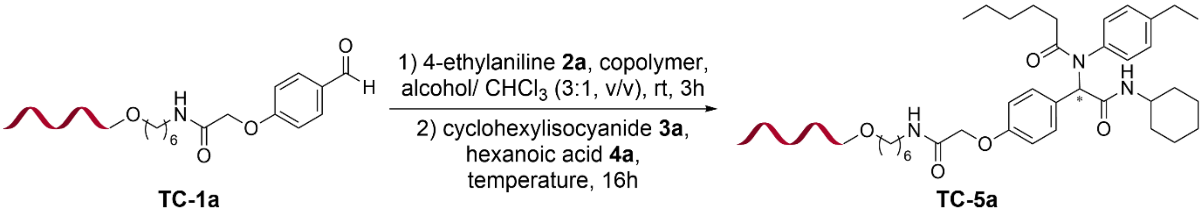

Table S3. Conversion rates for different reaction conditions for the The Ugi-four-component reaction.

No.	Solvent[a]	equivalents	Concentration	temperature	catalyst	Product conversion
**1**	MeOH/$CHCl_3$	250	15 mM	50°C	/	>95%
**2**	MeOH/$CHCl_3$	500	30 mM	50°C	/	>95%
**3**	MeOH/$CHCl_3$	1000	60 mM	50°C	/	>95%
**4**	MeOH/$CHCl_3$	2000	120 mM	50°C	/	>95%
**5**	MeOH/$CHCl_3$	2000	120 mM	37°C	/	>95%
**6**	MeOH/$CHCl_3$	2000	120 mM	rt[c]	/	>95%
**7**	MeOH/$CHCl_3$	2000	120 mM	rt	$ZnCl_2$	>95%
**8**	MeOH/$CHCl_3$	2000	120 mM	rt	$MgCl_2$	>95%
**9**	MeOH/$CHCl_3$	2000	120 mM	rt	$FeCl_2$	>95%
**10**	EtOH/$CHCl_3$	2000	120 mM	50°C	/	>95%
**11**	iPrOH/$CHCl_3$	2000	120 mM	50°C	/	>95%
**12[b]**	CF_3CH_2OH/$CHCl_3$	3000	180mM	60°C	/	>95%

[a] alcohol/$CHCl_3$ (3:1, vol/vol); [b]all reactants were added simultaneously, and the reaction was run for 48h; [c]rt means 25°C

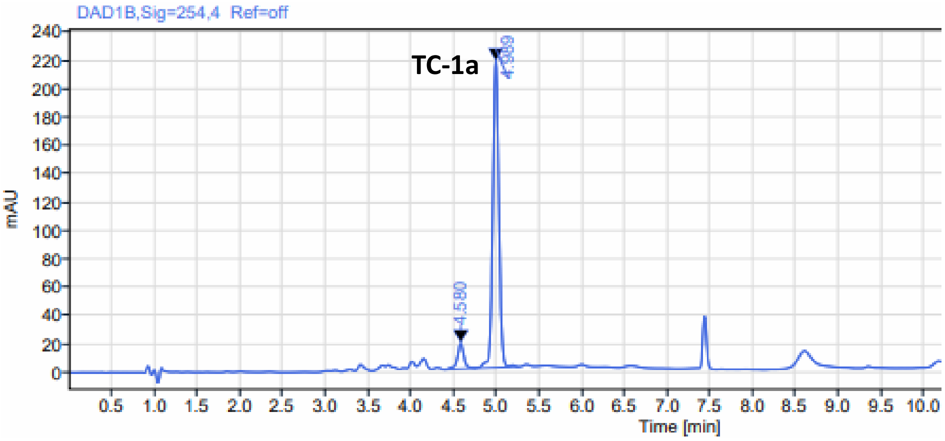

Figure S32. Analytical HPLC trace of starting material **TC-1a**.

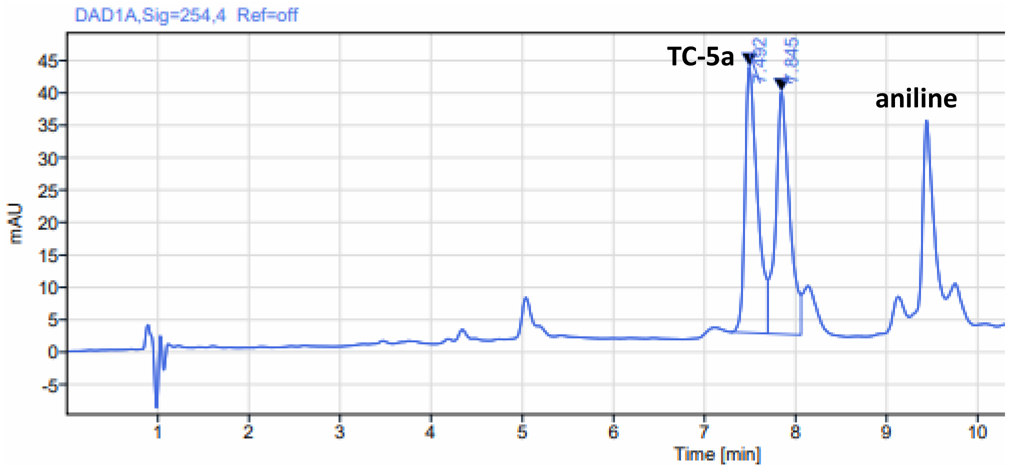

Figure S33. Crude analytical HPLC trace for product **TC-5a** with 250 equiv amine, 250 equiv isocyanide, 250 equiv acid in MeOH/$CHCl_3$ (3:1,v/v) at 50°C (entry 1).

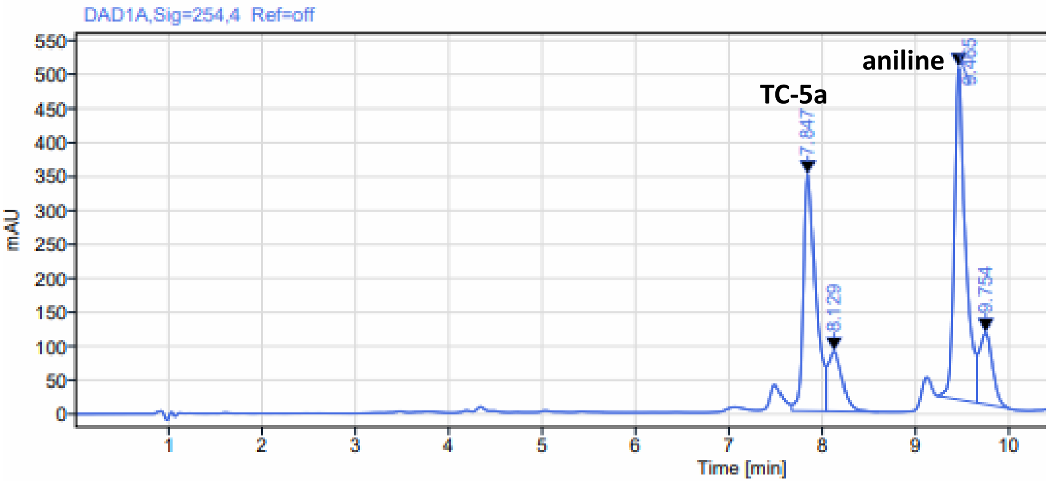

Figure S34. Crude analytical HPLC trace for product **TC-5a** with 500 equiv amine, 500 equiv isocyanide, 500 equiv acid in MeOH/$CHCl_3$ (3:1,v/v) at 50°C (entry 2).

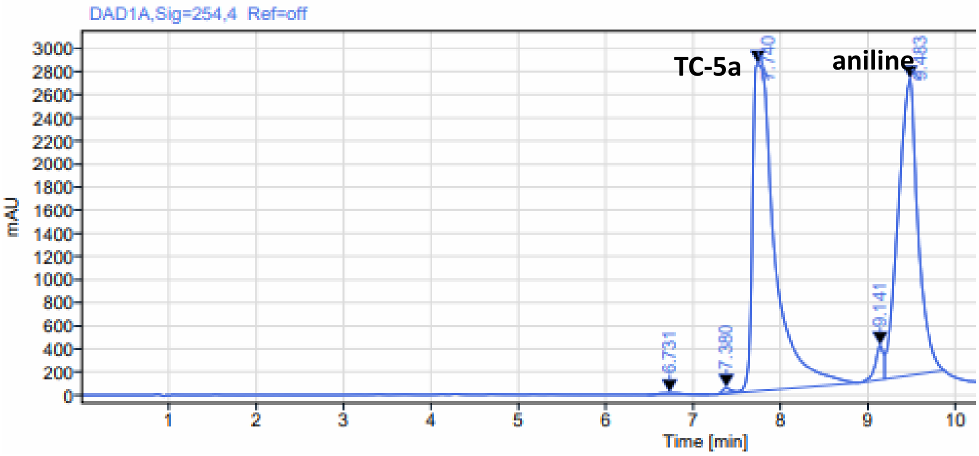

Figure S35. Crude analytical HPLC trace for product **TC-5a** with 1000 equiv amine, 1000 equiv isocyanide, 1000 equiv acid in MeOH/$CHCl_3$ (3:1,v/v) at 50°C (entry 3).

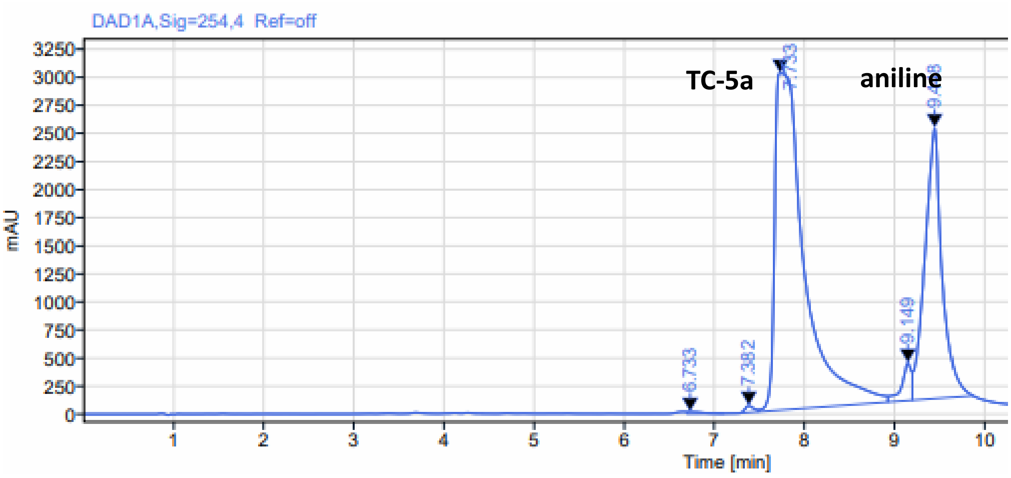

Figure S36. Crude analytical HPLC trace for product **TC-5a** with 2000 equiv amine, 2000 equiv isocyanide, 2000 equiv acid in MeOH/$CHCl_3$ (3:1,v/v) at 50°C (entry 4).

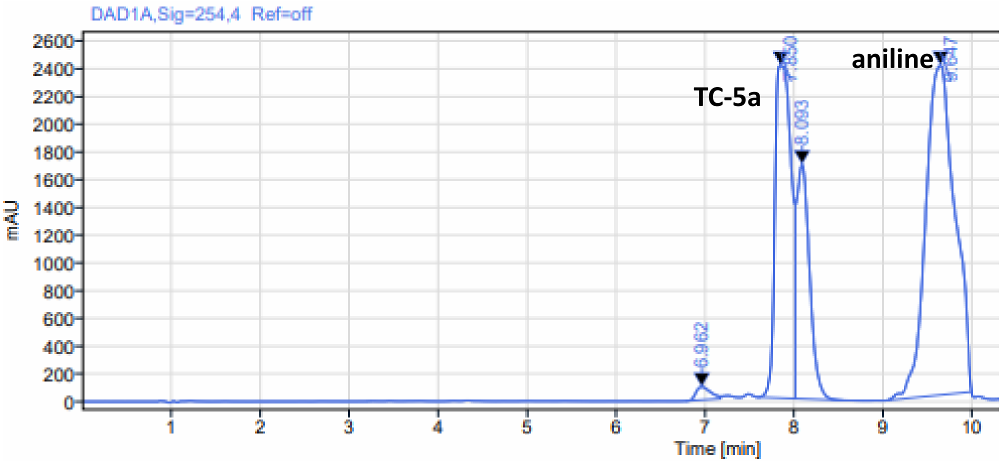

Figure S37. Crude analytical HPLC trace for product **TC-5a** with 2000 equiv amine, 2000 equiv isocyanide, 2000 equiv acid in MeOH/$CHCl_3$ (3:1,v/v) at 37°C (entry 5).

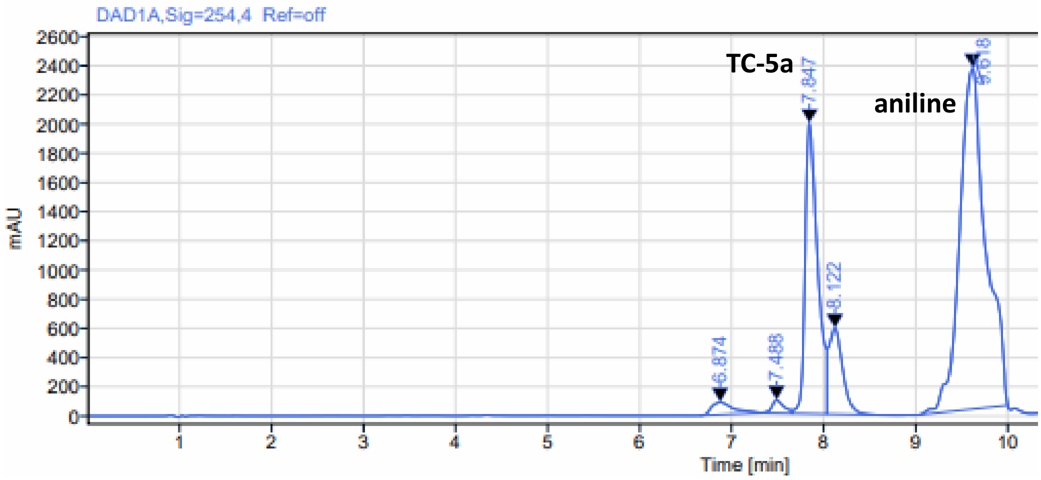

Figure S38. Crude analytical HPLC trace for product **TC-5a** with 2000 equiv amine, 2000 equiv isocyanide, 2000 equiv acid in MeOH/$CHCl_3$ (3:1,v/v) at rt (entry 6).

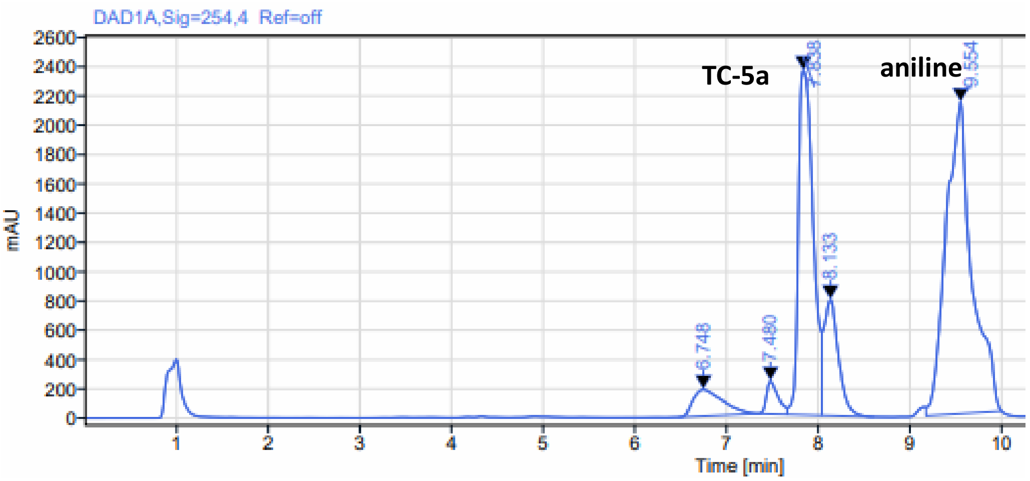

Figure S39. Crude analytical HPLC trace for product **TC-5a** with 2000 equiv amine, 2000 equiv isocyanide, 2000 equiv acid in MeOH/$CHCl_3$ (3:1,v/v) at rt with $ZnCl_2$ as catalyst (entry 7).

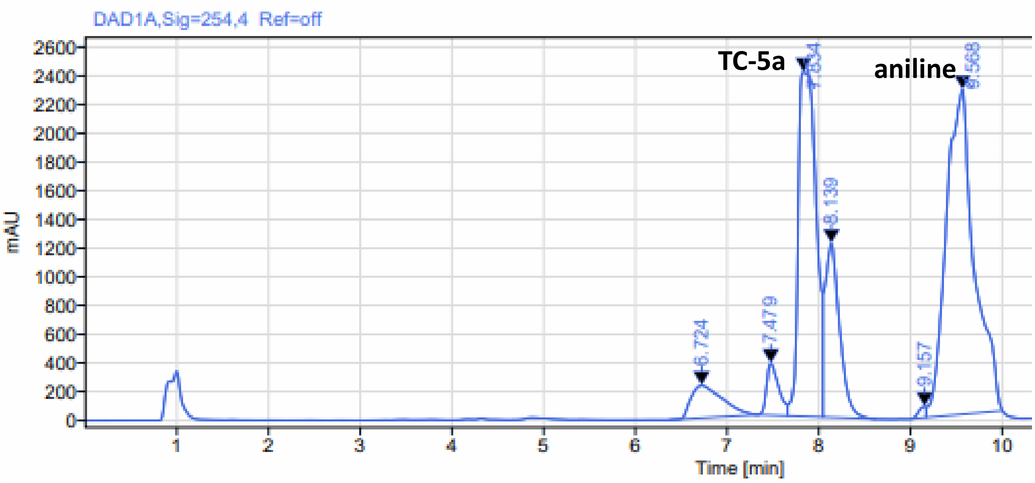

Figure S40. Crude analytical HPLC trace for product **TC-5a** with 2000 equiv amine, 2000 equiv isocyanide, 2000 equiv acid in MeOH/$CHCl_3$ (3:1,v/v) at rt with $MgCl_2$ as catalyst (entry 8).

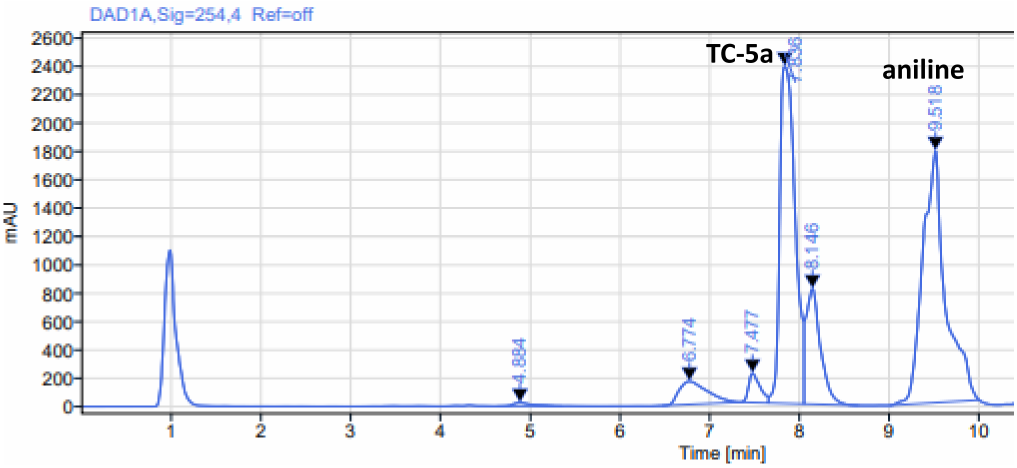

Figure S41. Crude analytical HPLC trace for product **TC-5a** with 2000 equiv amine, 2000 equiv isocyanide, 2000 equiv acid in MeOH/$CHCl_3$ (3:1,v/v) at rt with $FeCl_2$ as catalyst (entry 9).

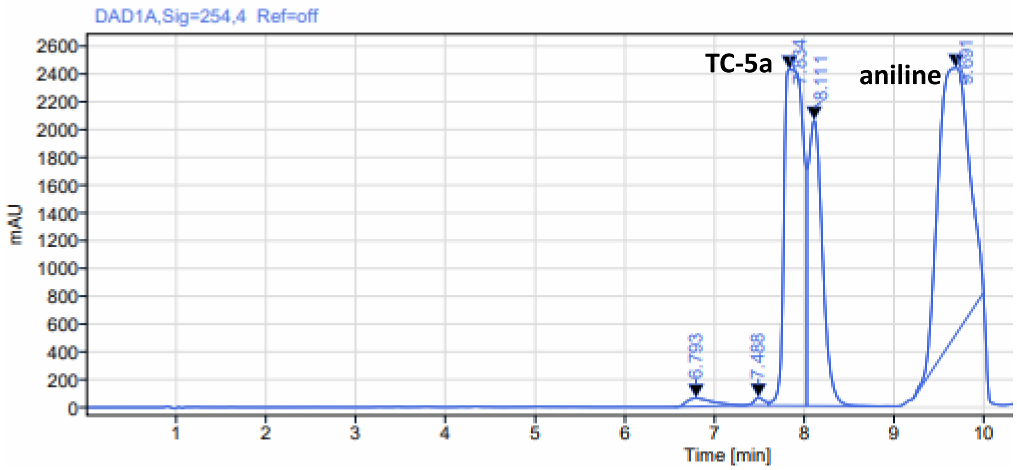

Figure S42. Crude analytical HPLC trace for product **TC-5a** with 2000 equiv amine, 2000 equiv isocyanide, 2000 equiv acid in EtOH/$CHCl_3$ (3:1,v/v) at 50°C (entry 10).

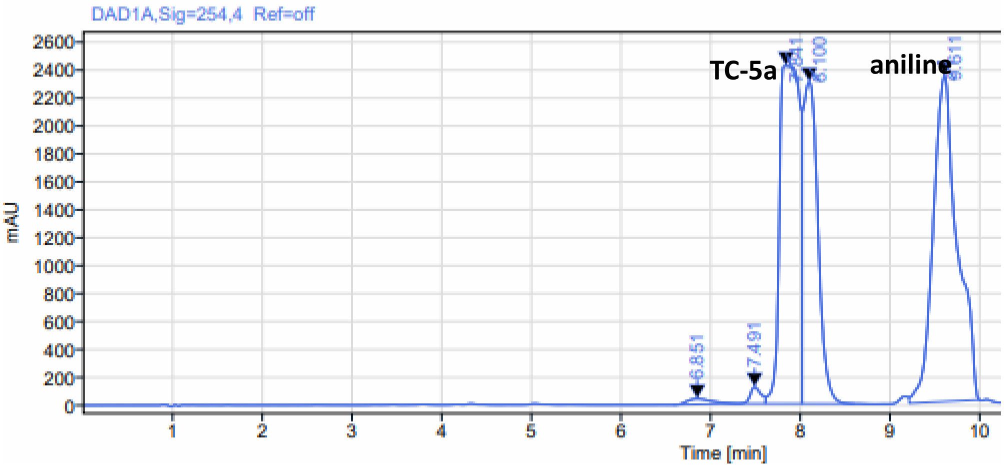

Figure S43. Crude analytical HPLC trace for product **TC-5a** with 2000 equiv amine, 2000 equiv isocyanide, 2000 equiv acid in iPrOH/$CHCl_3$ (3:1,v/v) at 50°C (entry 11).

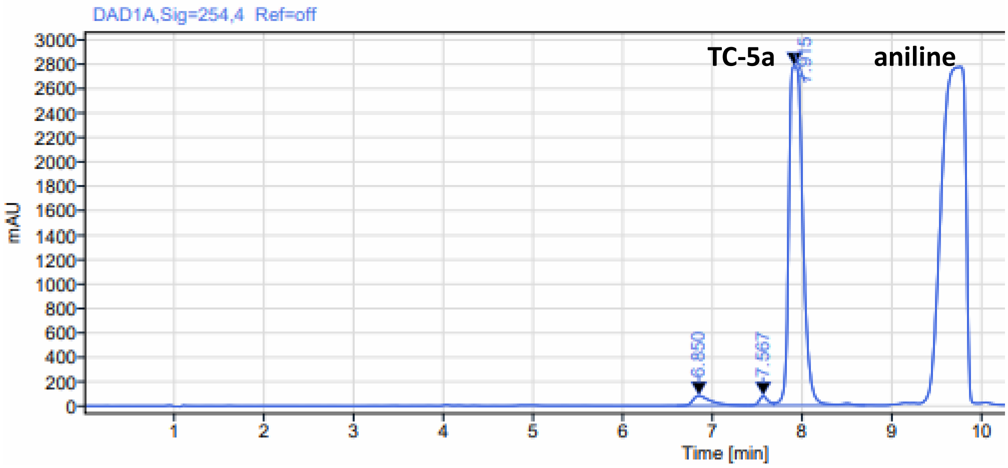

Figure S44. Crude analytical HPLC trace for product **TC-5a** with 3000 equiv amine, 3000 equiv isocyanide, 3000 equiv acid in CF_3CH_2OH /$CHCl_3$ (3:1,v/v) at 60°c for 2d (entry 12).

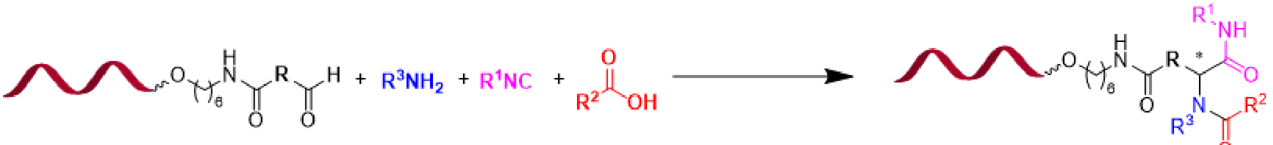

TC-1 2 3 4 TC-5

Table S4. MALDI-MS data of the reaction scope for the Ugi four-component reaction with 10merTC-coupled aldehydes.

			**MALDI-MS m/z**			
No.	R	R^1	R^2	R^3	MS calc.	MS found
TC-5a	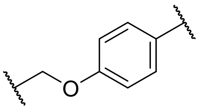	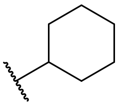	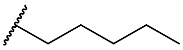	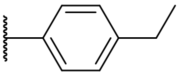	3580.0	3581.4
TC-5b	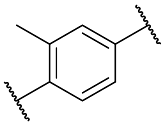	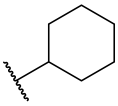	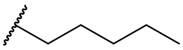	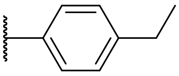	3564.0	3564.6
TC-5c	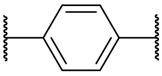	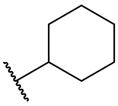	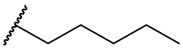	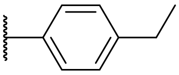	3549.0	3549.8
TC-5d	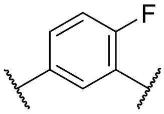	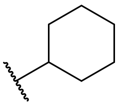	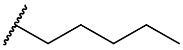	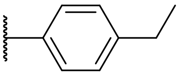	3568.0	3568.9
TC-5e	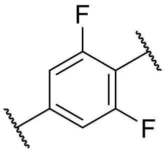	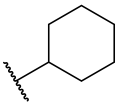	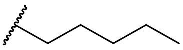	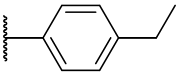	3585.0	3586.0
TC-5f	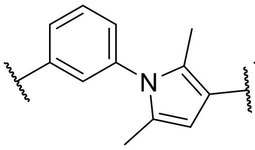	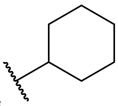	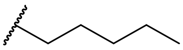	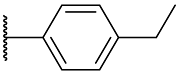	3643.0	3644.0
TC-5g	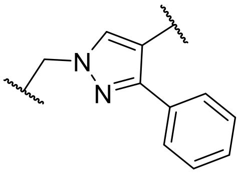	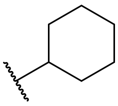	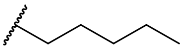	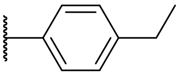	3630.0	3630.7
TC-5h	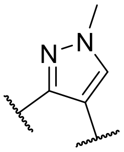	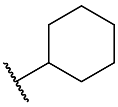	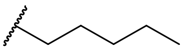	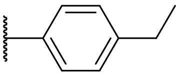	3554.0	3554.7

TC-5i	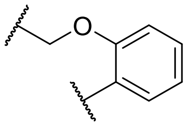	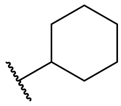	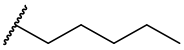	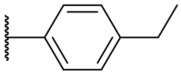	3580.0	3581.0
TC-5j	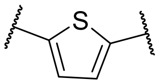	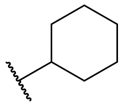	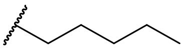	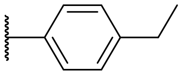	3555.0	3555.9
TC-5k	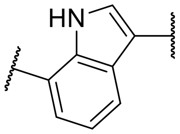	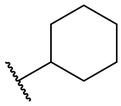	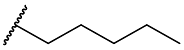	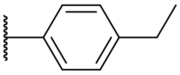	3588.0	3629.7 $[M+K^+]$
TC-5l	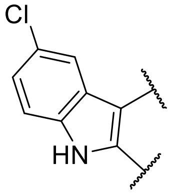	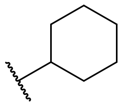	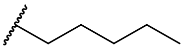	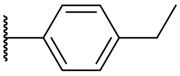	3620.0	3622.7
TC-5m	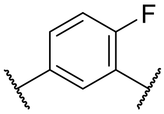	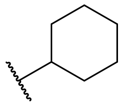	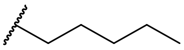	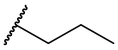	3500.0	3501.6
TC-5n	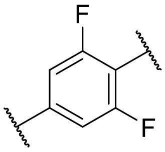	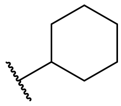	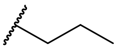	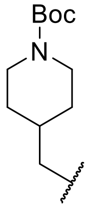	3646.0	3647.1
TC-5o	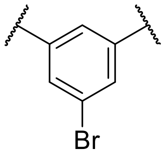	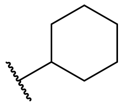	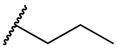	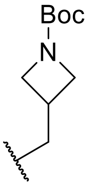	3661.0	3661.9
TC-5p	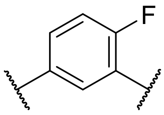	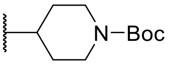	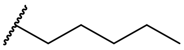	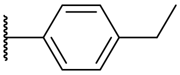	3686.0	3687.0
TC-5q	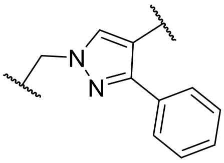	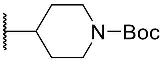	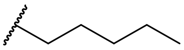	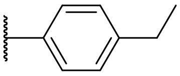	3730.0	3732.2
TC-5r	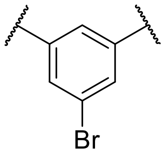	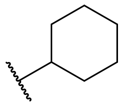	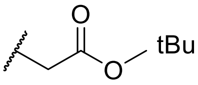	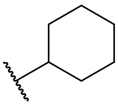	3578.0	3579.9

TC-5s	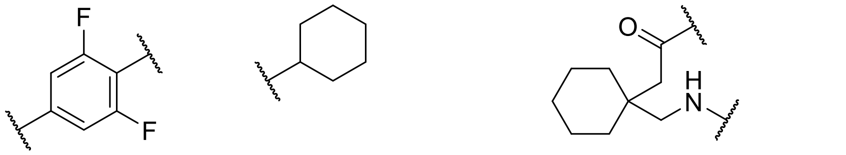	3514.0	3514.5
TC-5t	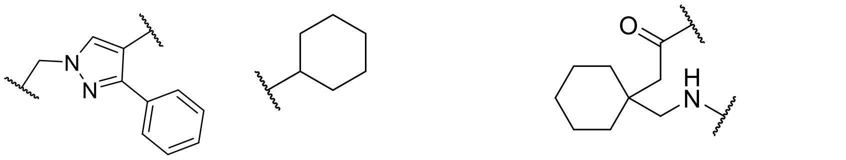	3558.0	3560.0
TC-5u	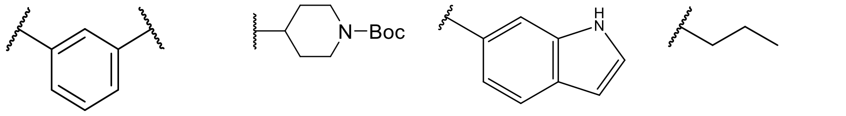	3628.0	3628.7
TC-5v	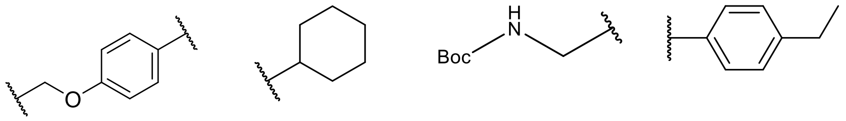	3634.0	3634.4
TC-5w	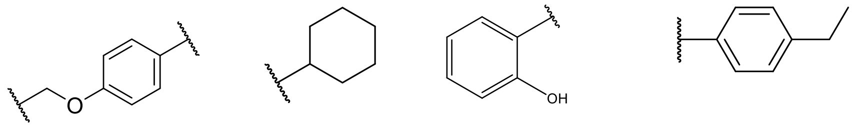	3597.0	3597.4
TC-5x	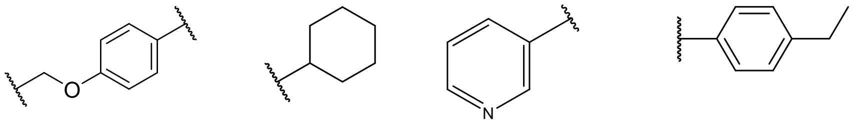	3582.0	3581.7
TC-5y	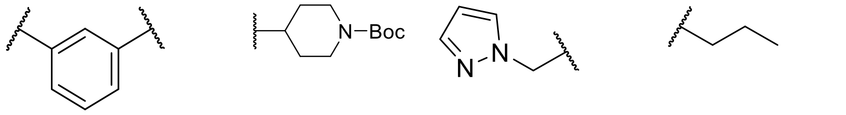	3594.0	3593.6
TC-5z	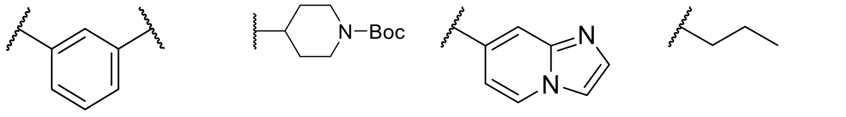	3630.0	3630.3
TC-5aa	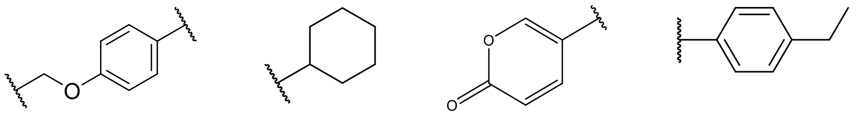	3598.0	3632.3 [M+ 2*NH_4^+]
TC-5ab	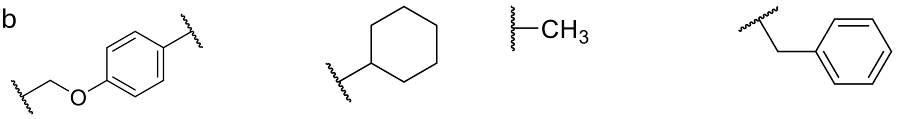	3504.0	3504.4
TC-5ac	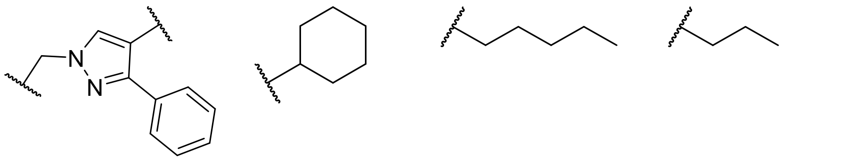	3562.0	3563.9
TC-5ad	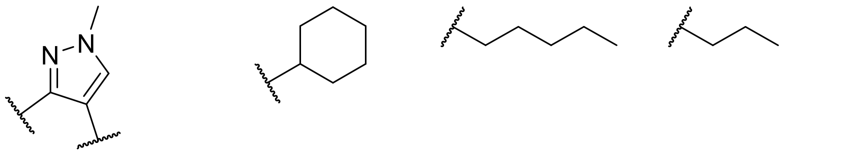	3486.0	3487.7

TC-5ae	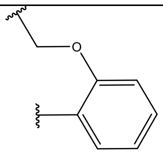	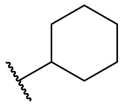	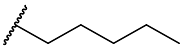	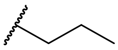	3512.0	3513.7
TC-5af	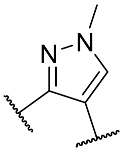	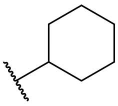	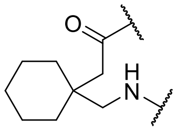		3482.0	3484.0
TC-5ag	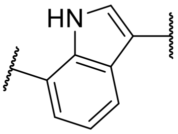	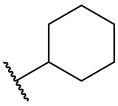	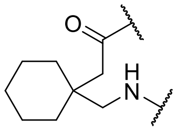		3517.0	3518.6
TC-5ah	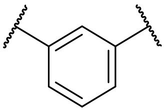	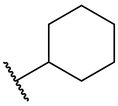	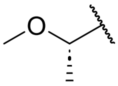	CH_3	3443.0	3444.0
TC-5ai	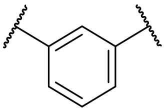	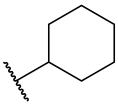	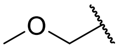	CH_3	3429.0	3429.9
TC-5aj	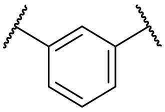	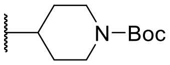	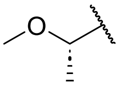	CH_3	3544.0	3545.0
TC-5ak	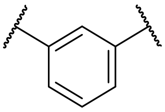	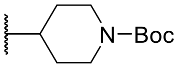	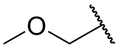	CH_3	3530.0	3530.8
TC-5al	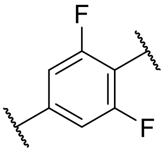	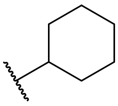	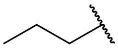	CH_3	3462.5	3464.1
TC-5am	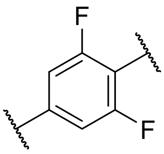	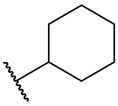	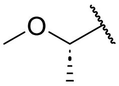	CH_3	3479.0	3479.9
TC-5an	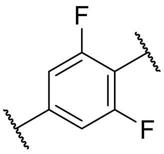	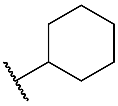	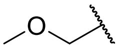	CH_3	3465.0	3465.7

TC-5ao	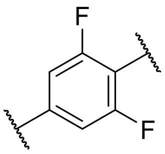	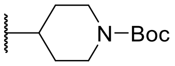	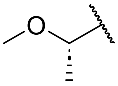	CH_3	3580.0	3581.0
TC-5ap	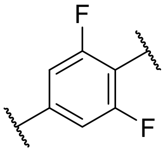	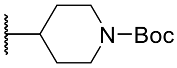	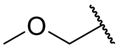	CH_3	3566.0	3567.1
TC-5aq	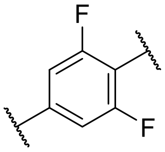	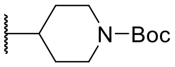	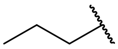	CH_3	3564.0	3565.0
TC-5ar	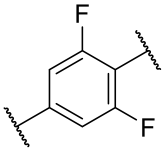	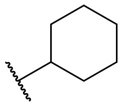	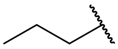	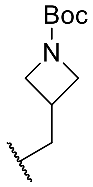	3618.0	3619.3
TC-5as	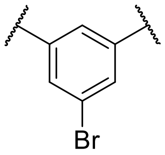	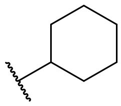	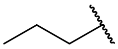	CH_3	3506.0	3507.7
TC-5at	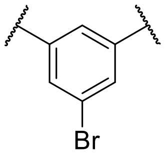	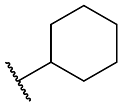	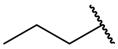	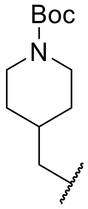	3689.0	3691.0
TC-5au	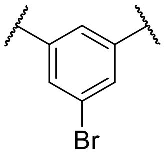	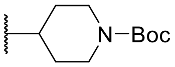	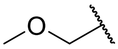	CH_3	3609.0	3610.9
TC-5av	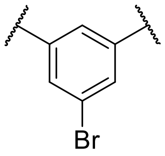	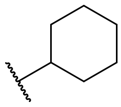	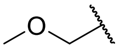	CH_3	3507.0	3510.0
TC-5aw	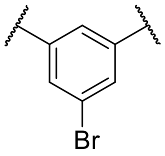	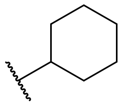	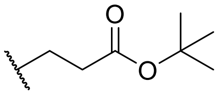	CH_3	3591.0	3593.9
TC-5ax	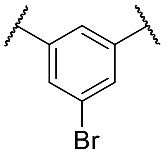	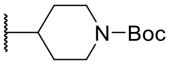	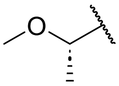	CH_3	3622.0	3624.9

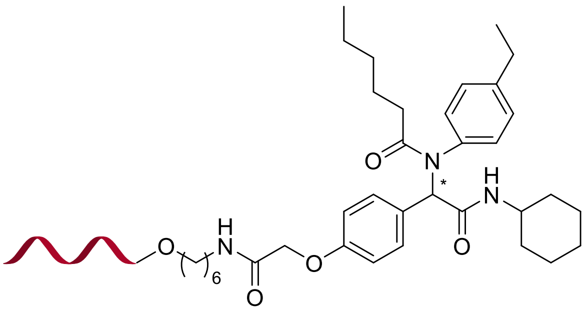

TC-5a

MS calc. 3580.0; found: 3581.4

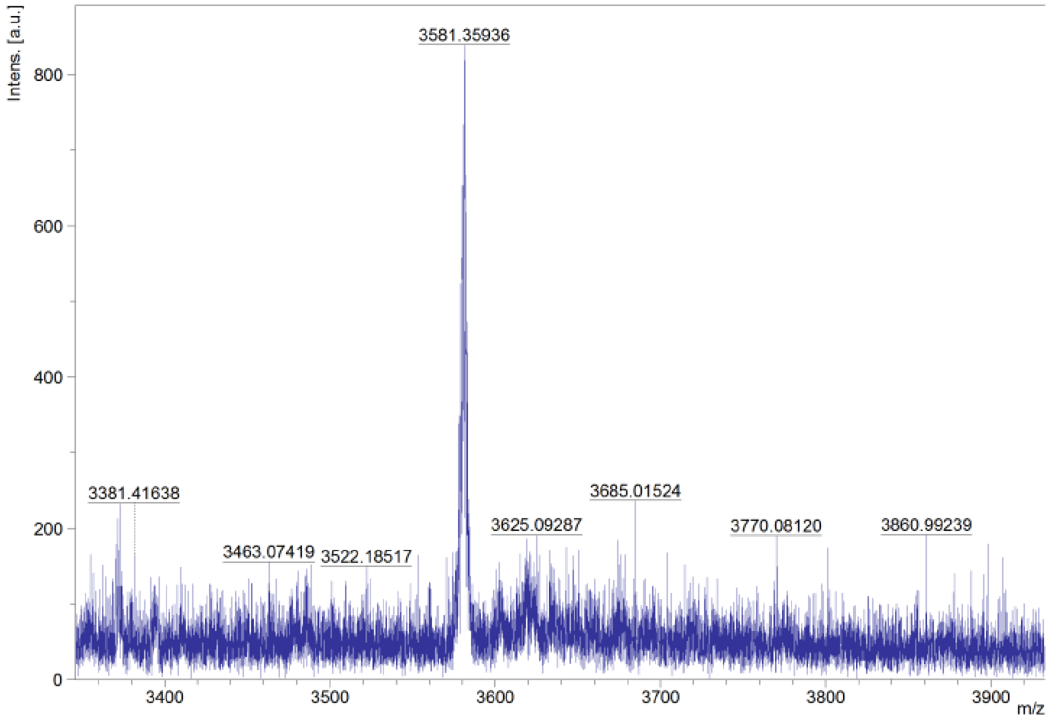

crude

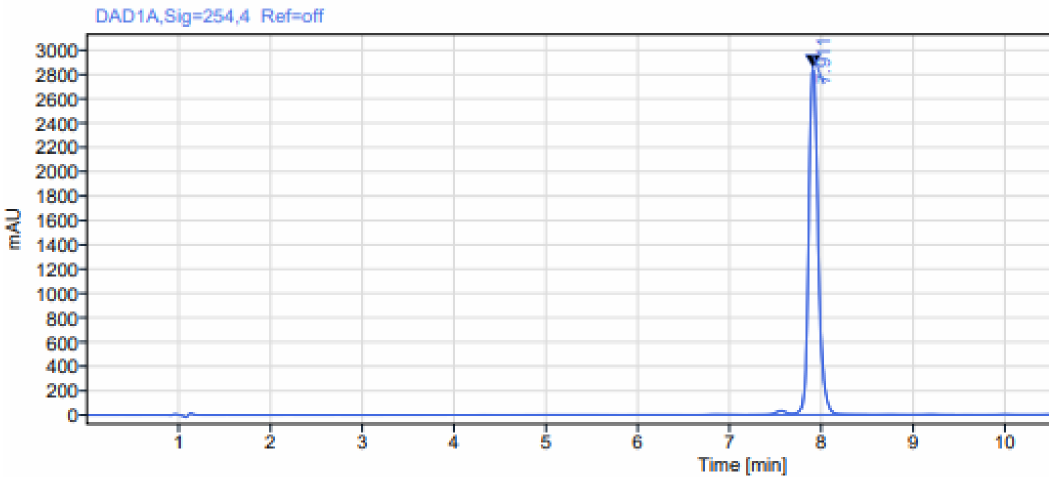

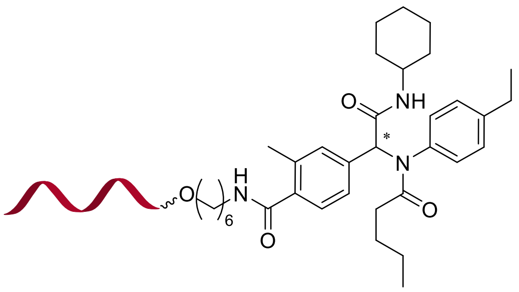

TC-5b

MS calc. 3564.0; found: 3564.6

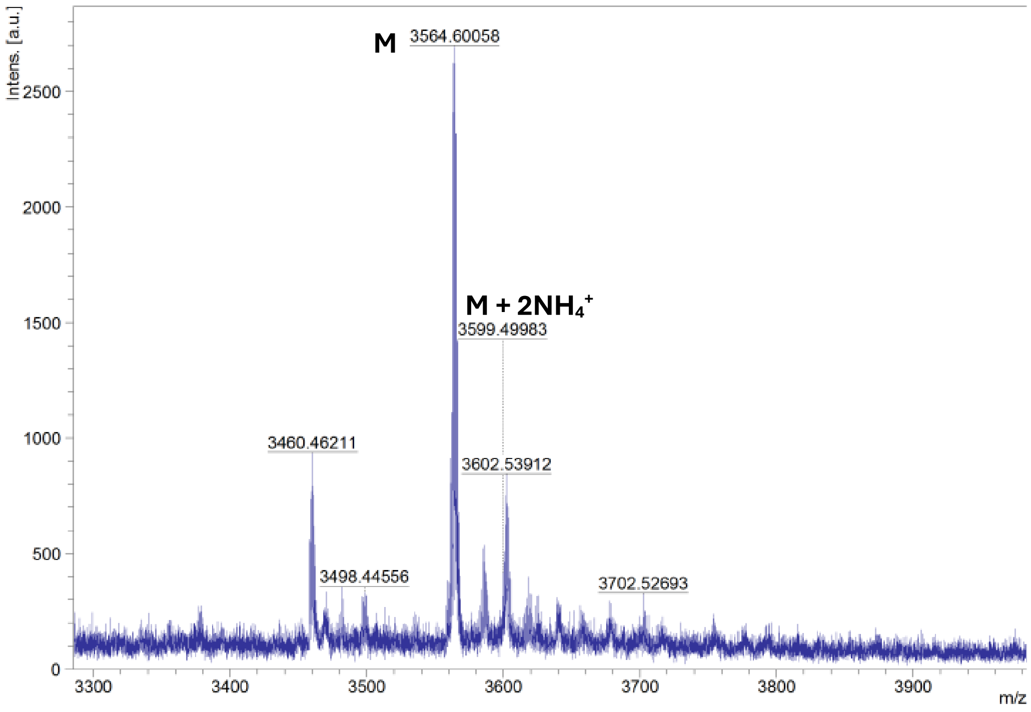

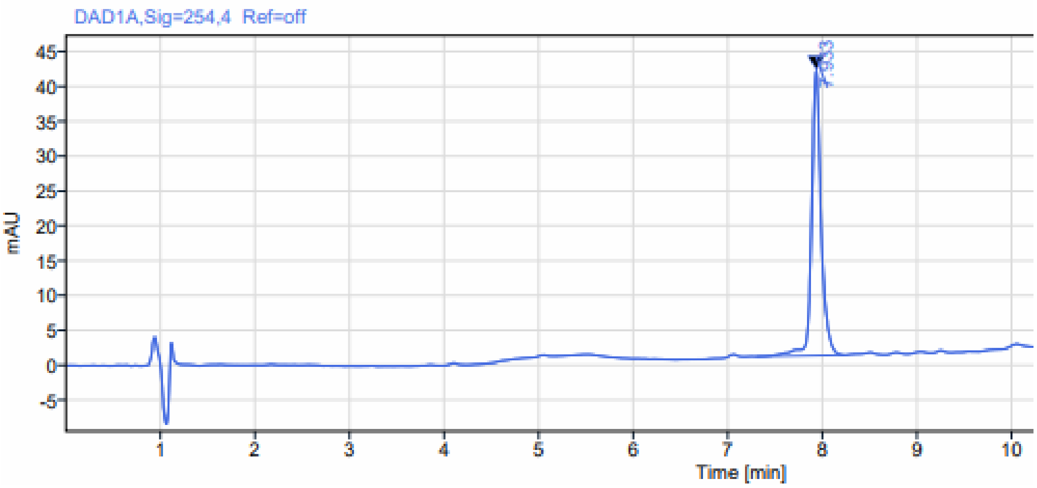

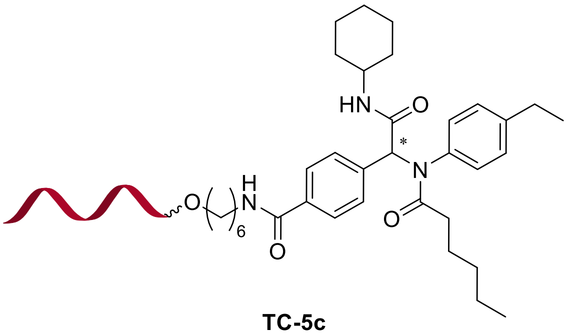

TC-5c

MS calc. 3549.0; found: 3549.8

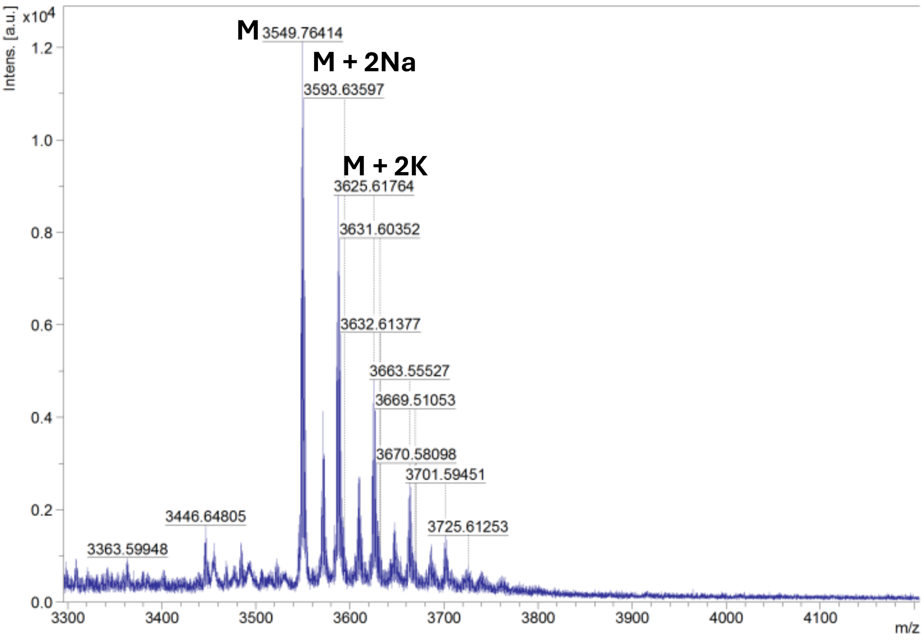

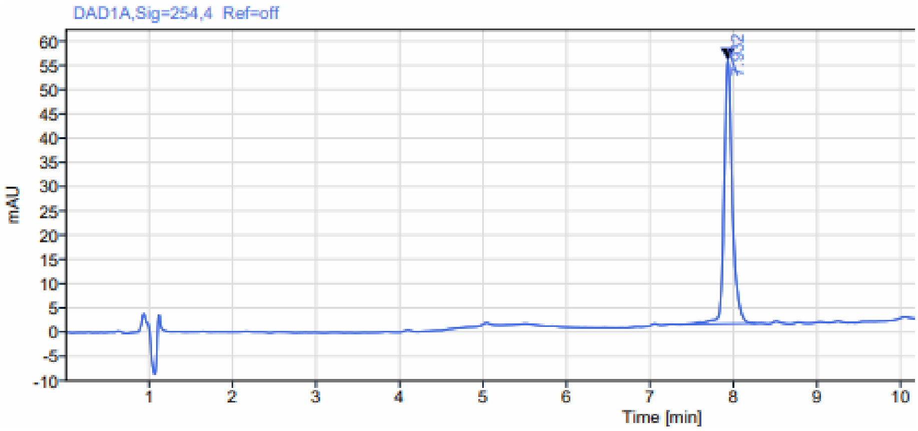

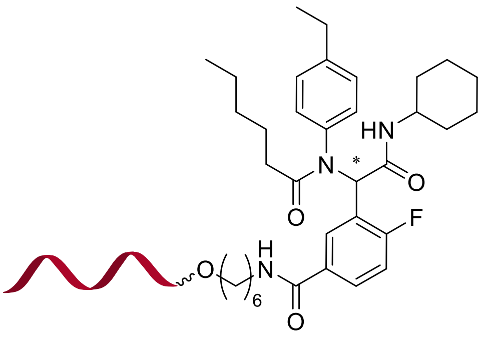

TC-5d

MS calc. 3568.10; found: 3568.9

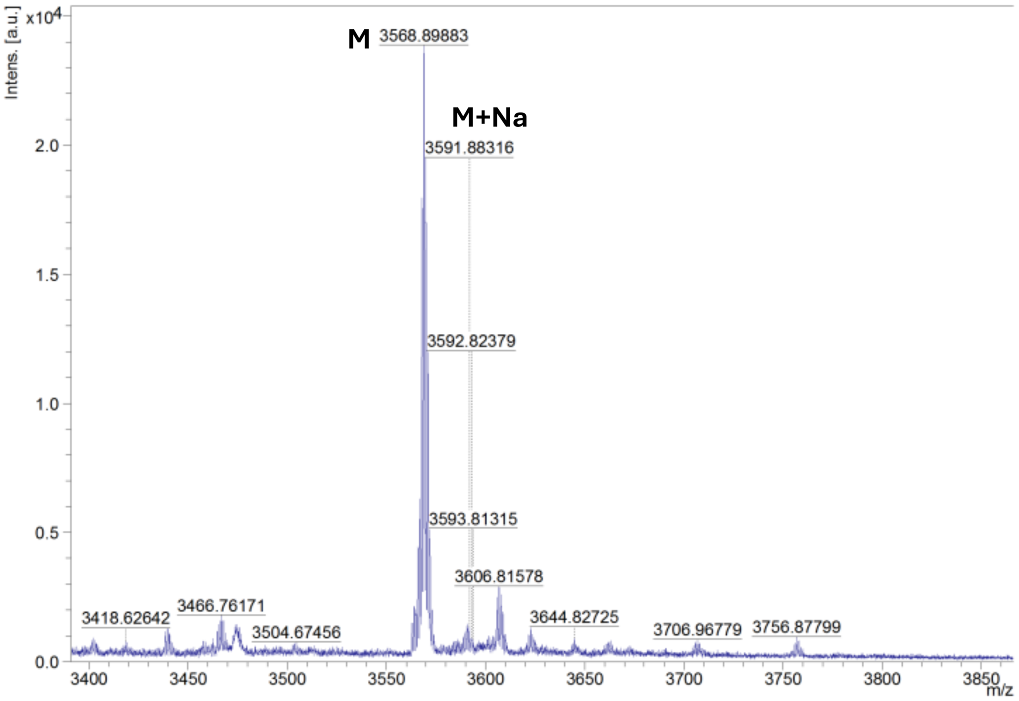

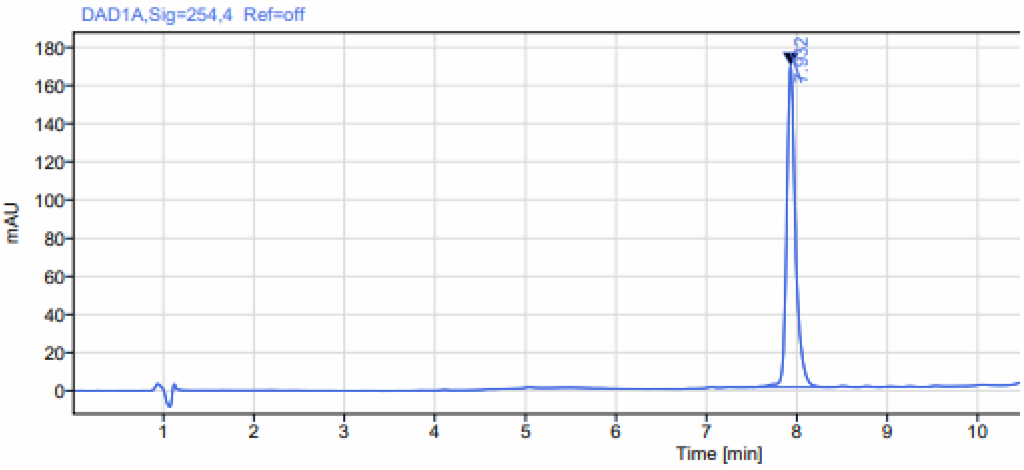

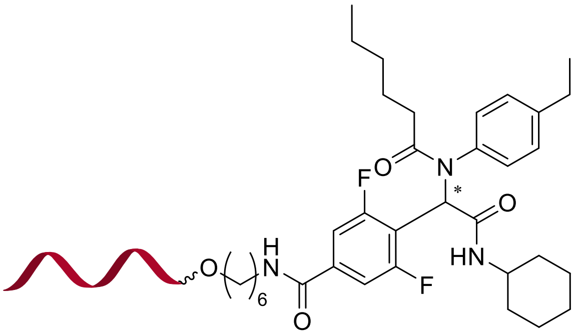

TC-5e

MS calc. 3585.0; found: 3586.0

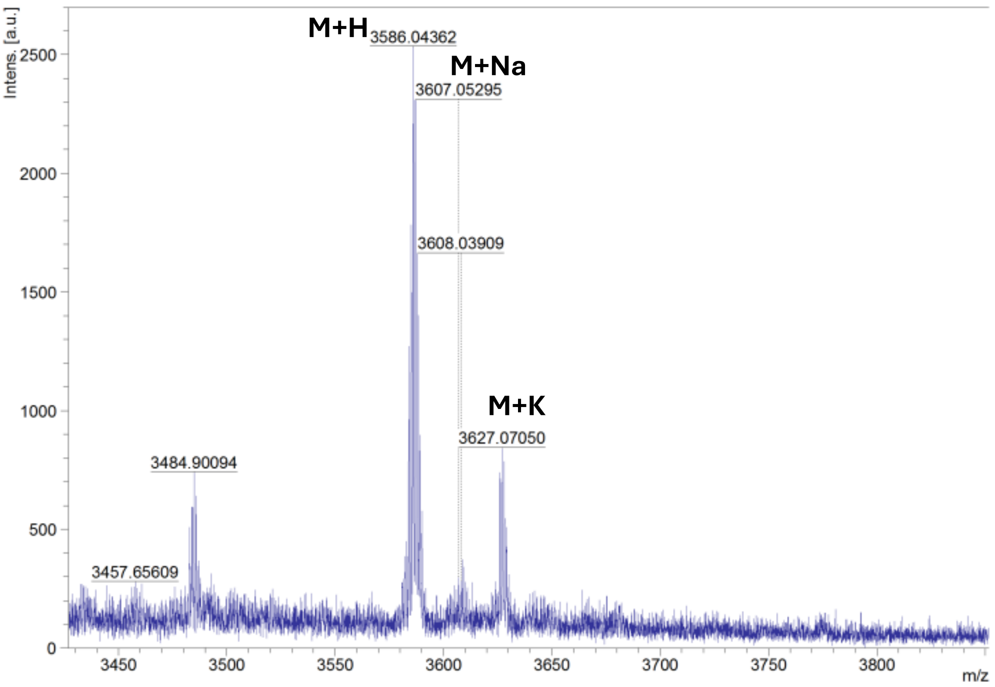

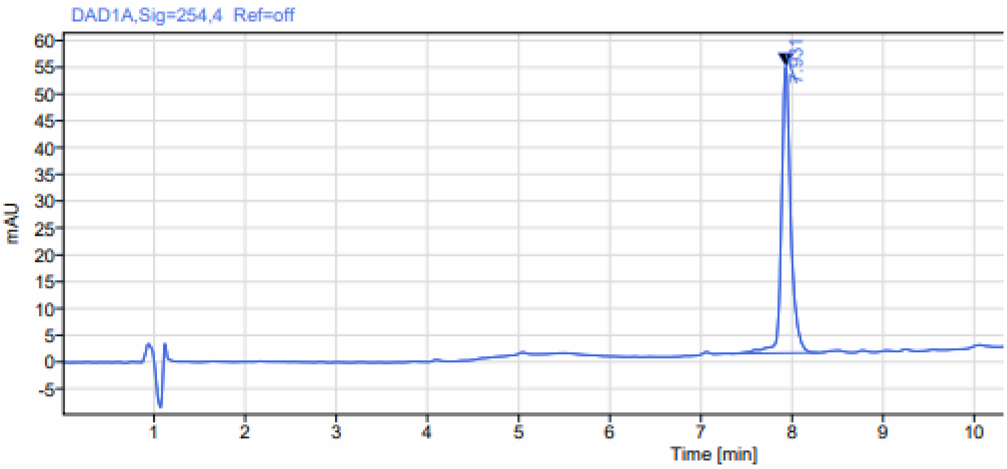

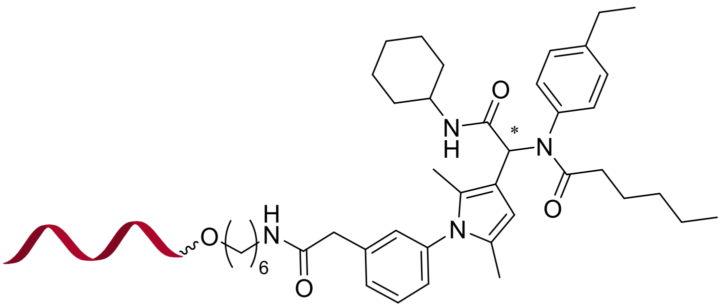

TC-5f

MS calc. 3643.0; found: 3644.0

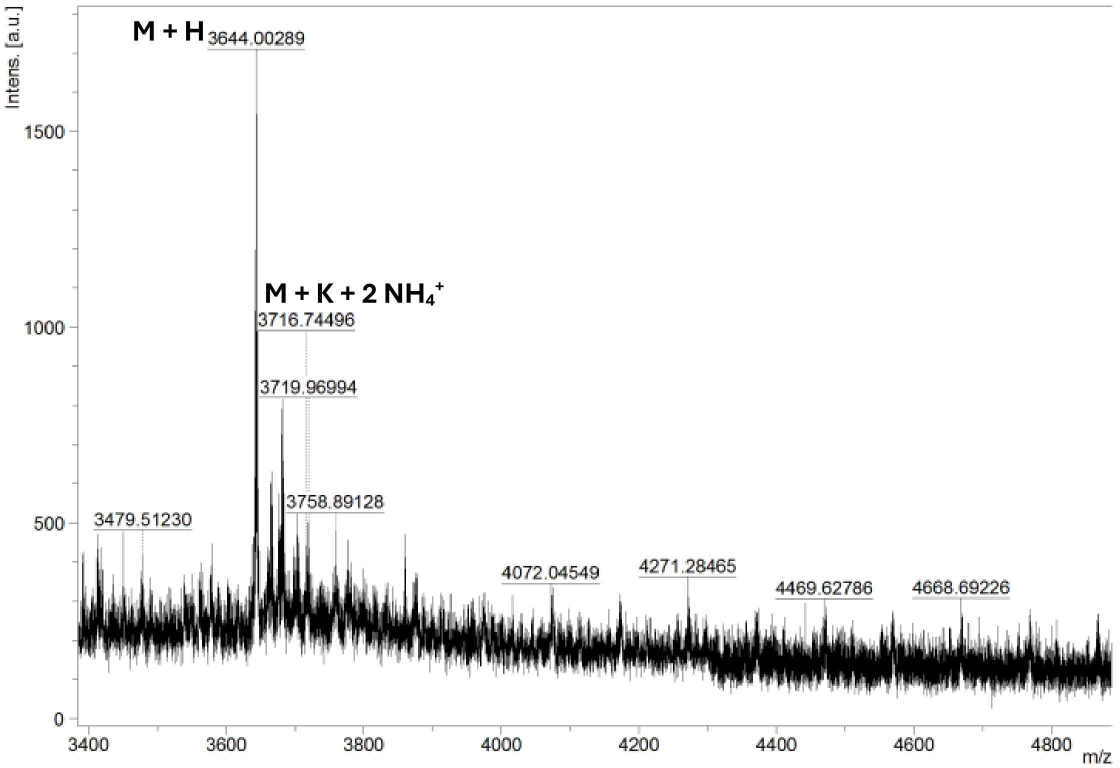

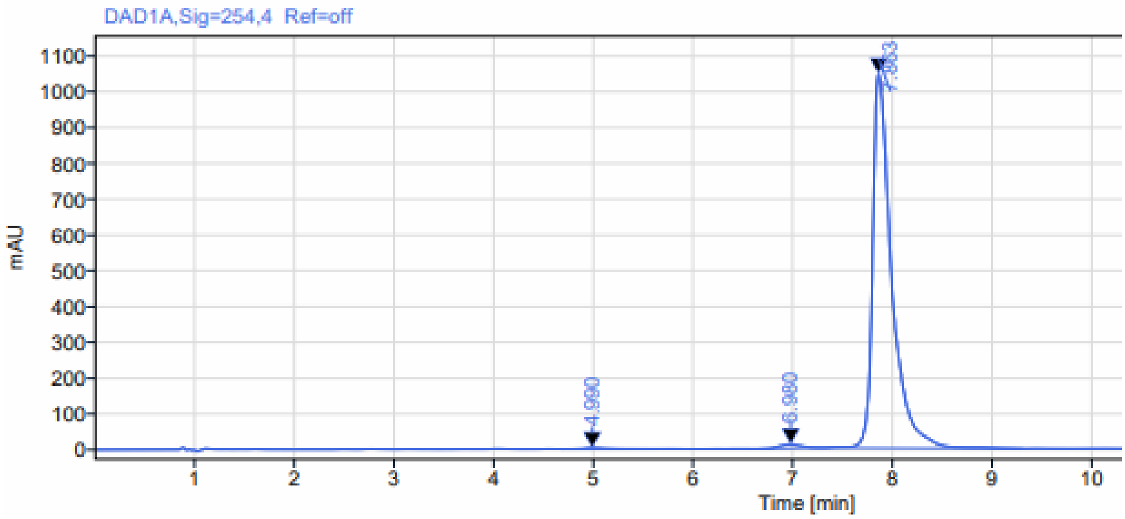

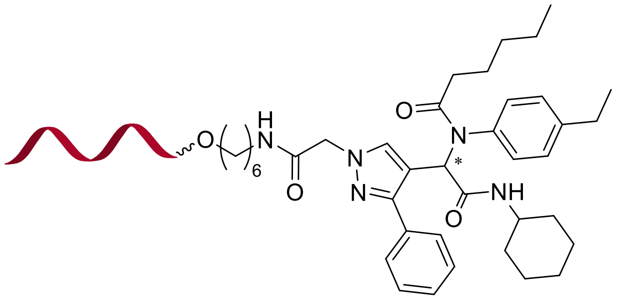

TC-5g

MS calc. 3630.0; found: 3630.7

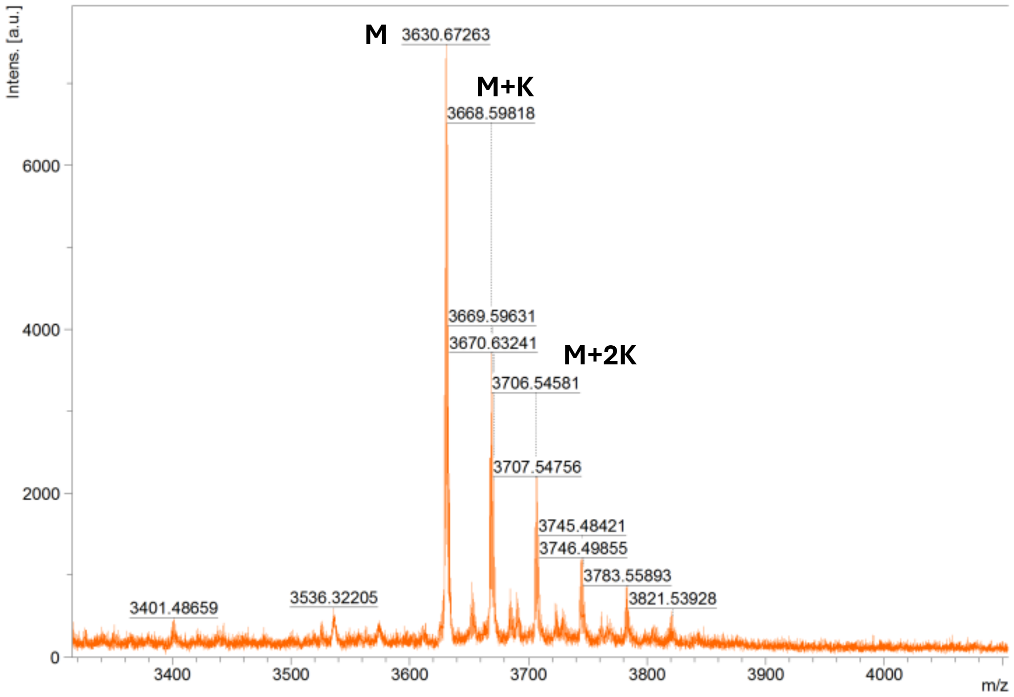

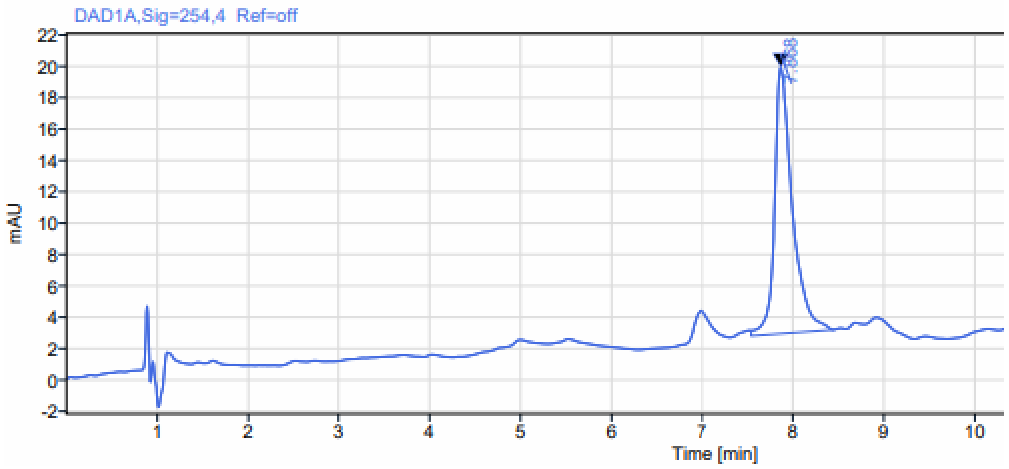

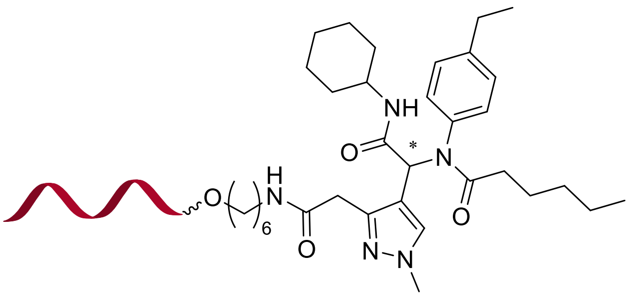

TC-5h

MS calc. 3554.0; found: 3554.7

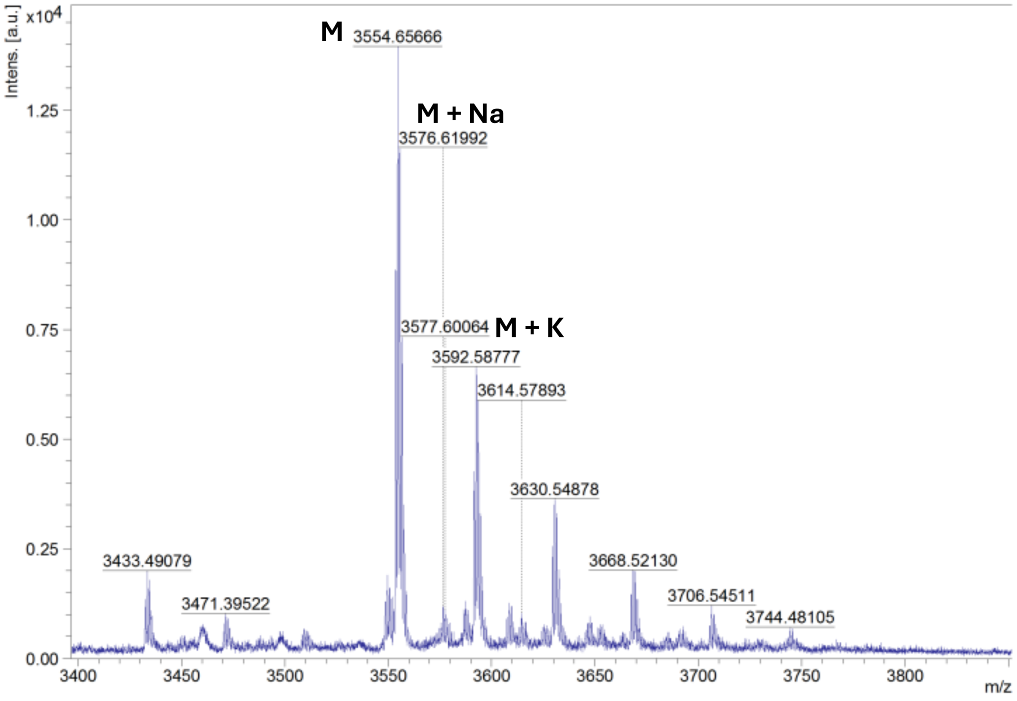

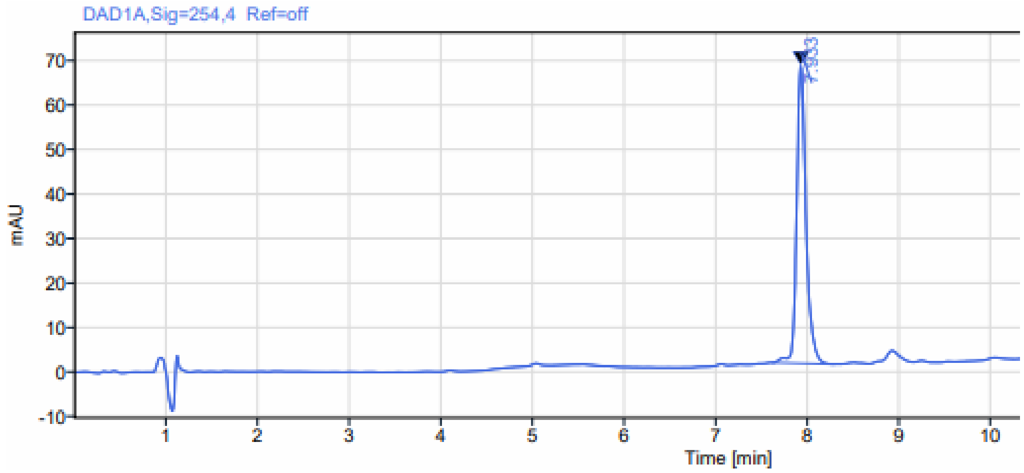

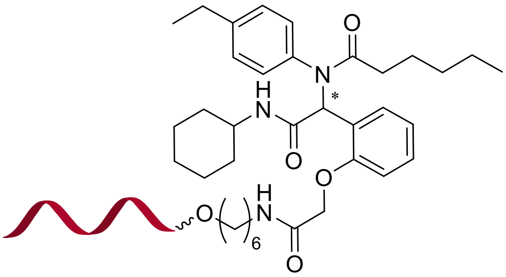

TC-5i

MS calc. 3580.0; found: 3581.0

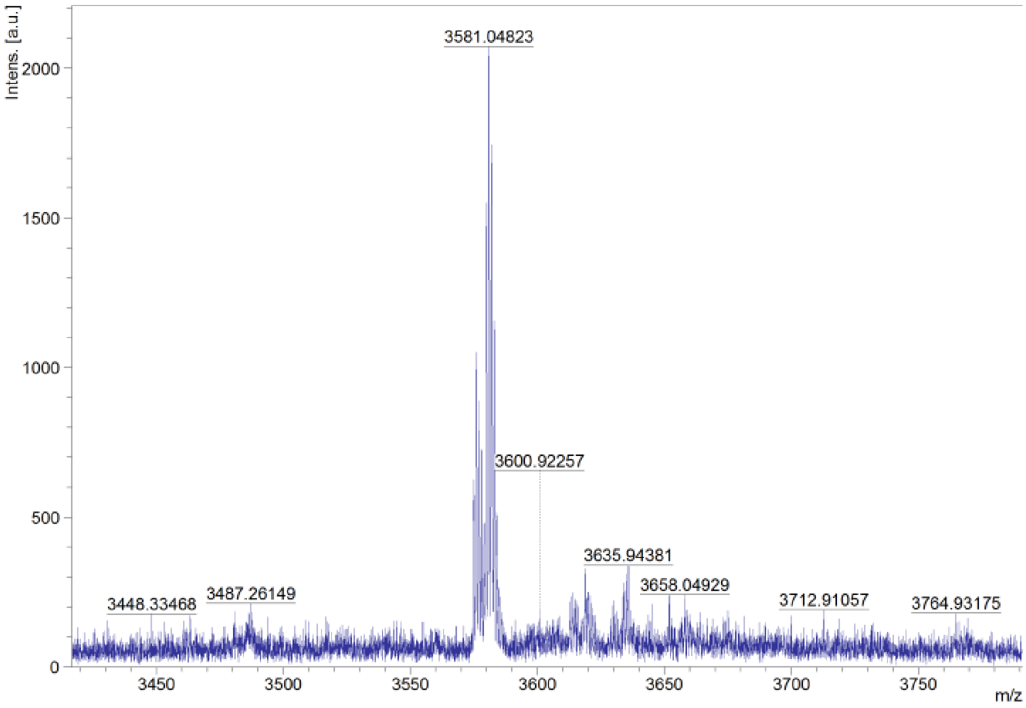

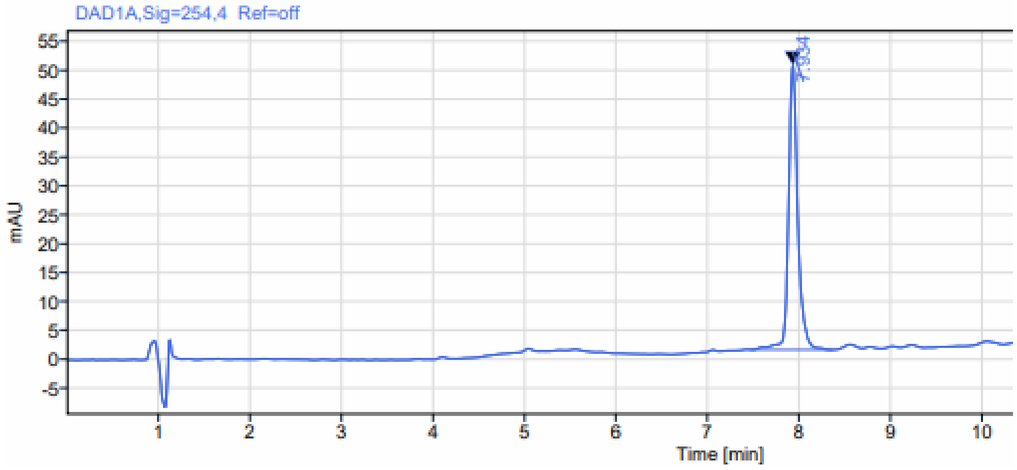

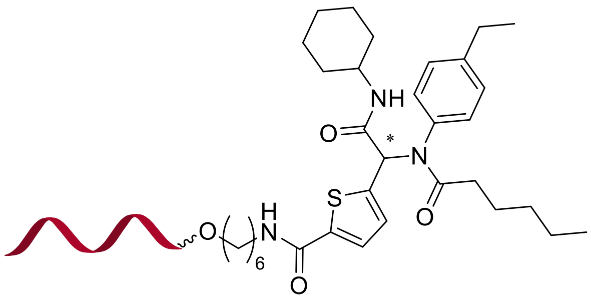

TC-5j

MS calc. 3555.0; found: 3555.9

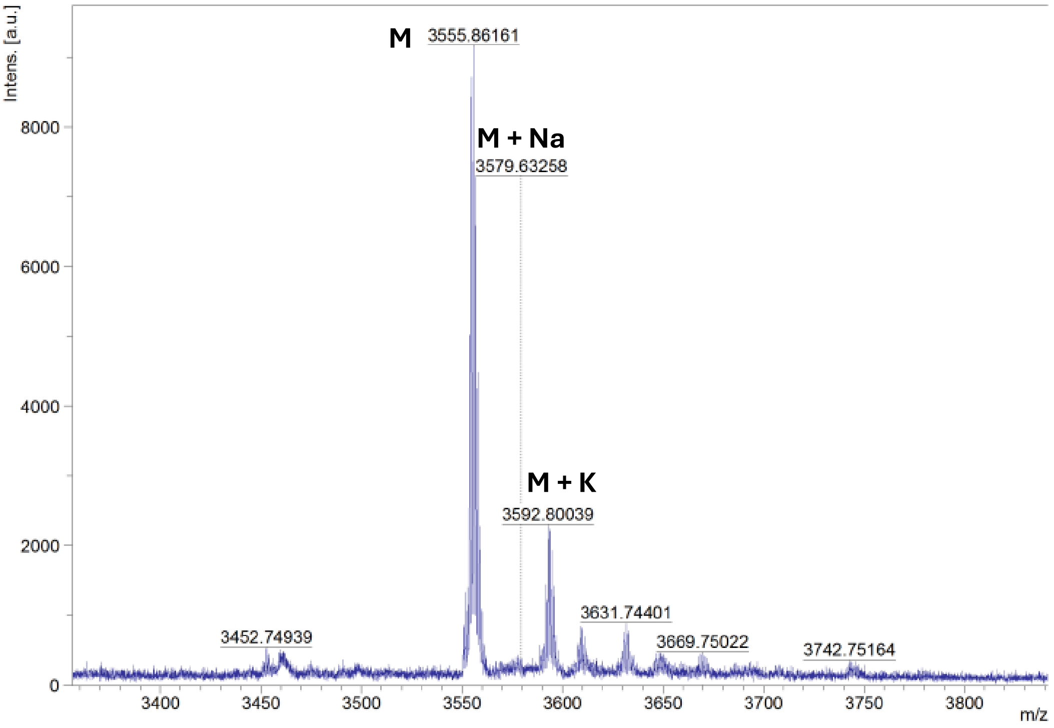

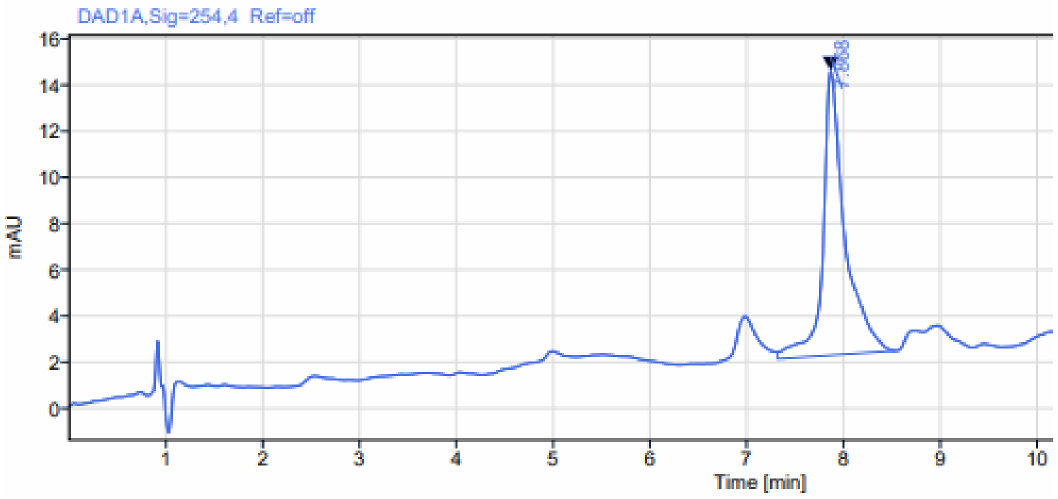

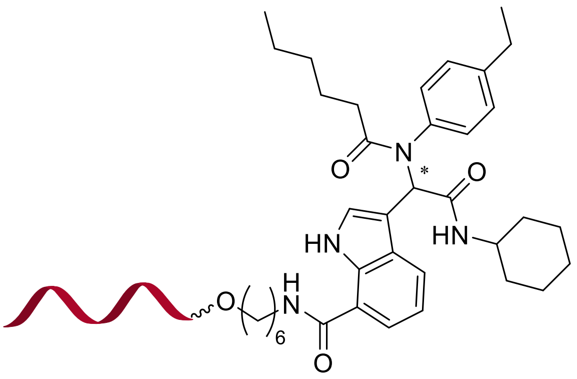

TC-5k

MS calc. 3588.0; found: 3629.7 [M+K^{+}]

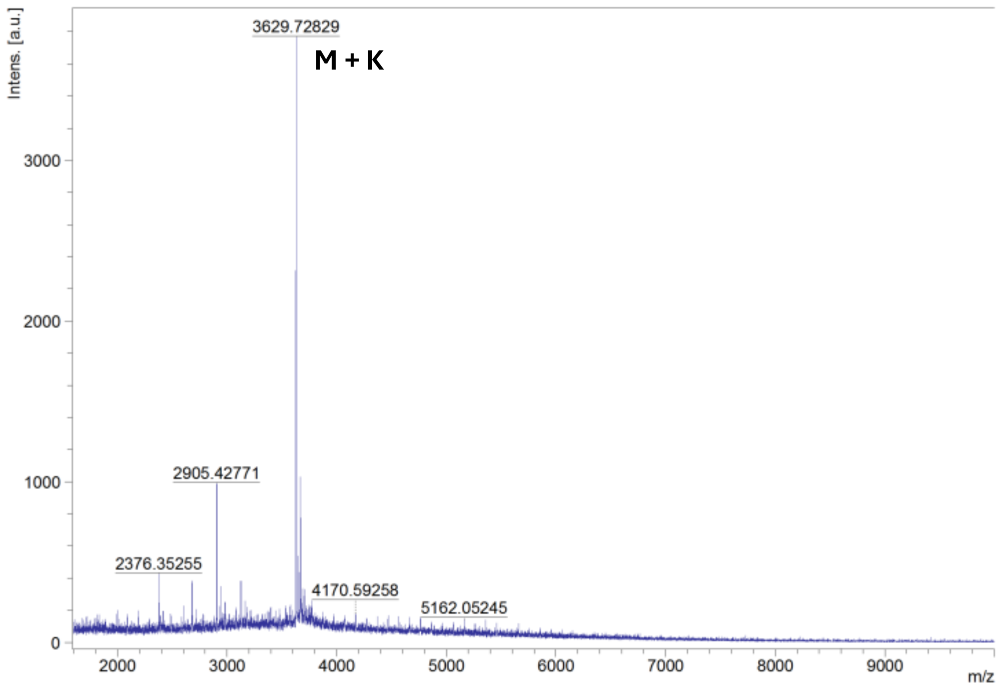

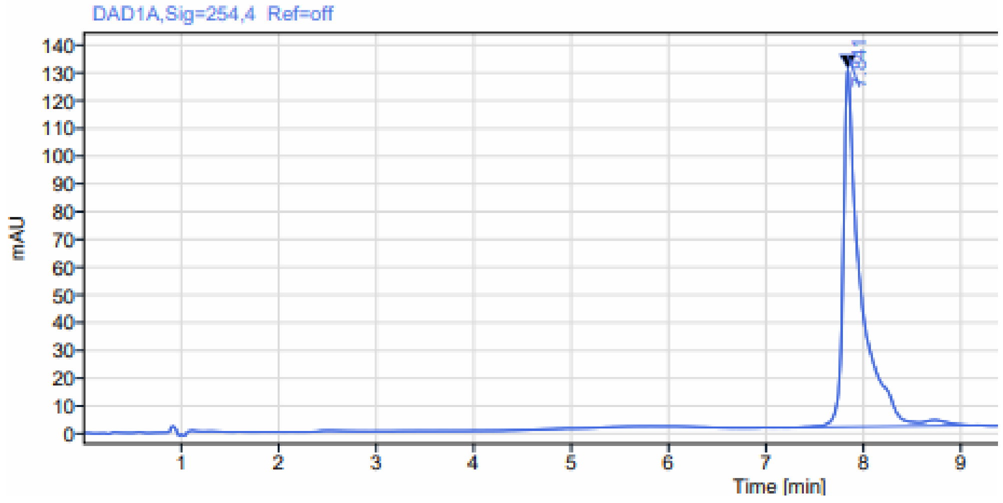

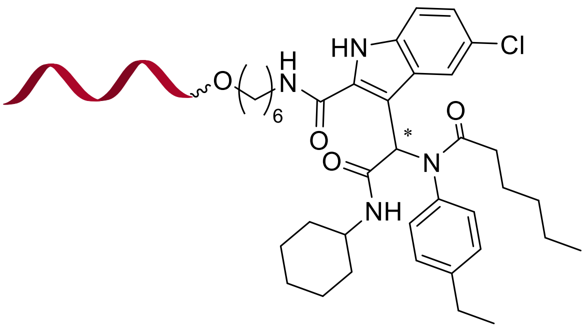

TC-5l

MS calc. 3620.00; found: 3622.7

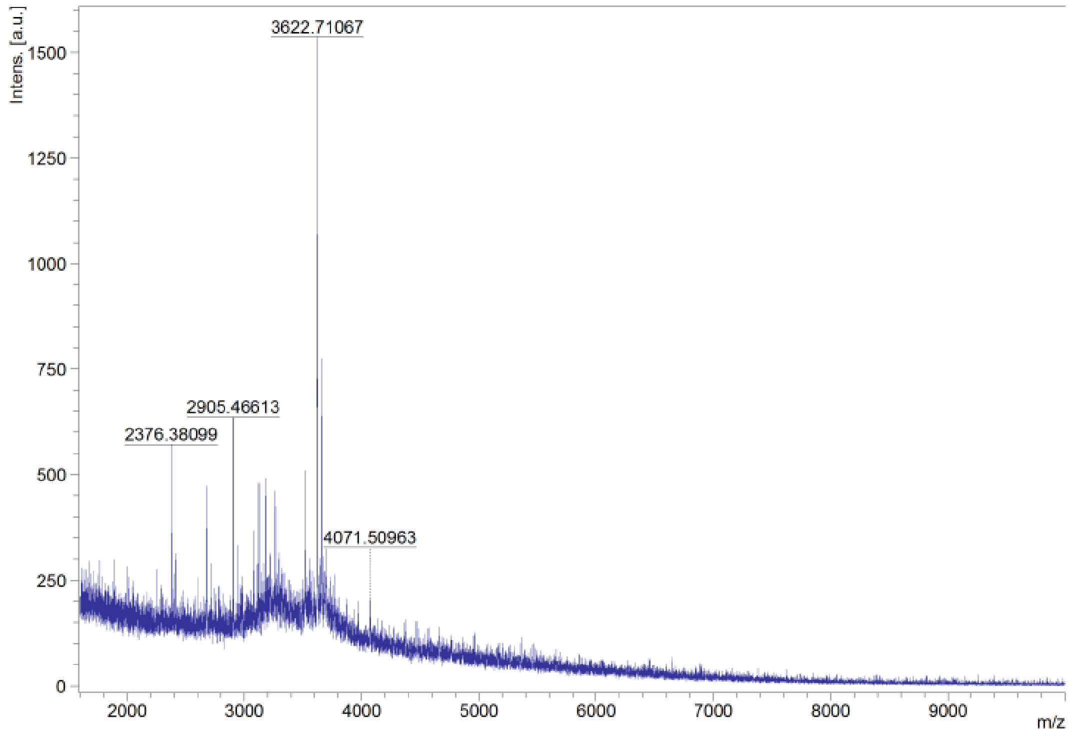

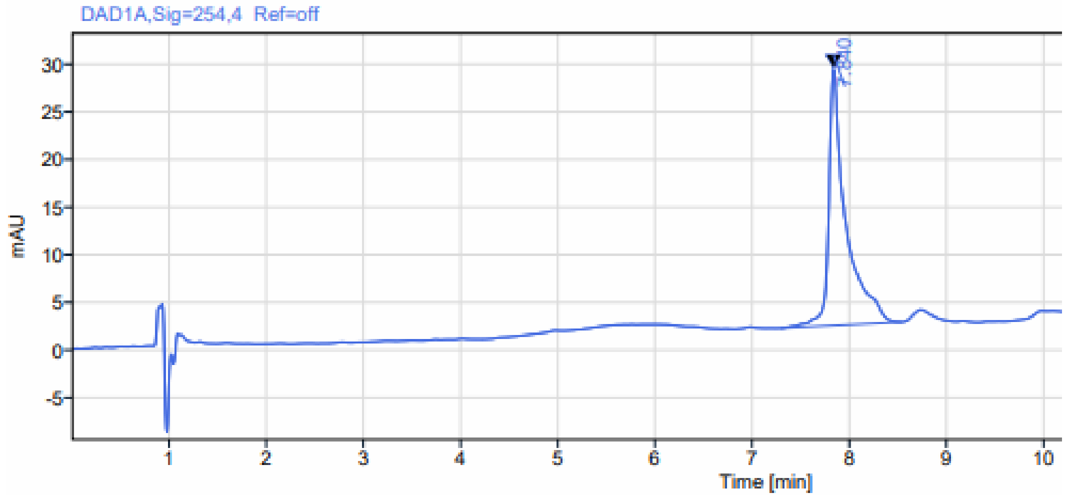

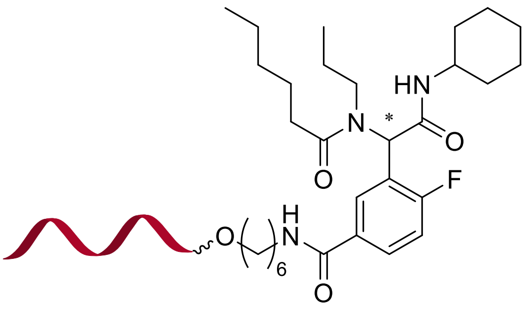

TC-5m

MS calc. 3500.0; found: 3501.6

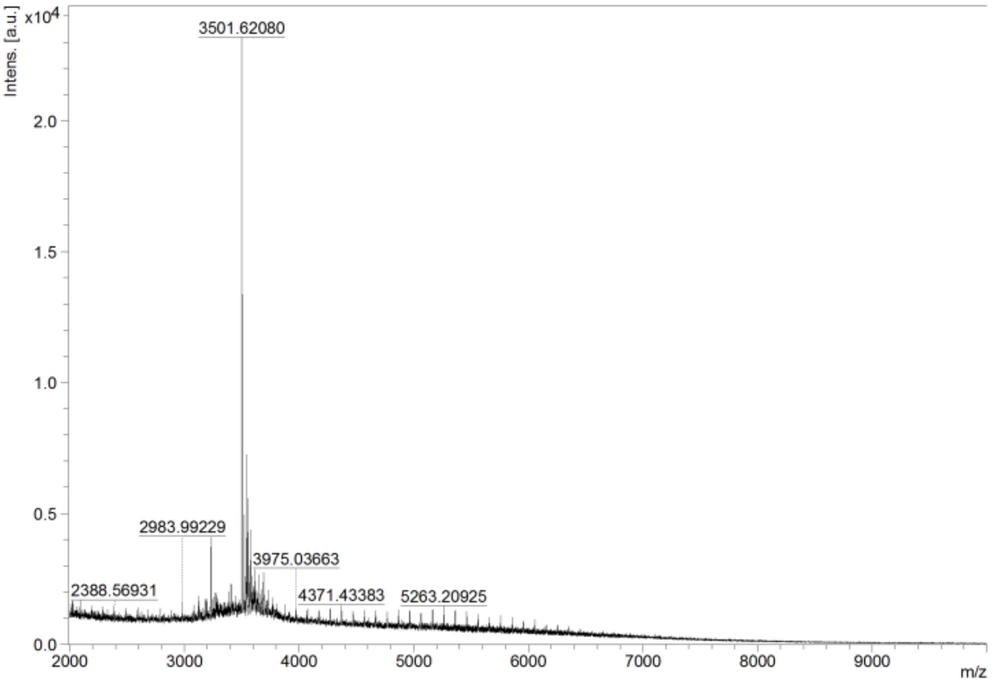

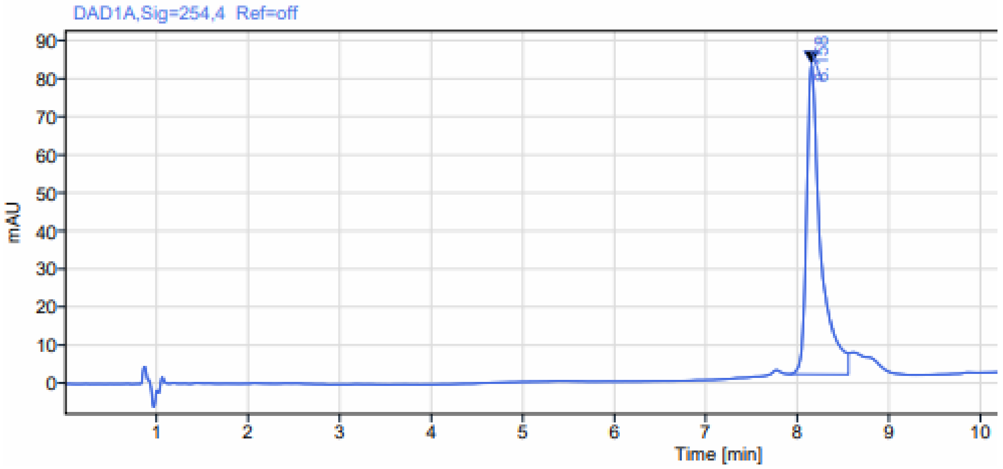

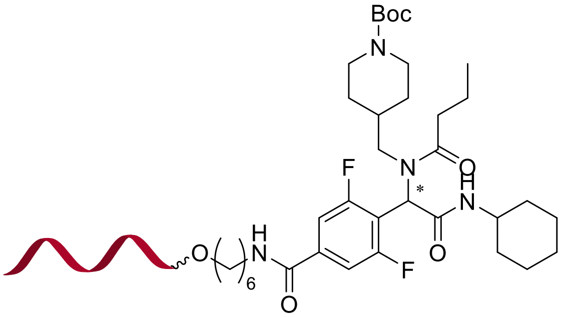

TC-5n

MS calc. 3646.0; found: 3647.1

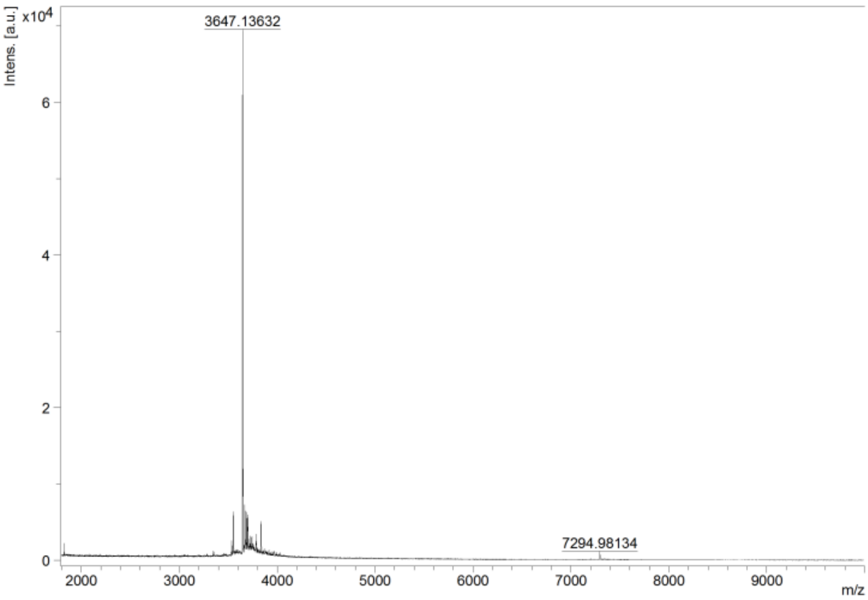

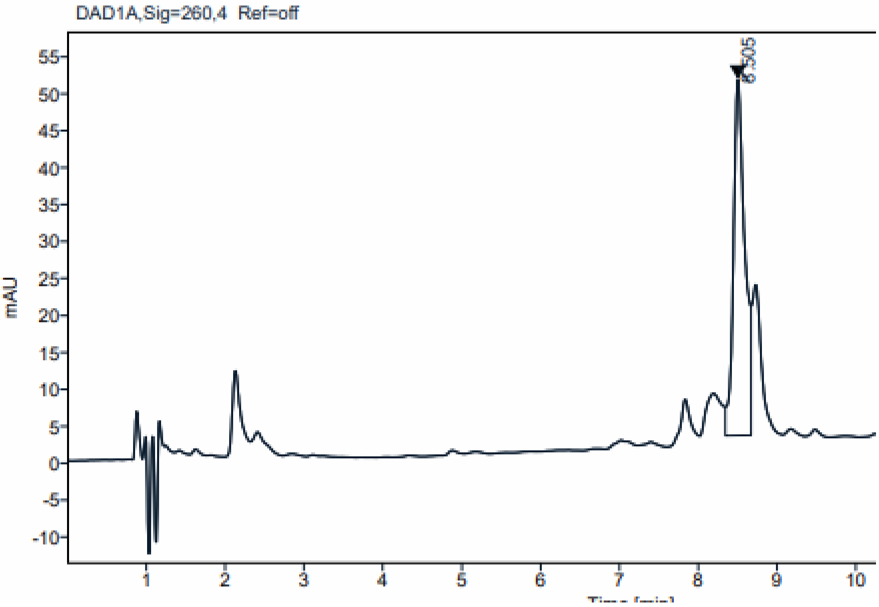

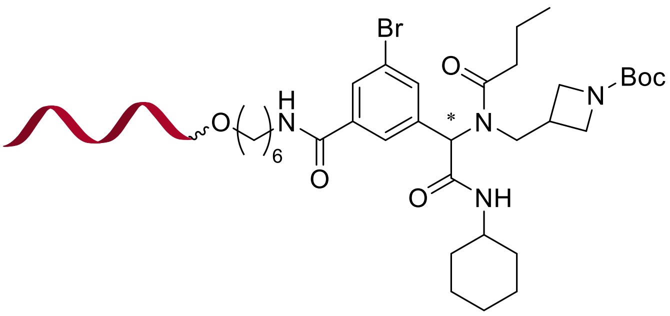

TC-5o

MS calc. 3661.0; found: 3661.9

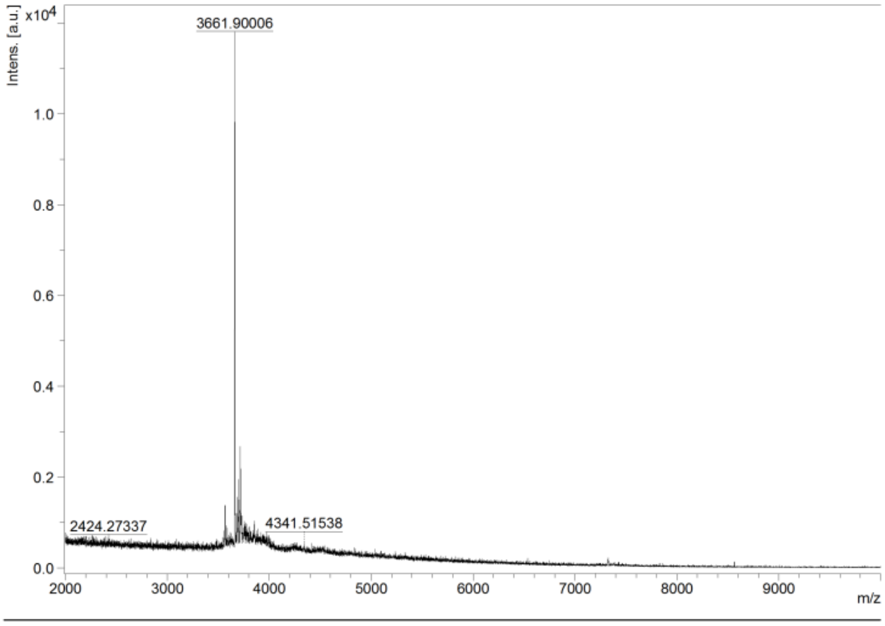

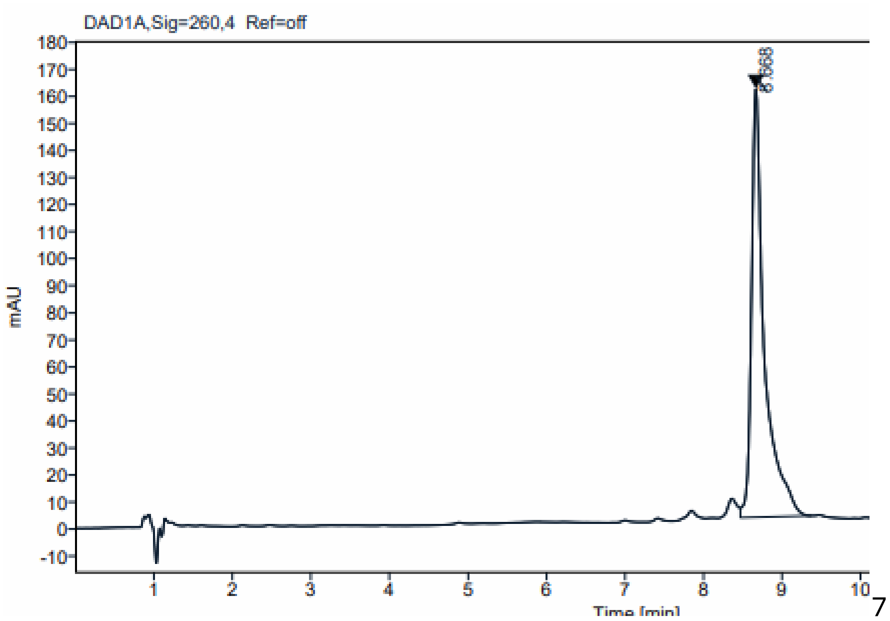

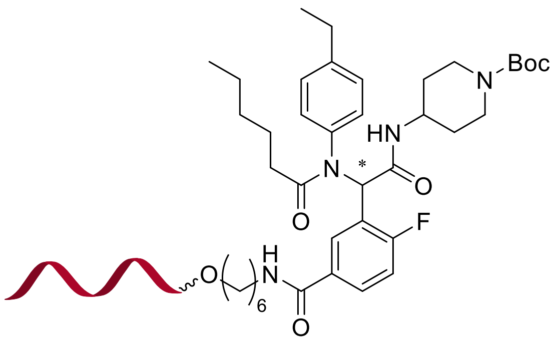

TC-5p

MS calc. 3686.0; found: 3687.0

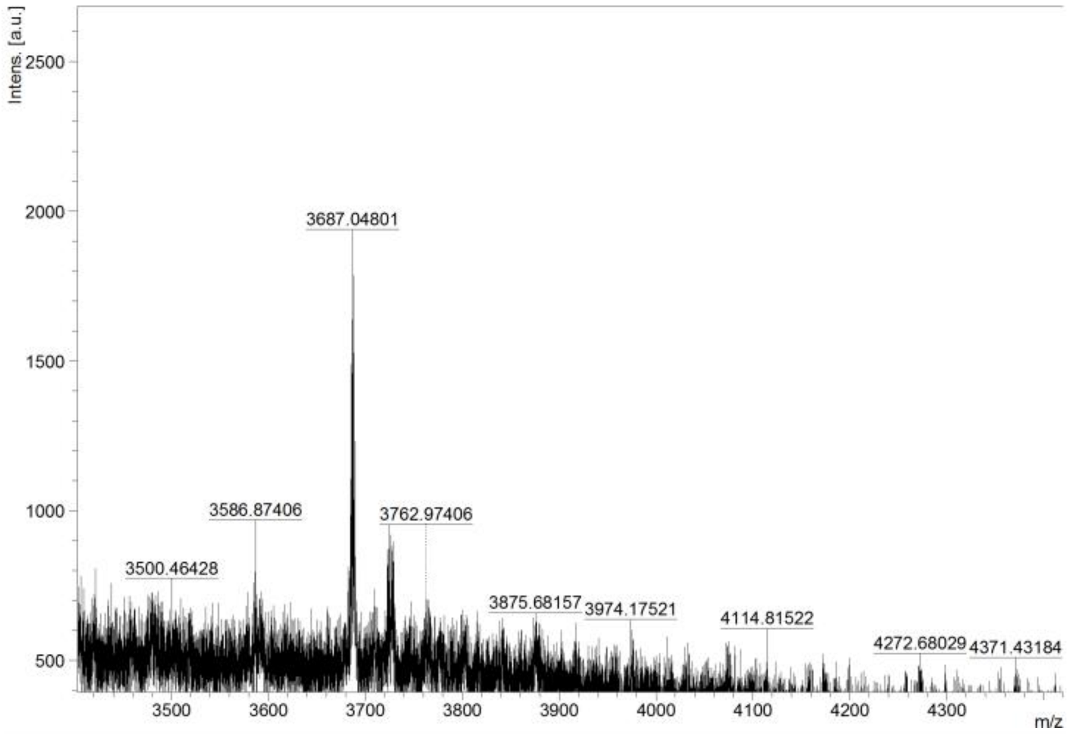

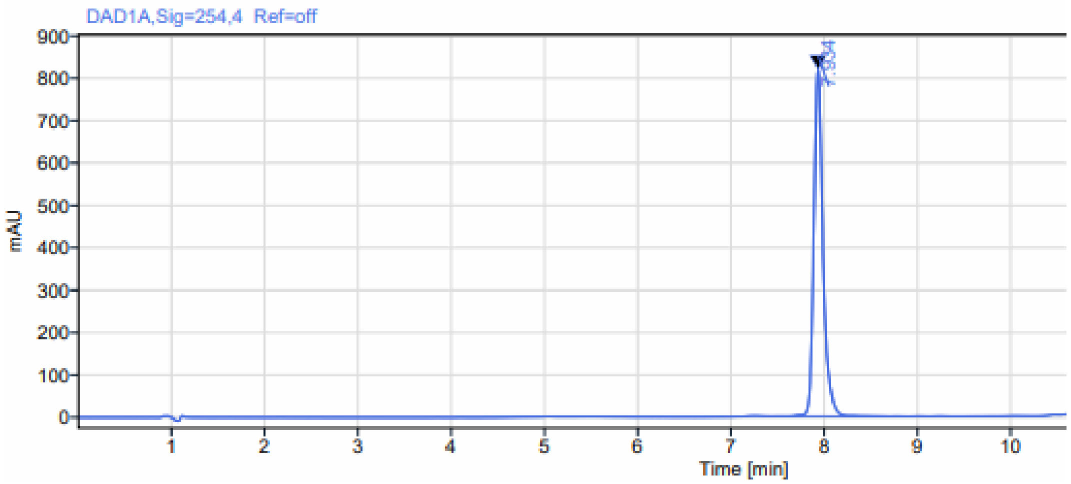

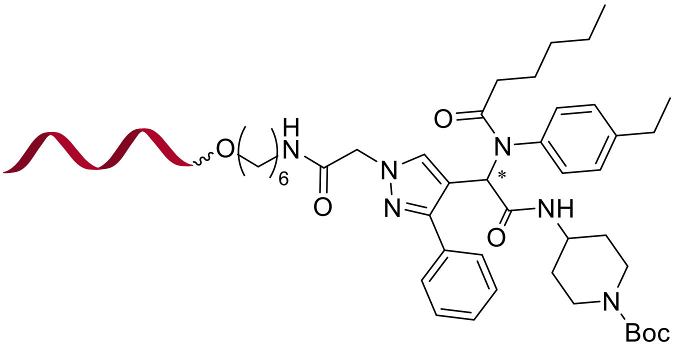

TC-5q

MS calc. 3730.0; found: 3732.2

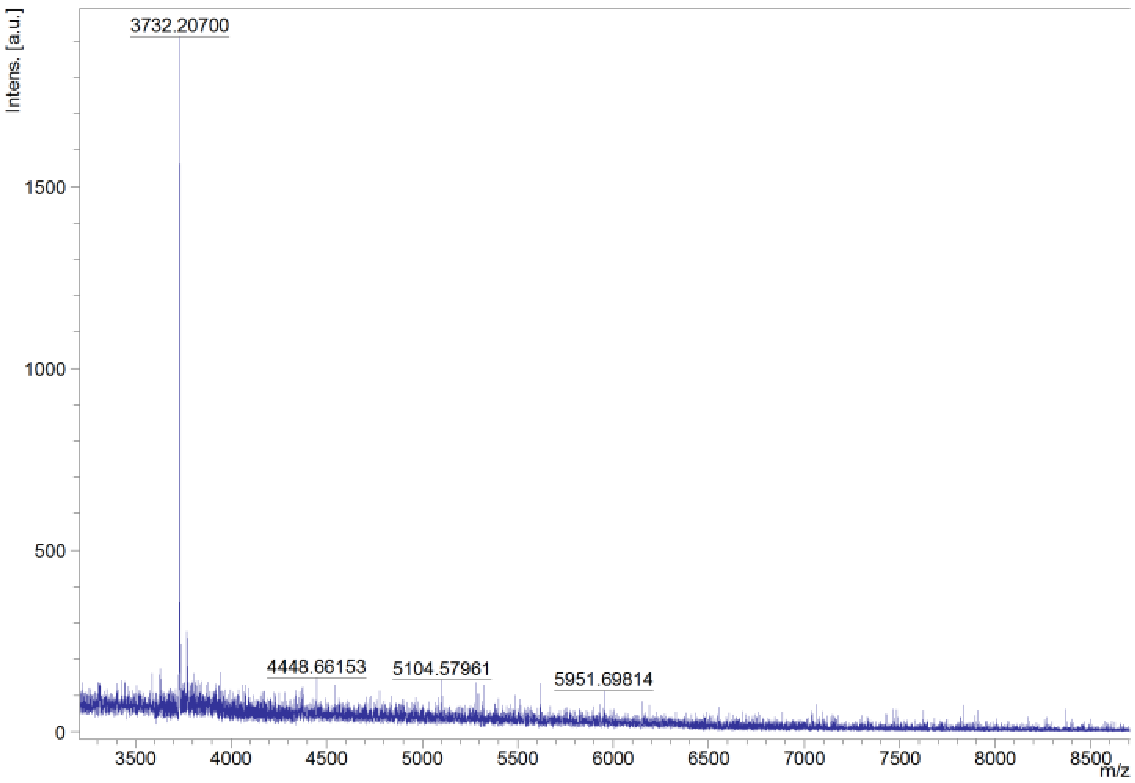

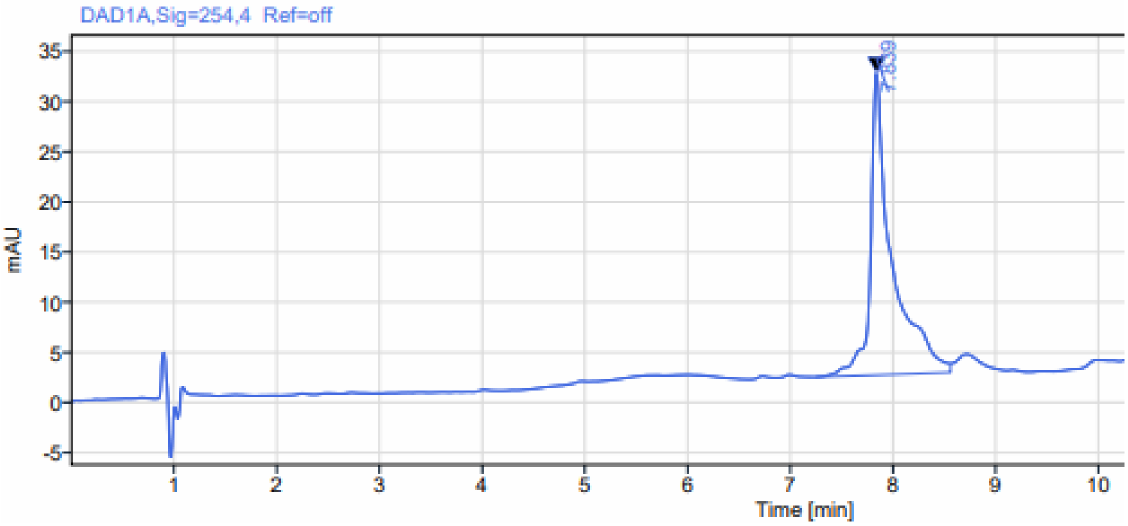

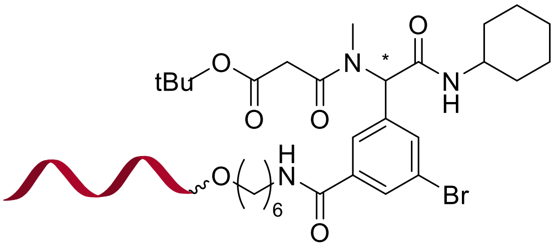

TC-5r

MS calc. 3578.0; found: 3579.9

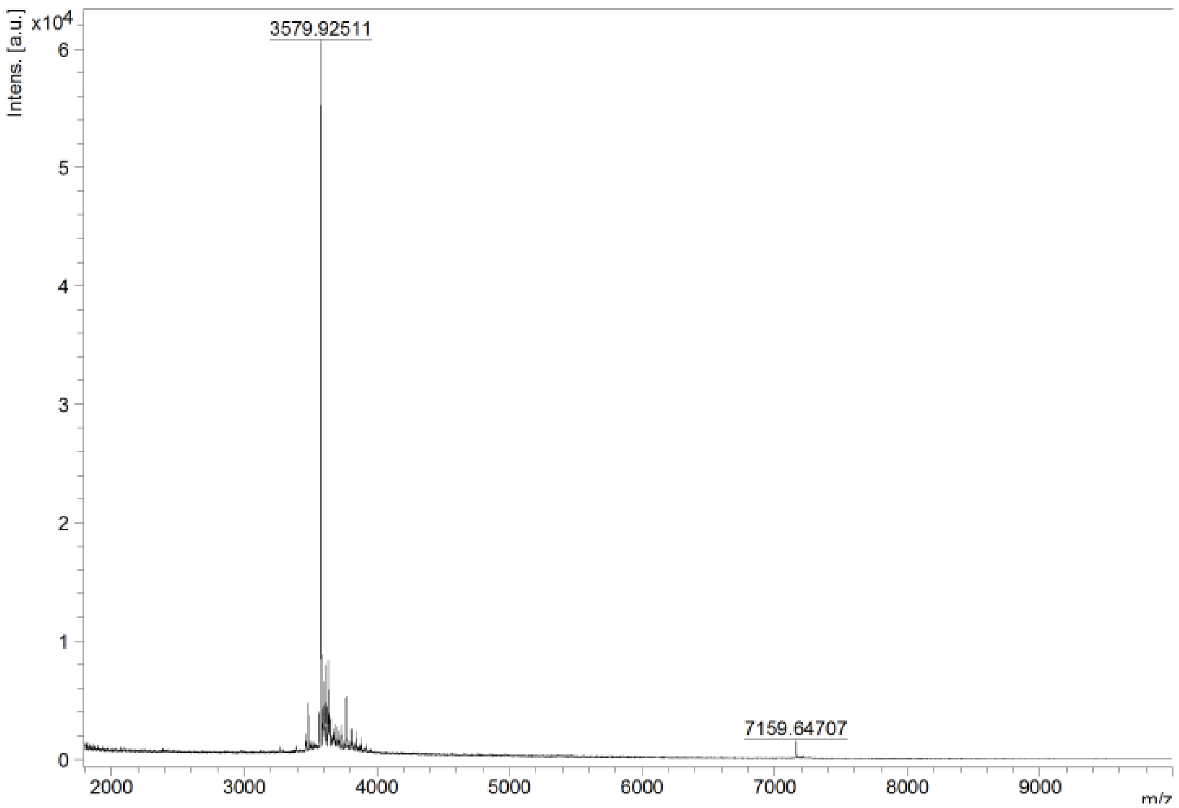

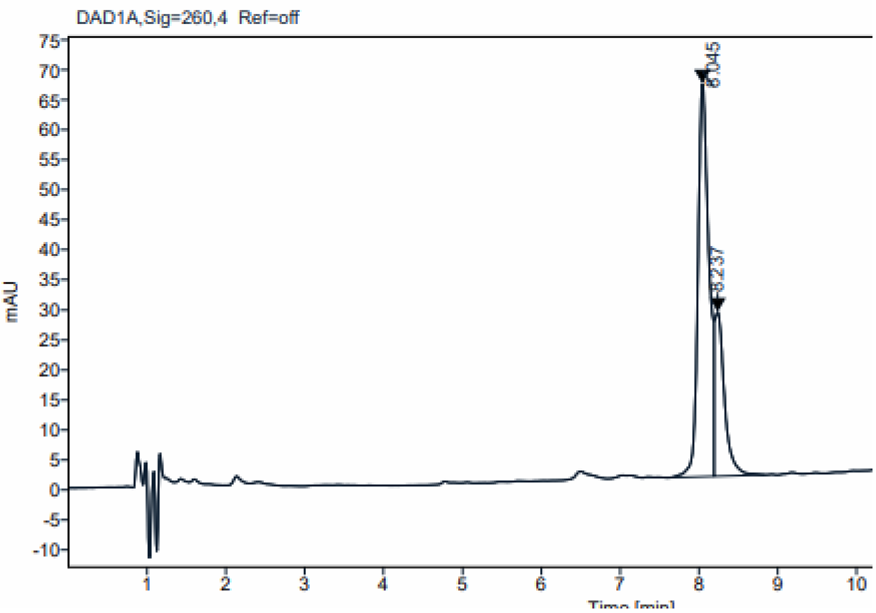

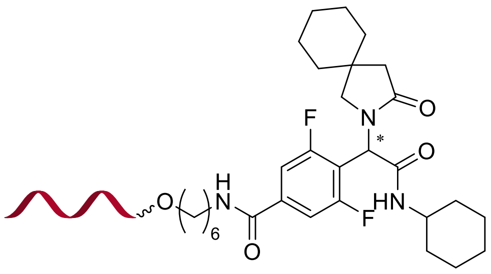

TC-5s

MS calc. 3514.0; found: 3514.5

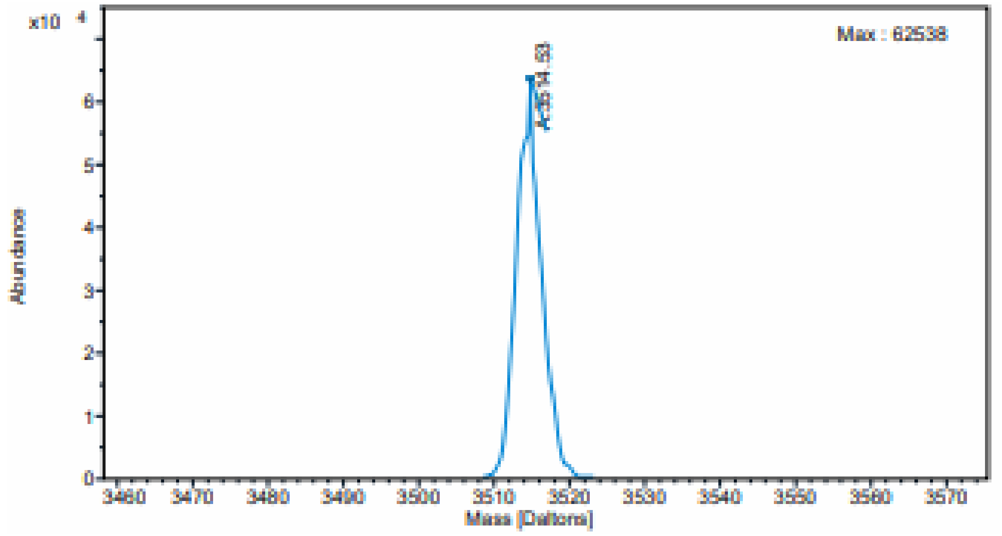

measured by LC-MS

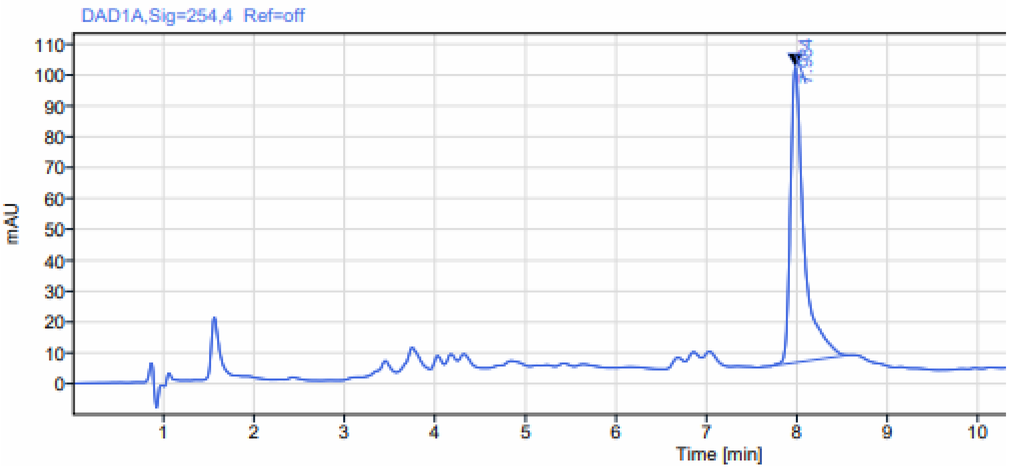

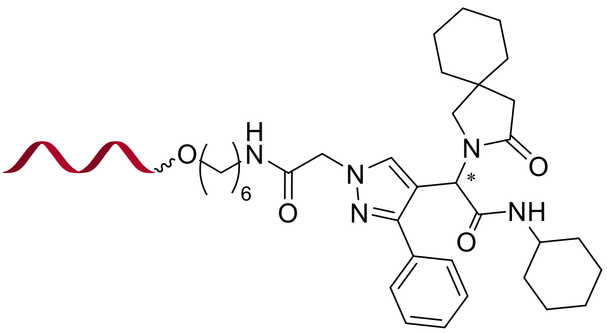

TC-5t

MS calc. 3558.0; found: 3560.0

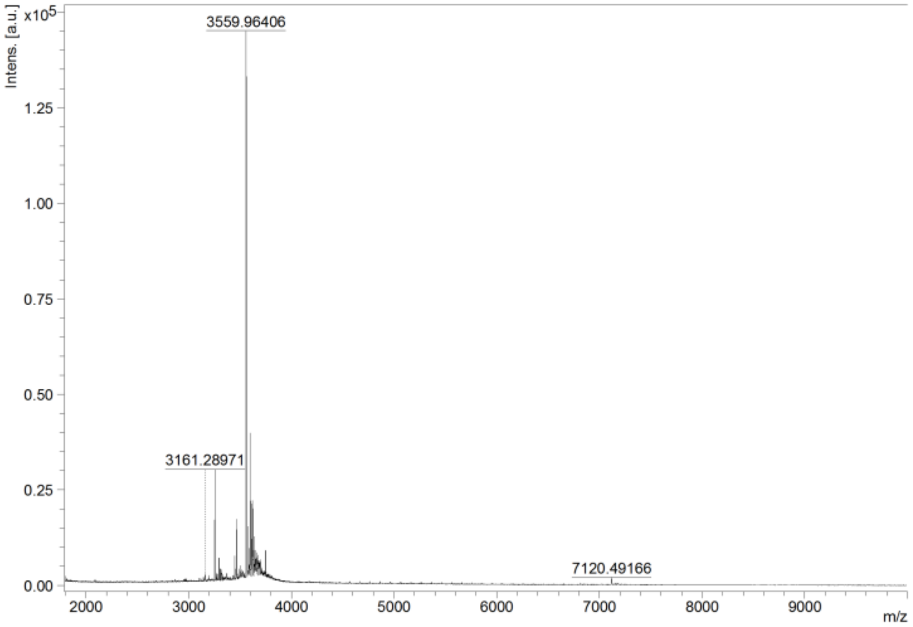

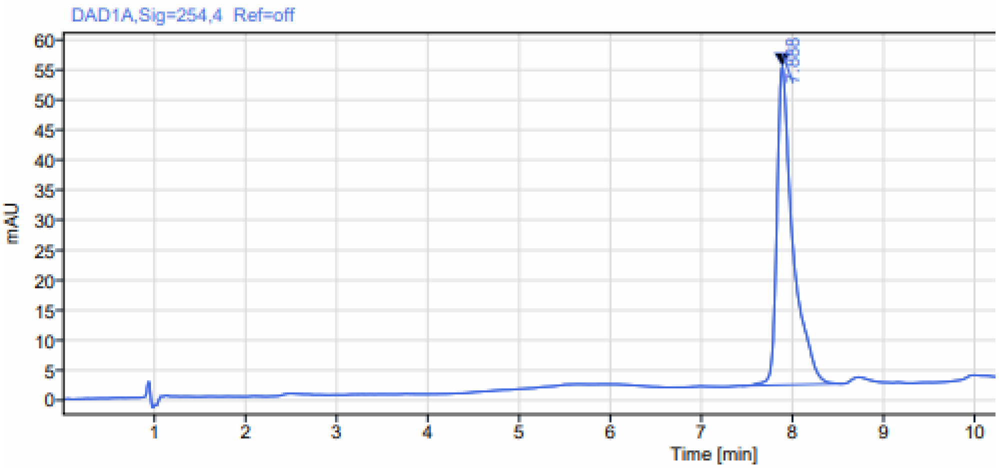

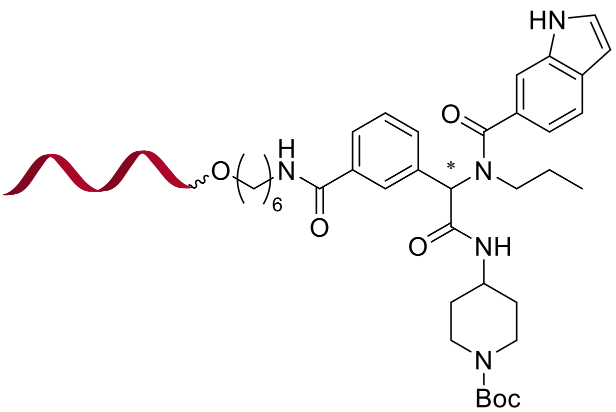

TC-5u

MS calc. 3628.0; found: 3628.7

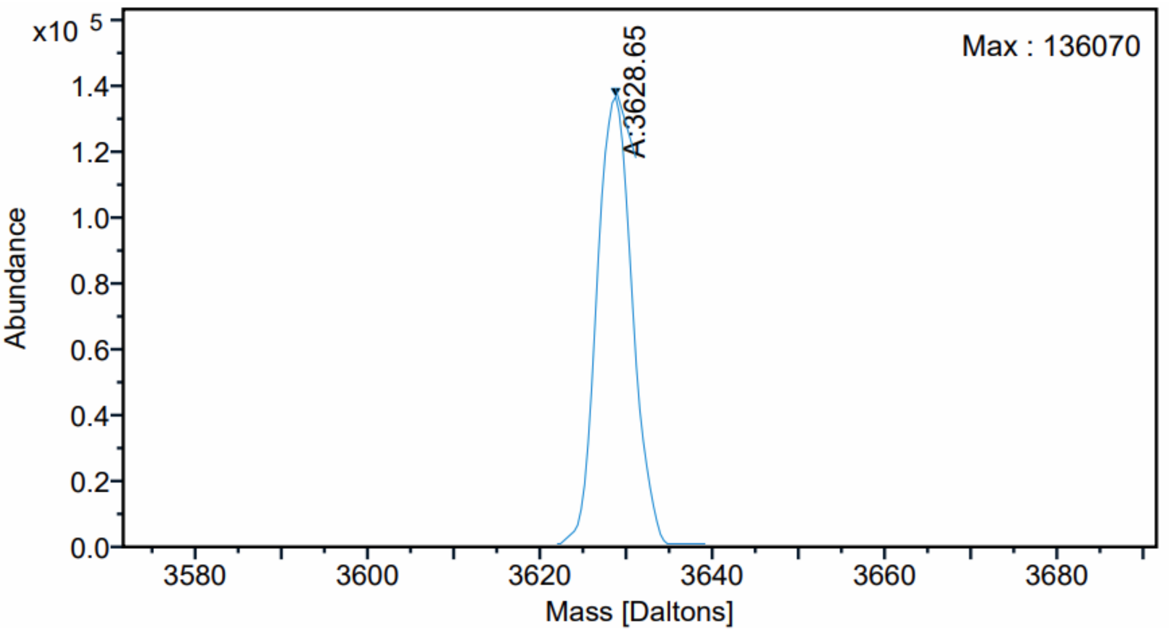

measured by LC-MS

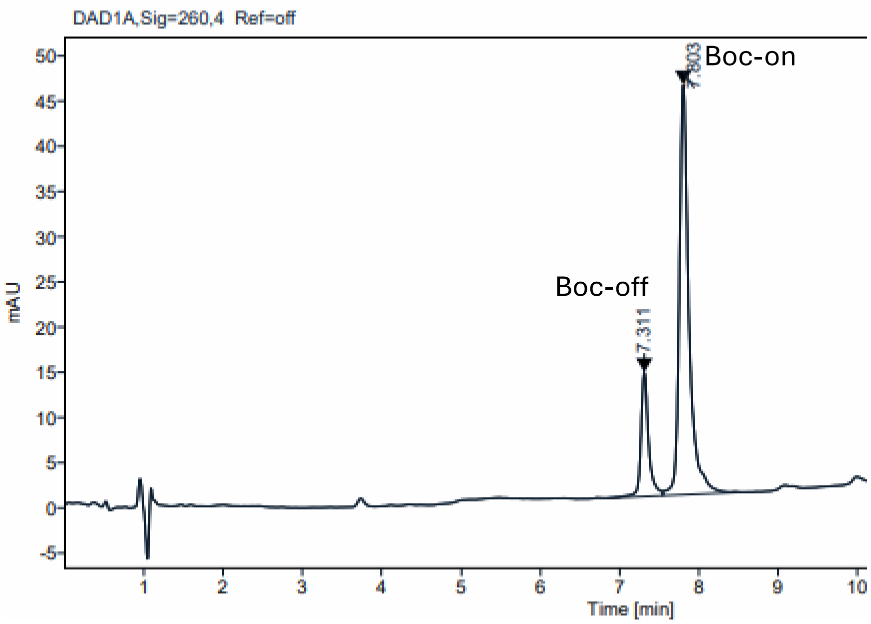

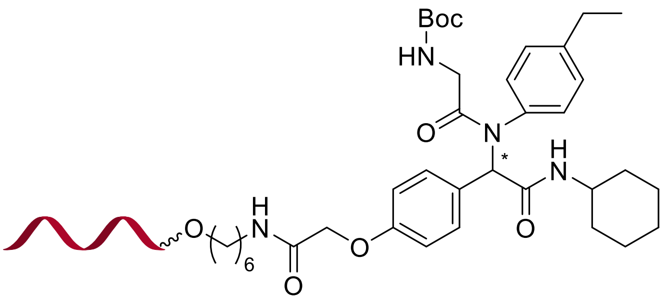

TC-5v

MS calc. 3634.0; found: 3634.4

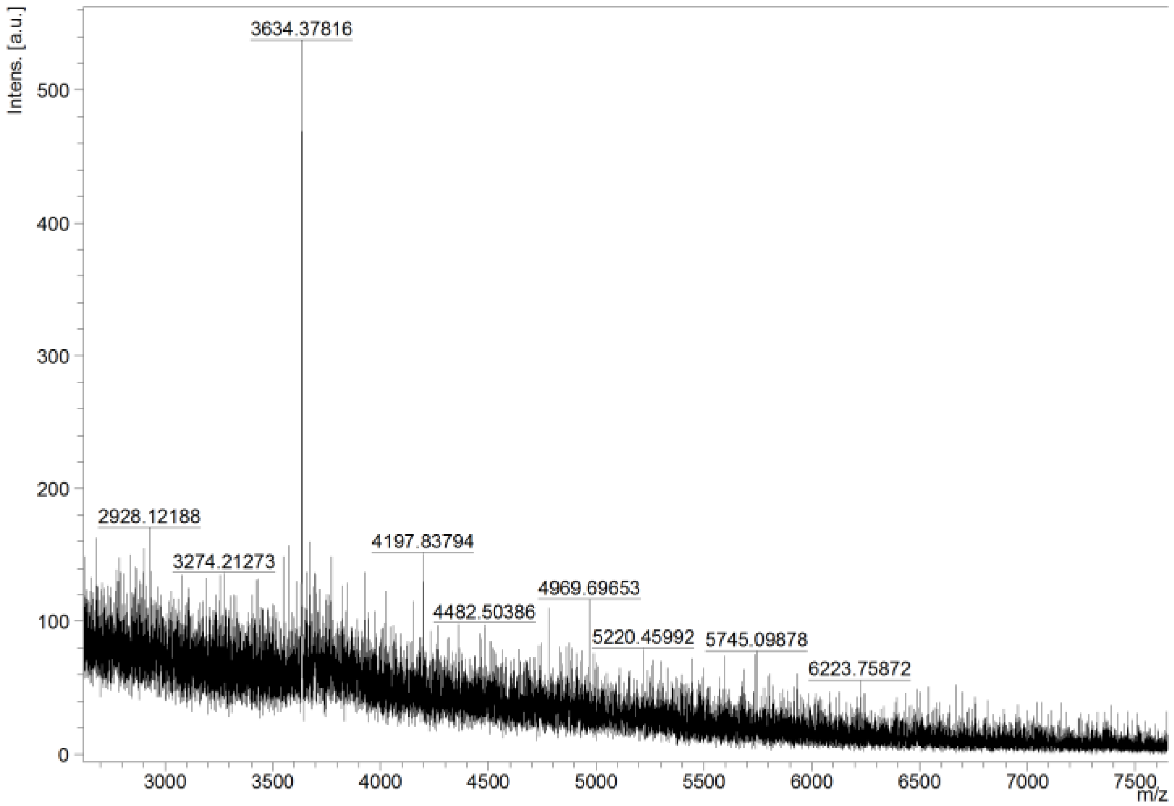

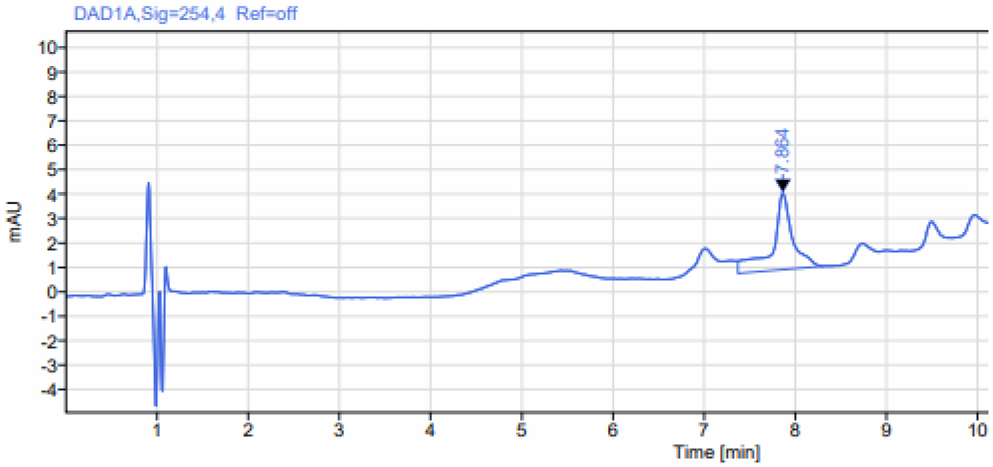

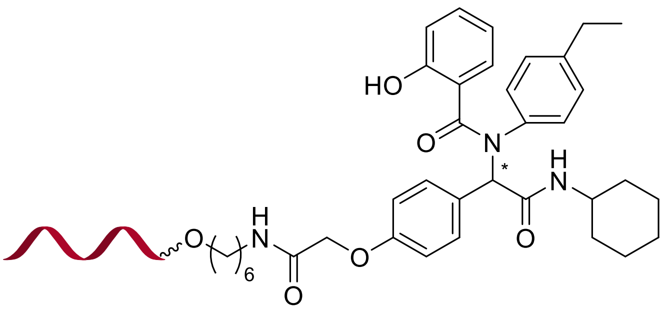

TC-5w

MS calc. 3597.0; found: 3597.4

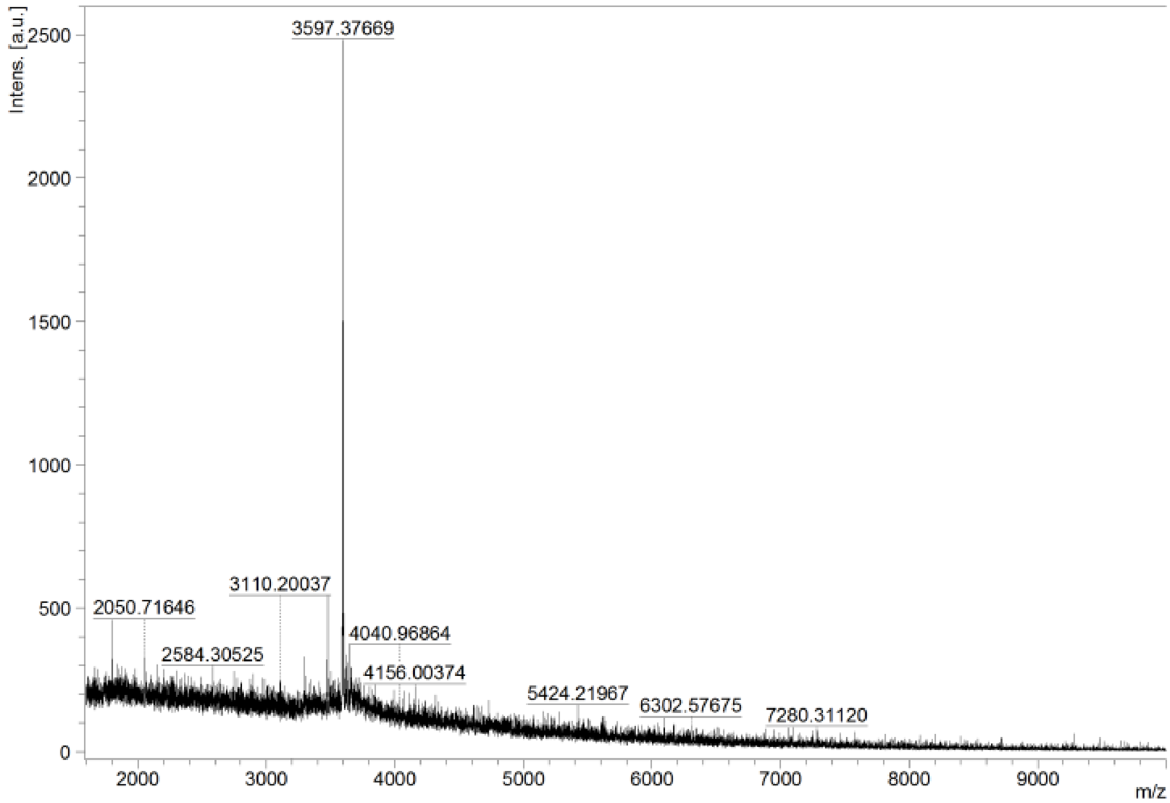

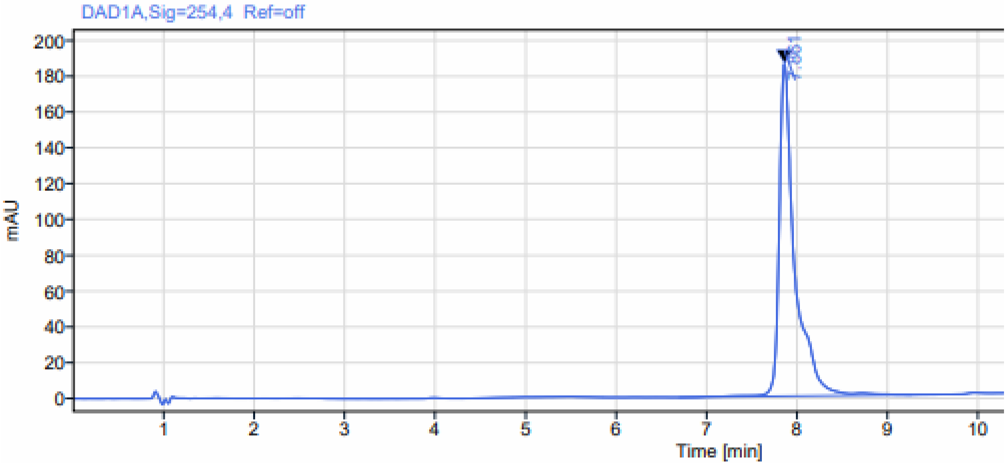

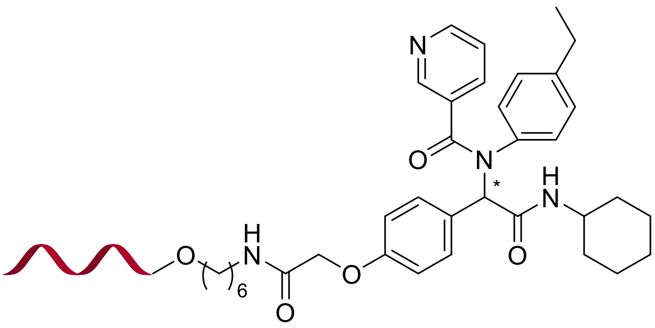

TC-5x

MS calc. 3582.0; found: 3581.7

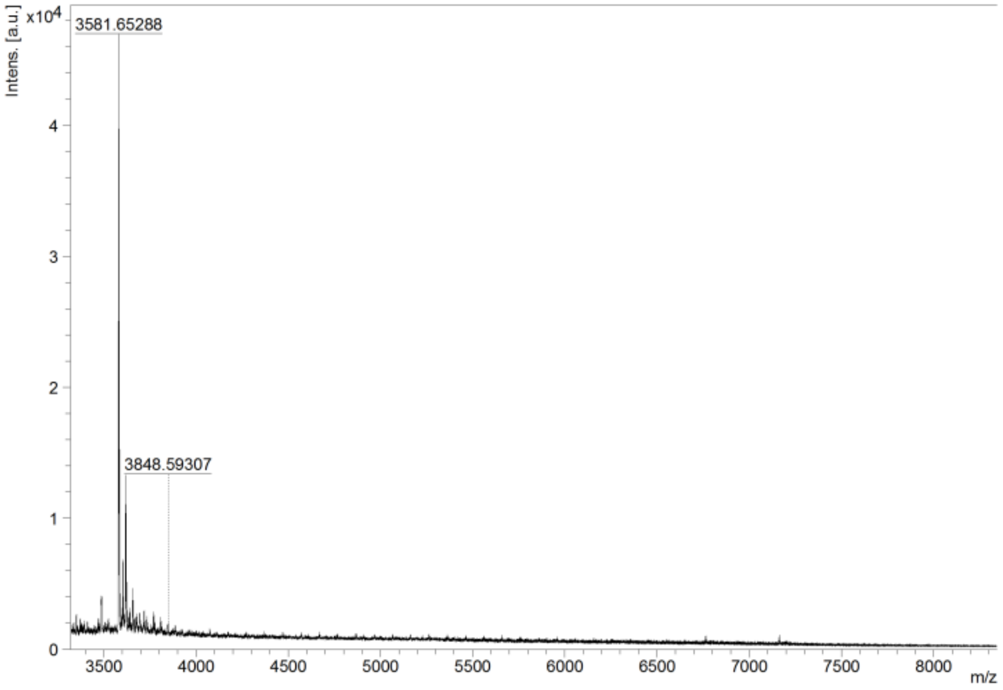

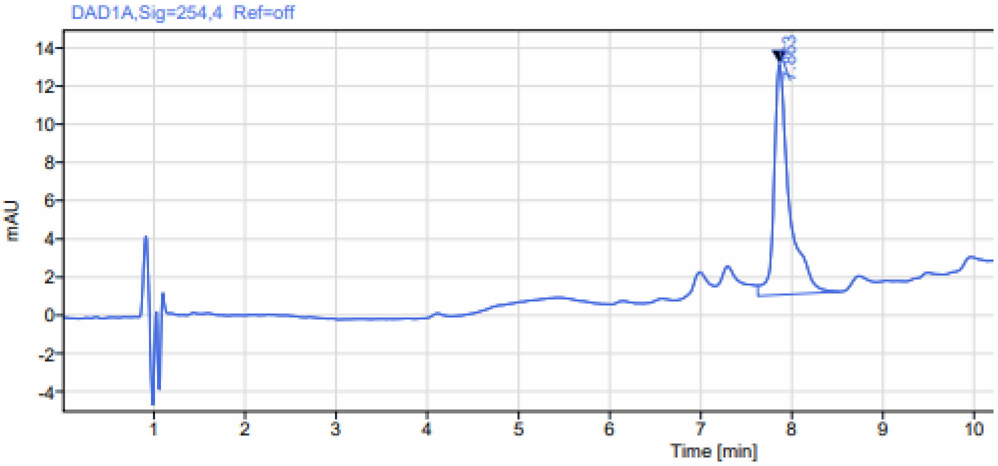

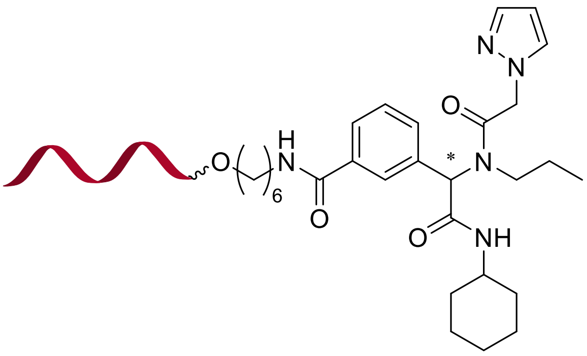

TC-5y

MS calc. 3594.0; found: 3593.6

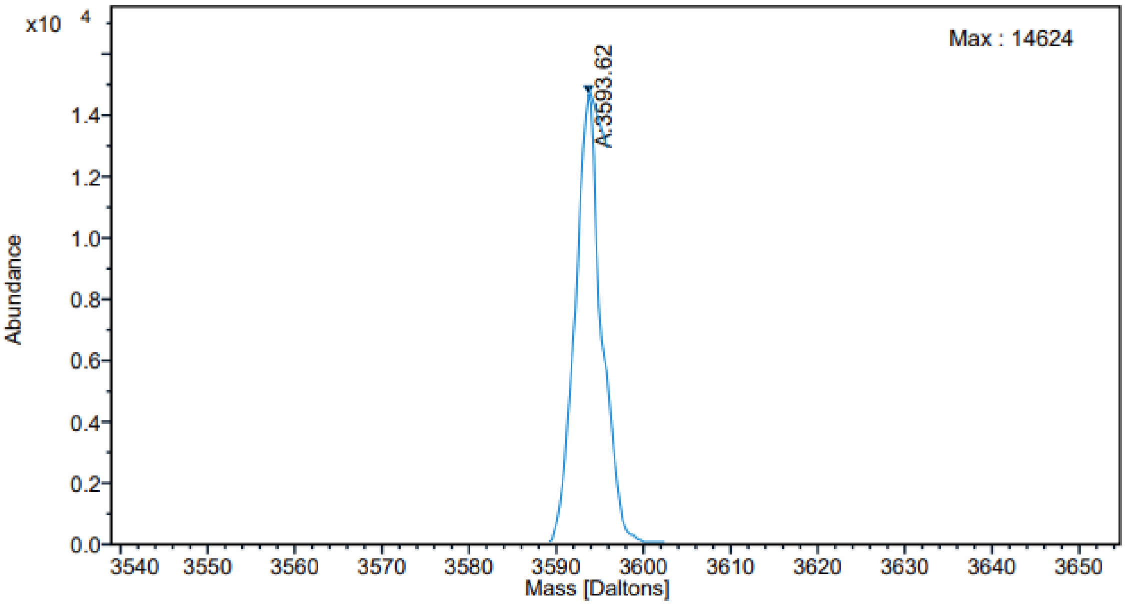

measured by LC-MS

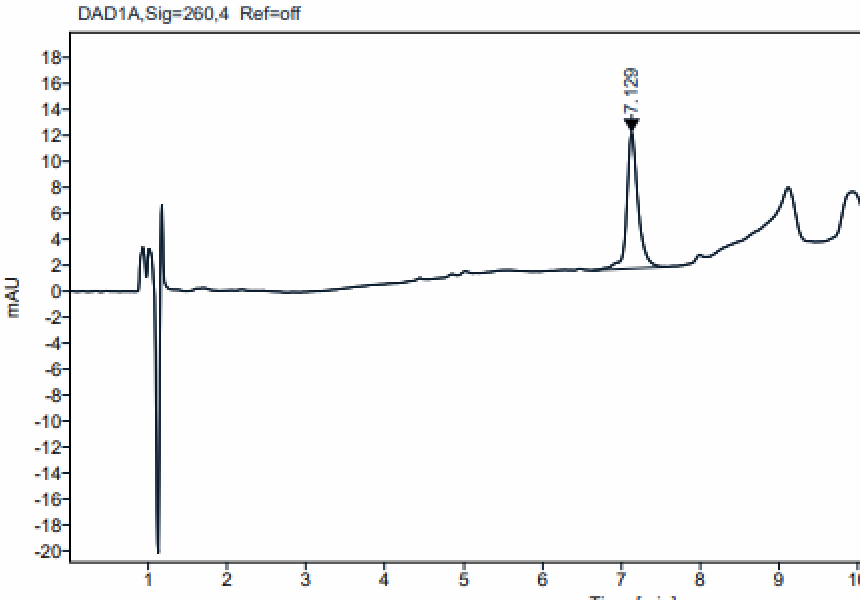

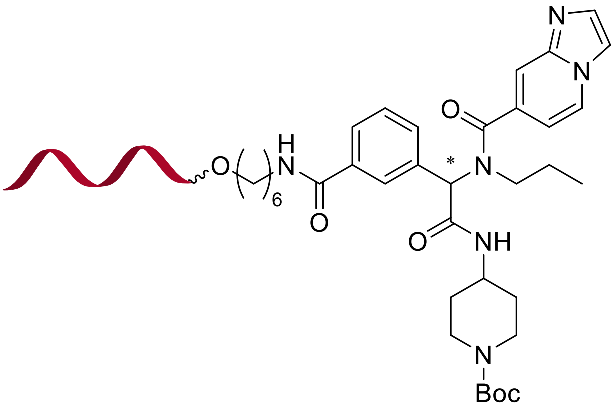

TC-5z

MS calc. 3630.0; found: 3630.3

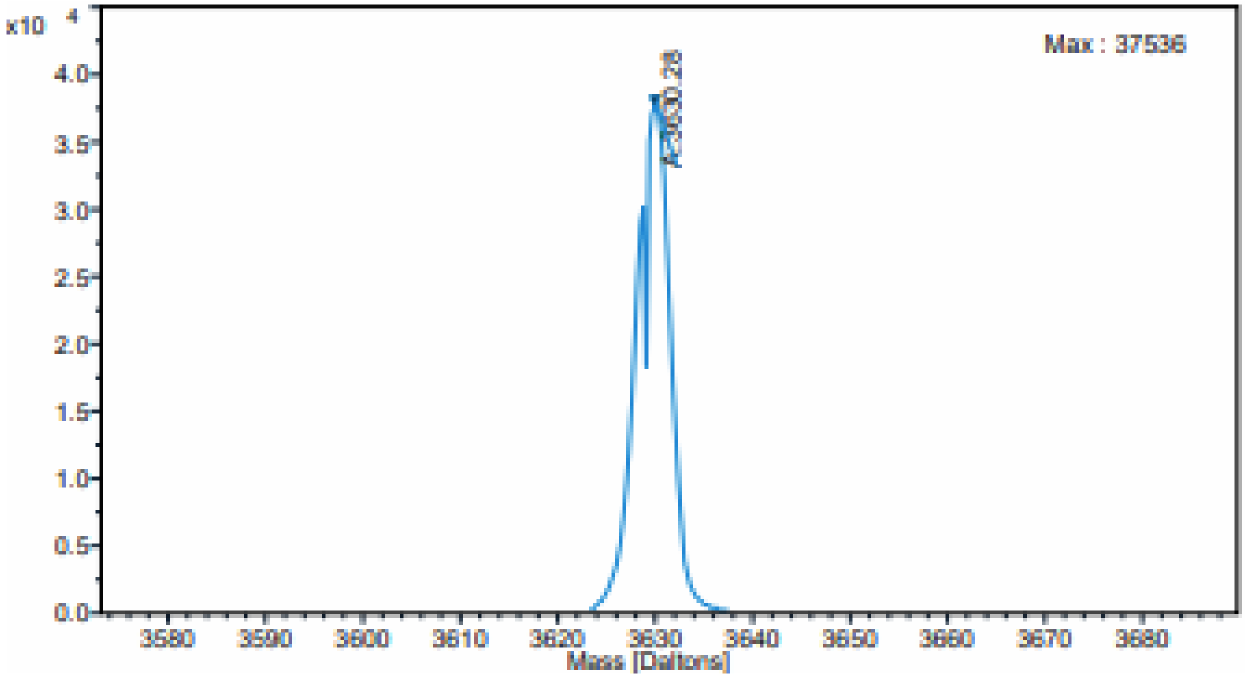

measured by LC-MS

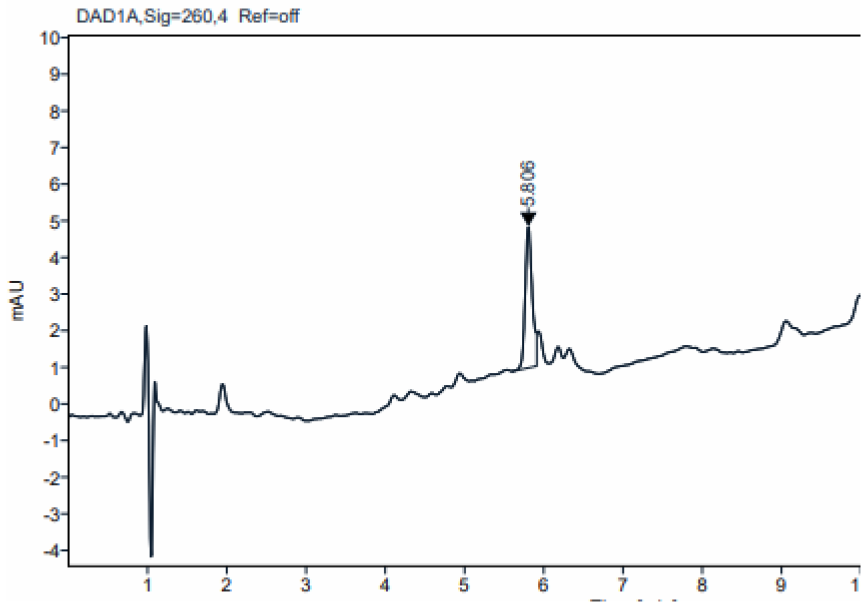

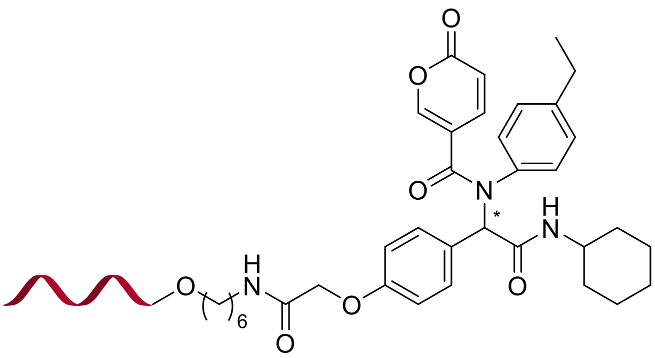

TC-5aa

MS calc. 3598.0; found: 3632.3 [M+2*NH_4^+]

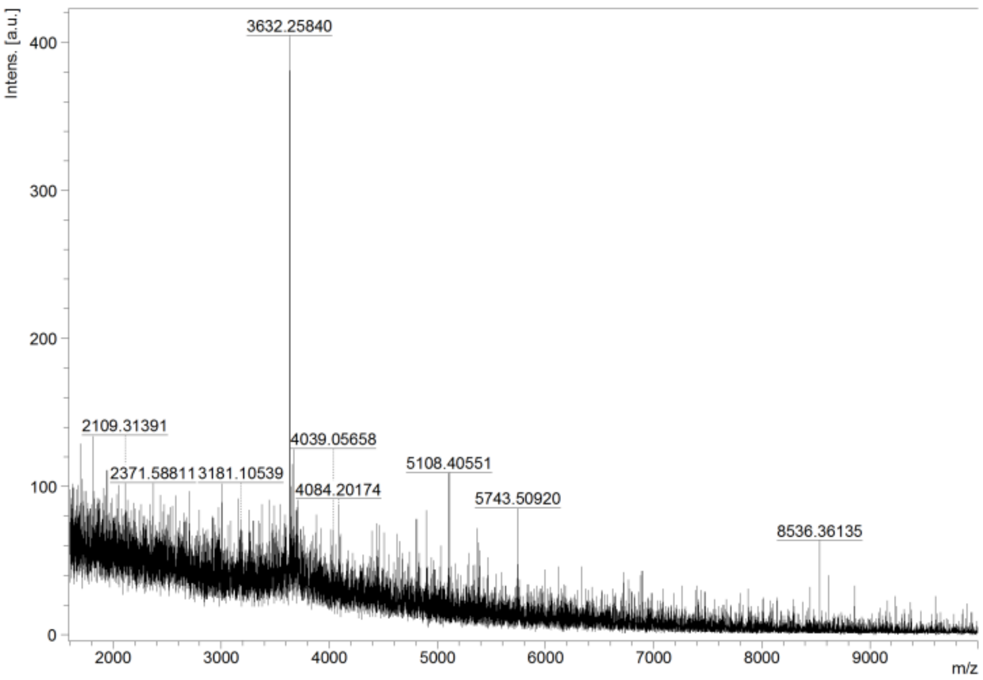

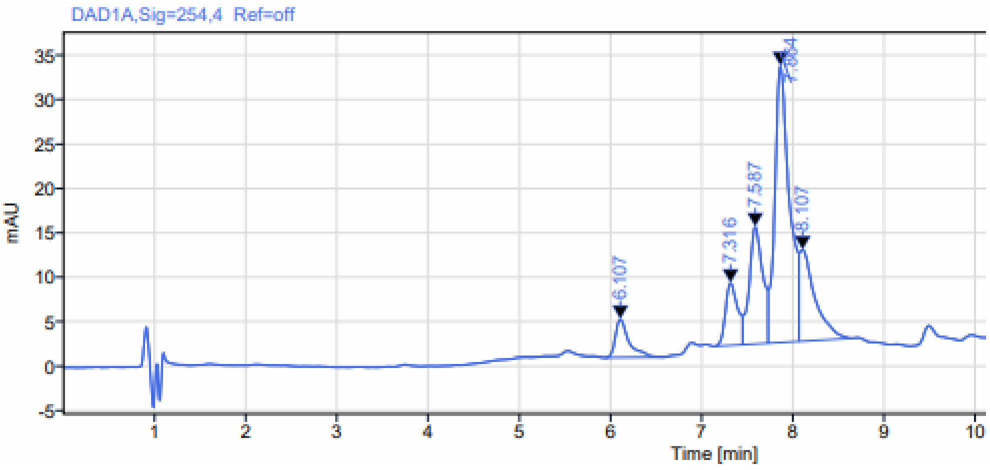

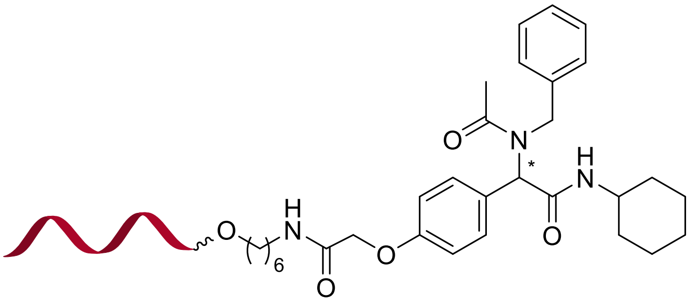

TC-5ab

MS calc. 3504.0; found: 3504.4

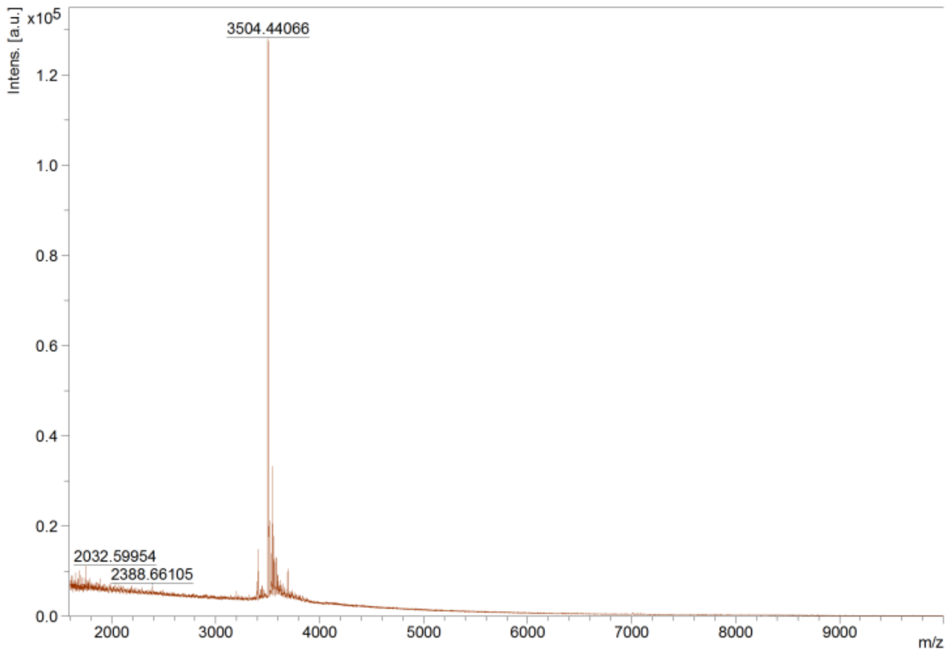

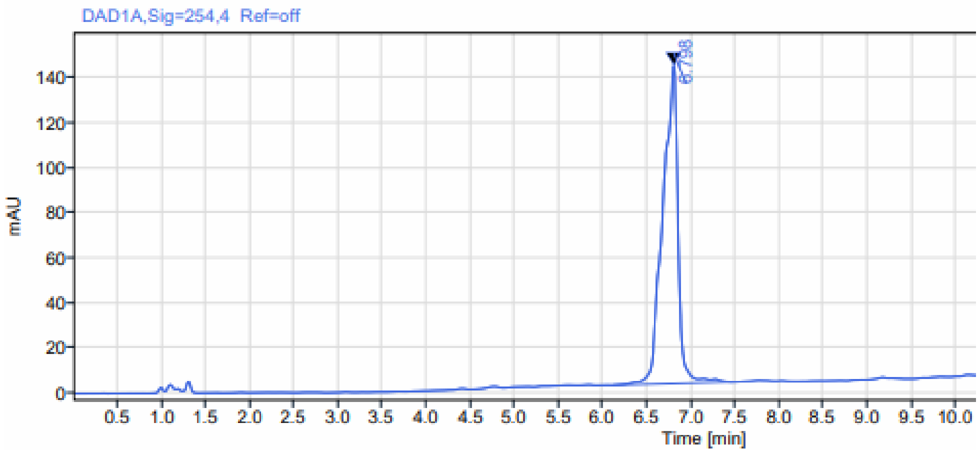

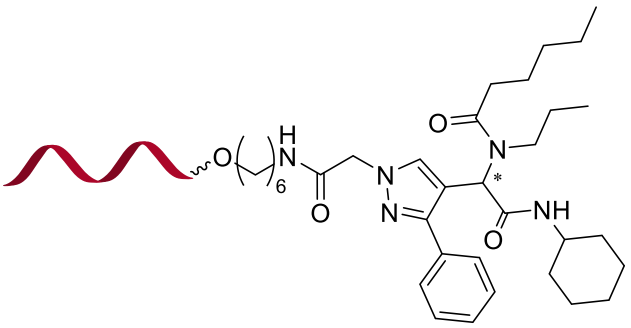

TC - 5ac

MS calc. 3562.0; found: 3563.9

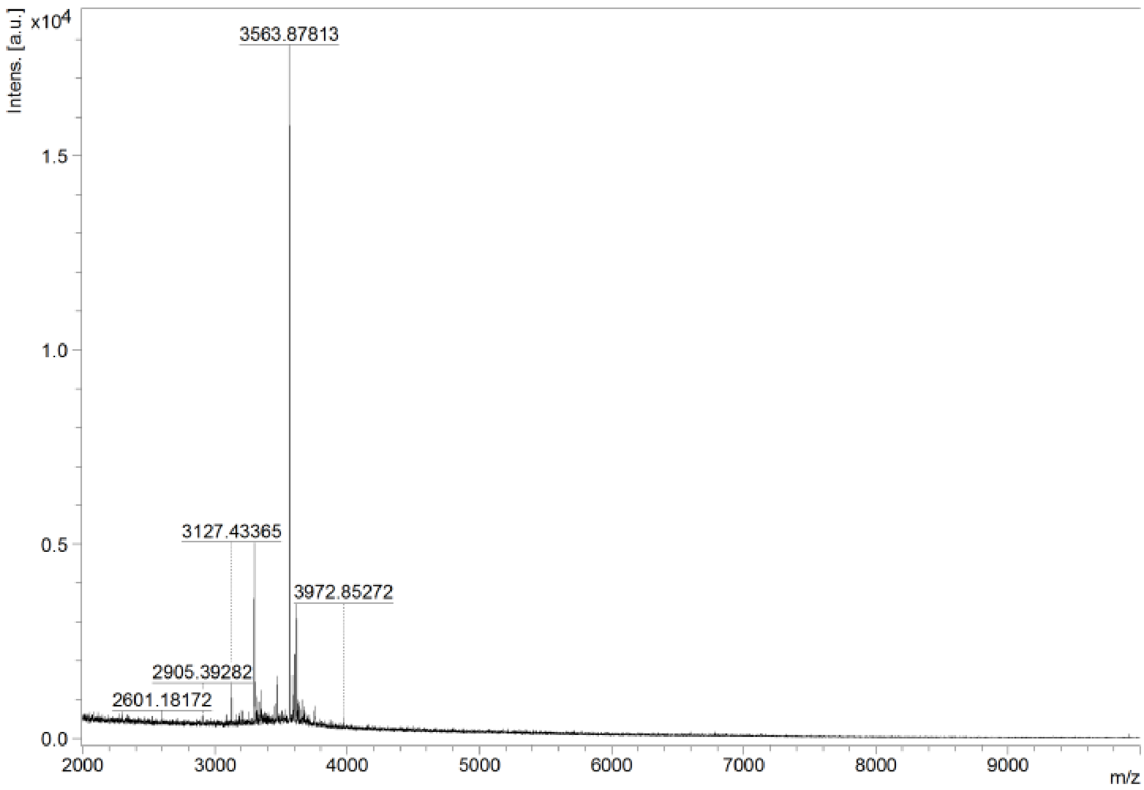

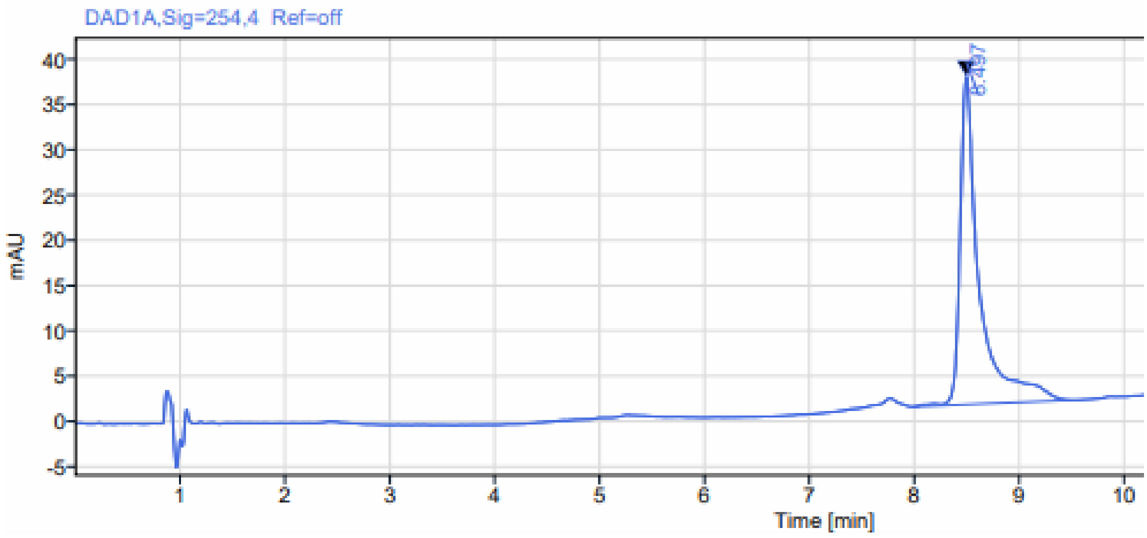

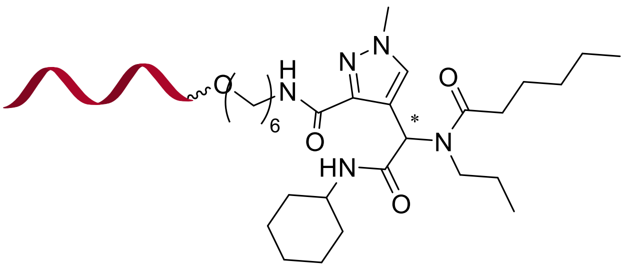

TC- 5ad

MS calc. 3486.0; found: 3487.7

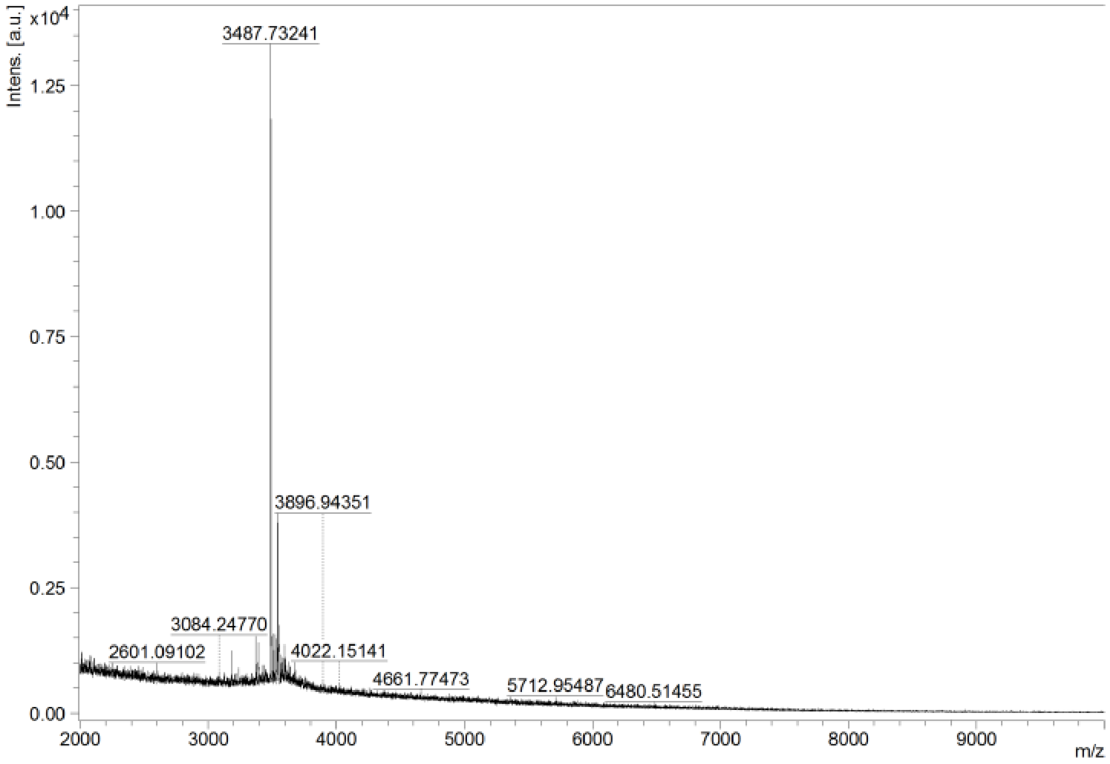

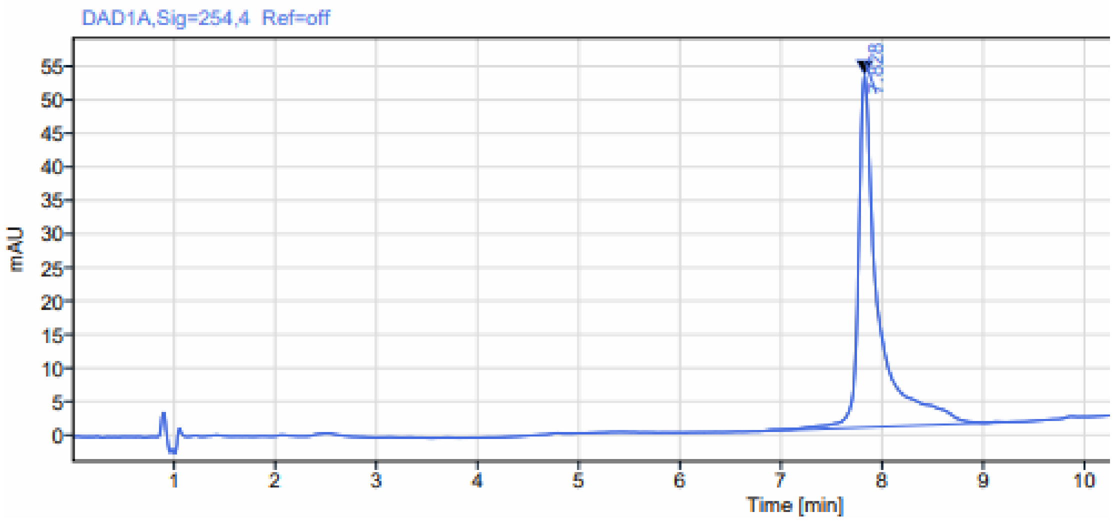

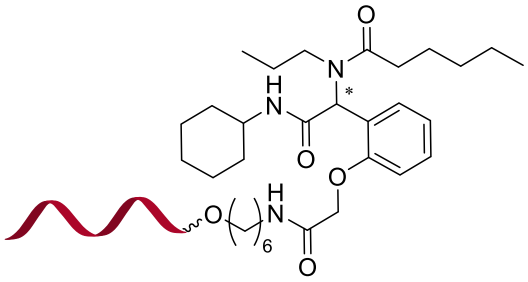

TC- 5ae

MS calc. 3512.0; found: 3513.7

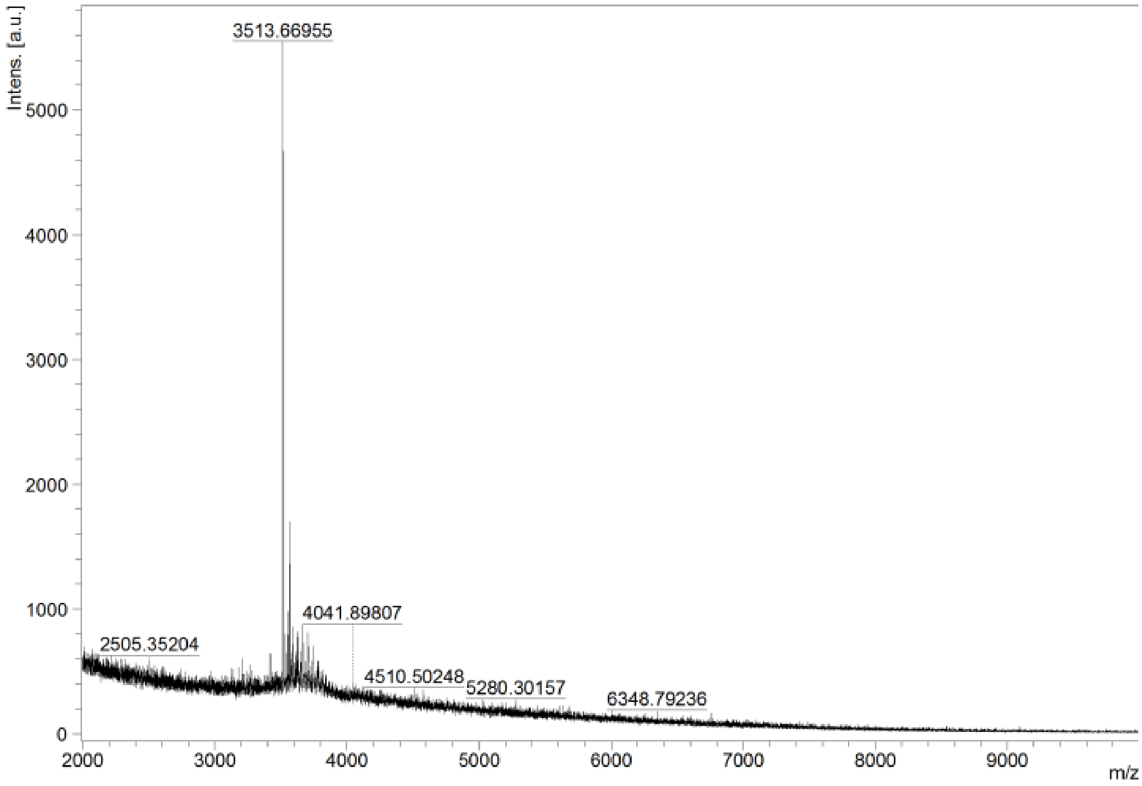

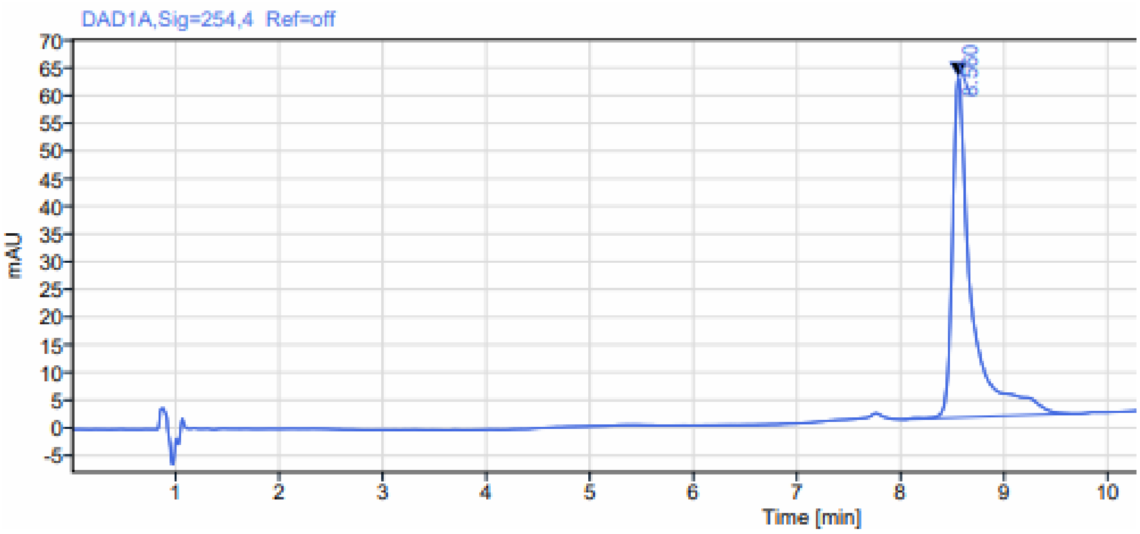

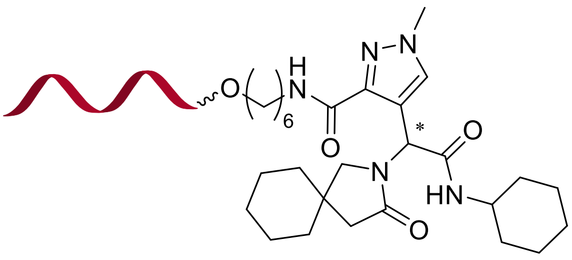

TC- 5af

MS calc. 3482.0; found: 3484.0

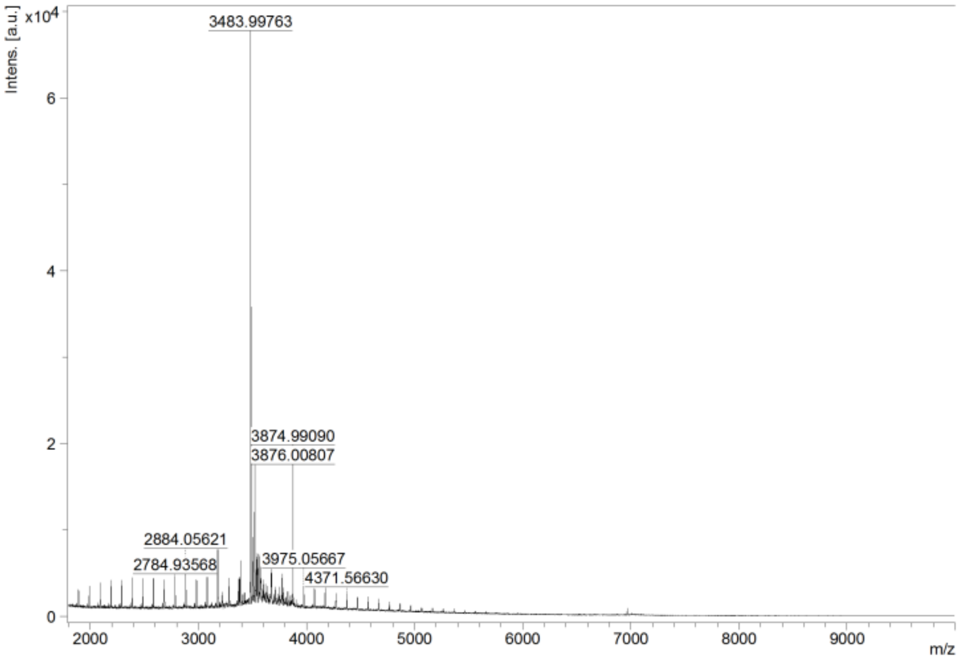

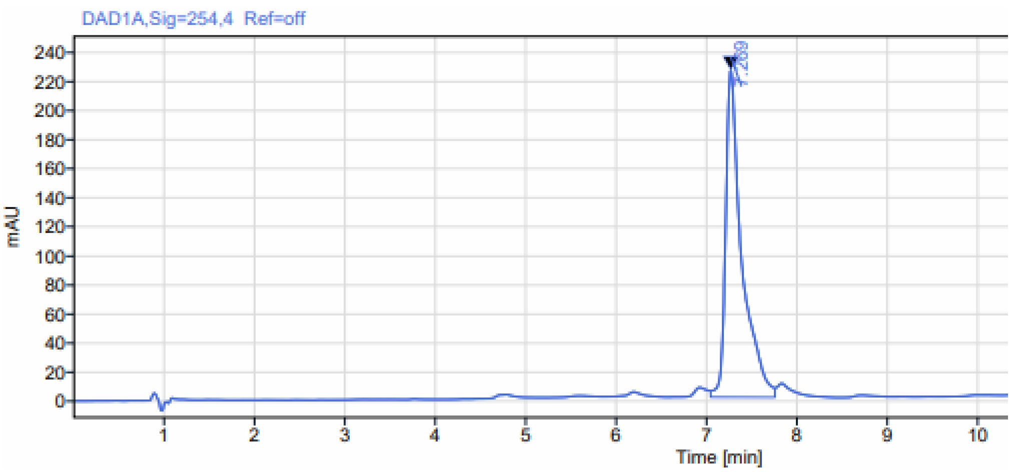

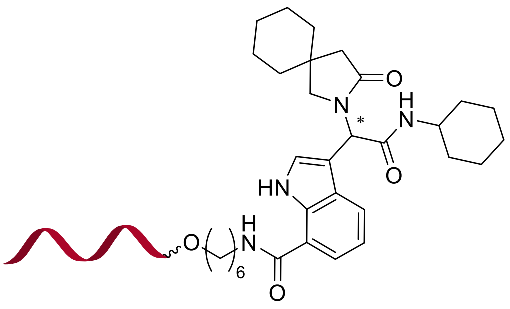

TC- 5ag

MS calc. 3517.0; found: 3518.6

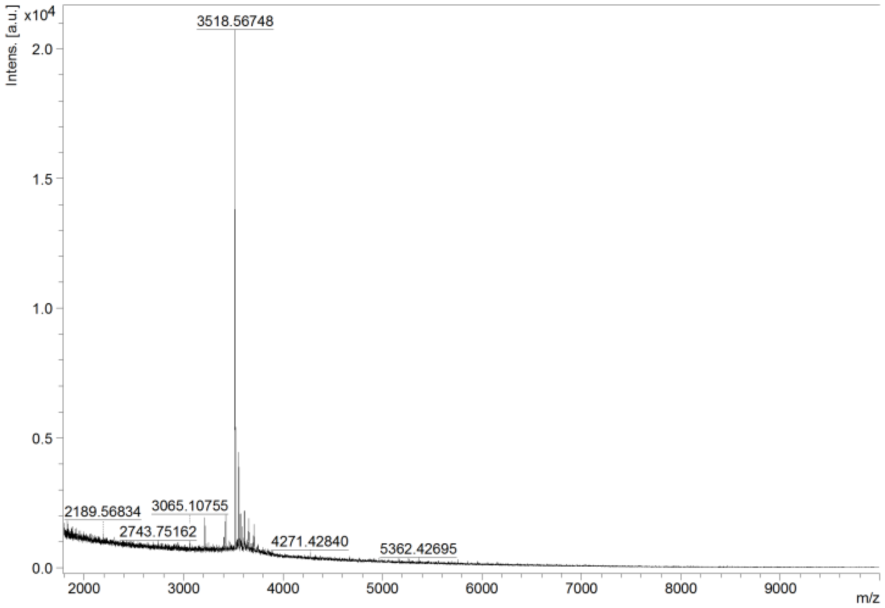

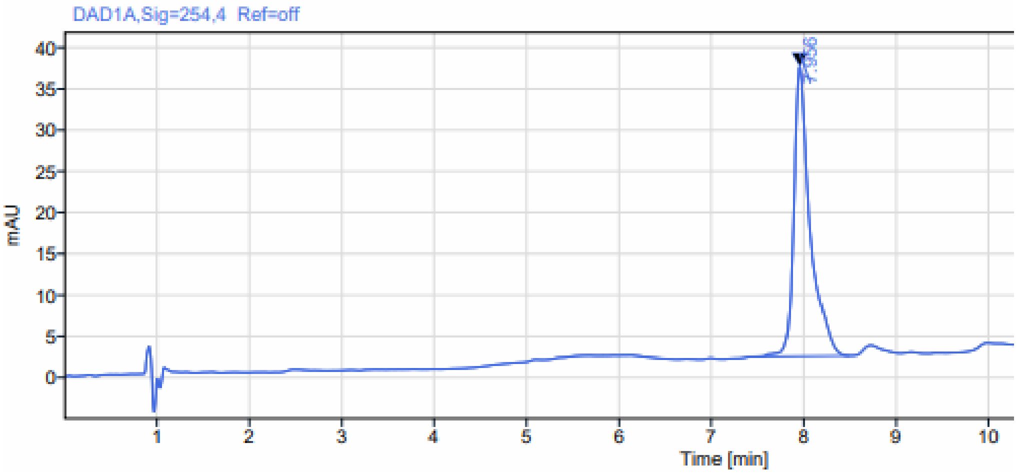

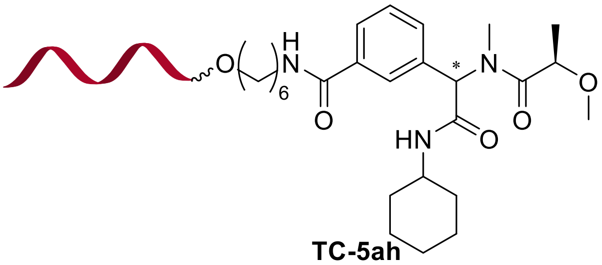

TC-5ah

MS calc. 3443.0; found: 3444.0

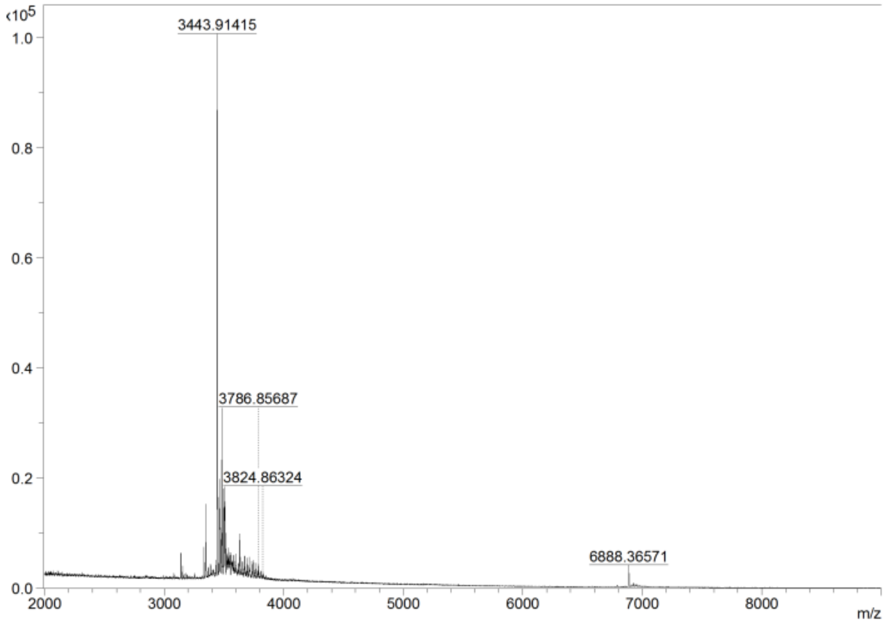

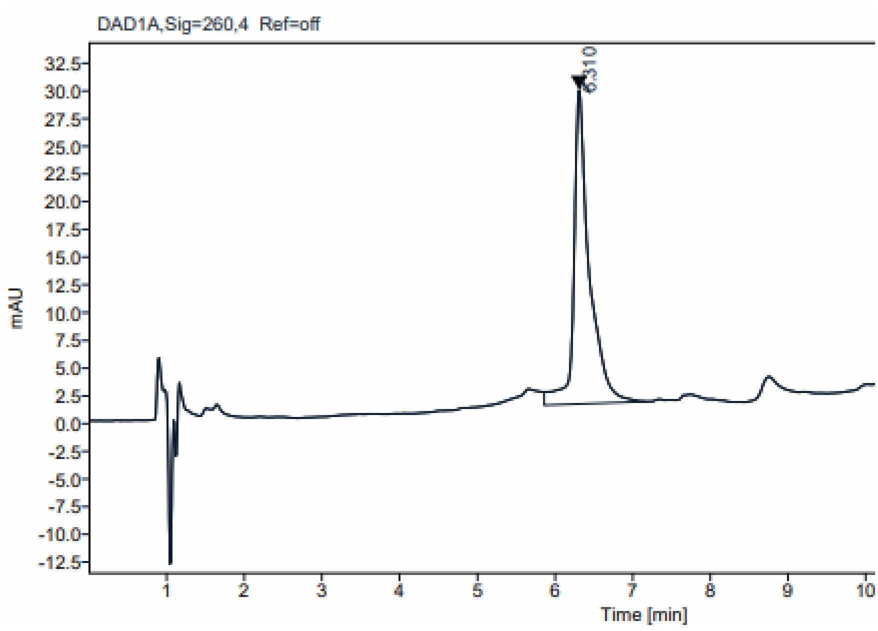

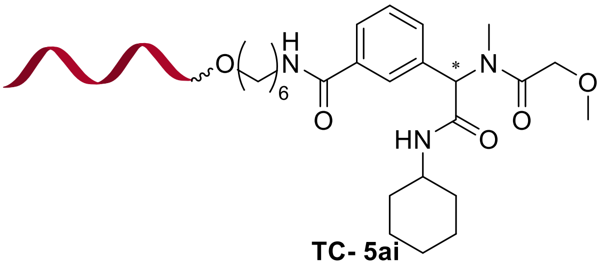

TC- 5ai

MS calc. 3429.0; found: 3429.9

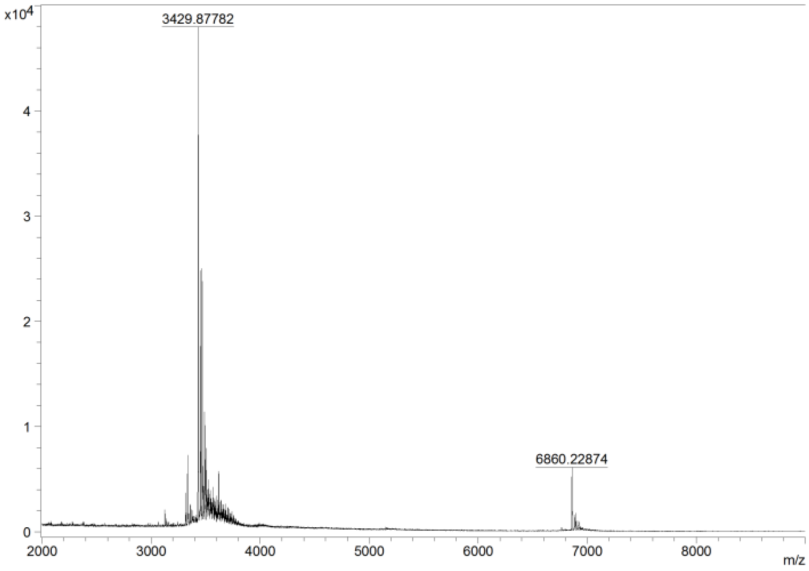

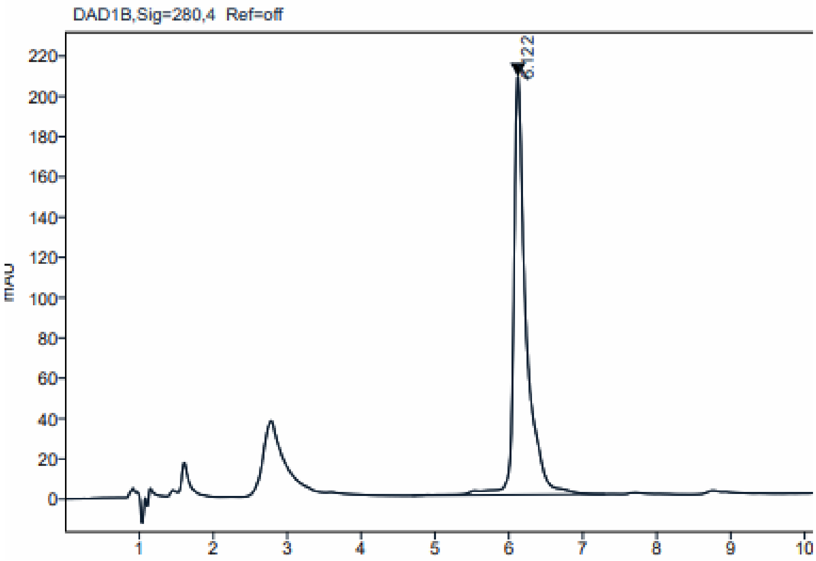

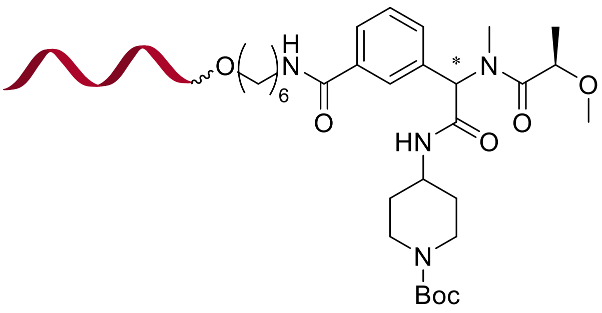

TC-5aj

MS calc. 3544.0; found: 3545.0

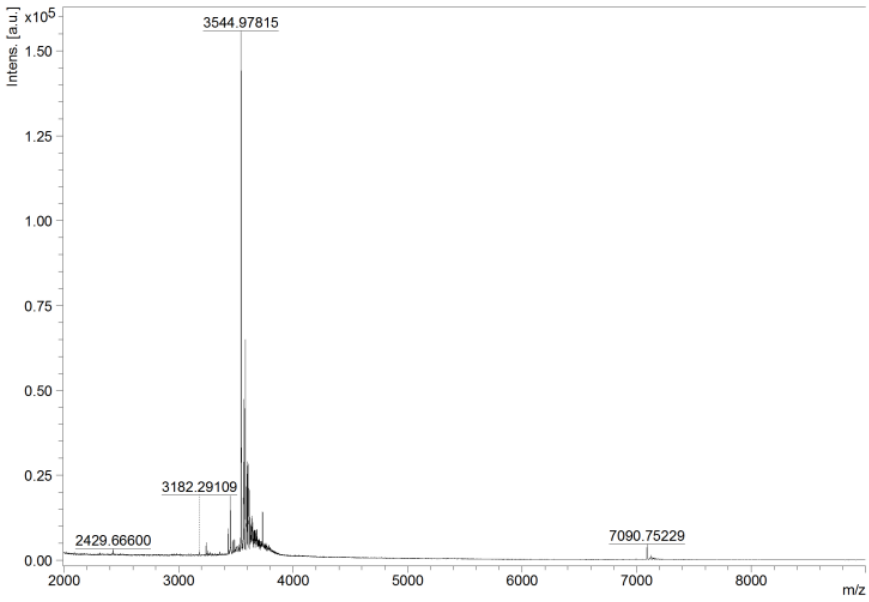

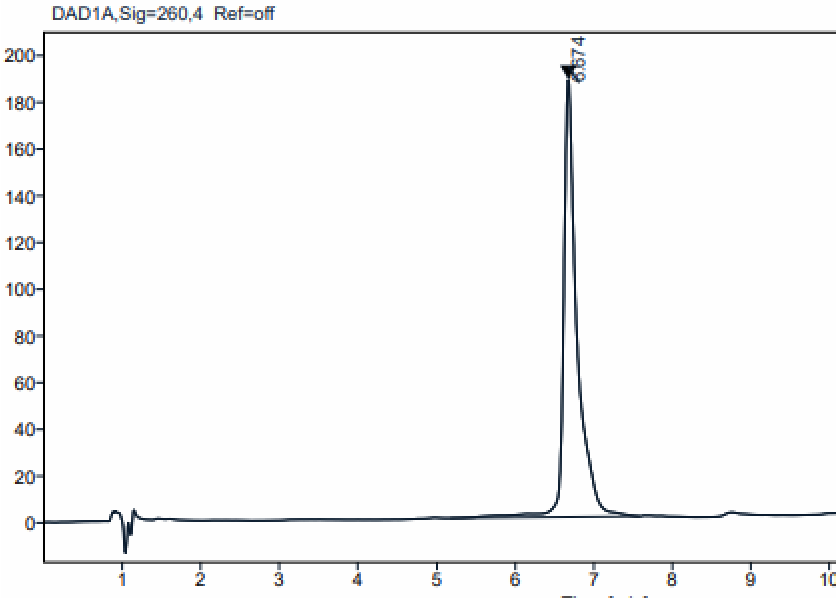

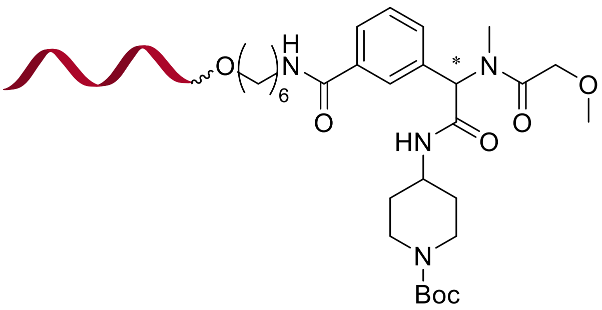

TC-5ak

MS calc. 3530.0; found: 3530.8

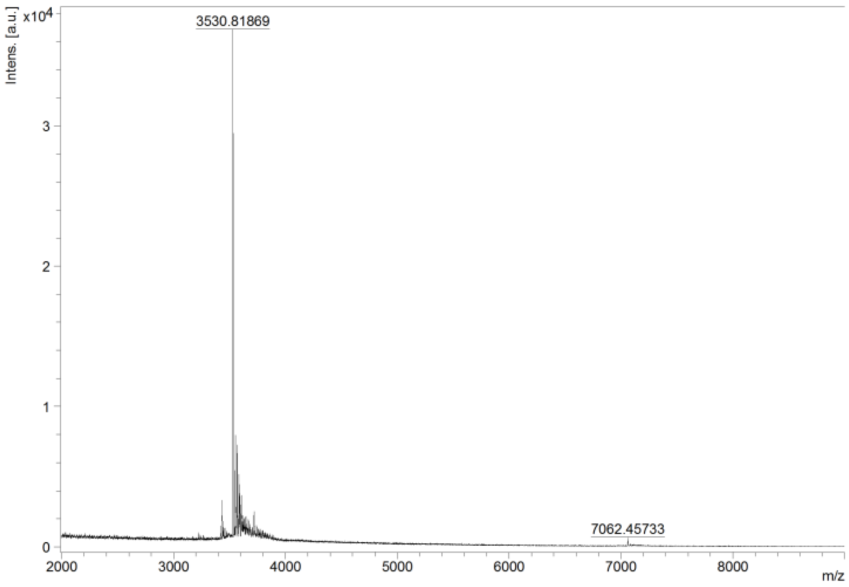

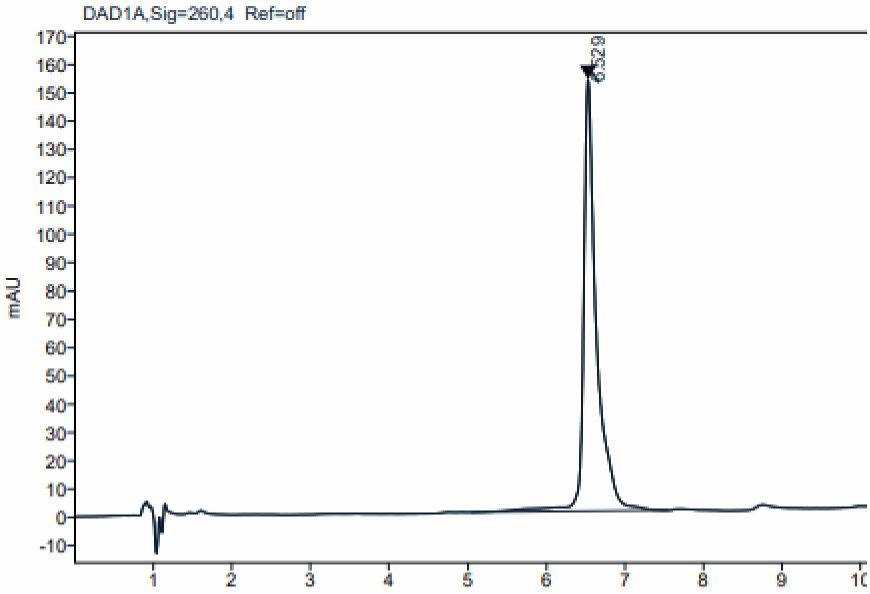

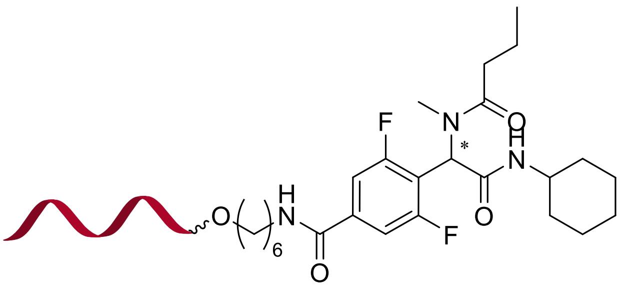

TC-5al

MS calc. 3462.5; found: 3464.1

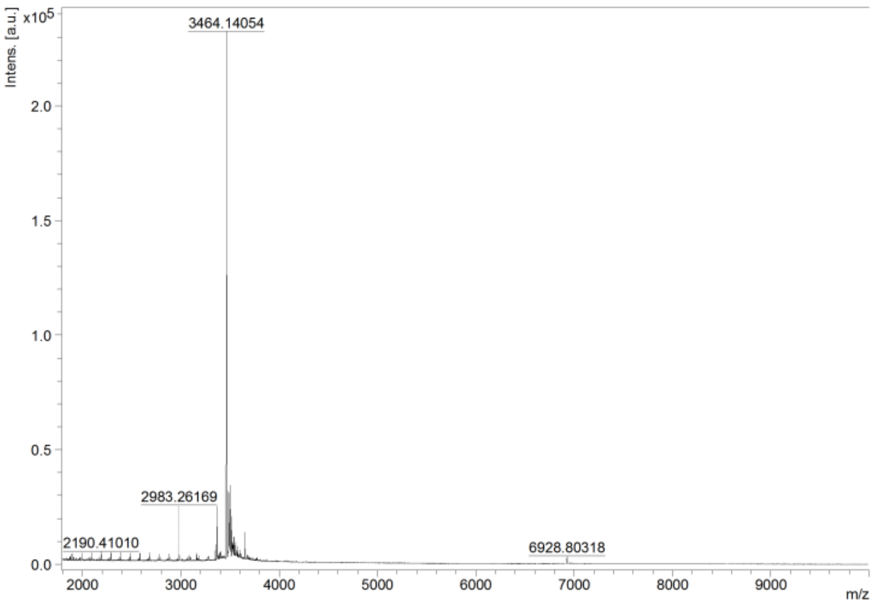

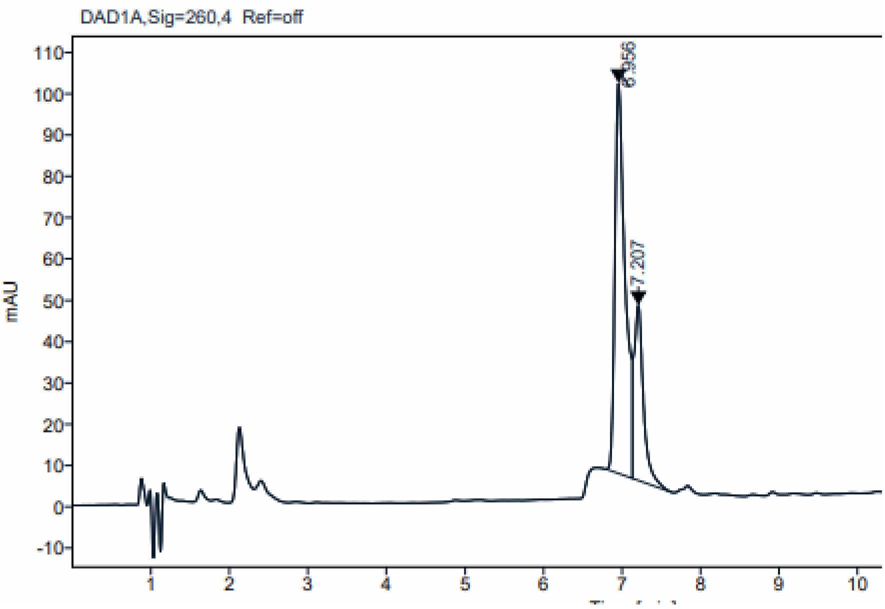

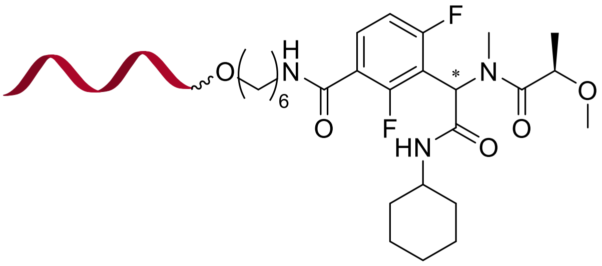

TC-5am

MS calc. 3479.0; found: 3479.9

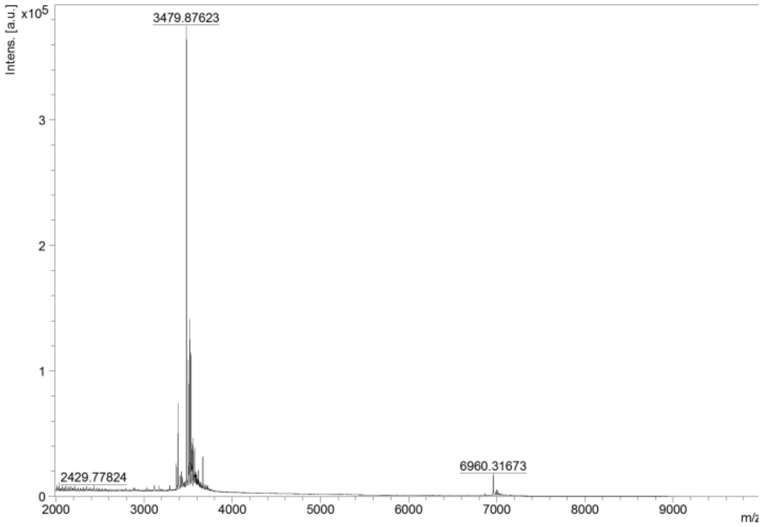

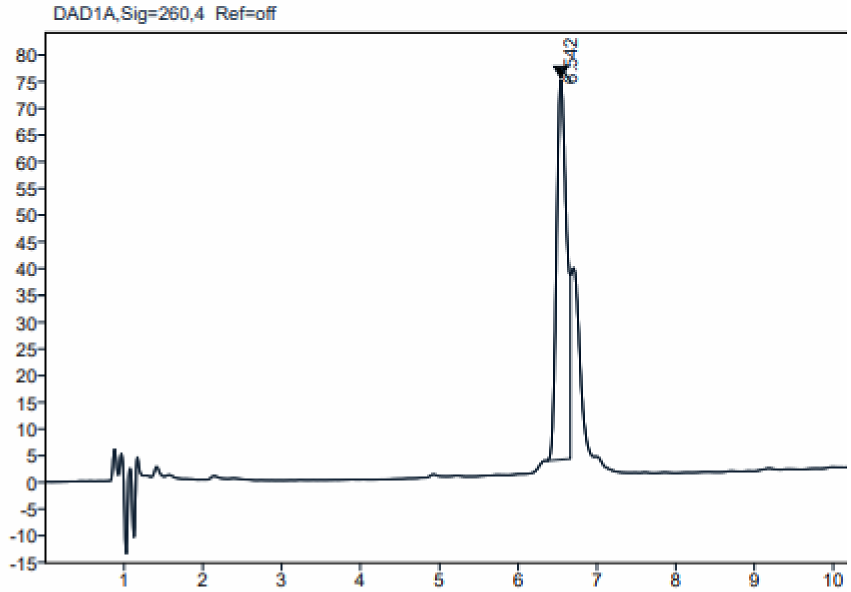

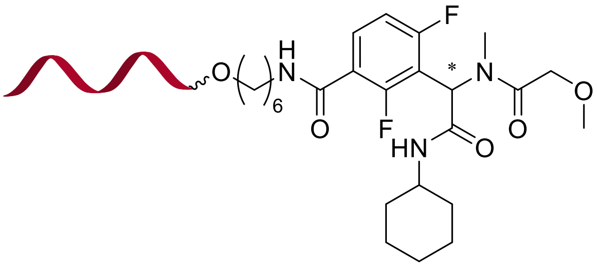

TC-5an

MS calc. 3465.0; found: 3465.7

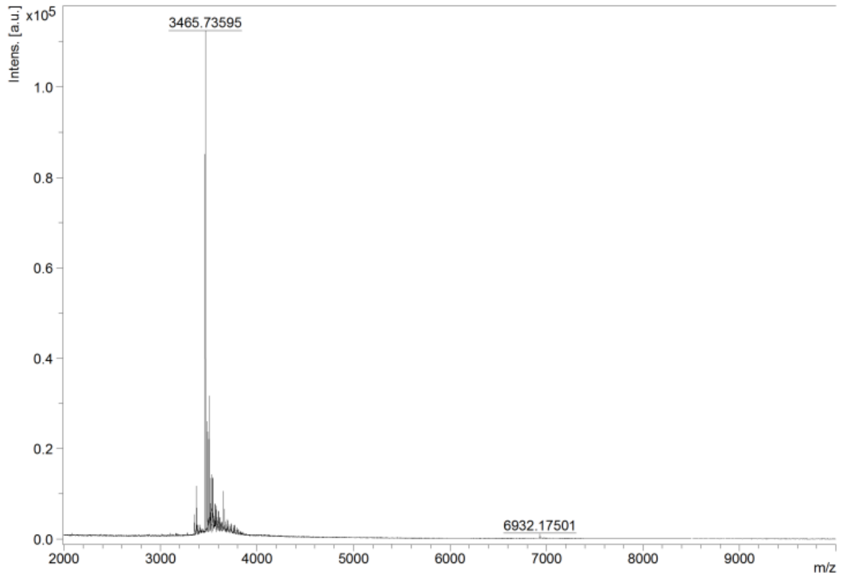

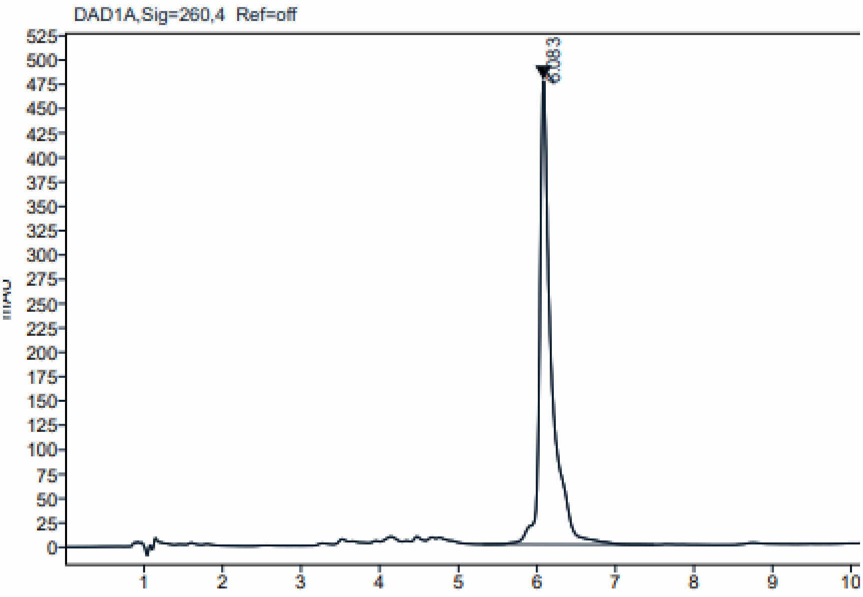

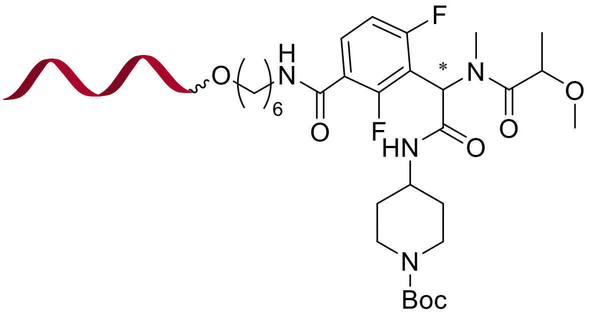

TC-5ao

MS calc. 3580.0; found: 3581.0

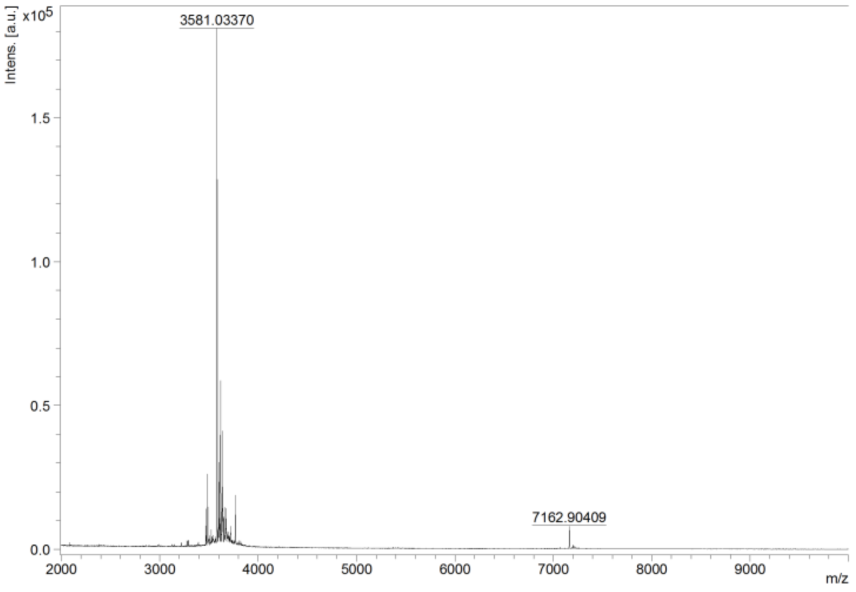

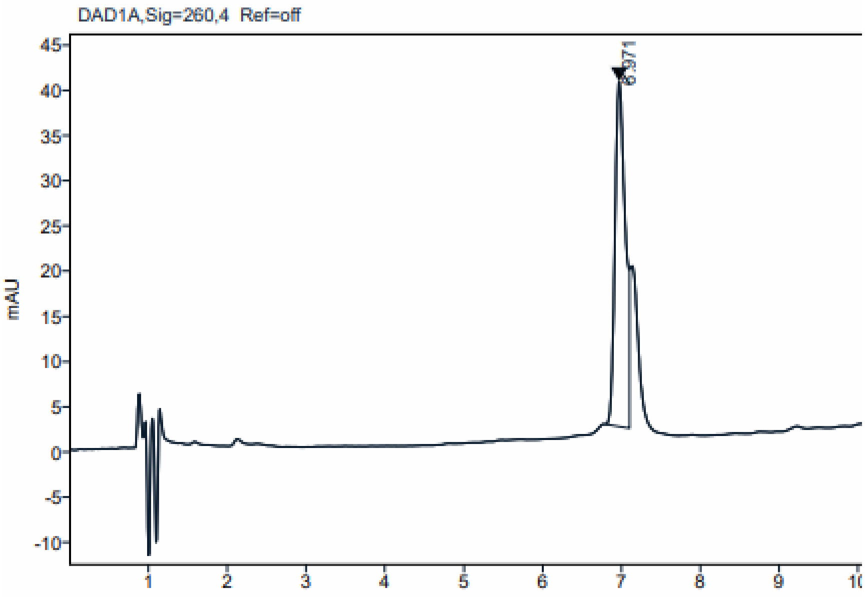

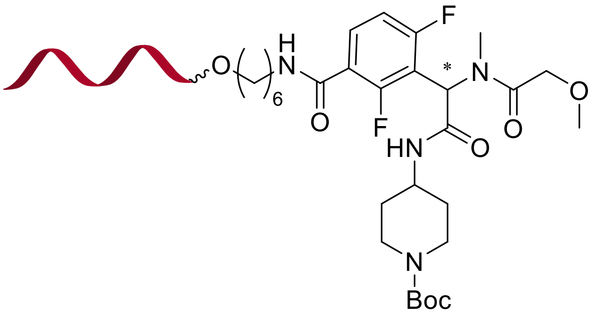

TC-5ap

MS calc. 3566.0; found: 3567.1

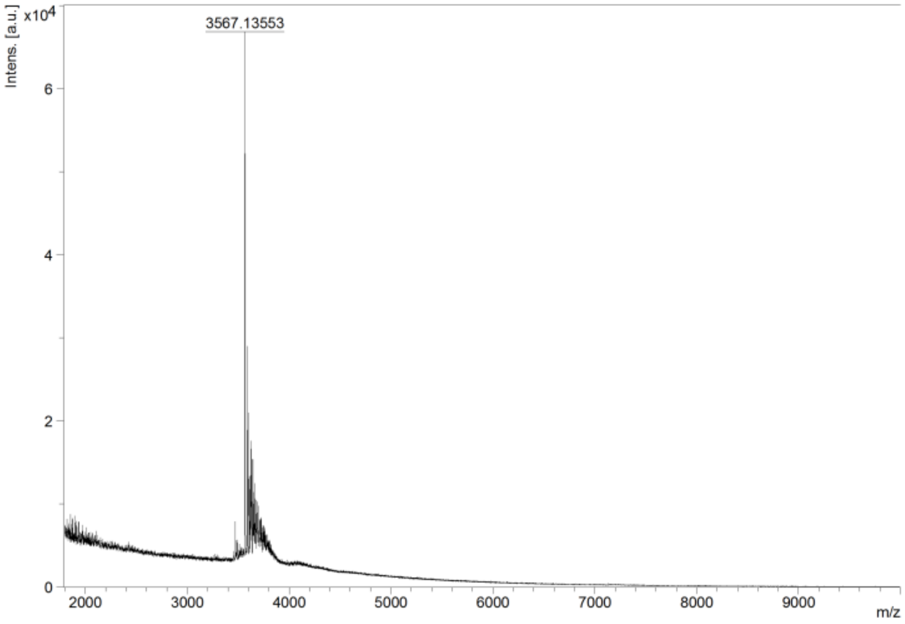

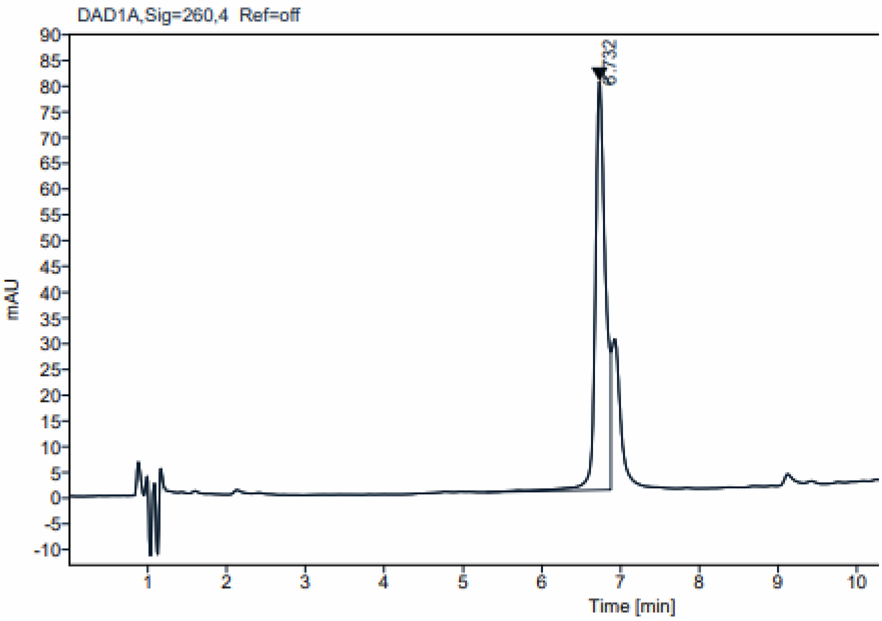

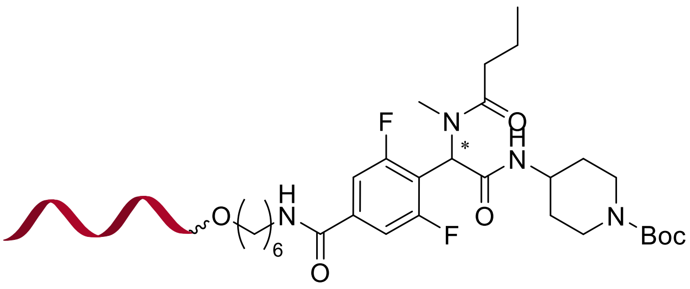

TC-5aq

MS calc. 3564.0; found: 3565.0

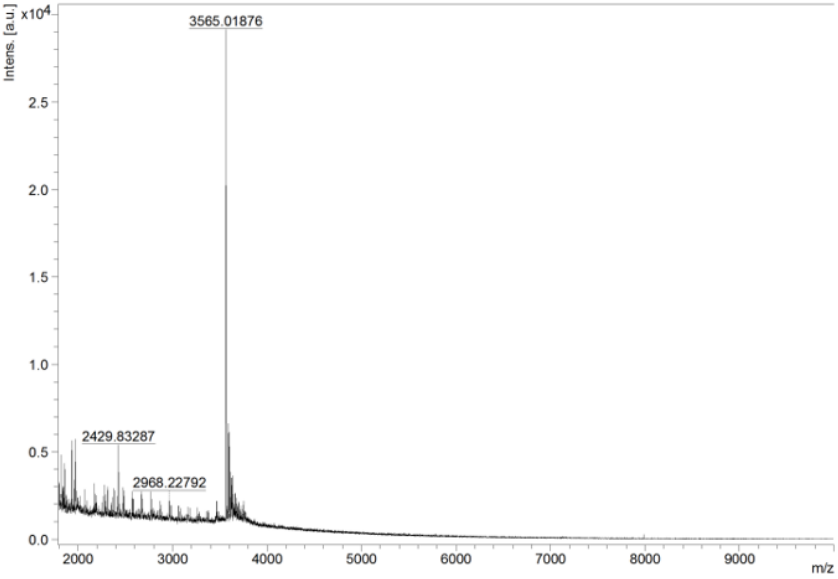

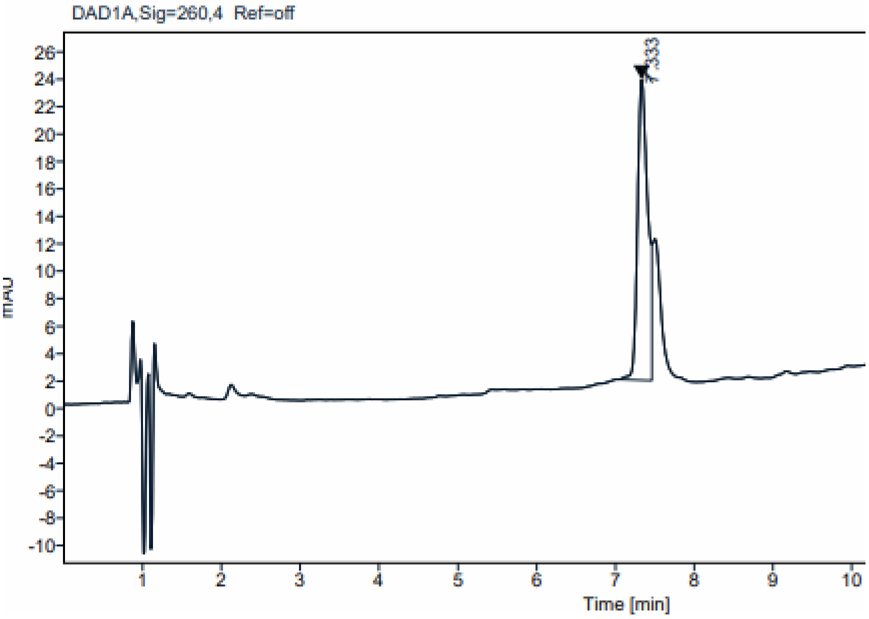

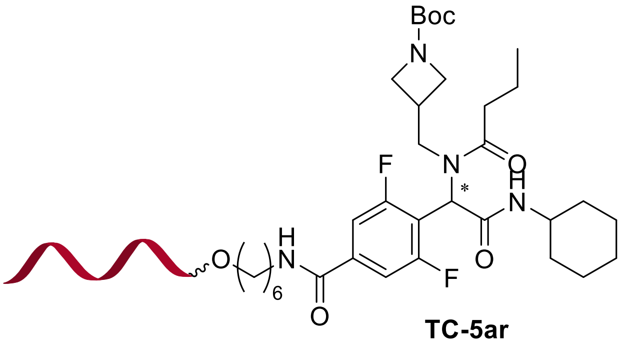

TC-5ar

MS calc. 3618.0; found: 3619.3

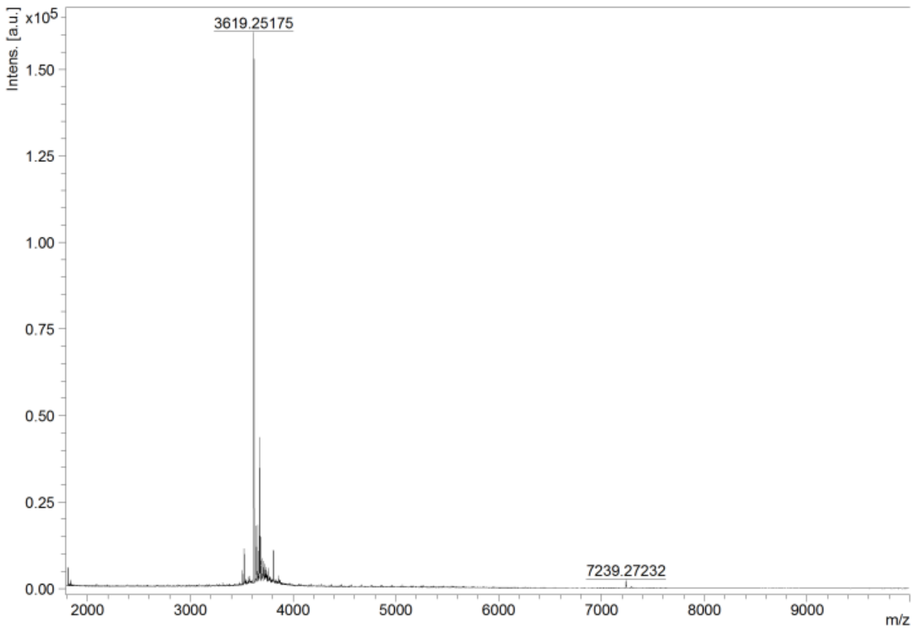

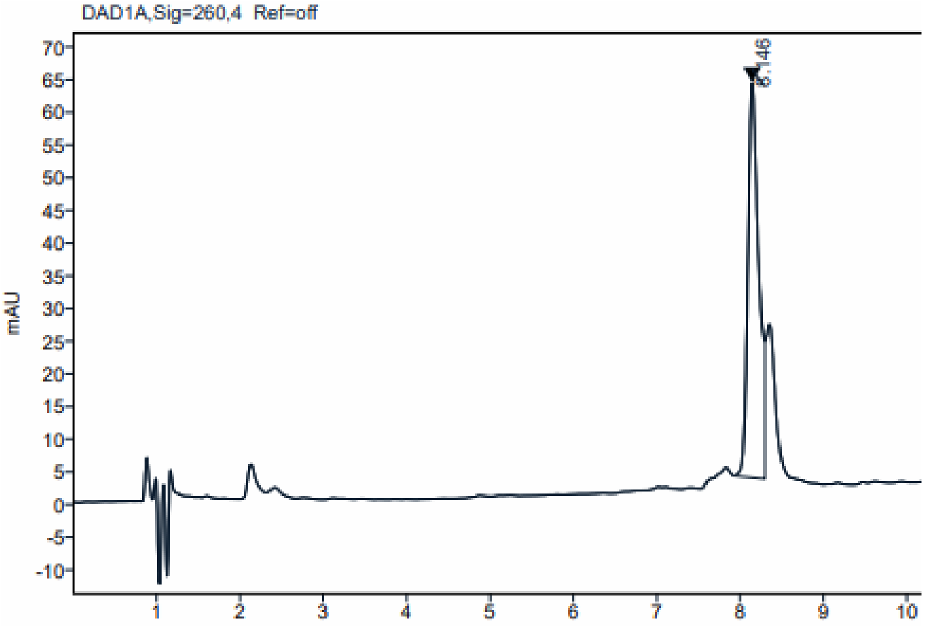

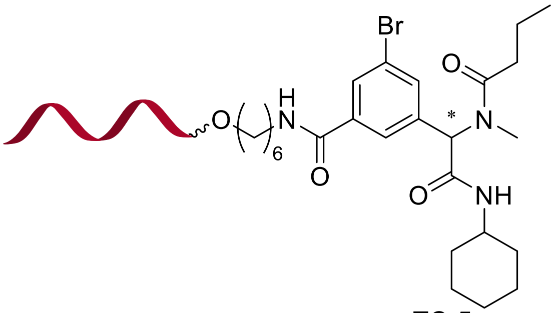

TC-5as

MS calc. 3506.0; found: 3507.7

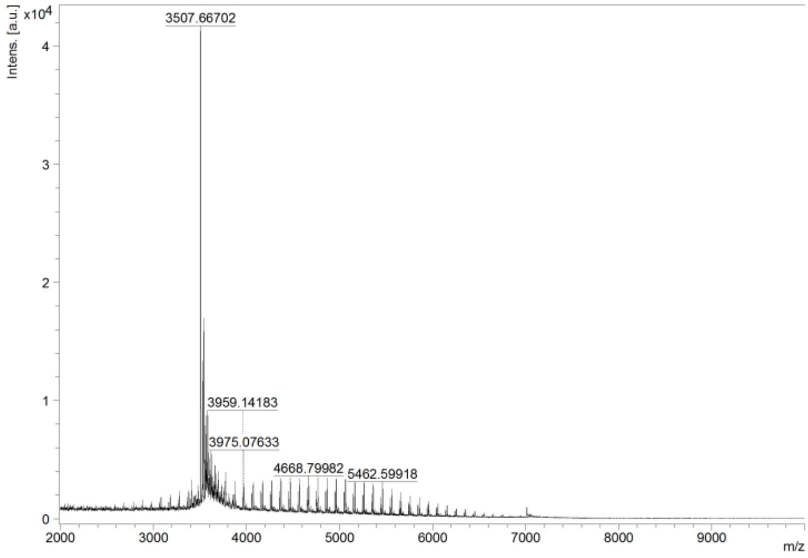

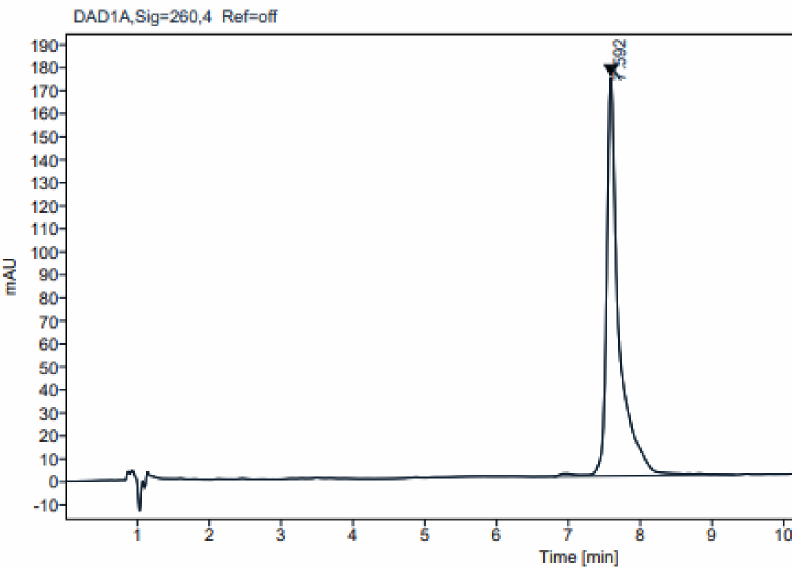

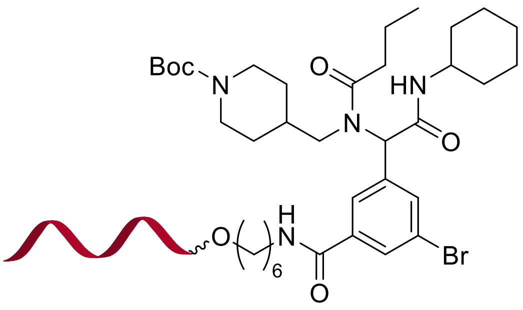

TC-5at

MS calc. 3689.0; found: 3691.0

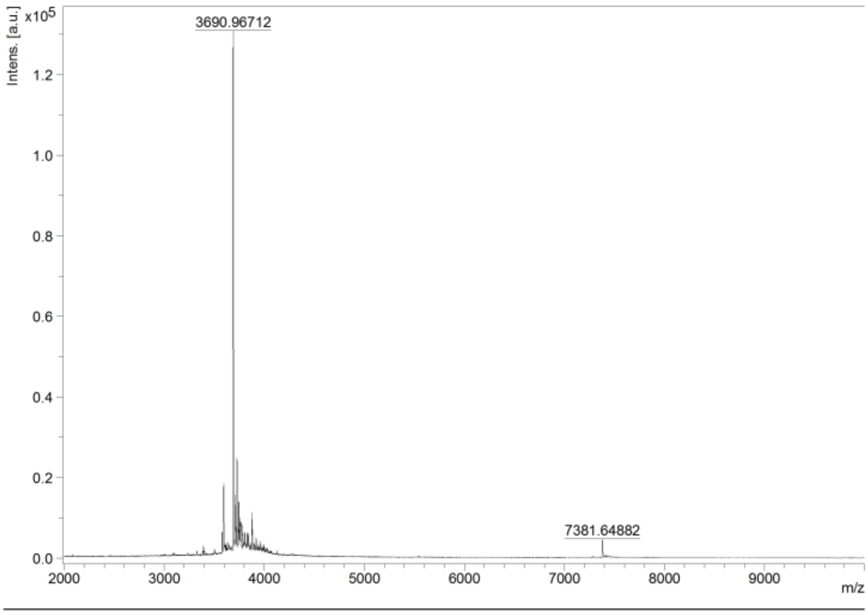

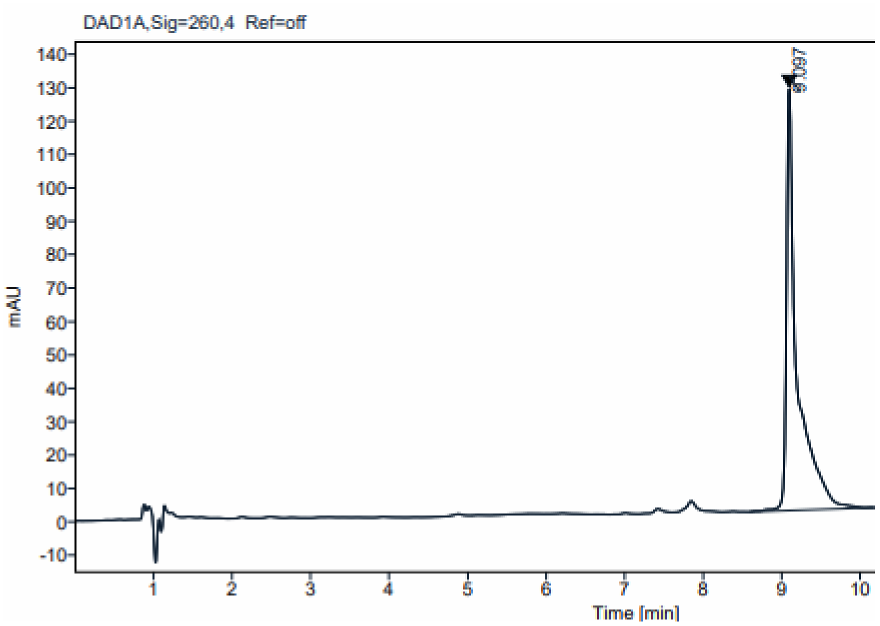

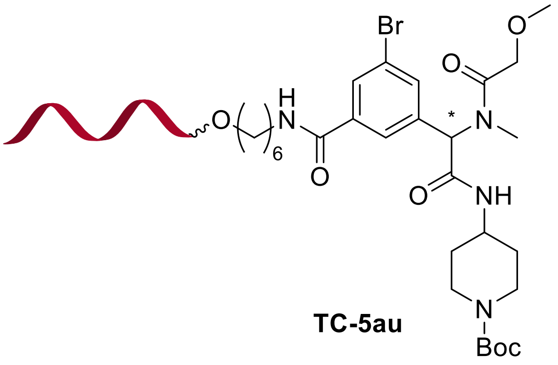

TC-5au

MS calc. 3609.0; found: 3610.9

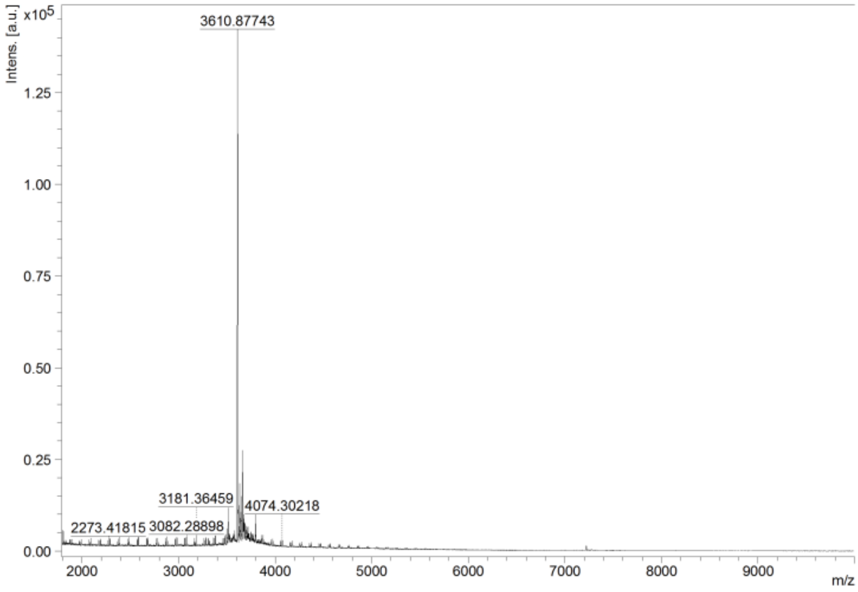

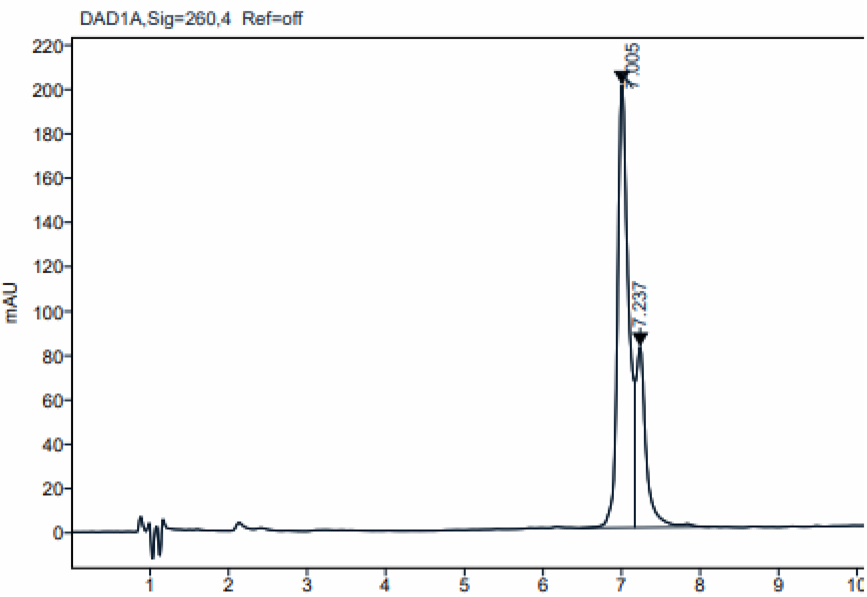

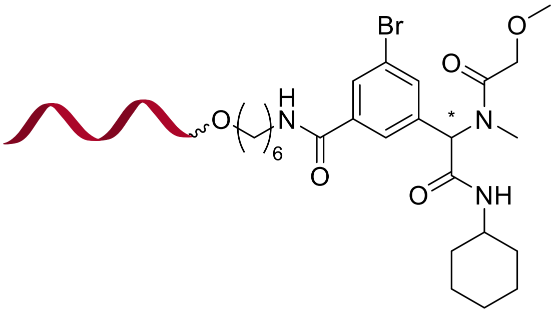

TC-5av

MS calc. 3507.0; found: 3510.00

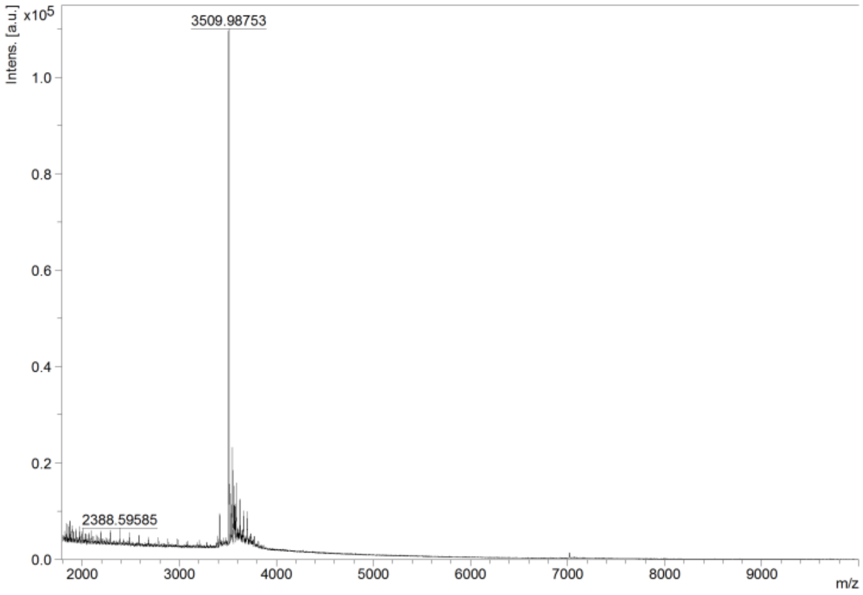

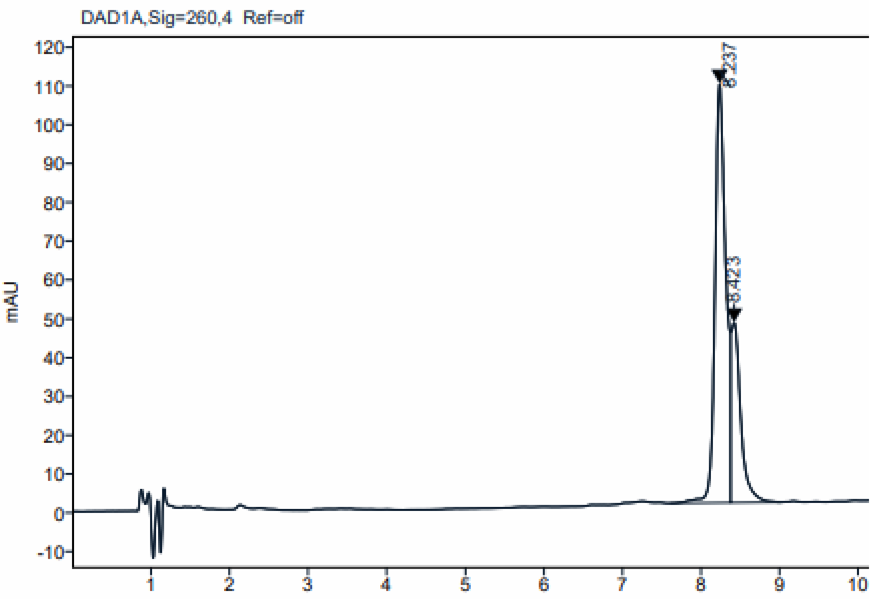

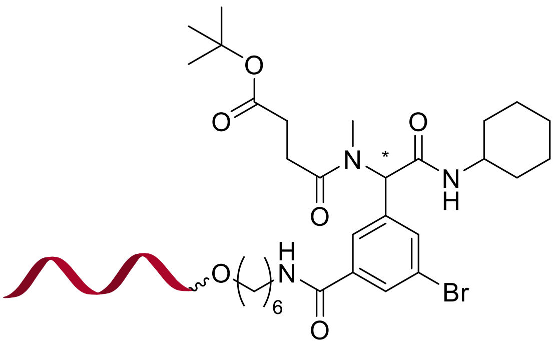

TC-5aw

MS calc. 3591.0; found: 3593.9

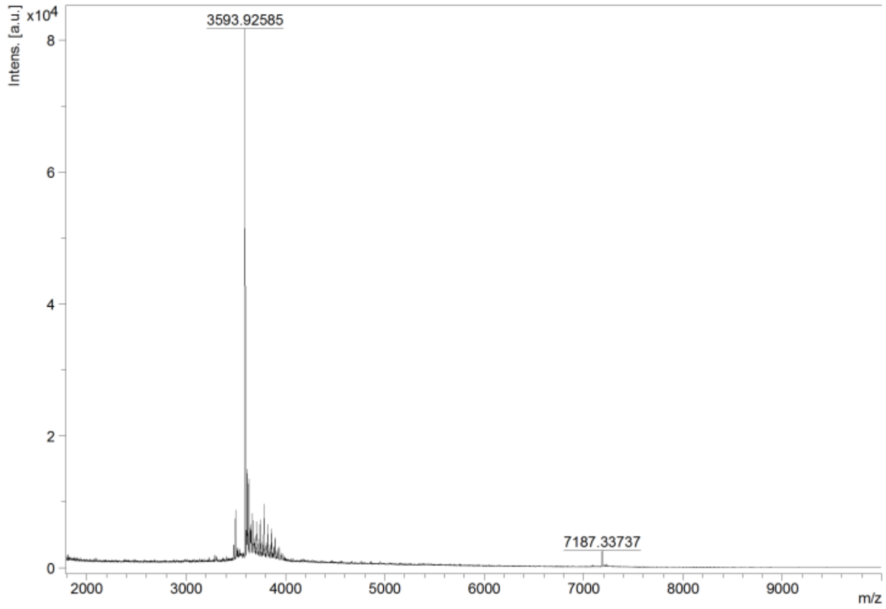

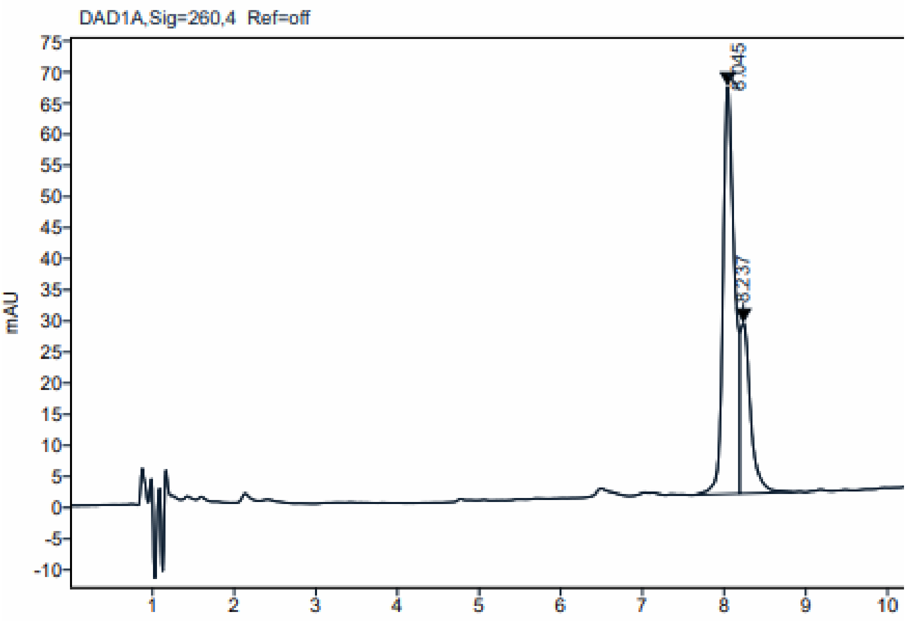

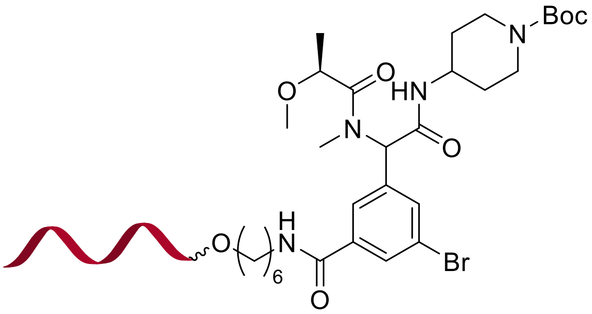

TC-5ax

MS calc. 3622.0; found: 3624.9

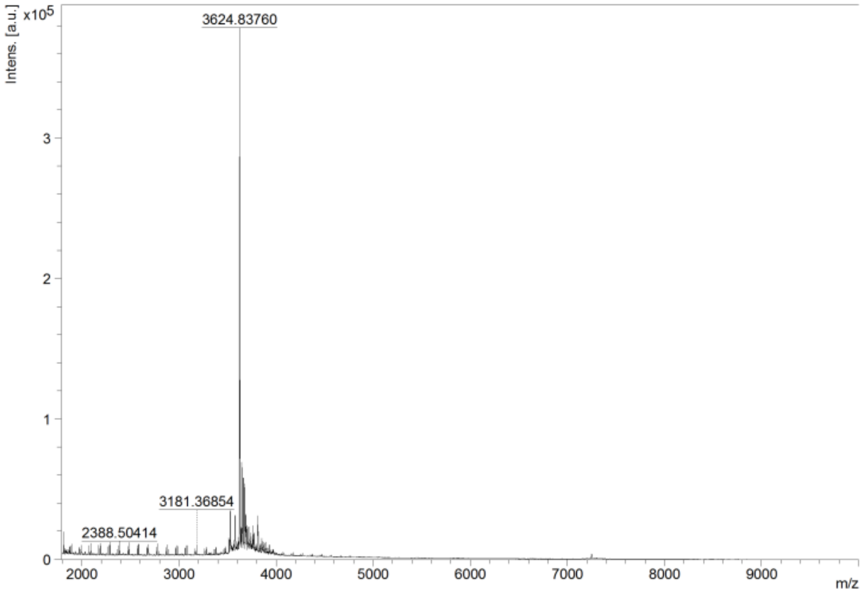

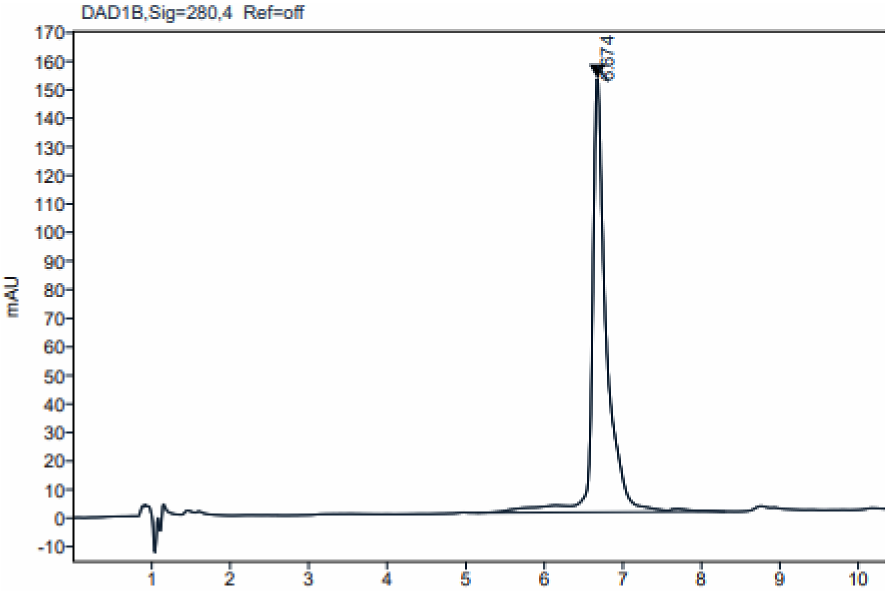

Table S5. Product conversions of the reaction scope for the Ugi four-component reaction with 10merTC-coupled aldehydes.

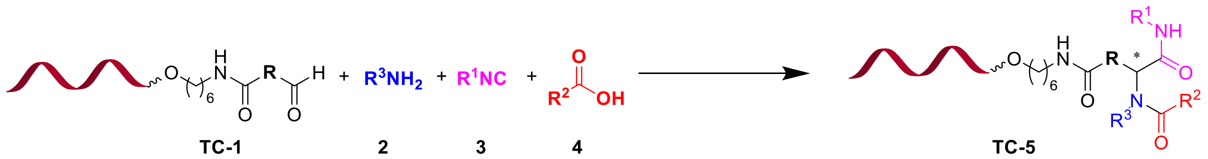

No.	R	R¹	R²	R³	Product conversion
TC-5a	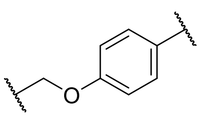	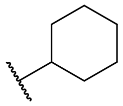	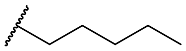	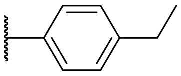	>95%
TC-5b	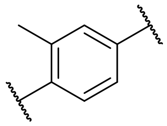	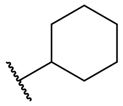	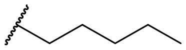	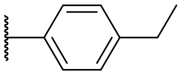	92%
TC-5c	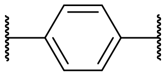	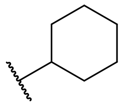	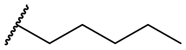	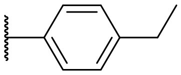	>95%
TC-5d	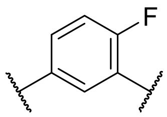	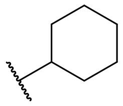	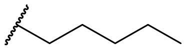	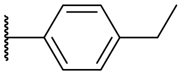	>95%
TC-5e	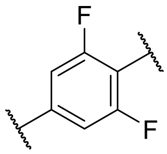	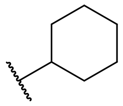	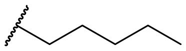	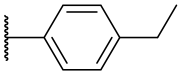	>95%
TC-5f	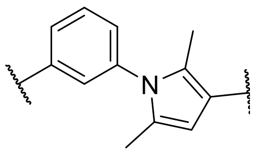	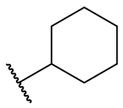	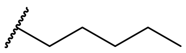	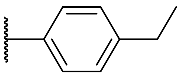	>95%
TC-5g	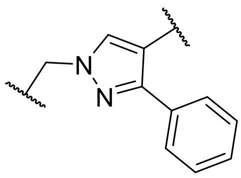	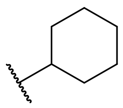	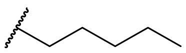	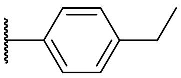	88%
TC-5h	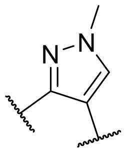	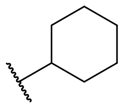	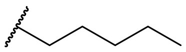	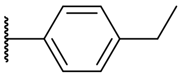	>95%

TC-5i	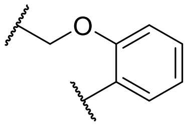	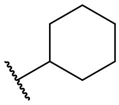	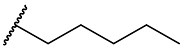	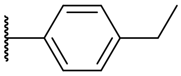	>95%
TC-5j	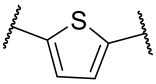	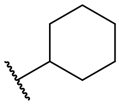	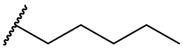	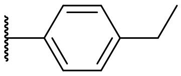	>95%
TC-5k	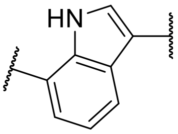	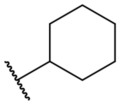	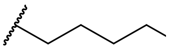	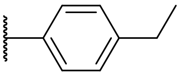	>95%
TC-5l	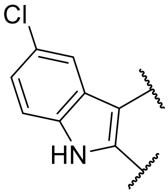	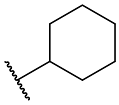	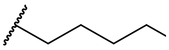	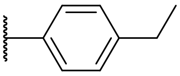	3620
TC-5m	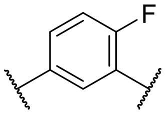	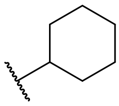	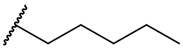	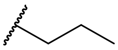	>95%
TC-5n	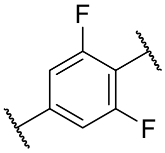	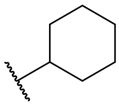	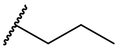	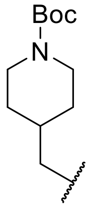	64,5%
TC-5o	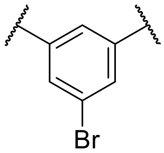	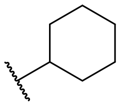	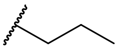	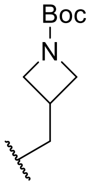	92%
TC-5p	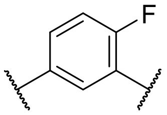	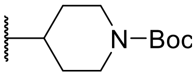	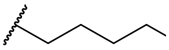	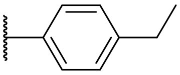	>95%
TC-5q	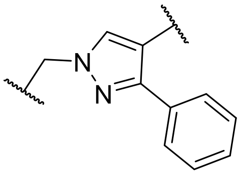	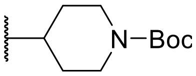	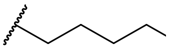	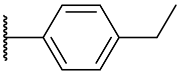	>95%
TC-5r	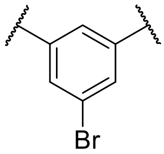	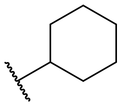	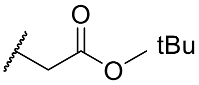	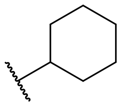	85%

TC-5s	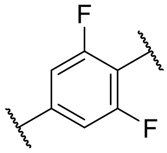	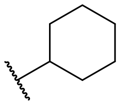	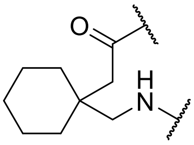		>95%
TC-5t	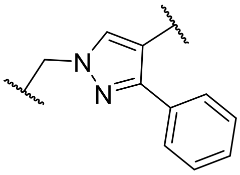	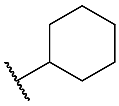	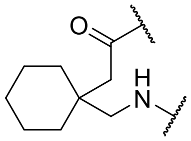		>95%
TC-5u	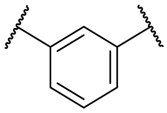	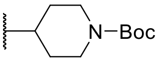	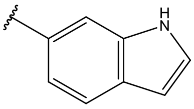	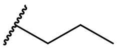	83%
TC-5v	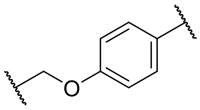	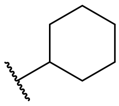	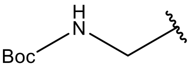	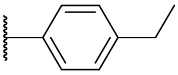	>95%
TC-5w	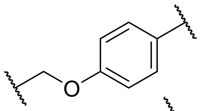	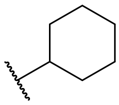	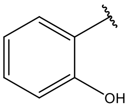	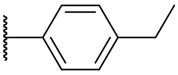	>95%
TC-5x	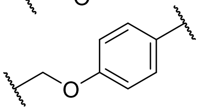	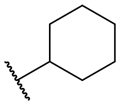	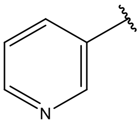	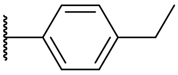	>95%
TC-5y	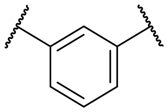	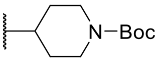	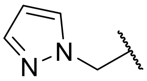	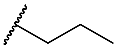	47%
TC-5z	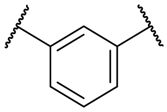	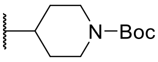	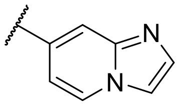	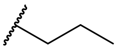	19%
TC-5aa	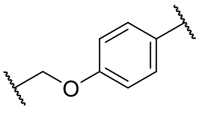	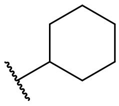	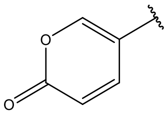	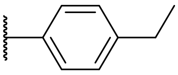	>95%
TC-5ab	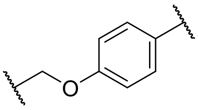	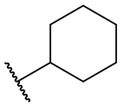	$-CH_3$	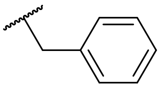	>95%
TC-5ac	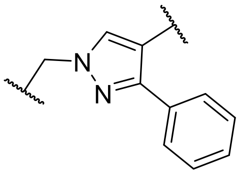	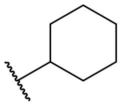	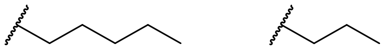		>95%

TC-5ad	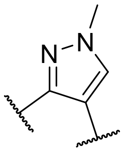	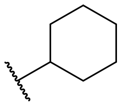	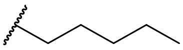	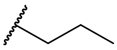	>95%
TC-5ae	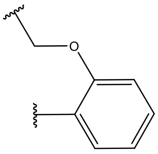	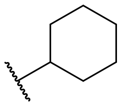	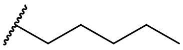	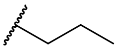	>95%
TC-5af	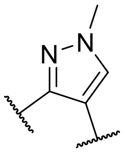	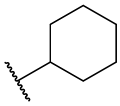		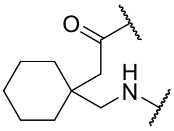	>95%
TC-5ag	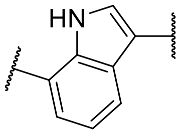	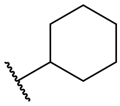		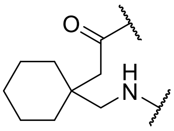	31%
TC-5ah	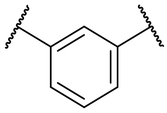	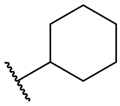	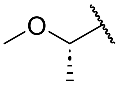	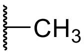	>95%
TC-5ai	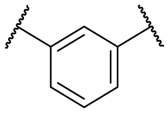	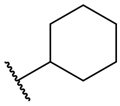	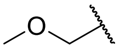	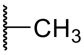	>95%
TC-5aj	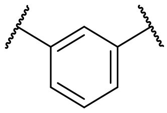	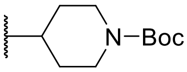	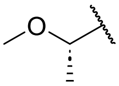	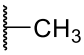	>95%
TC-5ak	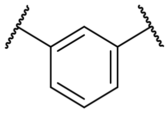	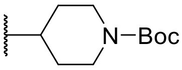	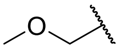	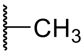	>95%
TC-5al	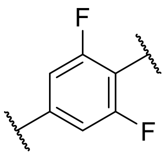	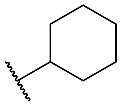	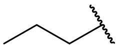	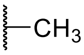	91%
TC-5am	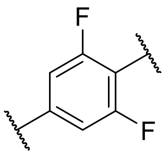	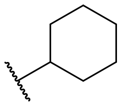	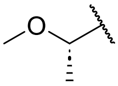	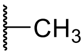	68%

TC-5an	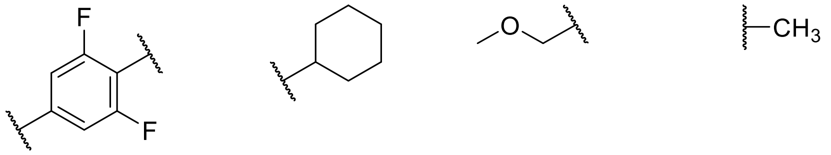	57%
TC-5ao	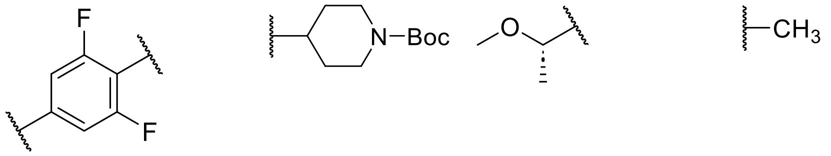	43,6%
TC-5ap	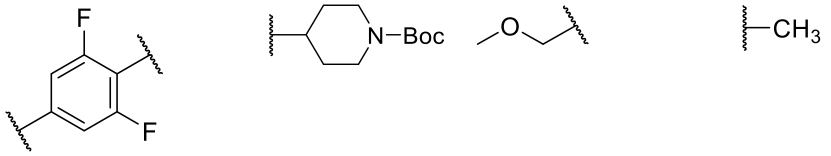	47%
TC-5aq	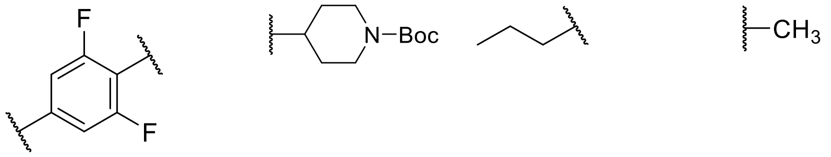	39%
TC-5ar	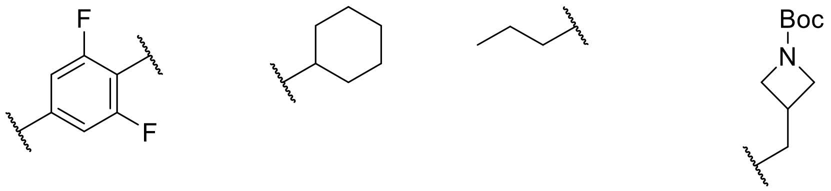	39,5%
TC-5as	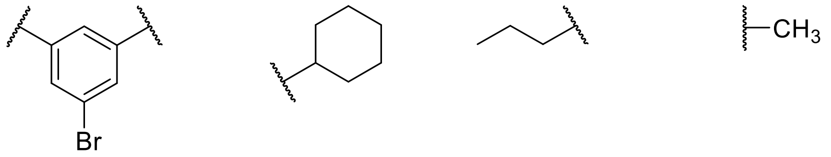	92%
TC-5at	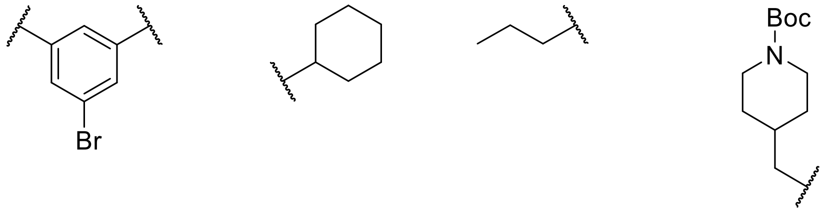	>95%
TC-5au	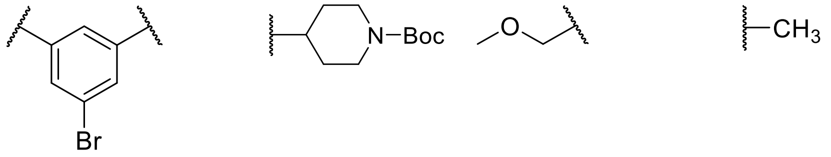	>95%
TC-5av	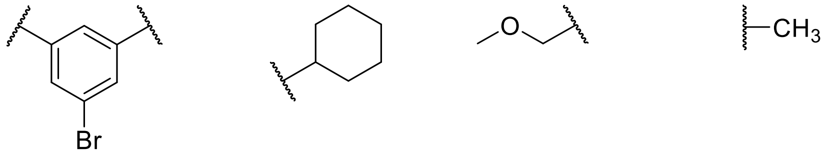	>95%
TC-5aw	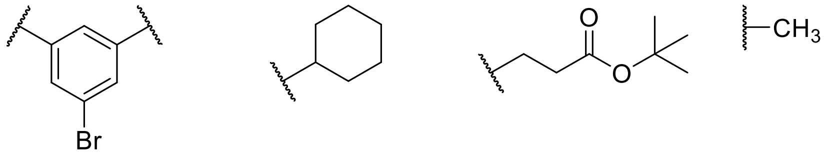	53,4%

TC-5ax	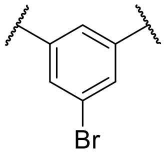	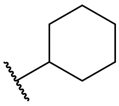	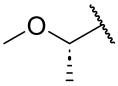	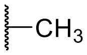	>95%

HPLC traces of representative examples of crude U-4CR products

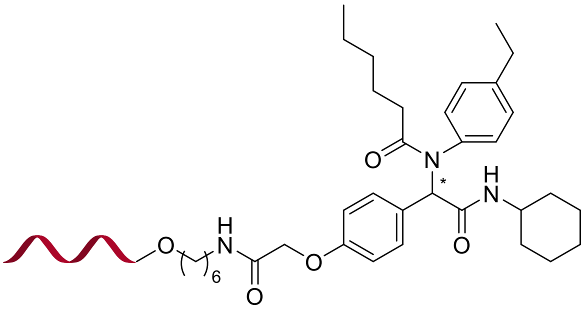

TC-5a

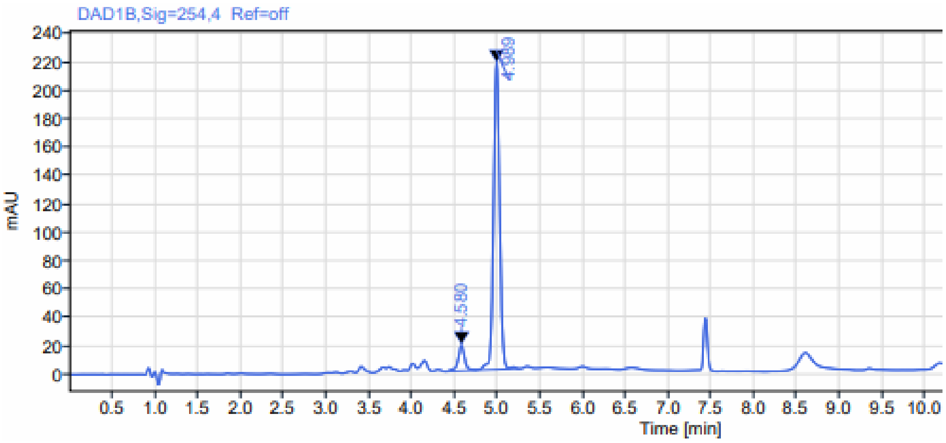

starting material

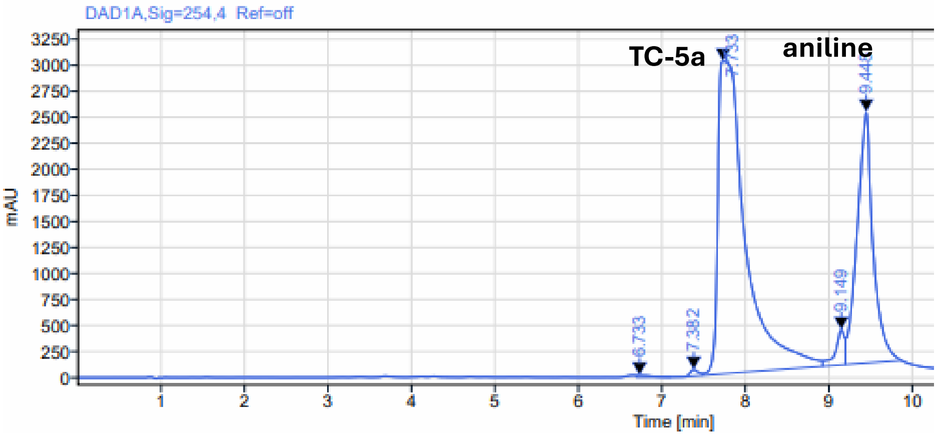

crude

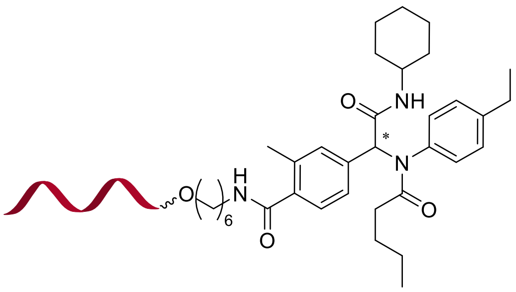

TC-5b

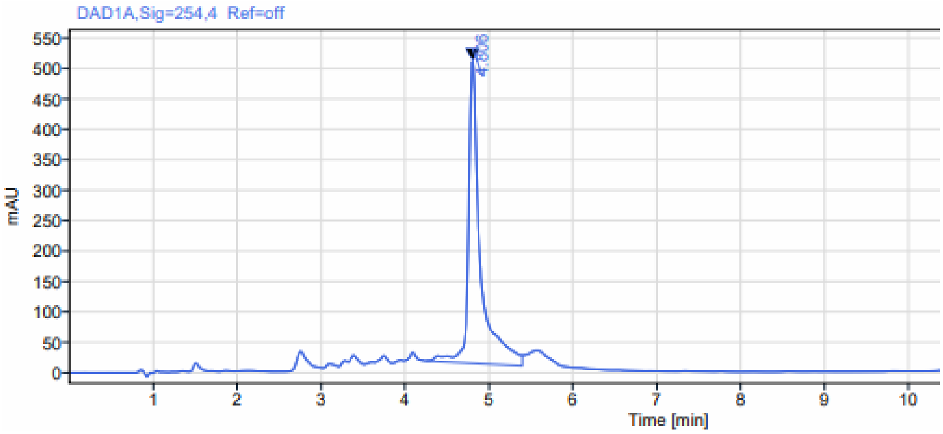

starting material

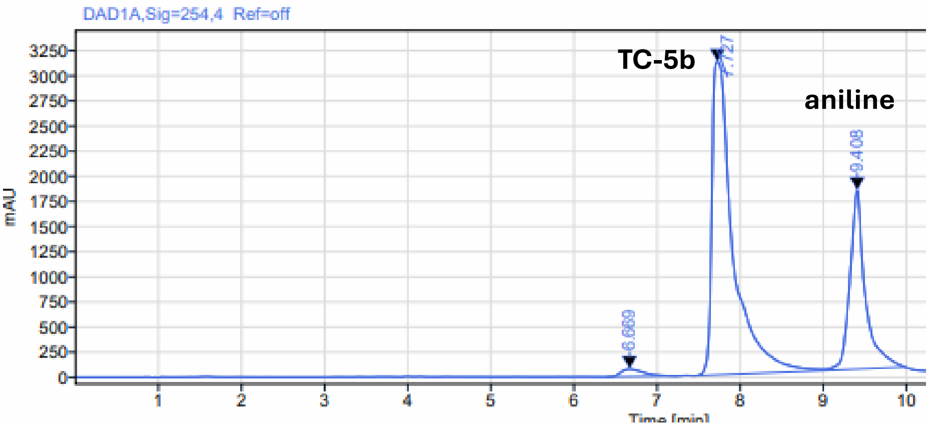

crude

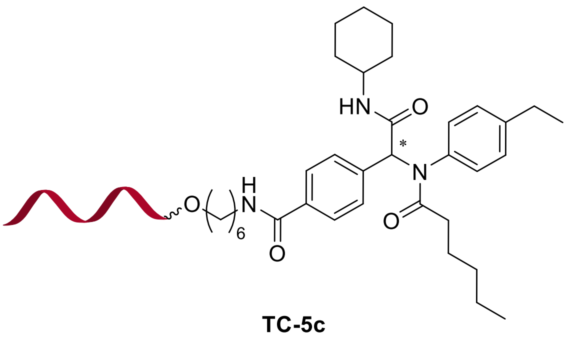

TC-5c

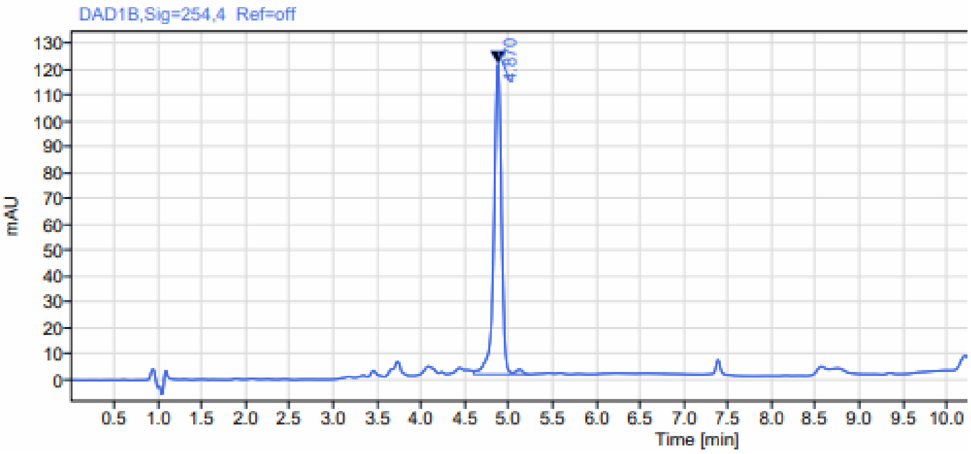

starting material

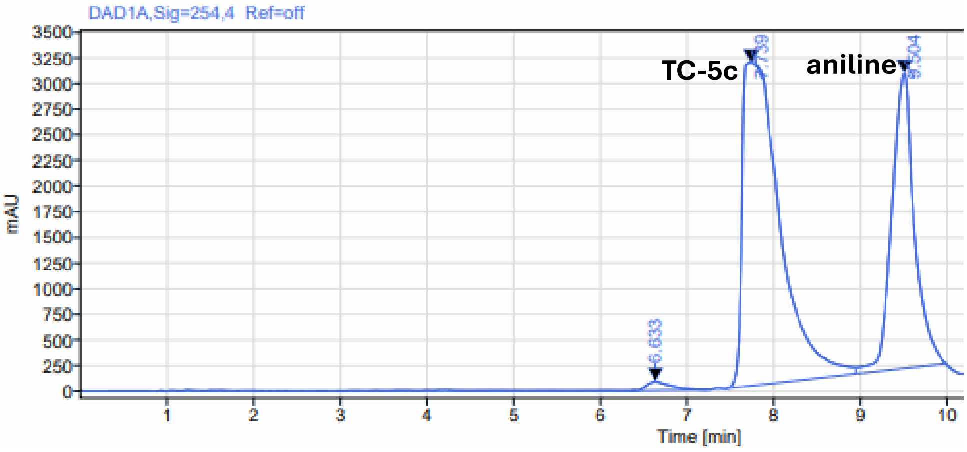

crude

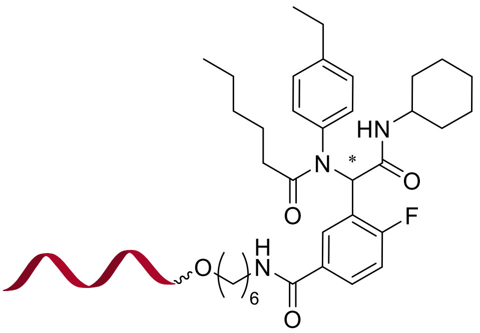

TC-5d

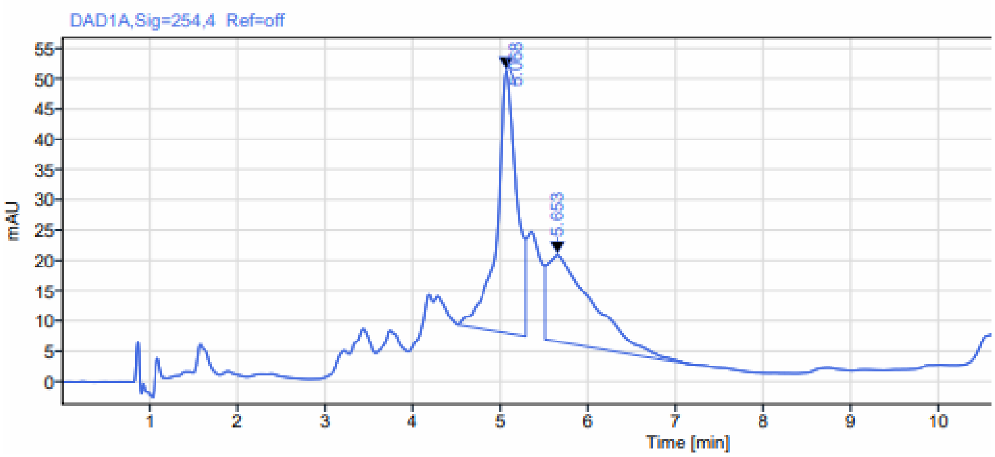

starting material

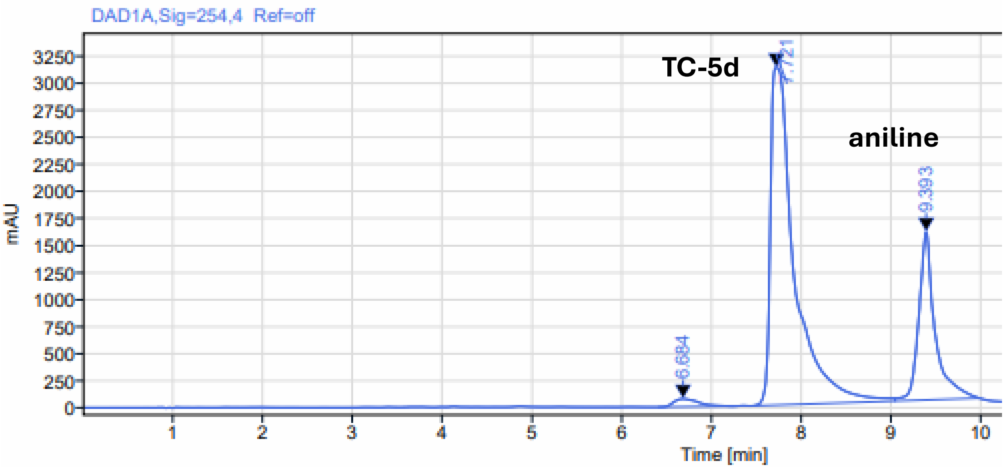

crude

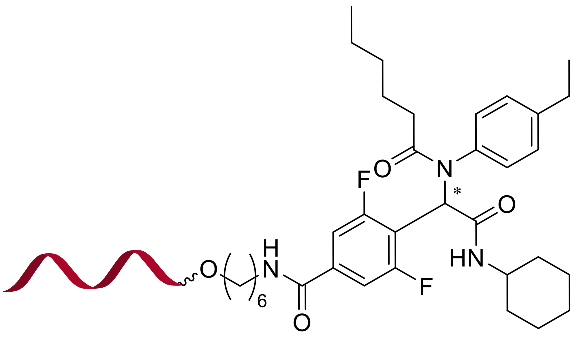

TC-5e

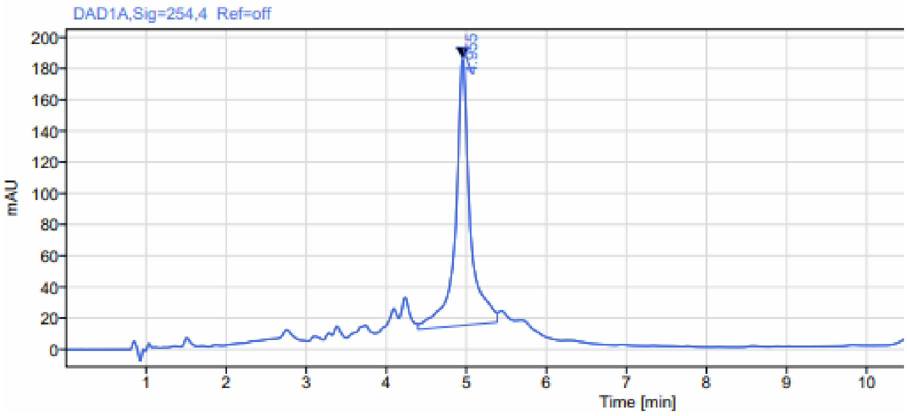

starting material

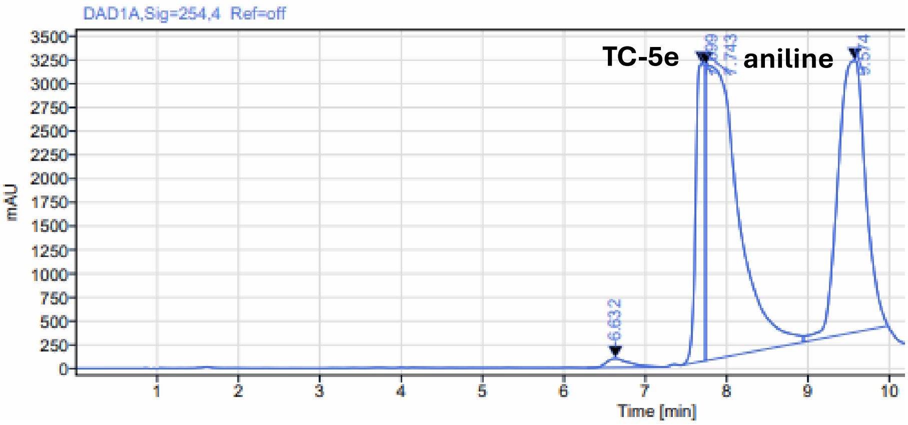

crude

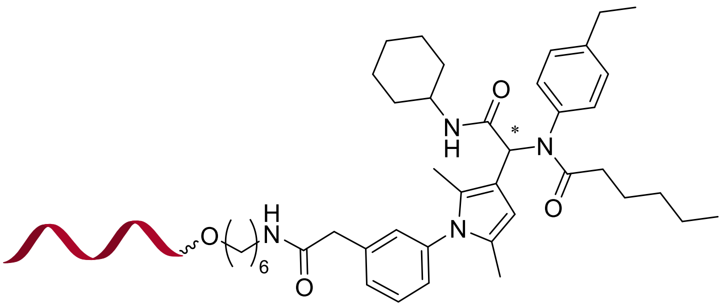

TC-5f

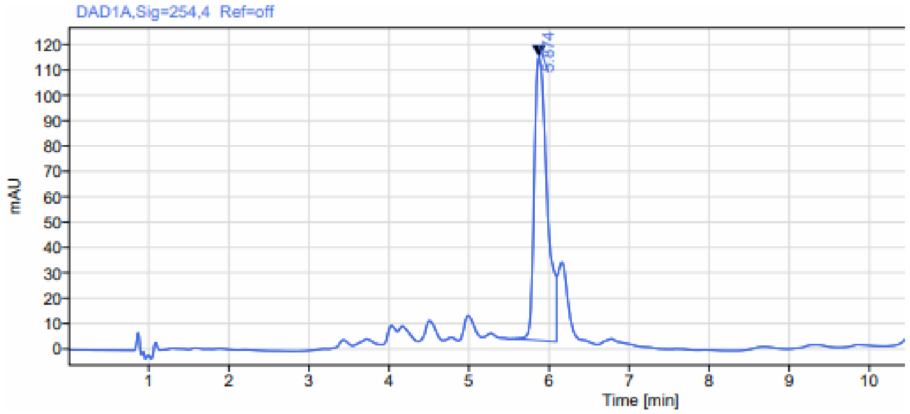

starting material

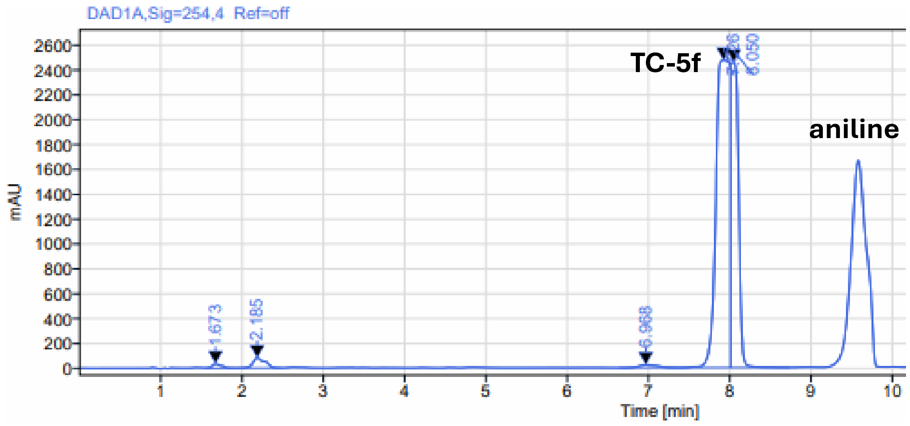

crude

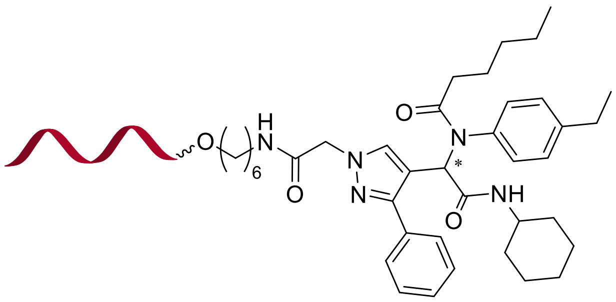

TC-5g

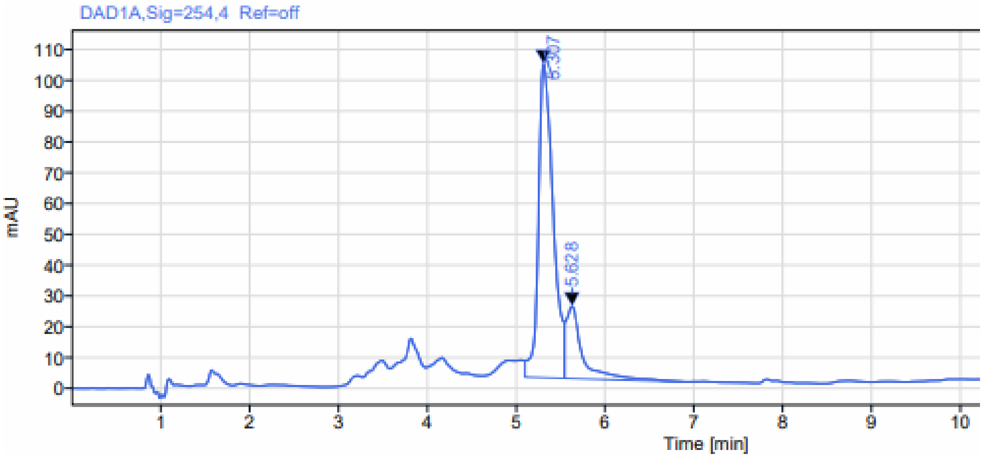

starting material

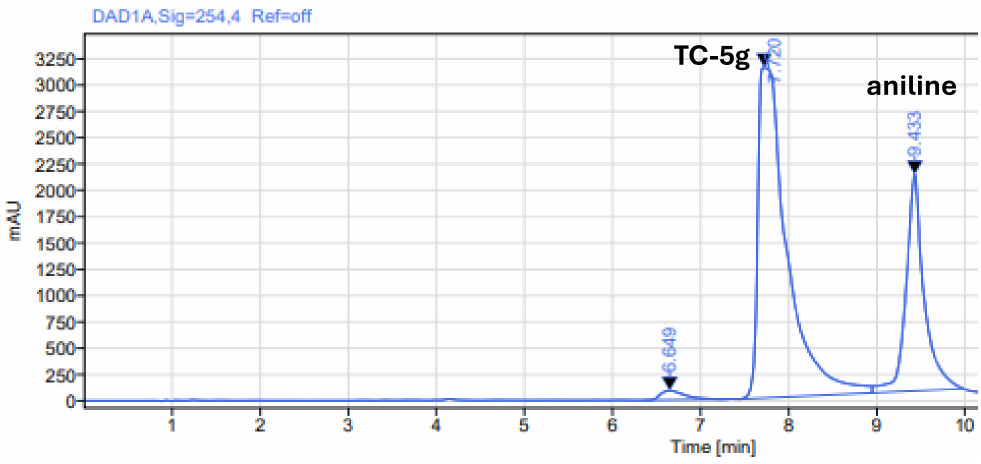

crude

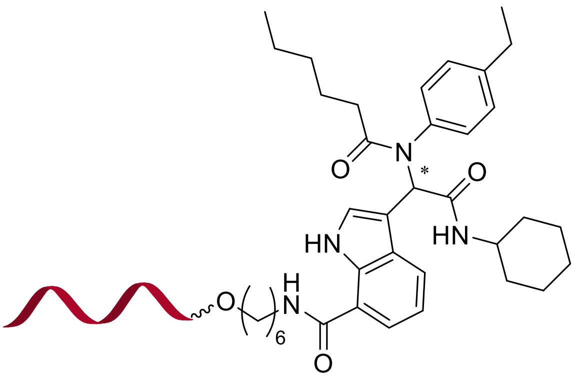

TC-5k

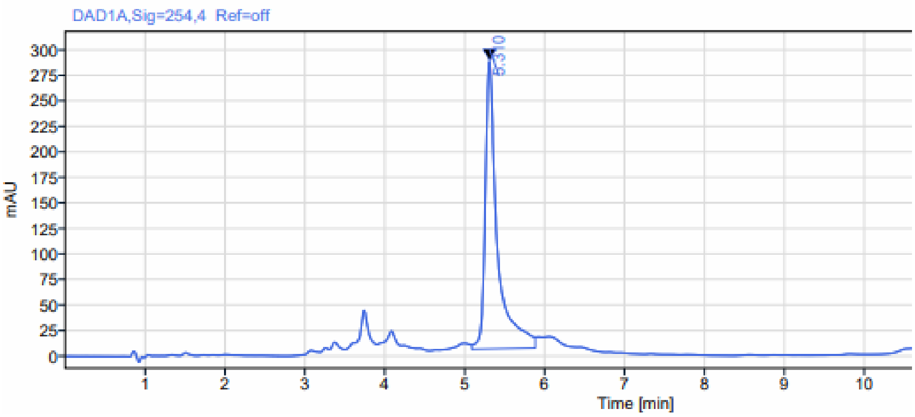

starting material

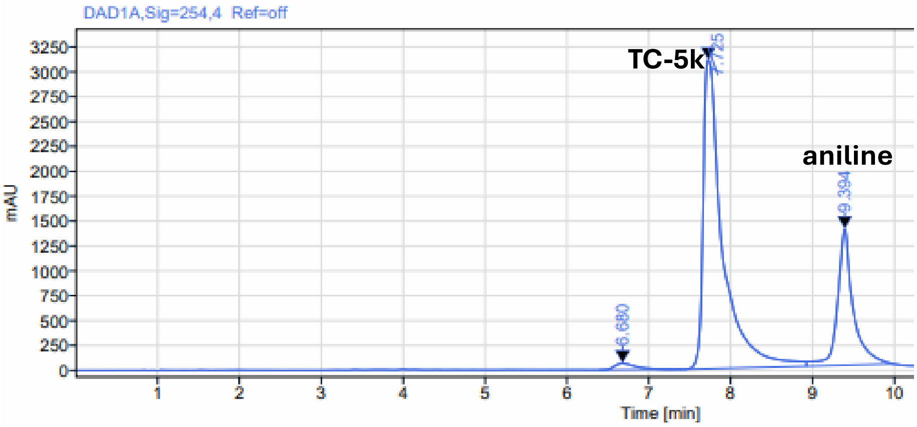

crude

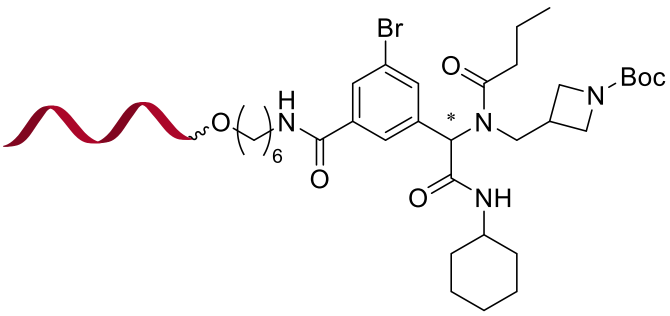

TC-5o

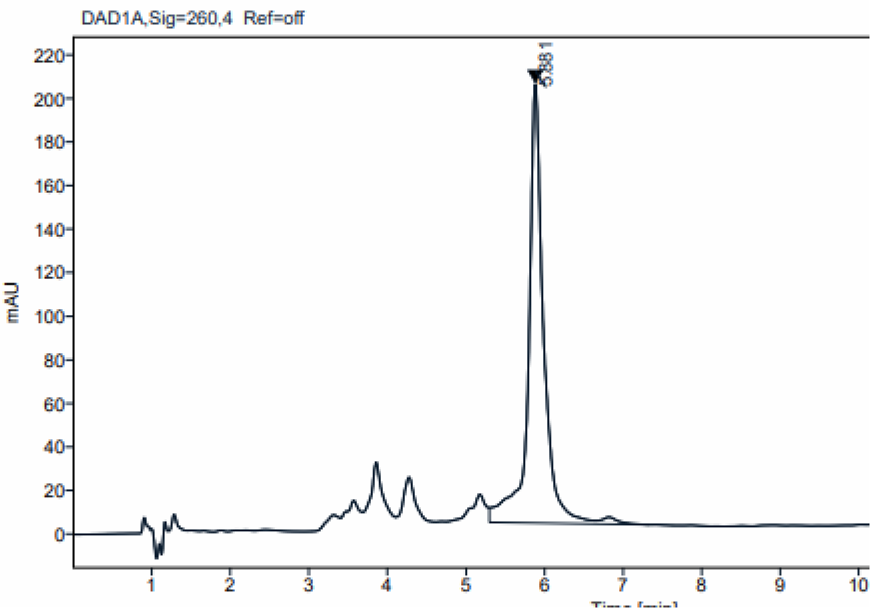

starting material

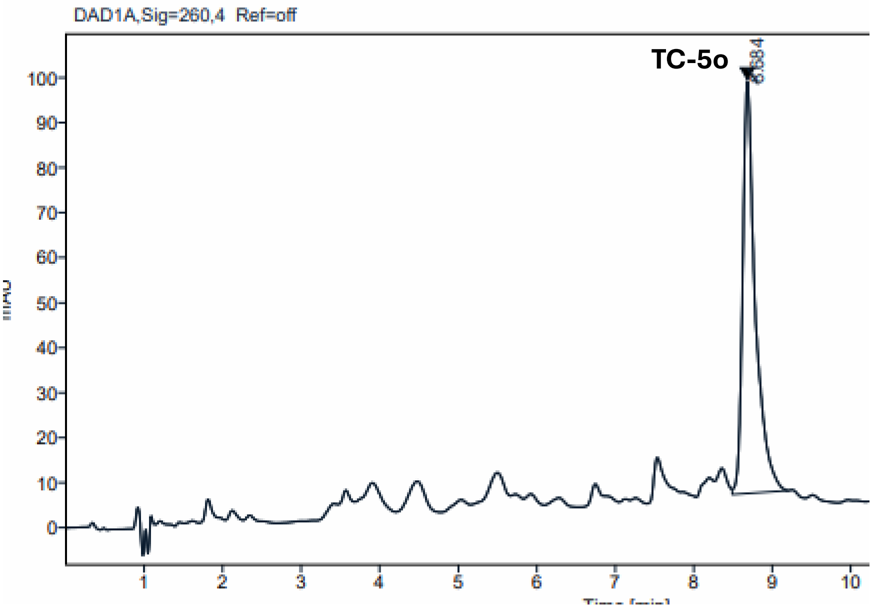

crude

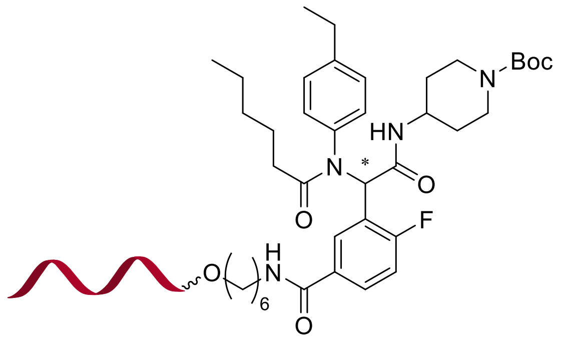

TC-5p

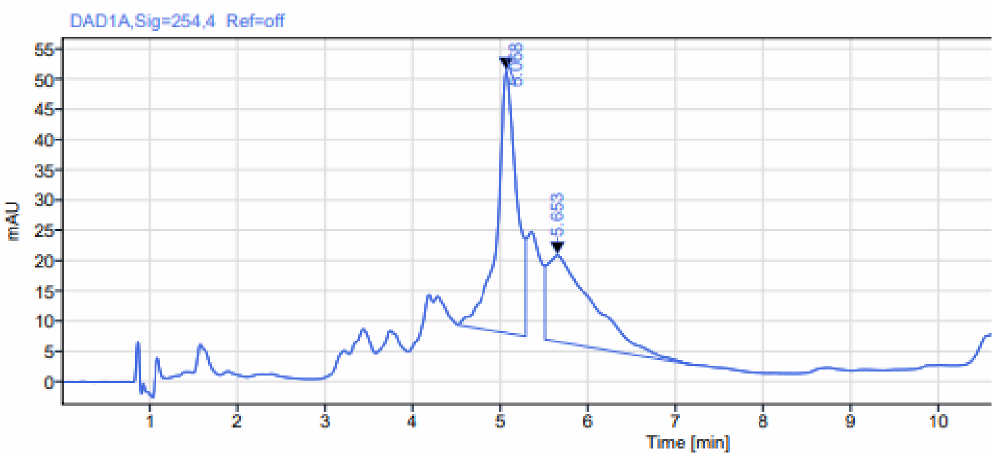

starting material

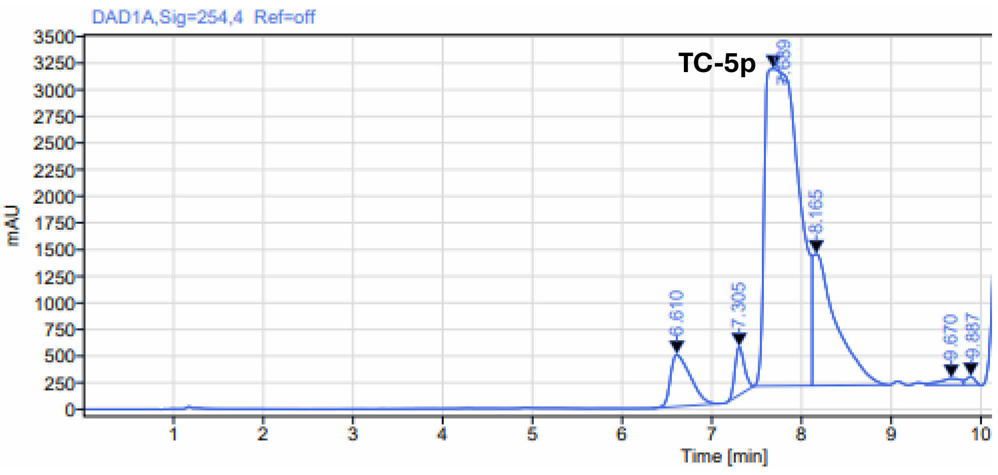

crude

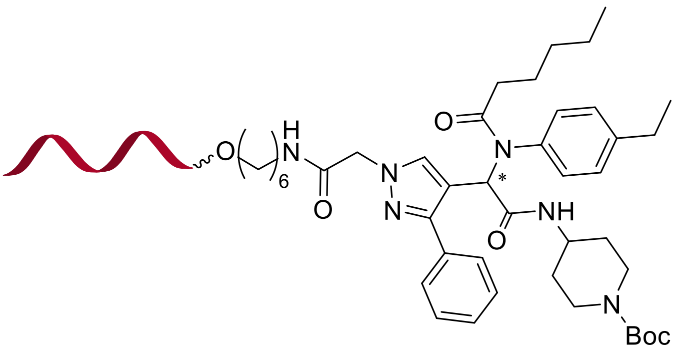

TC-5q

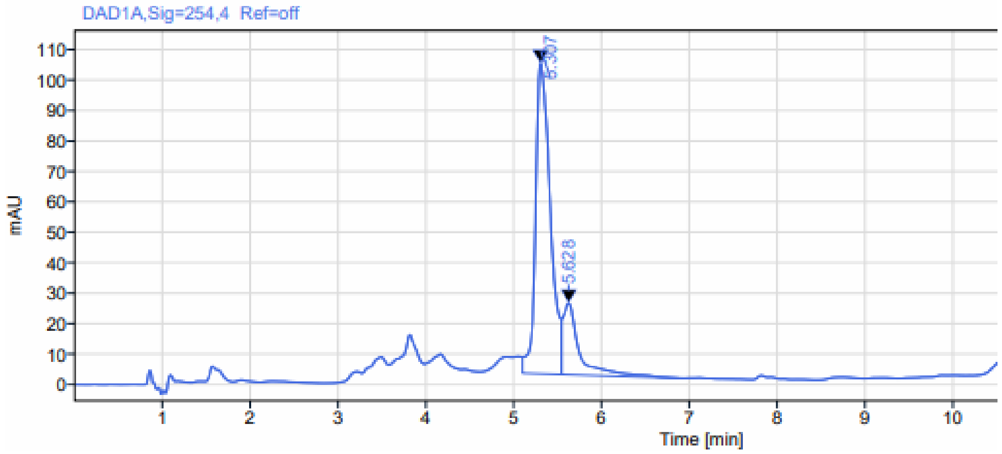

starting material

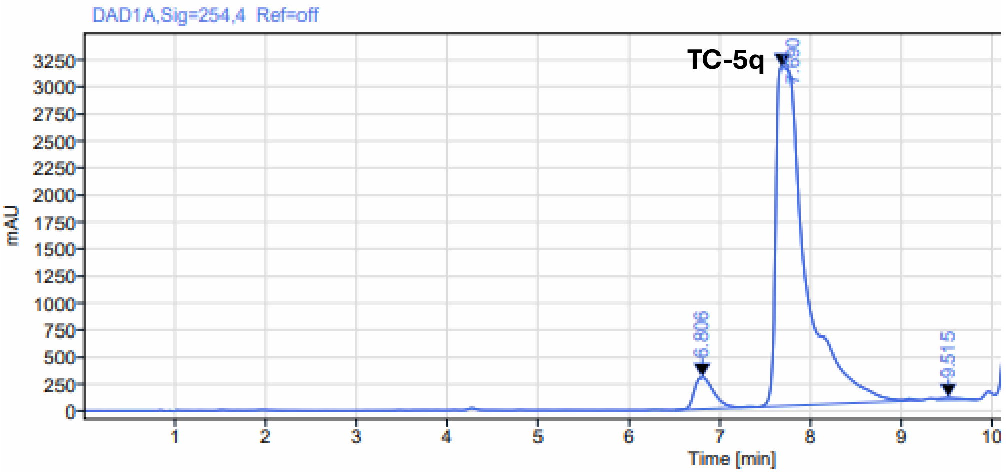

crude

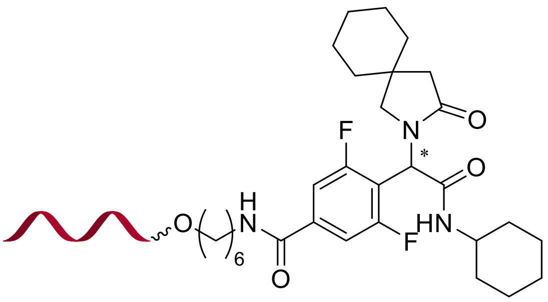

TC-5s

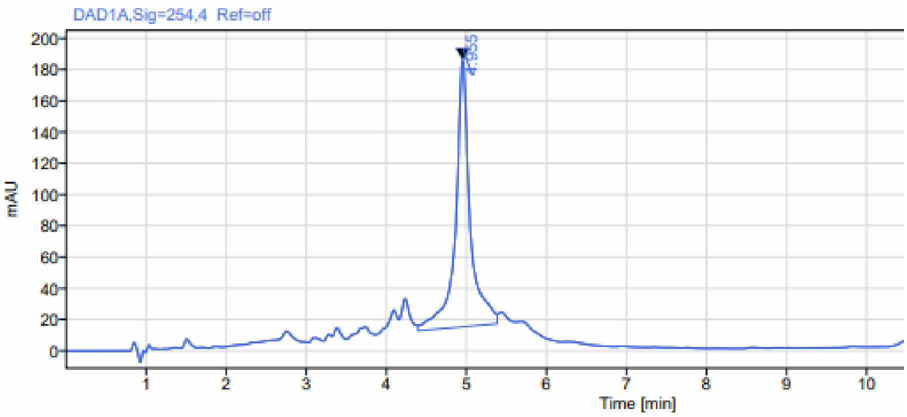

starting material

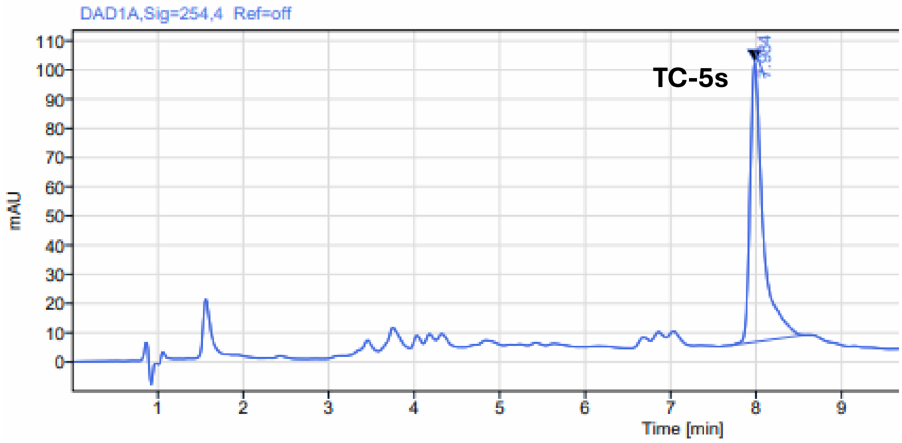

crude

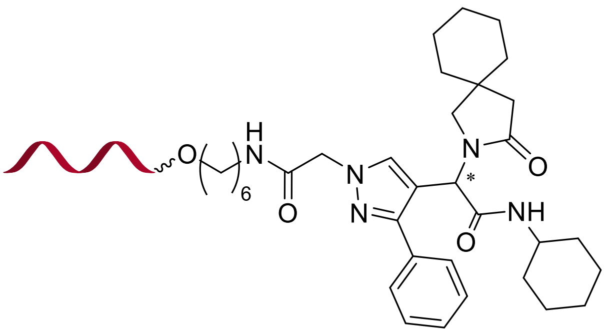

TC-5t

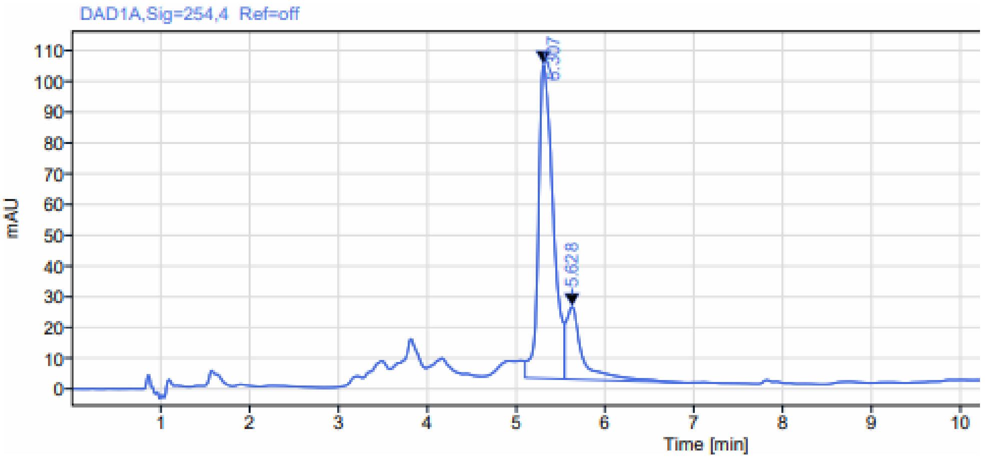

starting material

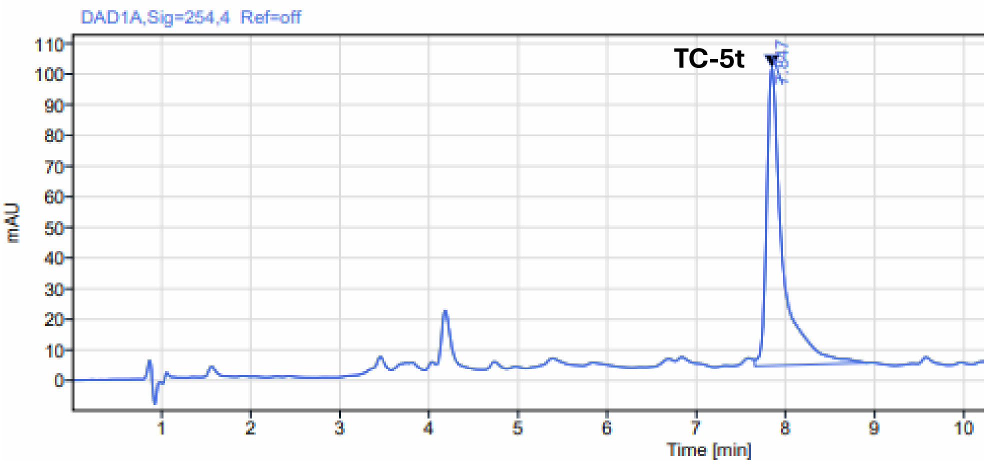

crude

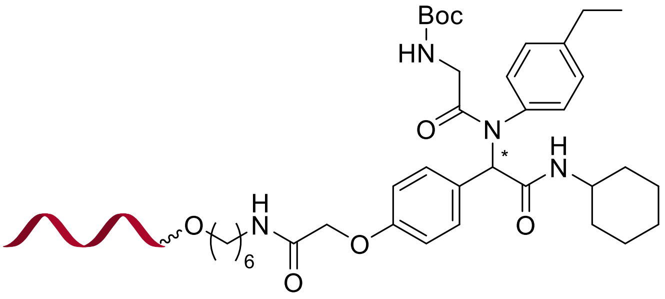

TC-5v

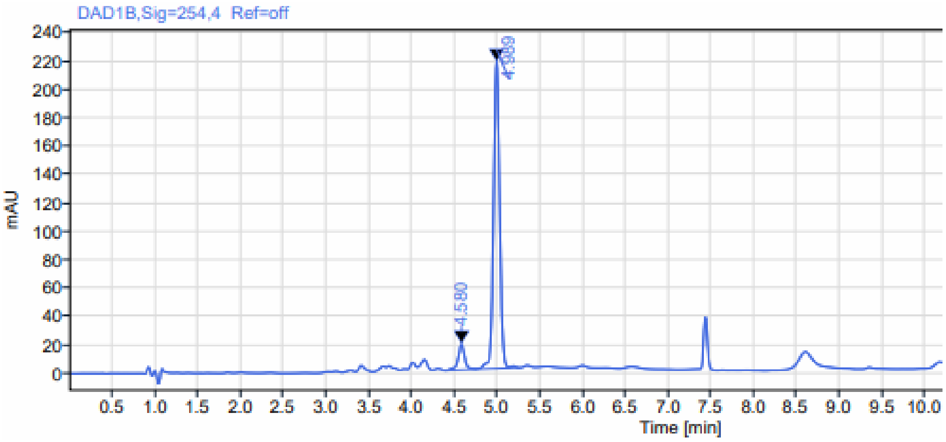

starting material

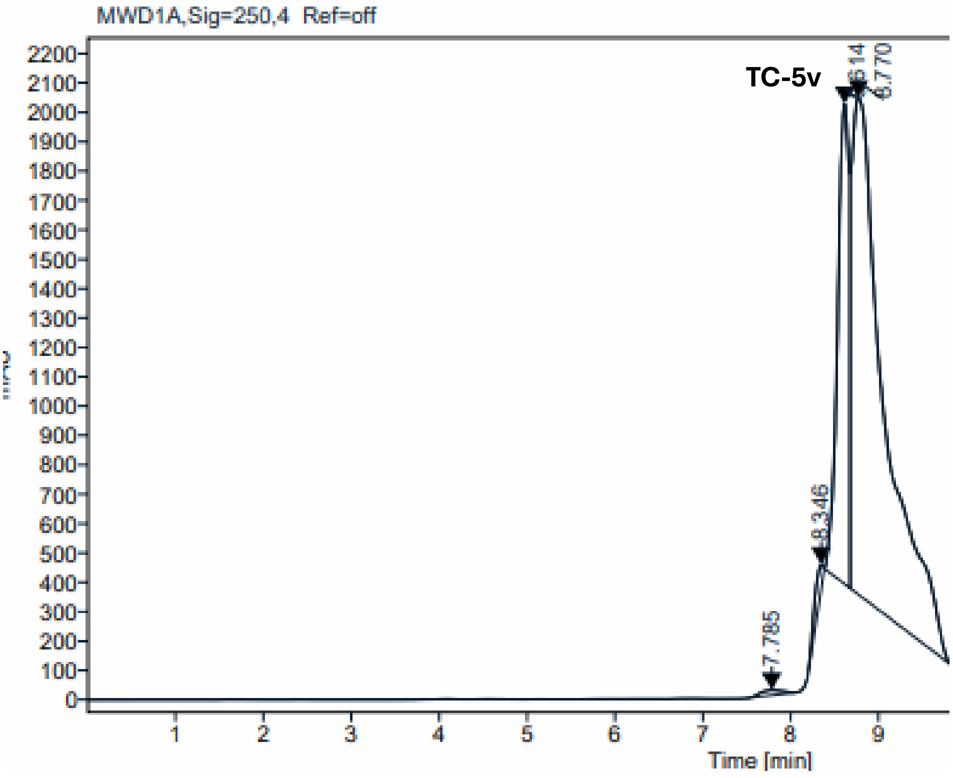

crude

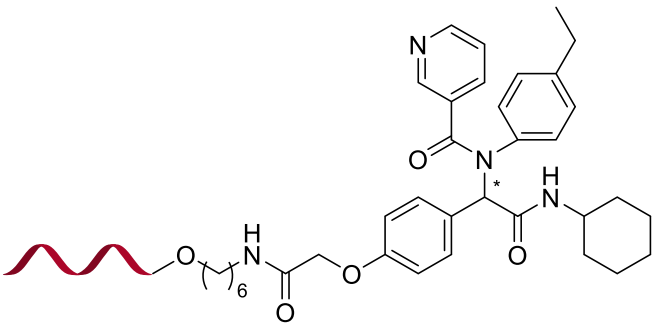

TC-5x

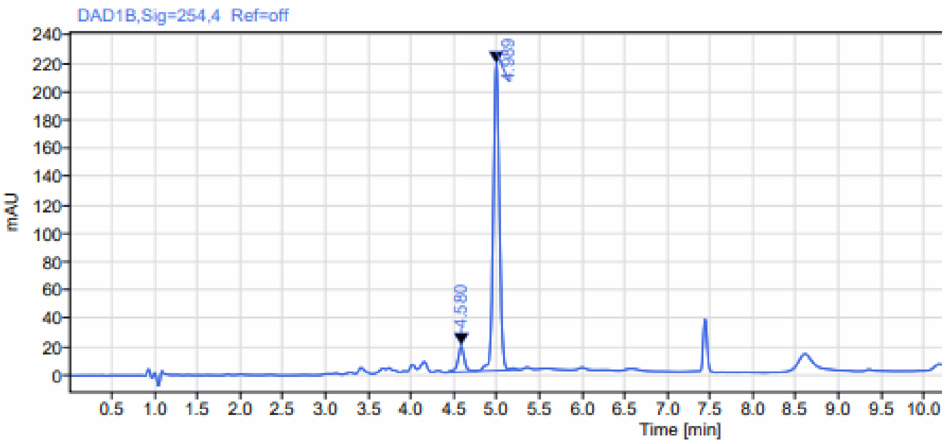

starting material

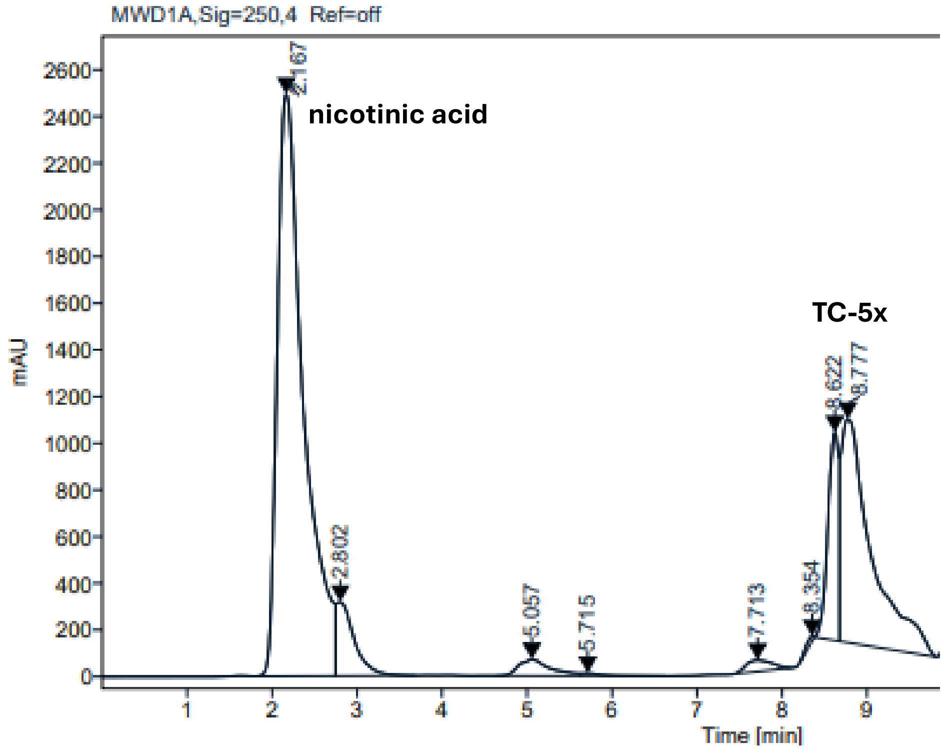

crude

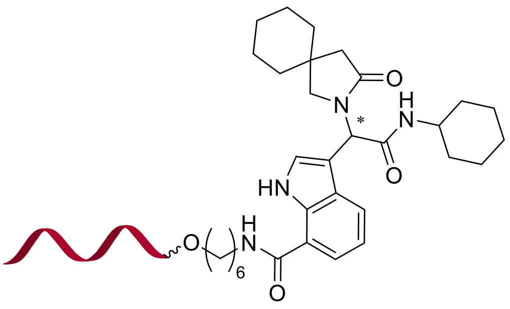

TC- 5ag

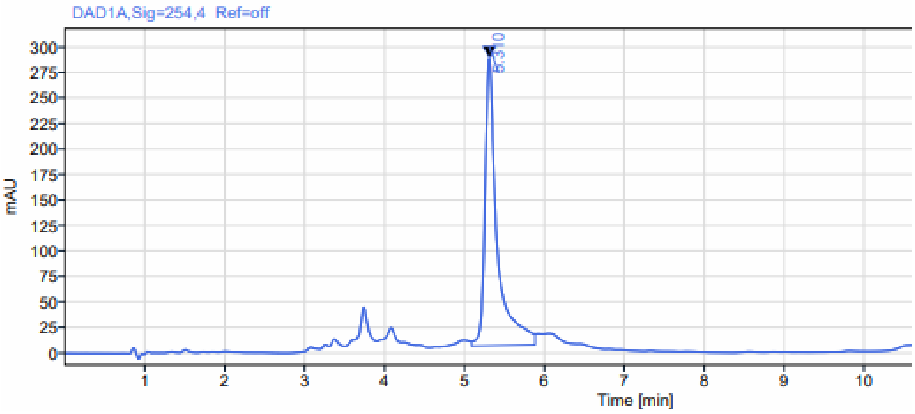

starting material

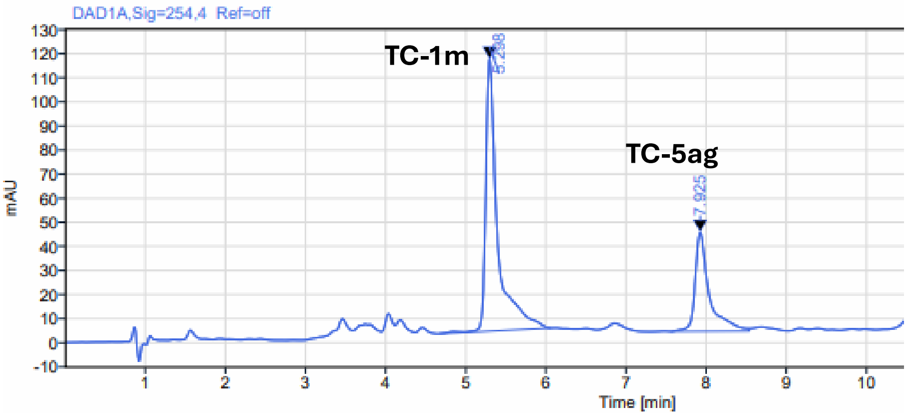

crude

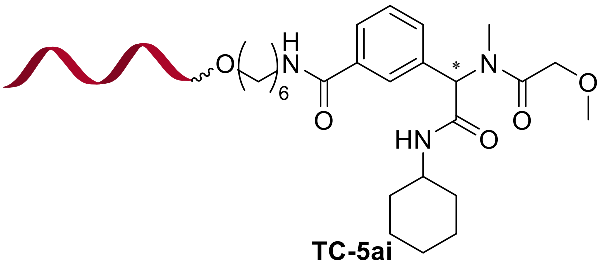

TC-5ai

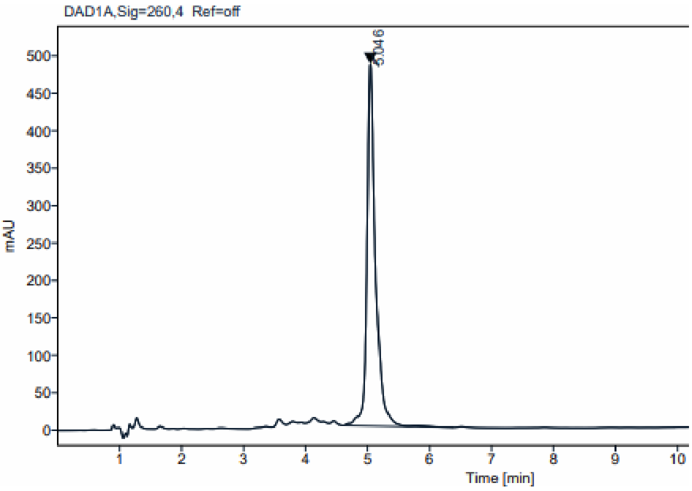

starting material

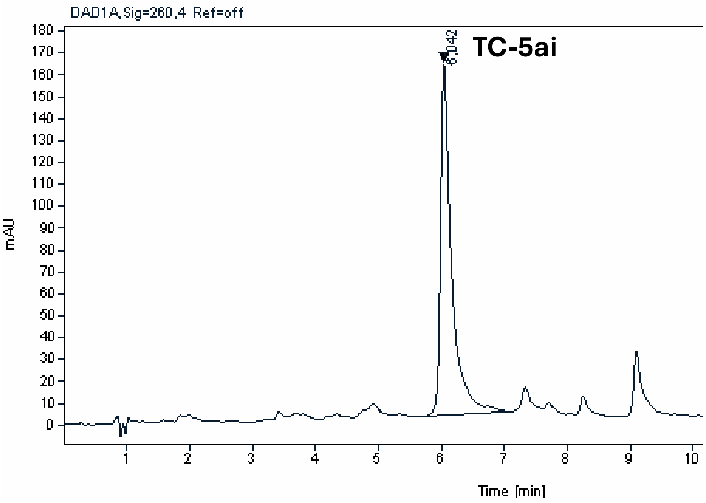

crude

Ugi-azide four-component reaction

Graphic presentation of the procedure

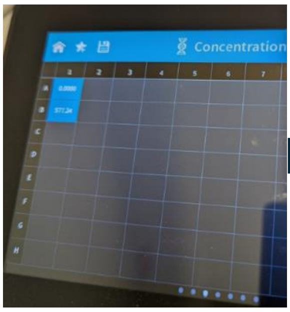

Nanodrop → measuring DNA concentration

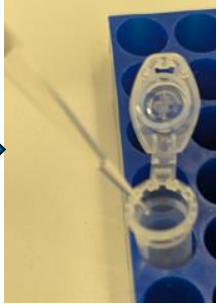

Pipetting 3 nmol DNA

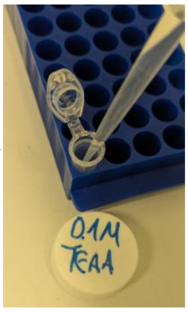

Addition of TEA as counterion

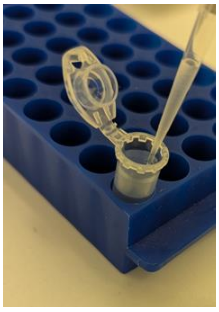

Addition of copolymer

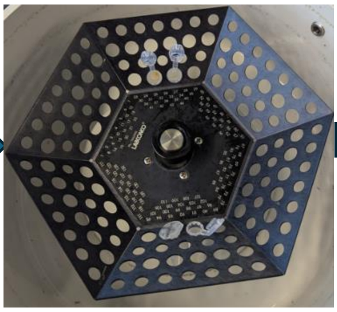

Solvent evaporation

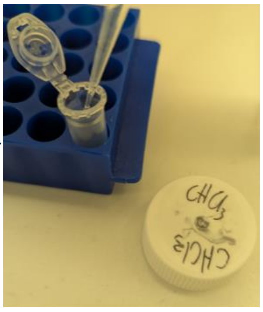

Addition of $CHCl_3$

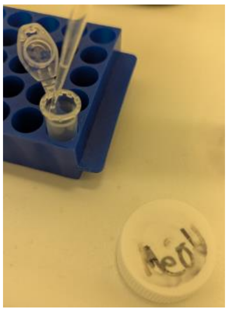

Addition of MeOH

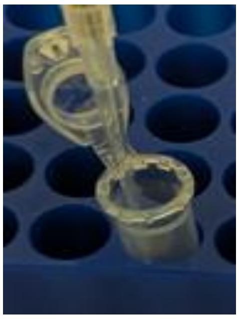

Addition of secondary amine

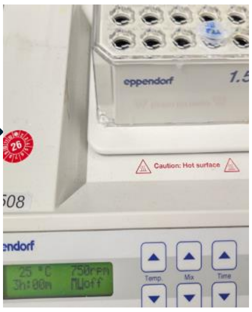

Imine formation

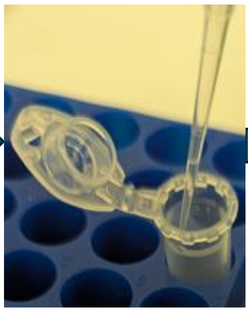

Addition of $TMSN_3$

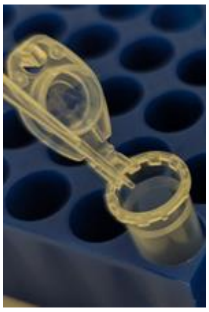

Addition of isocyanide

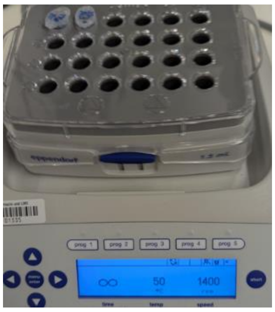

Shaking at 50°C for 16h (Table 2, entry 4)

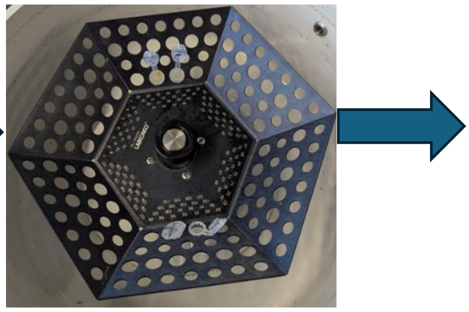

Solvent evaporation

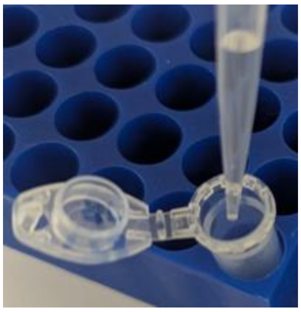

Addition of water for analysis & purification

Optimization of Ugi-azide four-component reaction

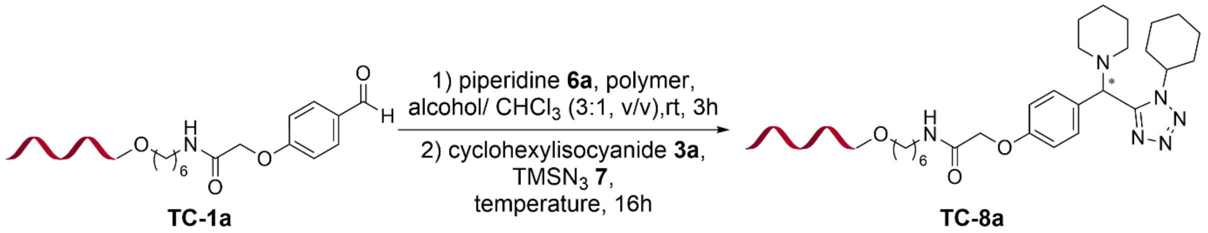

Table S6. Conversion rates for different reaction conditions for the Ugi-azide four-component reaction.

No.	Solvent[a]	equivalents	Concentration	temperature	catalyst	Product conversion
**1**	$MeOH/CHCl_3$	250	15 mM	50°C	/	61%
**2**	$MeOH/CHCl_3$	500	30 mM	50°C	/	70%
**3**	$MeOH/CHCl_3$	1000	60 mM	50°C	/	67%
**4**	$MeOH/CHCl_3$	2000	120 mM	50°C	/	90%
**5**	$MeOH/CHCl_3$	2000	120 mM	37°C	/	87%
**6**	$MeOH/CHCl_3$	2000	120 mM	rt[c]	/	67%
**7**	$MeOH/CHCl_3$	2000	120 mM	rt	$ZnCl_2$	51%
**8**	$MeOH/CHCl_3$	2000	120 mM	rt	$MgCl_2$	38%
**9**	$MeOH/CHCl_3$	2000	120 mM	rt	$FeCl_2$	n.d.
**10**	$EtOH/CHCl_3$	2000	120 mM	50°C	/	67%
**11**	$iPrOH/CHCl_3$	2000	120 mM	50°C	/	38%
**12**[b]	$CF_3CH_2OH/CHCl_3$	3000	180mM	60°C	/	25%

[a] alcohol/$CHCl_3$ (3:1, vol/vol); [b]all reactants were added simultaneously, and the reaction was run for 48h; [c]rt means 25°C

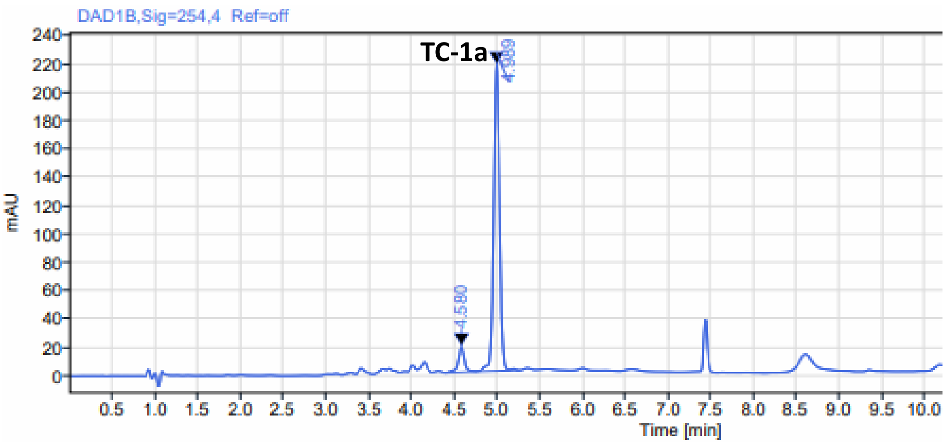

Figure S45. Analytical HPLC trace of starting material **TC-1a**.

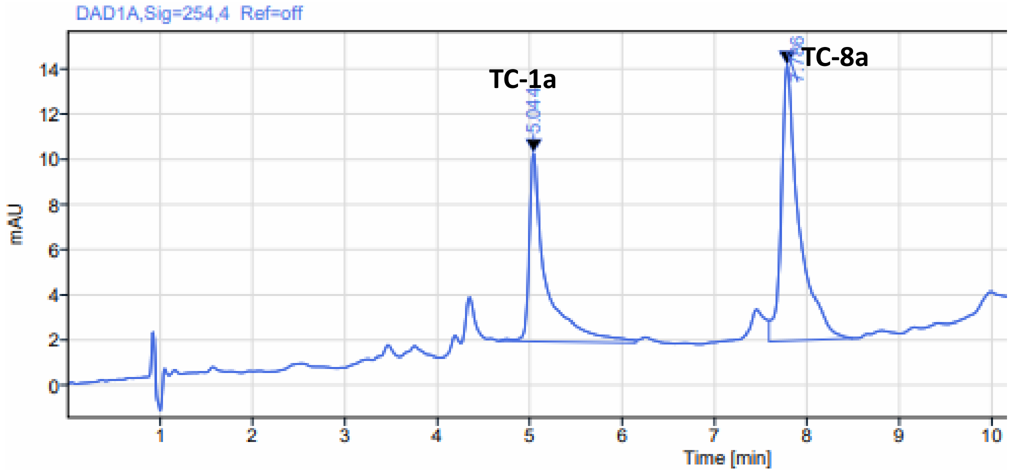

Figure S46. Crude analytical HPLC trace for product **TC-8a** with 250 equiv amine, 250 equiv isocyanide, 250 equiv azide in MeOH/$CHCl_3$ (3:1,v/v) at 50°C (entry 1).

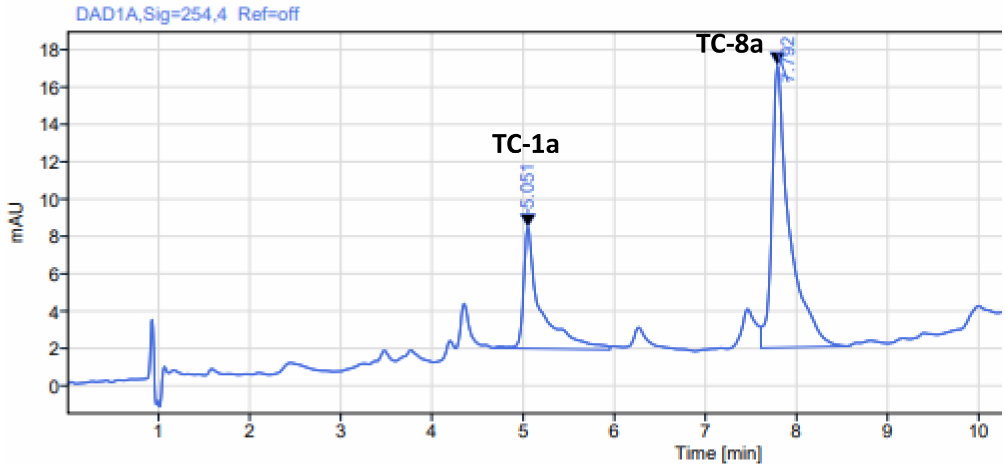

Figure S47. Crude analytical HPLC trace for product **TC-8a** with 500 equiv amine, 500 equiv isocyanide, 500 equiv azide in MeOH/$CHCl_3$ (3:1,v/v) at 50°C (entry 2).

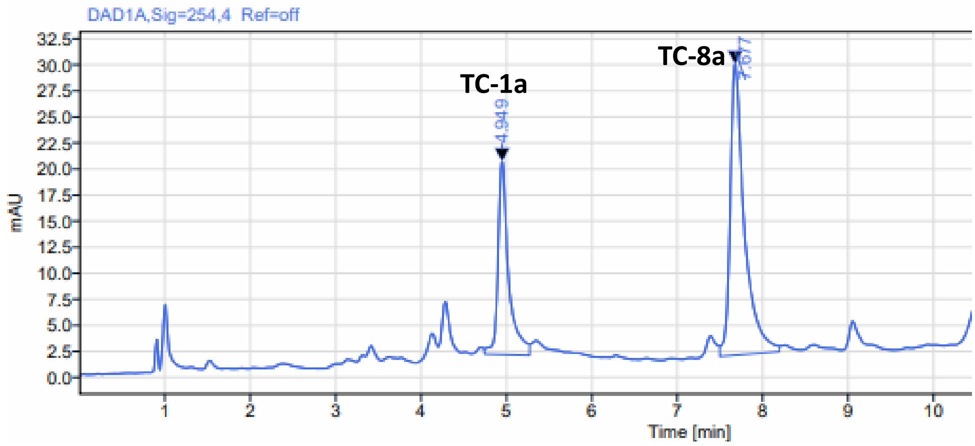

Figure S48. Crude analytical HPLC trace for product **TC-8a** with 1000 equiv amine, 1000 equiv isocyanide, 1000 equiv azide in MeOH/$CHCl_3$ (3:1,v/v) at 50°C (entry 3).

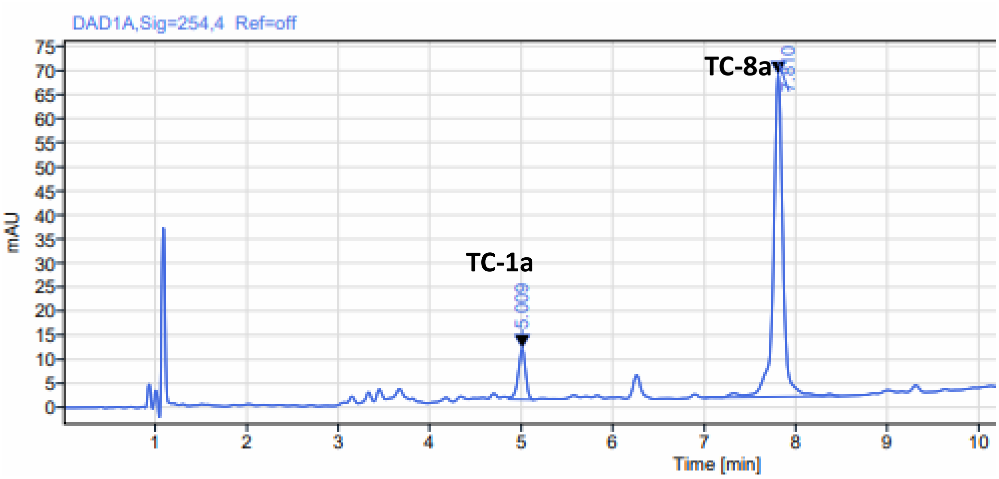

Figure S49. Crude analytical HPLC trace for product **TC-8a** with 2000 equiv amine, 2000 equiv isocyanide, 2000 equiv azide in MeOH/$CHCl_3$ (3:1,v/v) at 50°C (entry 4).

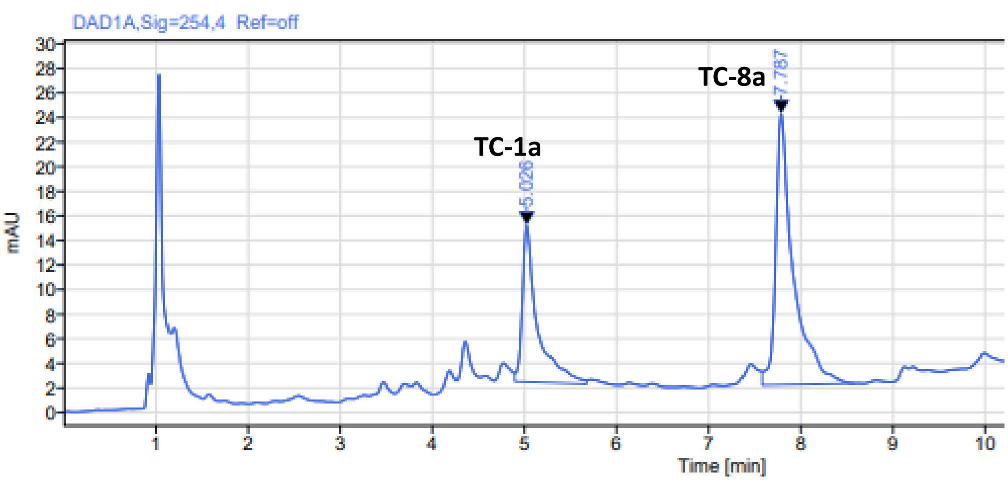

Figure S50. Crude analytical HPLC trace for product **TC-8a** with 2000 equiv amine, 2000 equiv isocyanide, 2000 equiv azide in MeOH/$CHCl_3$ (3:1,v/v) at 37°C (entry 5).

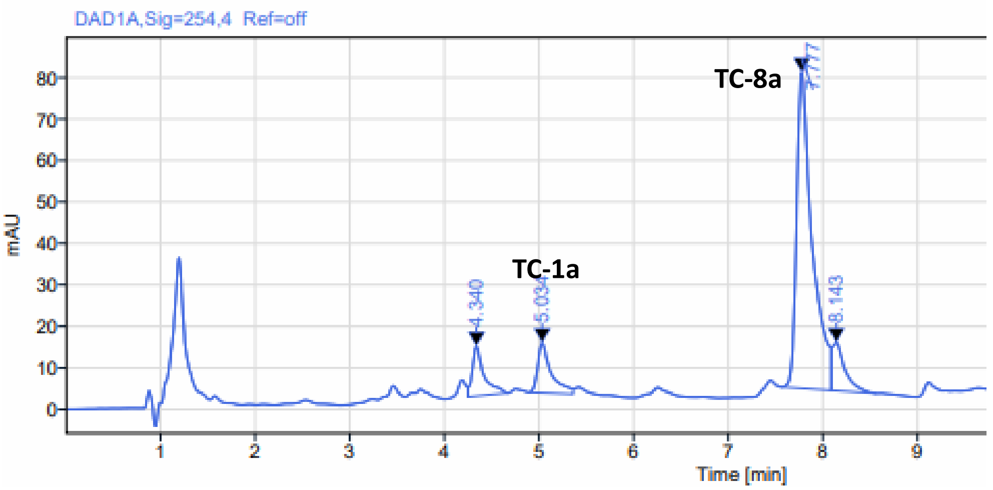

Figure S51. Crude analytical HPLC trace for product **TC-8a** with 2000 equiv amine, 2000 equiv isocyanide, 2000 equiv azide in MeOH/$CHCl_3$ (3:1,v/v) at rt (entry 6).

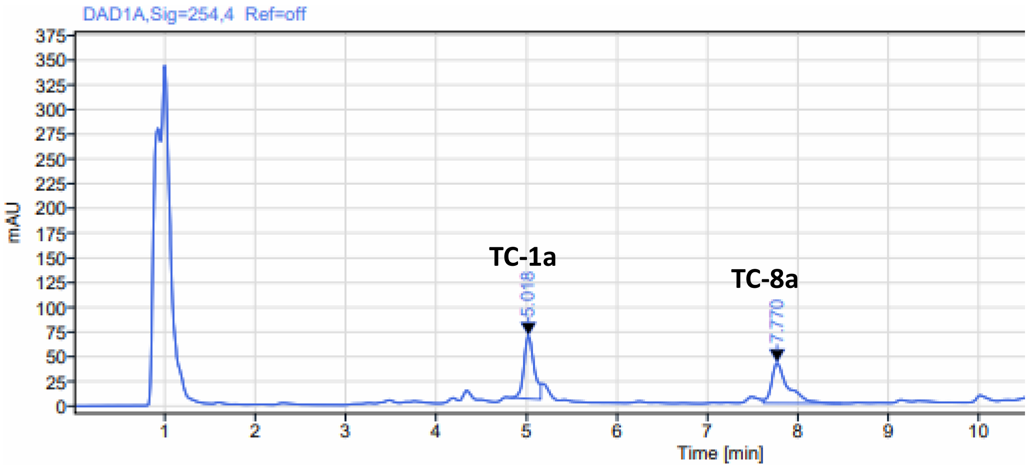

Figure S52. Crude analytical HPLC trace for product **TC-8a** with 2000 equiv amine, 2000 equiv isocyanide, 2000 equiv azide in MeOH/$CHCl_3$ (3:1,v/v) at rt with $ZnCl_2$ as catalyst (entry 7).

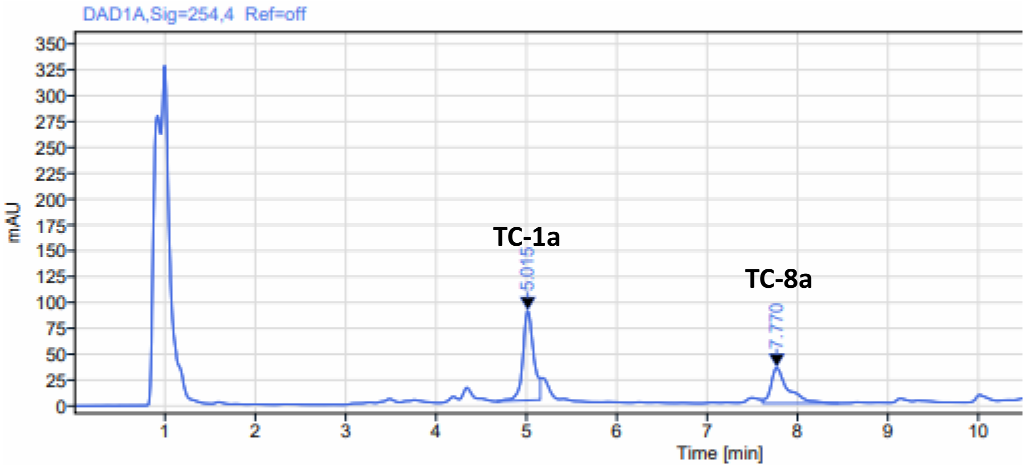

Figure S53. Crude analytical HPLC trace for product **TC-8a** with 2000 equiv amine, 2000 equiv isocyanide, 2000 equiv azide in MeOH/$CHCl_3$ (3:1,v/v) at rt with $MgCl_2$ as catalyst (entry 8).

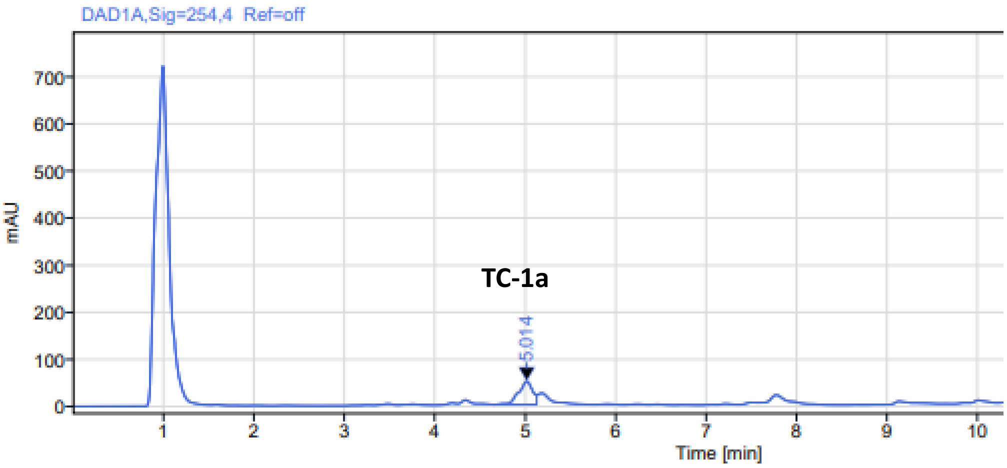

Figure S54. Crude analytical HPLC trace for product **TC-8a** with 2000 equiv amine, 2000 equiv isocyanide, 2000 equiv azide in MeOH/$CHCl_3$ (3:1,v/v) at rt with $FeCl_2$ as catalyst (entry 9).

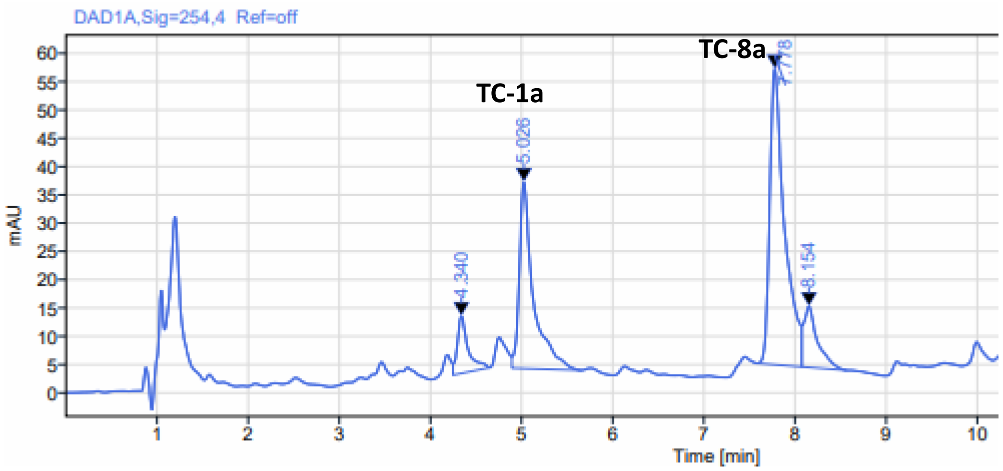

Figure S55. Crude analytical HPLC trace for product **TC-8a** with 2000 equiv amine, 2000 equiv isocyanide, 2000 equiv azide in EtOH/$CHCl_3$ (3:1,v/v) at 50°C (entry 10).

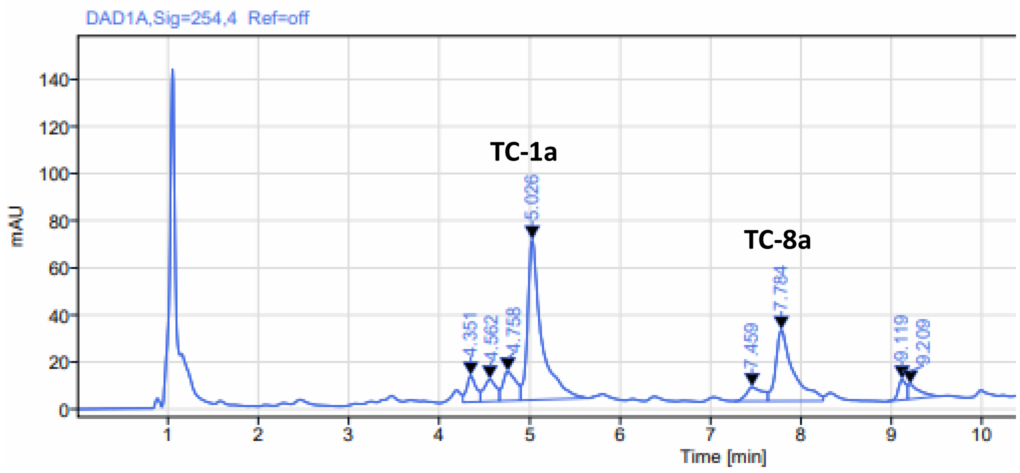

Figure S56. Crude analytical HPLC trace for product **TC-8a** with 2000 equiv amine, 2000 equiv isocyanide, 2000 equiv azide in iPrOH/$CHCl_3$ (3:1,v/v) at 50°C (entry 11).

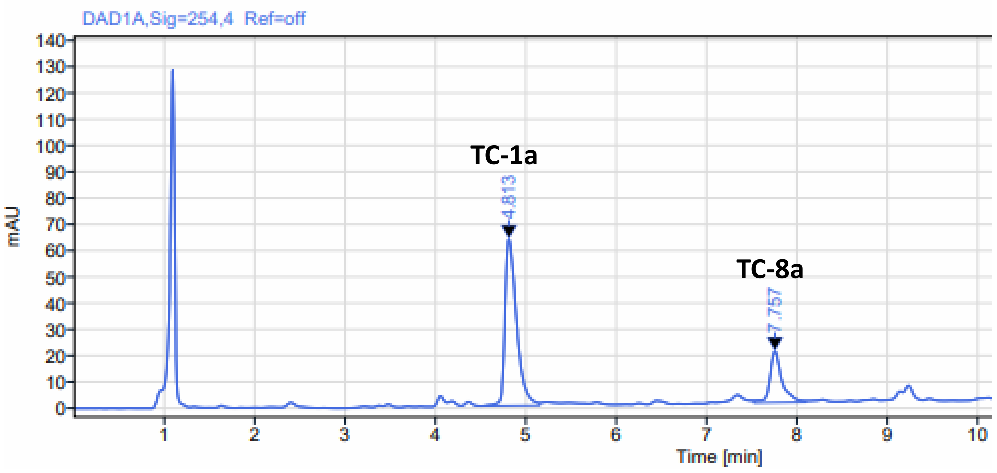

Figure S57. Crude analytical HPLC trace for product **TC-8a** with 3000 equiv amine, 3000 equiv isocyanide, 3000 equiv acid in CF_3CH_2OH /$CHCl_3$ (3:1,v/v) at 60°c for 2d (entry 12).

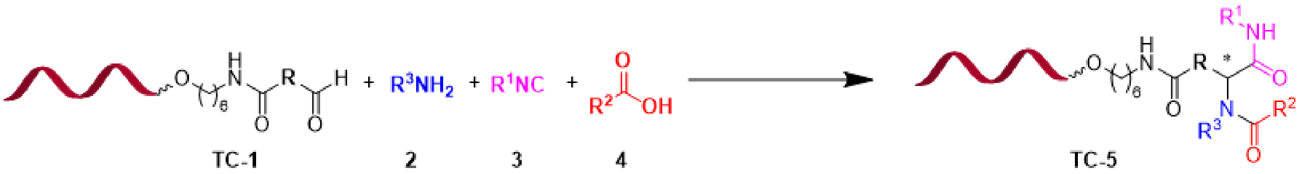

Table S7. MALDI-MS data & product conversions of the reaction scope for the Ugi-azide four-component reaction with 10mer TC-coupled starting materials.

				MALDI-MS m/z		Product conversion
No.	R	R^1	R^2	calculated	found	
TC-8a	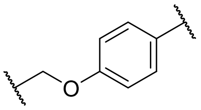	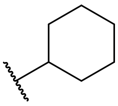	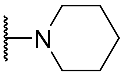	3465.0	3465.4	90%
TC-8b	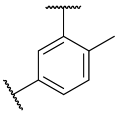	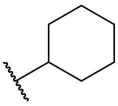	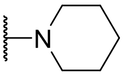	3450.0	3451.3	>95%
TC-8c	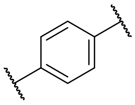	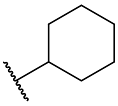	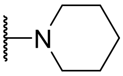	3435.0	3437.1	>95%
TC-8d	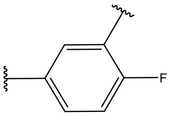	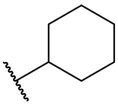	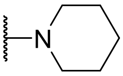	3453.0	3454.8	>95%
TC-8e	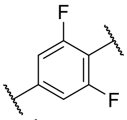	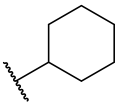	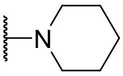	3471.0	3472.7	76%
TC-8f	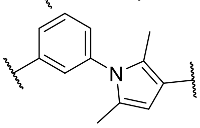	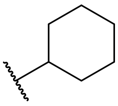	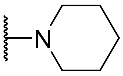	3530.0	3528.4	>95%
TC-8g	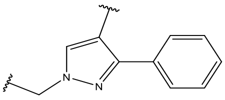	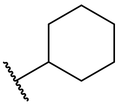	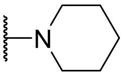	3515.0	3517.8	91%
TC-8h	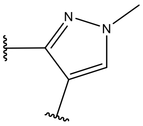	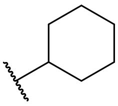	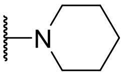	3439.0	3440.7	>95%
TC-8i	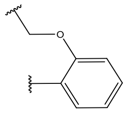	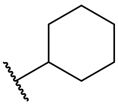	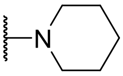	3465.0	3466.6	89%
TC-8j	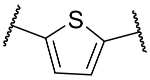	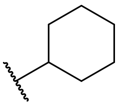	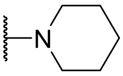	3441.0	3442.6	>95%

TC-8k	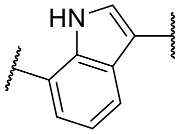	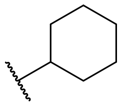	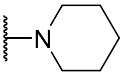	3474.0	3515.6 [M+CH_3COO^-]	85%
TC-8l	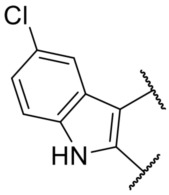	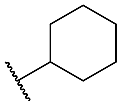	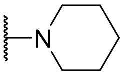	3508.0	3510.0	67%
TC-8m	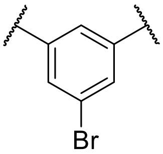	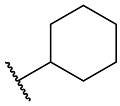	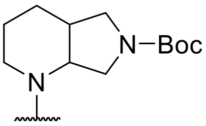	3656.0	3657.8	82%
TC-8n	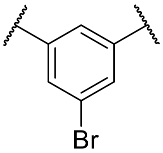	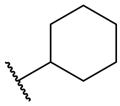	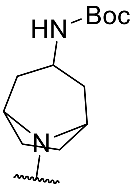	3656.0	3657.8	61%
TC-8o	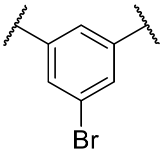	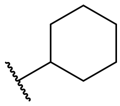	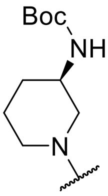	3630.0	3631.0	88%
TC-8p	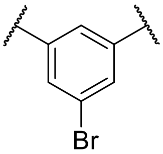	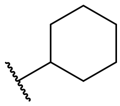	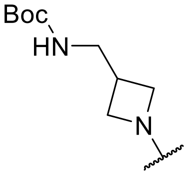	3616.0	3617.7	95%
TC-8q	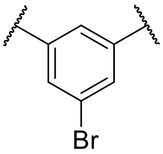	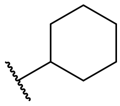	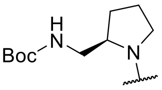	3629.0	3631.9	69%
TC-8r	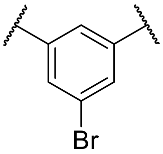	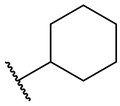	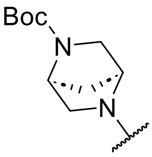	3627.0	3629.8	95%
TC-8s	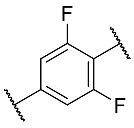	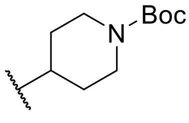	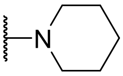	3571.0	3471.8 [M-Boc]	76%
TC-8t	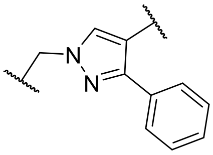	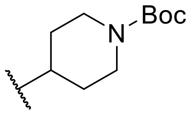	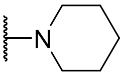	3615.0	3521.0 [M-Boc]	73%

TC-8u	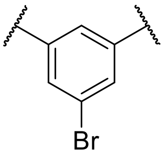	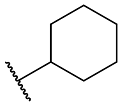	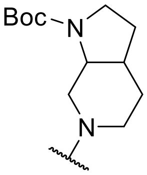	3655.0	3657.0	93%
TC-8v	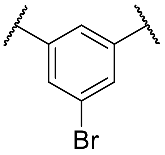	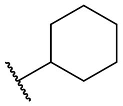	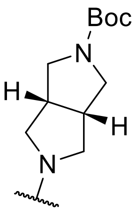	3641.0	3643.9	>95%

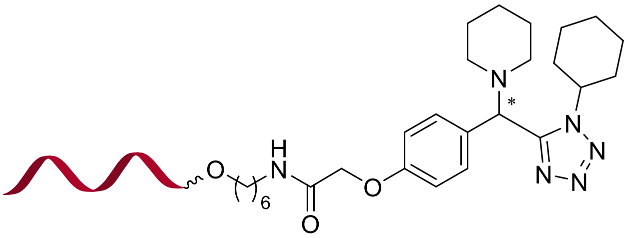

TC-8a

MS calc. 3465.0; found: 3465.4

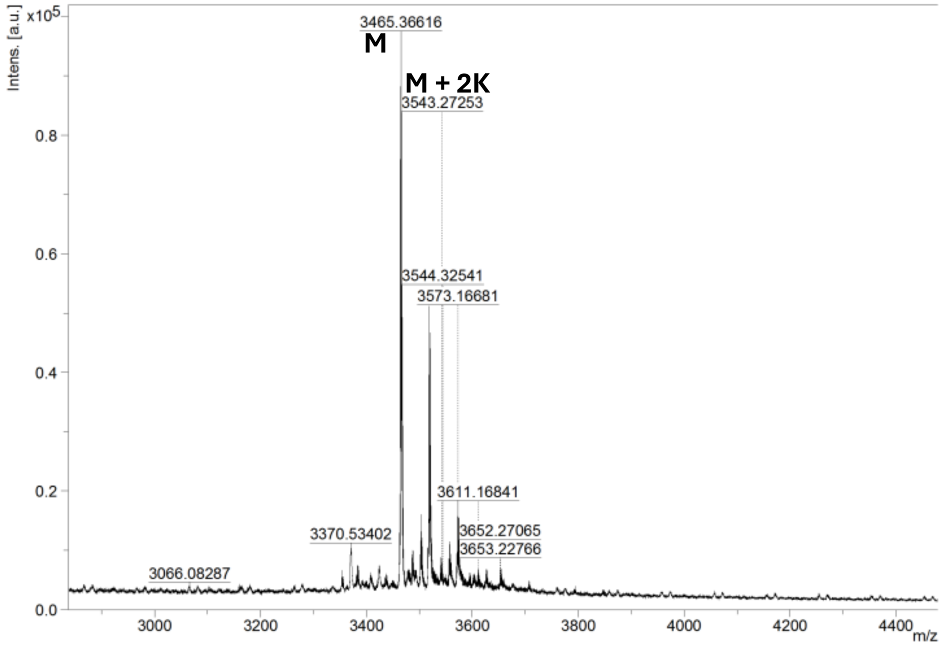

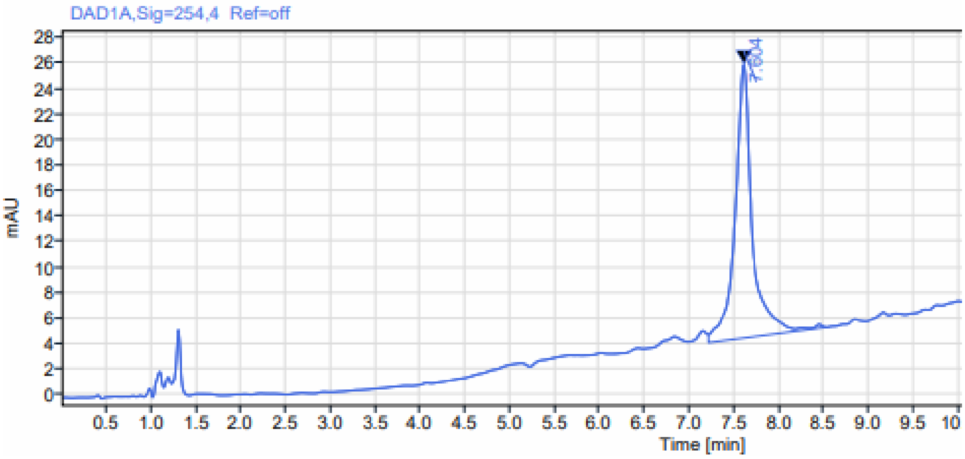

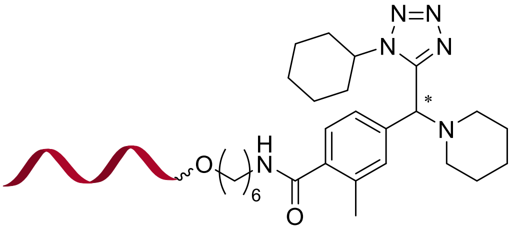

TC-8b

MS calc. 3450.0; found: 3451.3

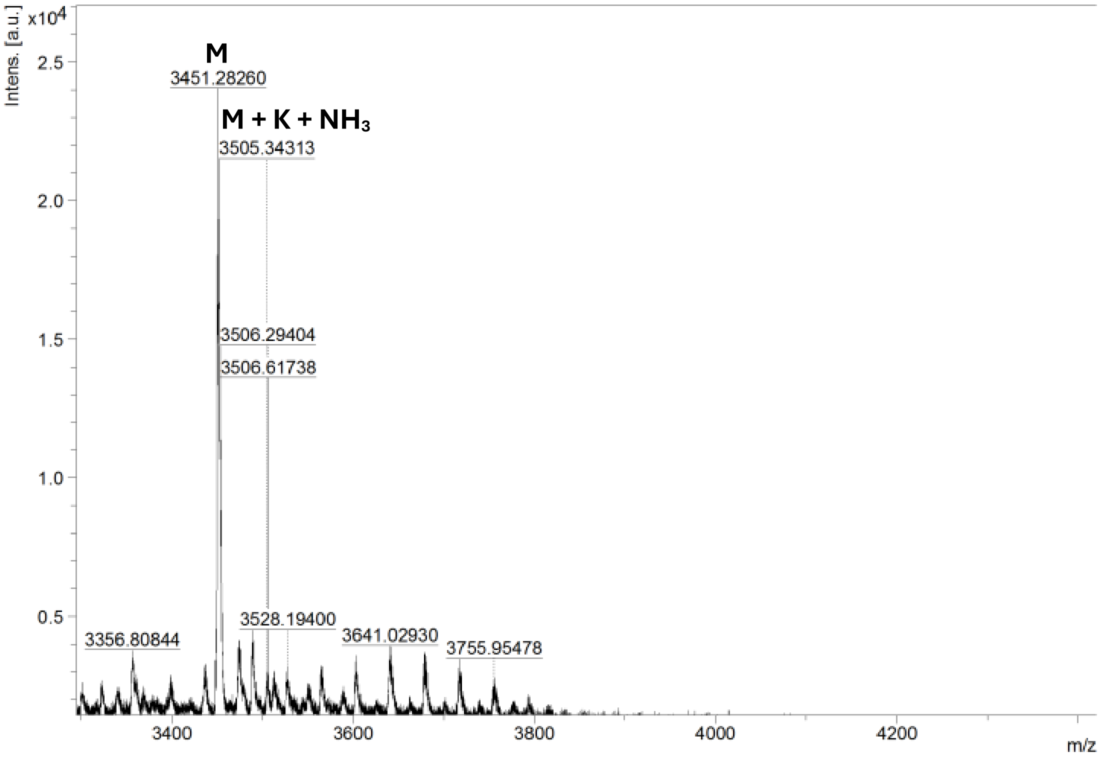

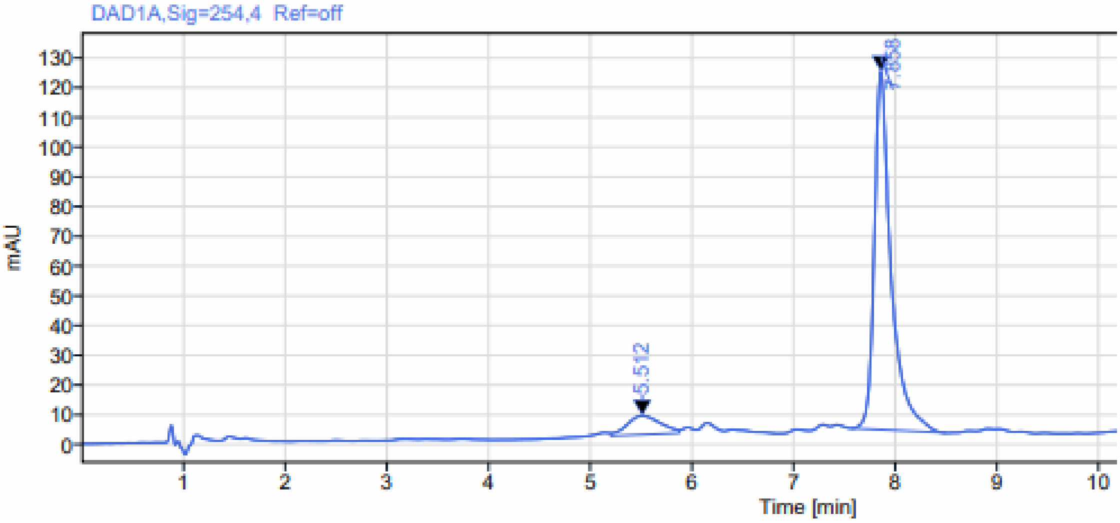

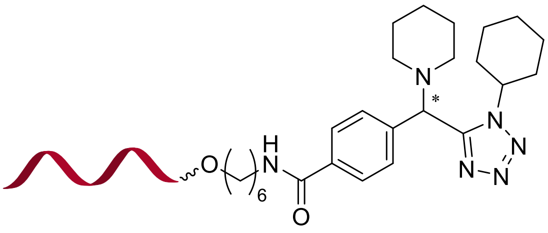

TC-8c

MS calc. 3435.0; found: 3437.1

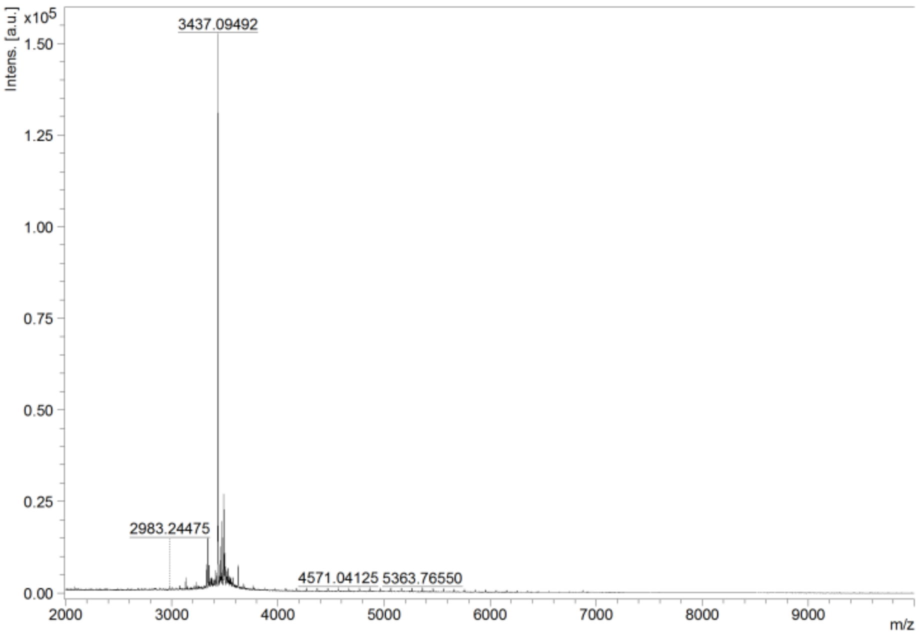

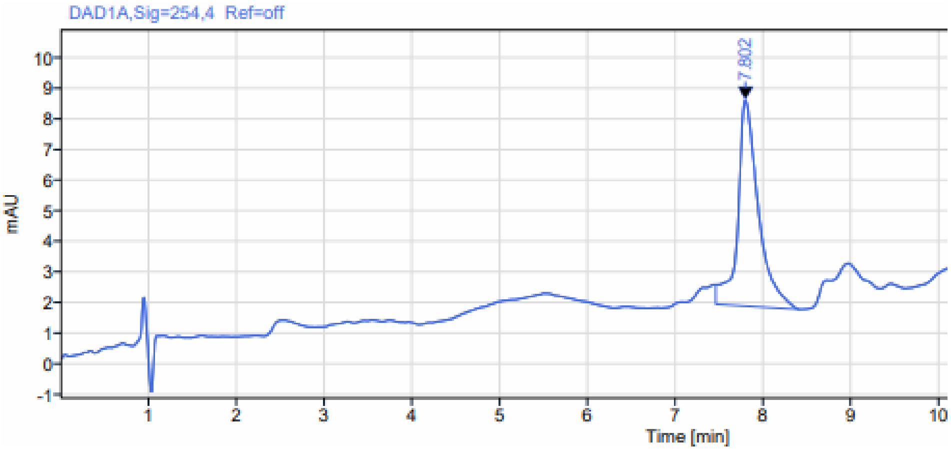

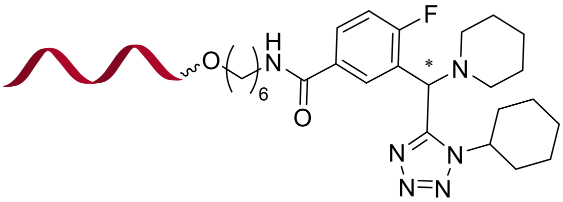

TC-8d

MS calc. 3453.0; found: 3454.7

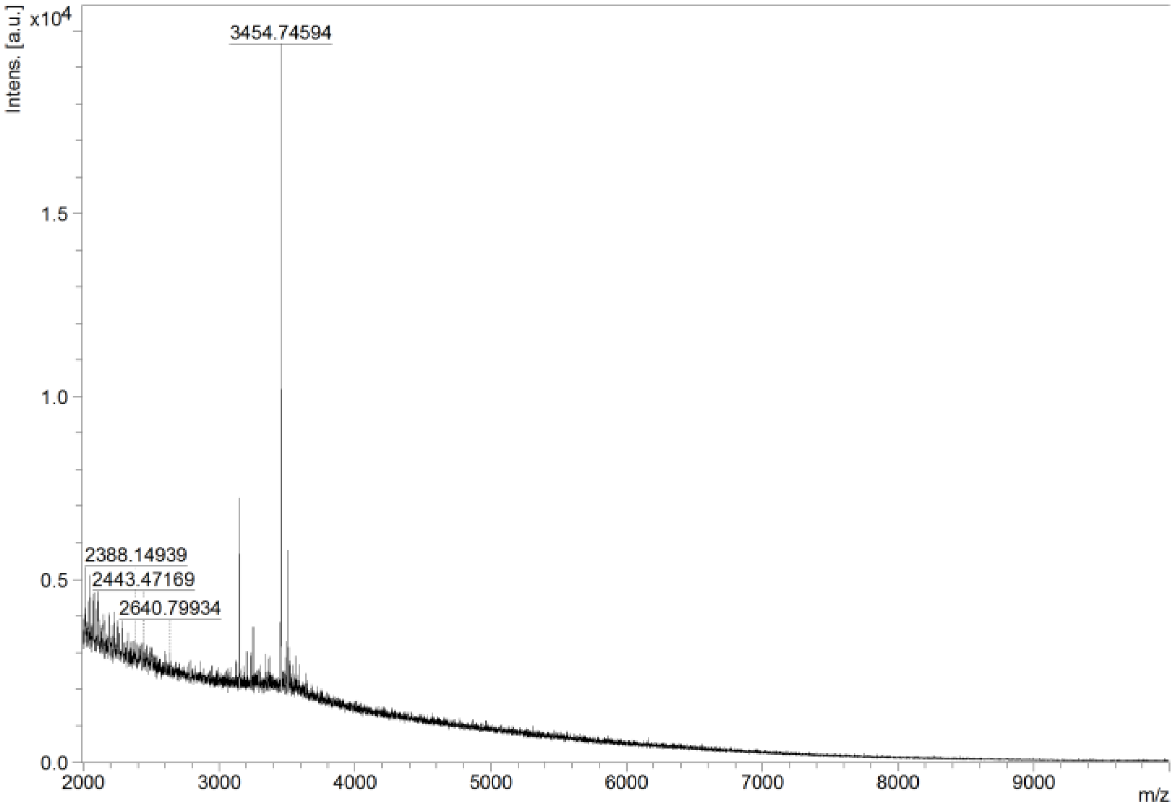

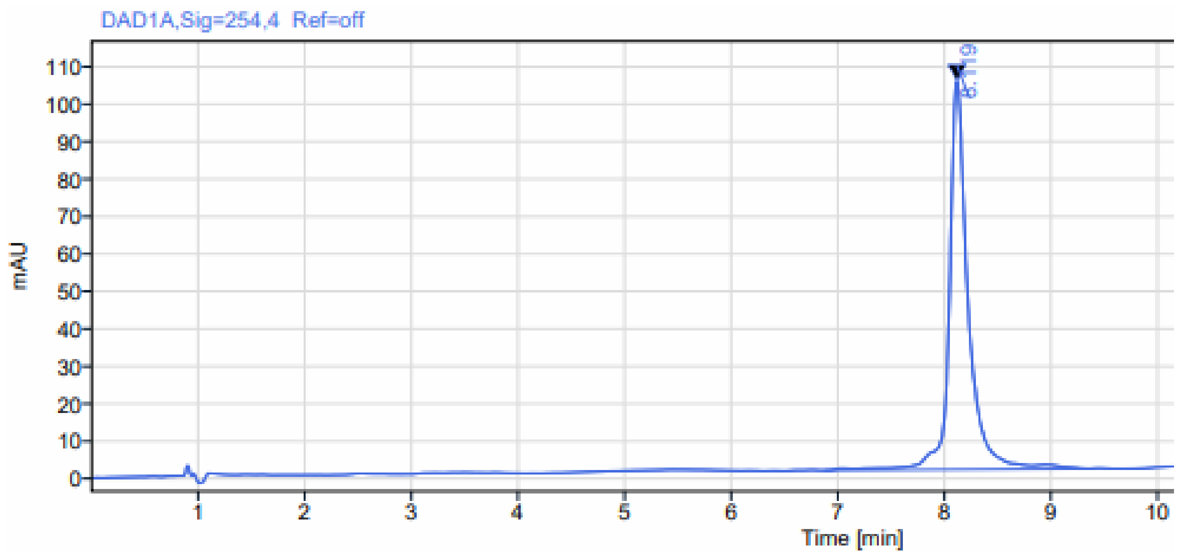

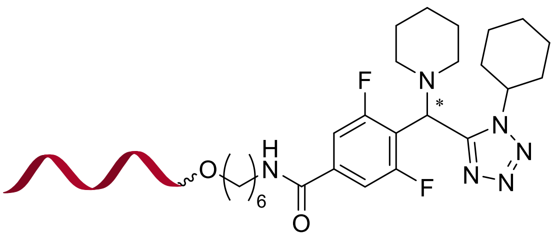

TC-8e

MS calc. 3471.0; found: 3472.7

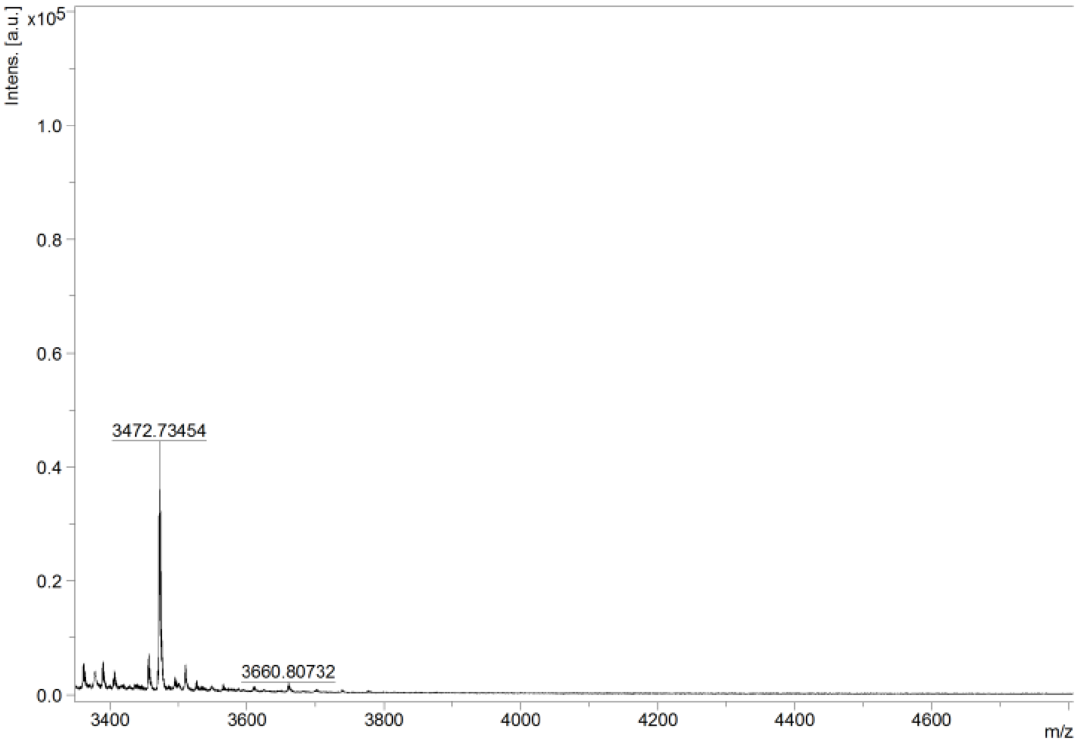

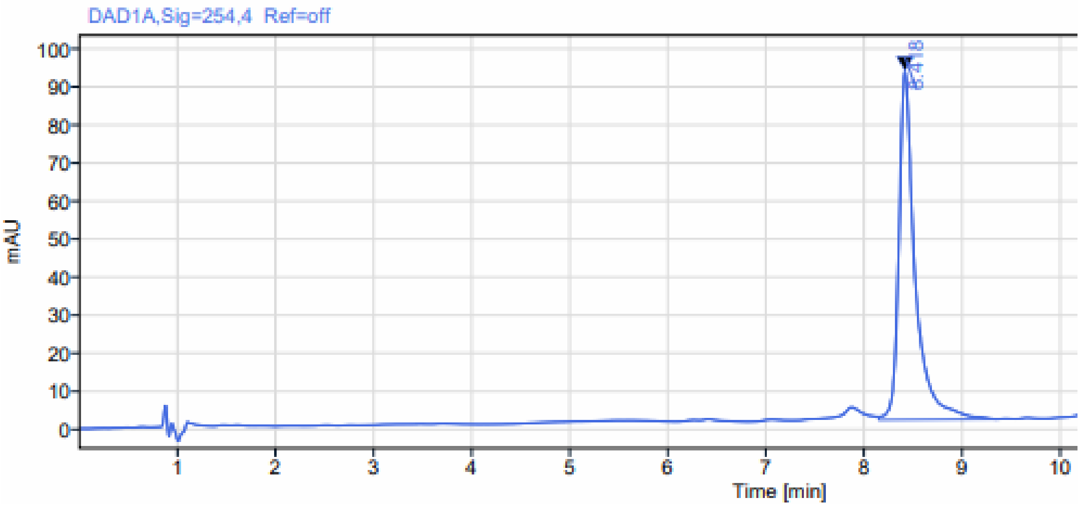

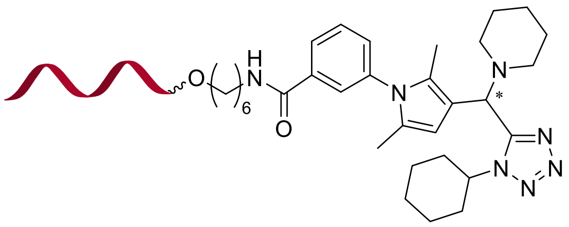

TC-8f

MS calc. 3530.0; found: 3628.4

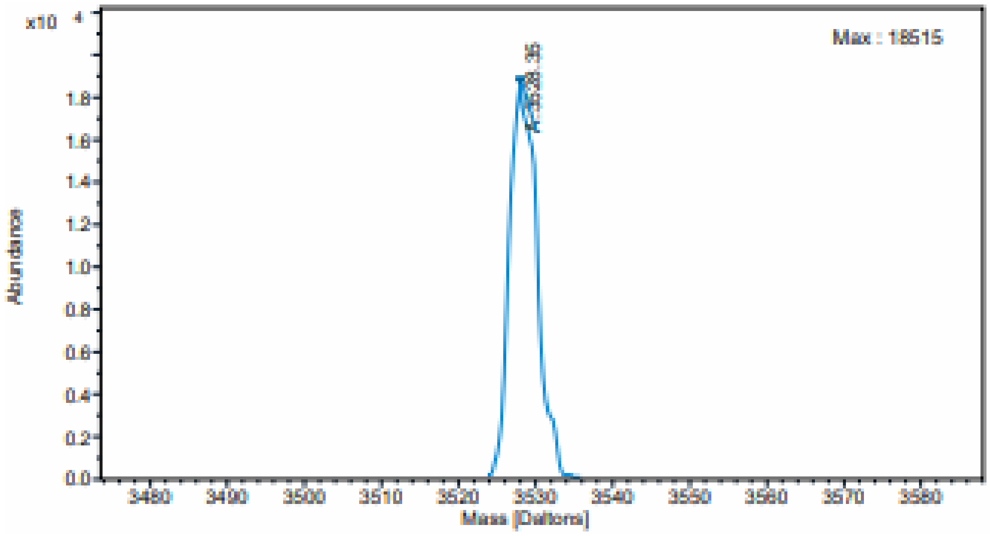

measured by LC-MS

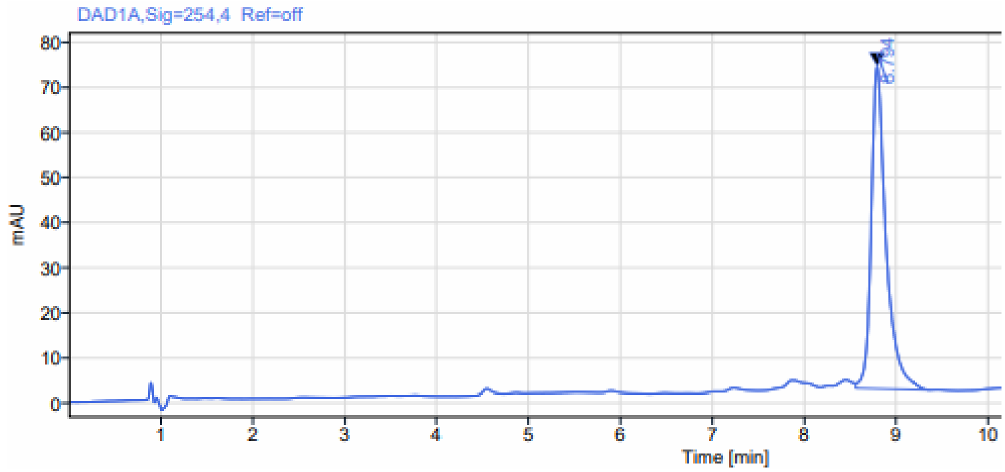

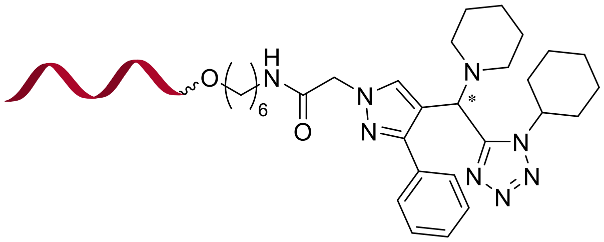

TC-8g

MS calc. 3515.0; found: 3516.8

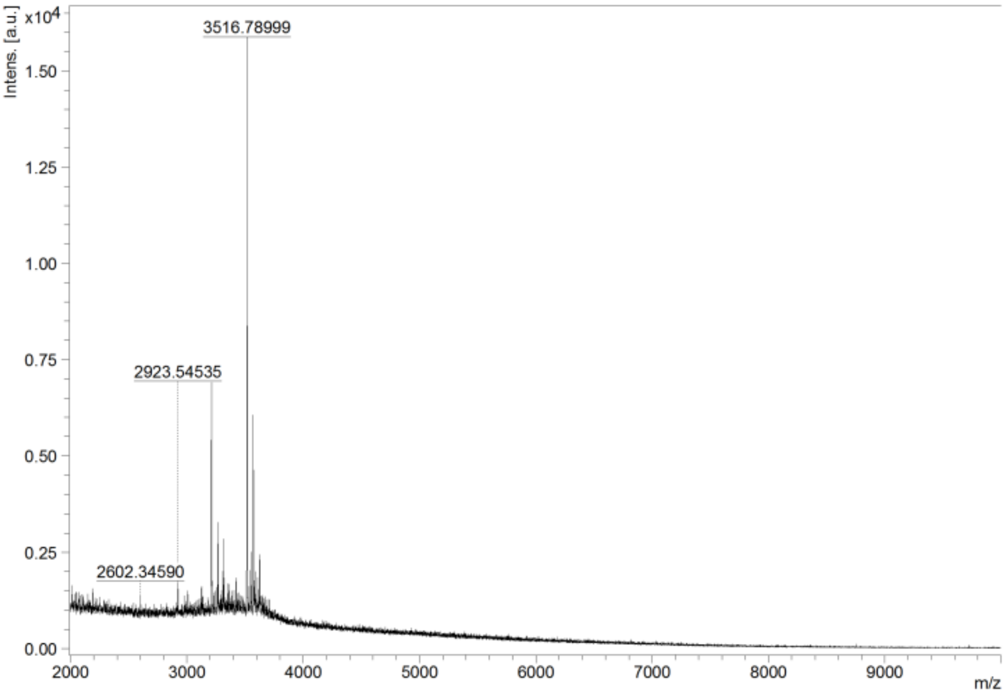

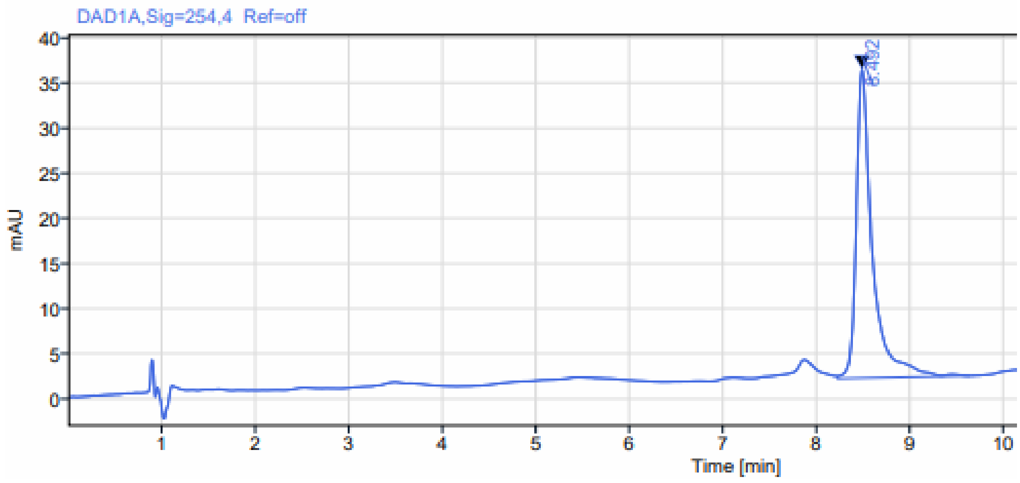

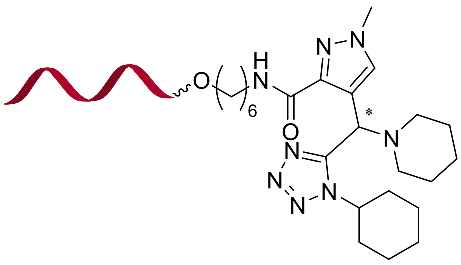

TC-8h

MS calc.3439.0 ; found: 3440.7

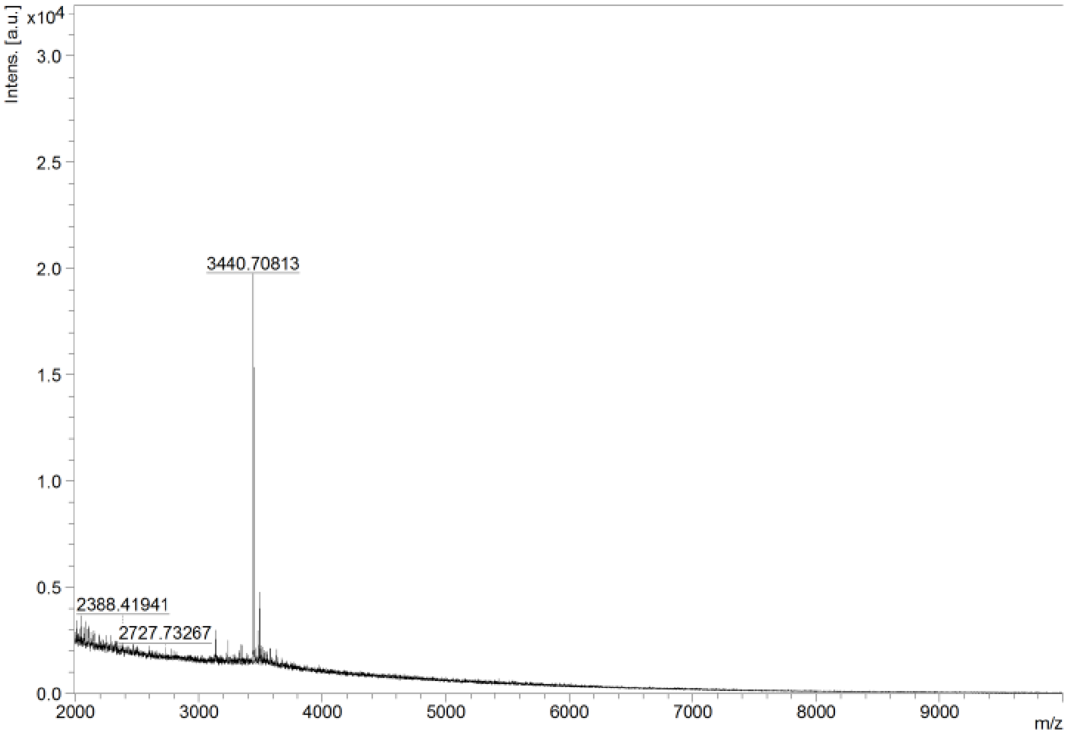

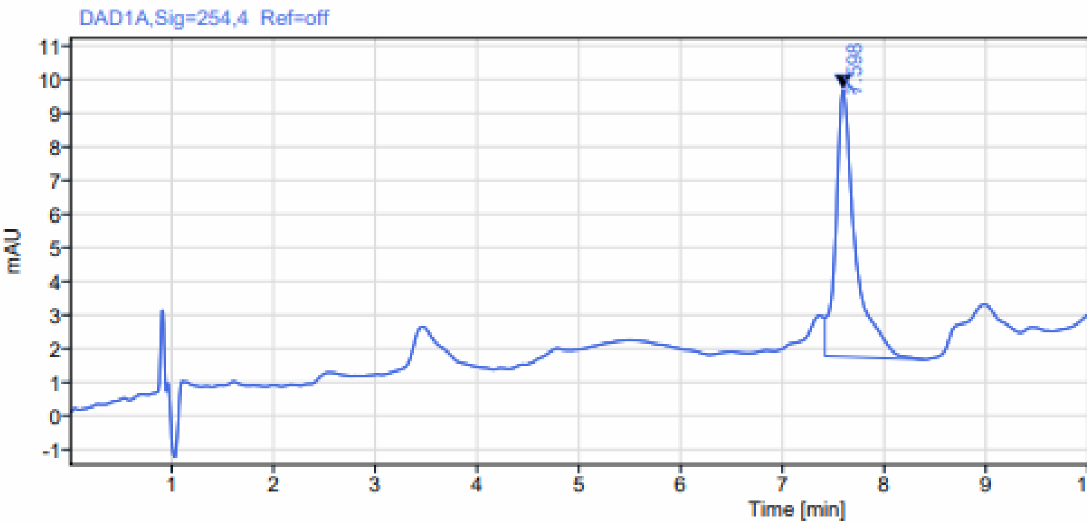

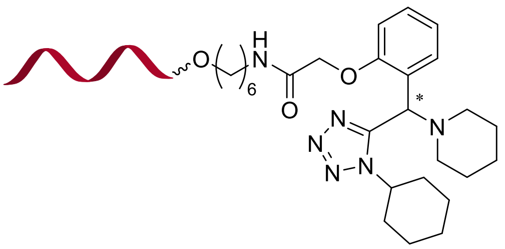

TC-8i

MS calc. 3465.0; found: 3466.5

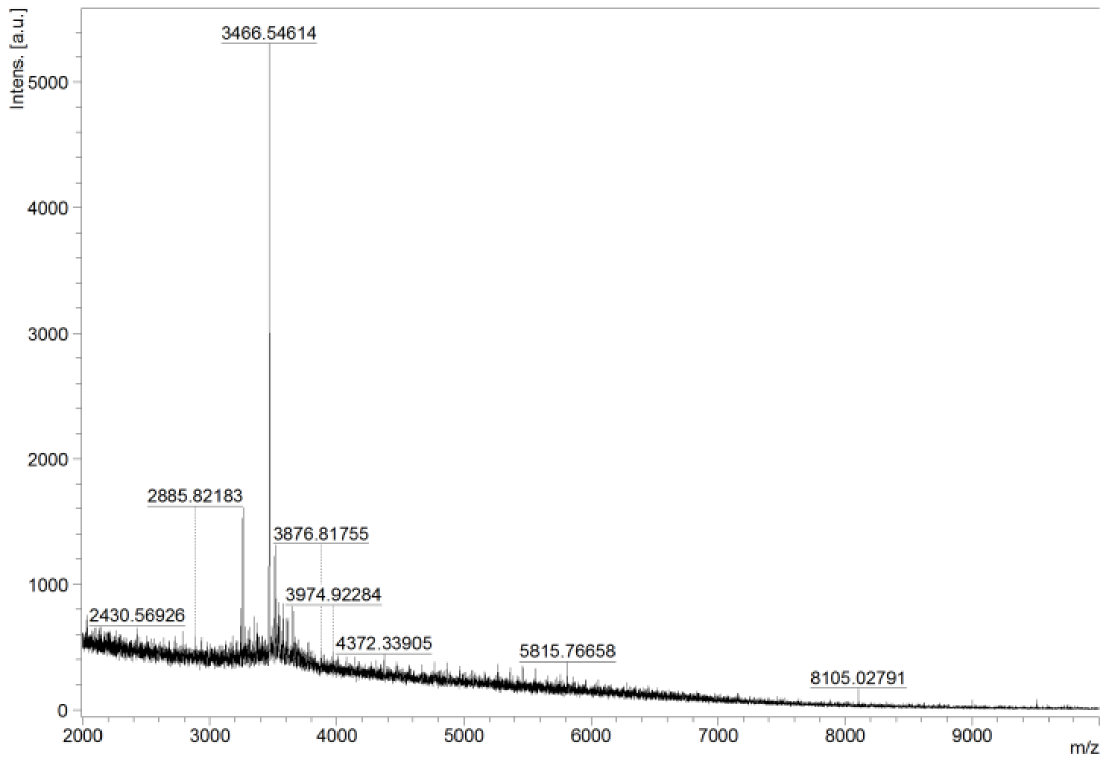

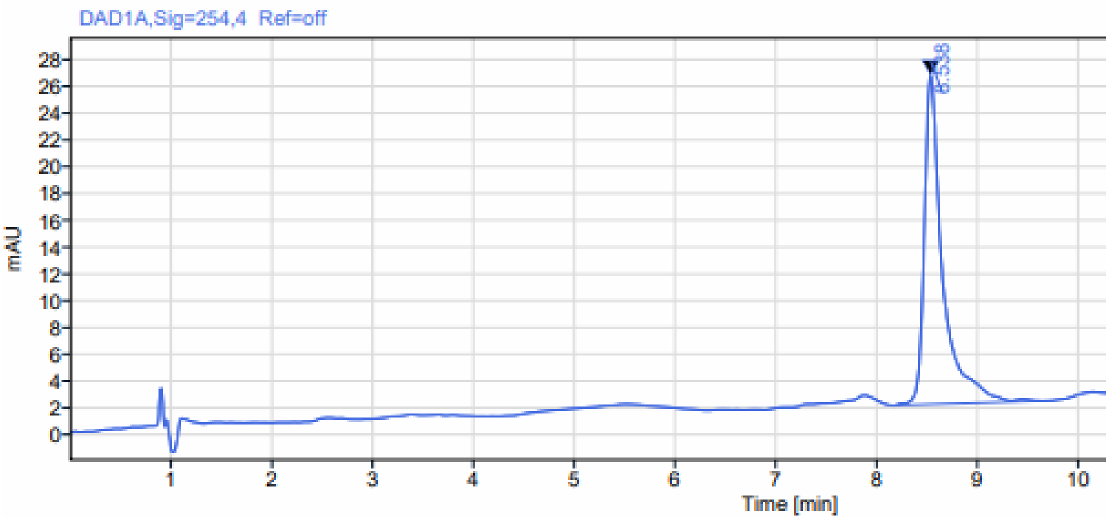

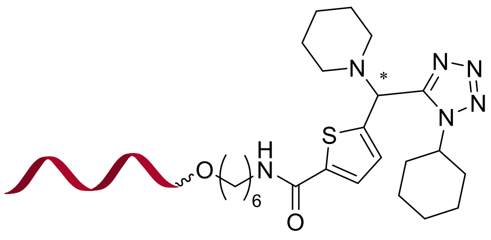

TC-8j

MS calc. 3441.0; found: 3442.6

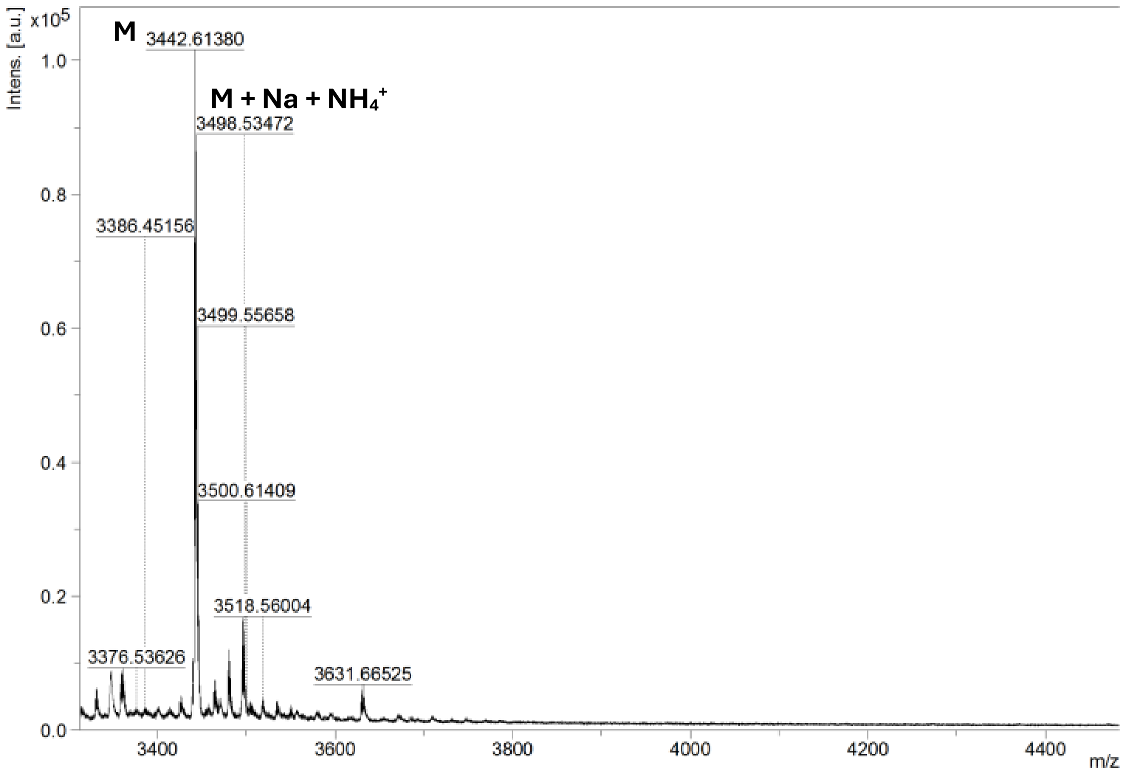

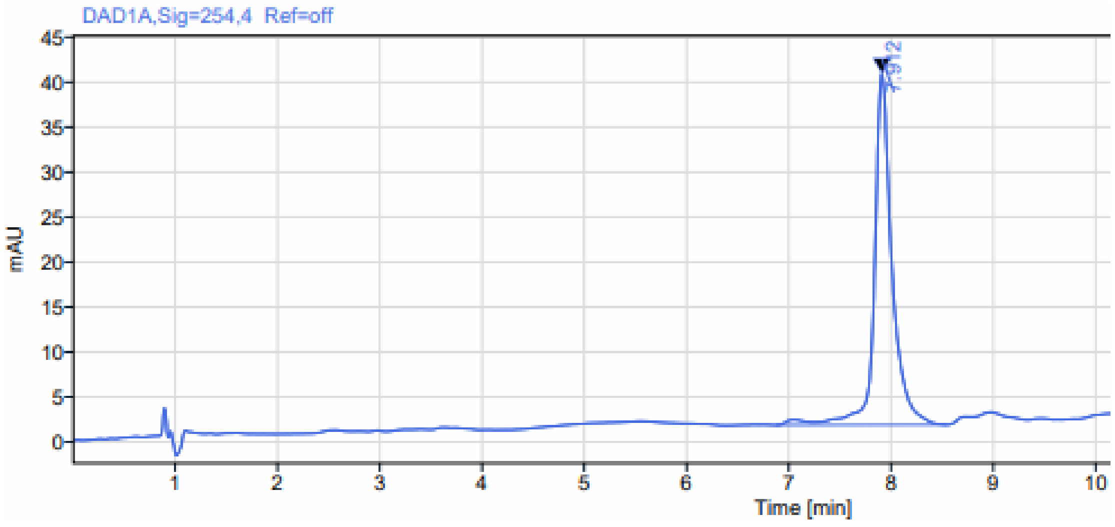

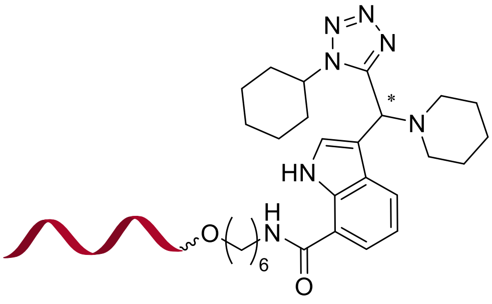

TC-8k

MS calc. 3474.0; found: 3515.6 [M+CH_3COO^-]

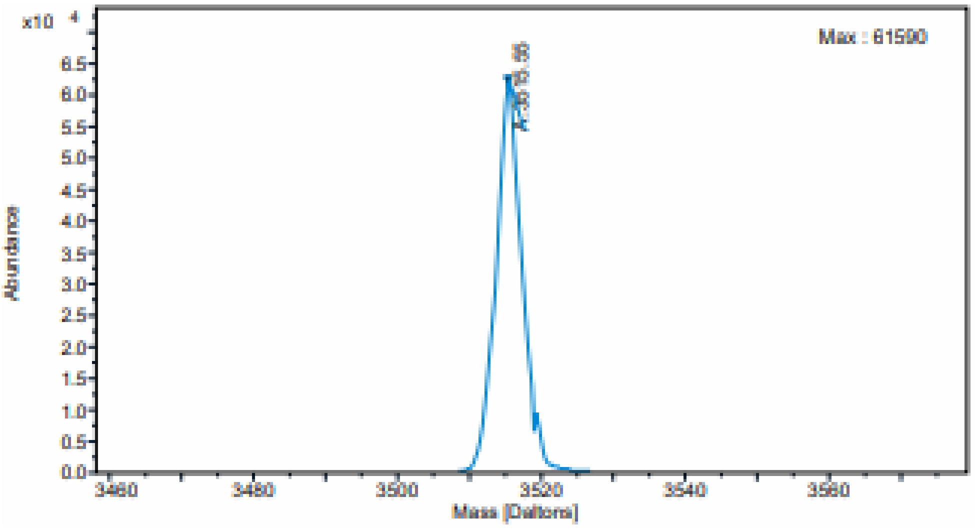

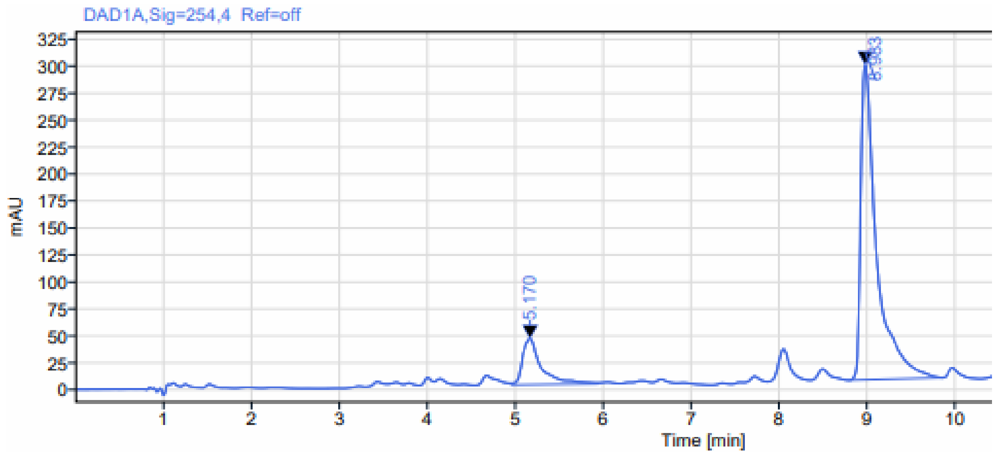

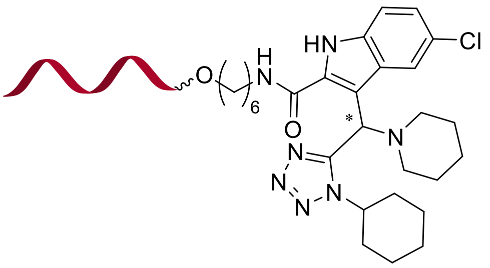

TC-8I

MS calc. 3508.0, found: 3510.0

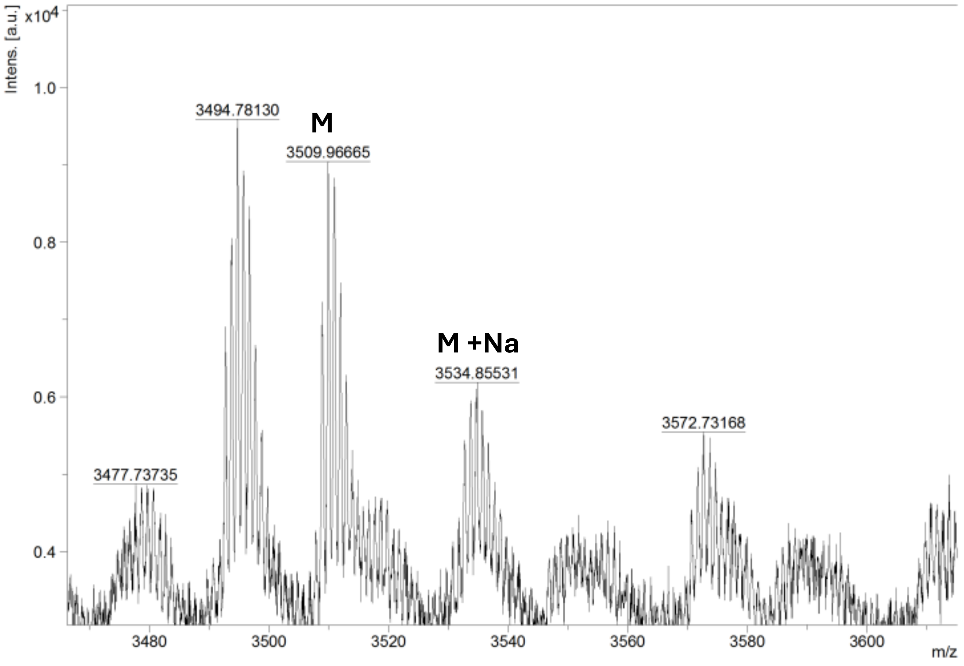

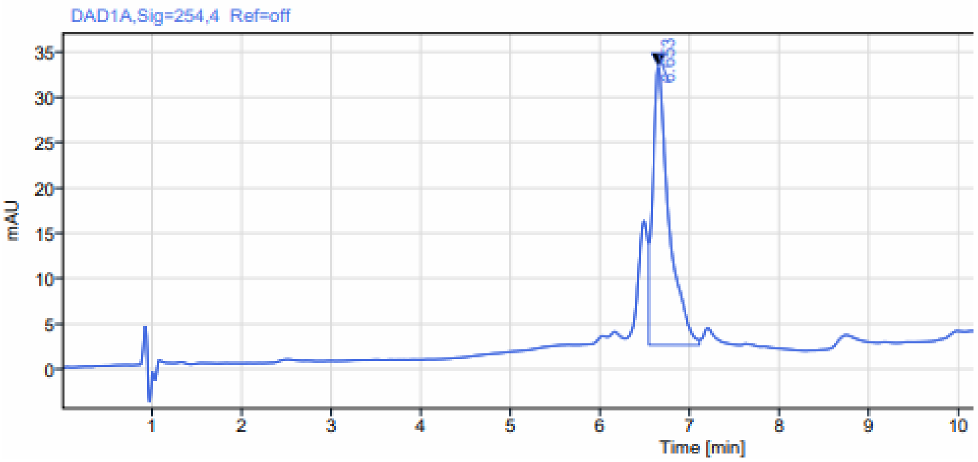

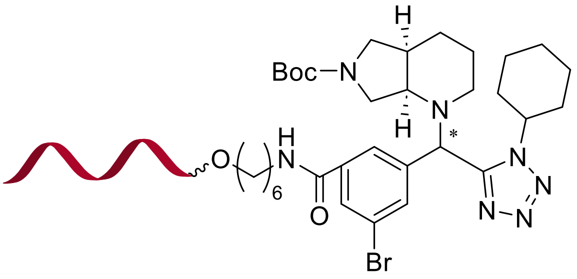

TC-8m

MS calc. 3656.0, found: 3657.8

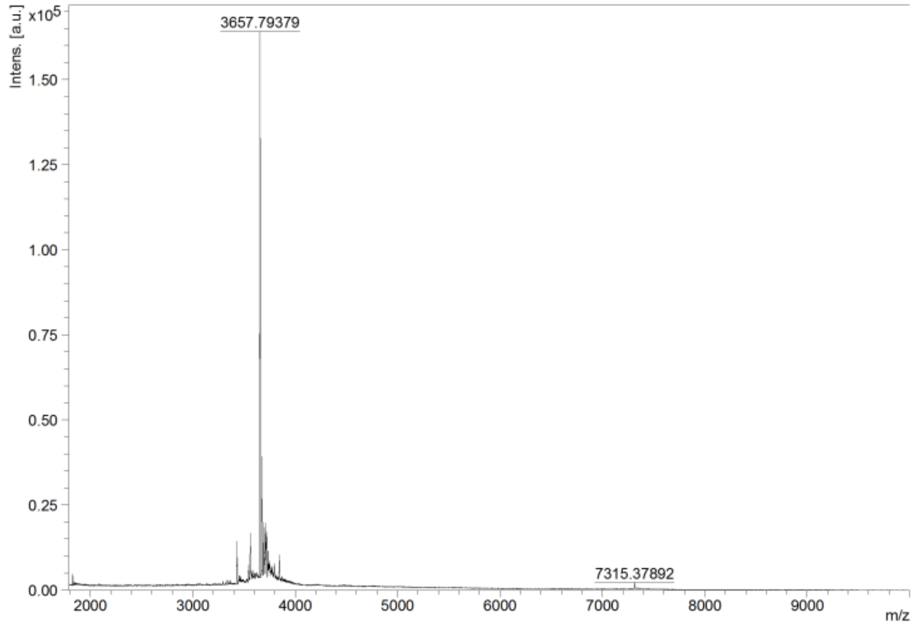

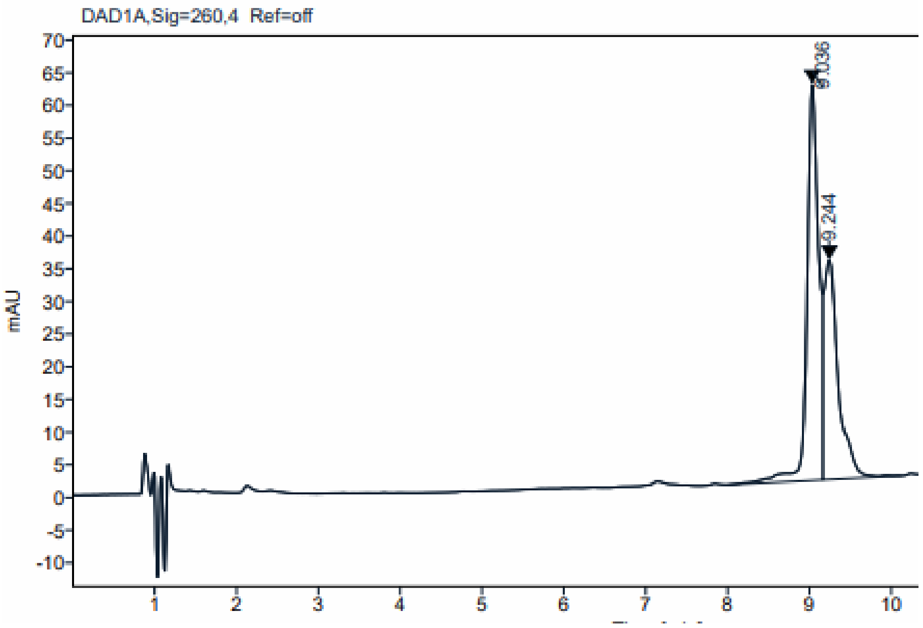

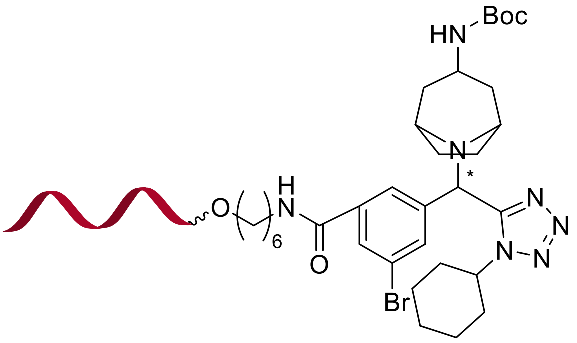

TC-8n

MS calc. 3656.0, found: 3657.9

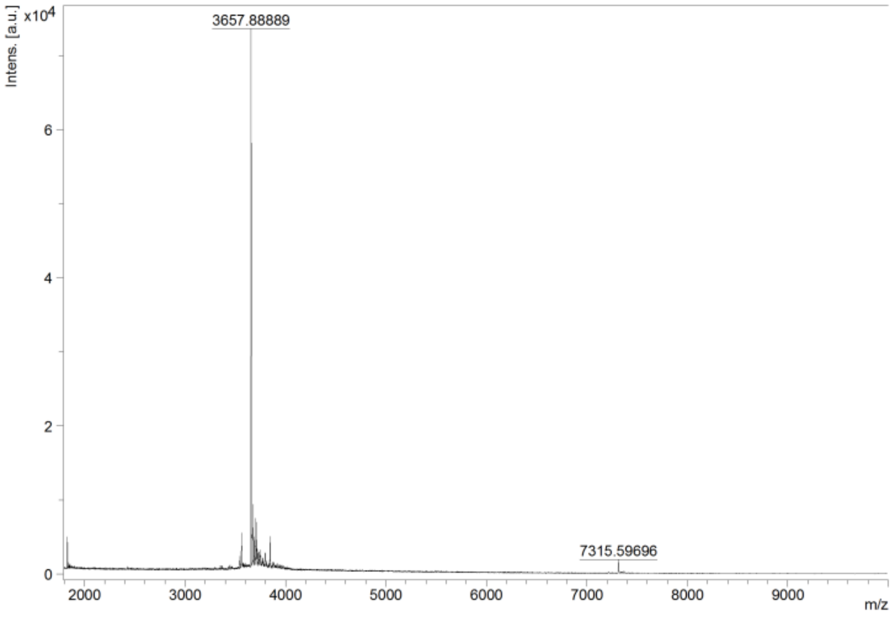

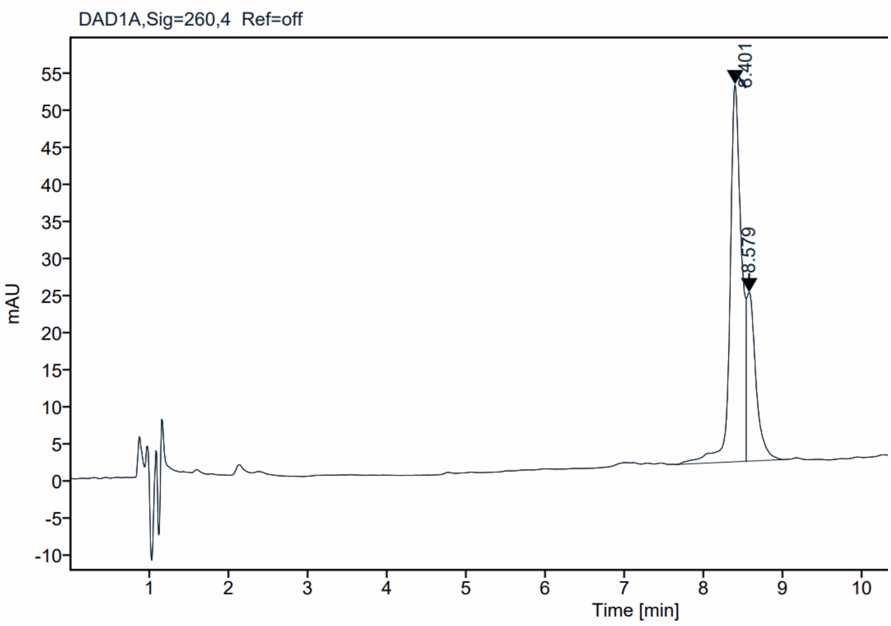

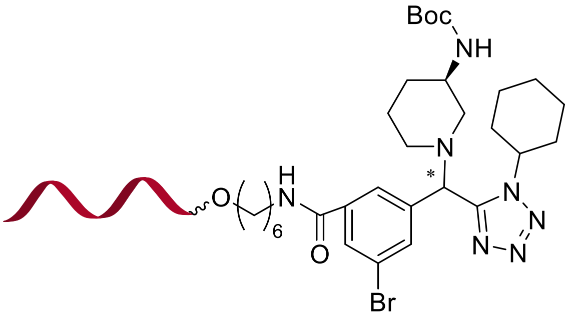

TC-8o

MS calc. 3630.0, found: 3631.0

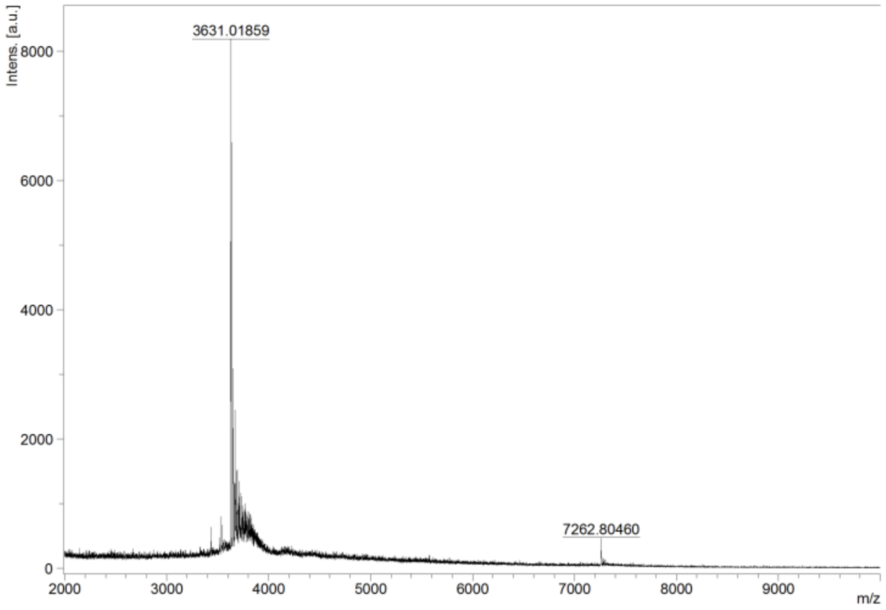

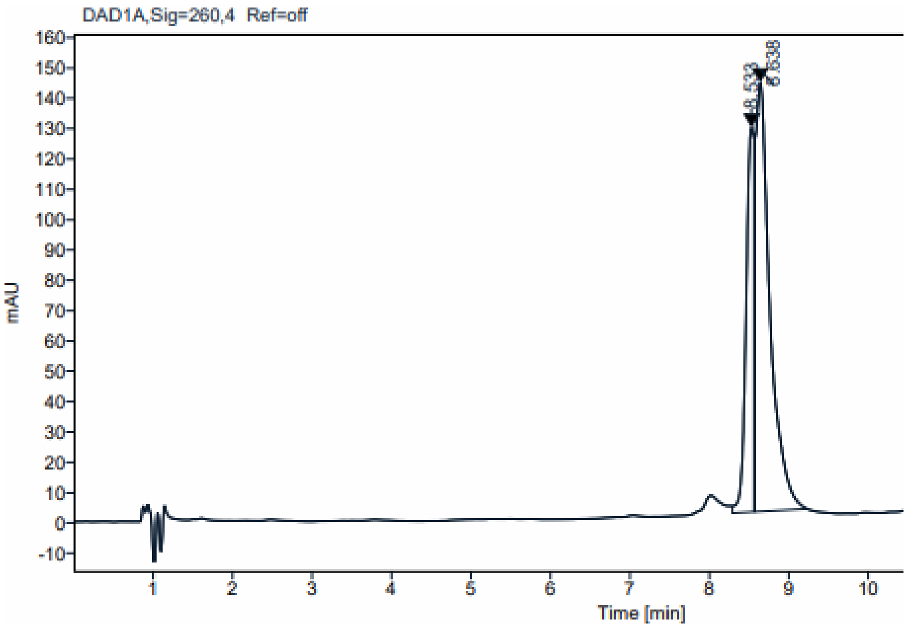

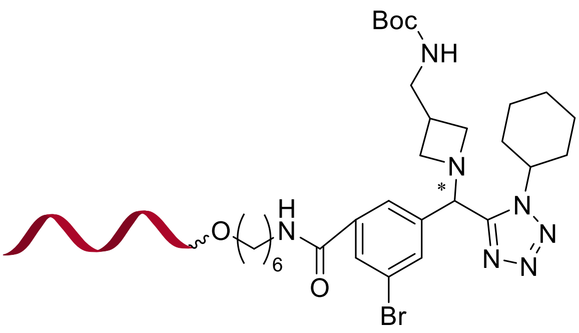

TC-8p

MS calc. 3616.0; found: 3617.7

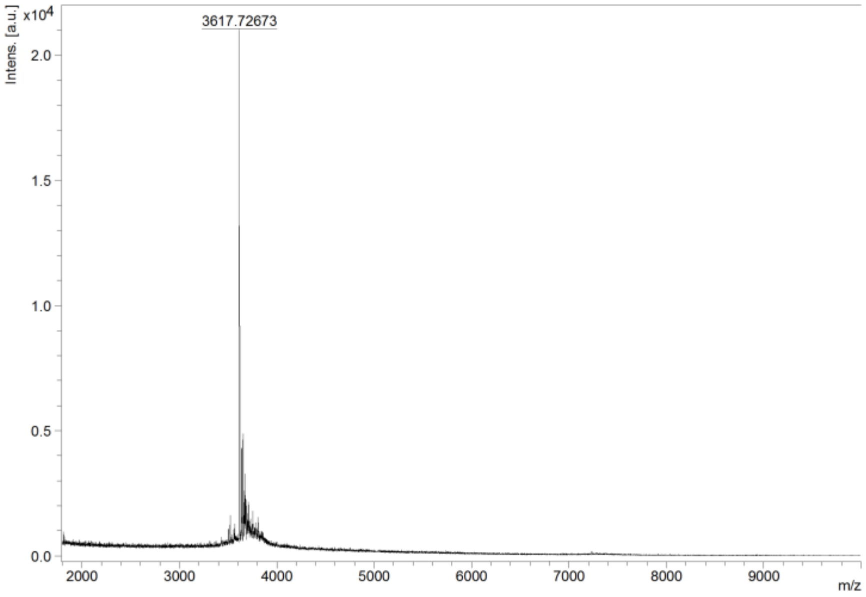

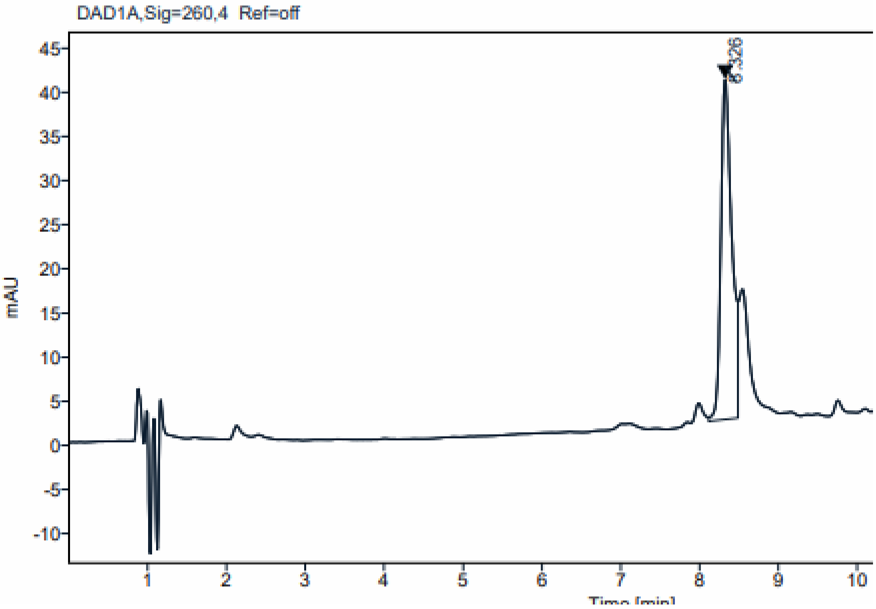

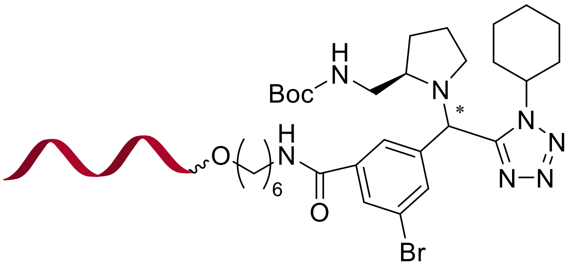

TC-8q

MS calc. 3629.0; found: 3631.9

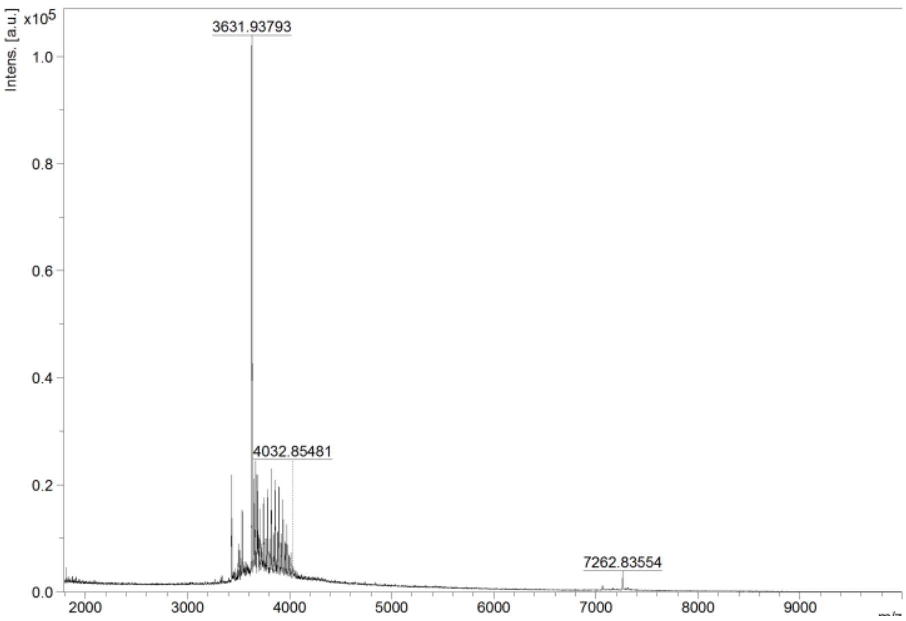

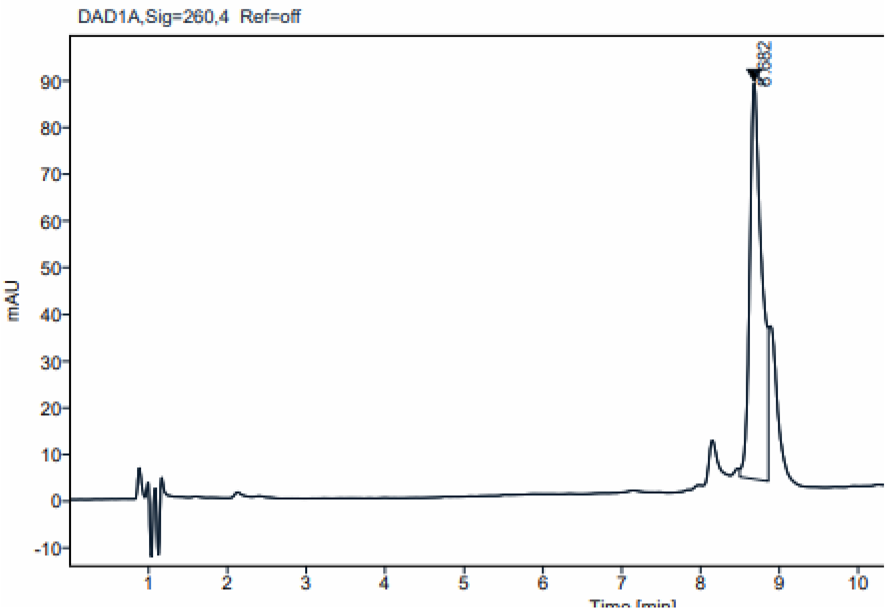

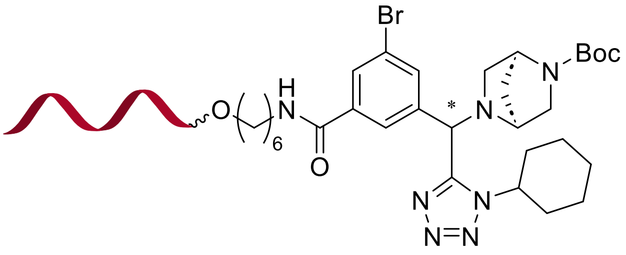

TC-8r

MS calc. 3627.0; found: 3629.8

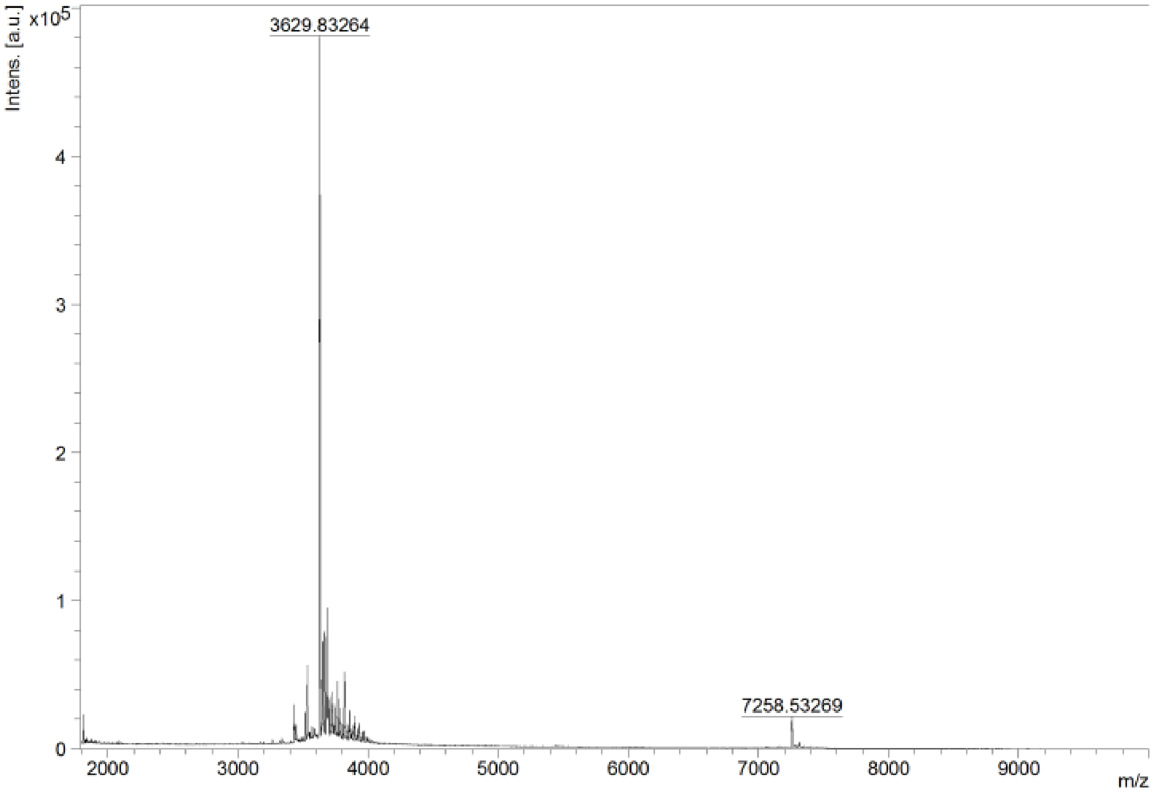

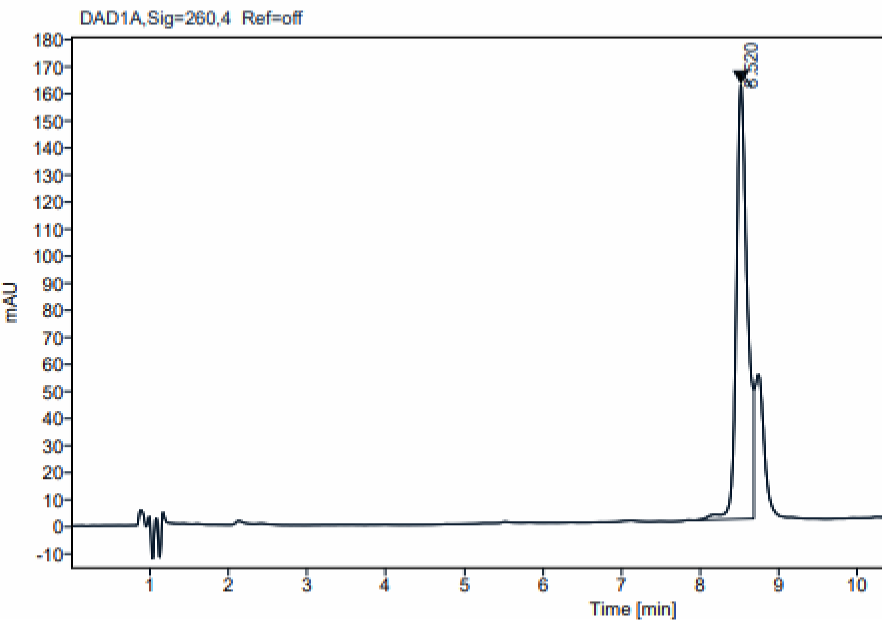

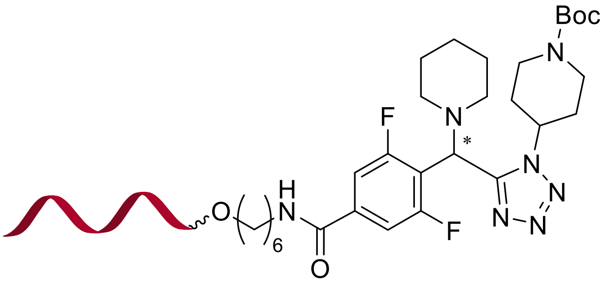

TC-8s

MS calc. 3571.0; found: 3471.8 [M-Boc]

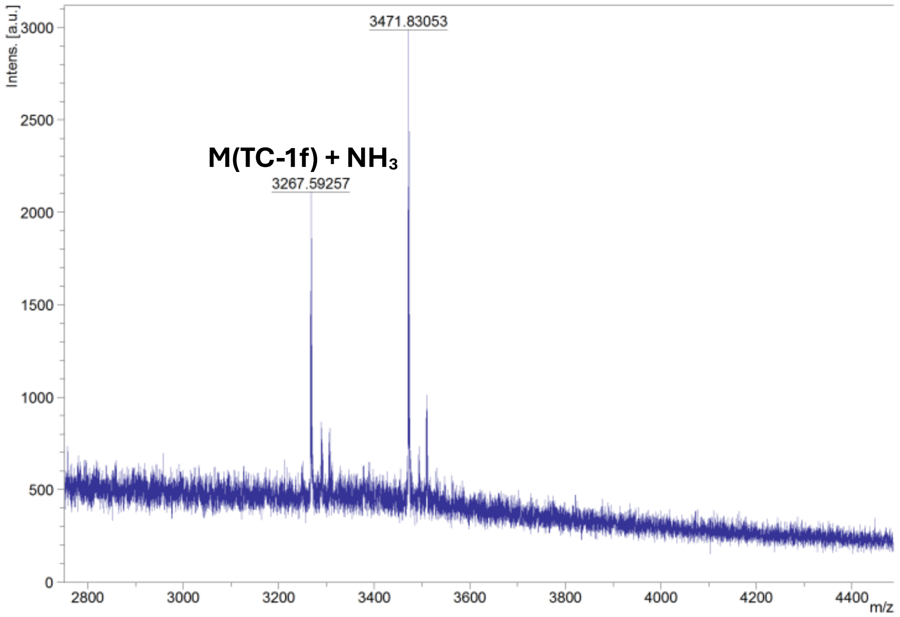

from crude

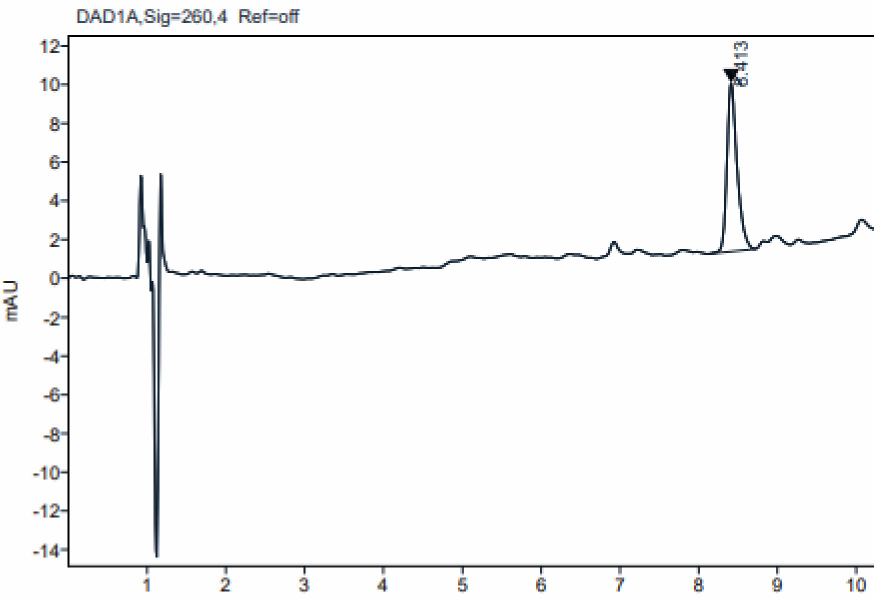

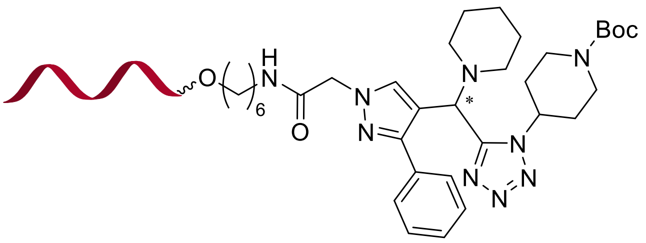

TC-8t

MS calc. 3614.0; found: 3521.0 [M-Boc]

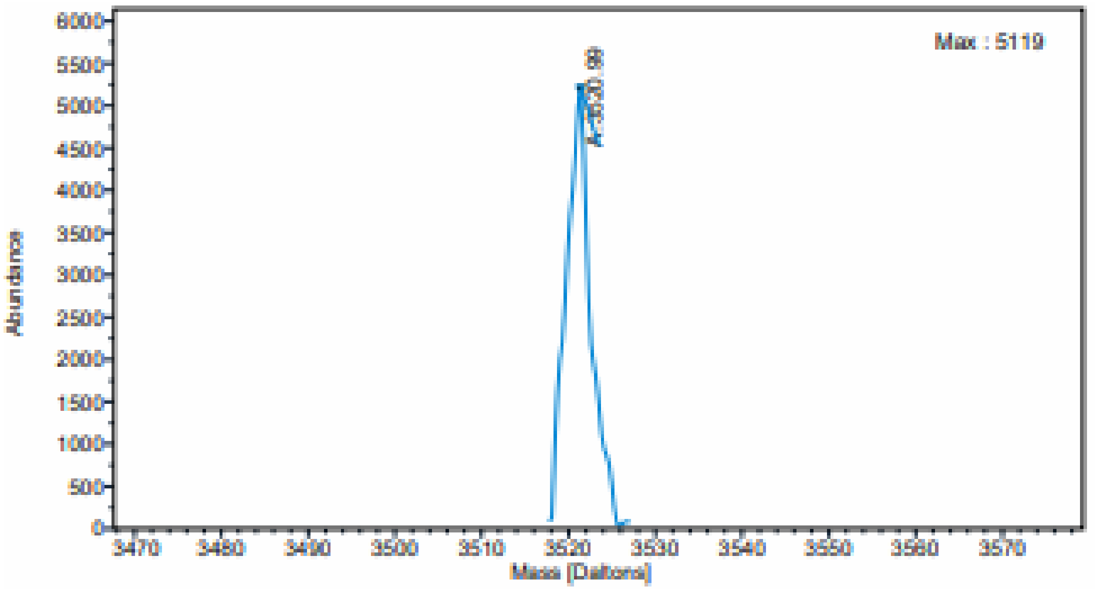

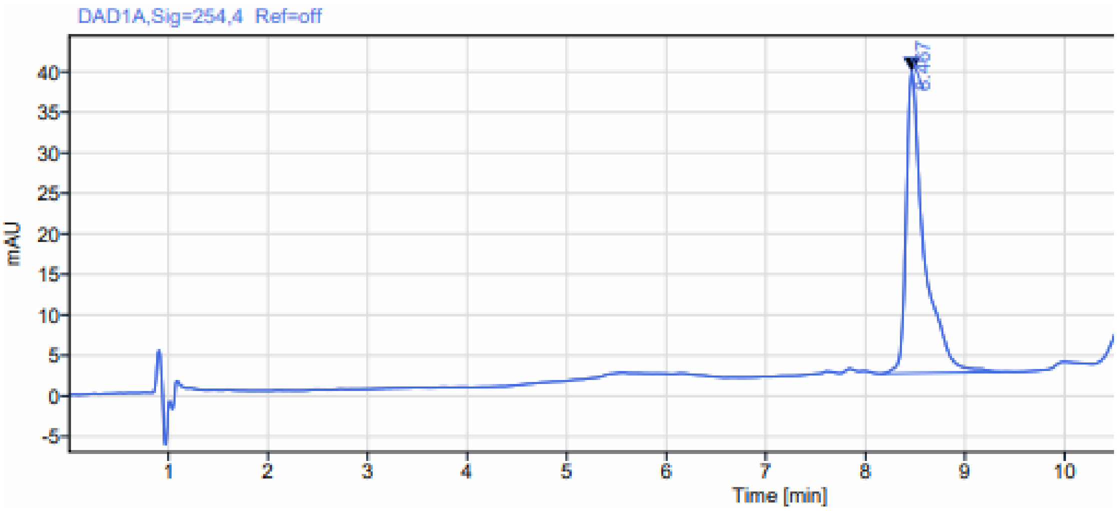

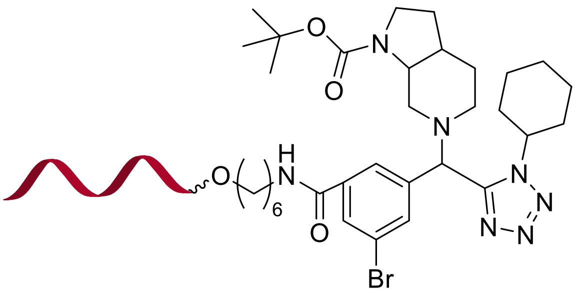

TC-8u

MS calc.3655.0; found: 3657.0

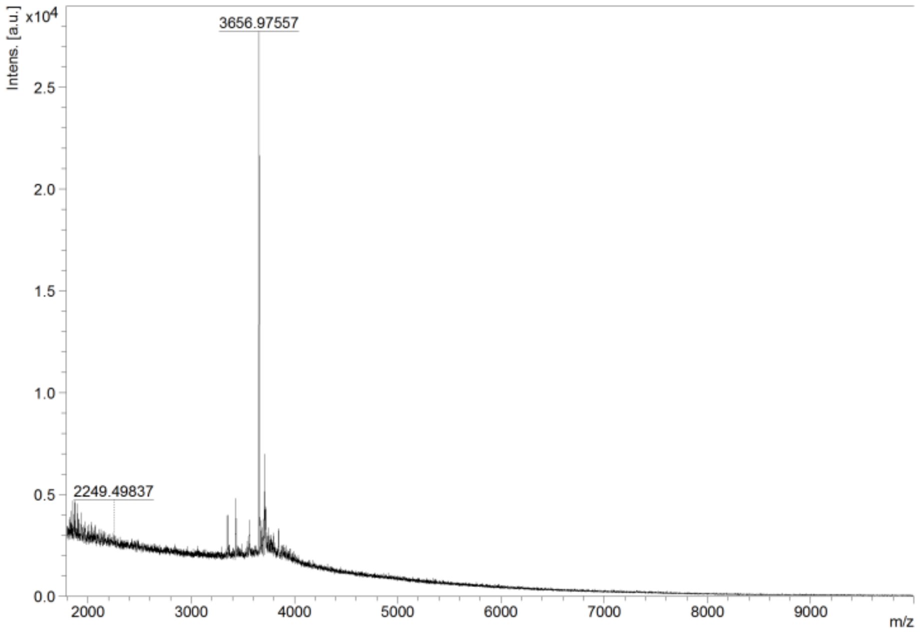

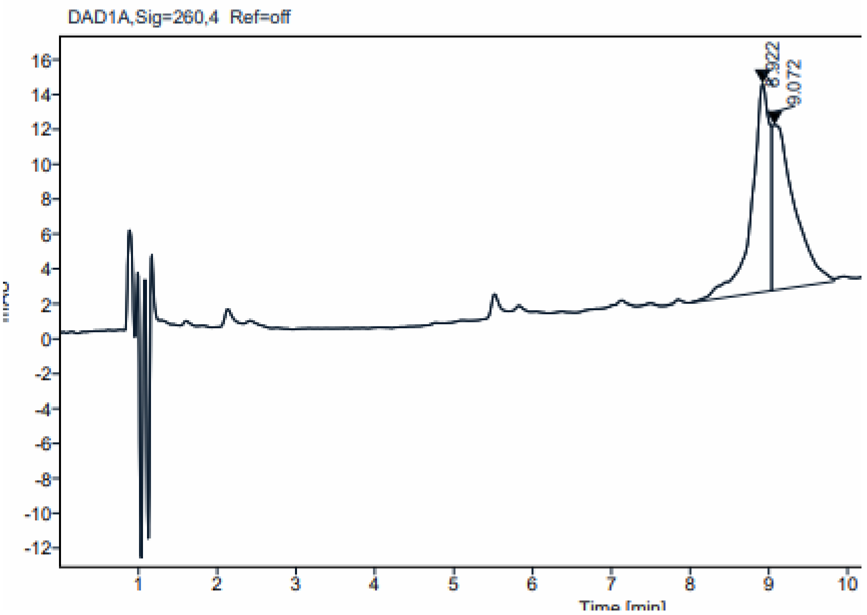

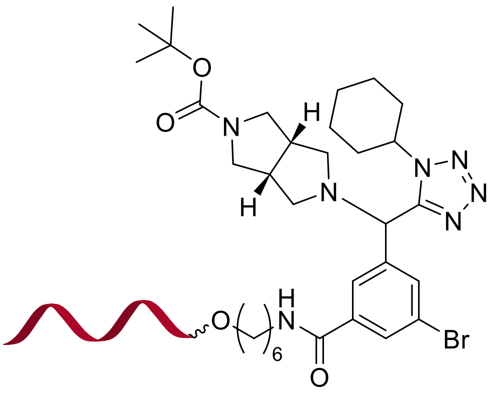

TC-8v

MS calc. 3641.0; found: 3643.9

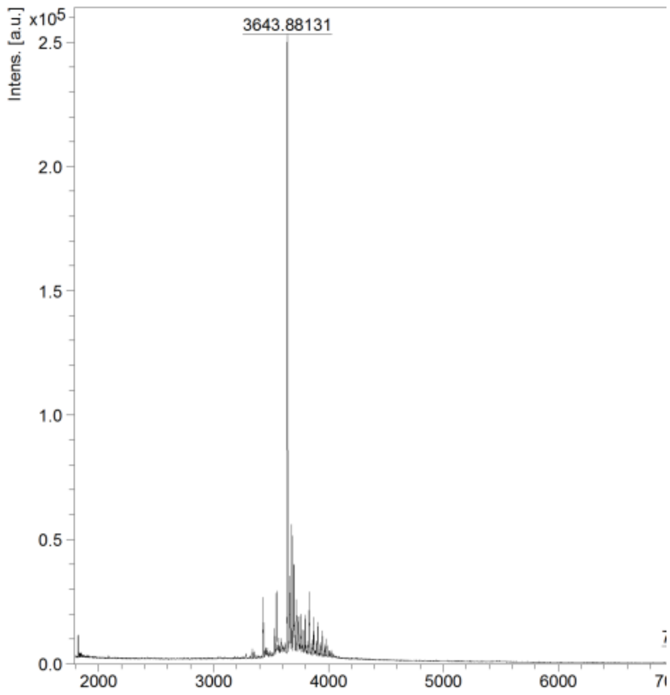

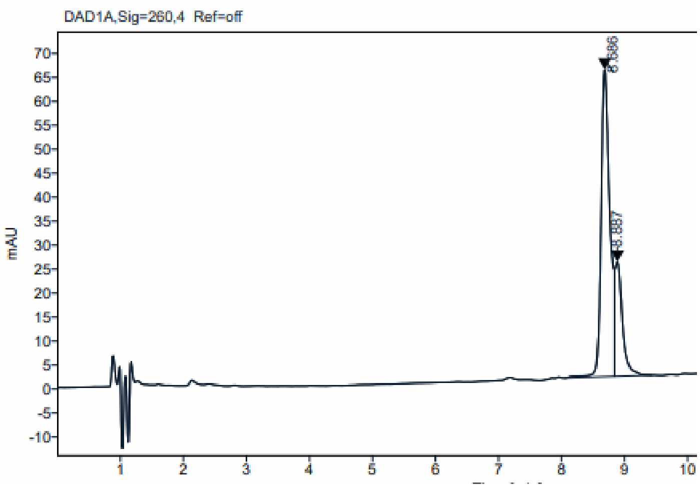

HPLC traces of representative examples of crude UA-4CR products

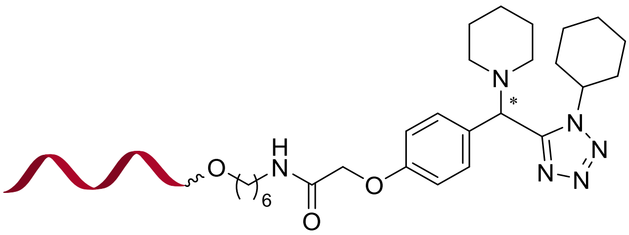

TC-8a

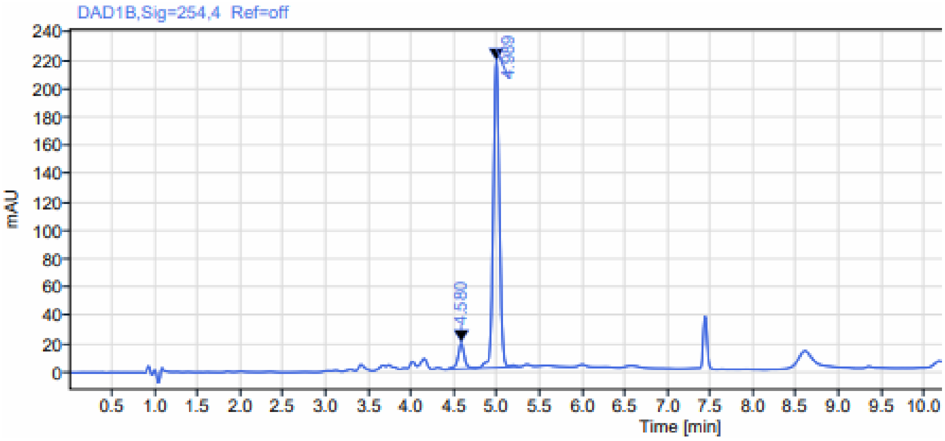

starting material

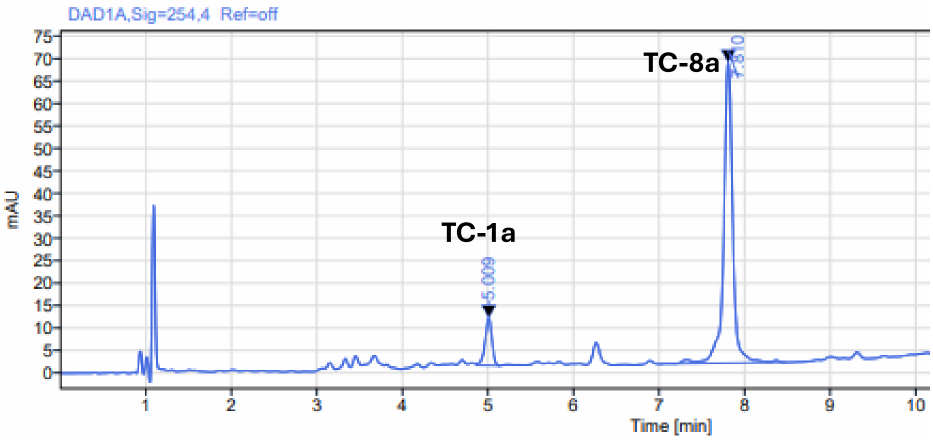

crude

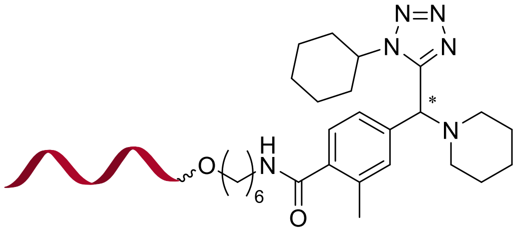

TC-8b

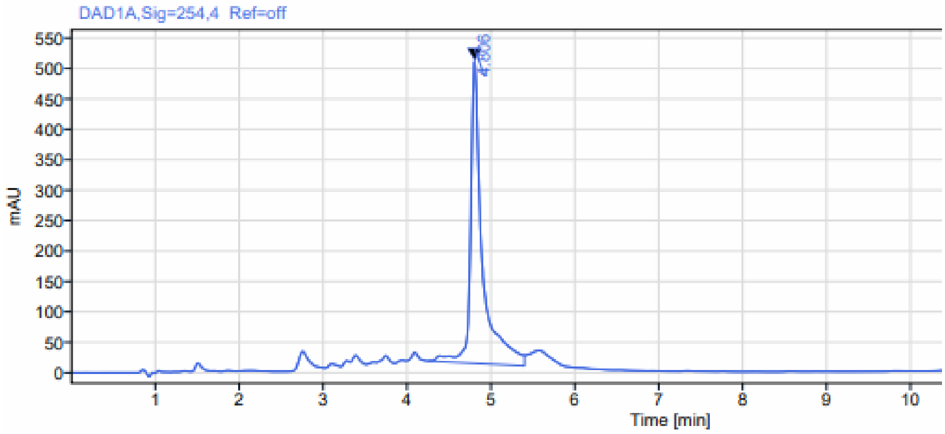

starting material

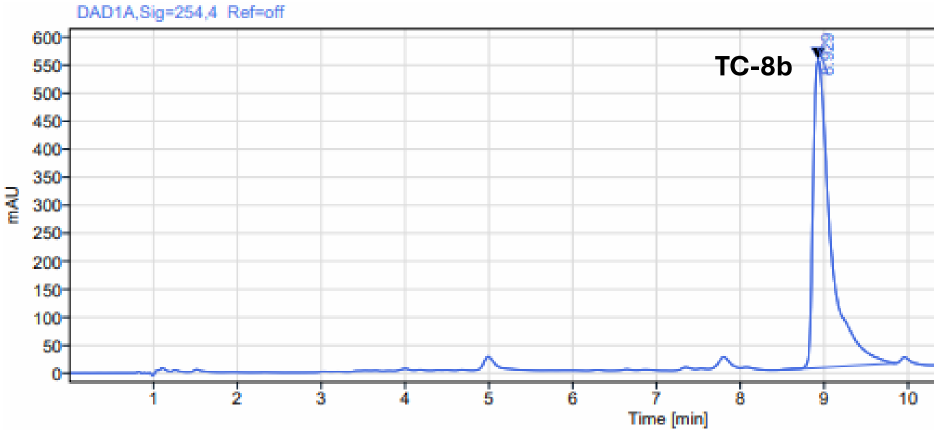

crude

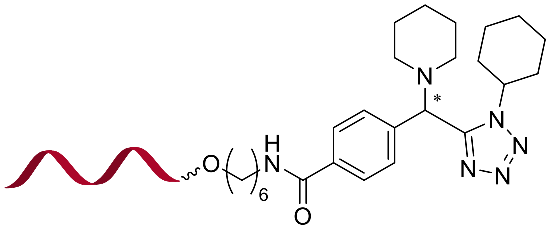

TC-8c

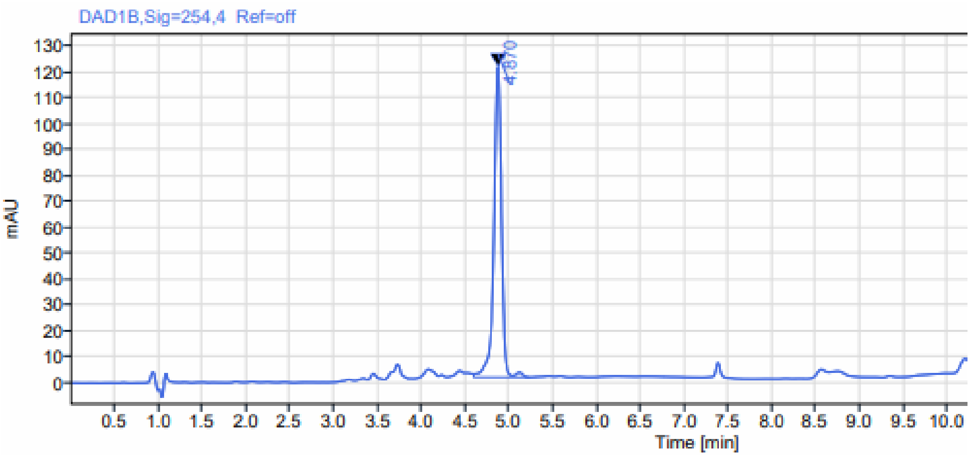

starting material

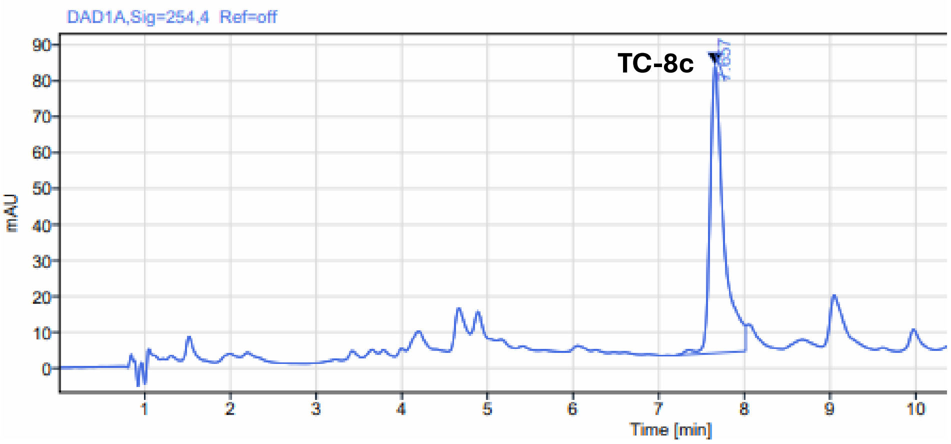

crude

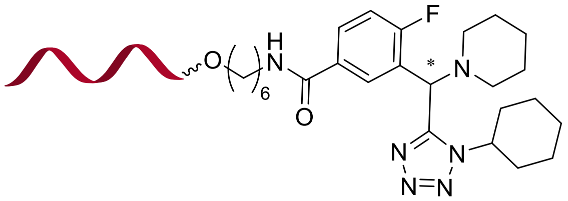

TC-8d

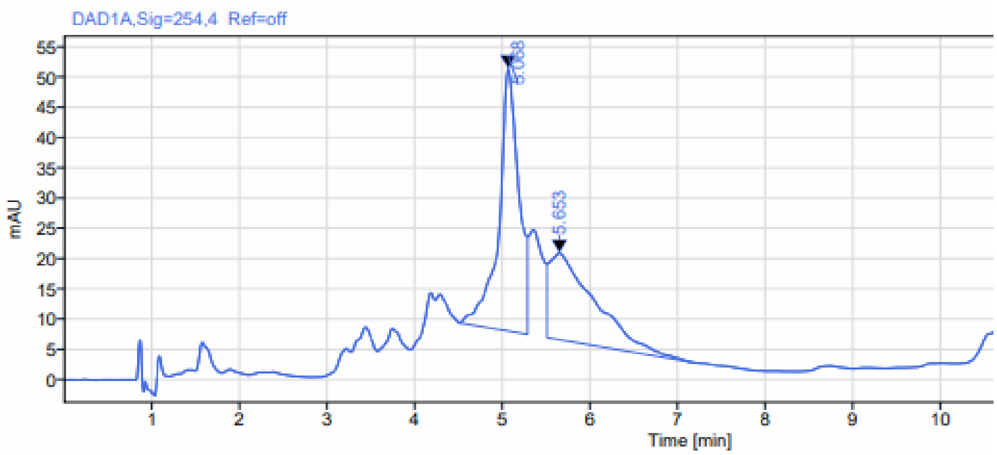

starting material

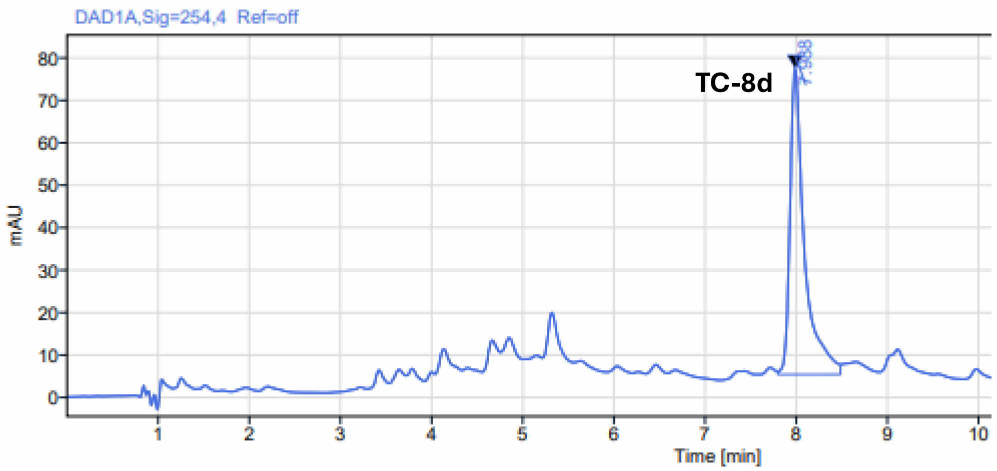

crude

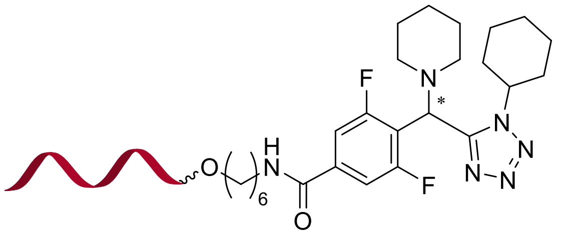

TC-8e

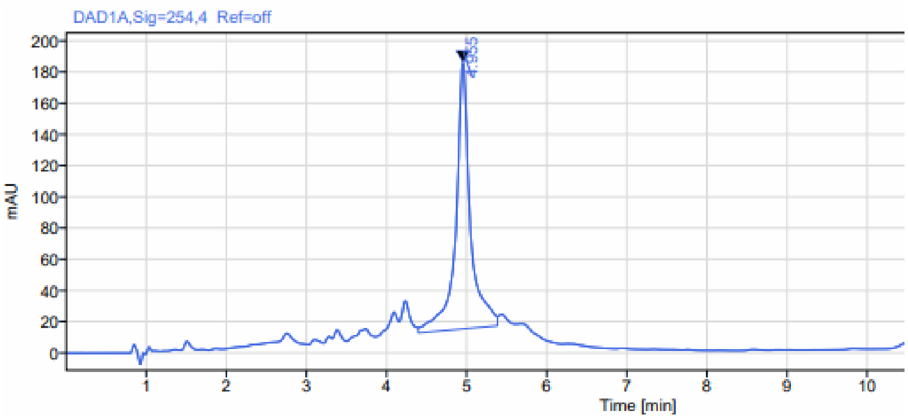

starting material

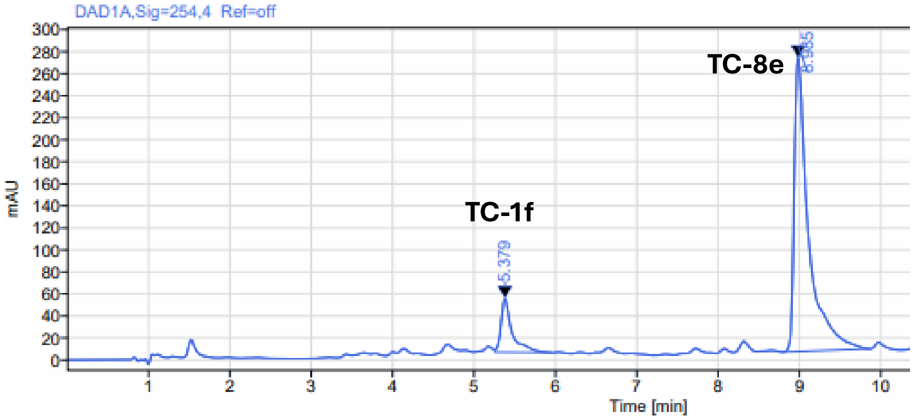

crude

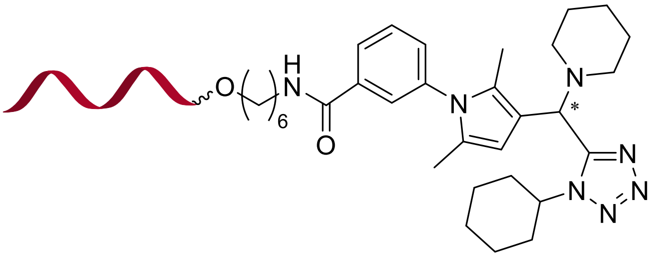

TC-8f

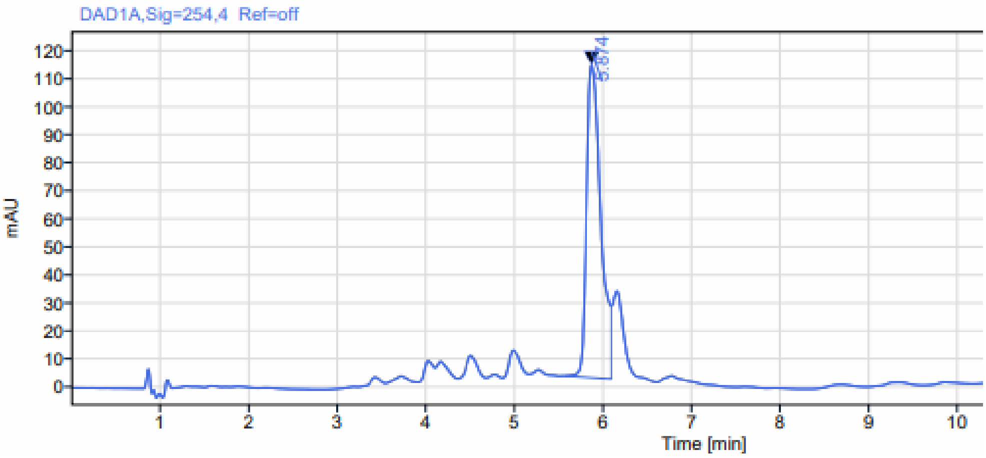

starting material

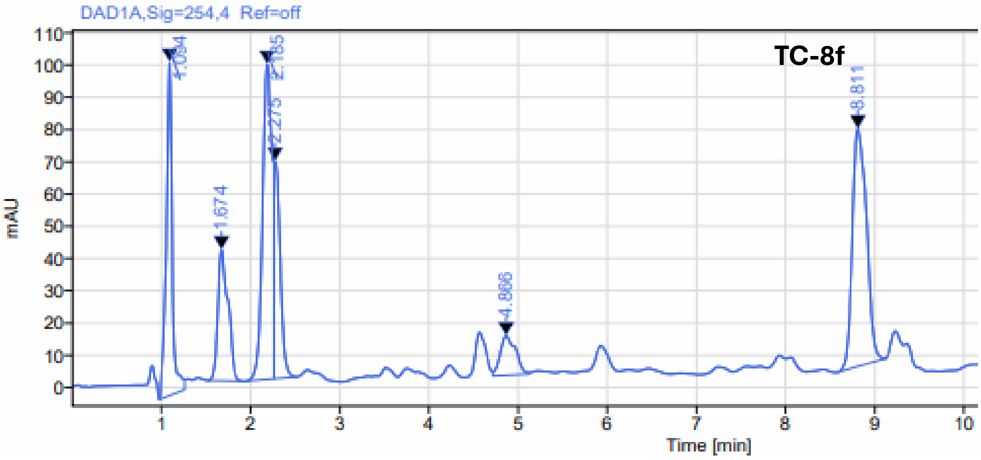

crude

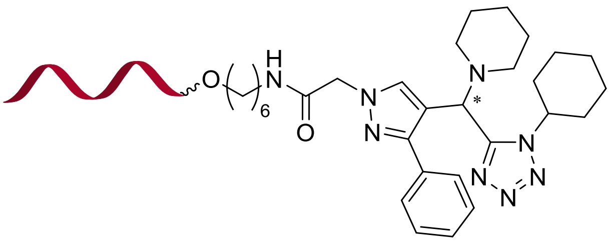

TC-8g

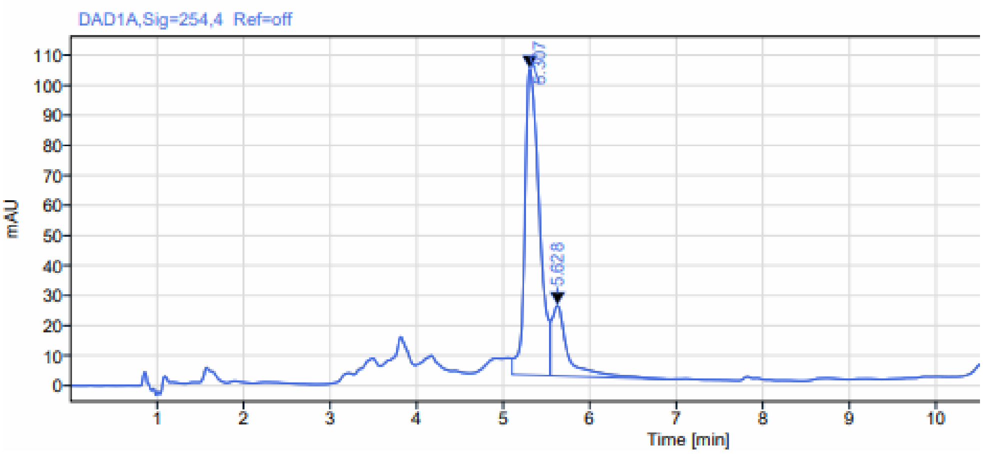

starting material

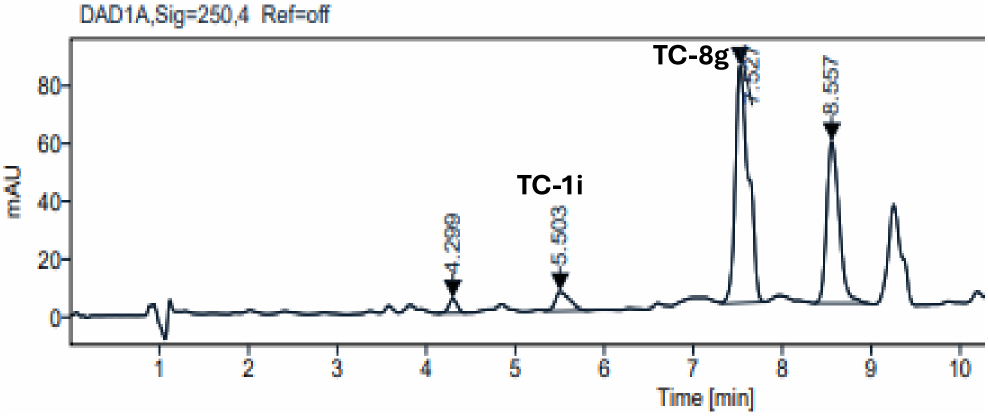

crude

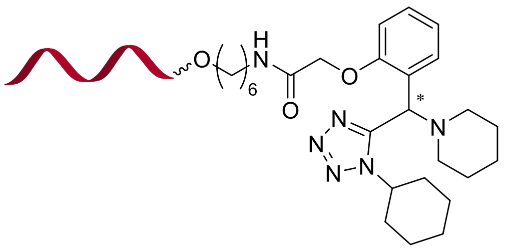

TC-8i

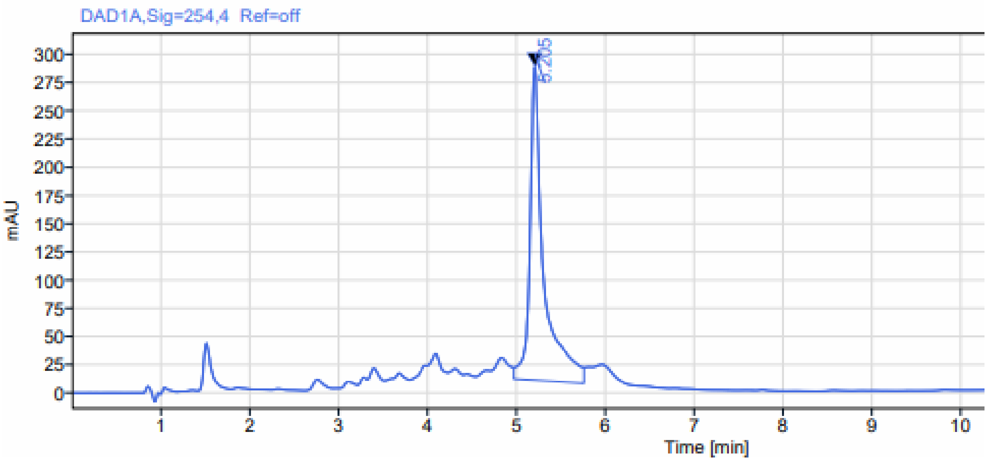

starting material

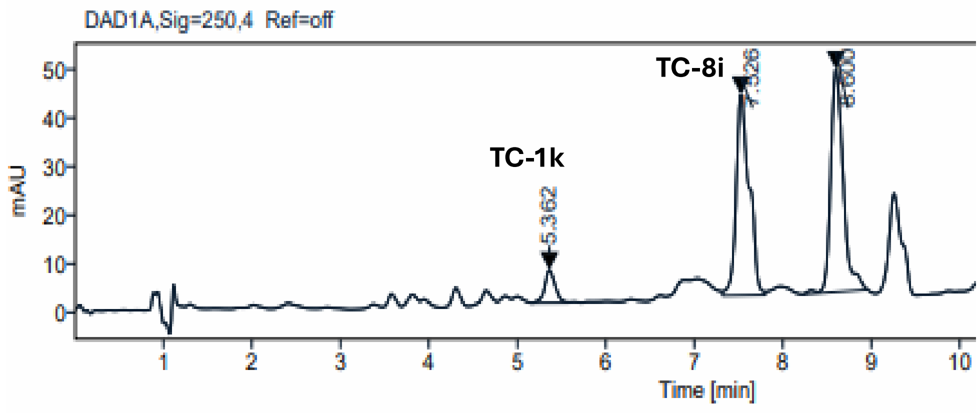

crude

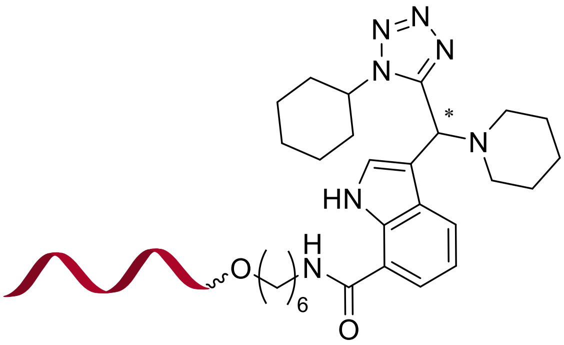

TC-8k

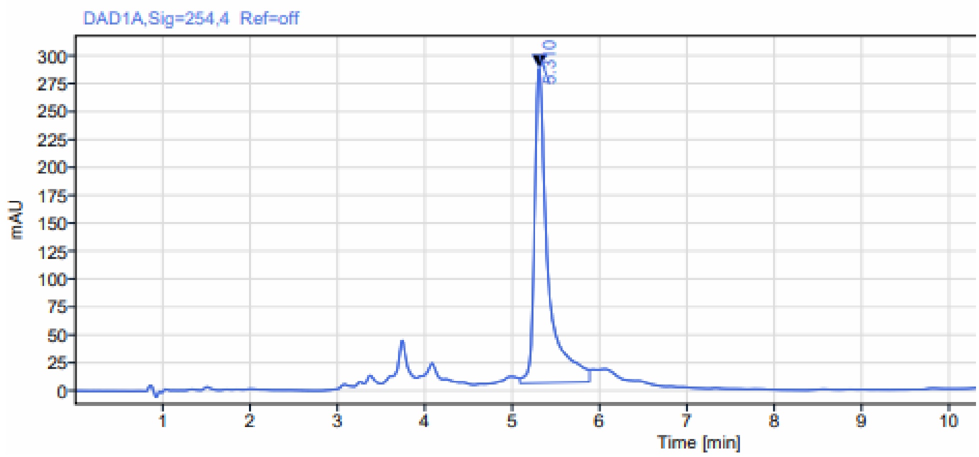

starting material

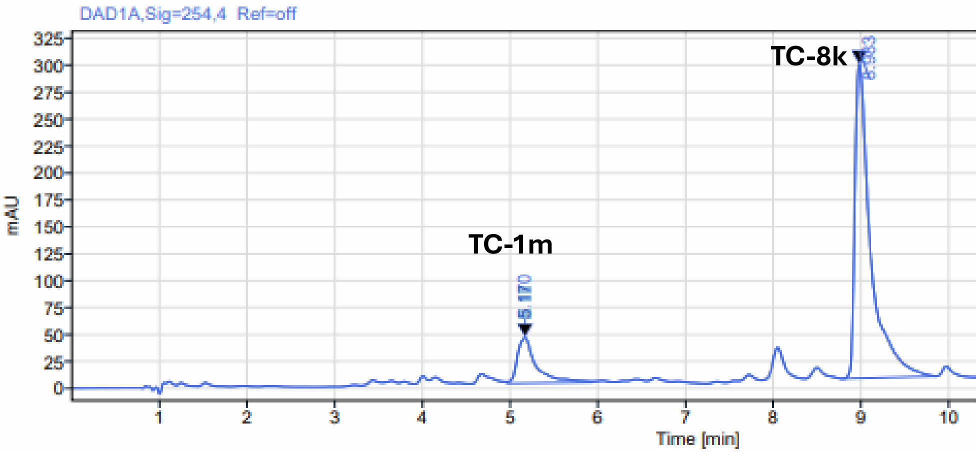

crude

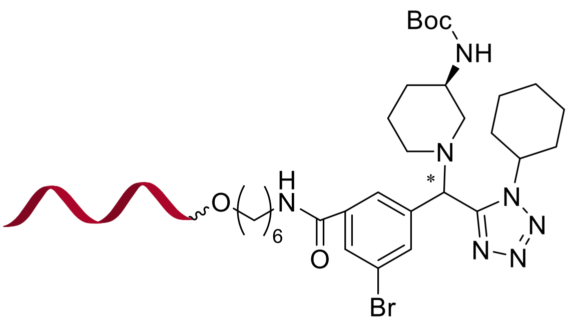

TC-8o

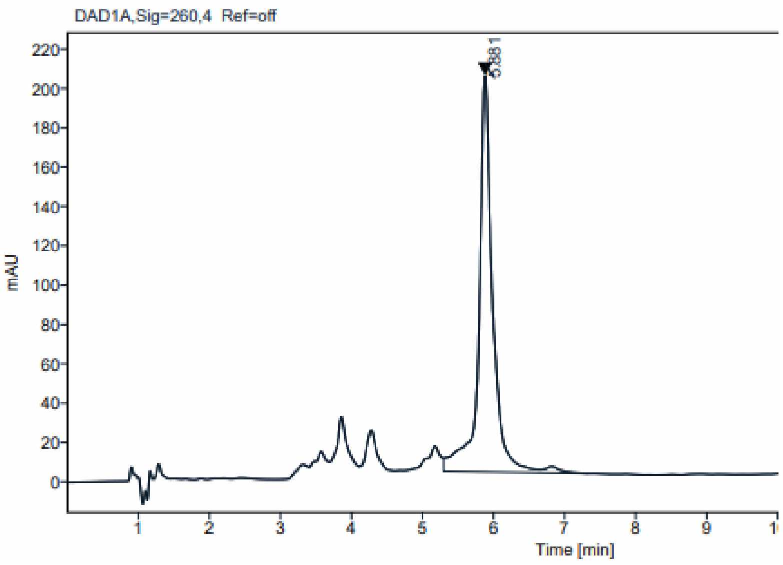

starting material

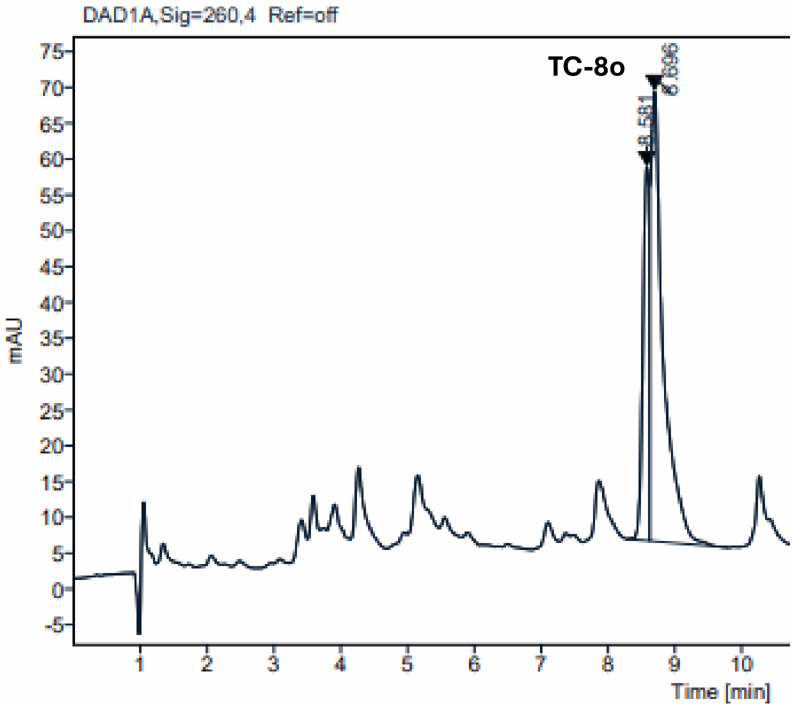

crude

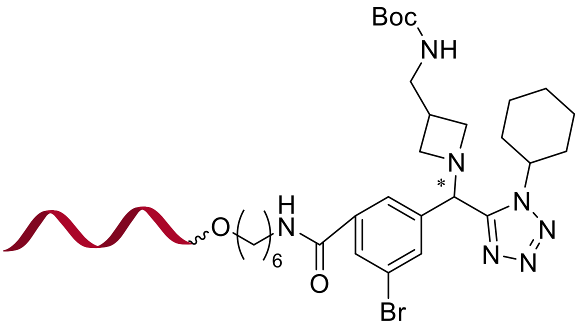

TC-8p

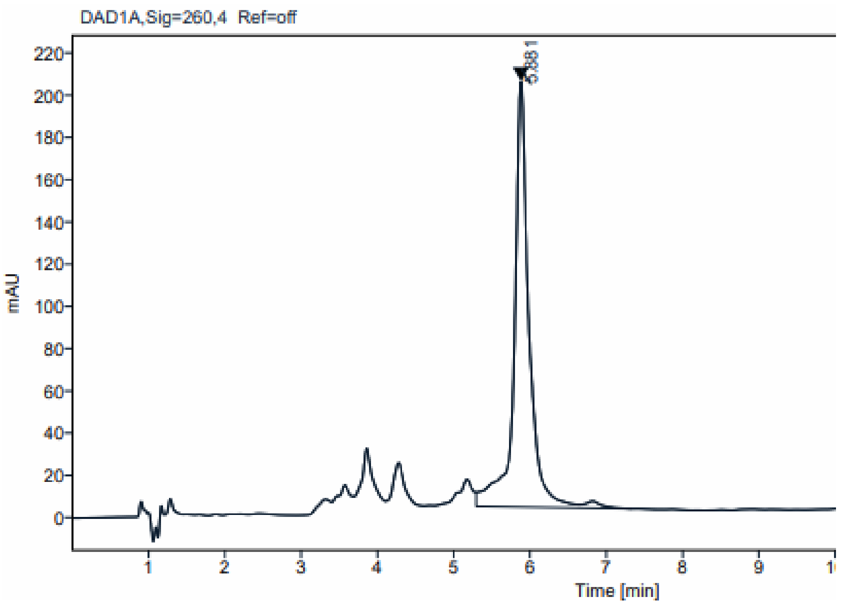

starting material

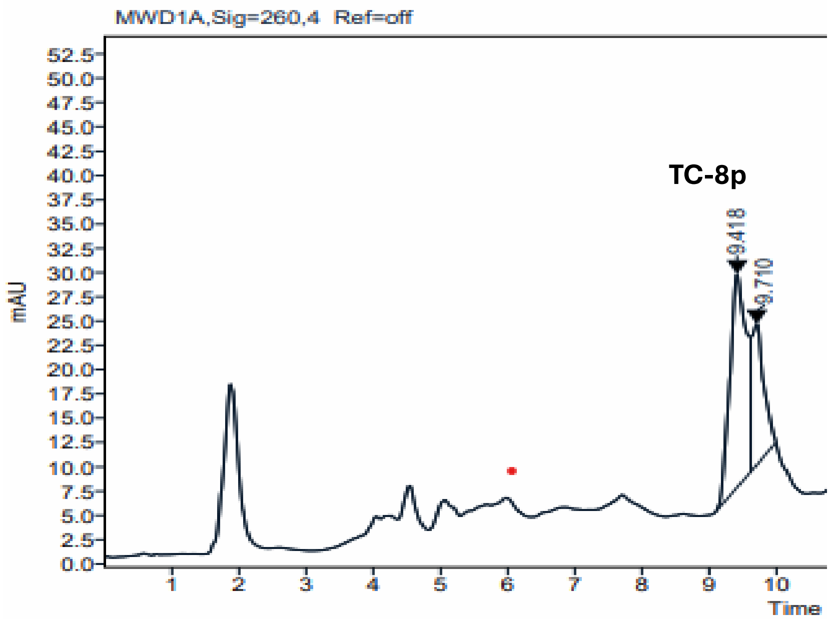

crude

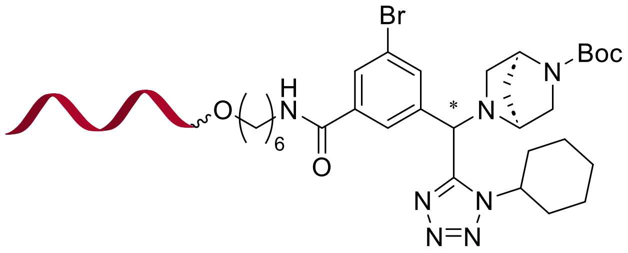

TC-8q

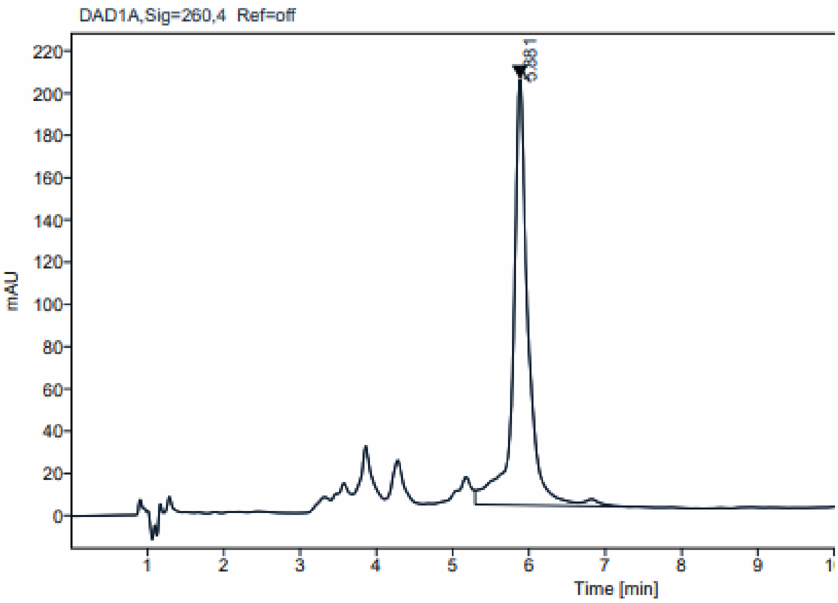

starting material

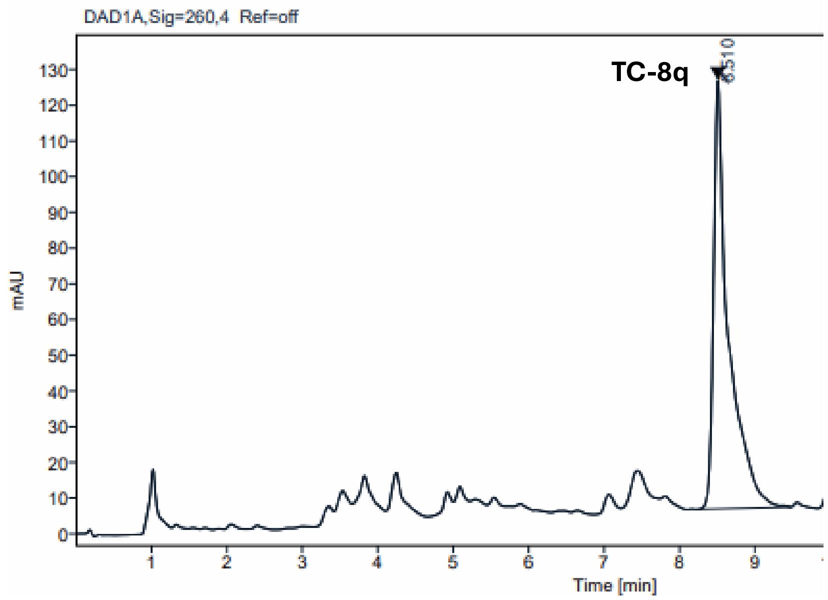

crude

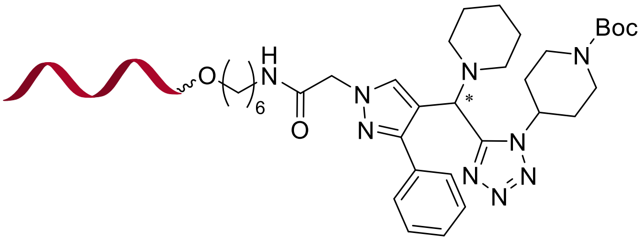

TC-8t

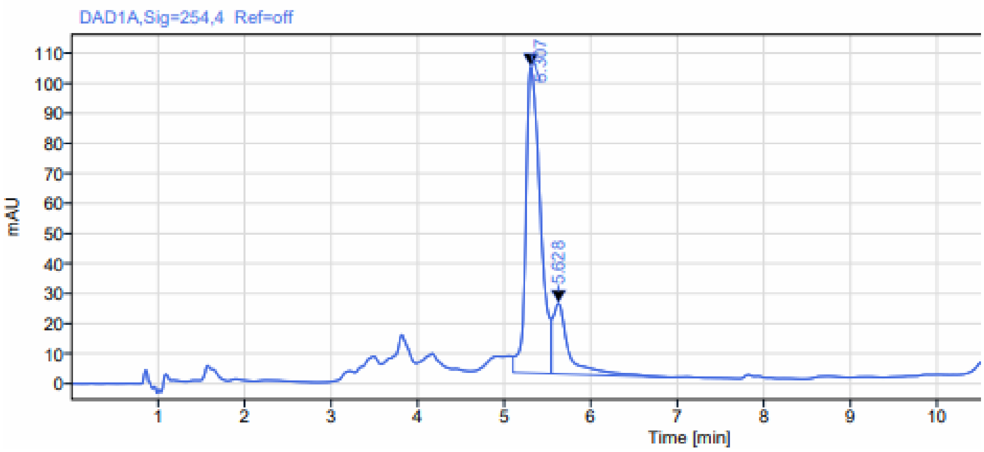

starting material

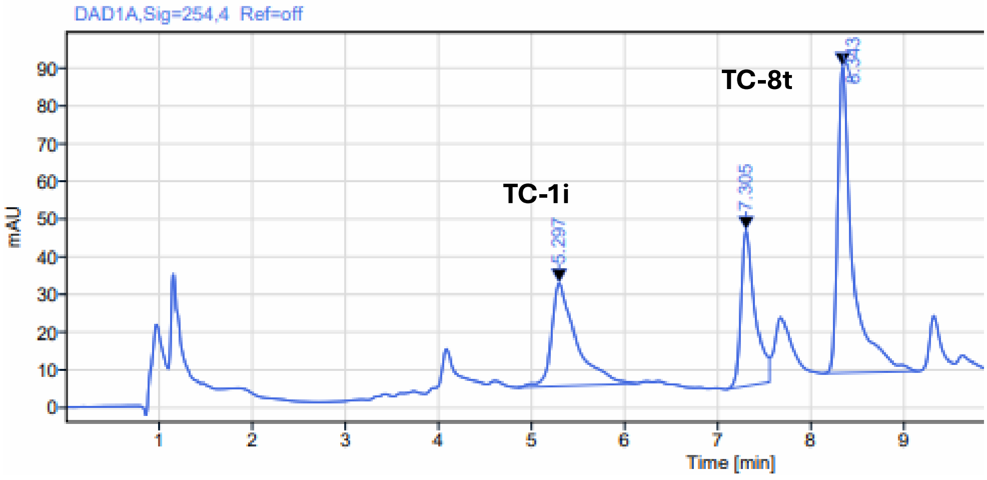

crude

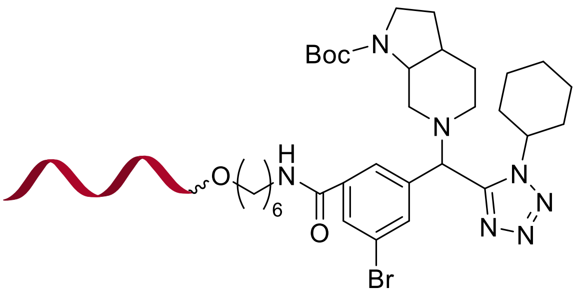

TC-8v

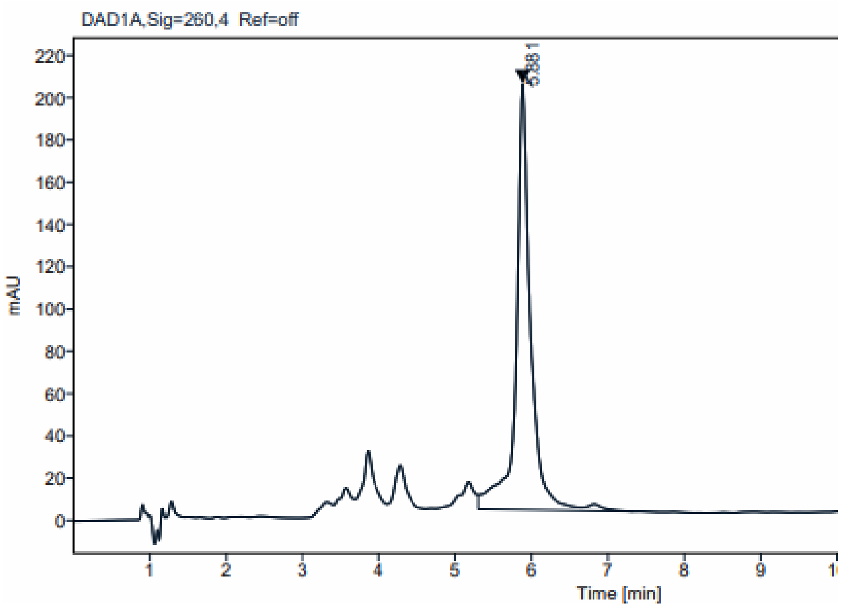

starting material

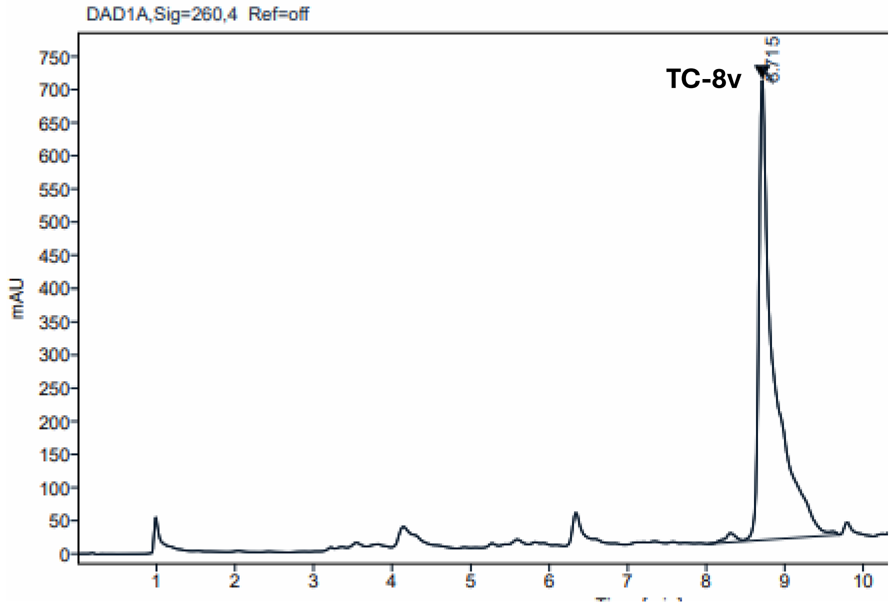

crude

Groebke-Blackburn-Bienaymé three-component reaction

Graphic presentation of the procedure

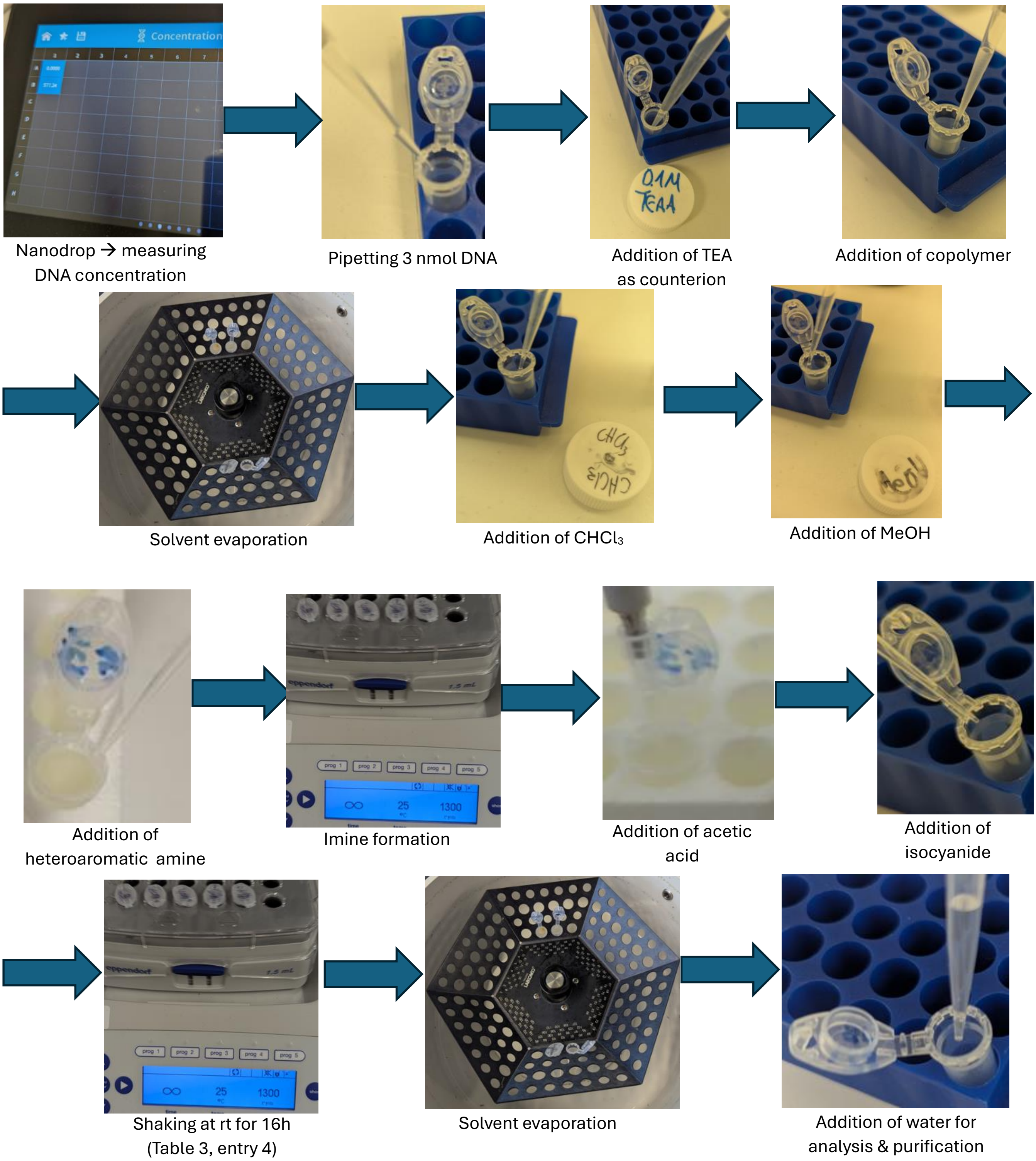

Optimization of Groebke-Blackburn-Bienaymé three-component reaction

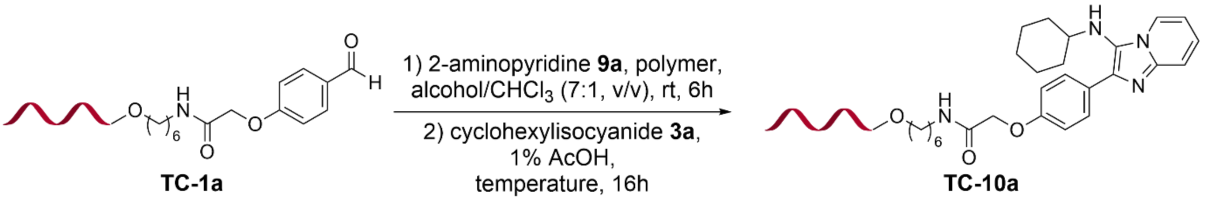

Table S8. Conversion rates for different reaction conditions for the Groebke-Blackburn-Bienaymé three-component reaction.

No.	Solvent[a]	equivalents	concentration	temperature	Catalyst[b]	Product conversion
**1**	MeOH/$CHCl_3$	250	9 mM	rt[c]	AcOH	81%
**2**	MeOH/$CHCl_3$	500	19 mM	rt	AcOH	89%
**3**	MeOH/$CHCl_3$	1000	37.5 mM	rt	AcOH	>95%
**4**	MeOH/$CHCl_3$	2000	75 mM	rt	AcOH	>95%
**5**	MeOH/$CHCl_3$	2000	75 mM	15°C	AcOH	95%
**6**	MeOH/$CHCl_3$	2000	75 mM	5°C	AcOH	>95%
**7**	EtOH/$CHCl_3$	2000	75 mM	rt	AcOH	>95%
**8**	iPrOH/$CHCl_3$	2000	75 mM	rt	AcOH	95%

[a] alcohol/$CHCl_3$ (7:1, vol/vol); [b] 1% of acetic acid; [c]rt means 25°C

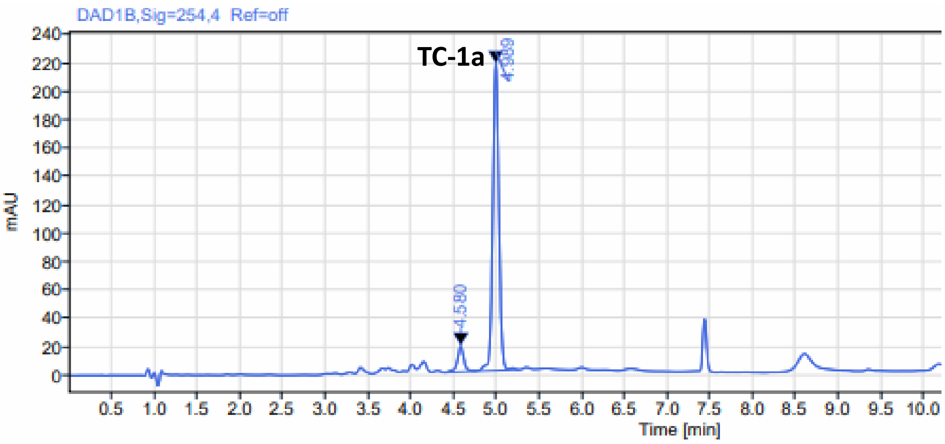

Figure S58. Analytical HPLC trace of starting material **TC-1a**.

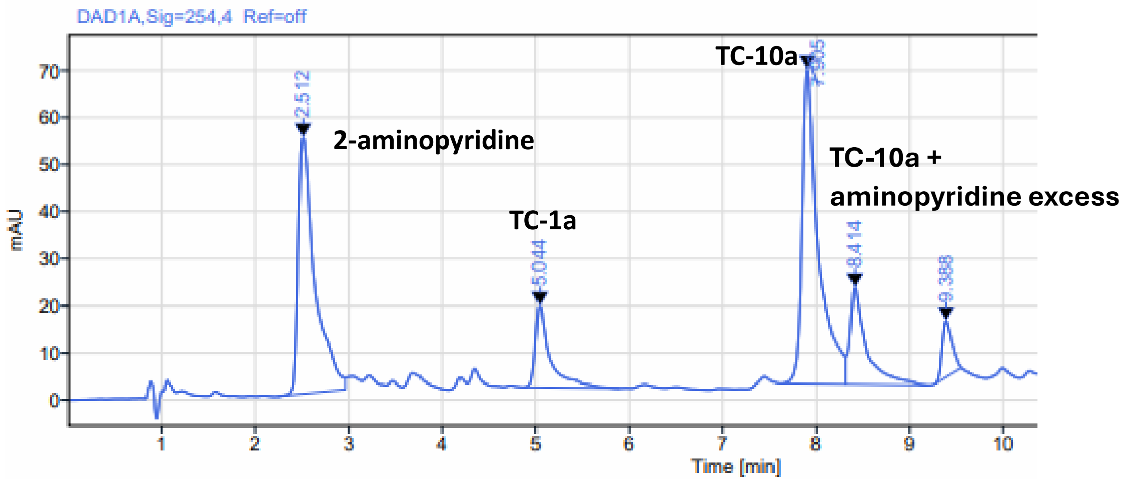

Figure S59. Crude analytical HPLC trace for product **TC-10a** with 250 equiv amine, 250 equiv isocyanide in MeOH/$CHCl_3$ (7:1, v/v) at rt (entry 1).

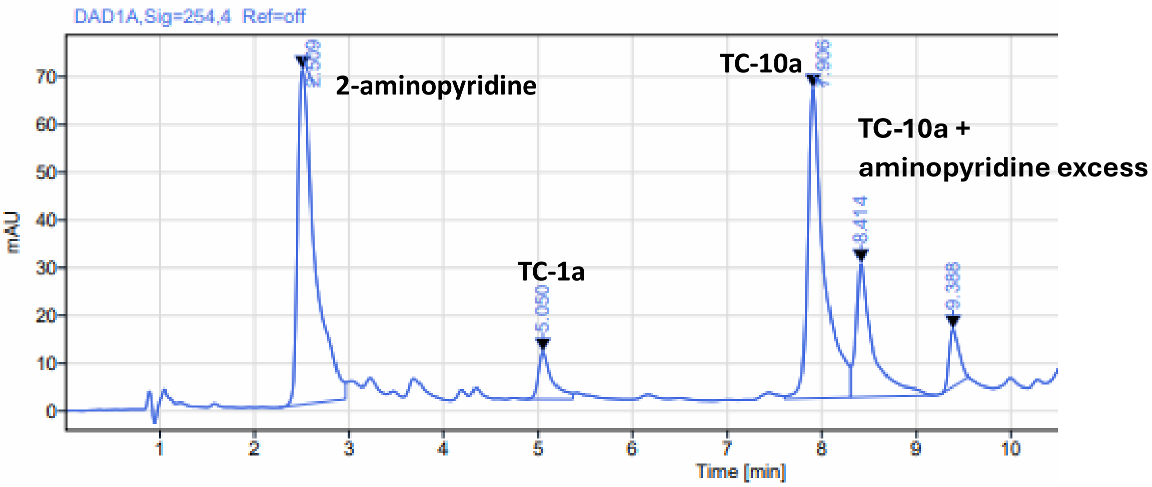

Figure S60. Crude analytical HPLC trace for product **TC-10a** with 500 equiv amine, 500 equiv isocyanide in MeOH/$CHCl_3$ (7:1, v/v) at rt (entry 2).

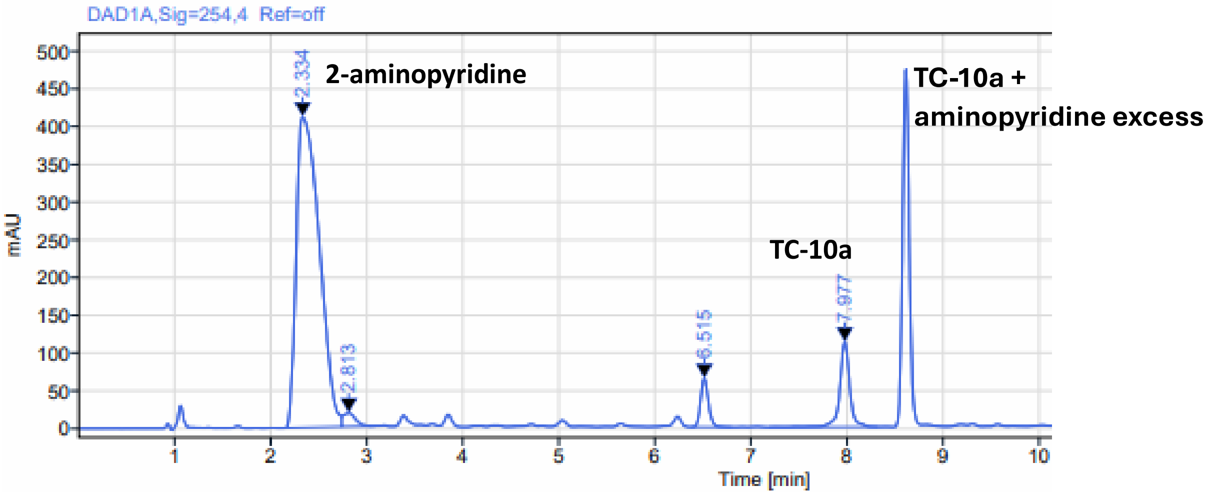

Figure S61. Crude analytical HPLC trace for Product **TC-10a** with 1000 equiv amine, 1000 equiv isocyanide in MeOH/$CHCl_3$ (7:1, v/v) at rt (entry 3).

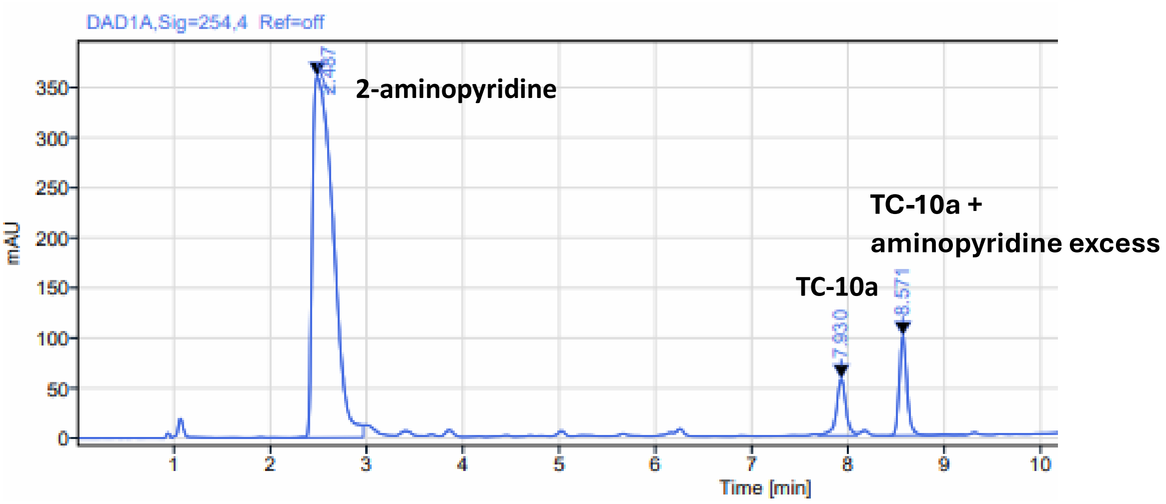

Figure S62. Crude analytical HPLC trace for Product **TC-10a** with 2000 equiv amine, 2000 equiv isocyanide in MeOH/$CHCl_3$ (7:1, v/v) at rt (entry 4).

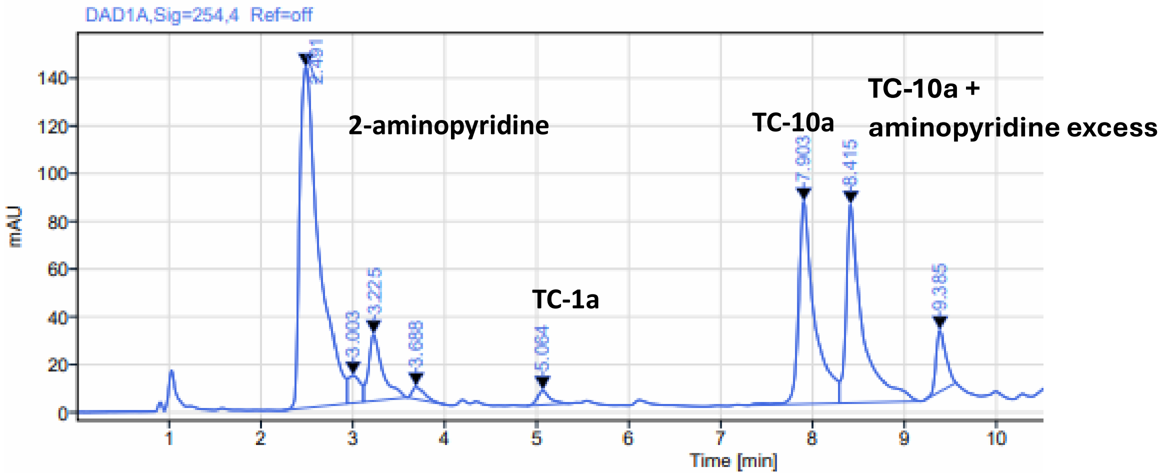

Figure S63. Crude analytical HPLC trace for product **TC-10a** with 2000 equiv amine, 2000 equiv isocyanide in MeOH/$CHCl_3$ (7:1, v/v) at 15°C (entry 5).

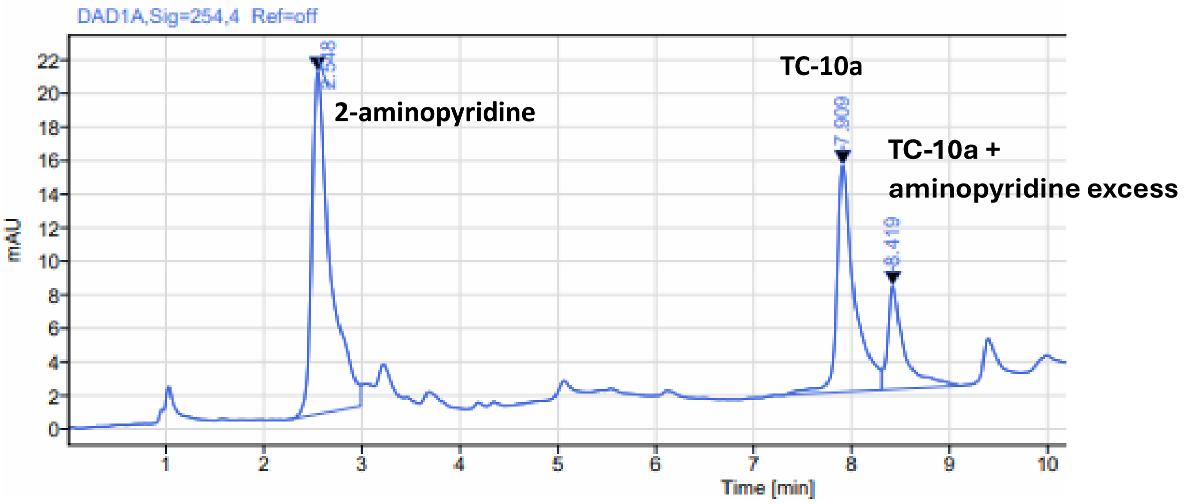

Figure S64. Crude analytical HPLC trace for product **TC-10a** with 2000 equiv amine, 2000 equiv isocyanide in MeOH/$CHCl_3$ (7:1, v/v) at 5°C (entry 6).

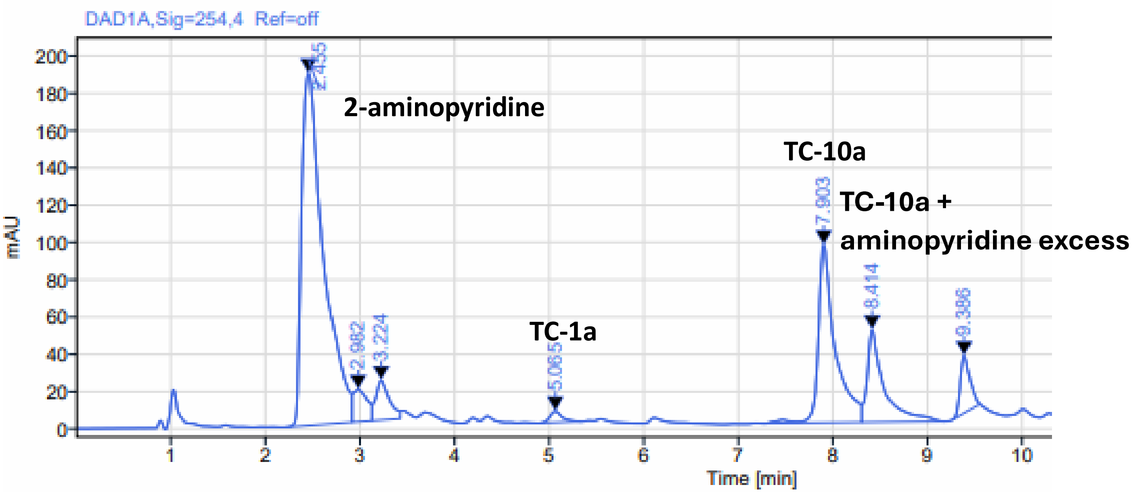

Figure S65. Crude analytical HPLC trace for product **TC-10a** with 1000 equiv amine, 1000 equiv isocyanide in EtOH/$CHCl_3$ (7:1, v/v) at rt (entry 7).

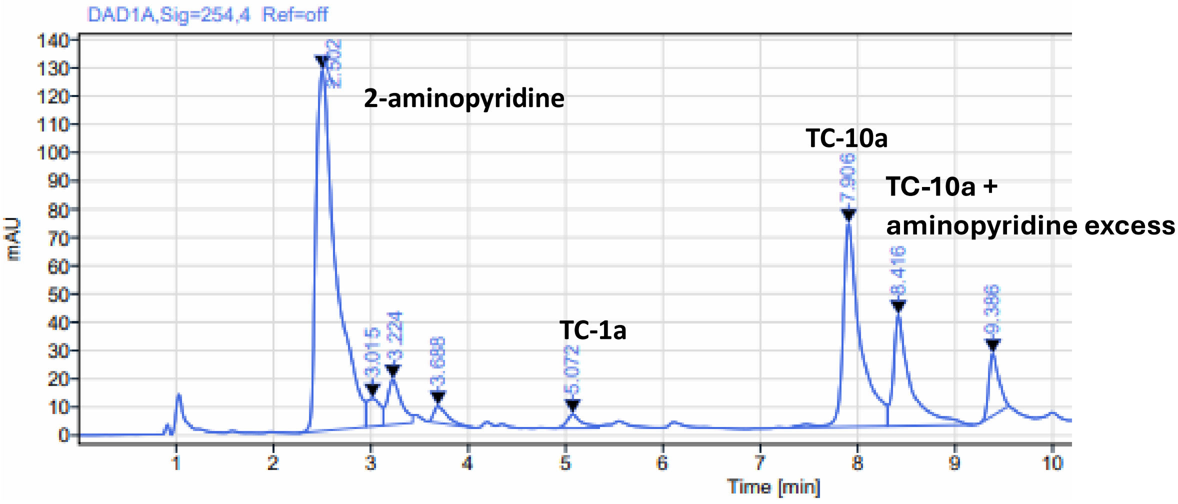

Figure S66. Crude analytical HPLC trace for product **TC-10a** with 1000 equiv amine, 1000 equiv isocyanide in iPrOH/$CHCl_3$ (7:1, v/v) at rt (entry 8).

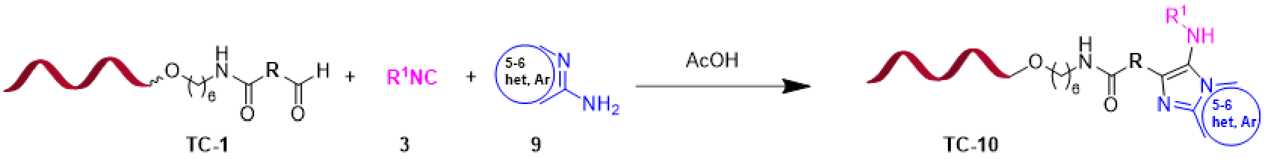

Table S9. MALDI-MS data & Product conversions of the reaction scope for the Groebke-Blackburn-Bienaymé three-component reaction with 10merTC-coupled aldehydes.

	R	R^1	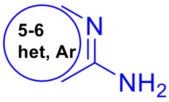	calculated	found	Product conversion
				MALDI-MS m/z		
TC-10a	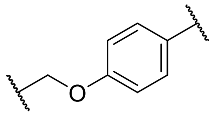	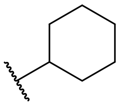	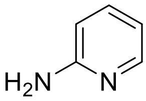	3431.0	3431.3	>95%
TC-10b	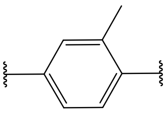	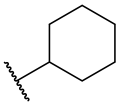	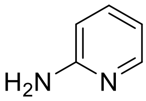	3415.0	3417.9	79%
TC-10c	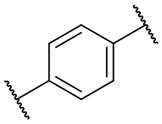	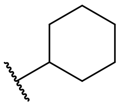	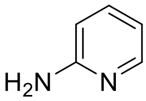	3401.0	3402.7	>95%
TC-10d	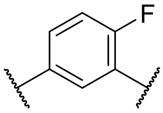	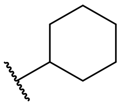	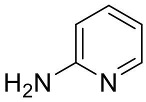	3420.0	3420.6	>95%
TC-10e	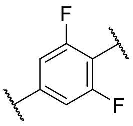	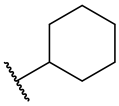	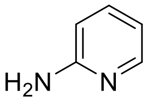	3437.0	3439.0	>95%
TC-10f	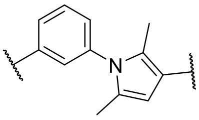	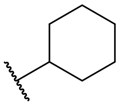	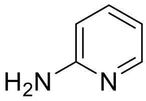	3496.0	3496.3	>95%
TC-10g	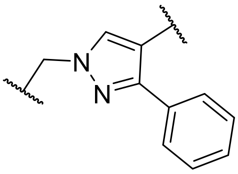	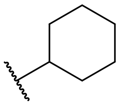	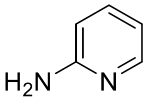	3481.0	3483.0	>95%
TC-10h	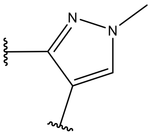	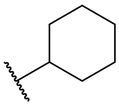	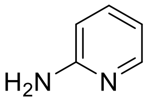	3405.0	3406.5	>95%
TC-10i	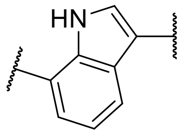	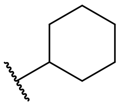	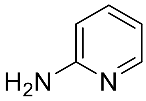	3441.0	3442.0	>95%

TC-10j	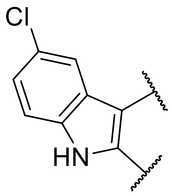	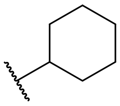	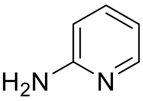	3475.0	3476.5	>95%
TC-10k	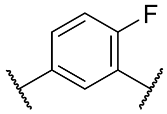	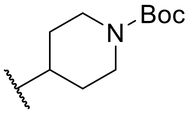	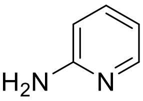	3520.0	3521.8	>95%
TC-10l	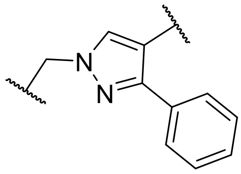	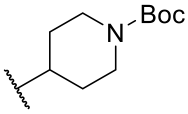	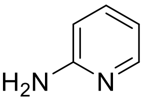	3582.0	3583.8	82%
TC-10m	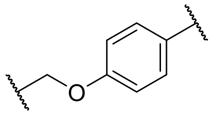	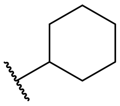	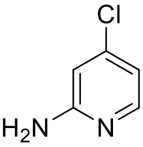	3465.0	3465.7	>95%
TC-10n	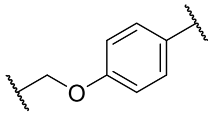	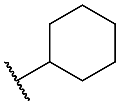	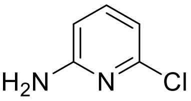	3465.0	3465.1	>95%
TC-10o	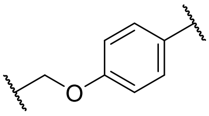	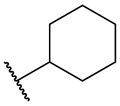	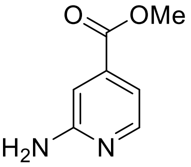	3489.0	3489.5	>95%
TC-10p	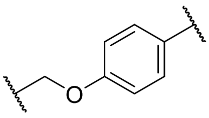	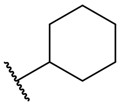	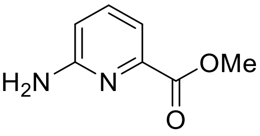	3489.0	3489.5	>95%
TC-10q	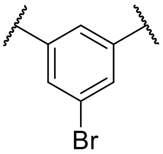	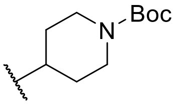	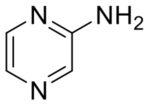	3582.0	3582.5	90,5%
TC-10r	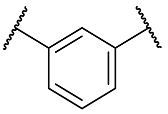	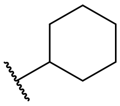	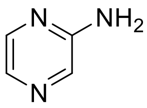	3402.0	3403.9	69%
TC-10s	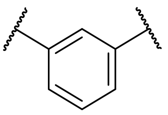	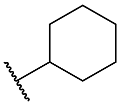	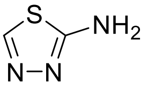	3408.0	3467.9 [M+K$^+$+NH$_4^+$]	>95%
TC-10t	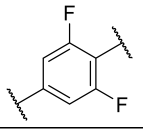	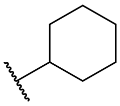	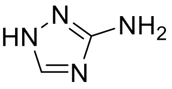	3427.0	3504.0 [M+2K$^+$]	>95%

TC-10u	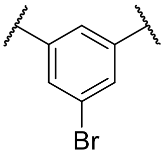	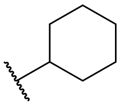	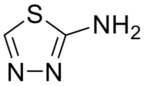	3486.0	3545.8 [M+ K^{+}+NH_4^{+}]	>95%
TC-10v	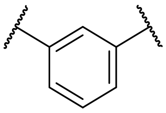	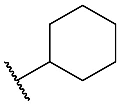	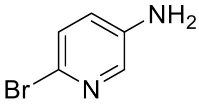	3480.0	3577.9 [M + TEA]	>95%
TC-10w	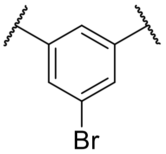	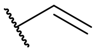	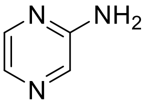	3670	n.d.	n.d.

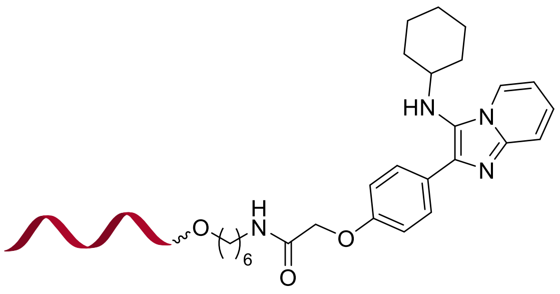

TC-10a

MS calc. 3434.0; found: 3431.3

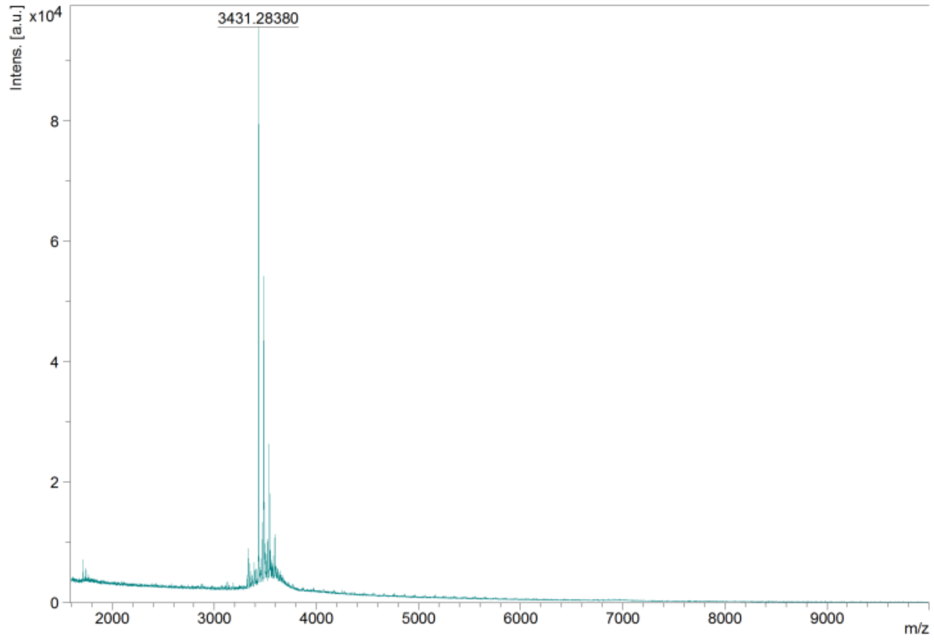

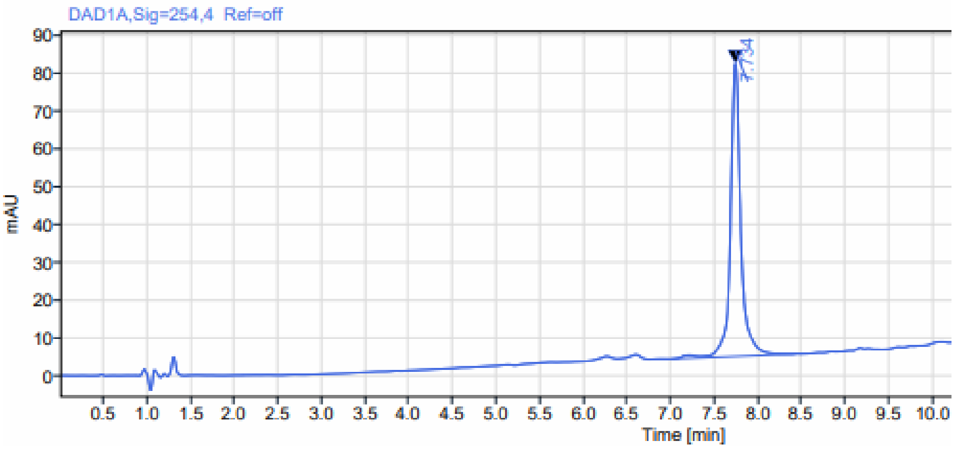

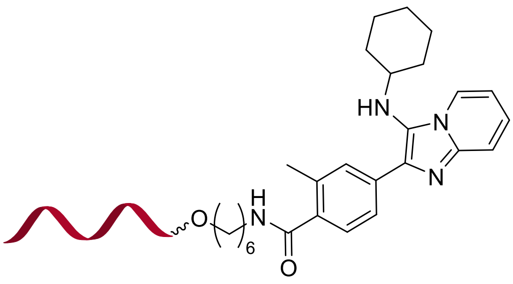

TC-10b

MS calc. 3415.0; found: 3416.9

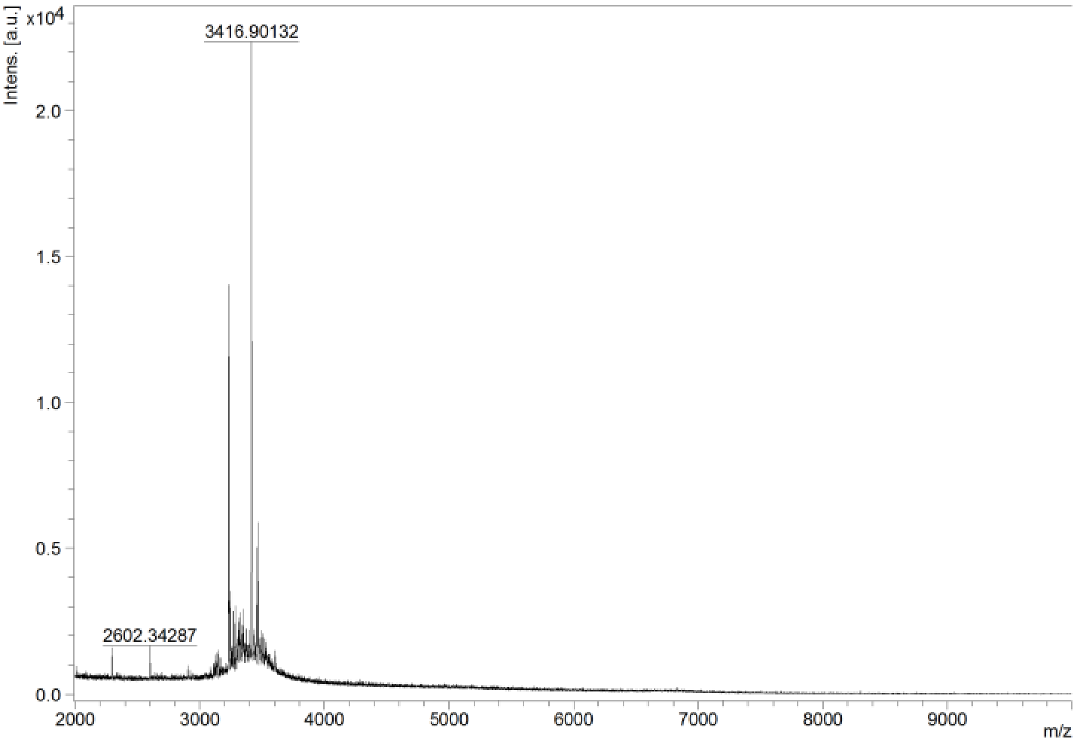

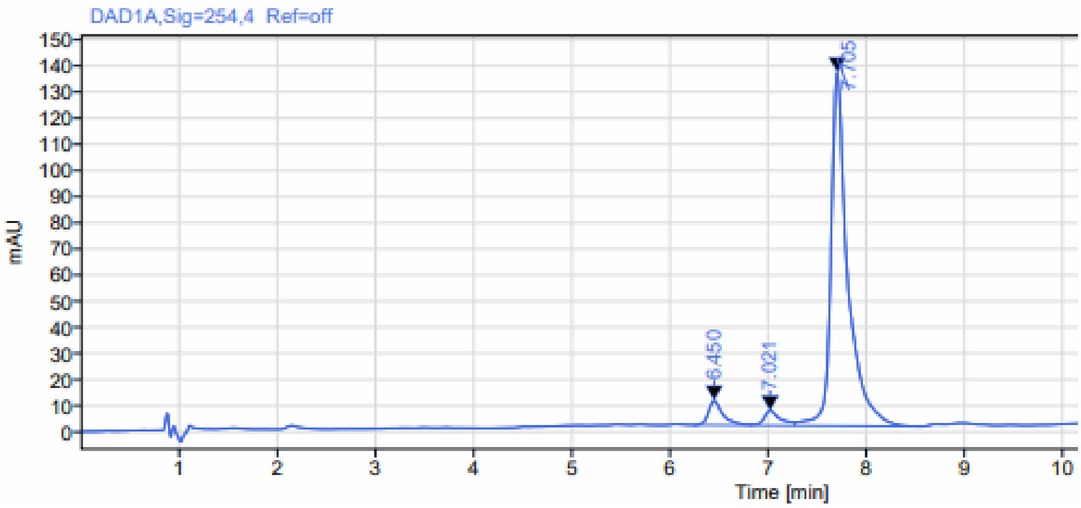

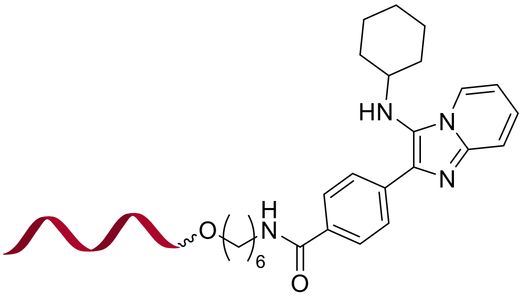

TC-10c

MS calc. 3401.0; found: 3402.7

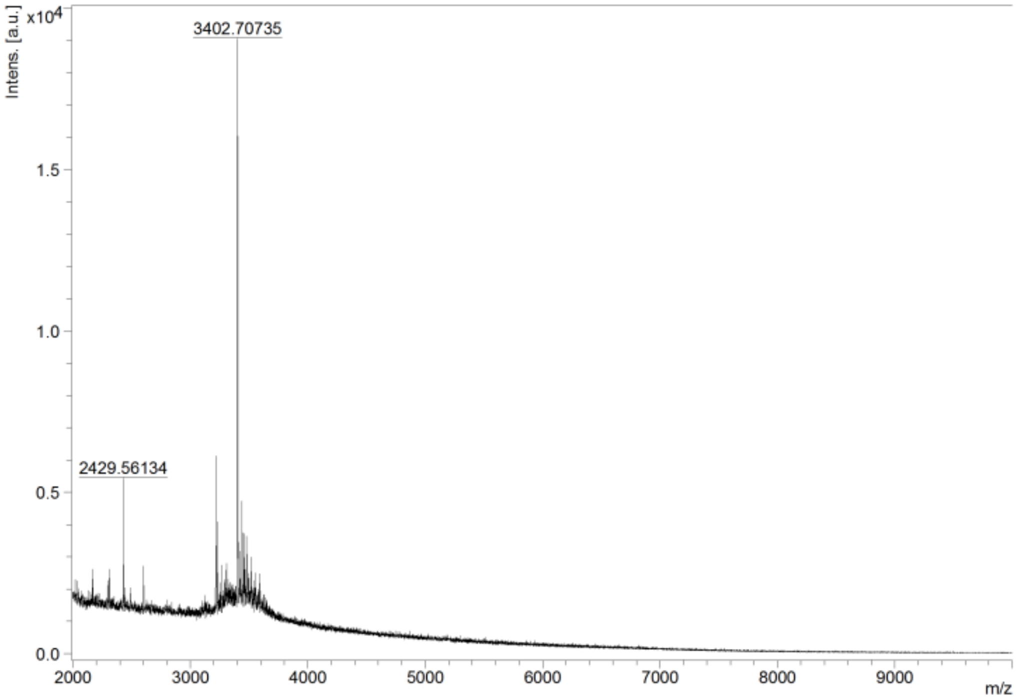

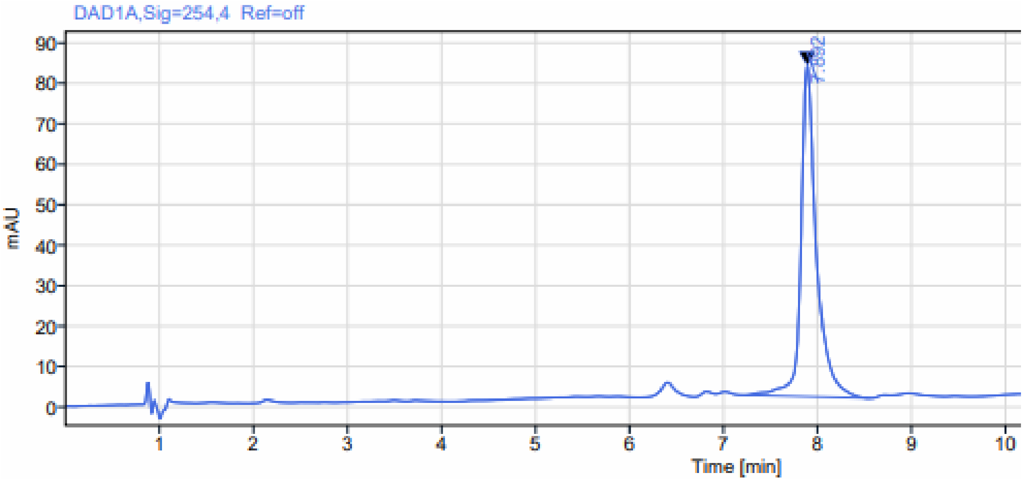

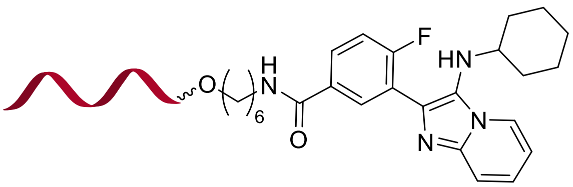

TC-10d

MS calc. 3420.0; found: 3420.6

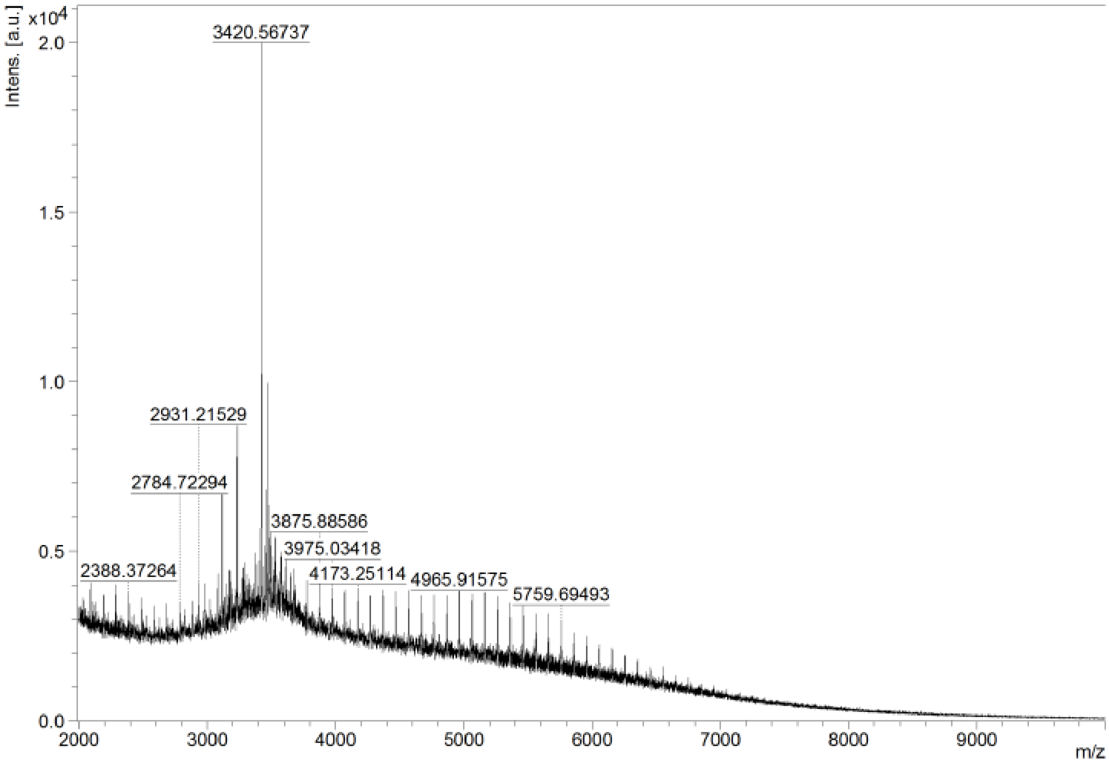

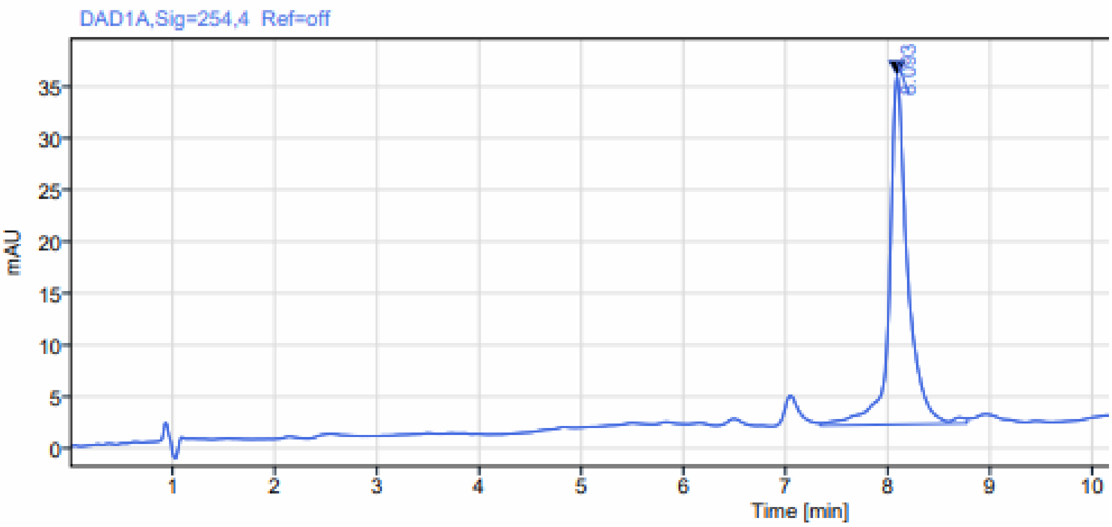

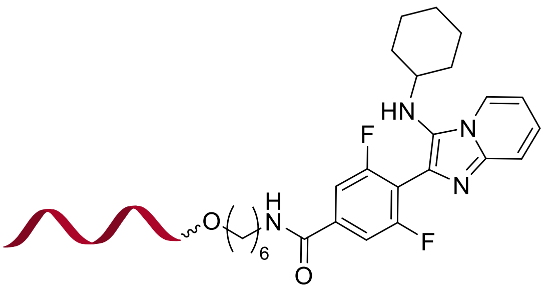

TC-10e

MS calc. 3437.0; found: 3439.0

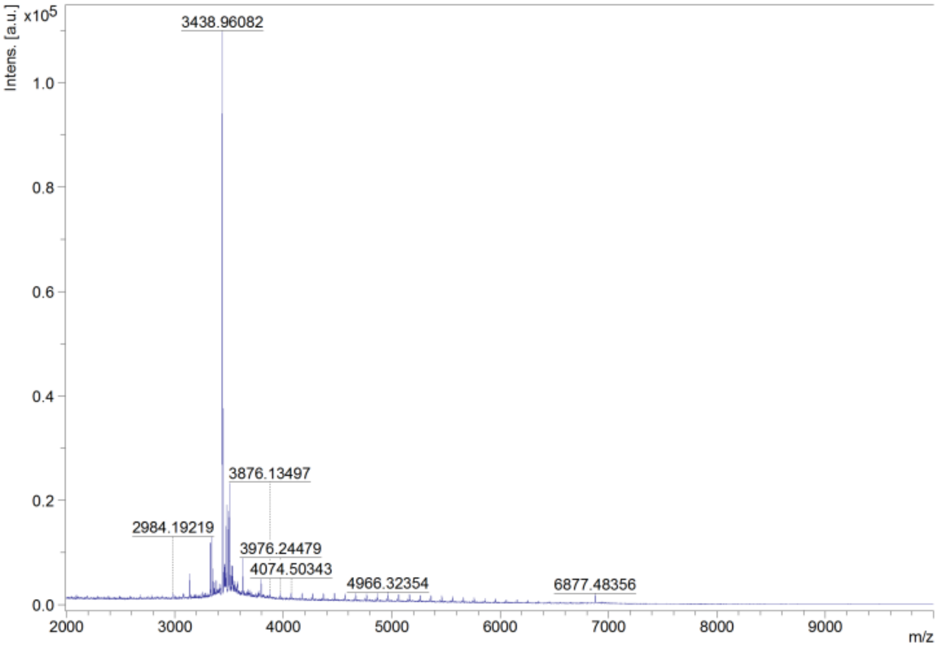

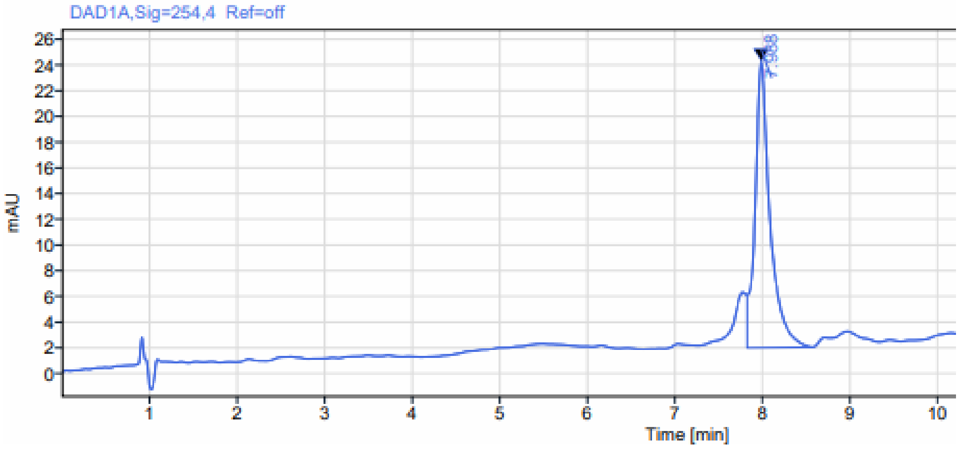

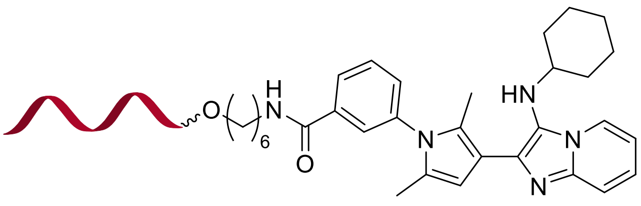

TC-10f

MS calc. 3496.0; found: 3496.3

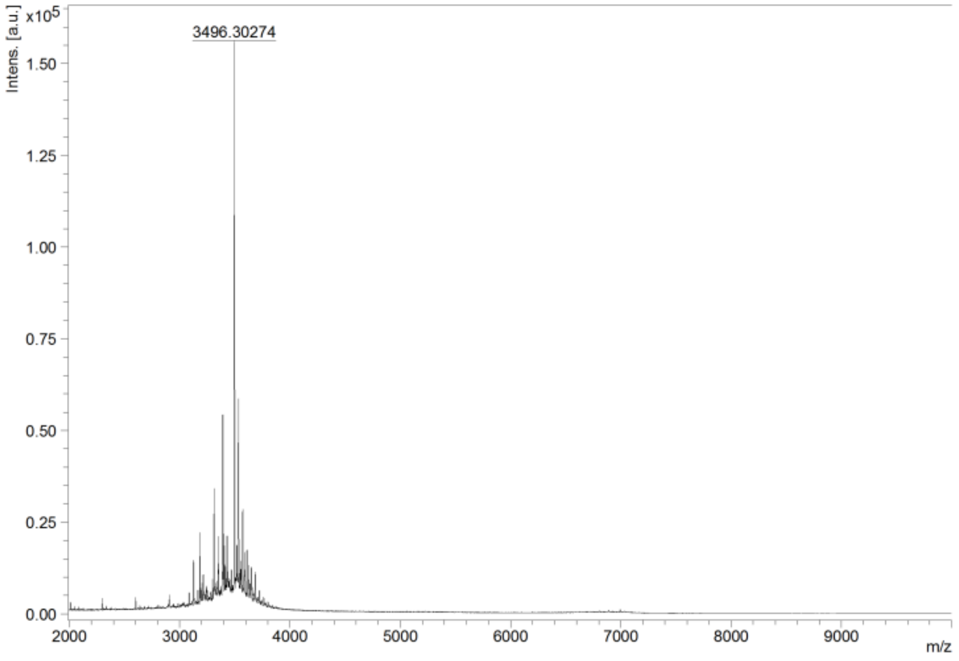

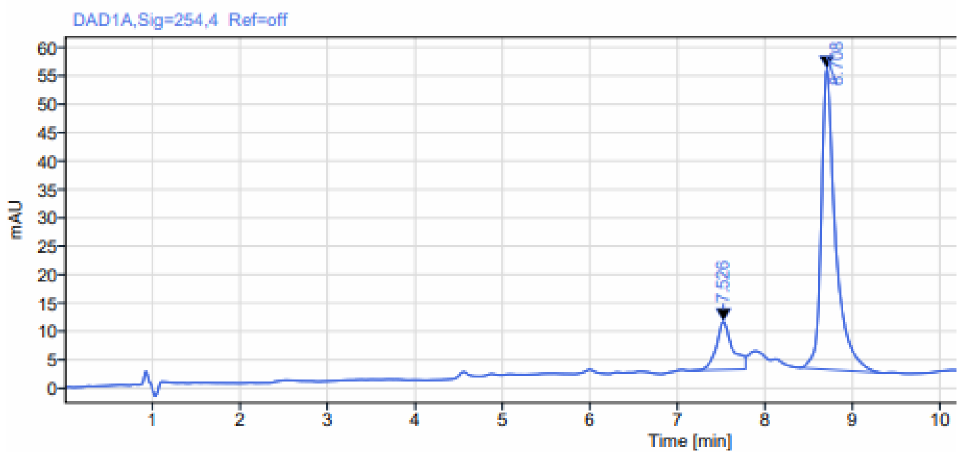

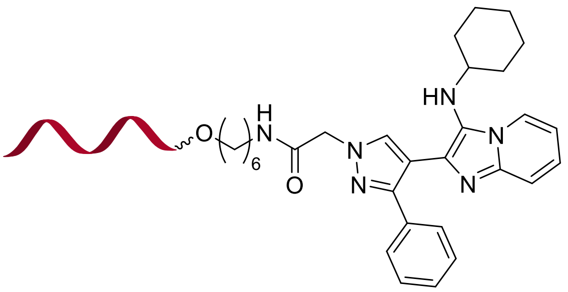

TC-10g

MS calc. 3481.0; found: 3483.0

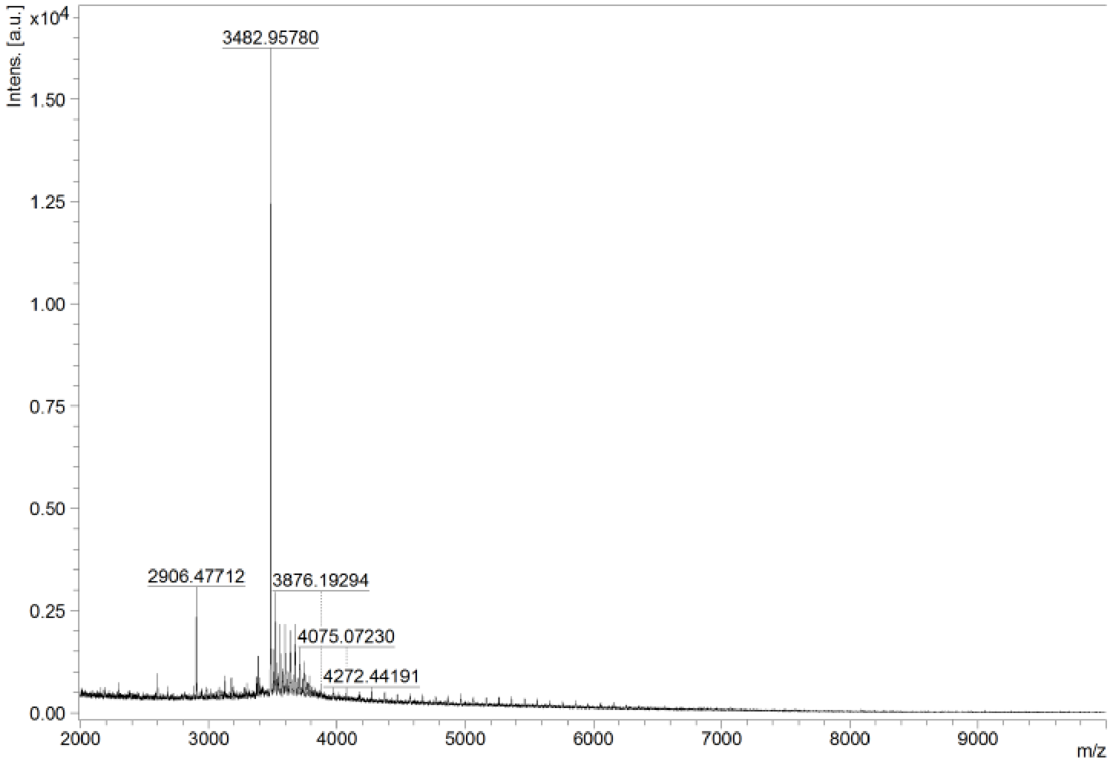

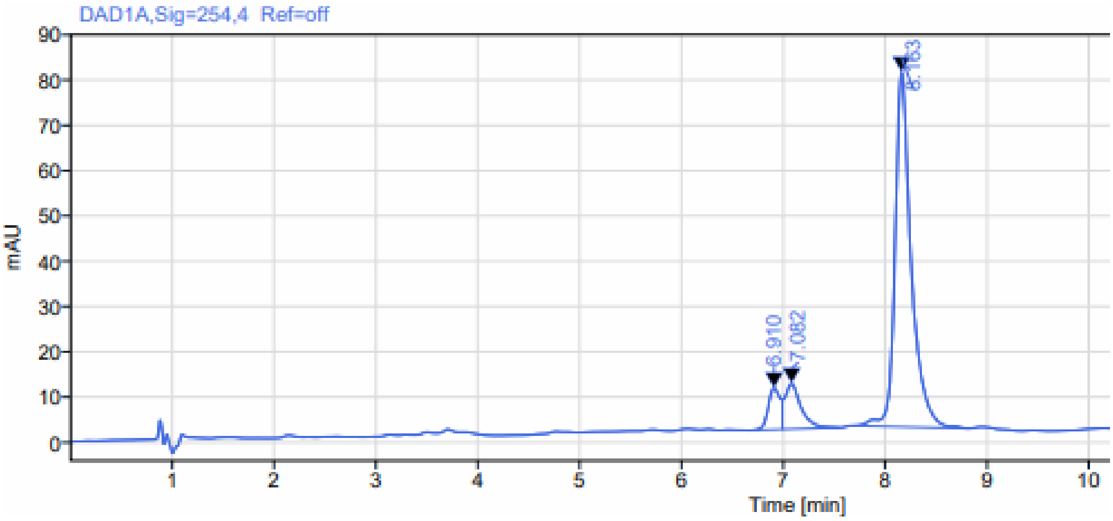

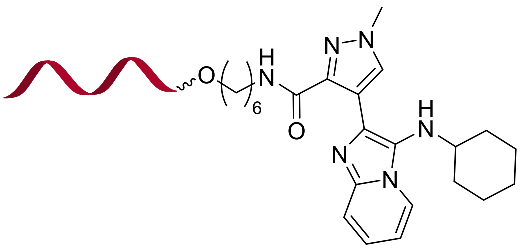

TC-10h

MS calc. 3405.0; found: 3406.5

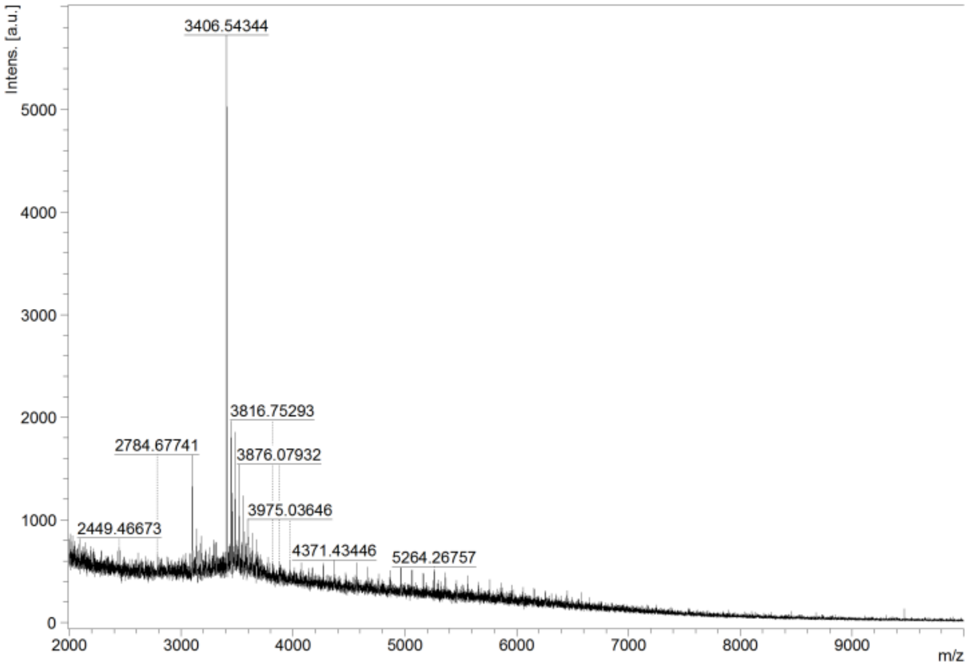

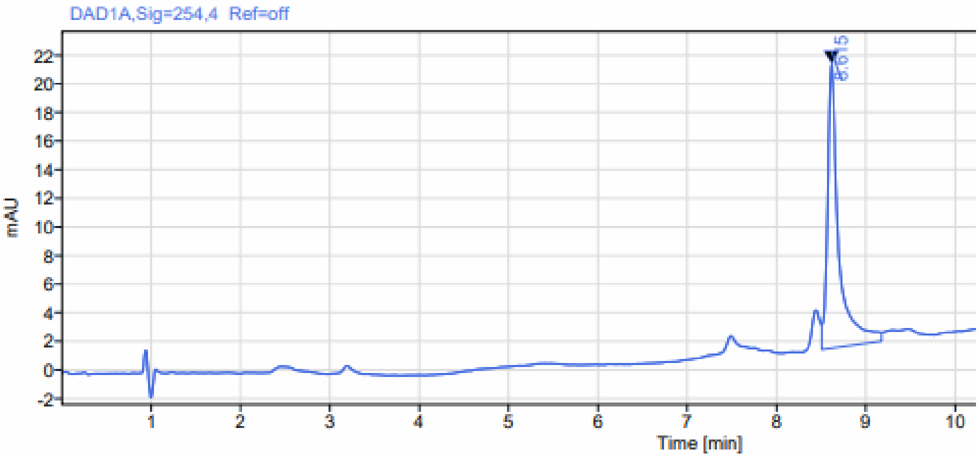

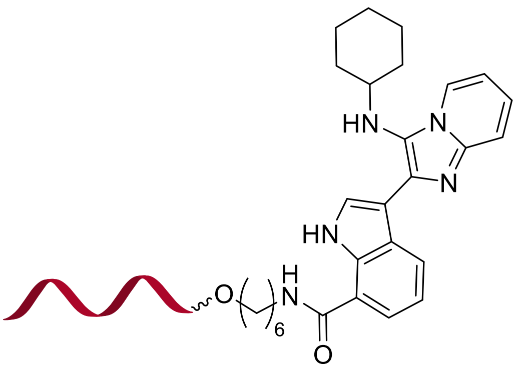

TC-10i

MS calc. 3441.0; found: 3442.0

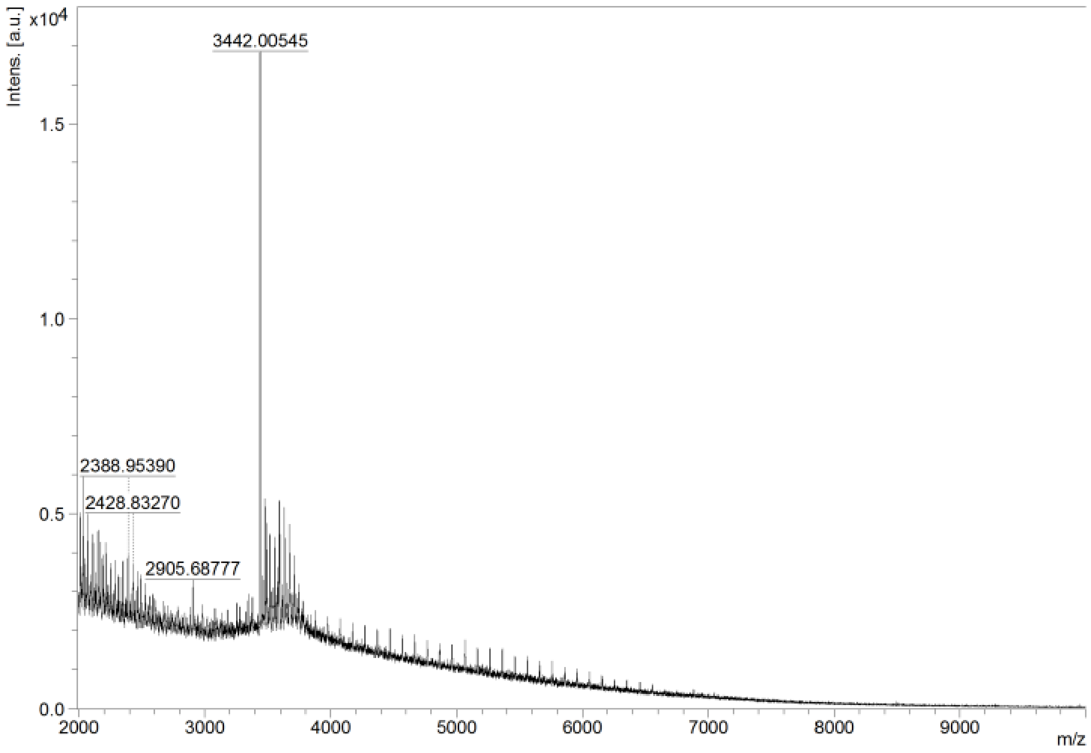

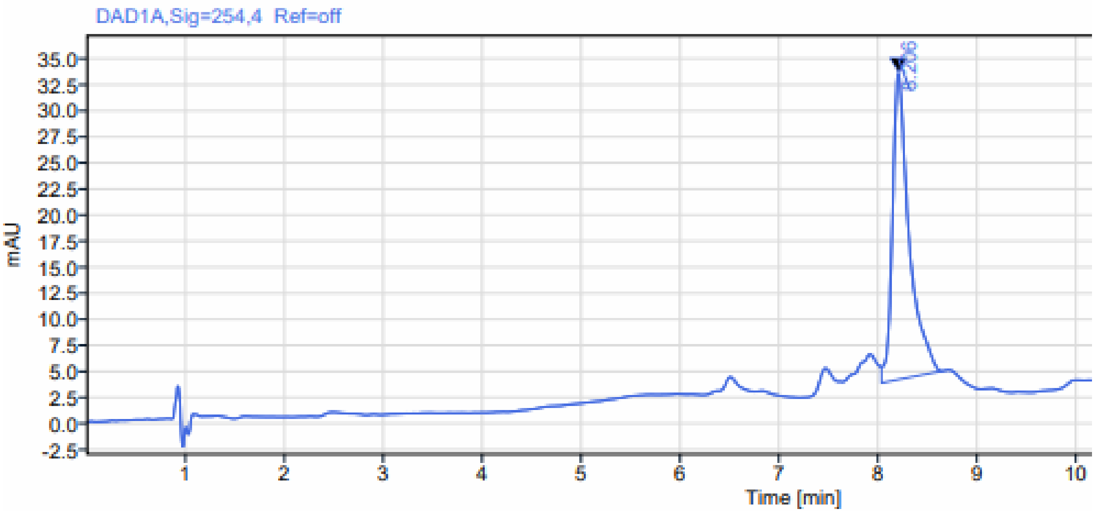

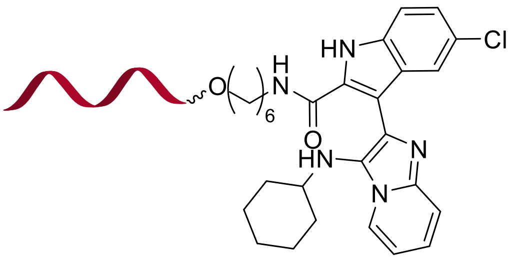

TC-10j

MS calc. 3475.0; found: 3476.5

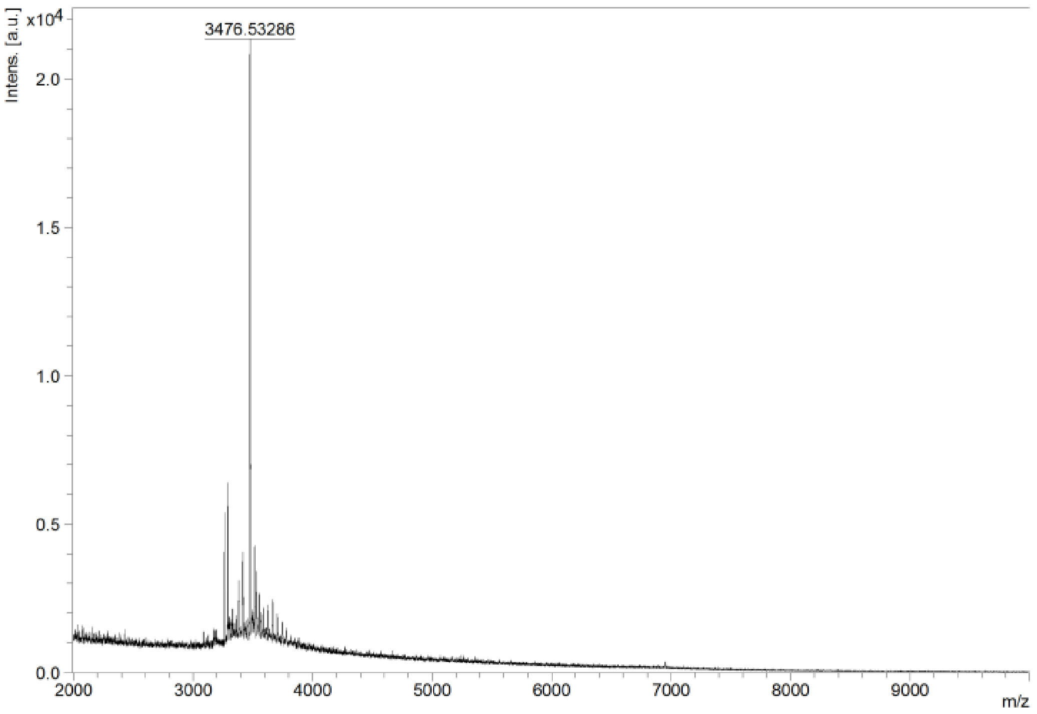

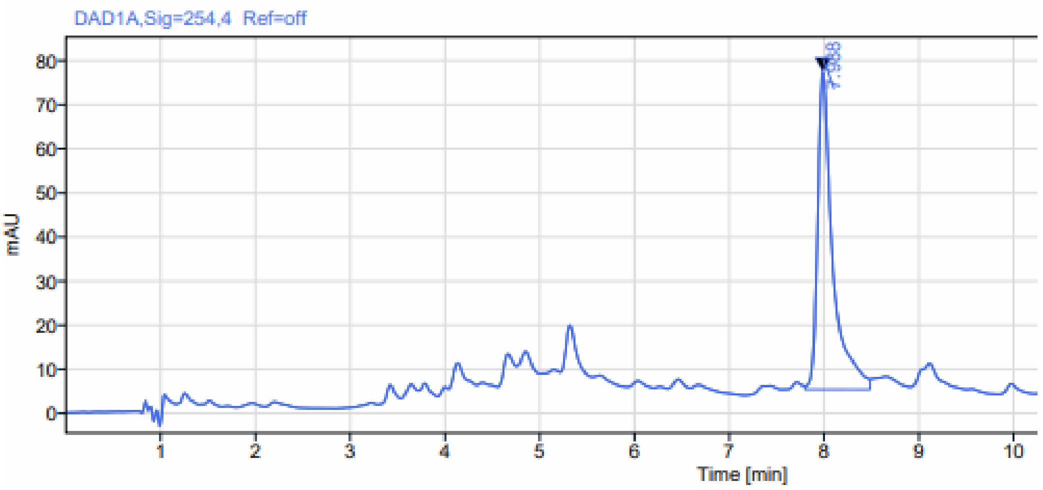

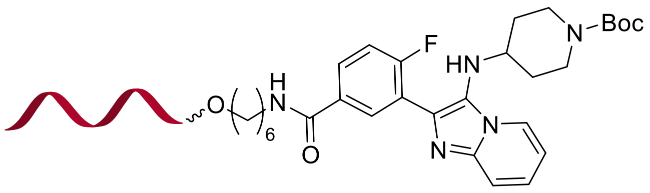

TC-10k

MS calc. 3520.0; found: 3521.8

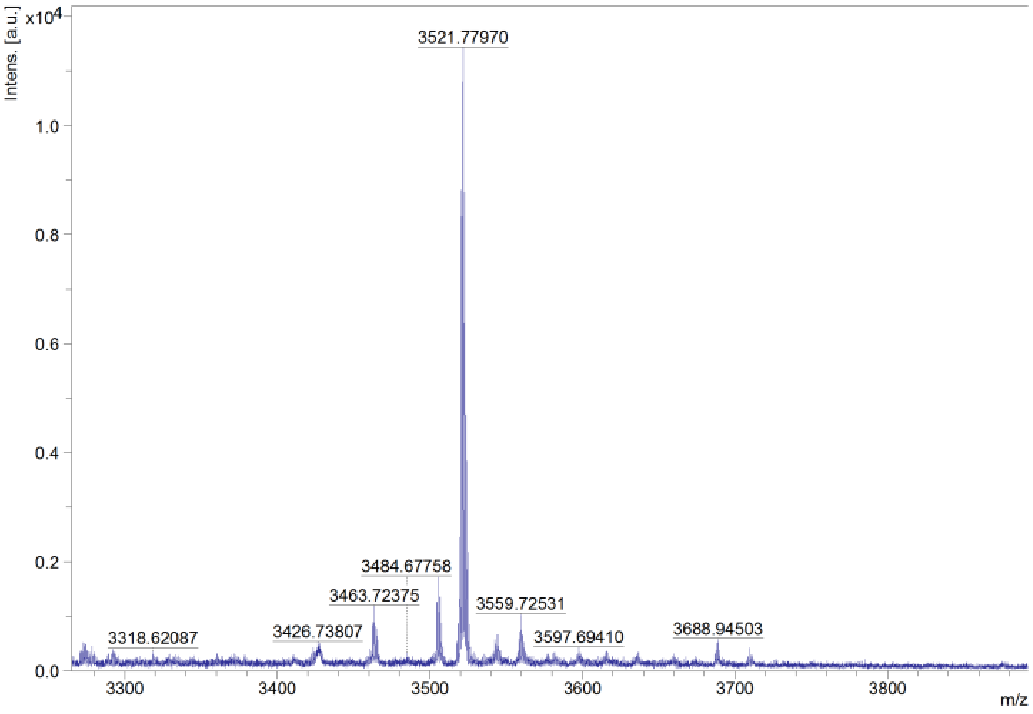

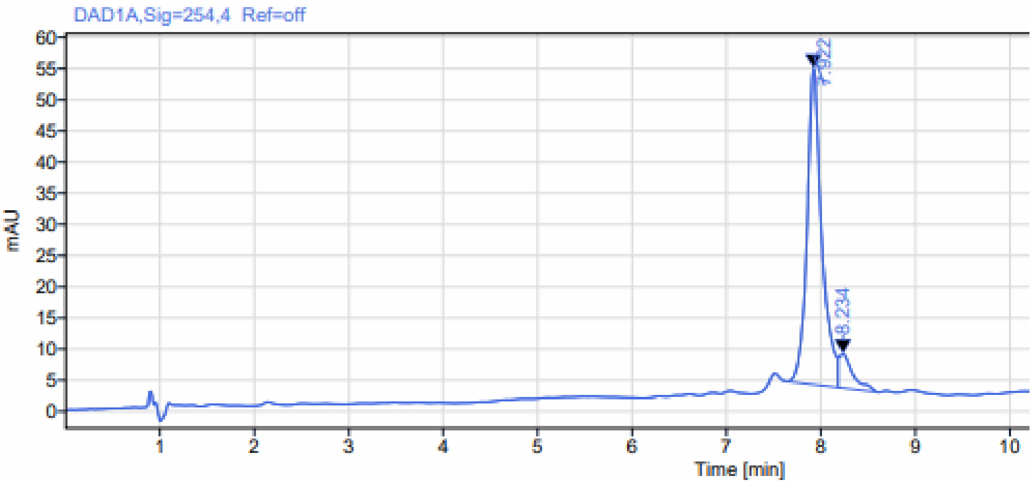

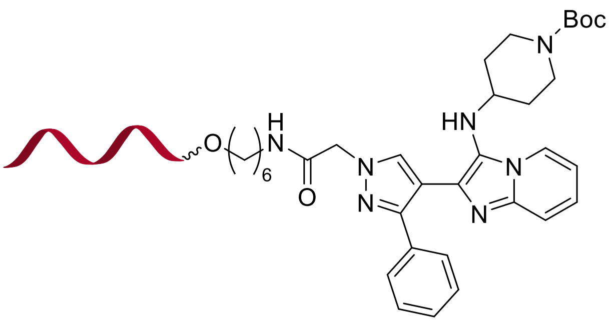

TC-10l

MS calc. 3582.0; found: 3583.8

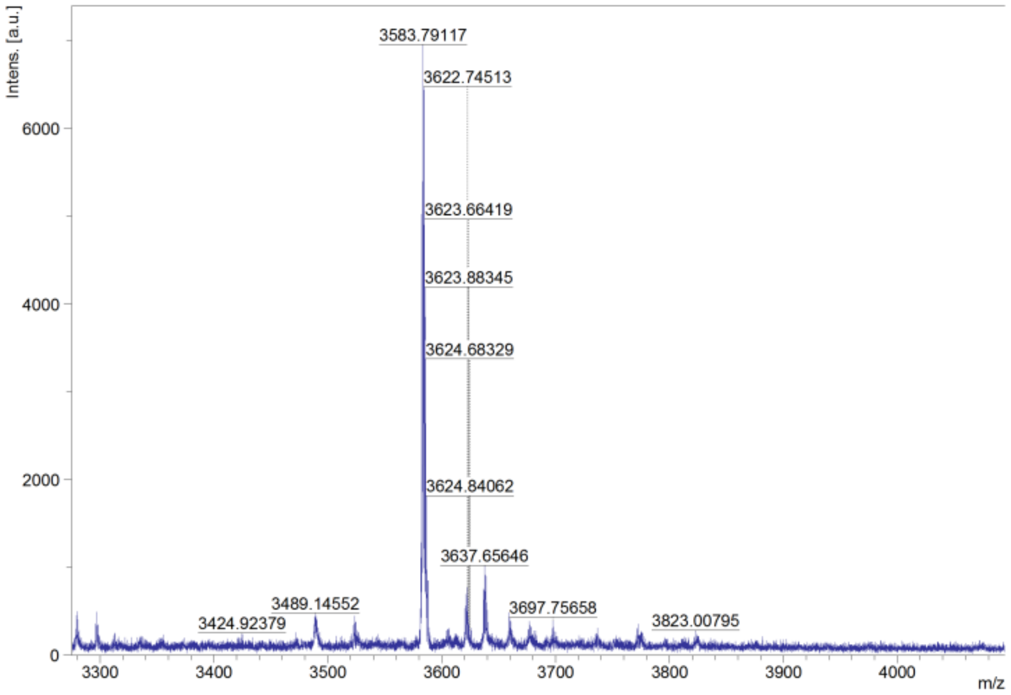

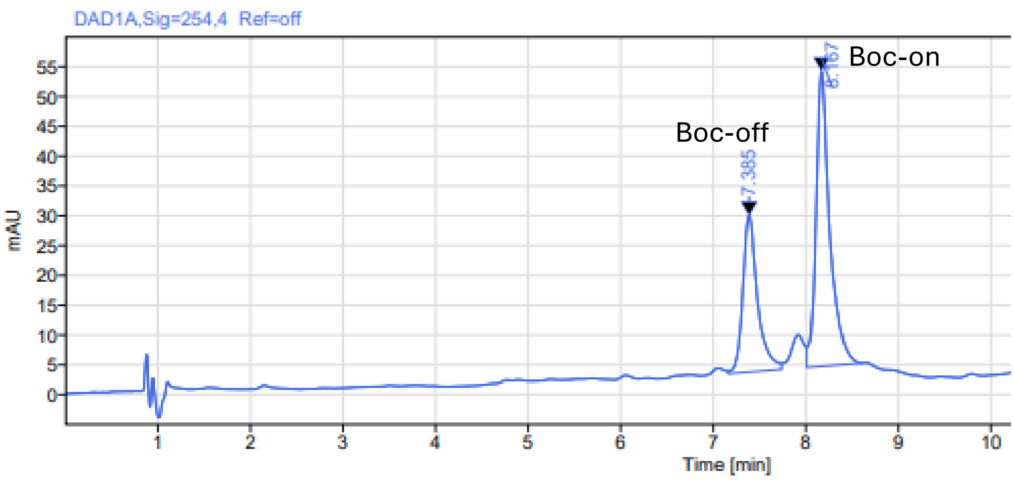

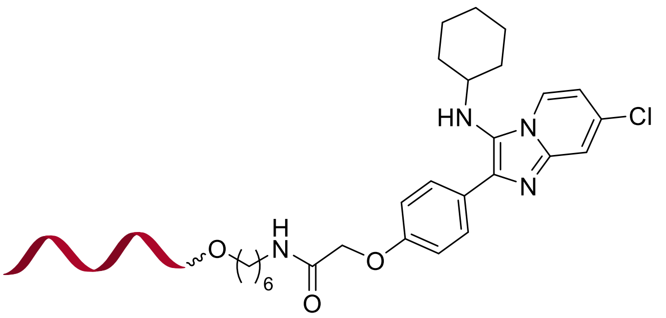

TC-10m

MS calc. 3465.0; found: 3465.7

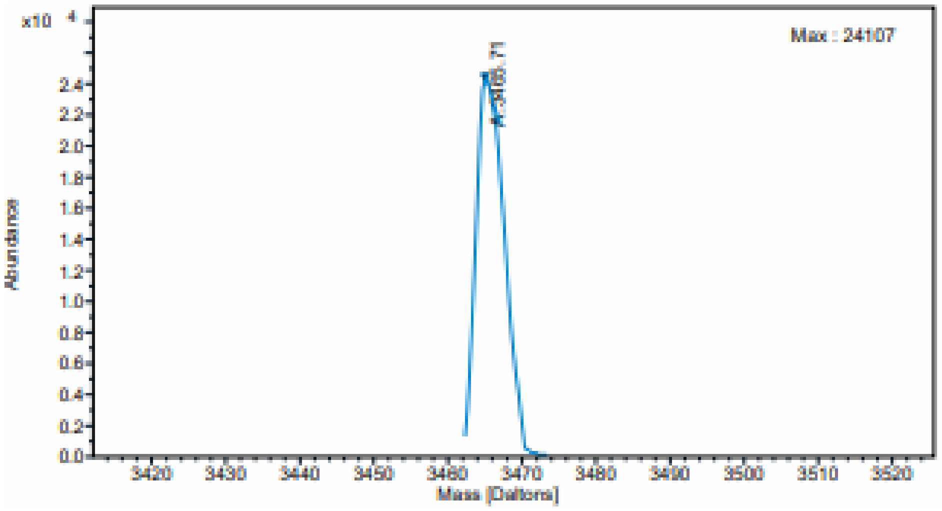

measured by LC-MS

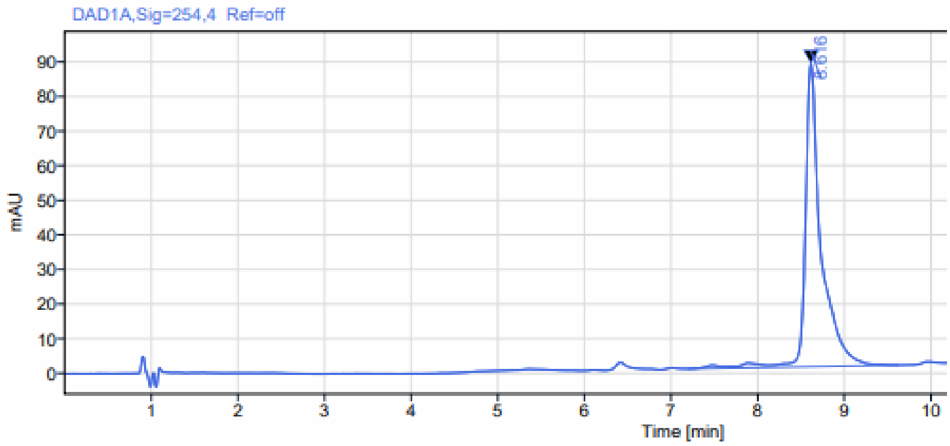

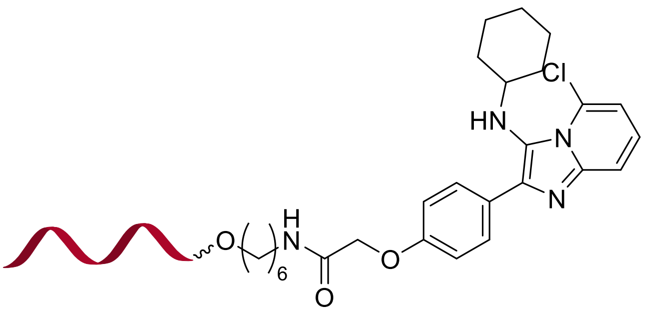

TC-10n

MS calc. 3465.0; found: 3465.1

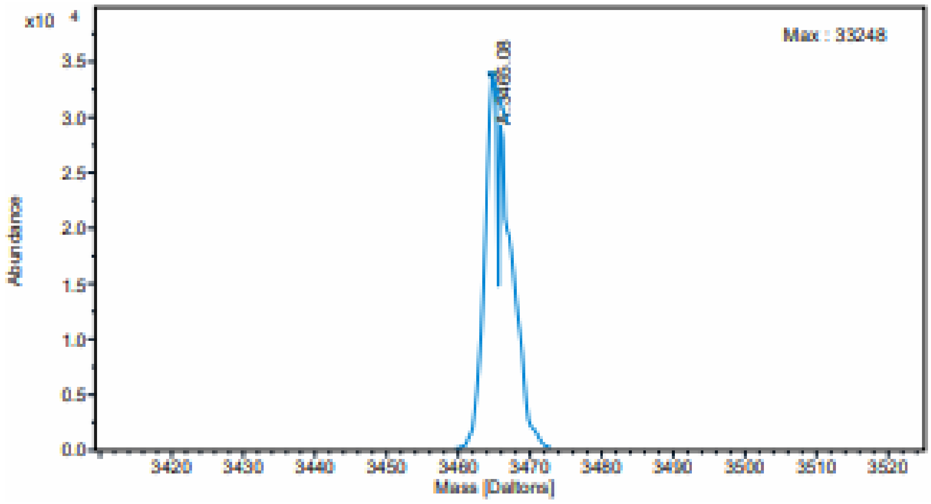

measured by LC-MS

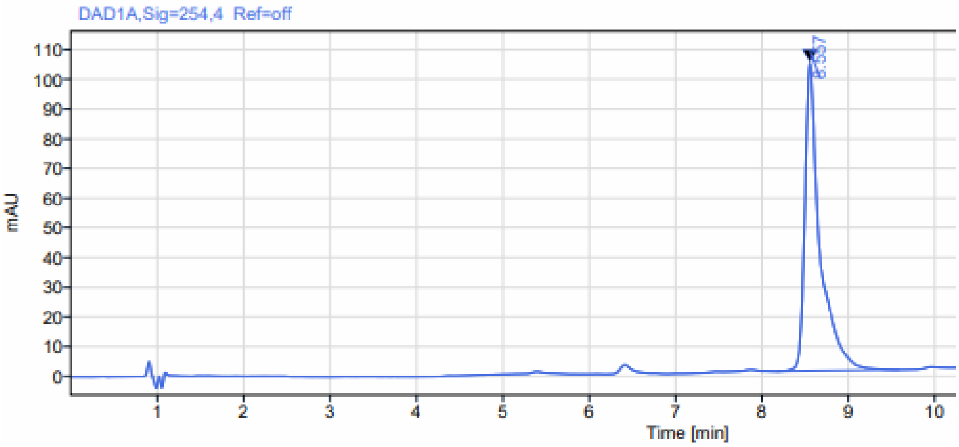

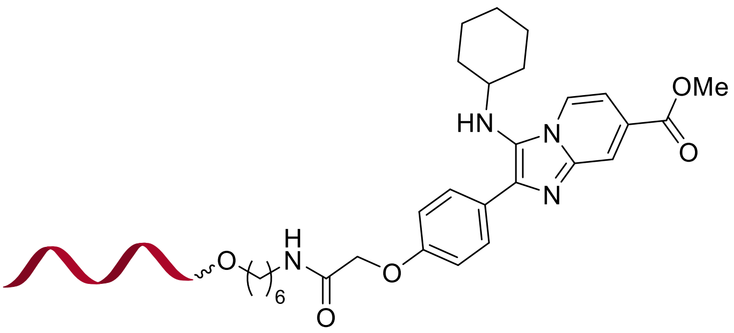

TC-10o

MS calc. 3489.0; found: 3489.5

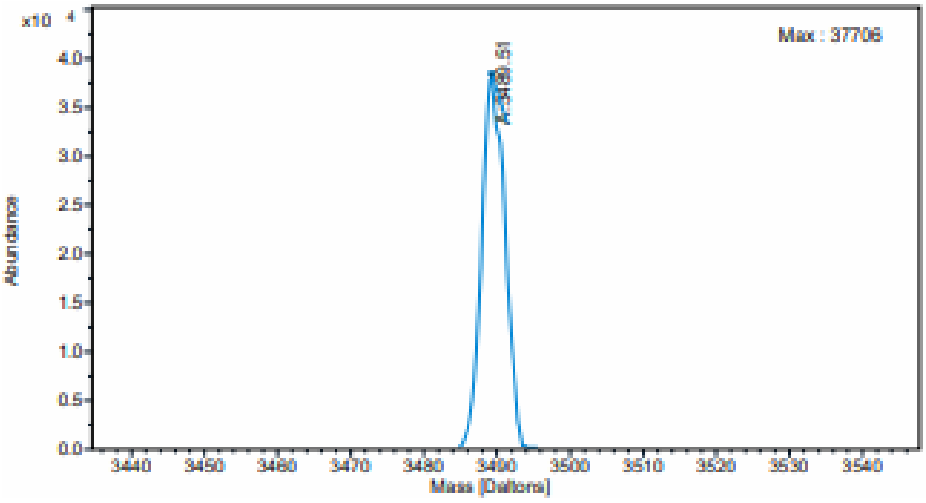

measured by LC-MS

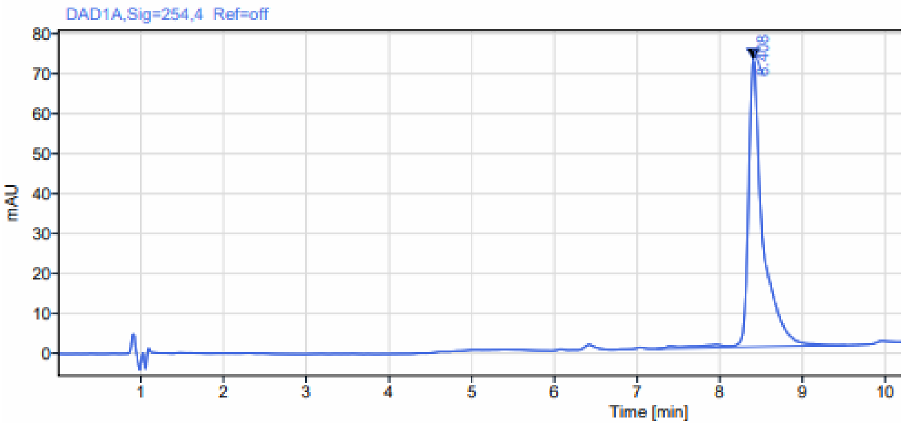

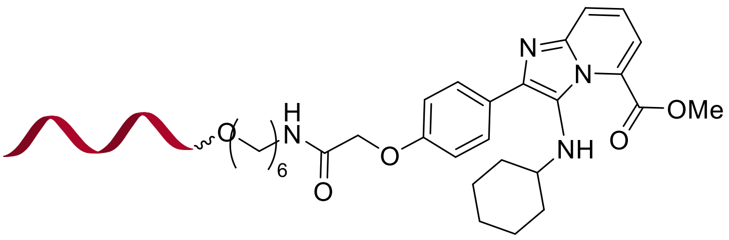

TC-10p

MS calc. 3489.0; found: 3489.5

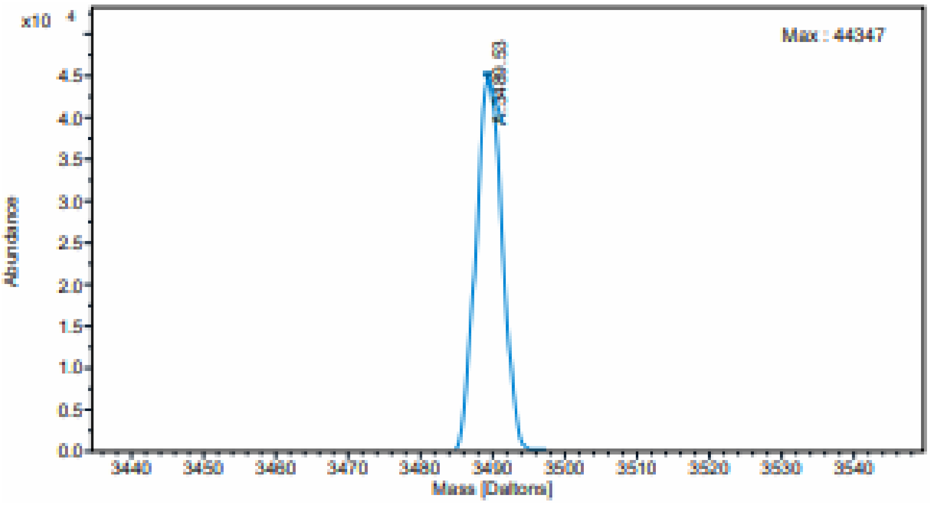

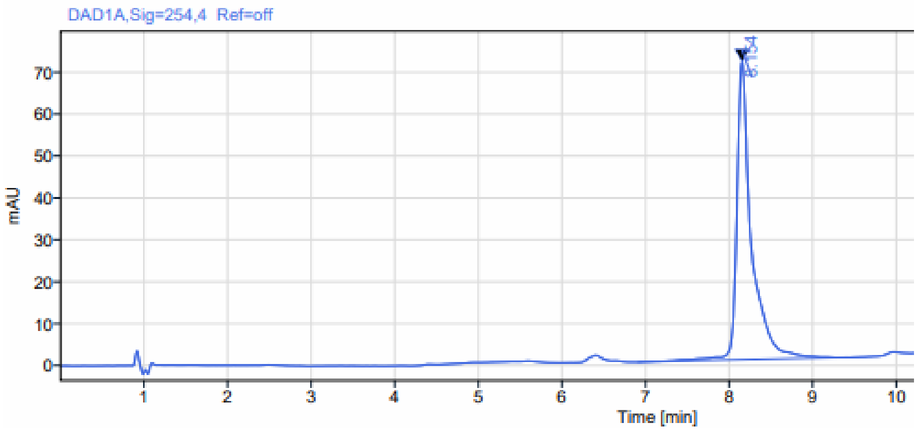

measured by LC-MS

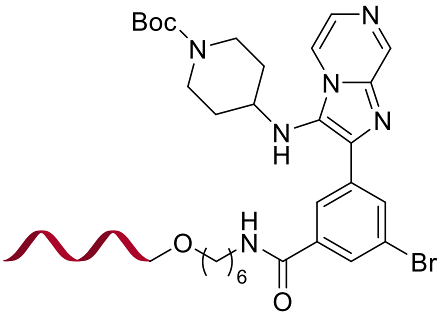

TC-10q

MS calc. 3582.0; found: 3583.7

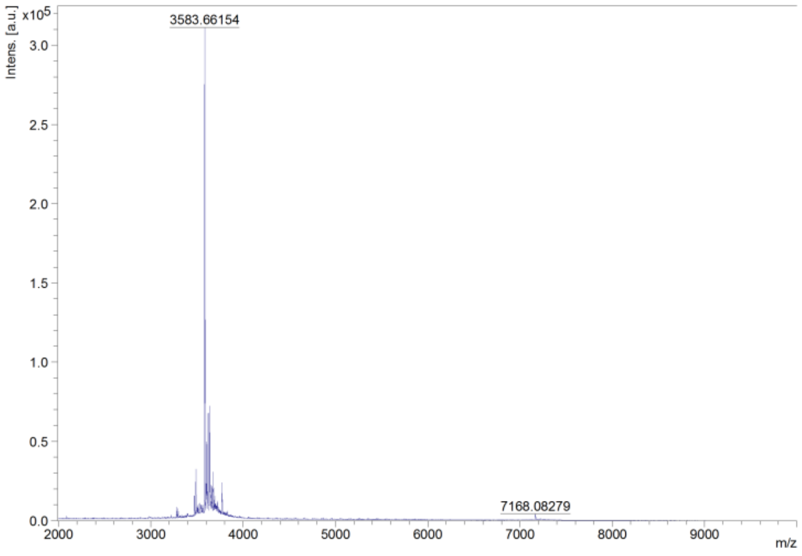

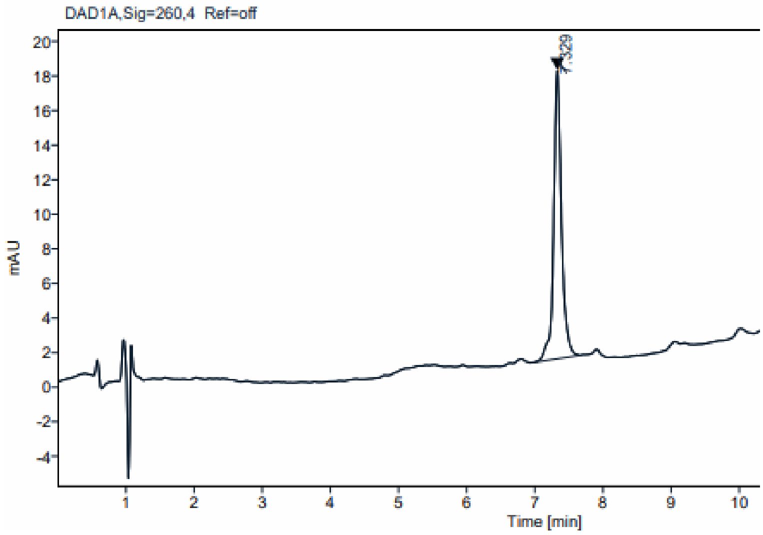

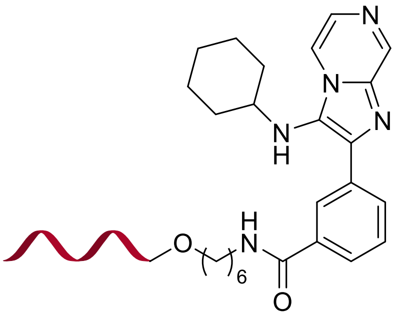

TC-10r

MS calc. 3402.0; found: 3403.9

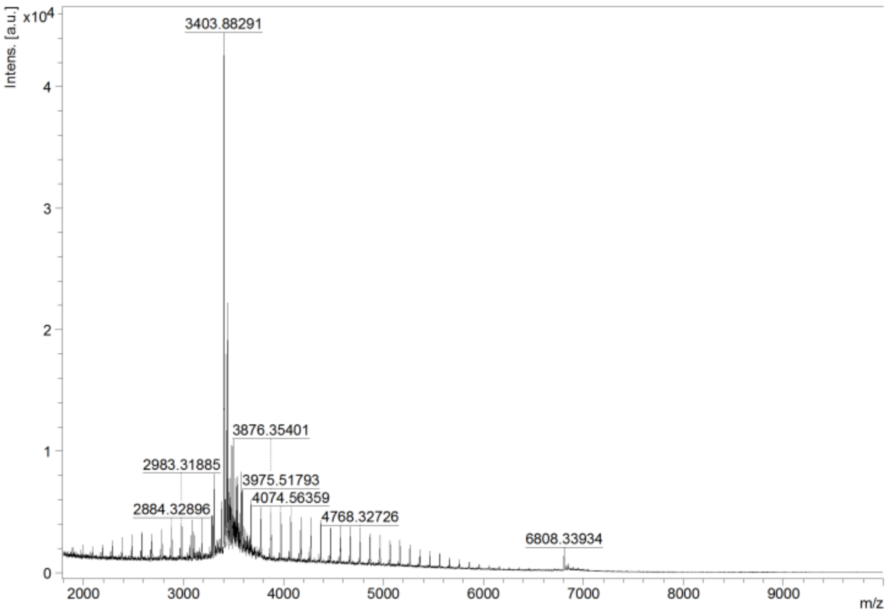

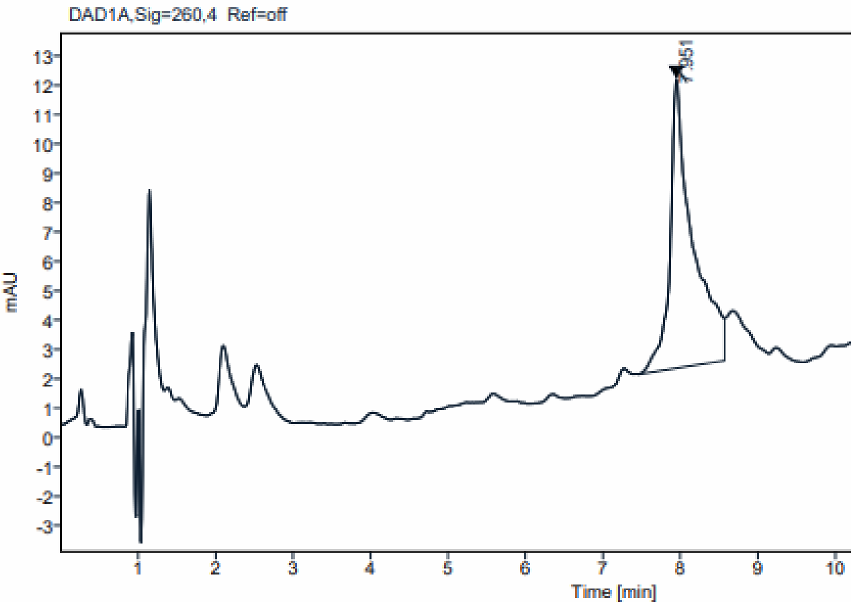

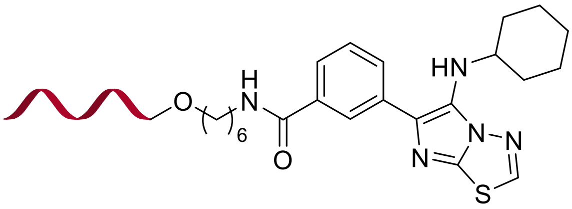

TC-10s

MS calc. 3407.0; found 3467.9 [M+K^++NH_4^+]

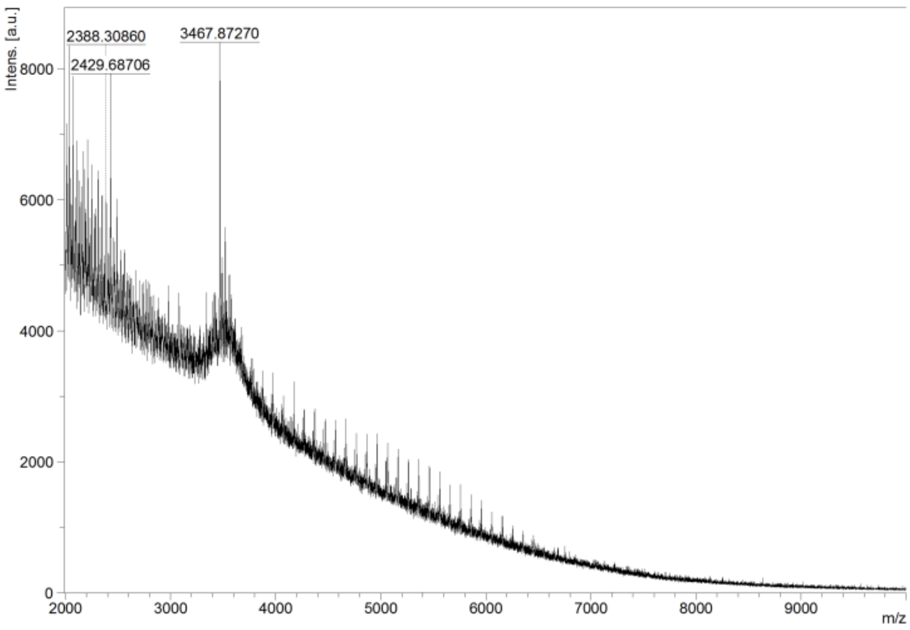

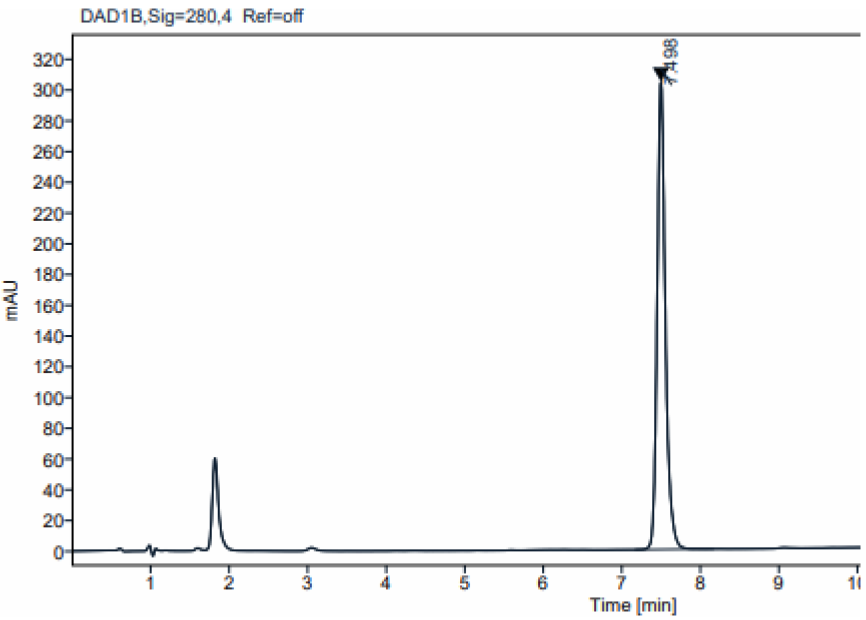

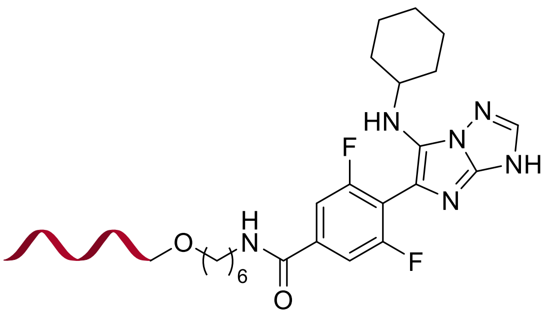

TC-10t

MS calc. 3427.0; found: 3504.0 [M+2K$^+$]

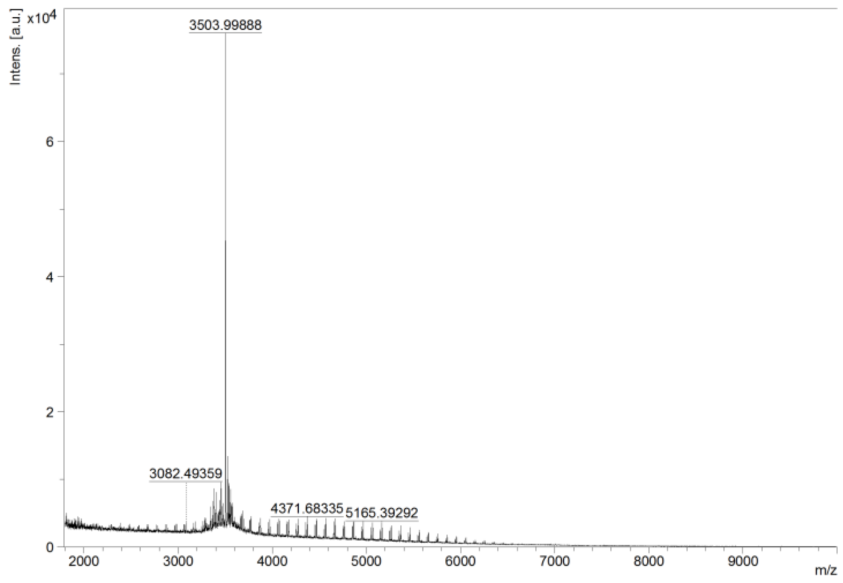

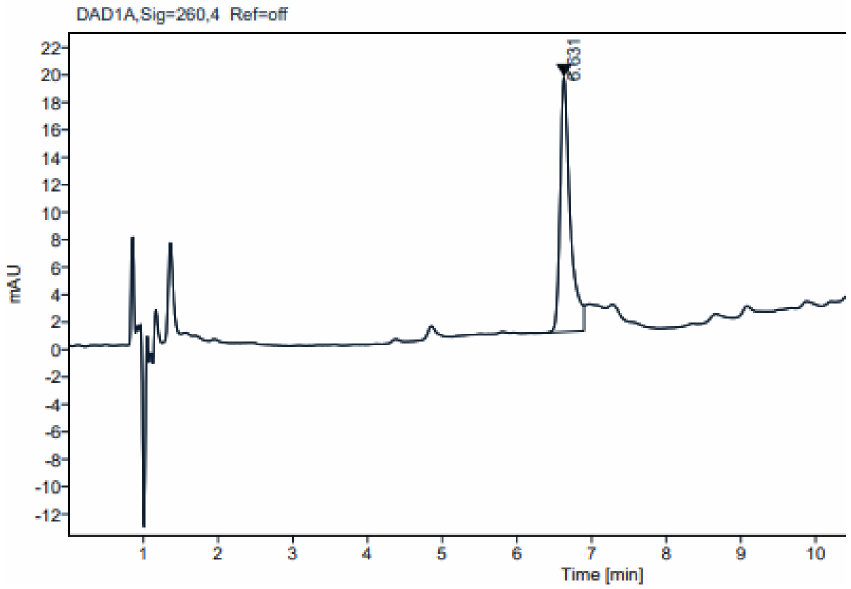

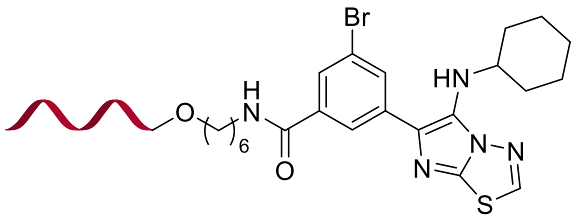

TC-10u

MS calc. 3486.0; found 3546.3 [M+K$^+$+NH$_4^+$]

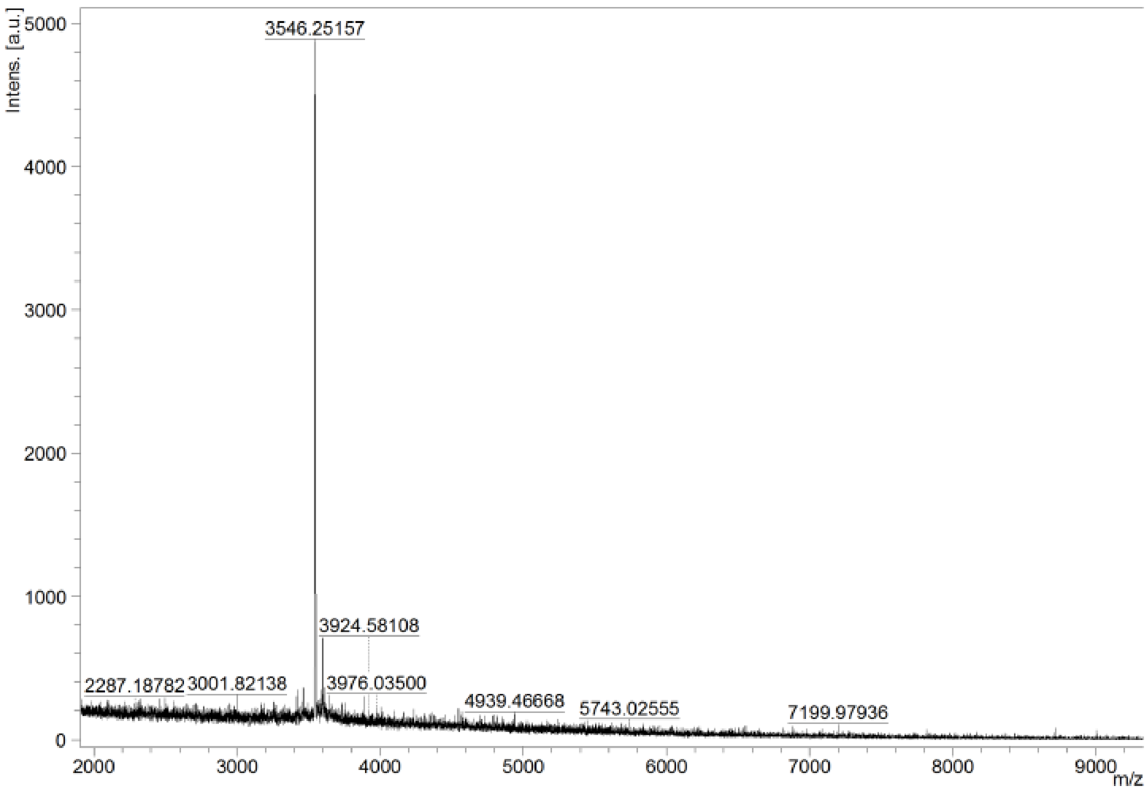

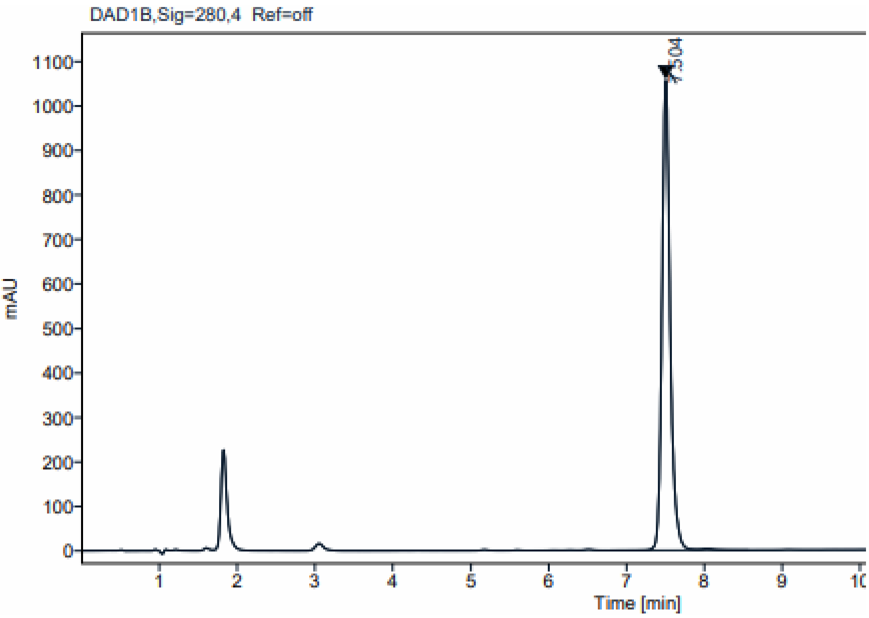

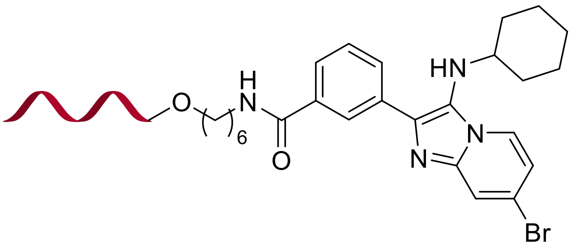

TC-10v

MS calc. 3480.0; found: 3577.9 [M+TEA]

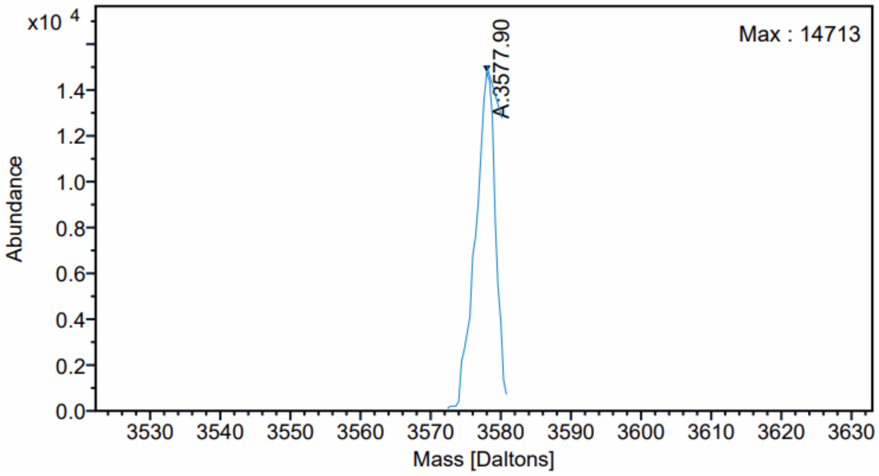

measured by LC-MS

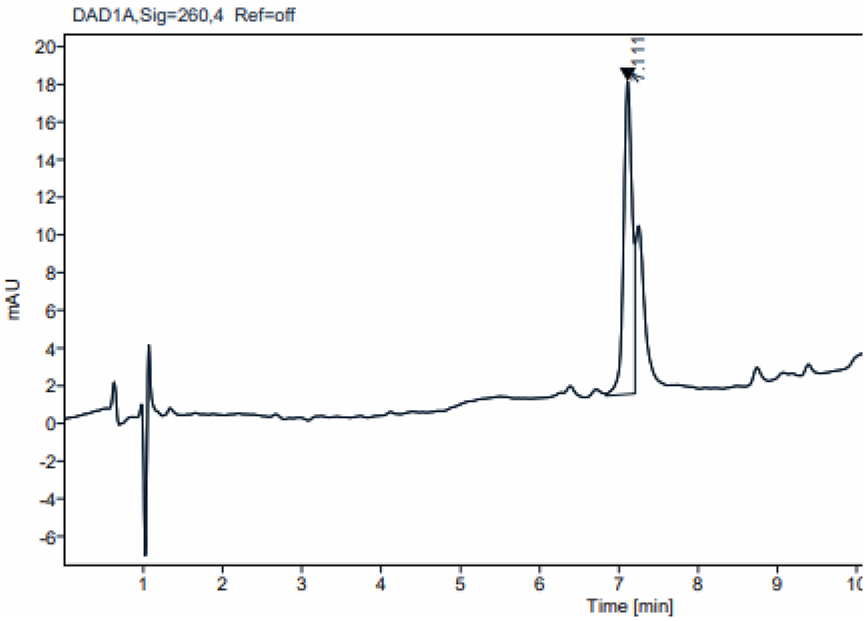

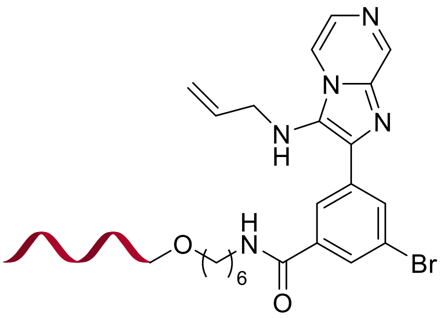

TC-10w

Mass not detected

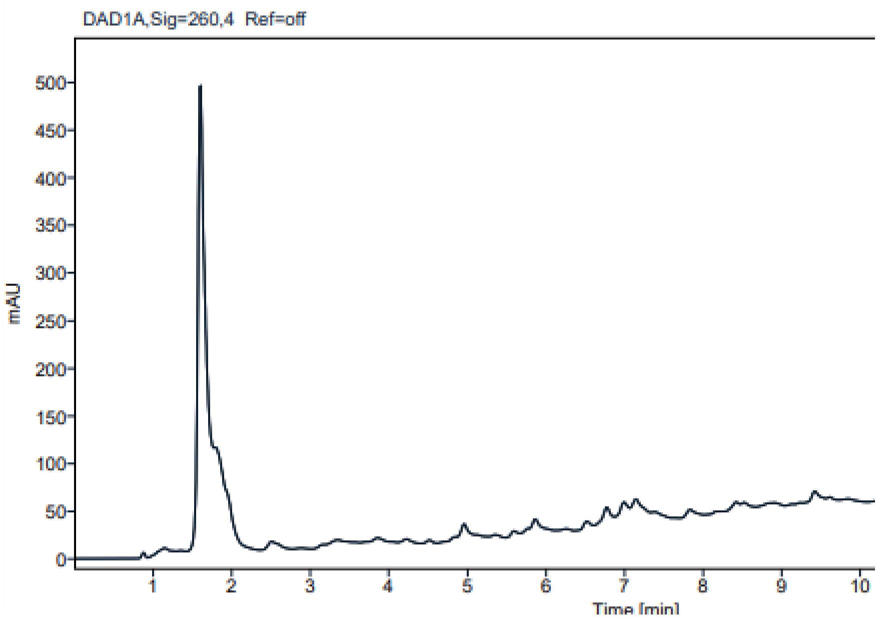

HPLC traces of representative examples of crude GBB-3CR products

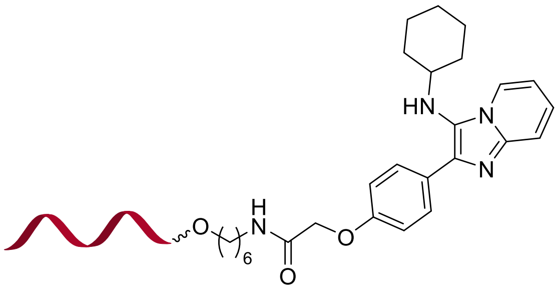

TC-10a

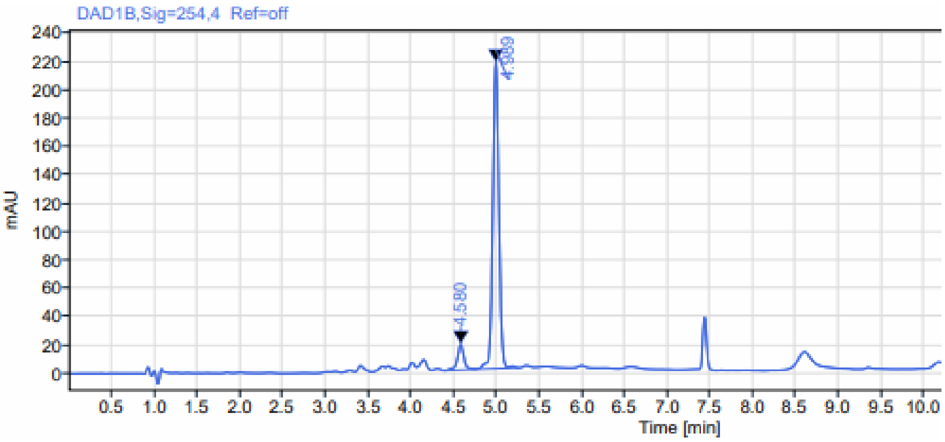

starting material

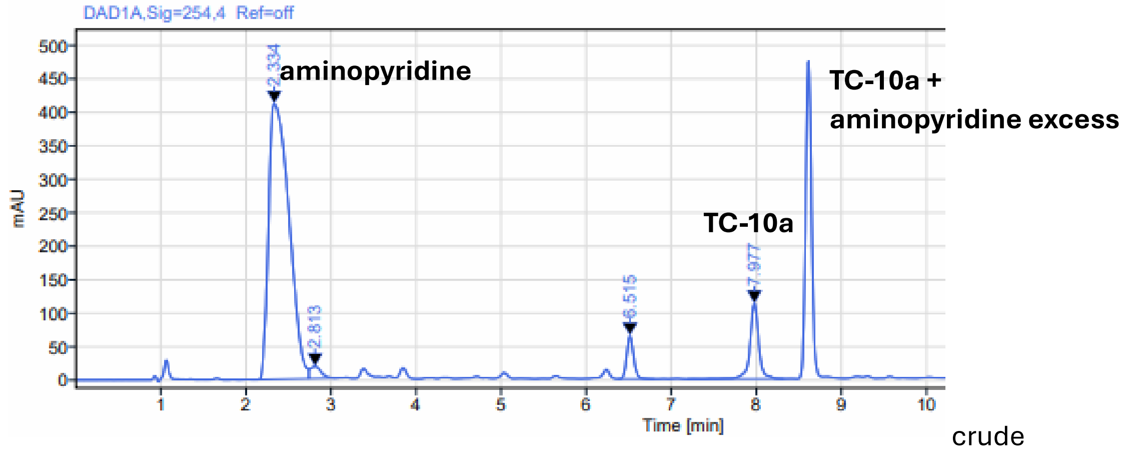

crude

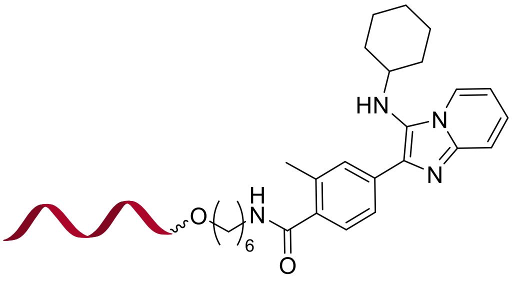

TC-10b

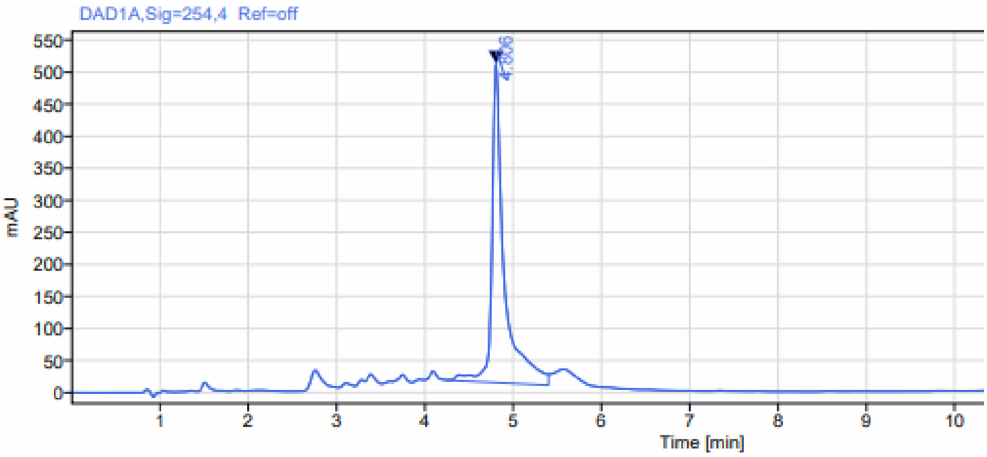

starting material

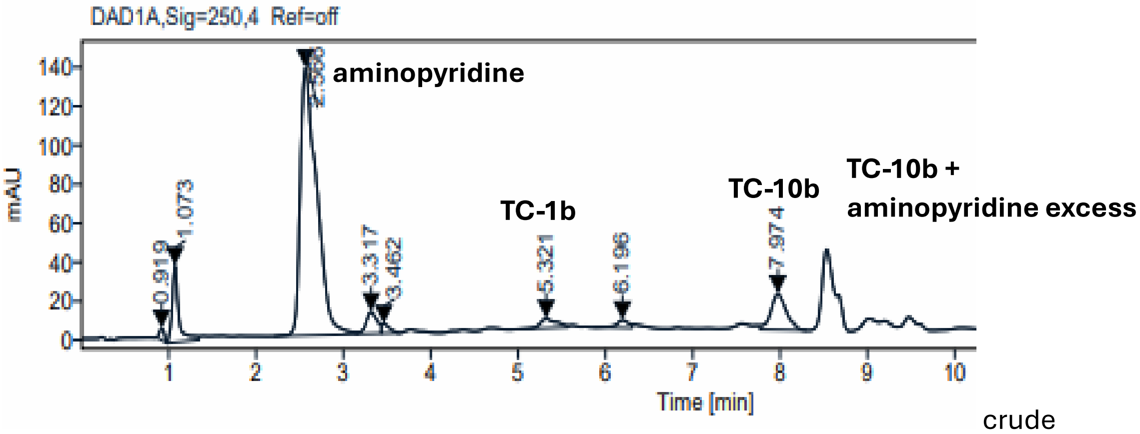

crude

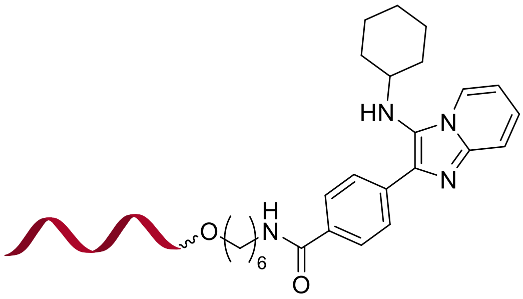

TC-10c

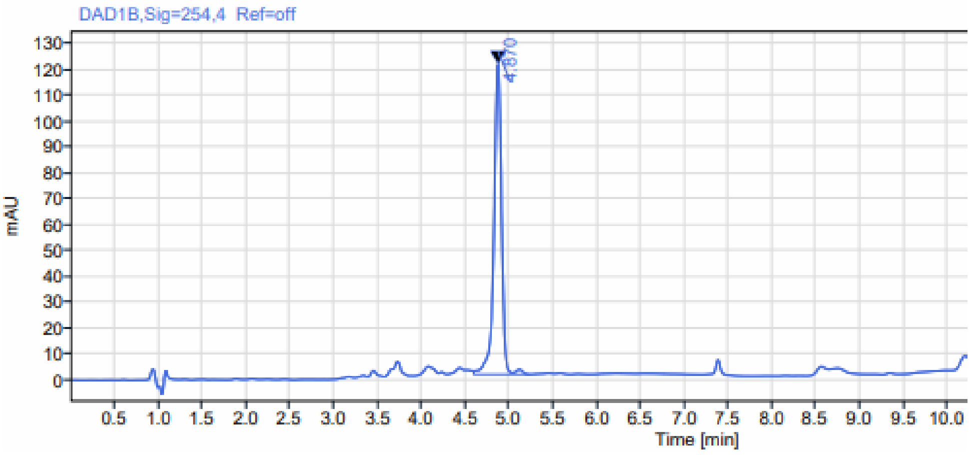

starting material

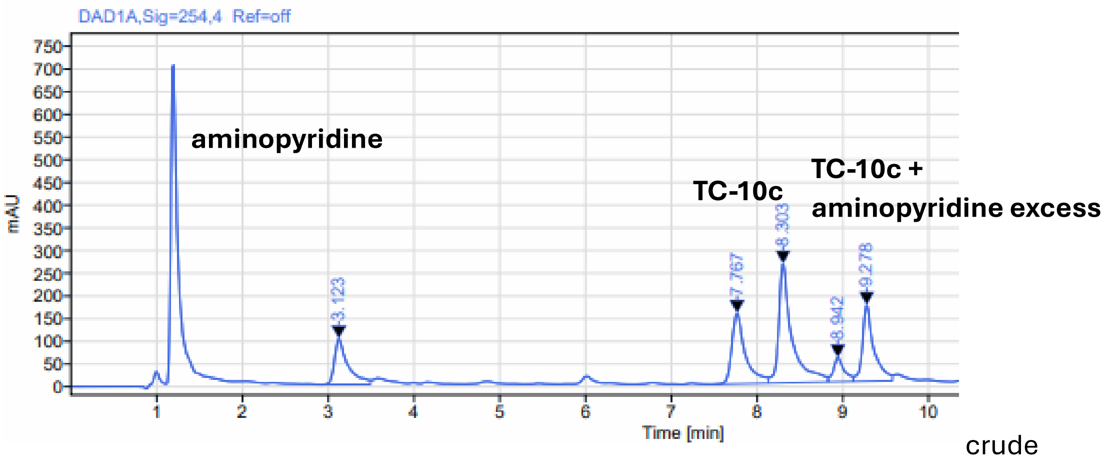

crude

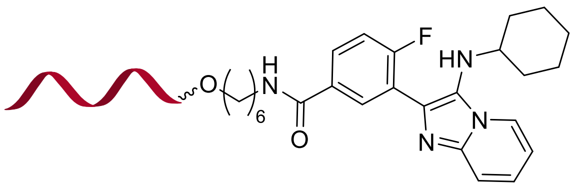

TC-10d

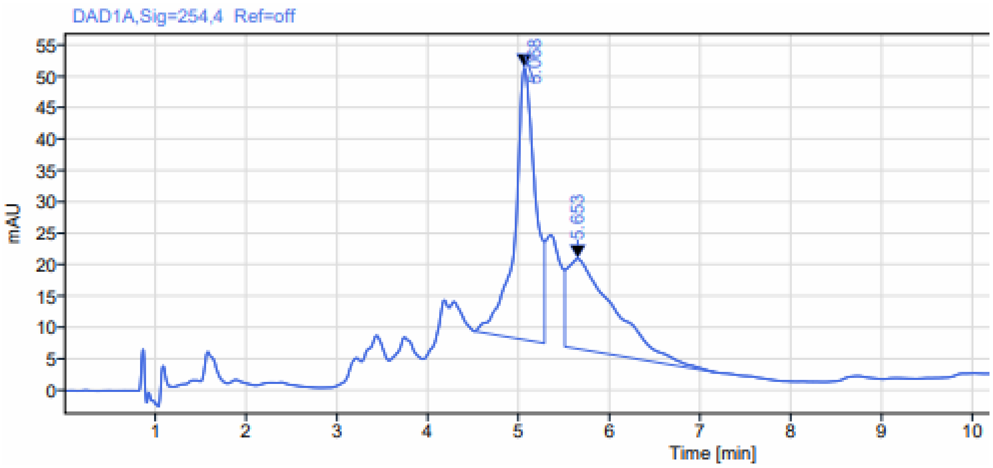

starting material

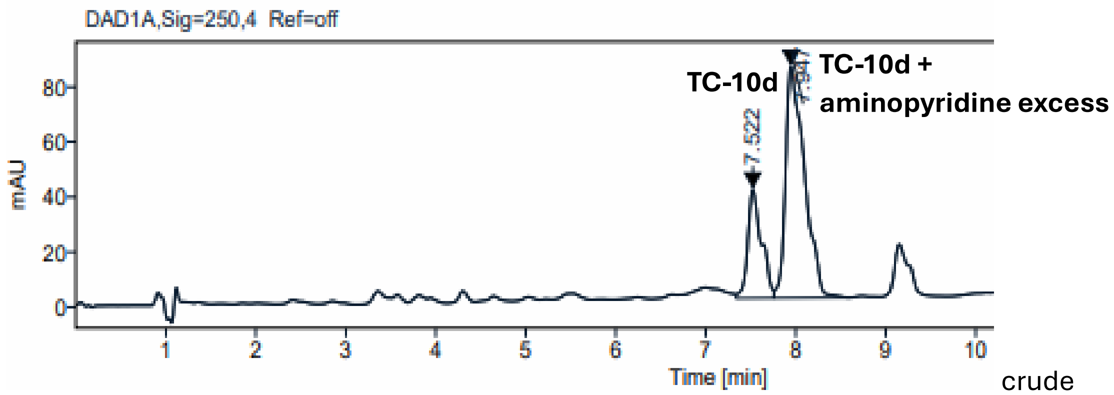

crude

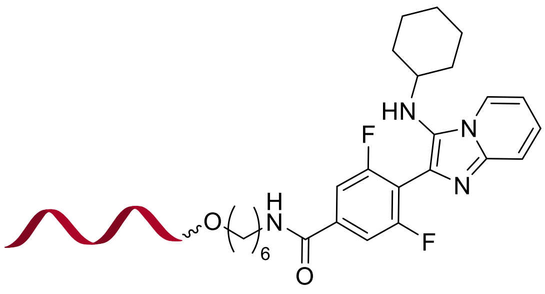

TC-10e

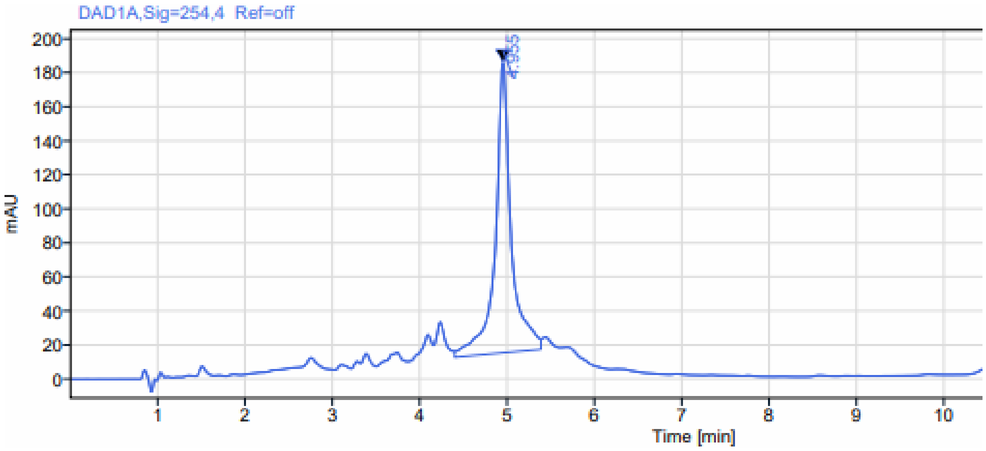

starting material

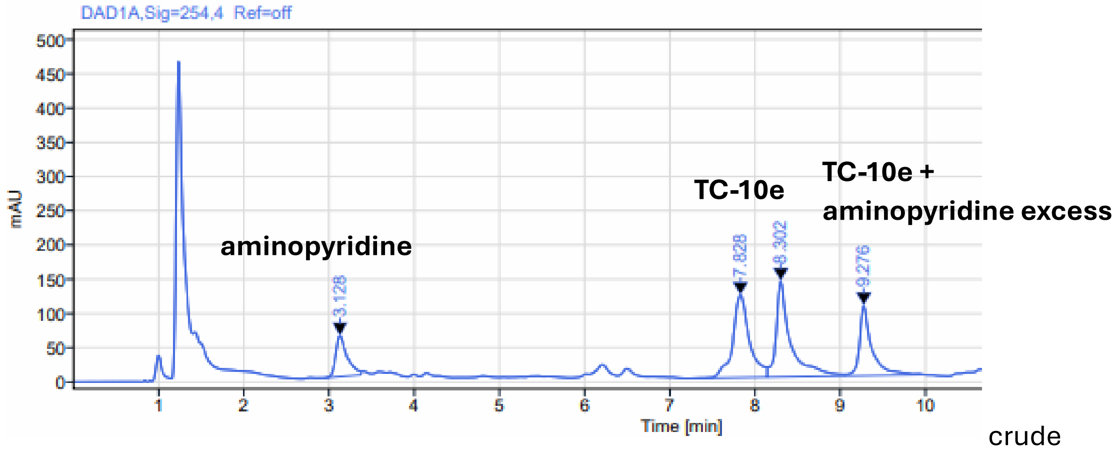

crude

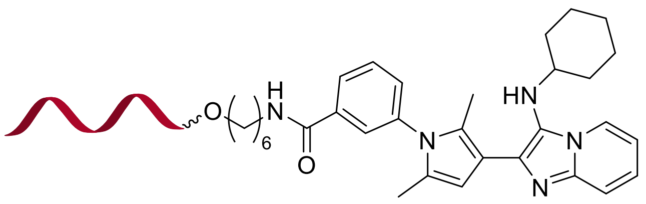

TC-10f

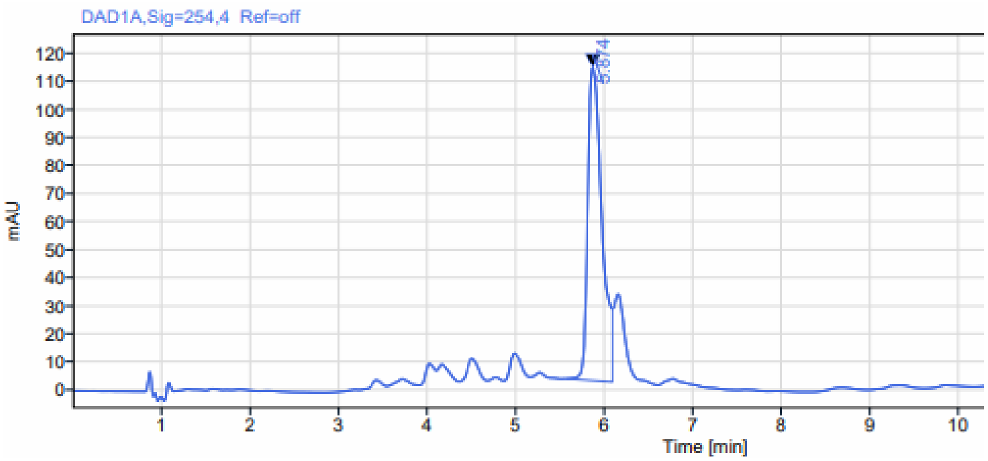

starting material

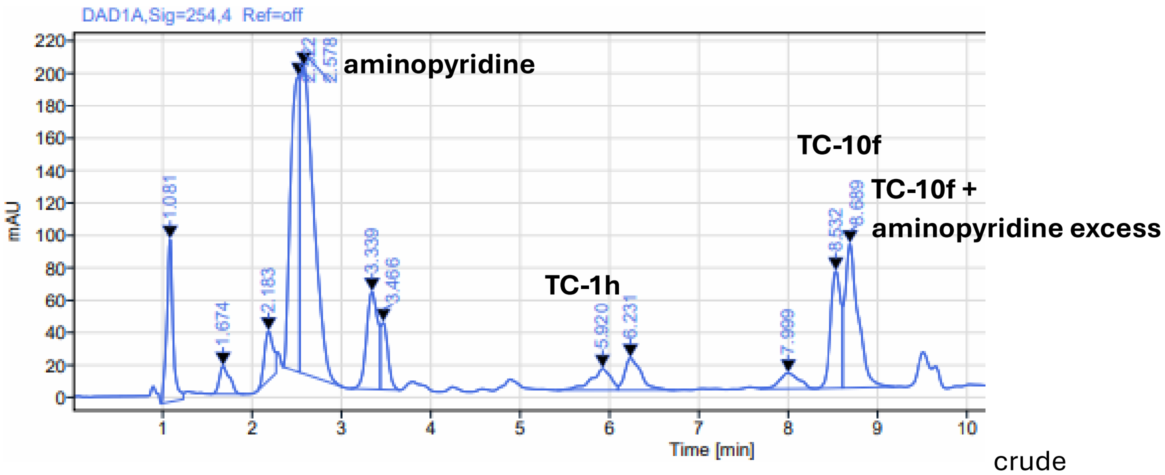

crude

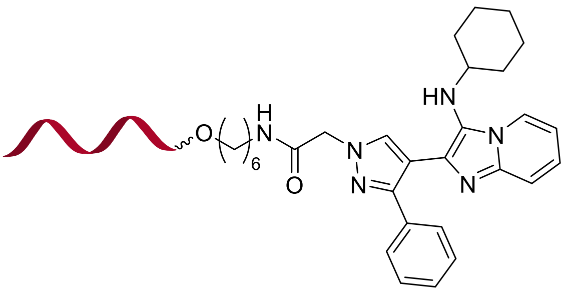

TC-10g

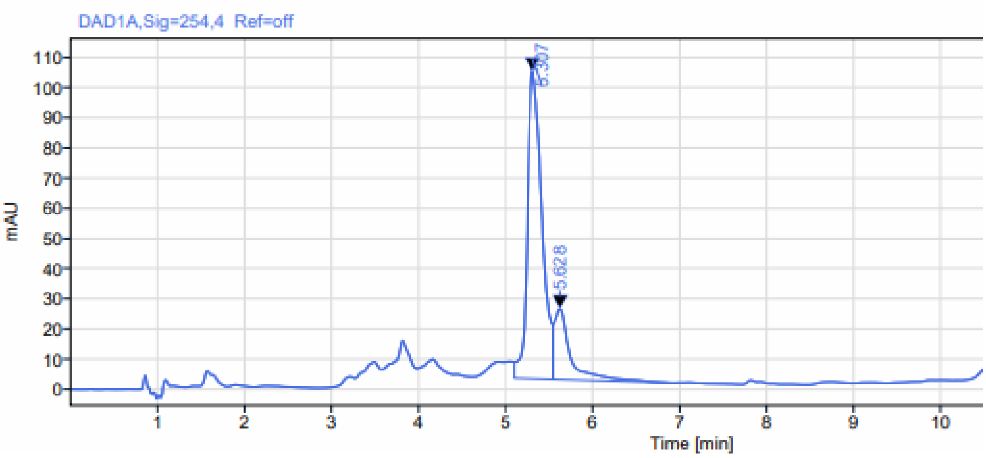

starting material

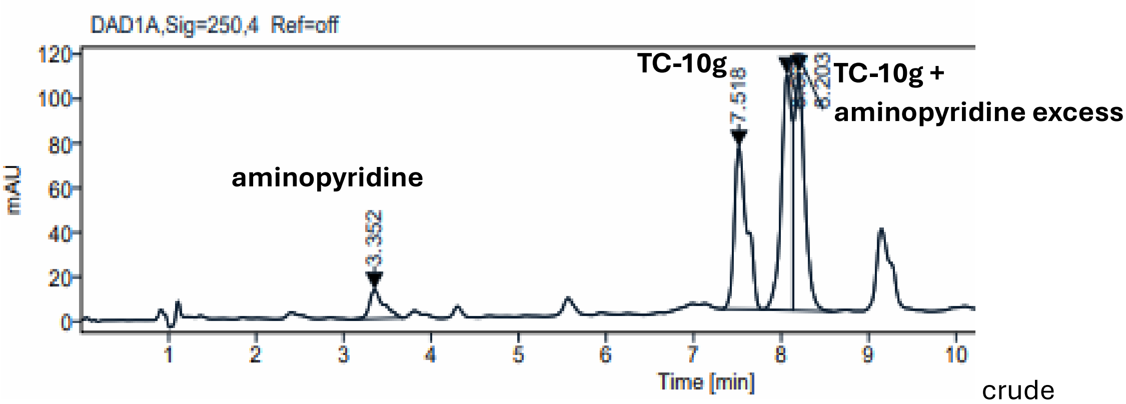

crude

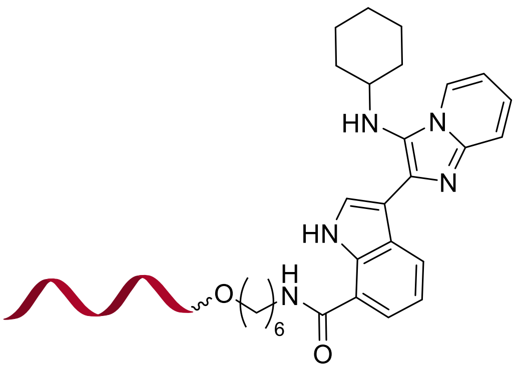

TC-10i

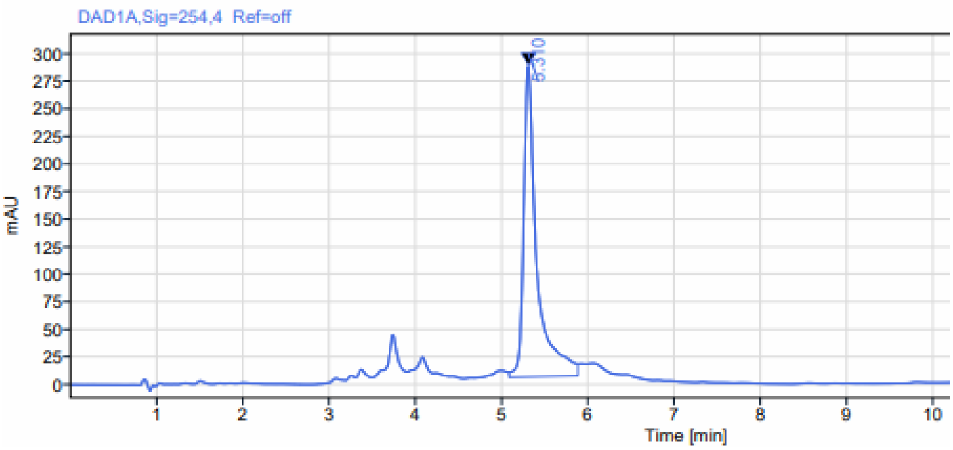

starting material

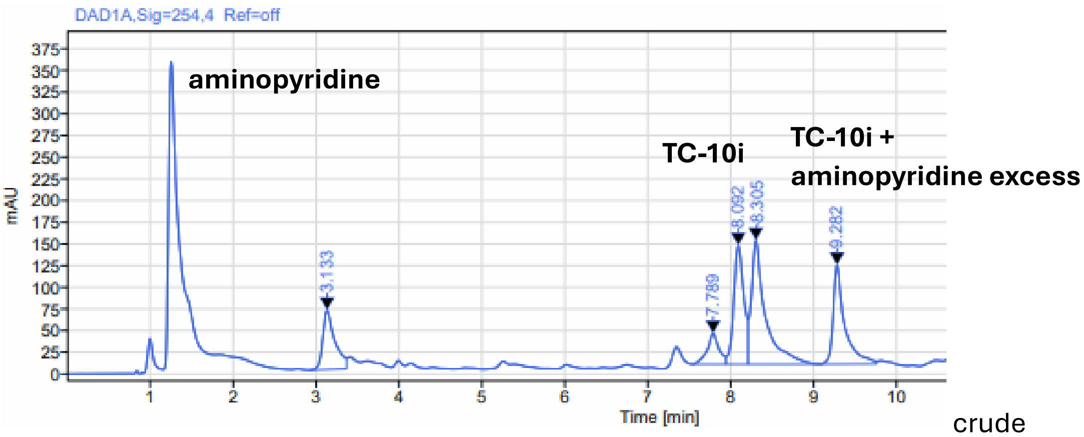

crude

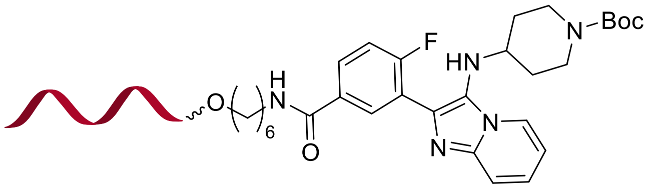

TC-10k

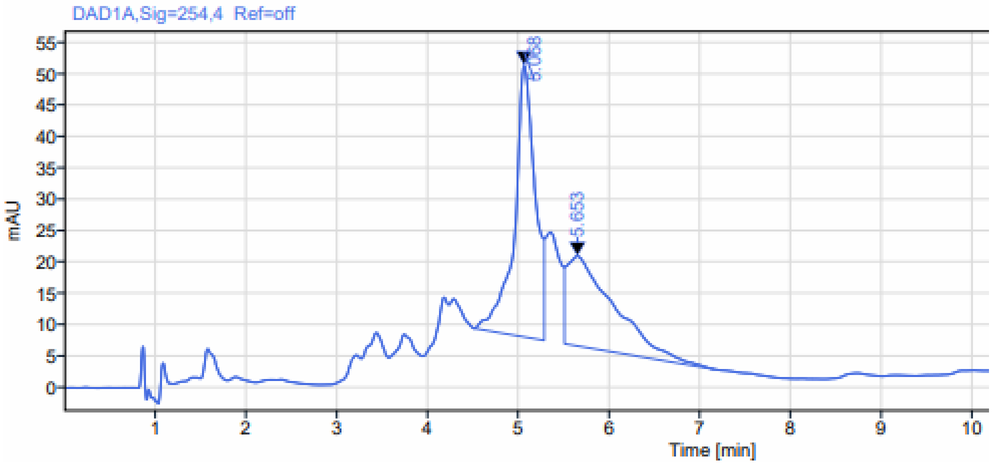

starting material

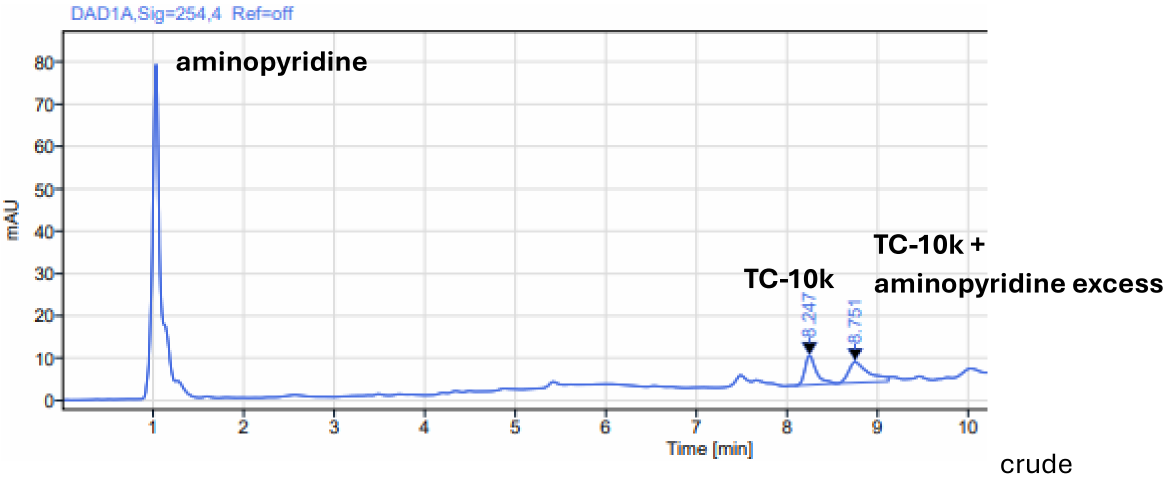

crude

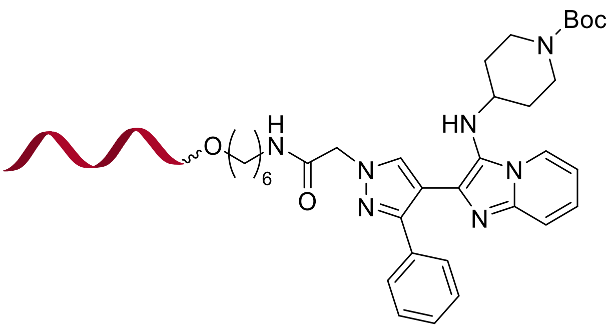

TC-10l

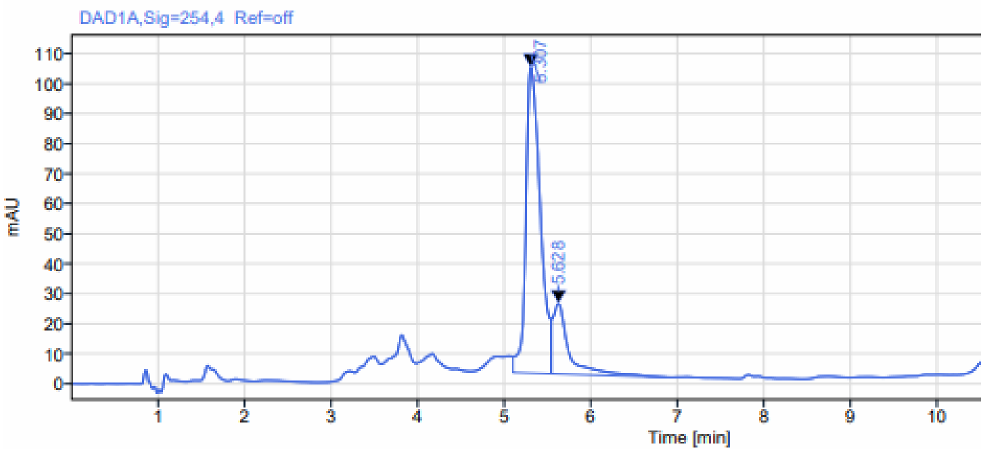

starting material

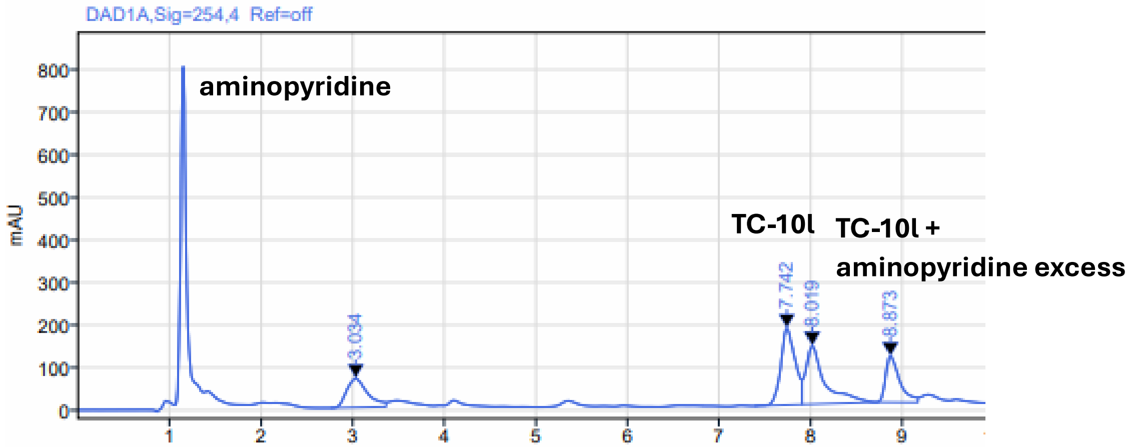

crude

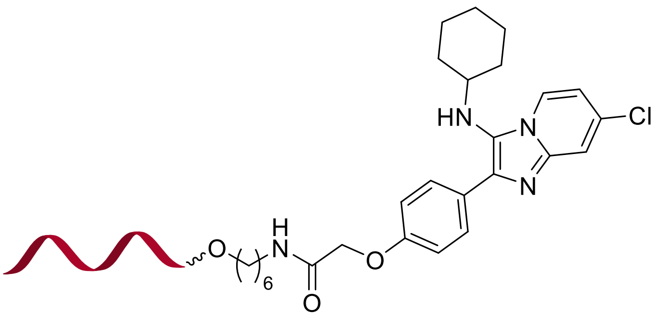

TC-10m

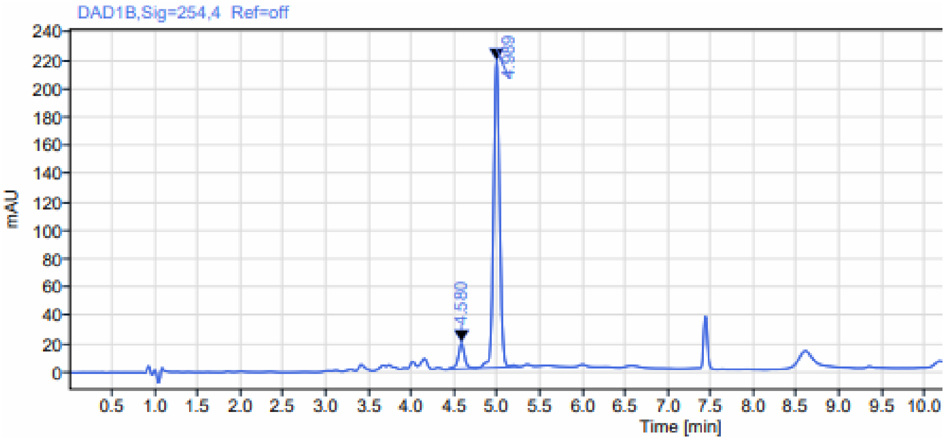

starting material

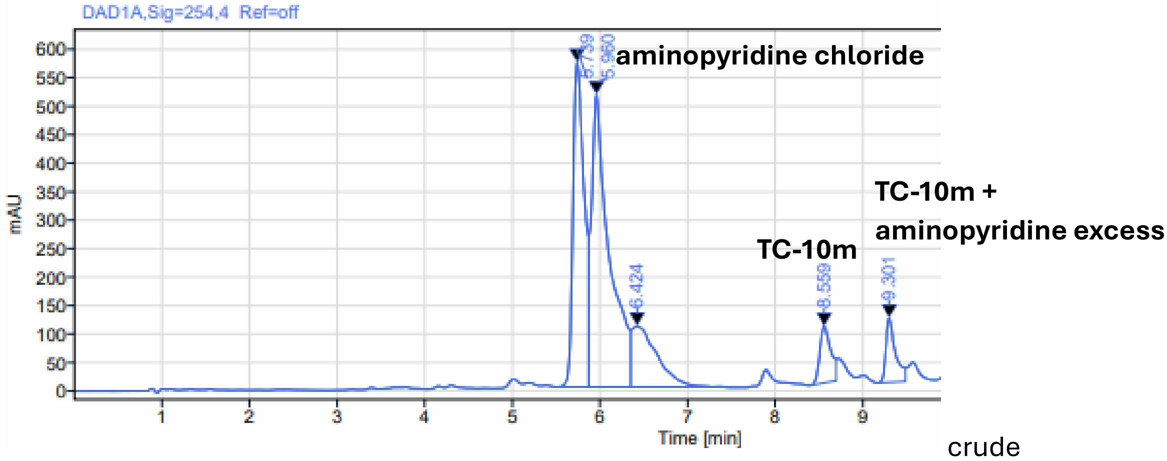

crude

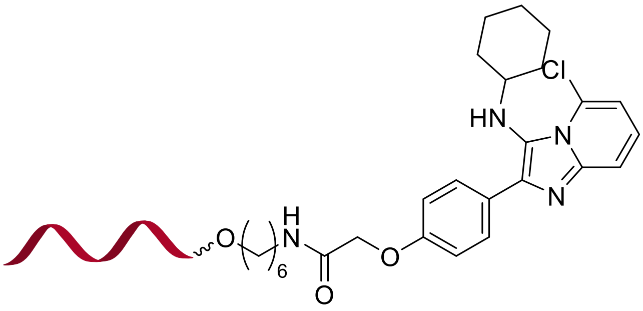

TC-10n

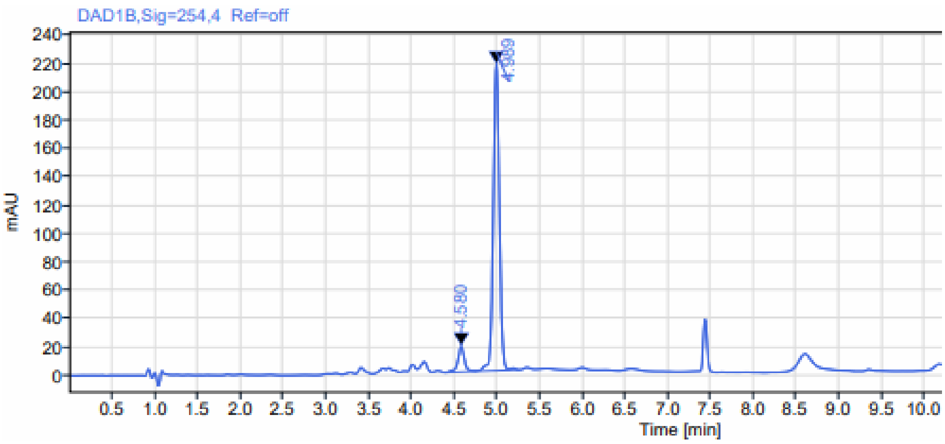

starting material

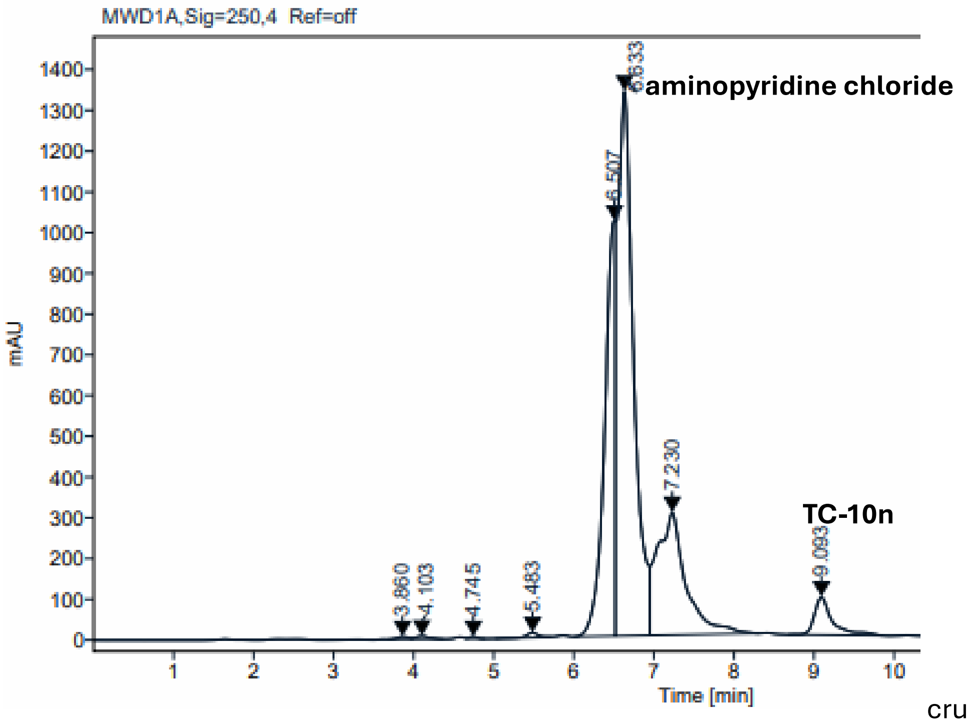

crude

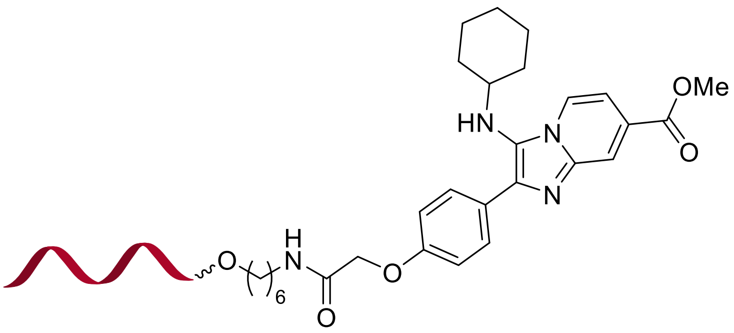

TC-10o

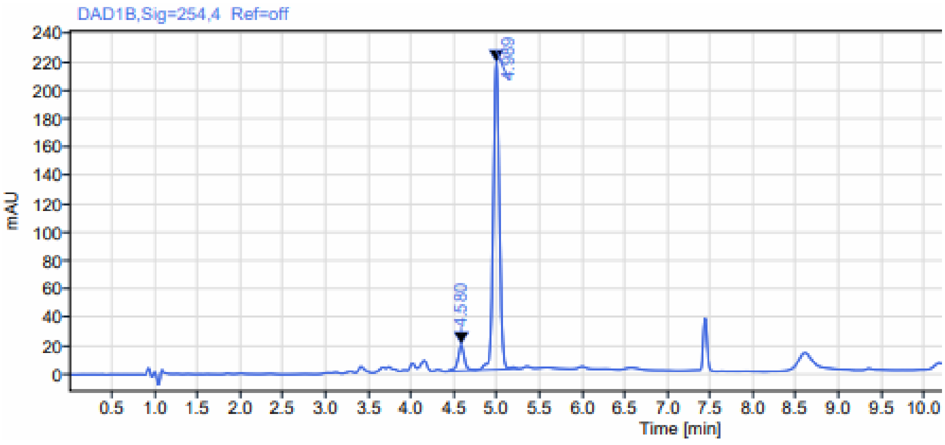

starting material

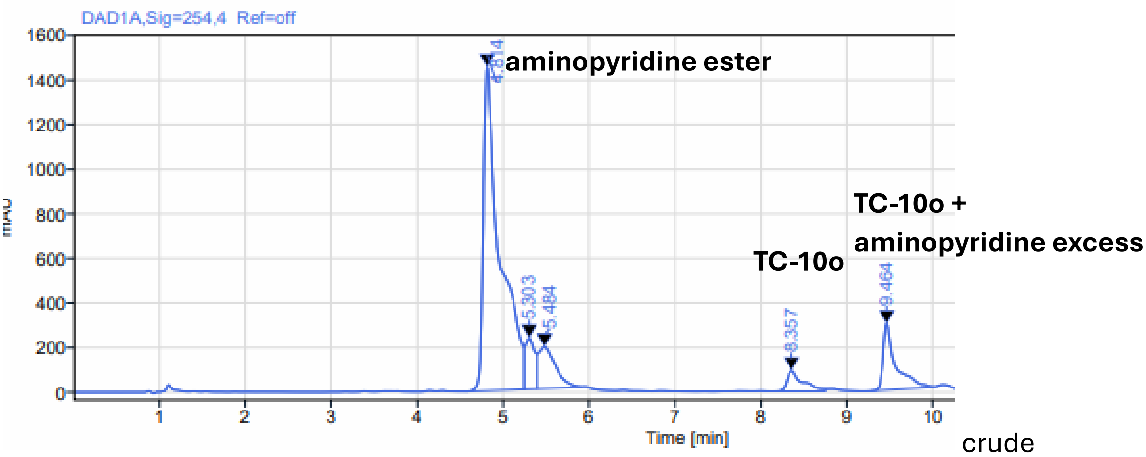

crude

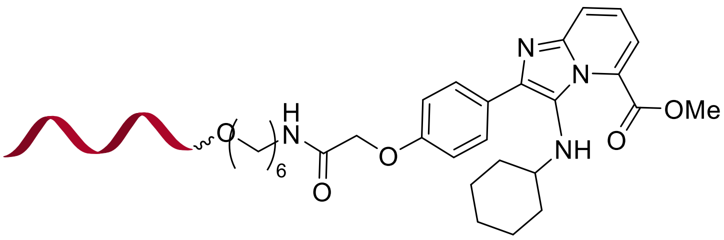

TC-10p

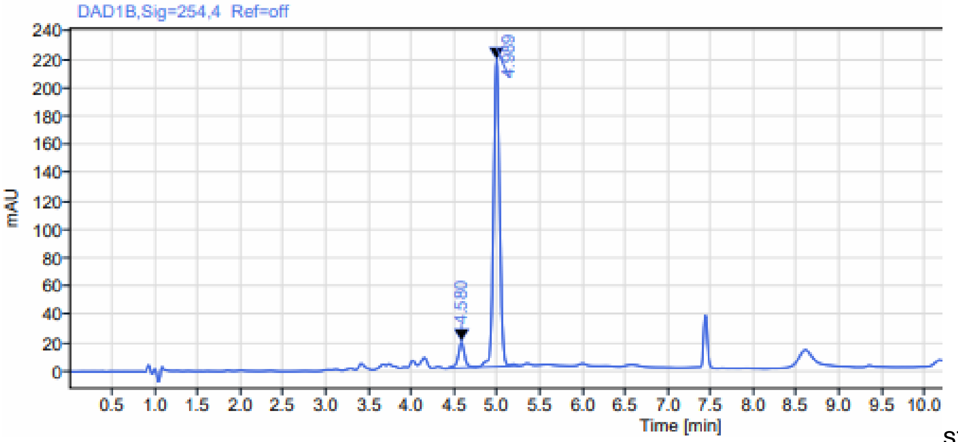

starting material

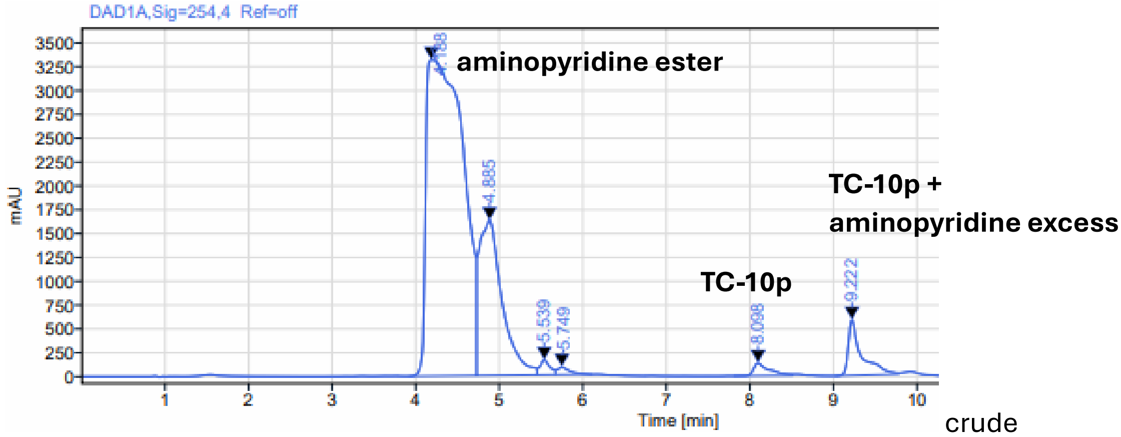

crude

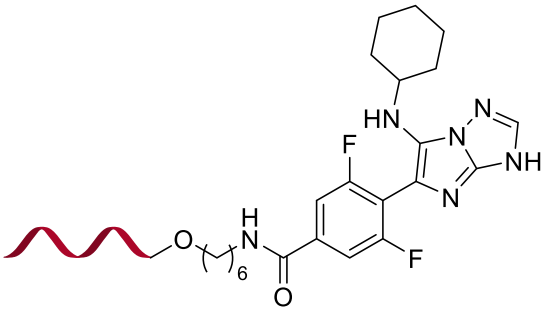

TC-10t

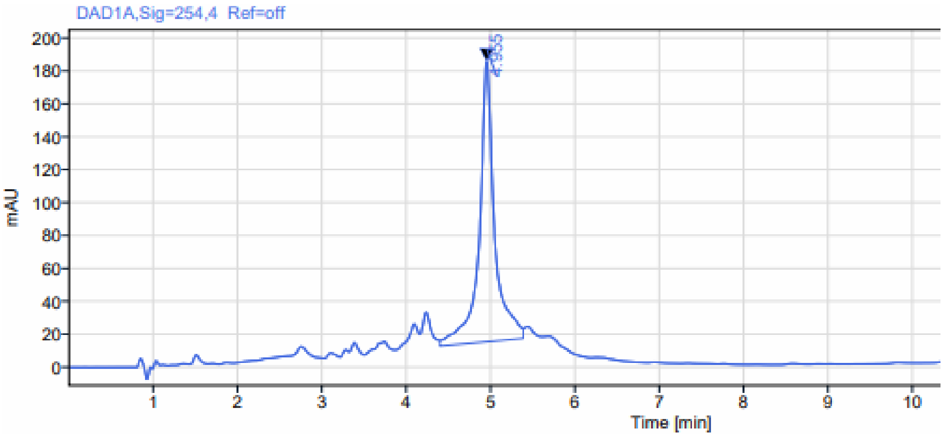

starting material

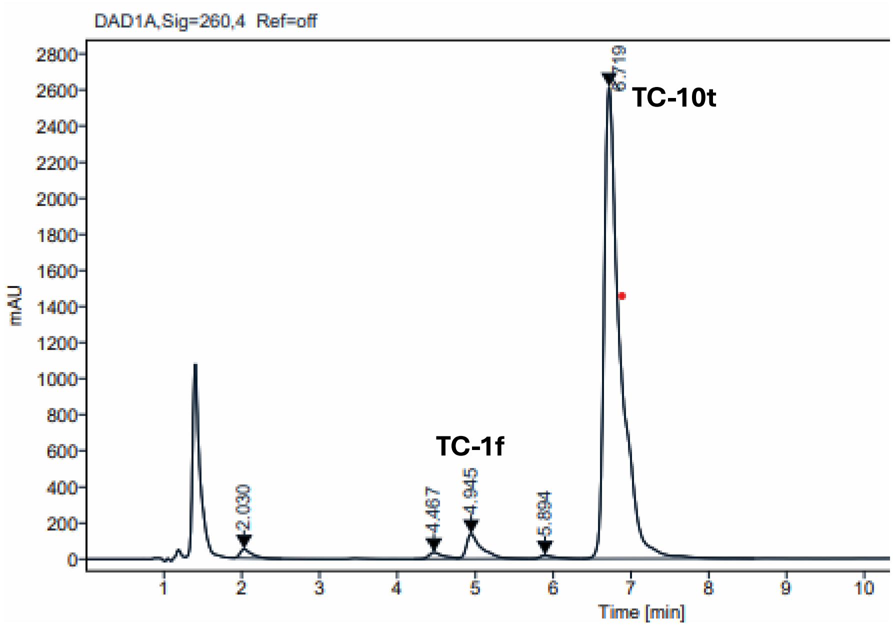

crude

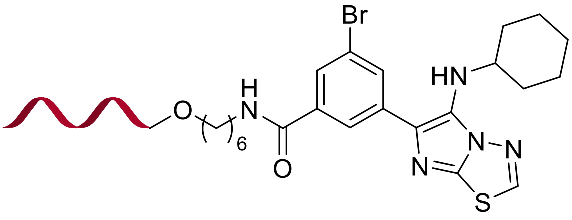

TC-10u

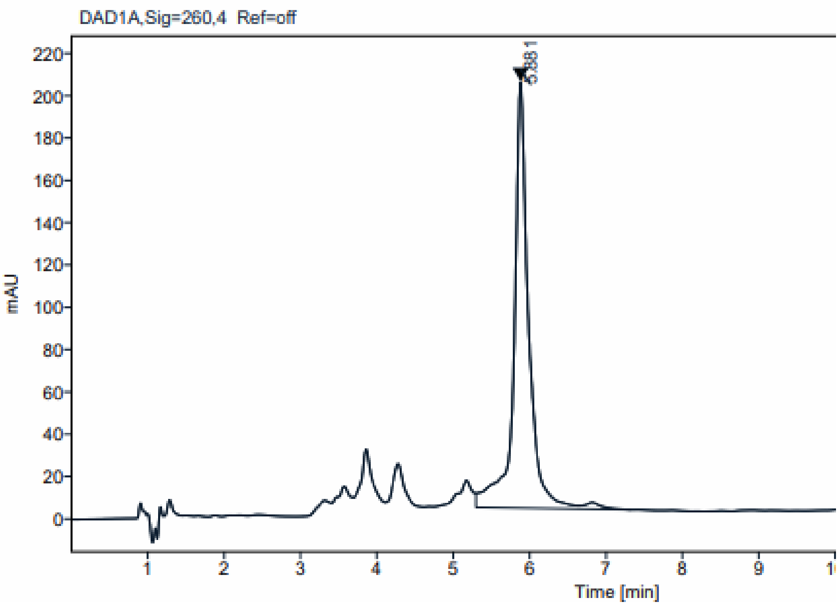

starting material

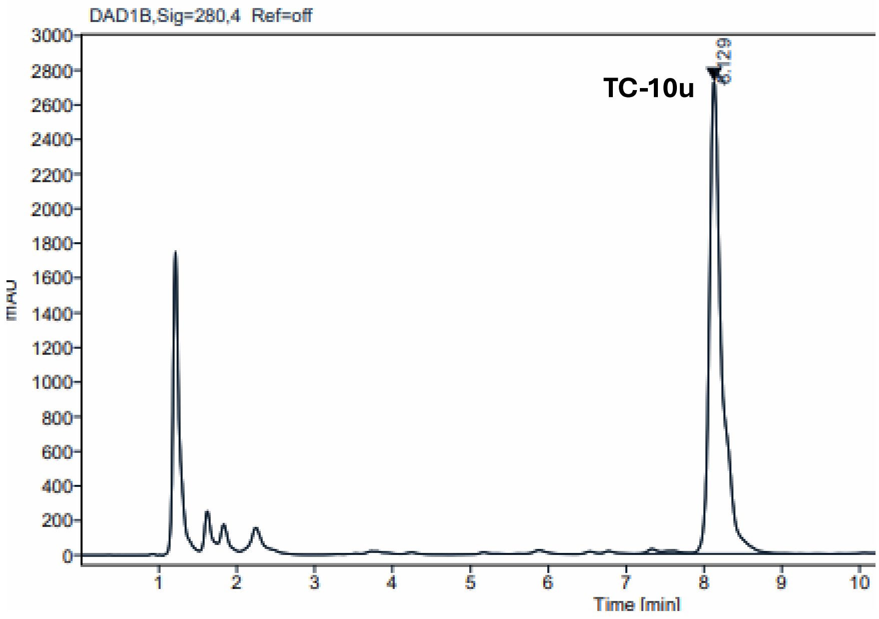

crude

IMCRs on different DNA sequences

Amide Coupling of Carboxylic Acid to CPG-Bound Oligonucleotide (14mer 7dATC)

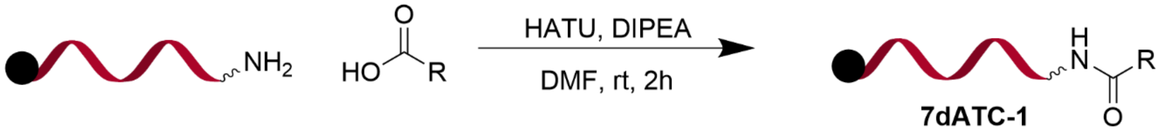

	Aldehyde
**7dATC-1a**	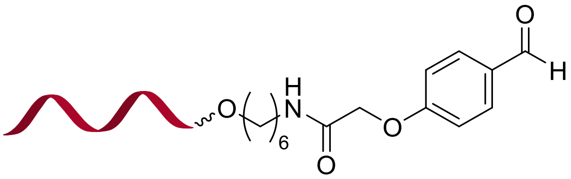
**7dATC-1b**	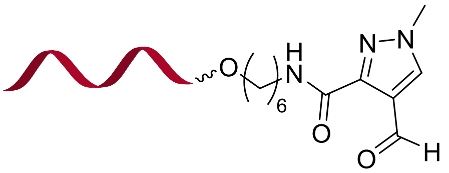
**7dATC-1c**	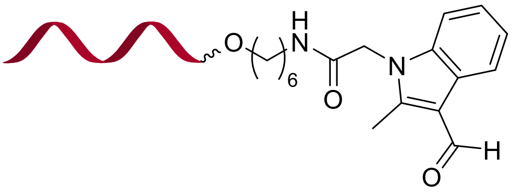
**7dATC-1d**	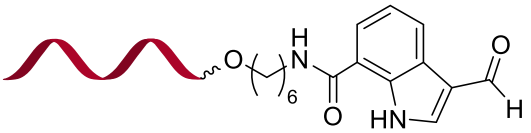
**7dATC-1e**	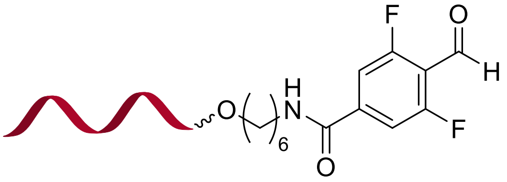
**7dATC-1f**	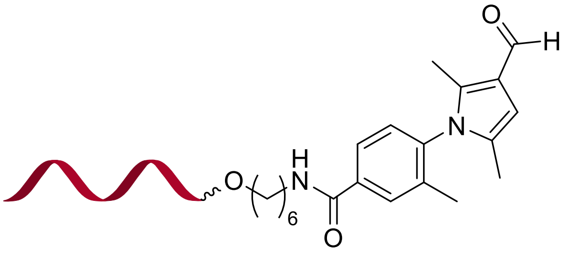

Amide Coupling of Carboxylic Acid to CPG-Bound Oligonucleotide (14mer ATGC)

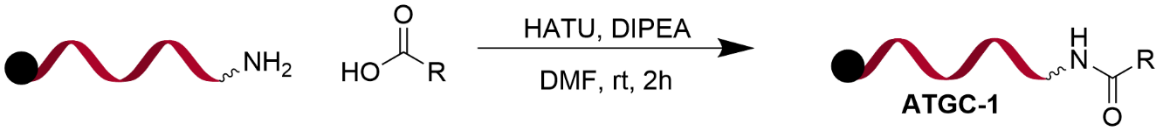

	Aldehyde
**ATGC-1a**	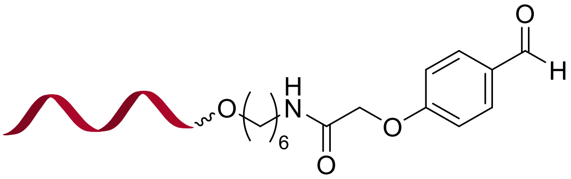
**ATGC-1b**	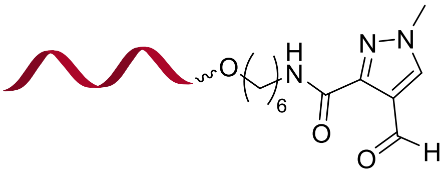
**ATGC-1c**	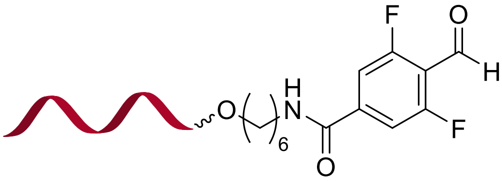
**ATGC-1d**	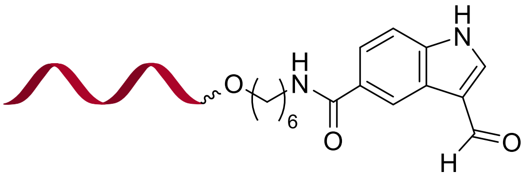

Ugi four-component reaction with 14mer 7dATC-coupled aldehydes

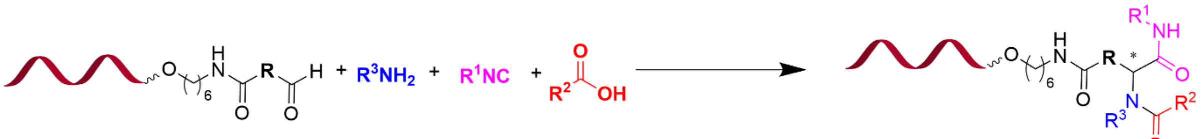

7dATC-1 2 3 4 7dATC-5

Table S10. MALDI-MS data of products of the Ugi four-component reaction with different aldehydes coupled to 14mer 7dATC.

					MALDI-MS m/z	
No.	R	R^1	R^2	R^3	calculated	found
7dATC-5a	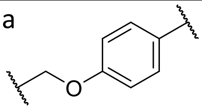	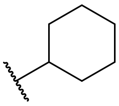	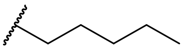	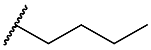	4868.0	4870.9
7dATC-5b	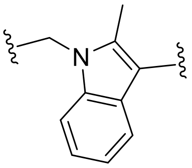	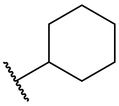	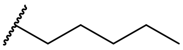	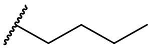	4905.0	4907.9
7dATC-5c	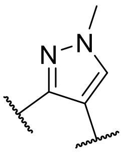	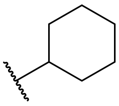	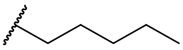	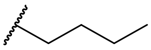	4842.0	4845.1
7dATC-5d	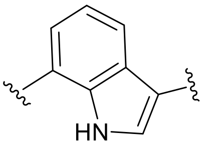	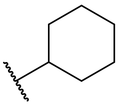	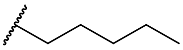	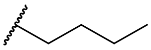	4877.0	4880.0

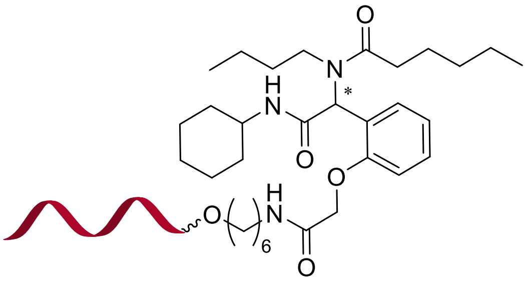

7dATC-5a

MS calc. 4686.0; found: 4870.9

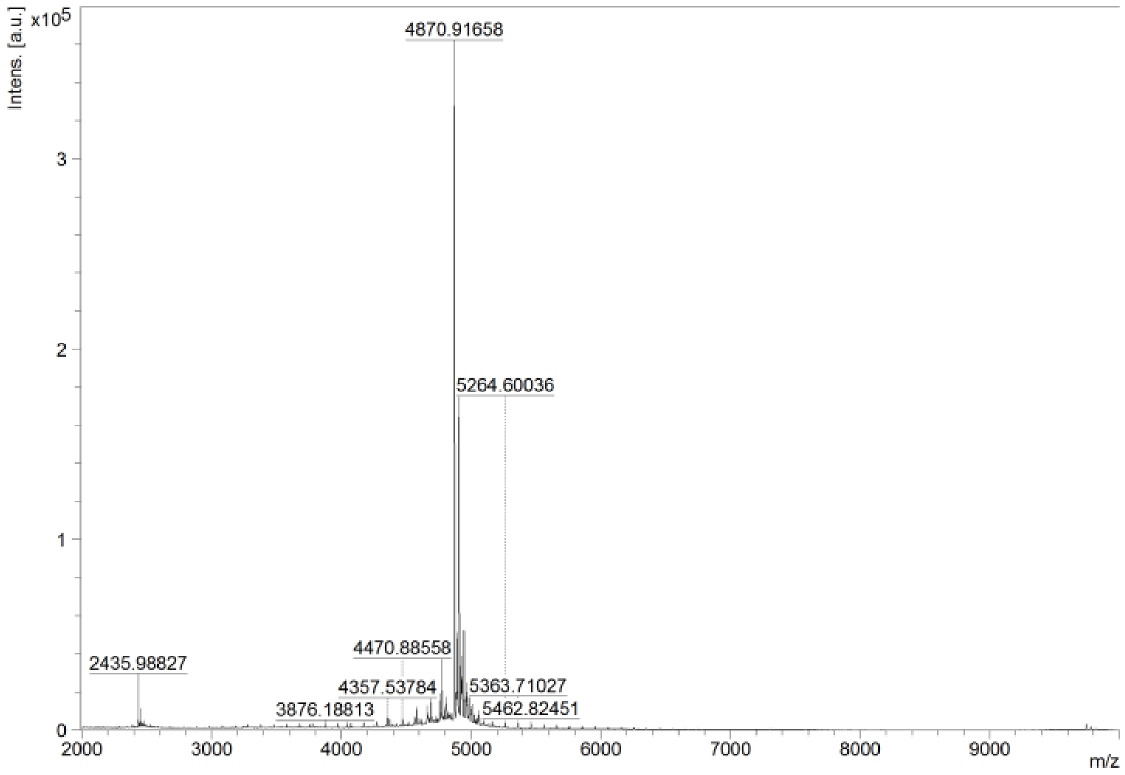

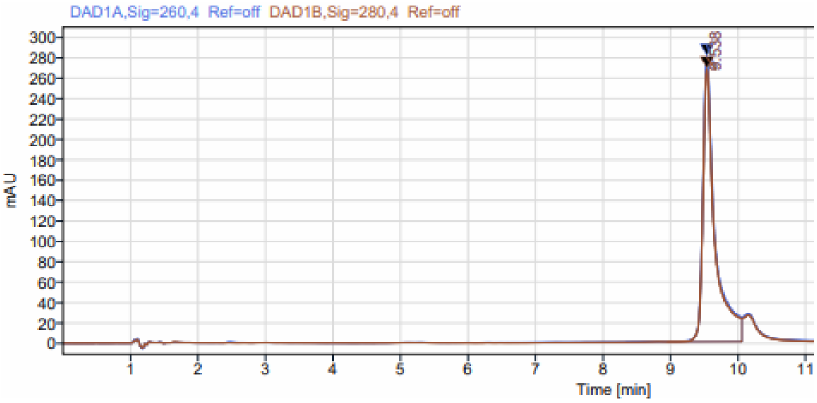

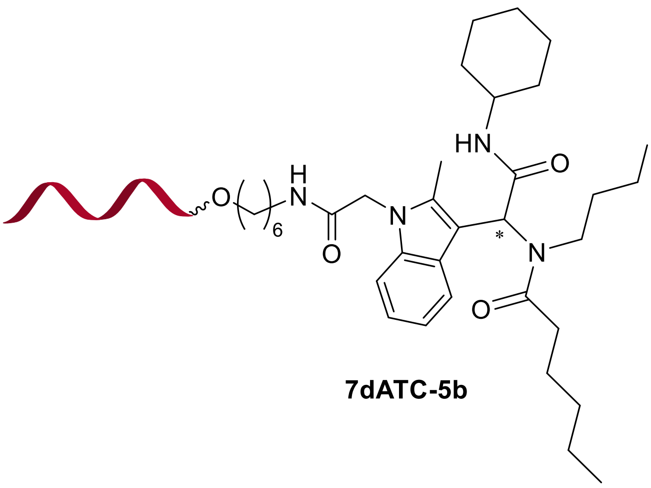

7dATC-5b

MS calc. 4905.0; found: 4907.9

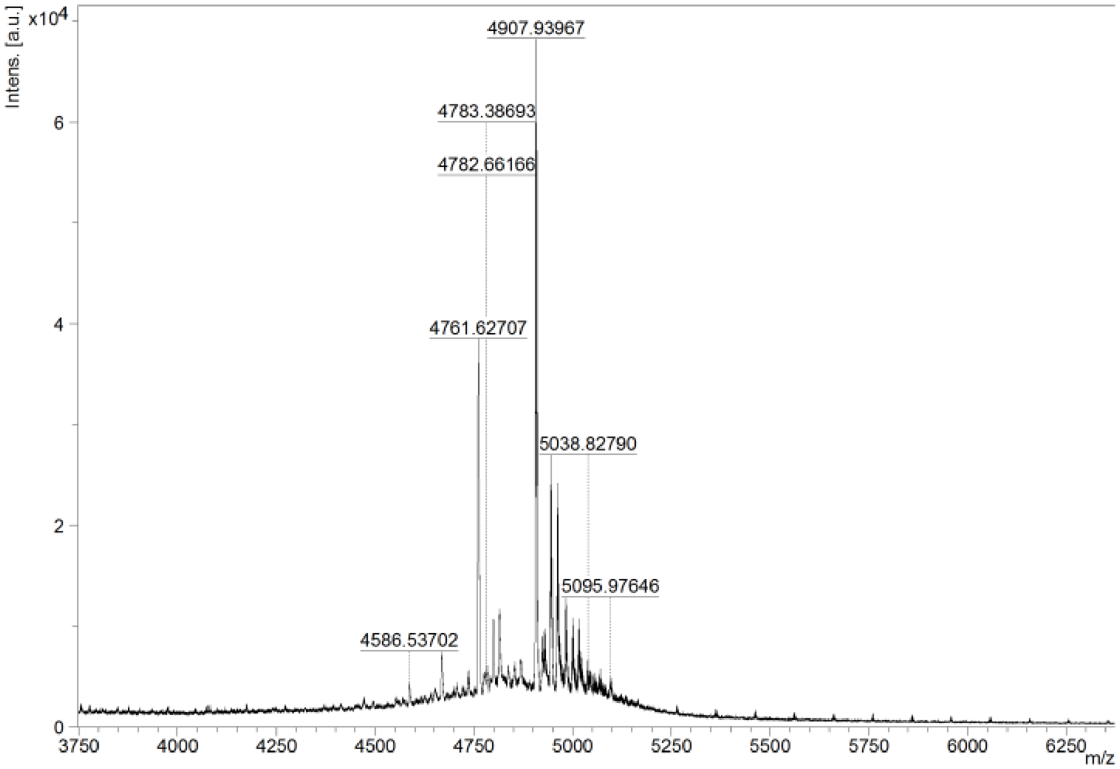

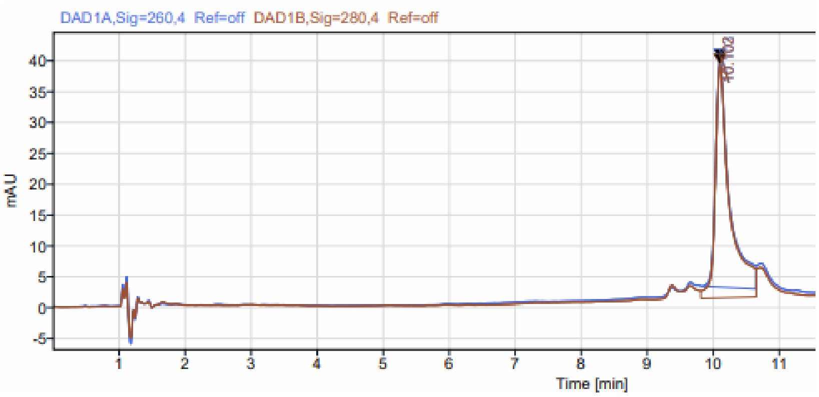

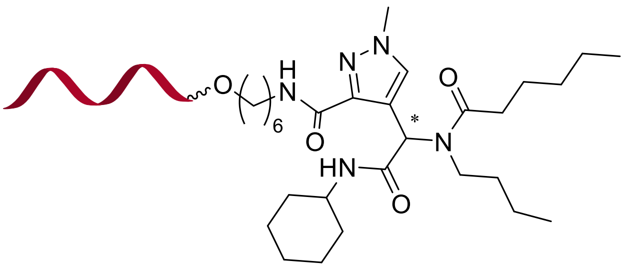

7dATC-5c

MS calc. 4842.0; found: 4845.1

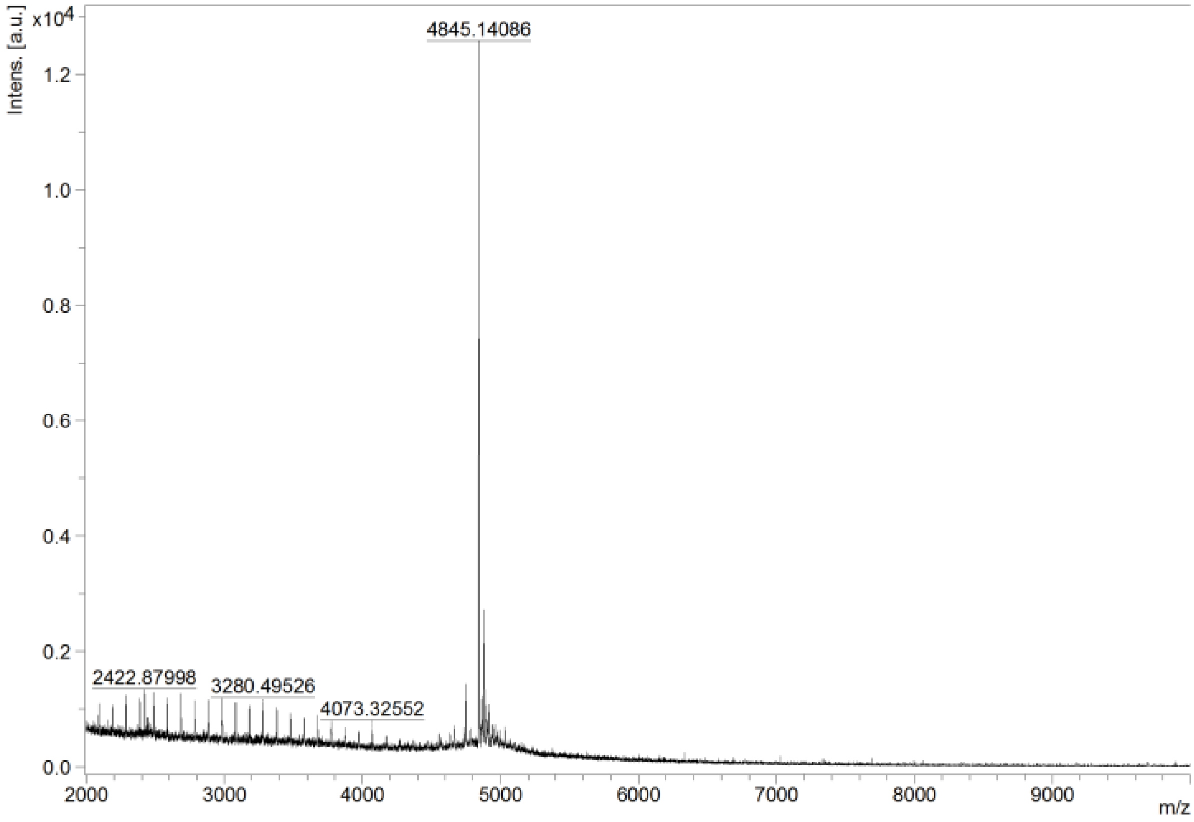

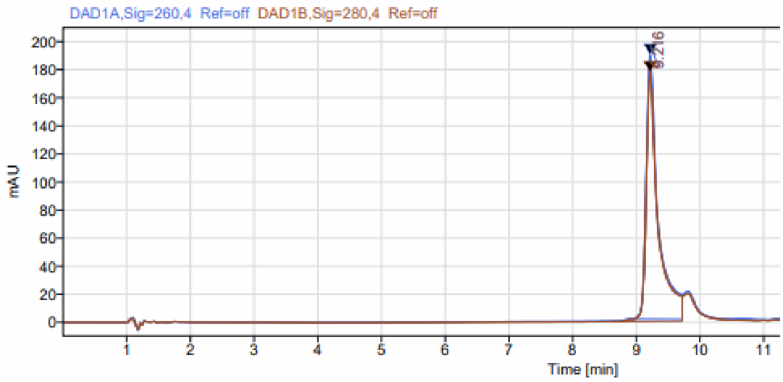

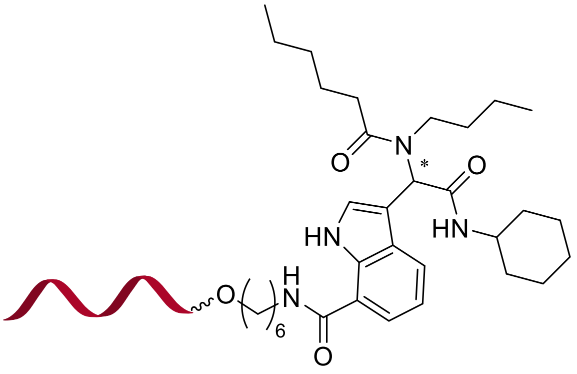

7dATC-5d

MS calc. 4877.0; found: 4880.0

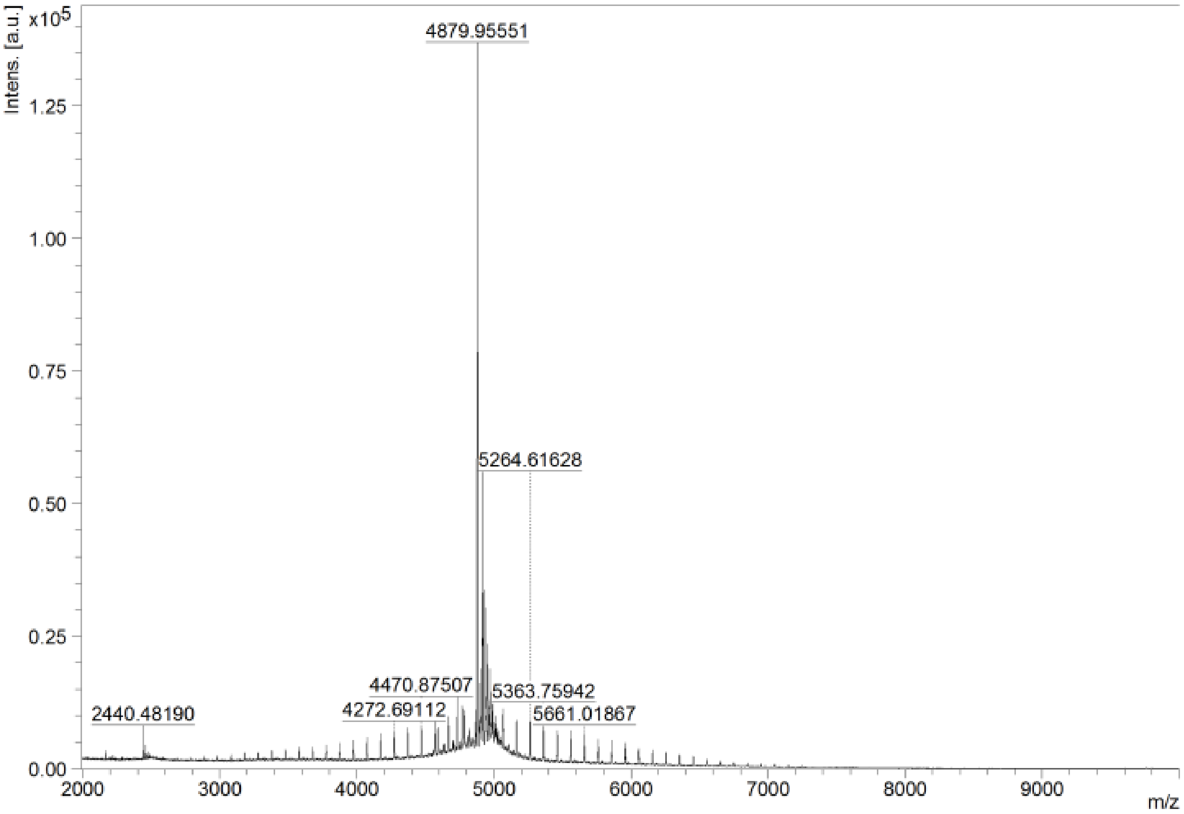

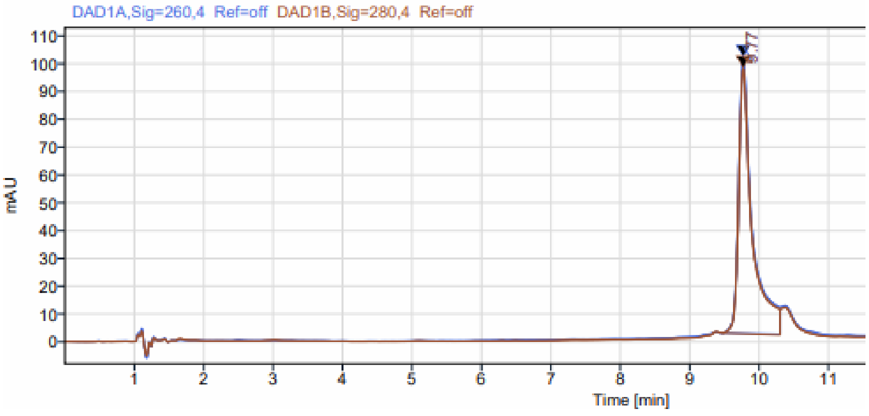

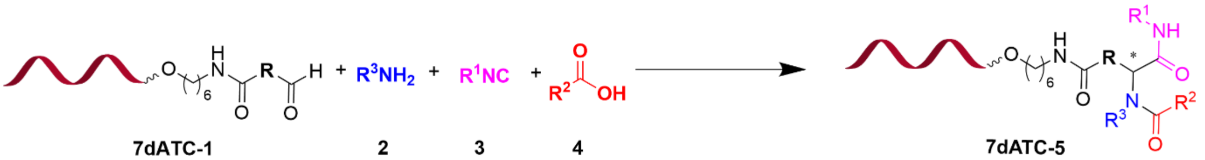

Table S11. Product conversions of products of the Ugi four-component reaction with different aldehydes coupled to 14mer 7dATC.

**No.**	**R**	**R¹**	**R²**	**R³**	**Product conversion**
7dATC-5a	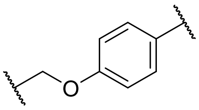	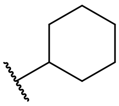	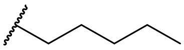	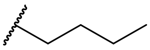	57%
7dATC-5b	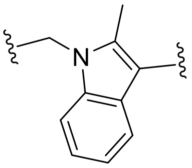	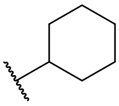	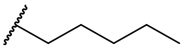	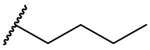	91%
7dATC-5c	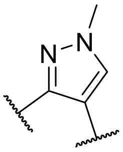	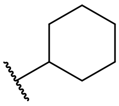	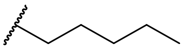	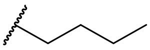	61%
7dATC-5d	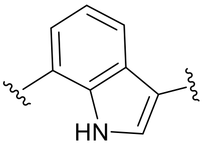	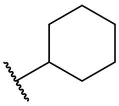	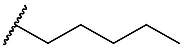	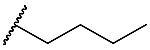	79%

Ugi four-component reaction with 14mer ATGC-coupled aldehydes

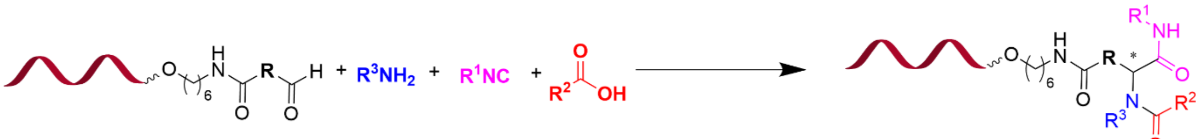

ATGC-1 2 3 4 ATGC-5

Table S12. MALDI-MS data of products of the Ugi four-component reaction with different aldehydes coupled to 14mer ATGC.

			**MALDI-MS m/z**			
No.	R	R^1	R^2	R^3	calculated	found
ATGC-5a	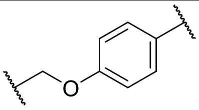	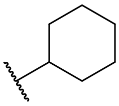	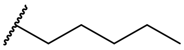	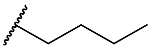	4861.0	4862.9
ATGC-5b	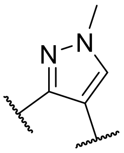	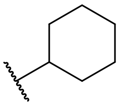	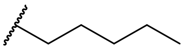	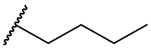	4826.0	4827.6
ATGC-5c	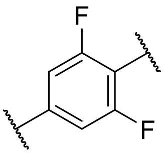	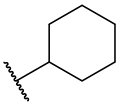	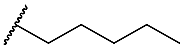	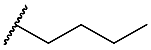	4867.0	4868.8
ATGC-5d	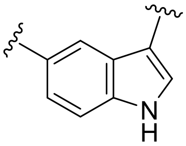	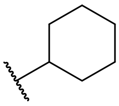	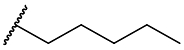	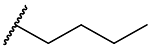	4861.0	4862.7

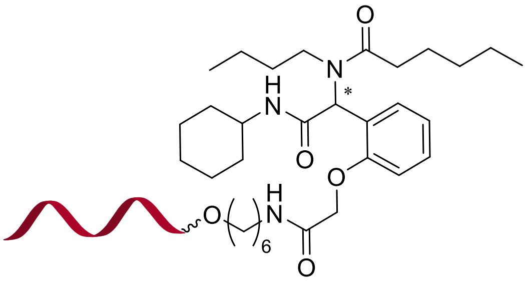

ATGC-5a

MS calc. 4861.0; found: 4862.8

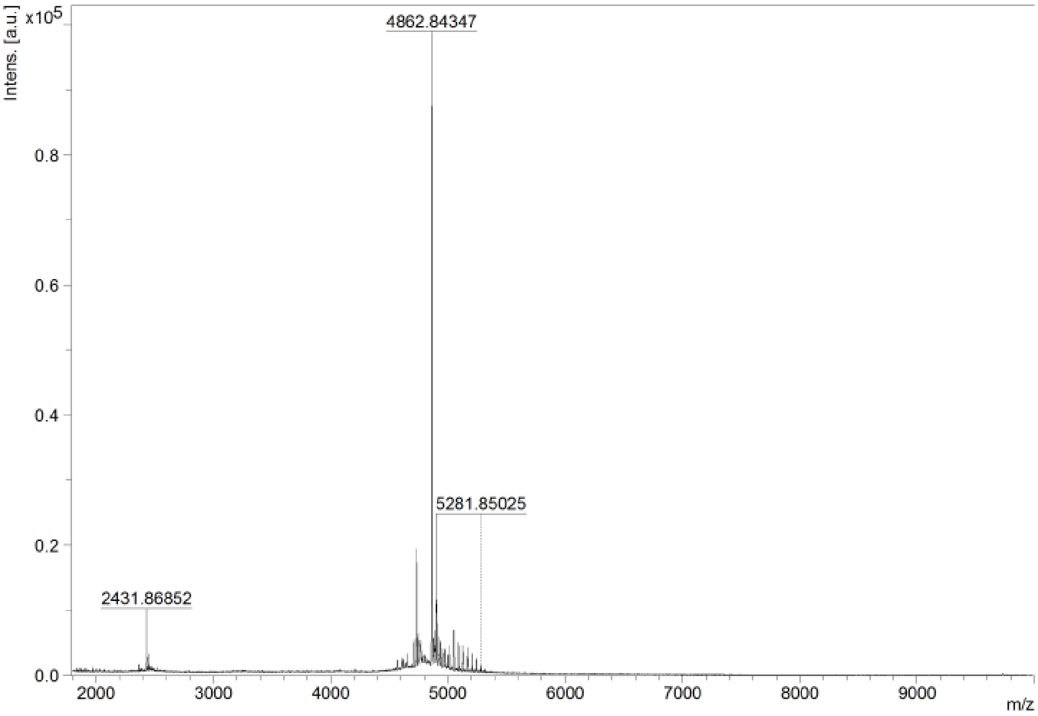

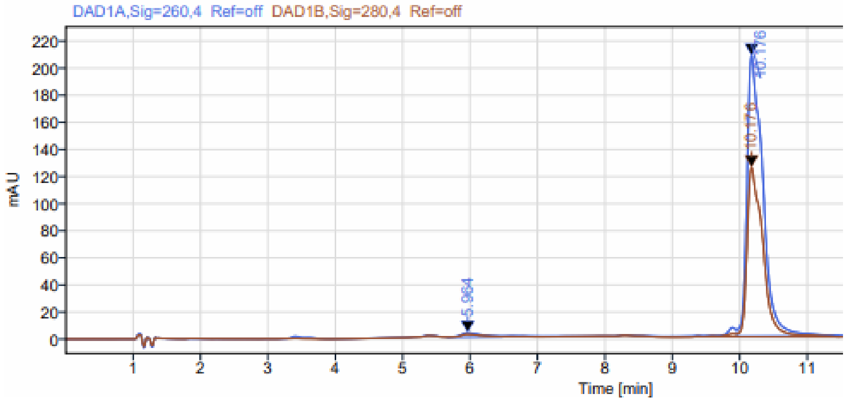

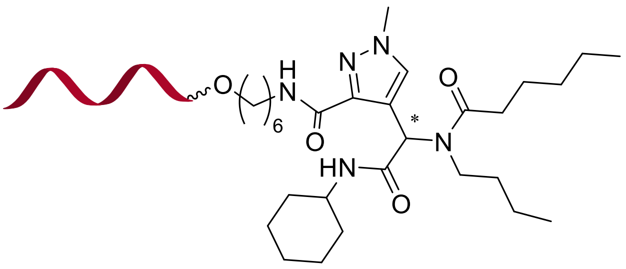

ATGC-5b

MS calc. 4826.0; found: 4827.6

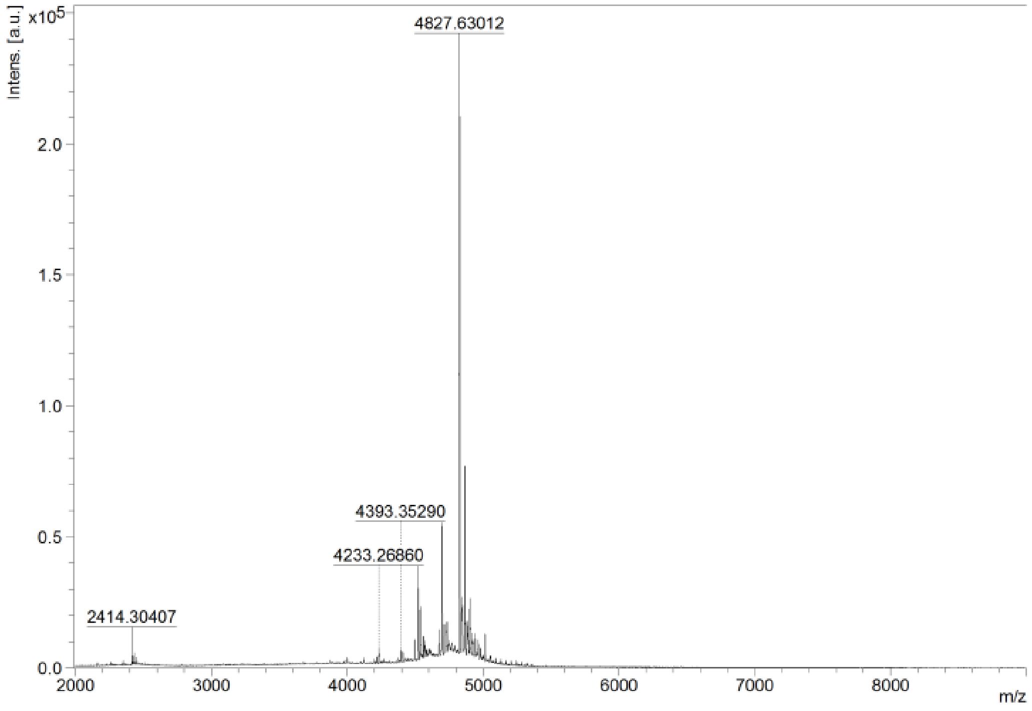

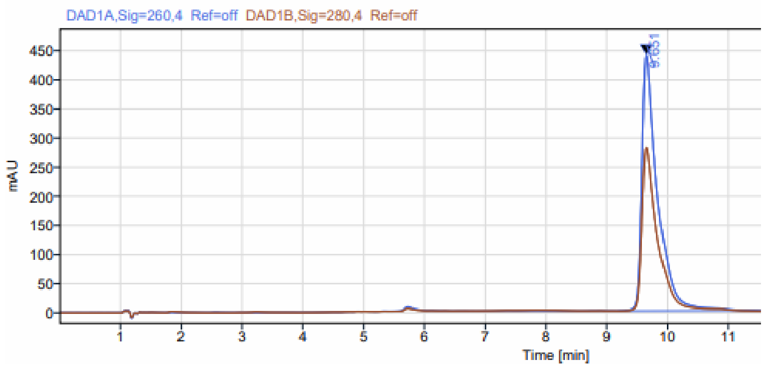

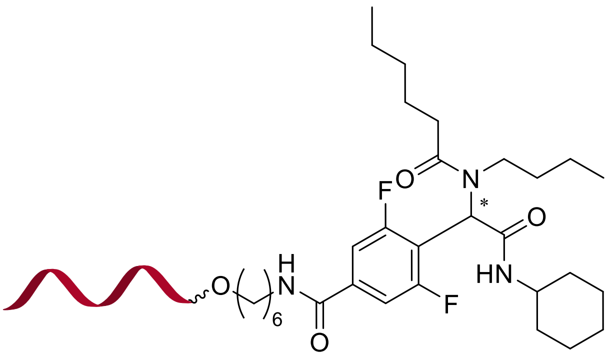

ATGC-5c

MS calc. 4867.0; found: 4868.8

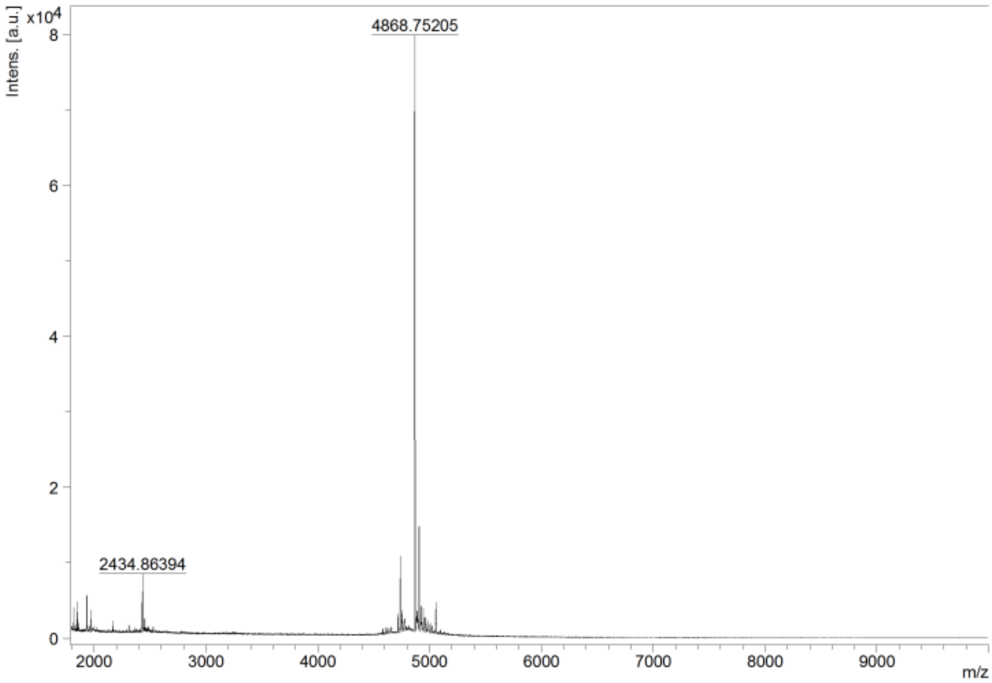

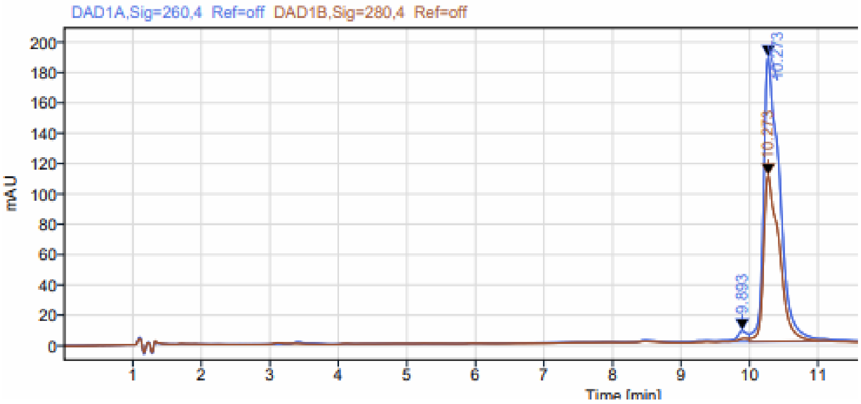

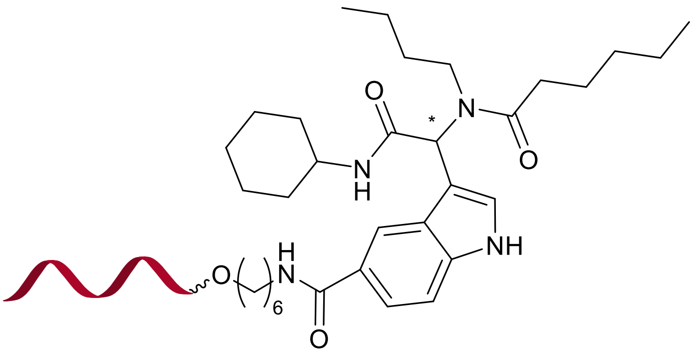

ATGC-5d

MS calc. 4861.0; found: 4862.7

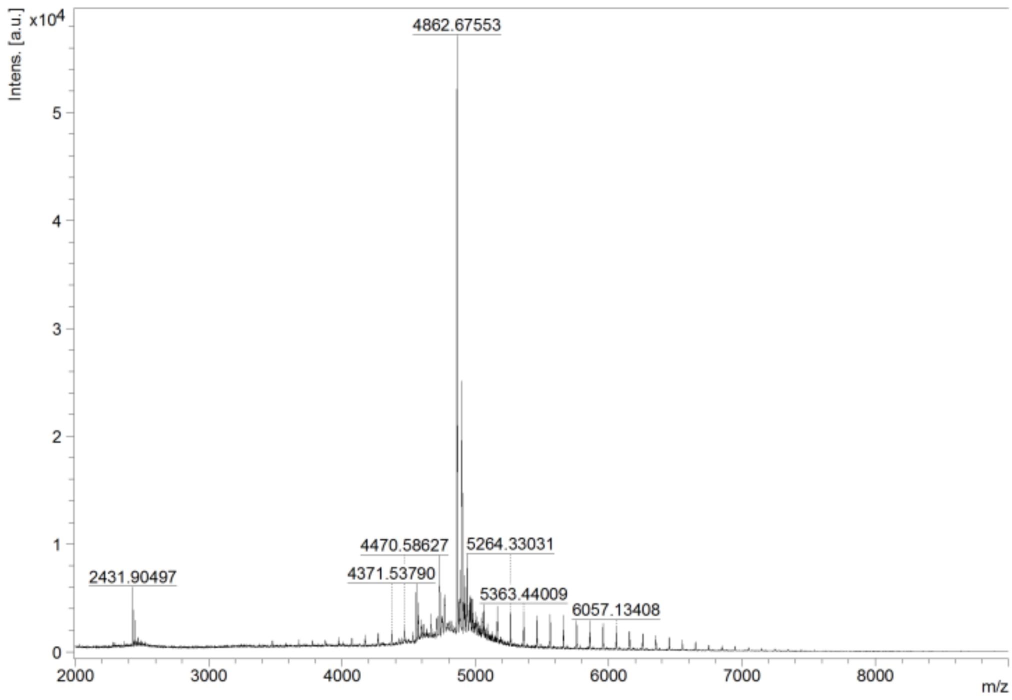

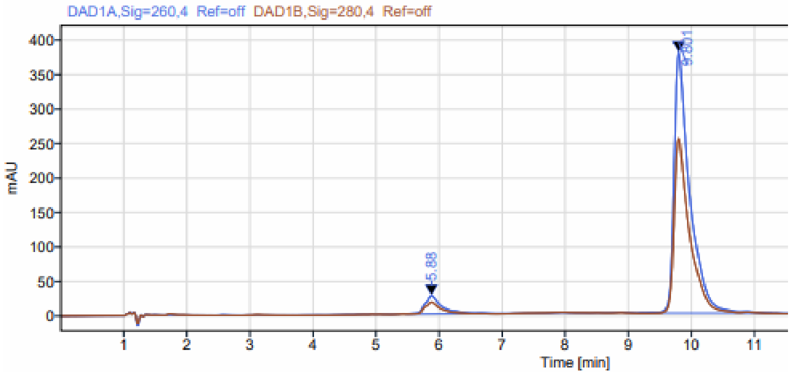

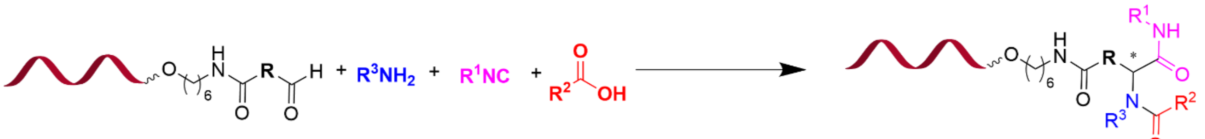

ATGC-1 2 3 4 ATGC-5

Table S13. Product conversions of products of the Ugi four-component reaction with different aldehydes coupled to 14mer ATGC.

No.	R	R^1	R^2	R^3	Product conversion
ATGC-5a	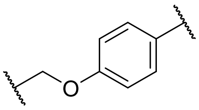	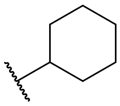	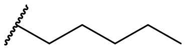	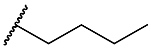	96%
ATGC-5b	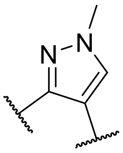	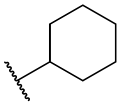	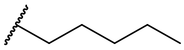	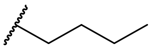	79%
ATGC-5c	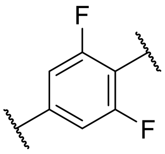	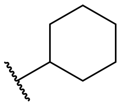	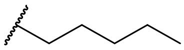	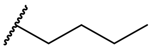	90%
ATGC-5d	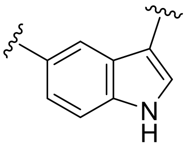	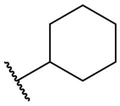	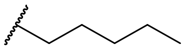	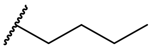	57%

Ugi four-component reaction with a mixture of 14mer 7dATC-coupled aldehydes

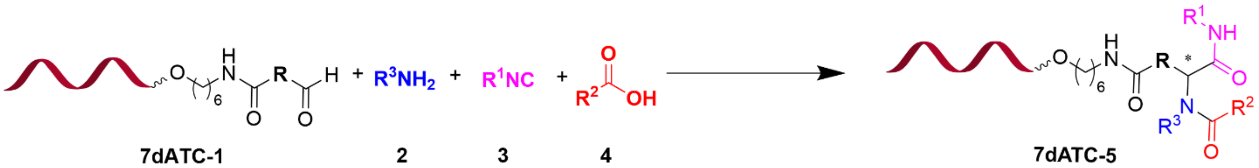

7dATC-1 2 3 4 7dATC-5

Table S14. MALDI-MS data of products of the Ugi four-component reaction with different aldehydes coupled to 14mer 7dATC. Mixture experiment.

MALDI-MS m/z MIXTURE						
No.	R	R1	R2	R3	calculated	found
MixUgi1	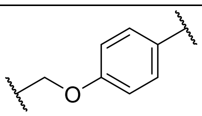	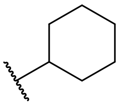	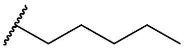	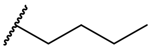	4868.0	4870.5
MixUgi2	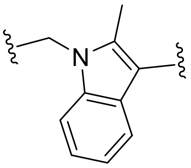	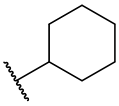	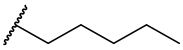	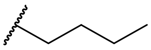	4905.0	4908.4
MixUgi3	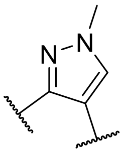	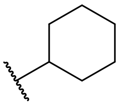	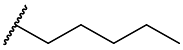	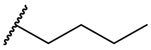	4842.0	4844.5
MixUgi4	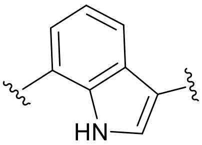	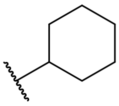	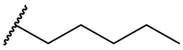	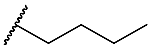	4877.0	4880.4

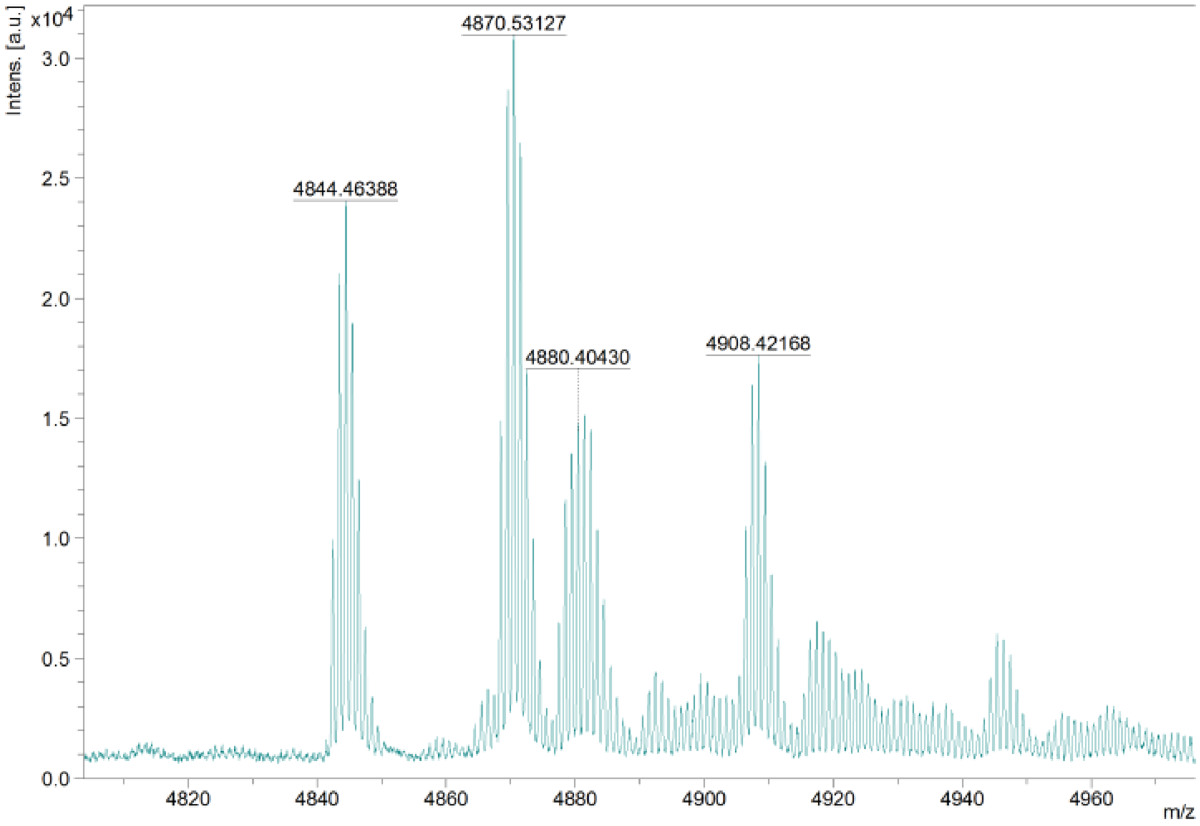

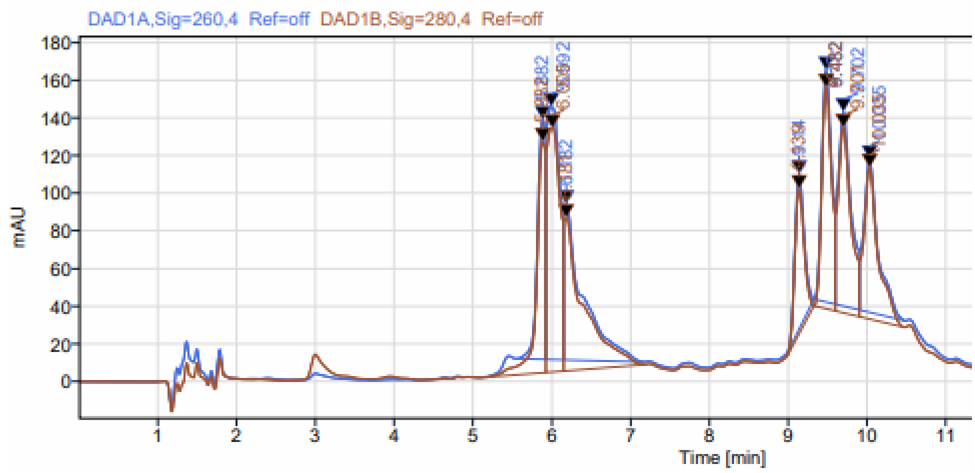

Reaction conditions: 7dATC-substrates (3 nmol), copolymer (90 nmol), primary amine **2** (6 µmol) in MeOH/$CHCl_3$ (50 µL; 3:1, v/v) for 3h at rt. Addition of isocyanide **3** (6 µmol) and carboxylic acid **4** (6 µmol). The reaction was run for 16h at 50°C.

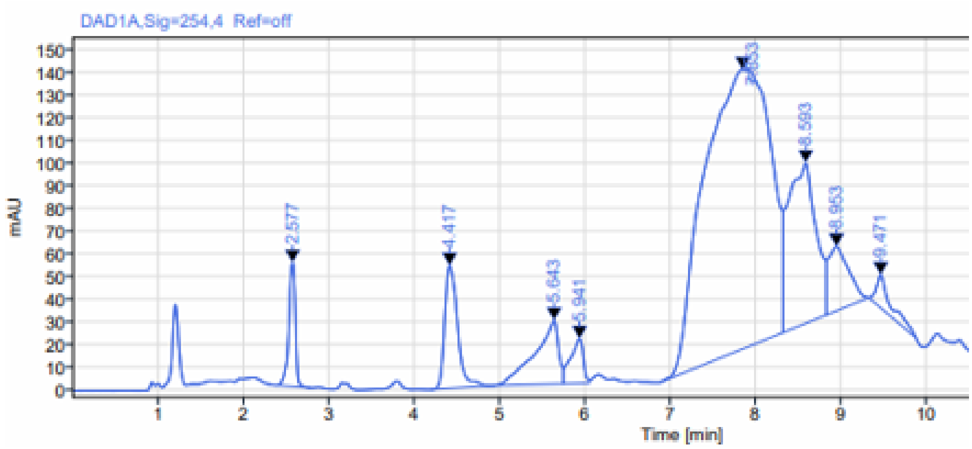

Reaction conditions: 7dATC-substrates (3 nmol), copolymer (90 nmol), primary amine **2** (6 µmol), isocyanide **3** (6 µmol) and carboxylic acid **4** (6 µmol) in $CF_3CH_2OH/CHCl_3$ (50 µL; 3:1, v/v). The reaction was run for 48h at 60°C.

Ugi-azide four-component reaction with 14mer 7dATC-coupled aldehydes

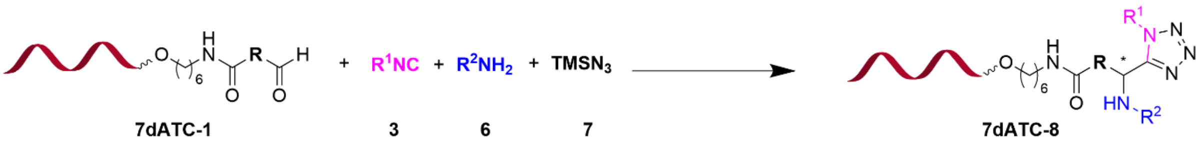

7dATC-1 3 6 7 7dATC-8

Table S15. MALDI-MS data of products of the Ugi-azide four-component reaction with different aldehydes coupled to 14mer 7dATC.

				MALDI-MS m/z	
No.	R	R^1	R^2	calculated	found
7dATC-8a	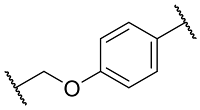	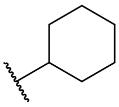	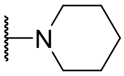	4808.0	4810.0
7dATC-8b	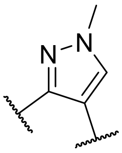	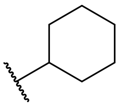	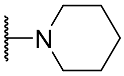	4782.0	4783.8
7dATC-8c	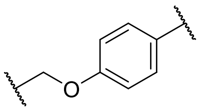	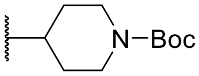	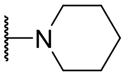	4908.0	4912.4
7dATC-8d	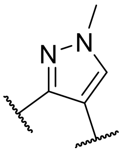	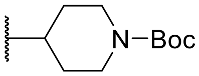	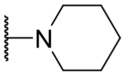	4882.0	4885.0

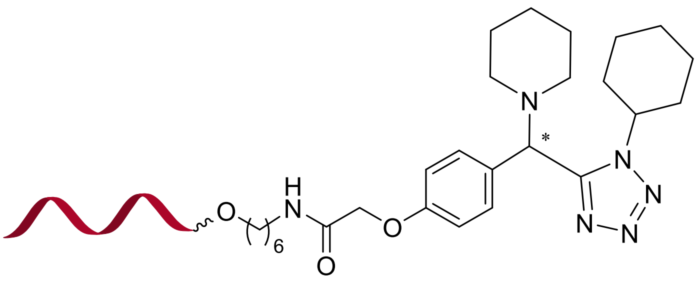

7dATC-8a

MS calc. 4808.0; found: 4810.0

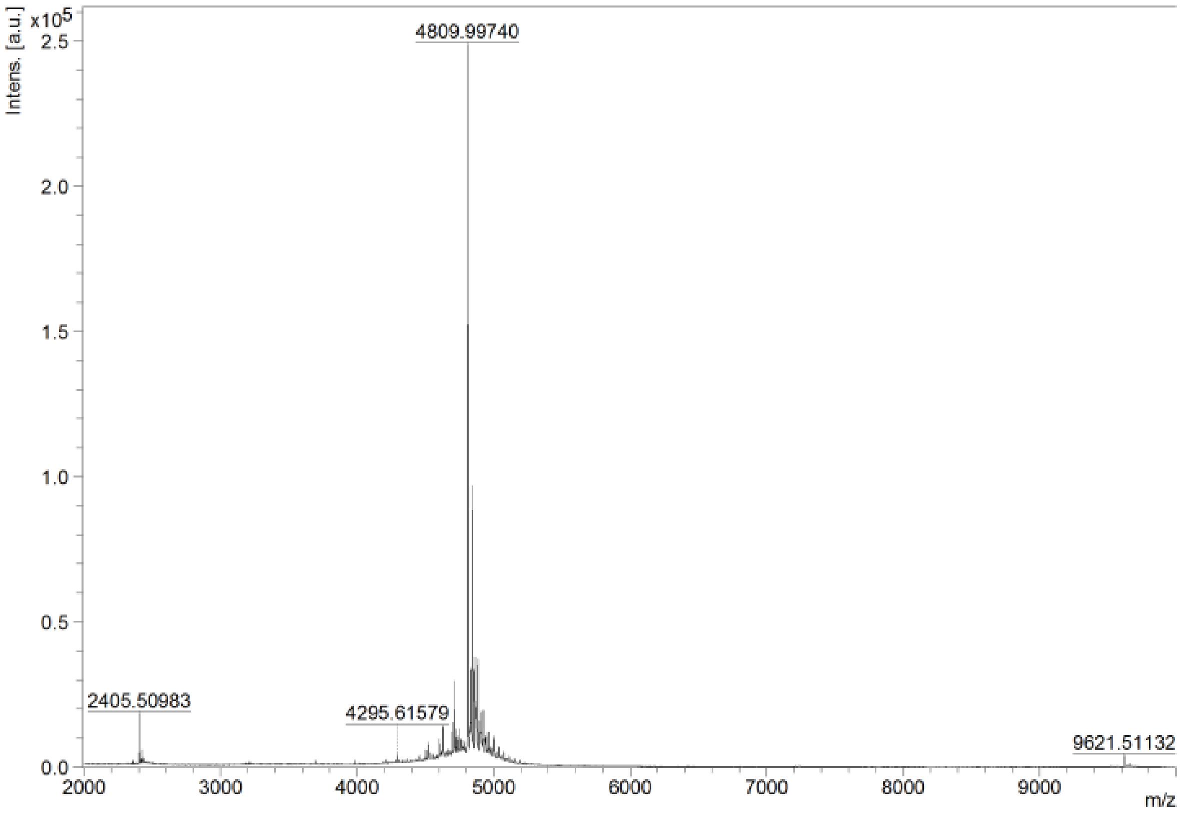

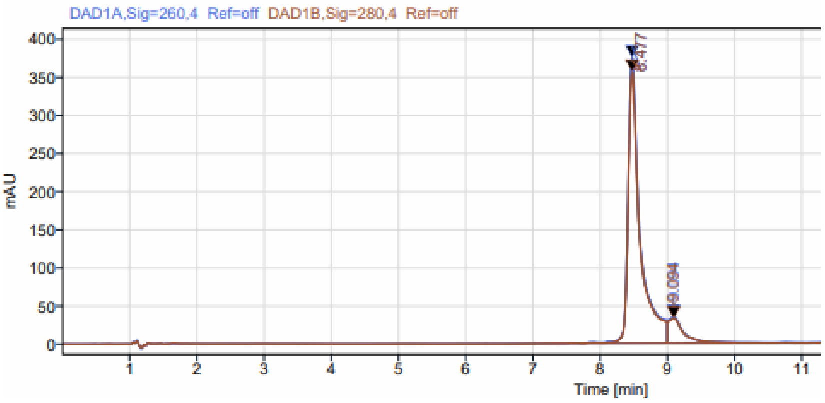

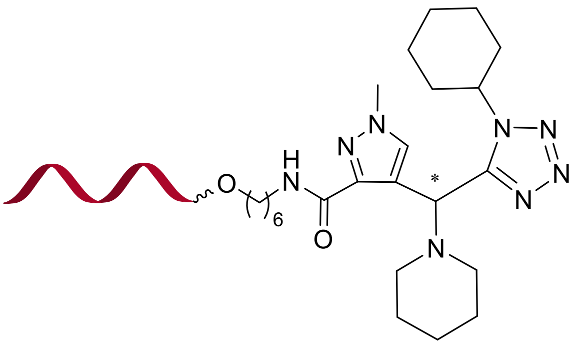

7dATC-8b

MS calc. 4782.0; found: 4783.8

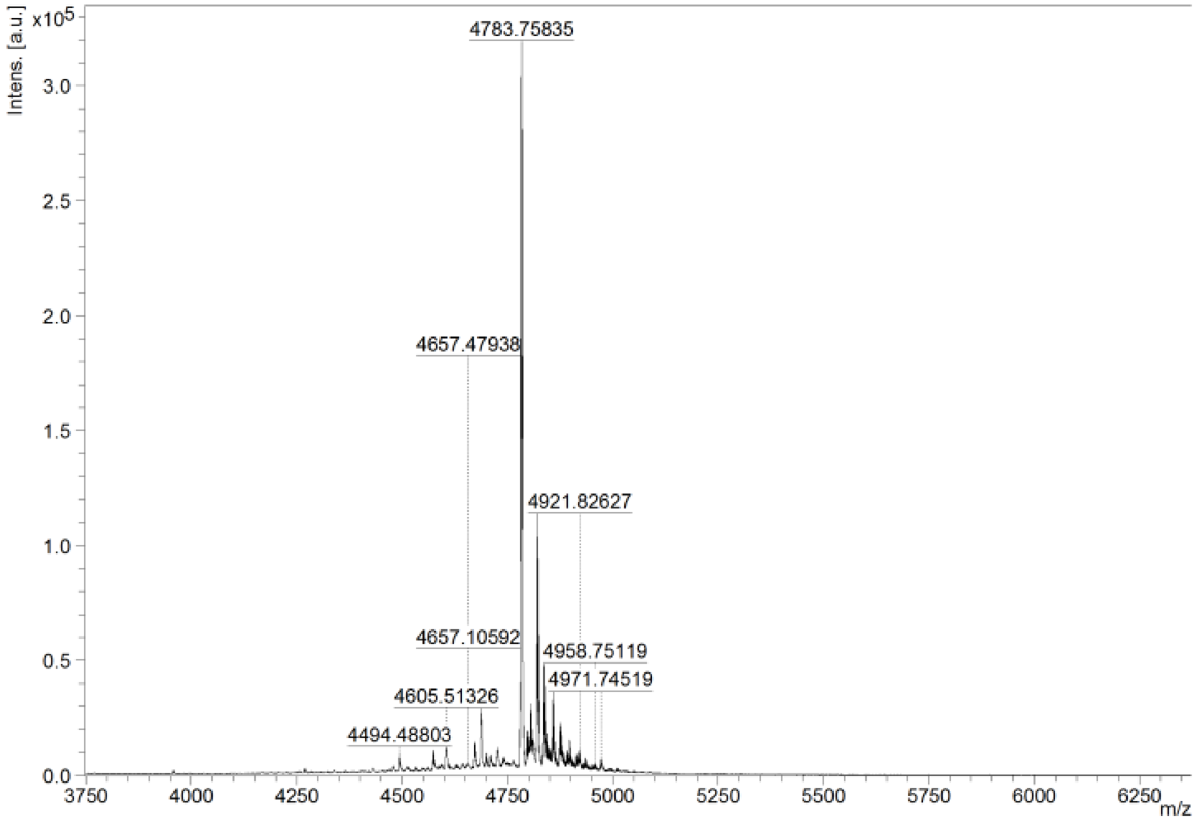

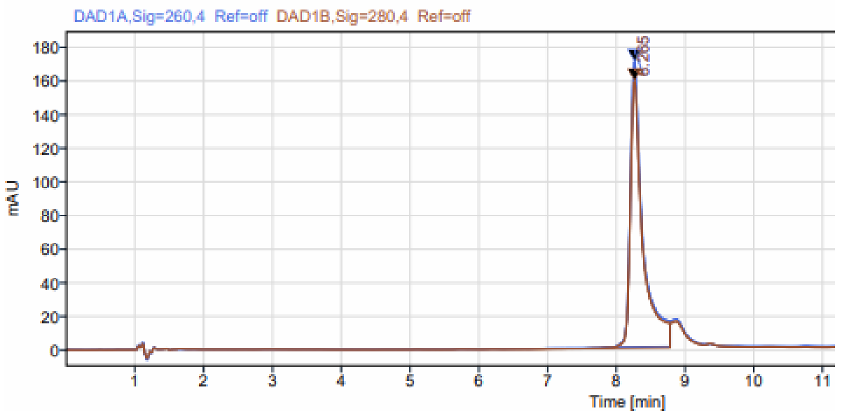

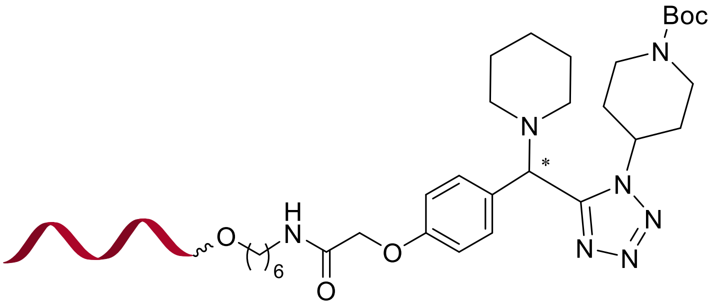

7dATC-8c

MS calc. 4908.0; found: 4912.4

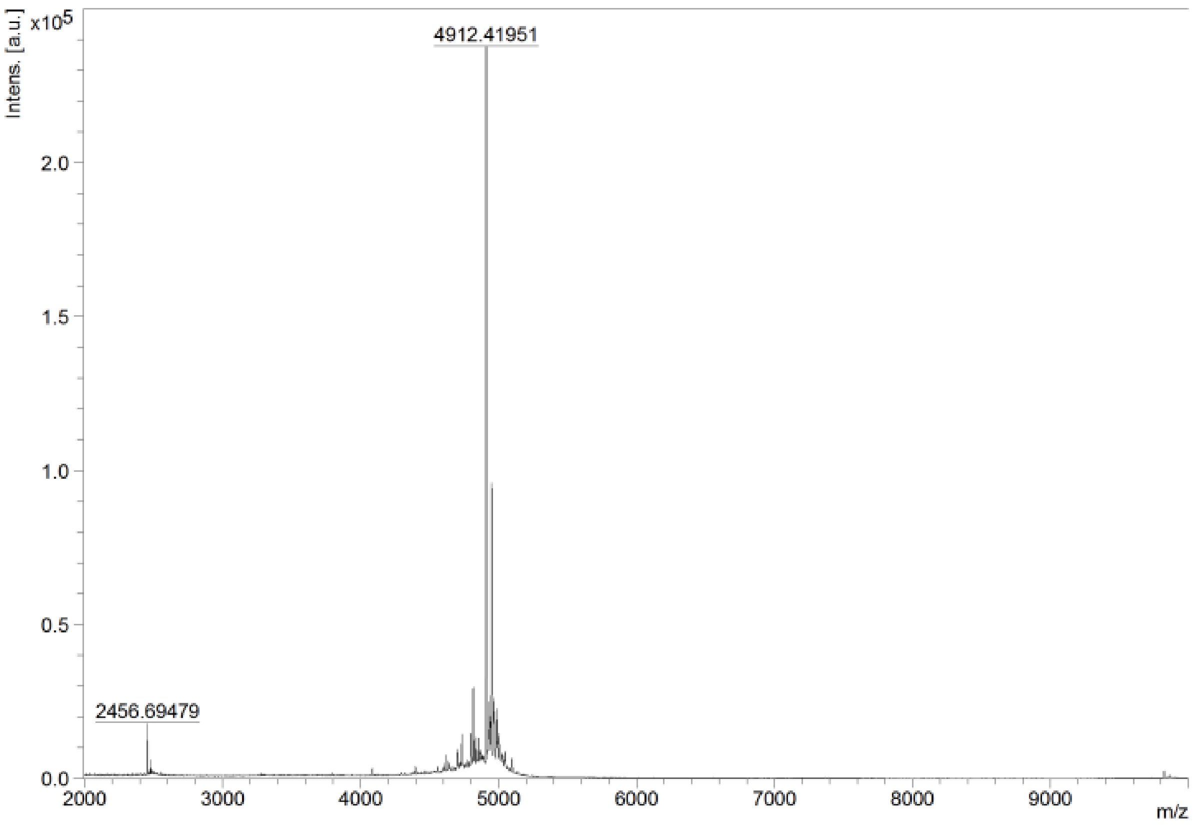

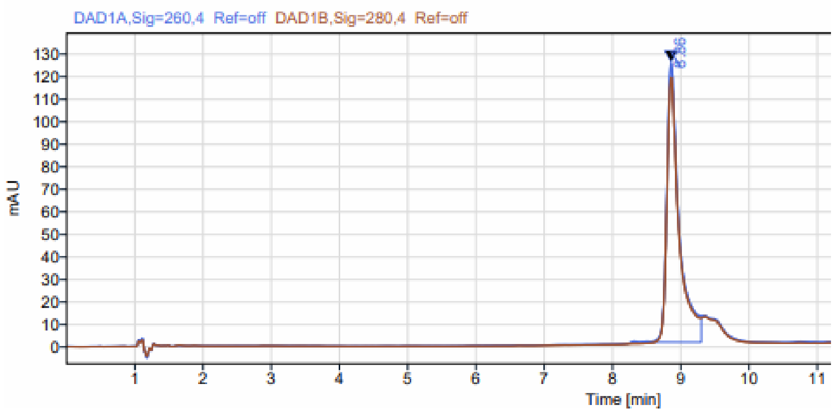

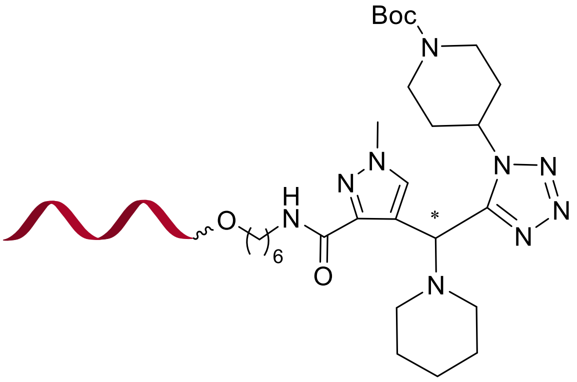

7dATC-8d

MS calc. 4882.0; found: 4885.0

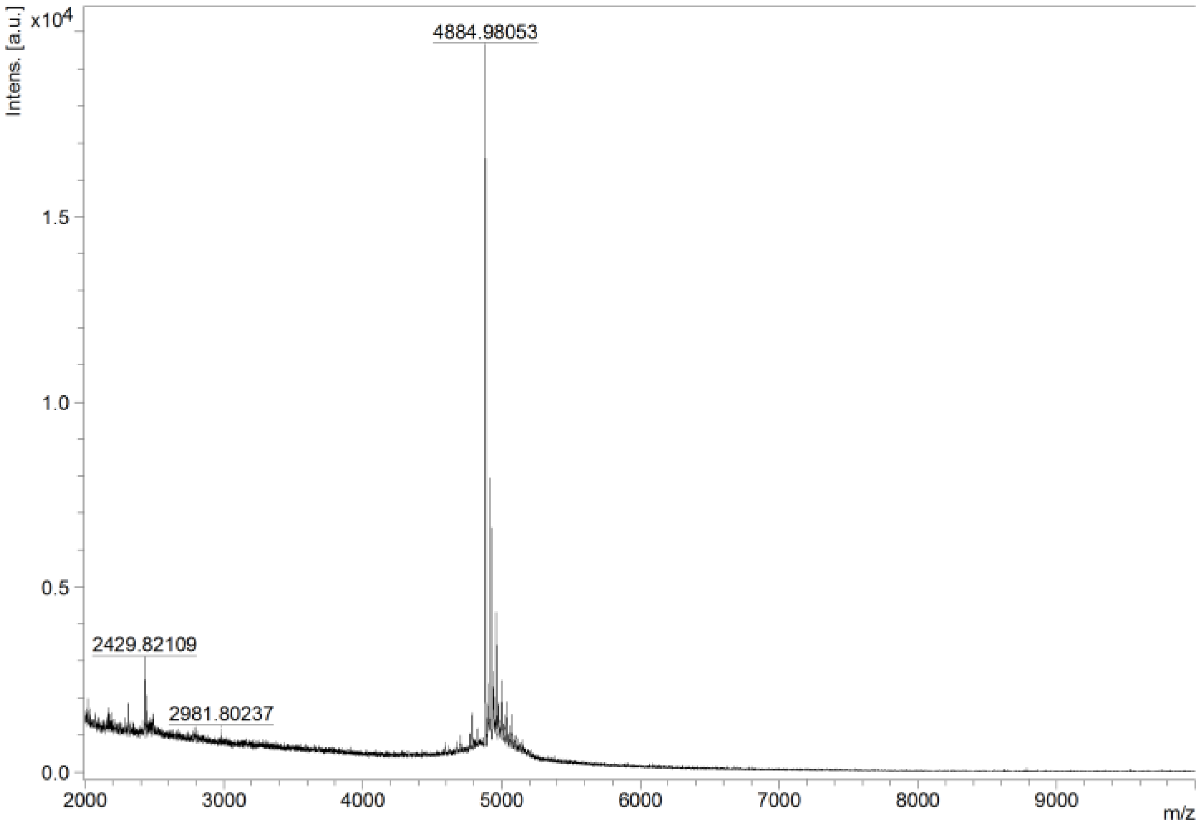

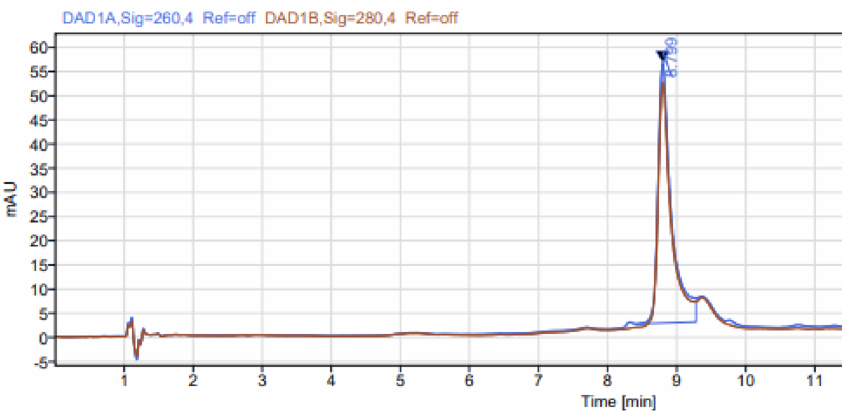

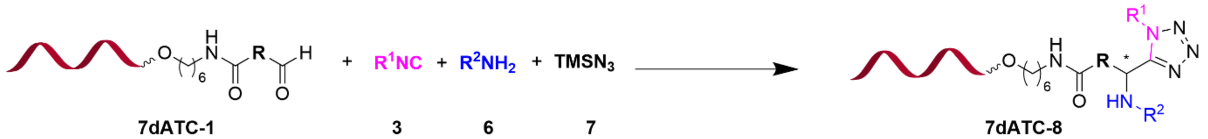

7dATC-1 3 6 7 7dATC-8

Table S16. Product conversions of products of the Ugi-azide four-component reaction with different aldehydes coupled to 14mer 7dATC.

No.	R	R^1	R^2	Product conversion
7dATC-8a	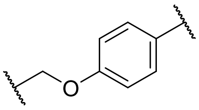	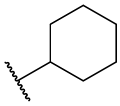	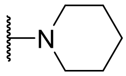	77%
7dATC-8b	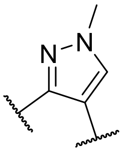	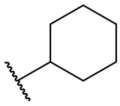	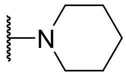	52%
7dATC-8c	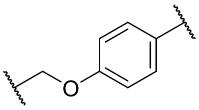	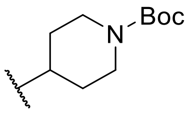	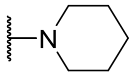	36%
7dATC-8d	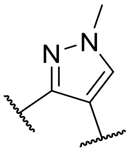	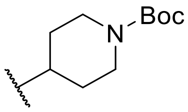	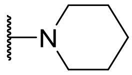	41%

Ugi-azide four-component reaction with 14mer ATGC-coupled aldehydes

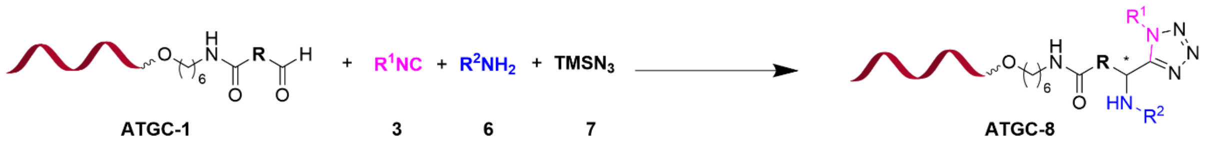

Table S17. MALDI-MS data of products of the Ugi-azide four-component reaction with different aldehydes coupled to 14mer ATGC.

				MALDI-MS m/z	
No.	R	R^1	R^2	calculated	found
ATGC-8a	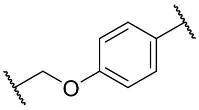	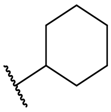	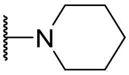	4799.0	4801.7
ATGC-8b	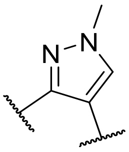	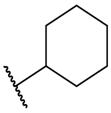	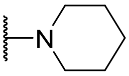	4764.0	4766.7
ATGC-8c	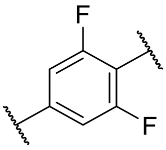	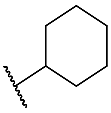	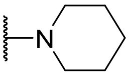	4805.0	4807.8

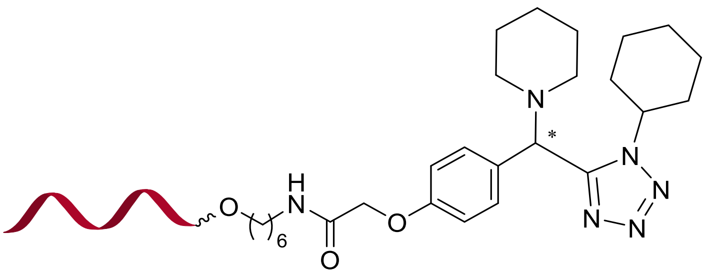

ATGC-8a

MS calc. 4799.0; found: 4801.7

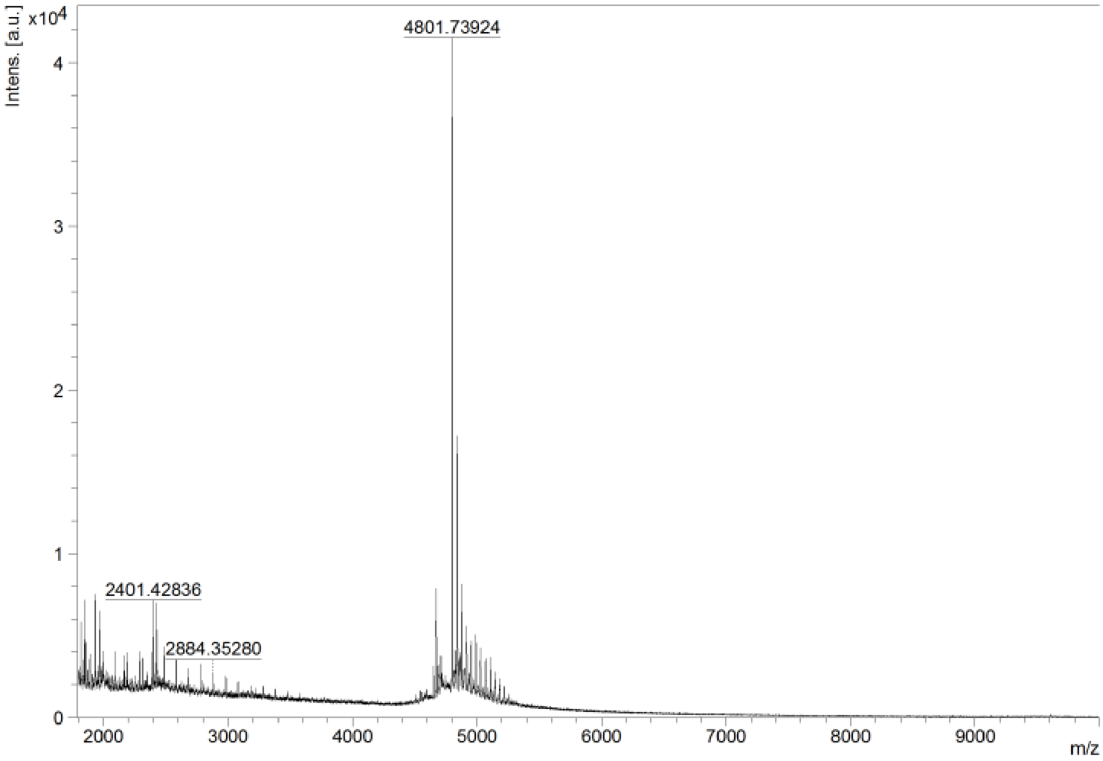

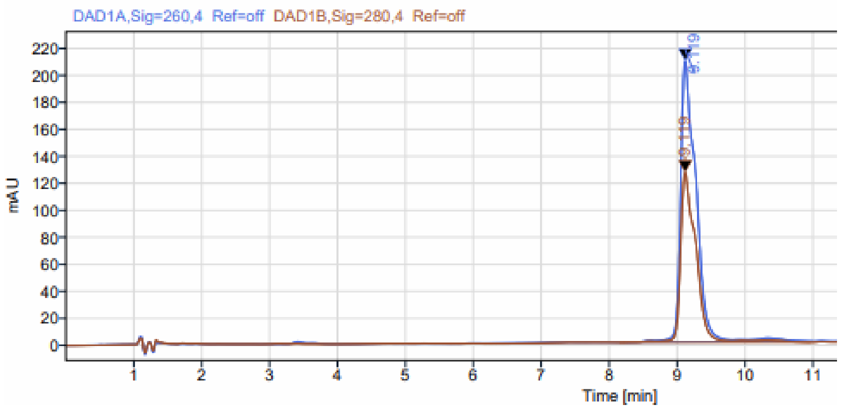

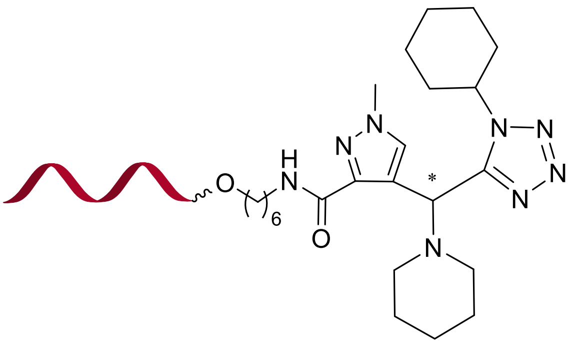

ATGC-8b

MS calc. 4794.0; found: 4766.7

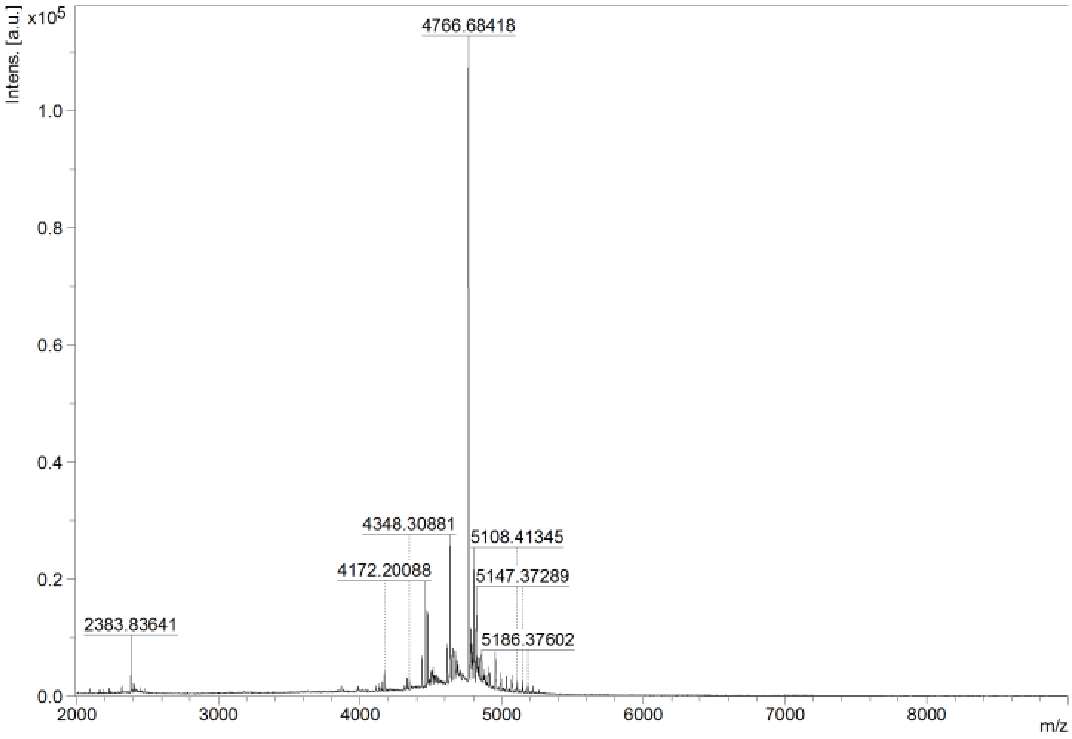

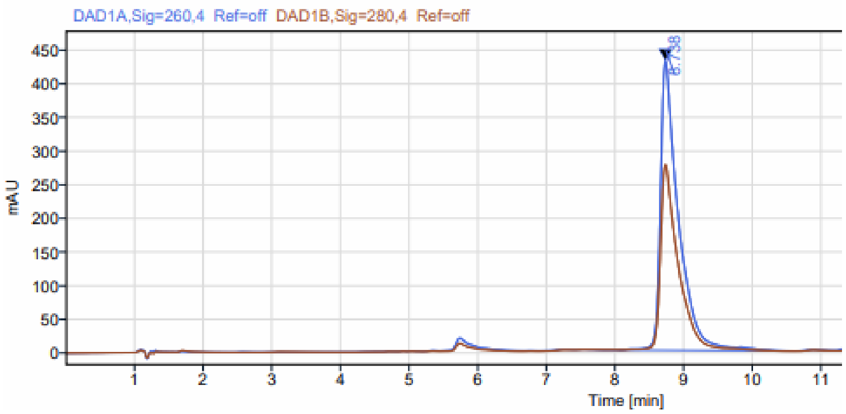

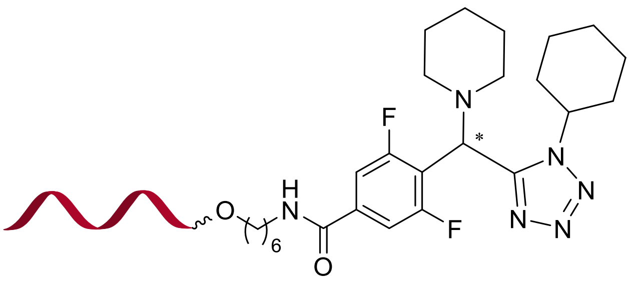

ATGC-8c

MS calc. 4805.0; found: 4807.8

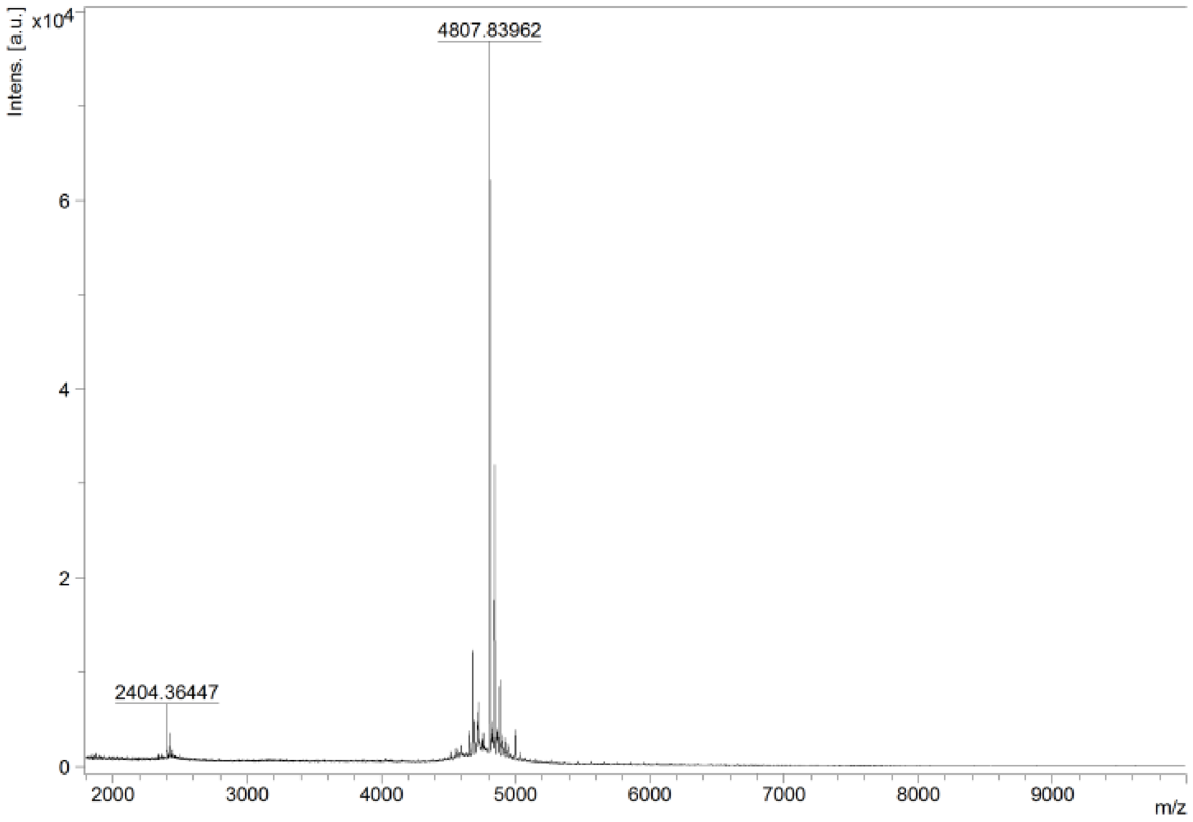

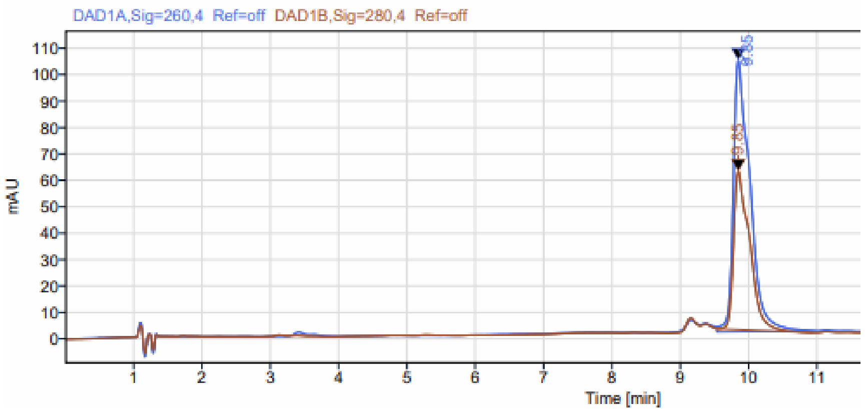

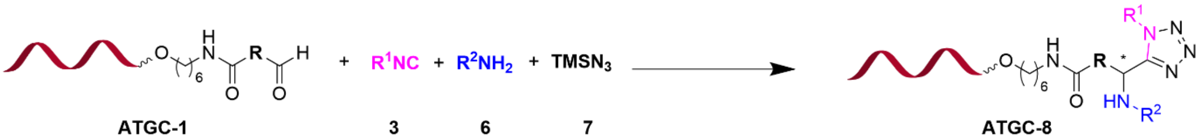

ATGC-1 3 6 7 ATGC-8

Table S18. Product conversions of products of the Ugi-azide four-component reaction with different aldehydes coupled to 14mer ATGC.

No.	R	R^1	R^2	Product conversion
ATGC-8a	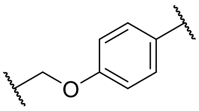	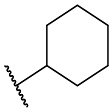	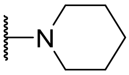	79%
ATGC-8b	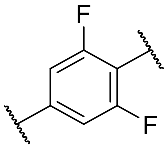	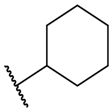	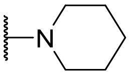	62%
ATGC-8c	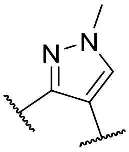	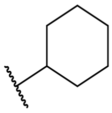	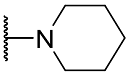	60%

Ugi-azide four-component reaction with a mixture of 14mer 7dATC-coupled aldehydes

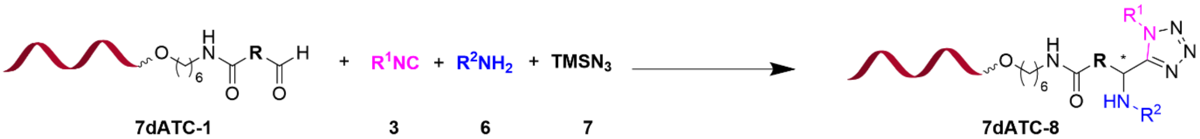

7dATC-1 3 6 7 7dATC-8

Table S19. MALDI-MS data of products of the Ugi-azide four-component reaction with different aldehydes coupled to 14mer 7dATC. Mixture experiment.

MALDI-MS m/z MIXTURE					
No.	R	R^1	R^2	calculated	found
MixUA1	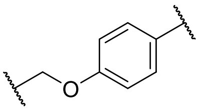	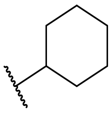	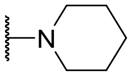	4808.0	4809.5
MixUA2	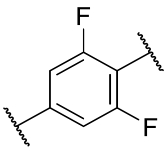	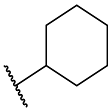	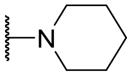	4816.0	4816.4
MixUA3	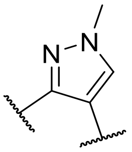	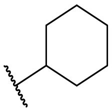	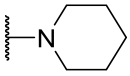	4782.0	4783.4
MixUA4	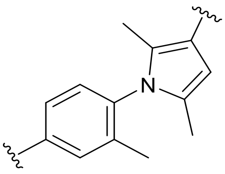	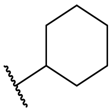	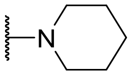	4886.0	4885.5

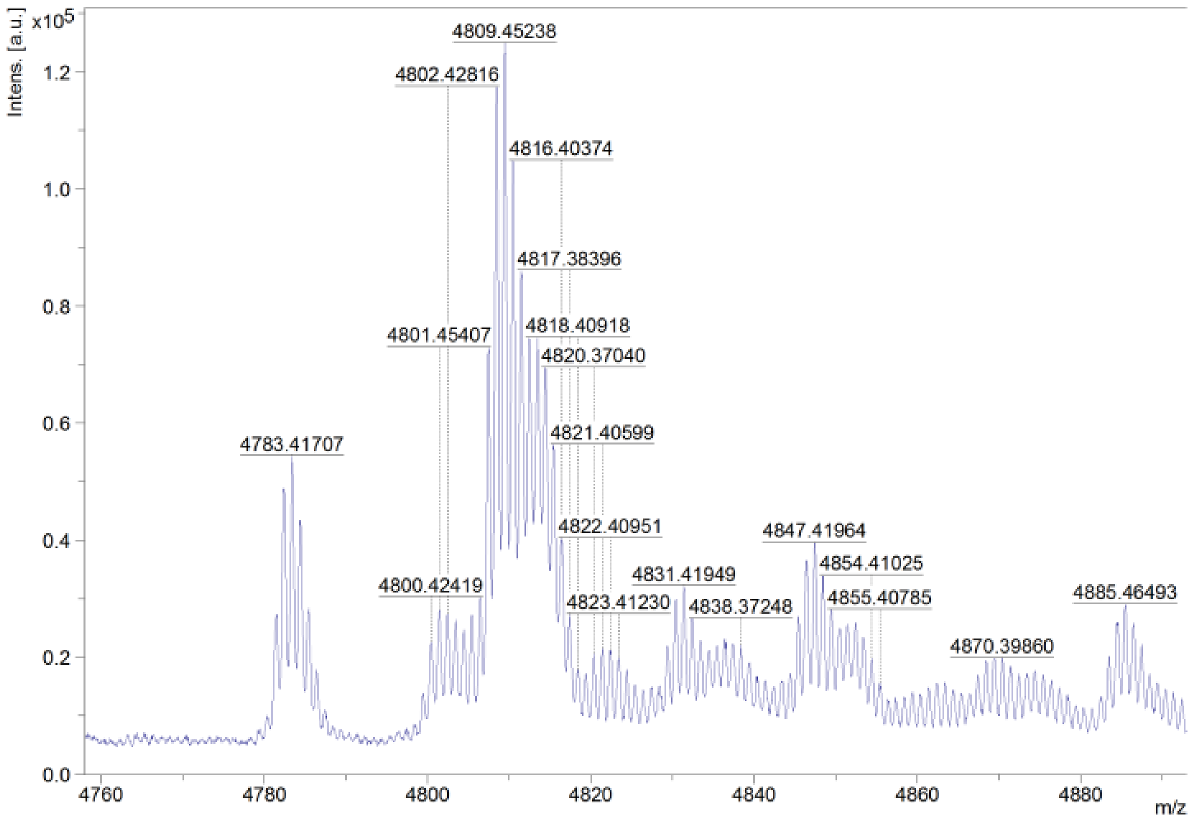

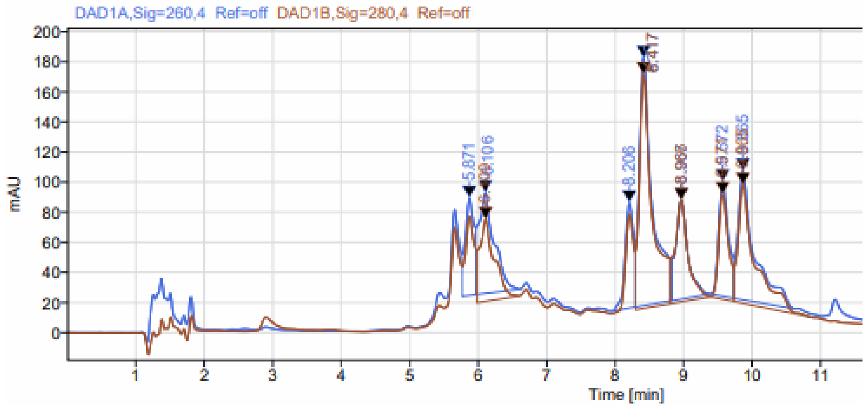

Reaction conditions: 7dATC-substrates (3 nmol), copolymer (90 nmol) and secondary amine **6** (6 µmol) in MeOH/$CHCl_3$ (50 µL; 3:1, v/v) for 3h at rt. Addition of isocyanide **3** (6 µmol) and $TMSN_3$ **7** (6 µmol). The reaction was run for 16h at 50°C.

Groebke-Blackburn-Bienaymé three-component reaction with 14mer 7dATC-coupled aldehydes

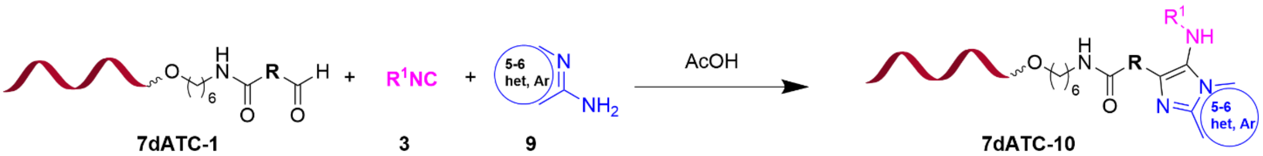

7dATC-1 3 9 7dATC-10

Table S20. MALDI-MS data of products of the Groebke-Blackburn-Bienaymé three-component reaction with different aldehydes coupled to 14mer 7dATC.

			MALDI-MS m/z		
No.	R	R^1	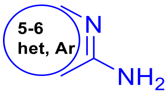	calculated	found
7dATC-10a	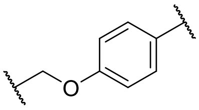	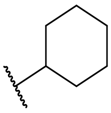	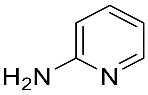	4774.0	4776.0
7dATC-10b	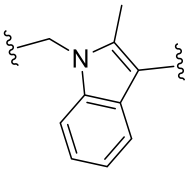	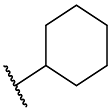	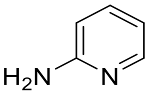	4811.0	4812.8
7dATC-10c	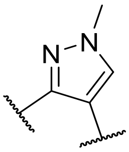	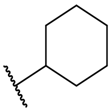	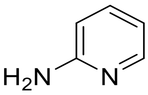	4748.0	4749.8
7dATC-10d	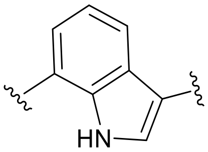	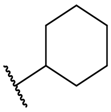	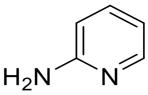	4783.0	4784.9
7dATC-10e	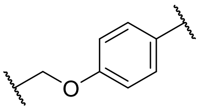	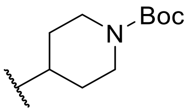	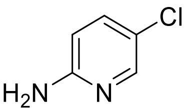	4910.0	4910.5

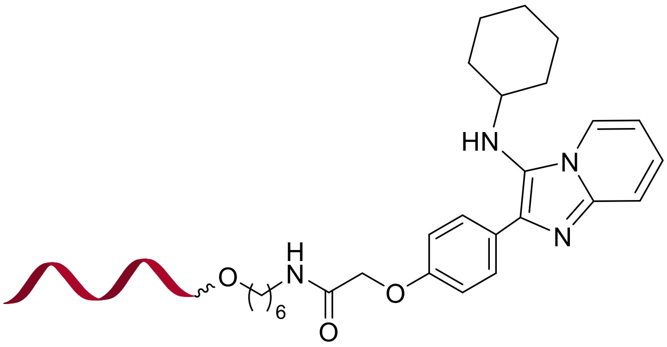

7dATC-10a

MS calc. 4774.0; found: 4776.0

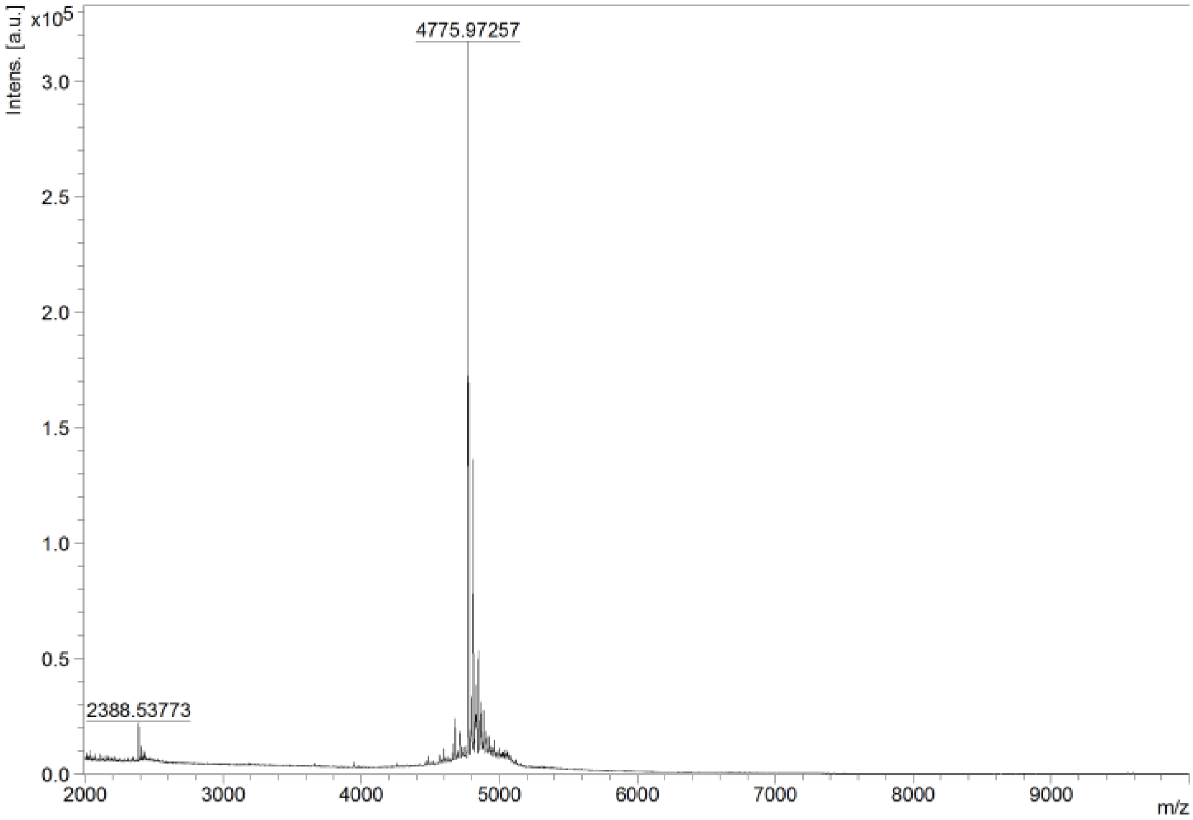

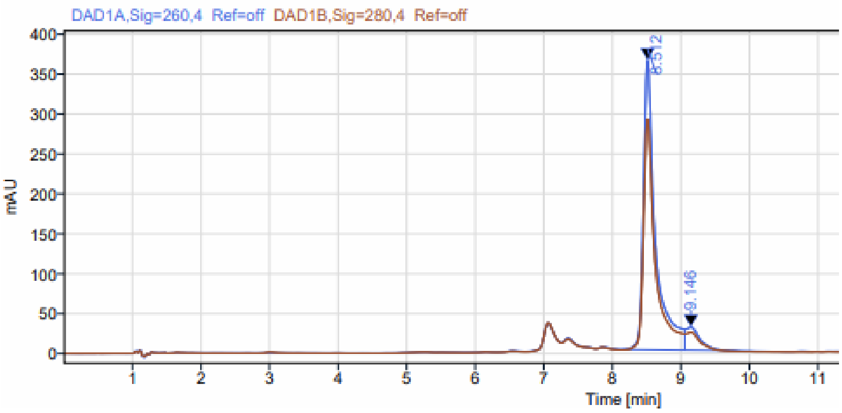

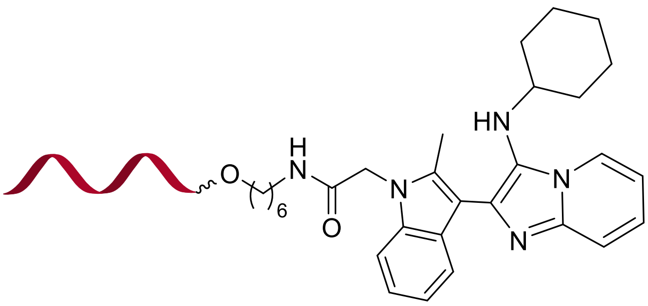

7dATC-10b

MS calc. 4811.0; found: 4812.8

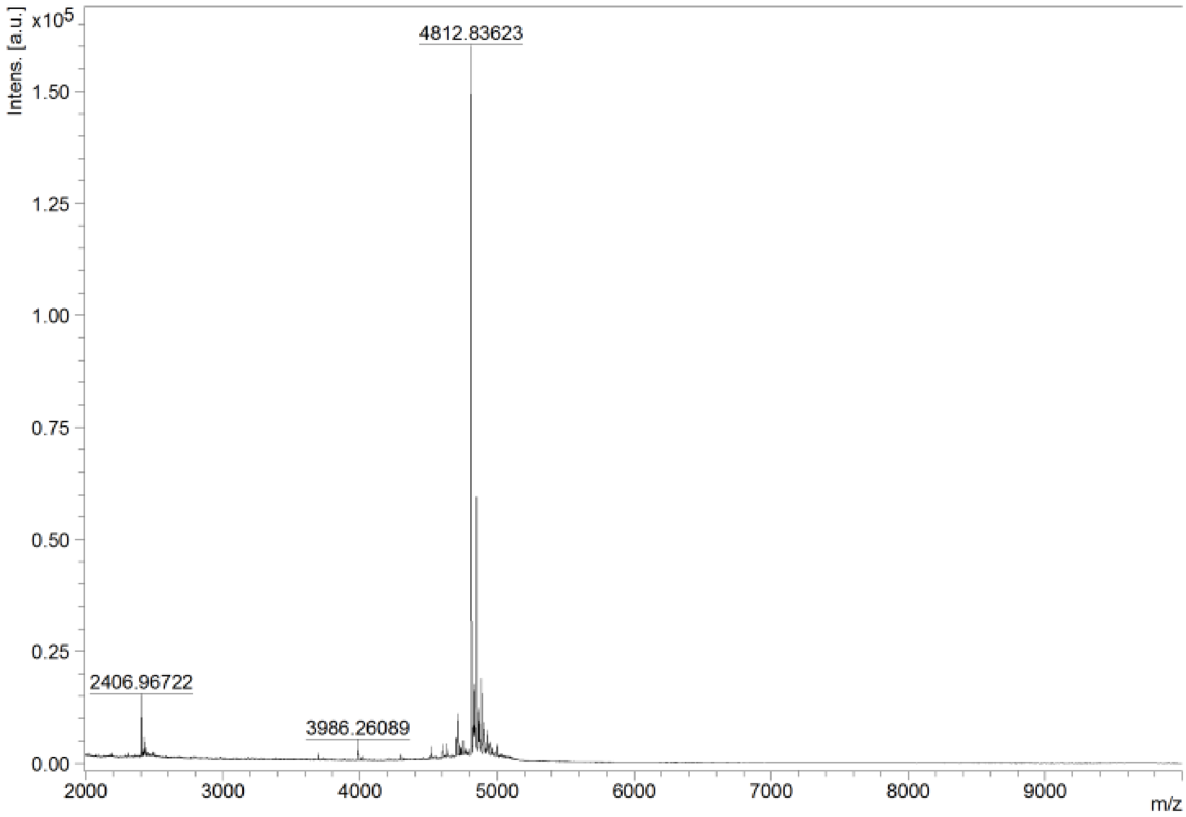

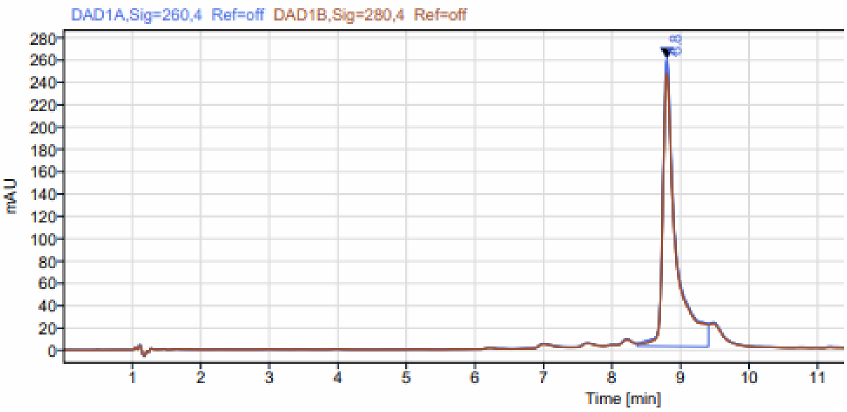

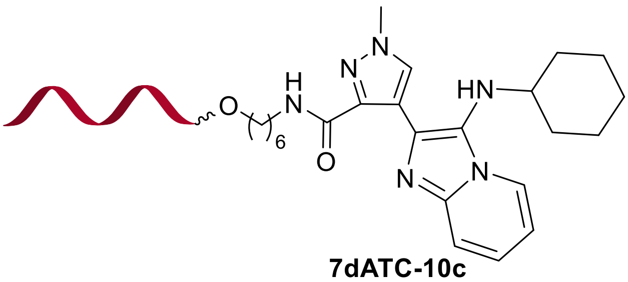

7dATC-10c

MS calc. 4748.0; found: 4749.8

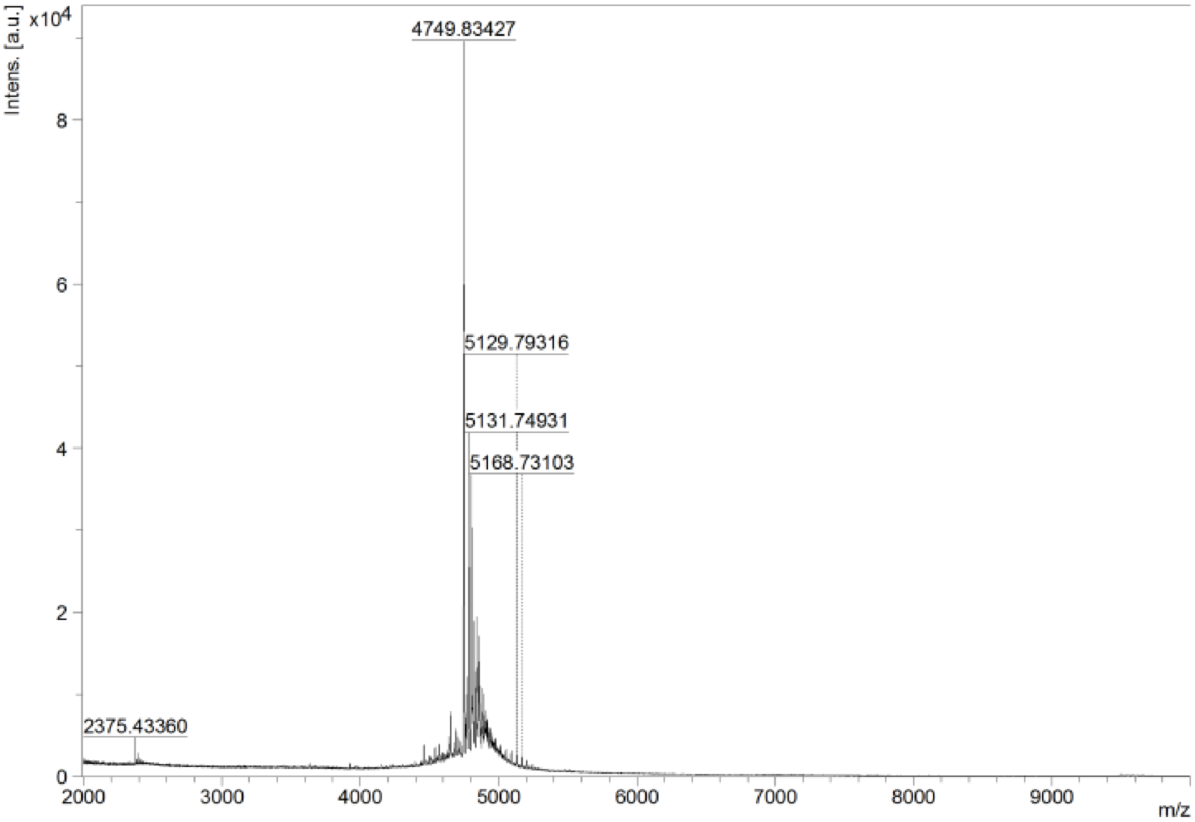

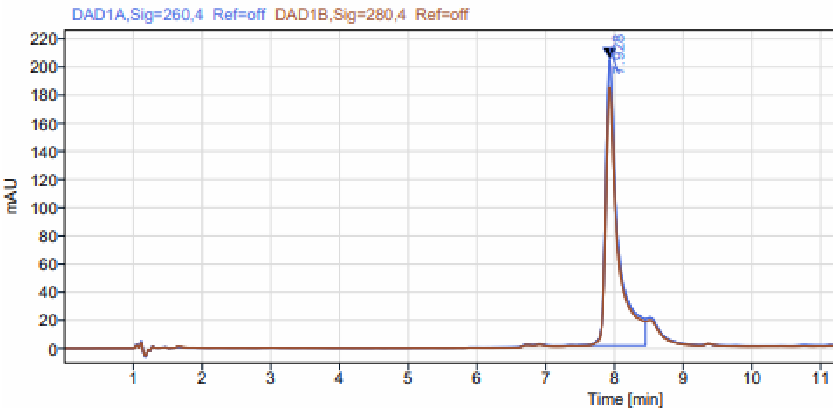

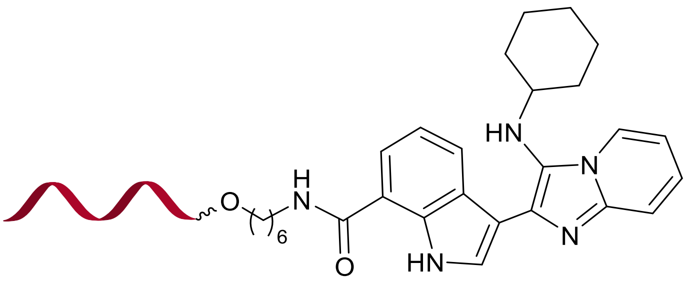

7dATC-10d

MS calc. 4783.0; found: 4784.9

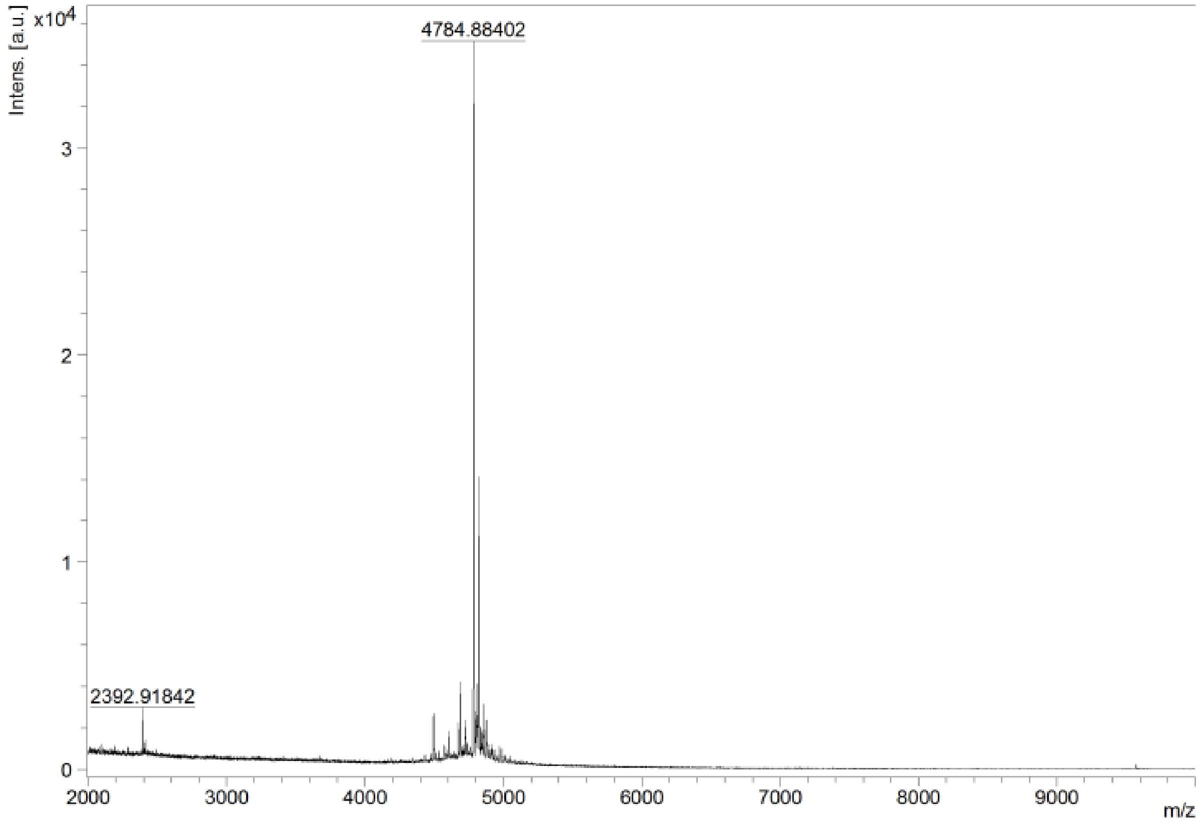

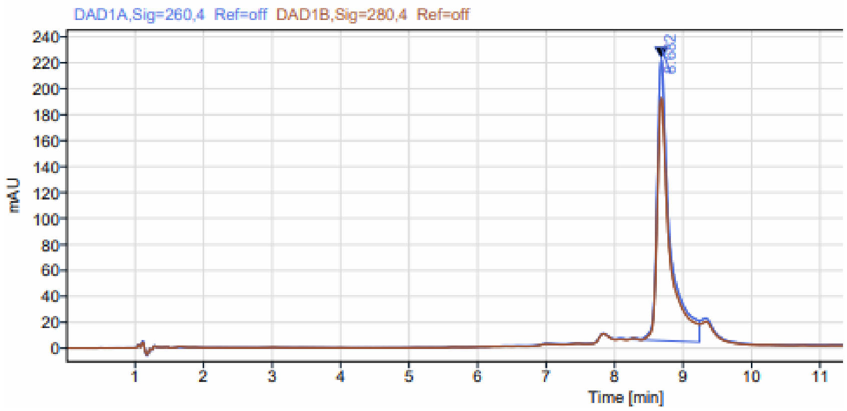

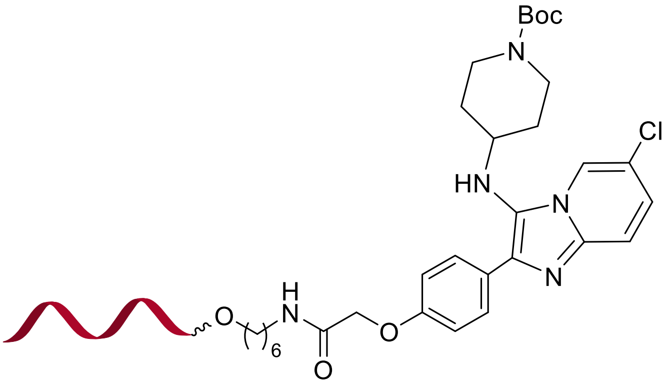

7dATC-10e

MS calc. 4910.0; found: 4910.5

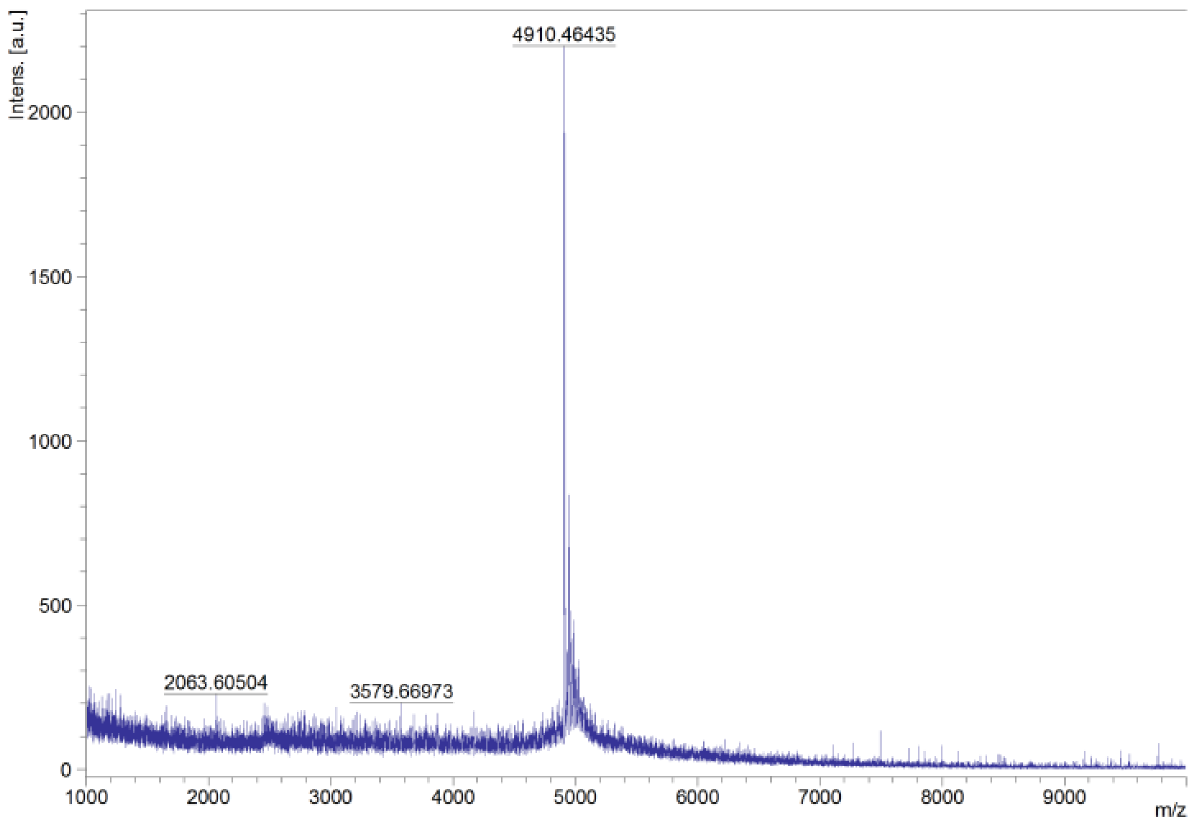

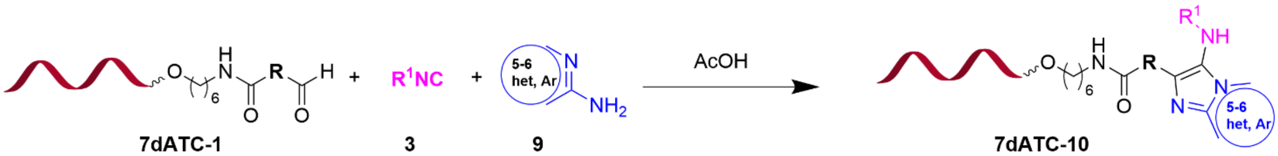

7dATC-1 **3** **9** **7dATC-10**

Table S21. Product conversions of products of the Groebke-Blackburn-Bienaymé three-component reaction with different aldehydes coupled to 14mer 7dATC.

**No.**	**R**	**R1**	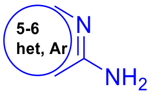	**Product conversion**
7dATC-10a	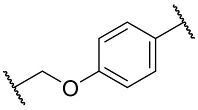	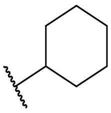	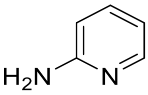	90%
7dATC-10b	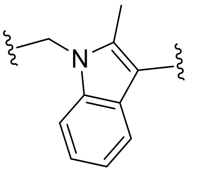	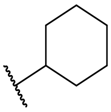	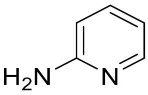	92%
7dATC-10c	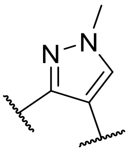	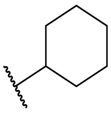	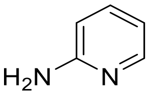	84%
7dATC-10d	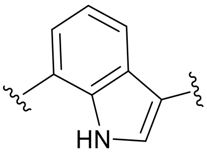	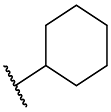	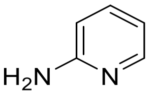	89%
7dATC-10e	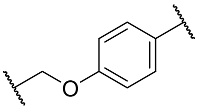	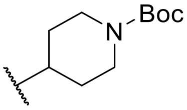	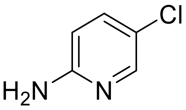	71%

Groebke-Blackburn-Bienaymé three-component reaction with 14mer ATGC-coupled aldehydes

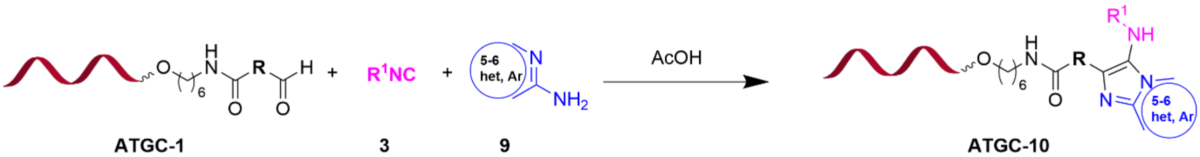

ATGC-1 3 9 ATGC-10

Table S22. MALDI-MS data of products of the Groebke-Blackburn-Bienaymé three-component reaction with different aldehydes coupled to 14mer ATGC.

				**MALDI-MS m/z**	
No.	R	R^1	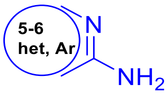	calculated	found
ATGC-10a	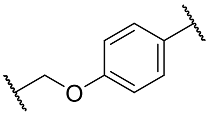	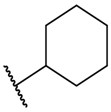	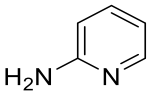	4765.0	4767.7
ATGC-10b	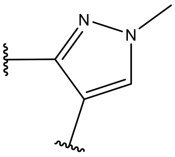	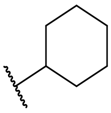	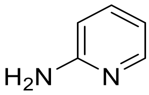	4730.0	4732.7
ATGC-10c	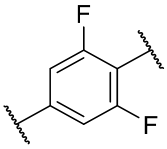	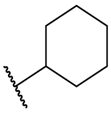	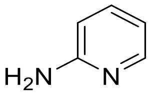	4771.0	4773.6
ATGC-10d	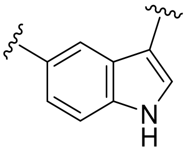	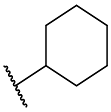	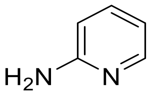	4765.0	4767.5

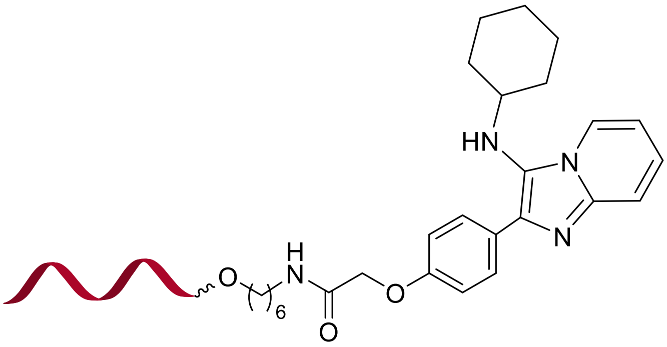

ATGC-10a

MS calc. 4765.0; found: 4767.7

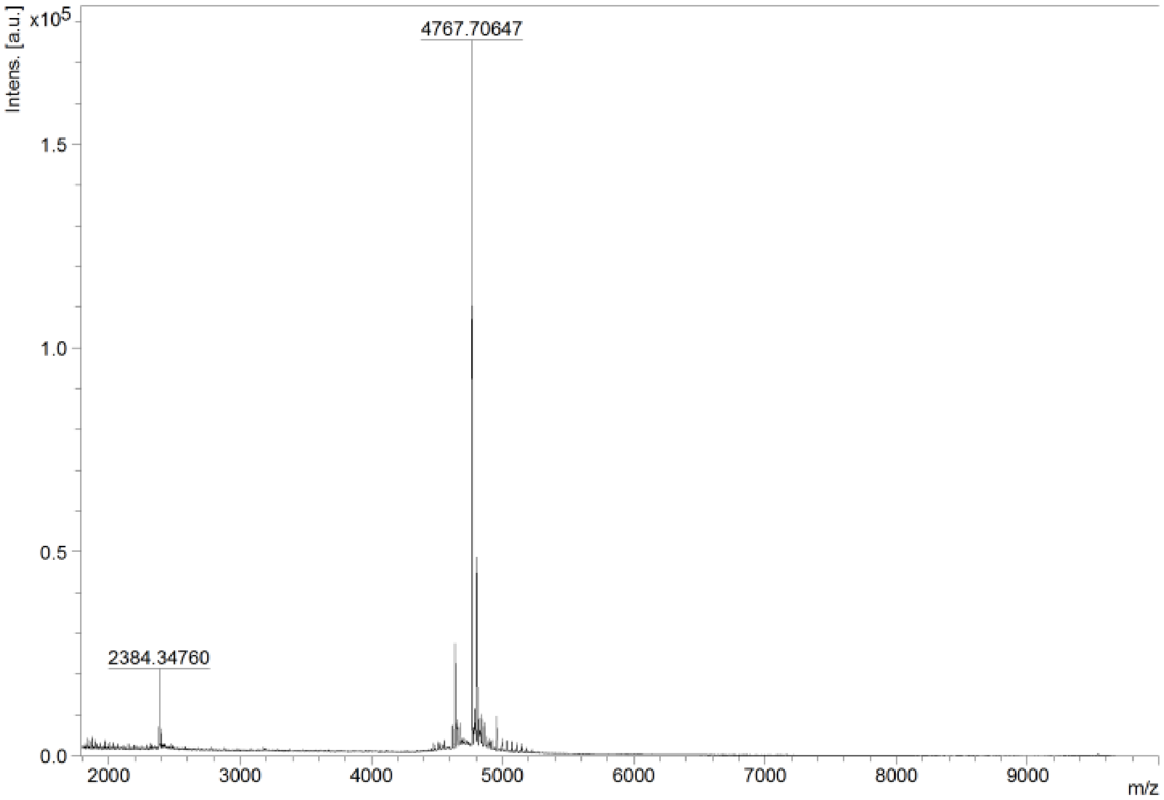

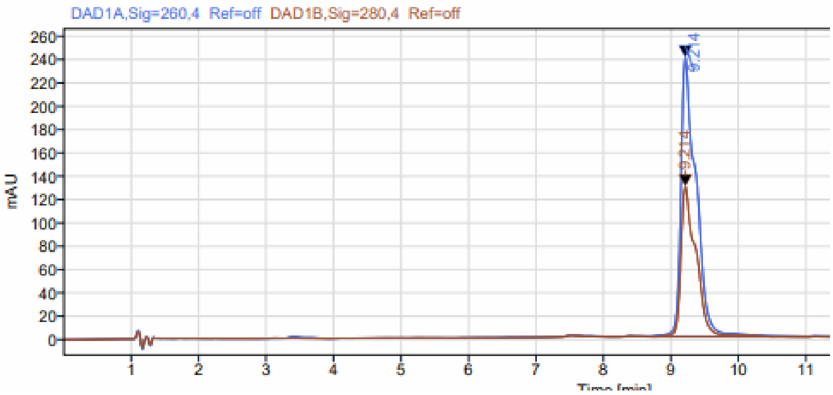

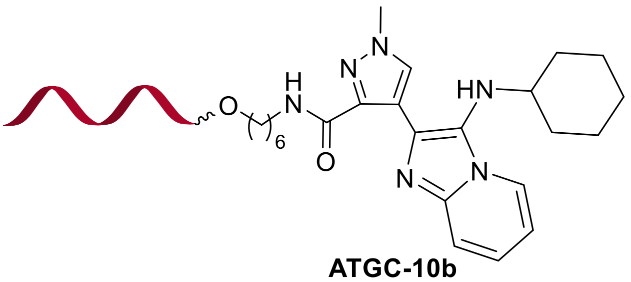

ATGC-10b

MS calc. 4730.0; found: 4732.7

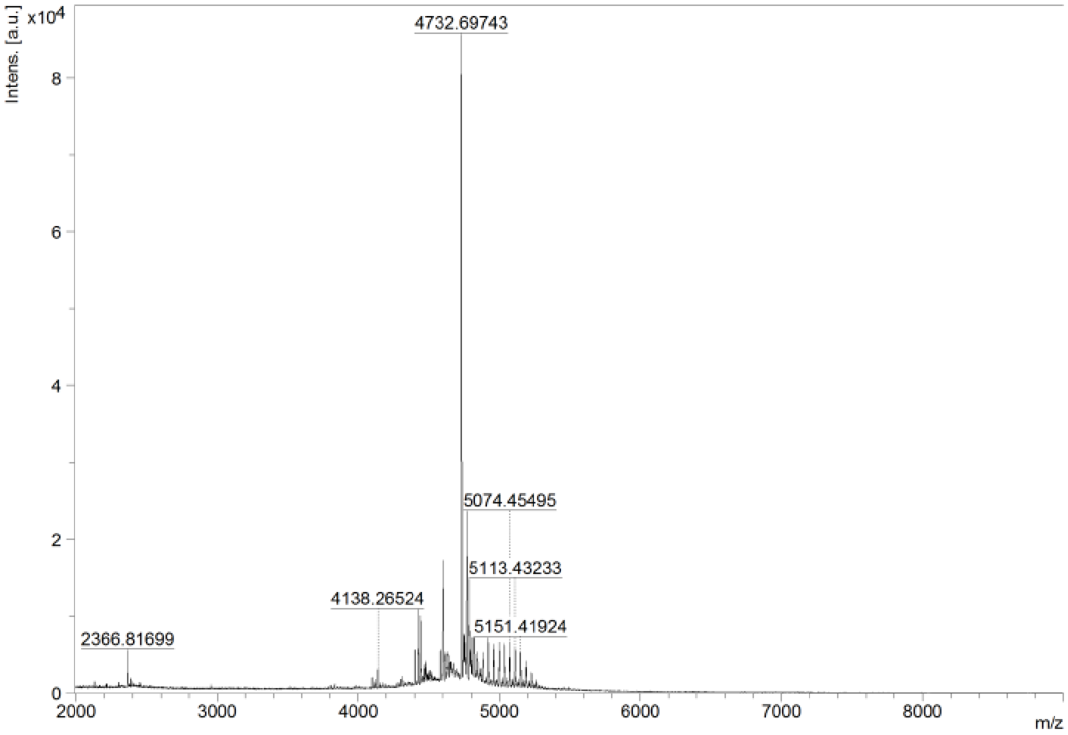

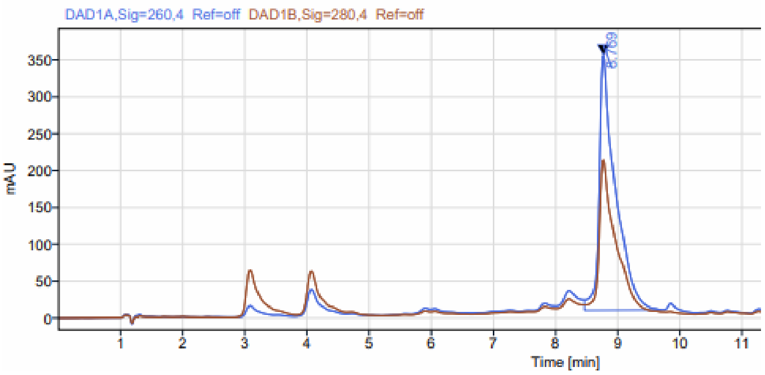

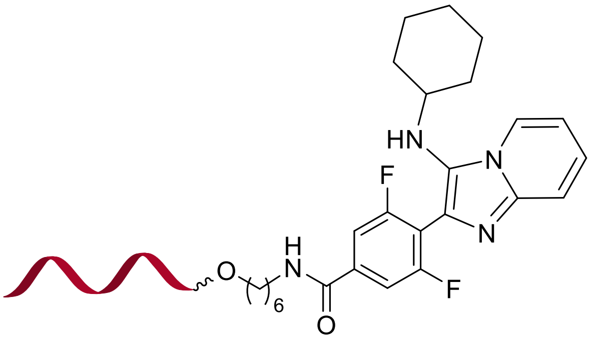

ATGC-10c

MS calc. 4771.0; found: 4773.6

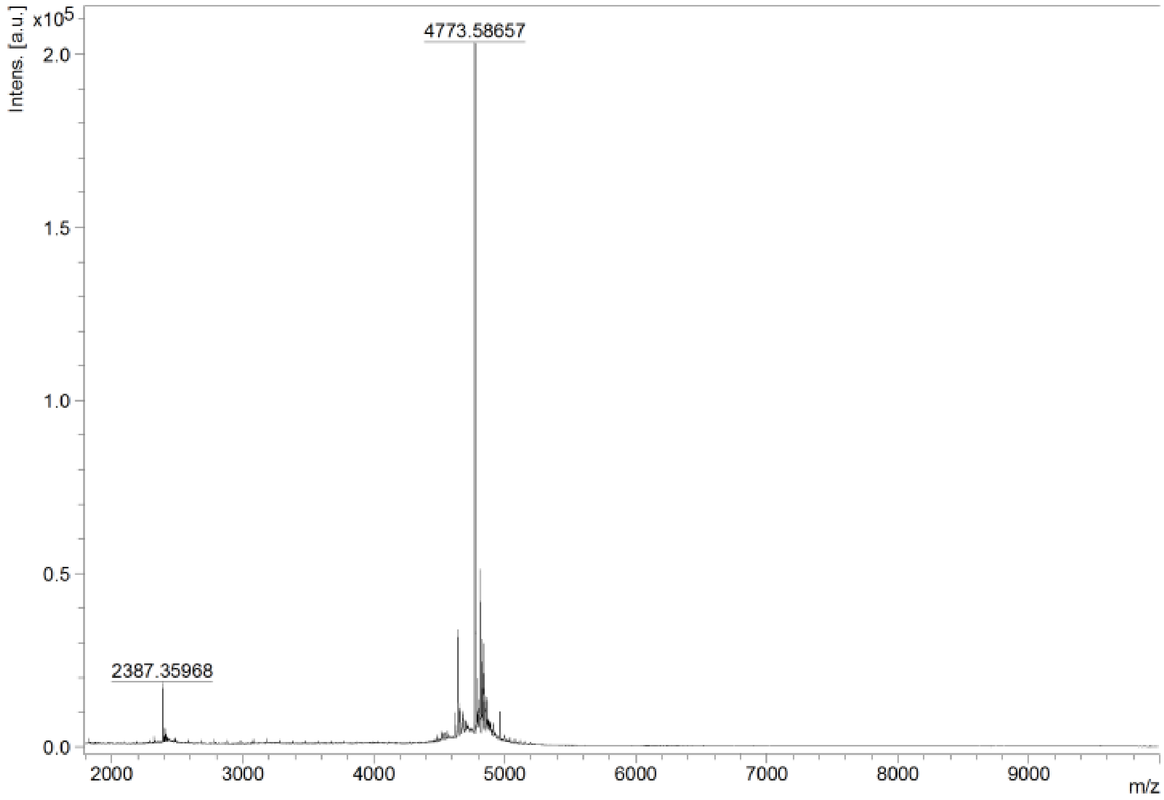

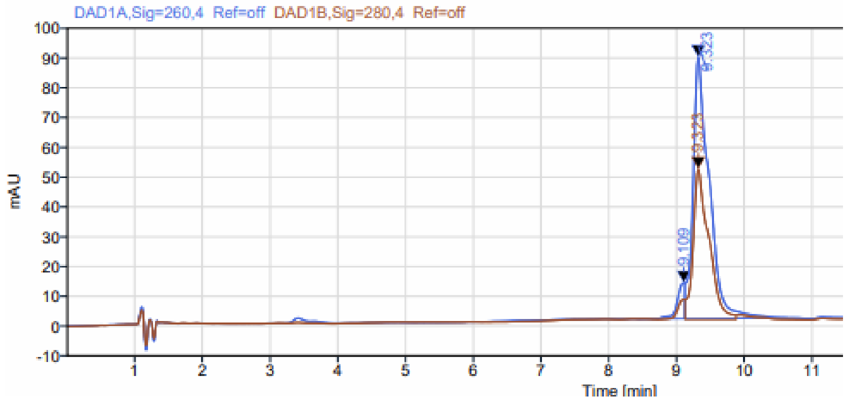

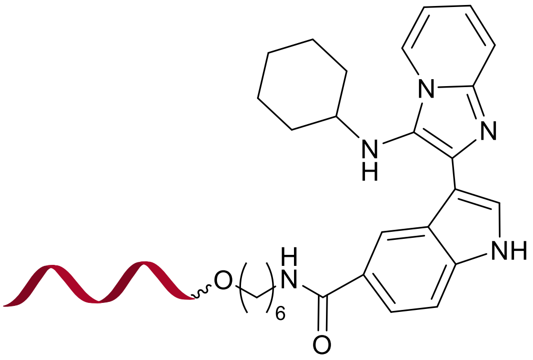

ATGC-10d

MS calc. 4765.0; found: 4767.5

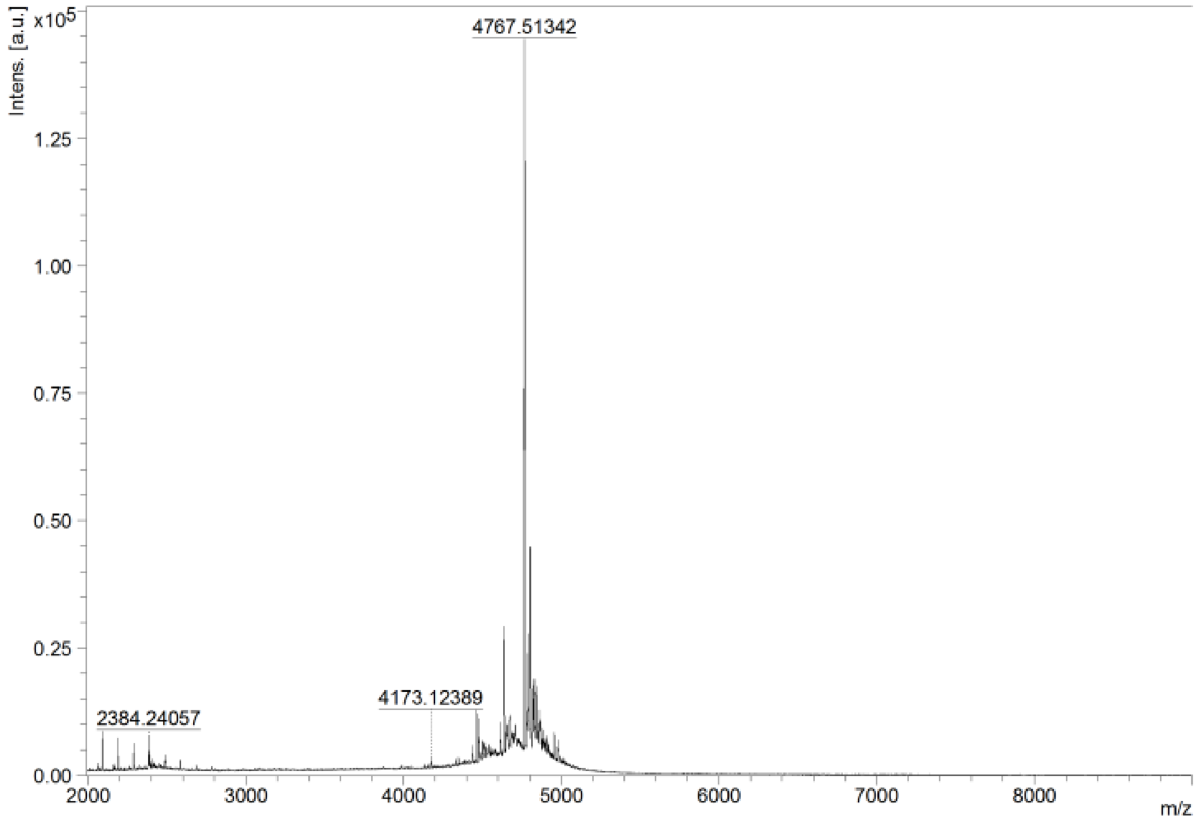

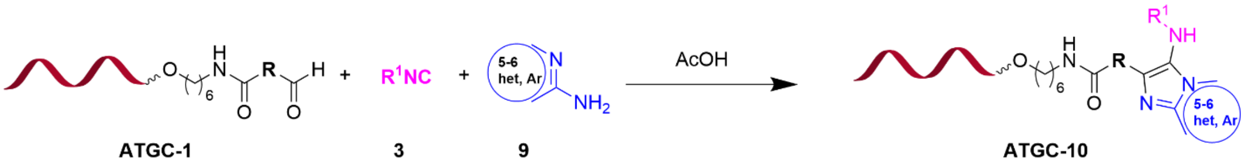

ATGC-1 3 9 ATGC-10

Table S23. Product conversions of products of the Groebke-Blackburn-Bienaymé three-component reaction with different aldehydes coupled to 14mer ATGC.

No.	**R**	**R[1]**	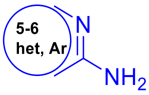	**Product conversion**
ATGC-10a	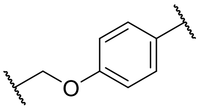	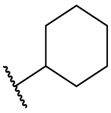	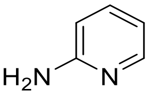	>95%
ATGC-10b	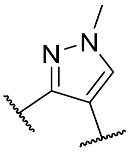	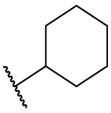	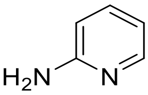	87%
ATGC-10c	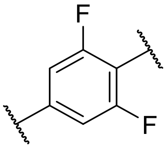	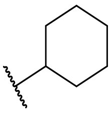	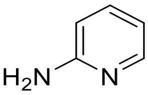	>95%
ATGC-10d	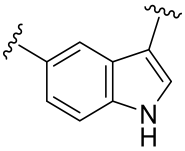	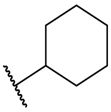	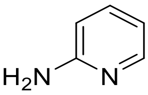	90%

Groebke-Blackburn-Bienaymé three-component reaction with a mixture of 14mer 7dATC-coupled aldehydes

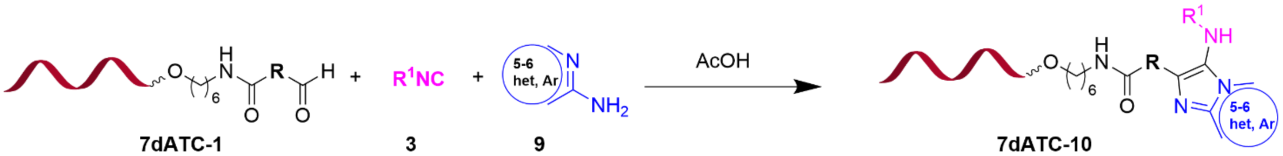

7dATC-1 **3** **9** **7dATC-10**

Table S24. MALDI-MS data of products of the Groebke-Blackburn-Bienaymé three-component reaction with different aldehydes coupled to 14mer 7dATC. Mixture experiment.

**MALDI-MS m/z MIXTURE**					
No.	R	R^1	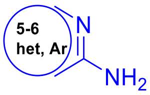	calculated	found
MixGBB1	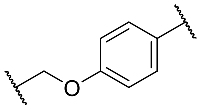	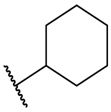	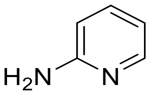	4774.0	4775.3
MixGBB2	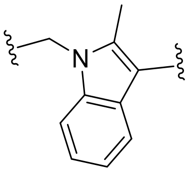	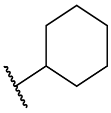	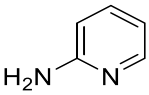	4811.0	4812.3
MixGBB3	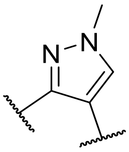	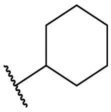	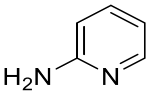	4748.0	4749.3
MixGBB4	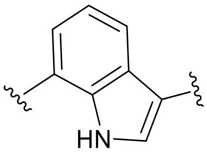	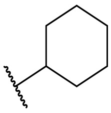	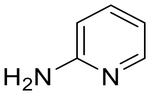	4783.0	4785.3

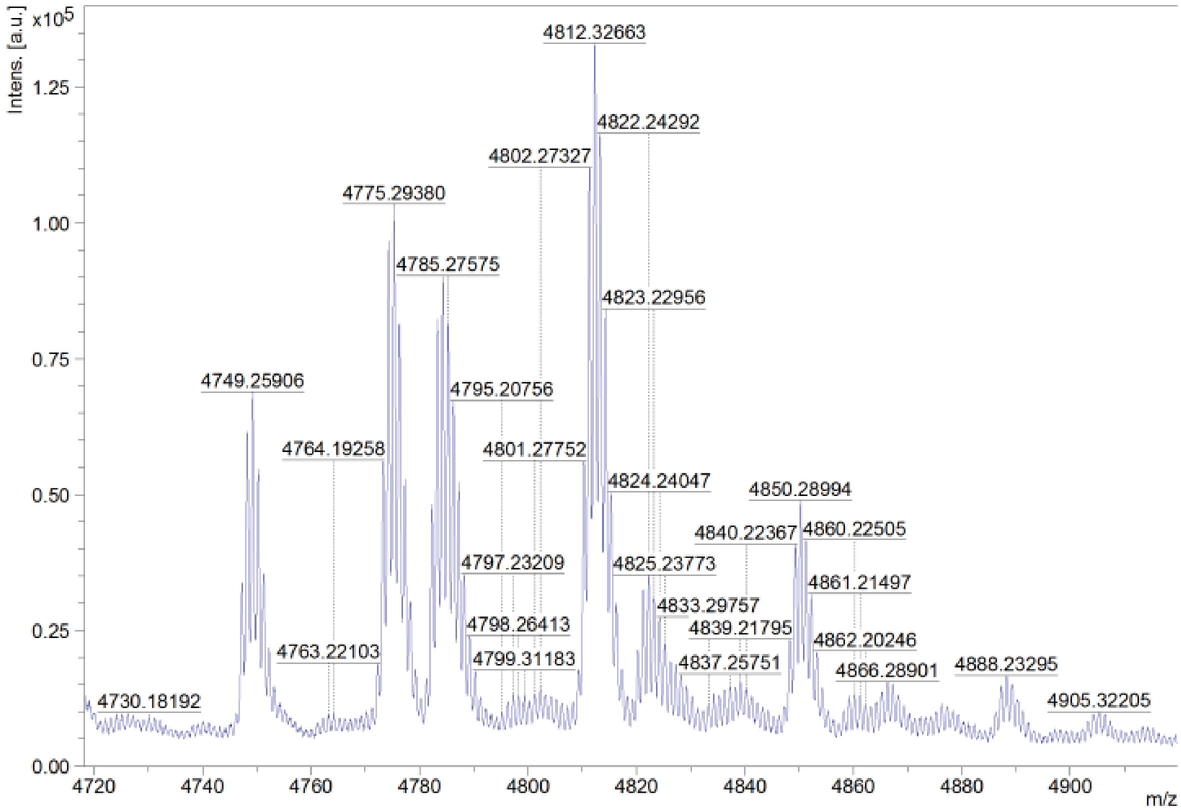

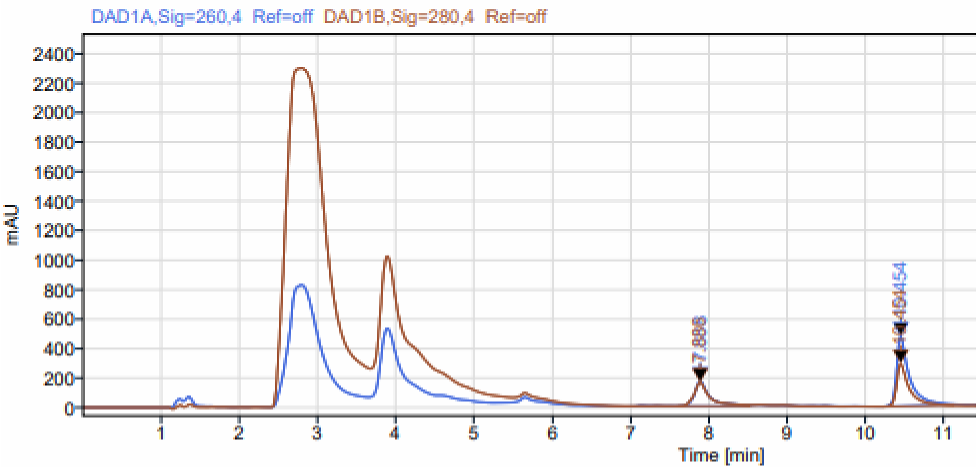

Reaction conditions: 7dATC-substrates (3 nmol), copolymer (90 nmol) and heteroaromatic amine **9** (6 µmol) in MeOH/$CHCl_3$ (80 µL; 7:1, v/v) for 6h at rt. Addition of isocyanide **3** (6 µmol) and acetic acid (0.8 µL; c= 1 vol%). The reaction was run for 16h at 25°C.

Quality assessment of DNA barcodes after multicomponent reactions by qPCR, PCR and Sanger sequencing

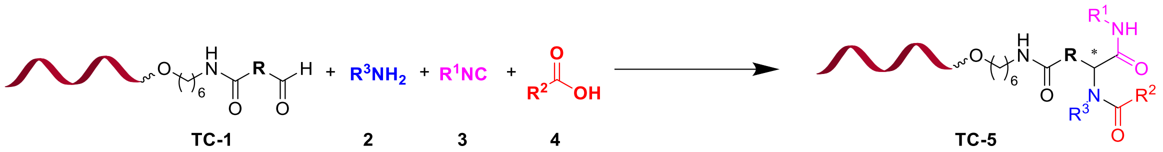

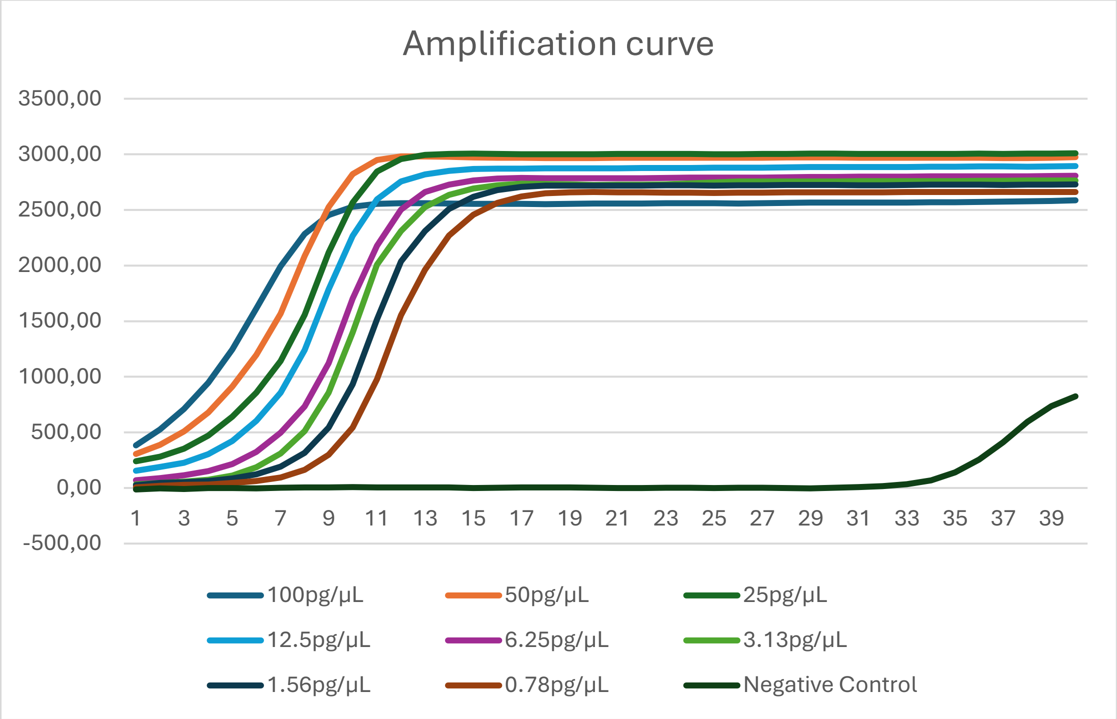

Figure S67. qPCR amplification curves for a dilution series of the ligated U-4CR barcode.

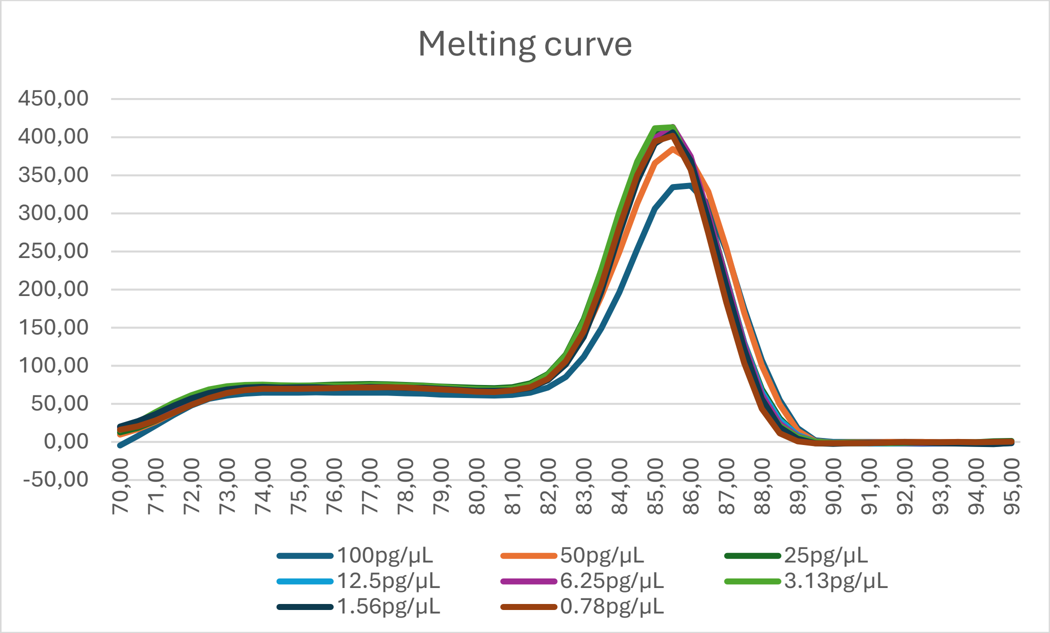

Figure S68. Melting curve analysis of U-4CR barcode amplicons from the qPCR shown **in Fig. S67**.

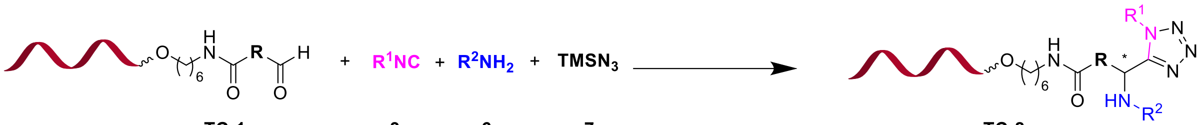

TC-1 3 6 7 TC-8

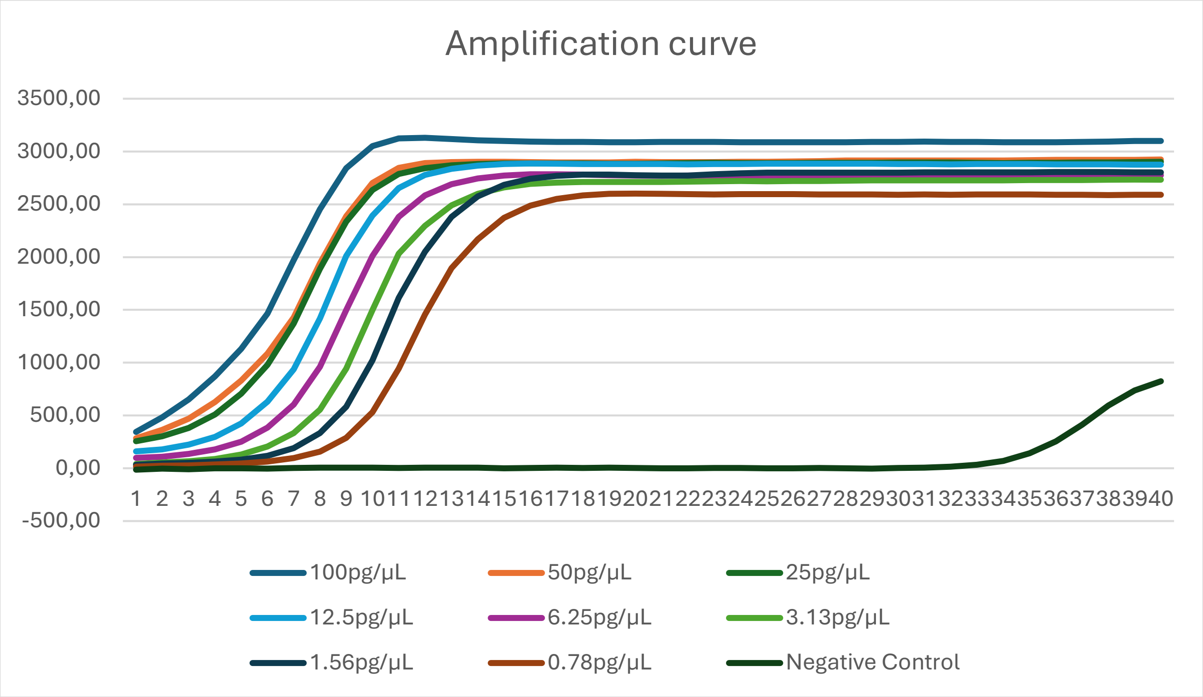

Figure S69. qPCR amplification curves for a dilution series of the ligated UA-4CR barcode.

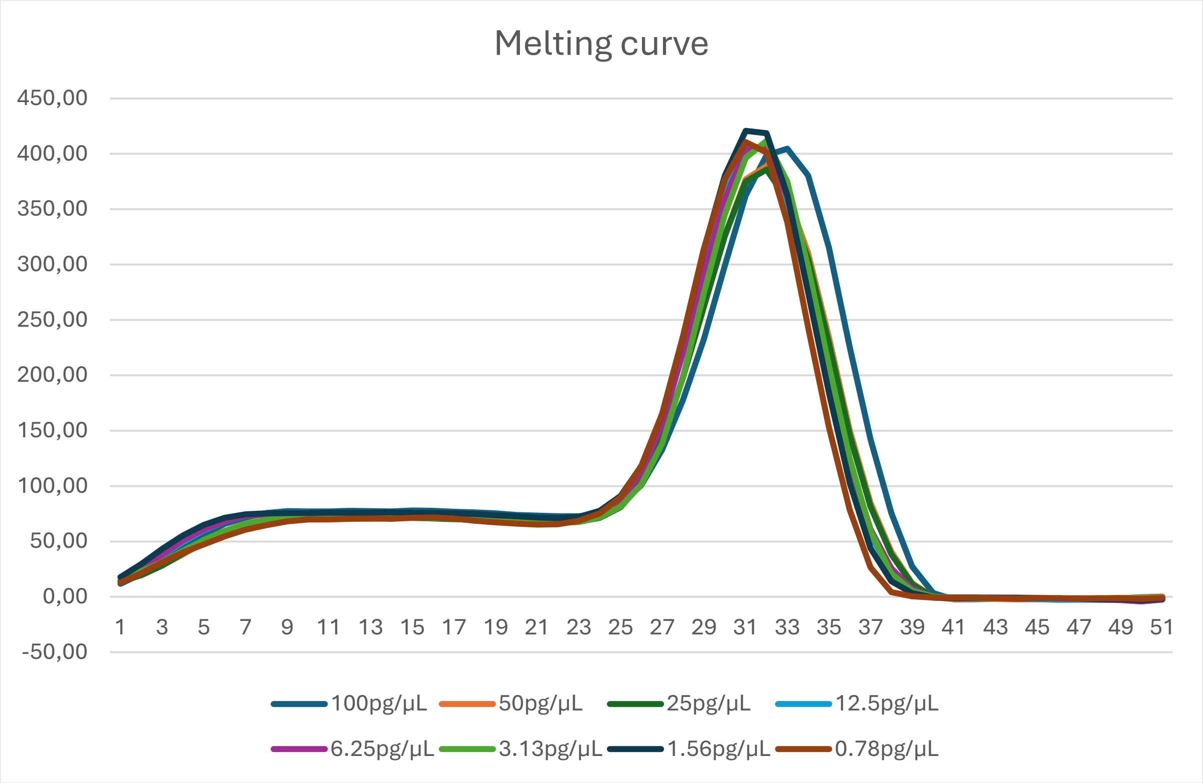

Figure S70. Melting curve analysis of UA-4CR barcode amplicons from the qPCR shown **in Fig. S69**.

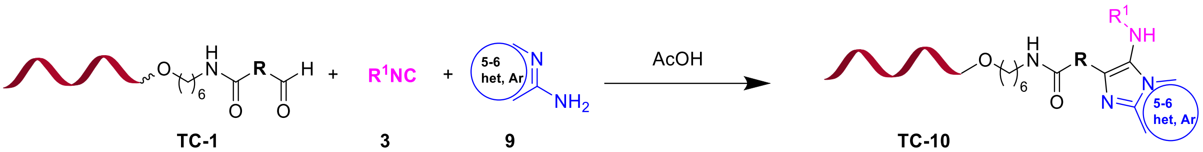

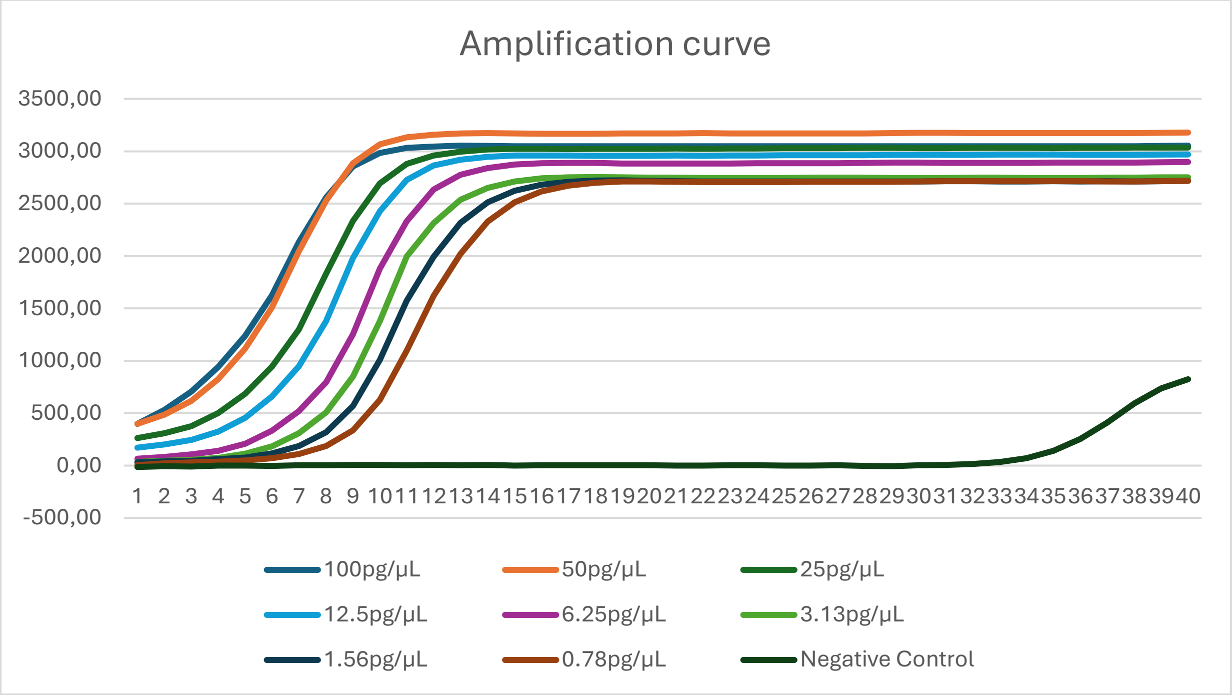

Figure S71. qPCR amplification curves for a dilution series of the ligated GBB-3CR barcode.

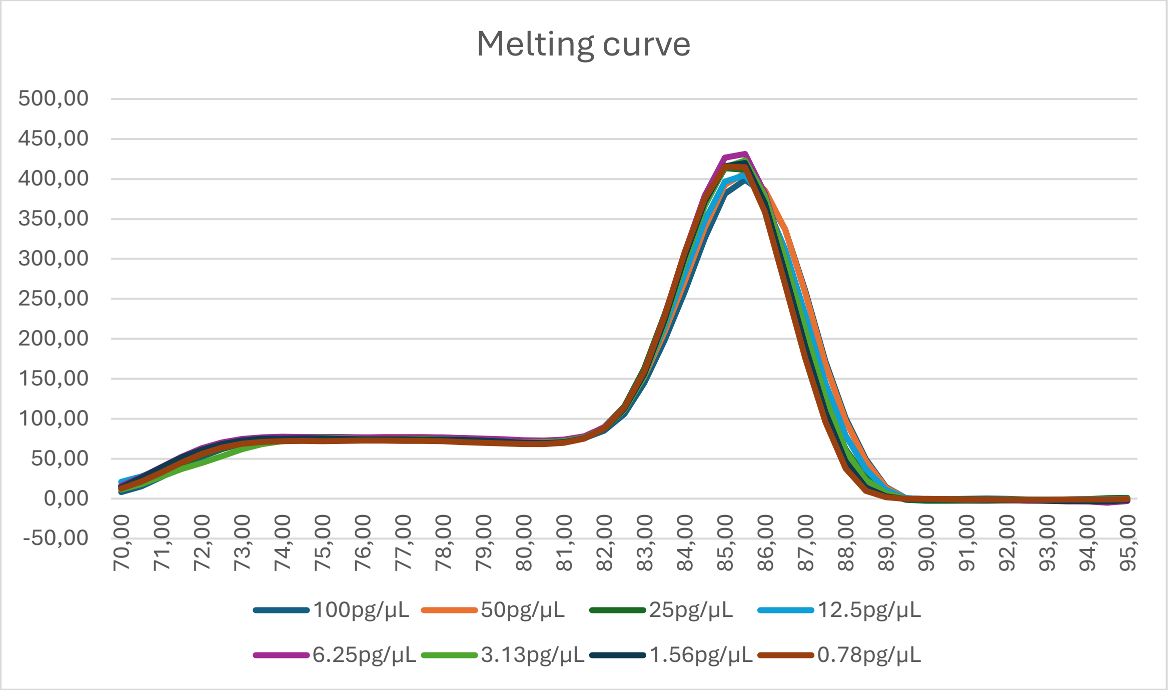

Figure S72. Melting curve analysis of GBB-3CR barcode amplicons from the qPCR shown in **Fig. S71**.

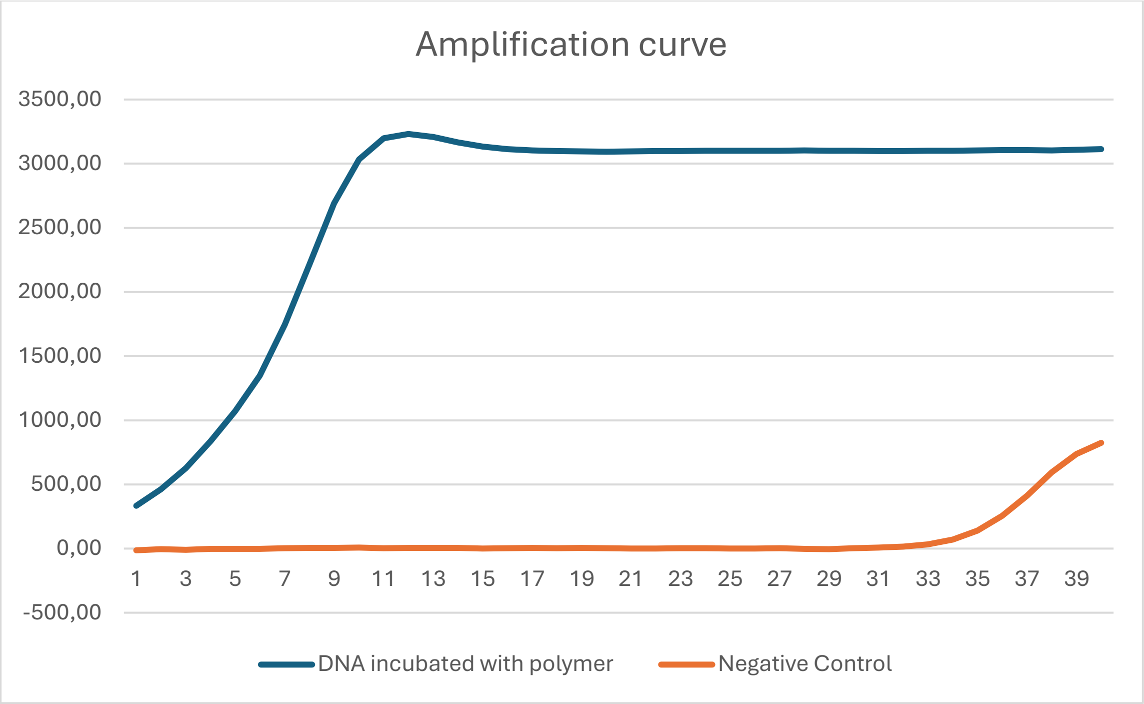

Figure S73. qPCR amplification curve for the ligated GBB-3CR barcode. In this experiment we added 30 eq of the polymer on purpose to the polymerase chain reaction to assess possible interference with DNA copying.

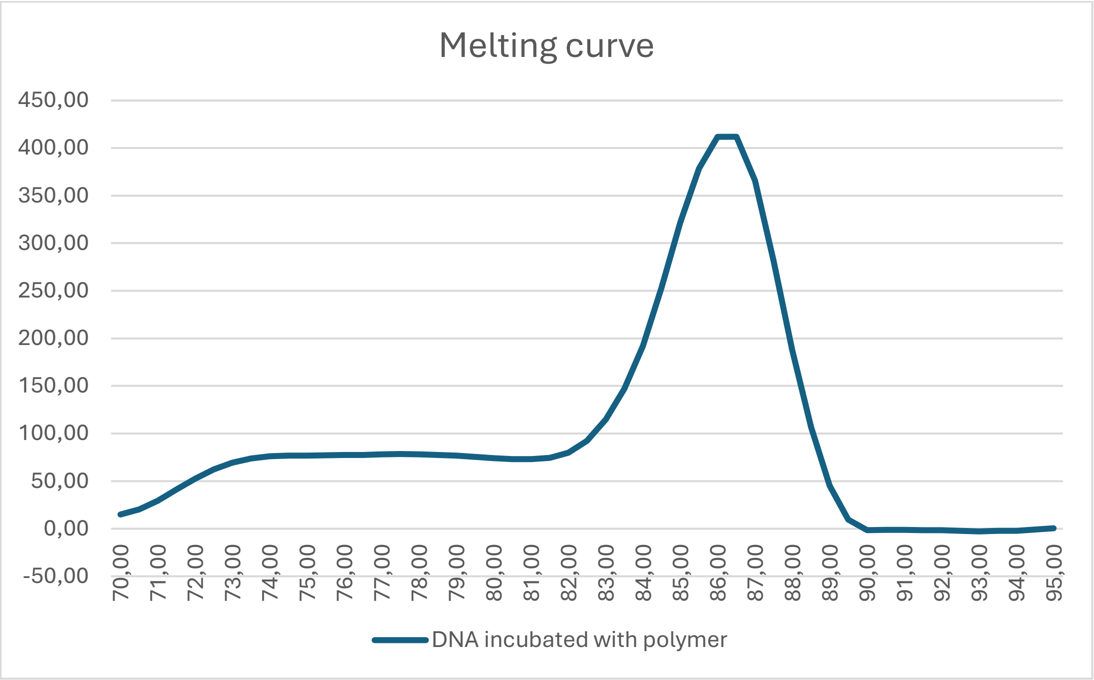

Figure S74. Melting curve analysis of the barcode amplicon from the qPCR shown in **Fig. S73**.

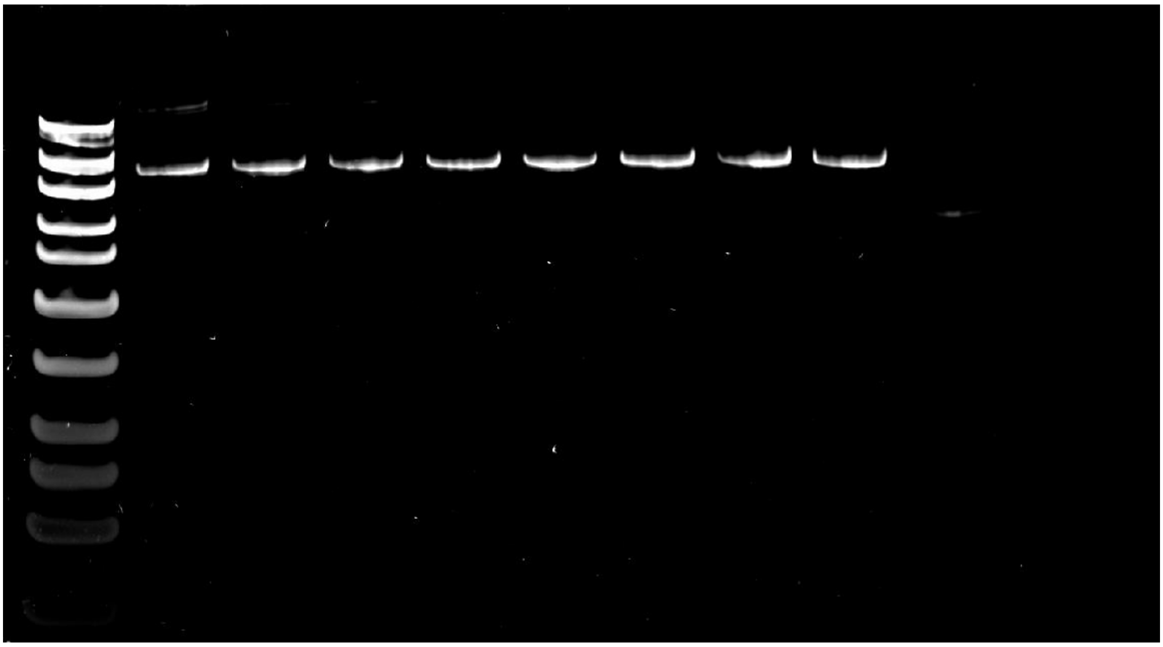

From left to right:

1) Ladder
2) 100 pg/µL DNA after qPCR
3) 50 pg/µL DNA after qPCR
4) 25 pg/µL DNA after qPCR
5) 12.5 pg/µL DNA after qPCR
6) 6.25 pg/µL DNA after qPCR
7) 3.13 pg/µL DNA after qPCR
8) 1.56 pg/µL DNA after qPCR
9) 0.78 pg/µL DNA after qPCR
10) Negative control

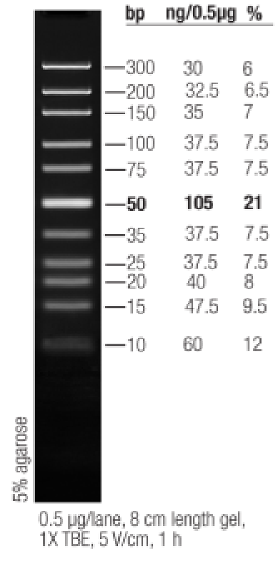

GeneRuler DNA Ladder, Ultra Low Range, Thermo Scientific

Figure S75. Agarose gel analysis of amplicons of the U-4CR barcode (qPCR, see **Fig. S67**).

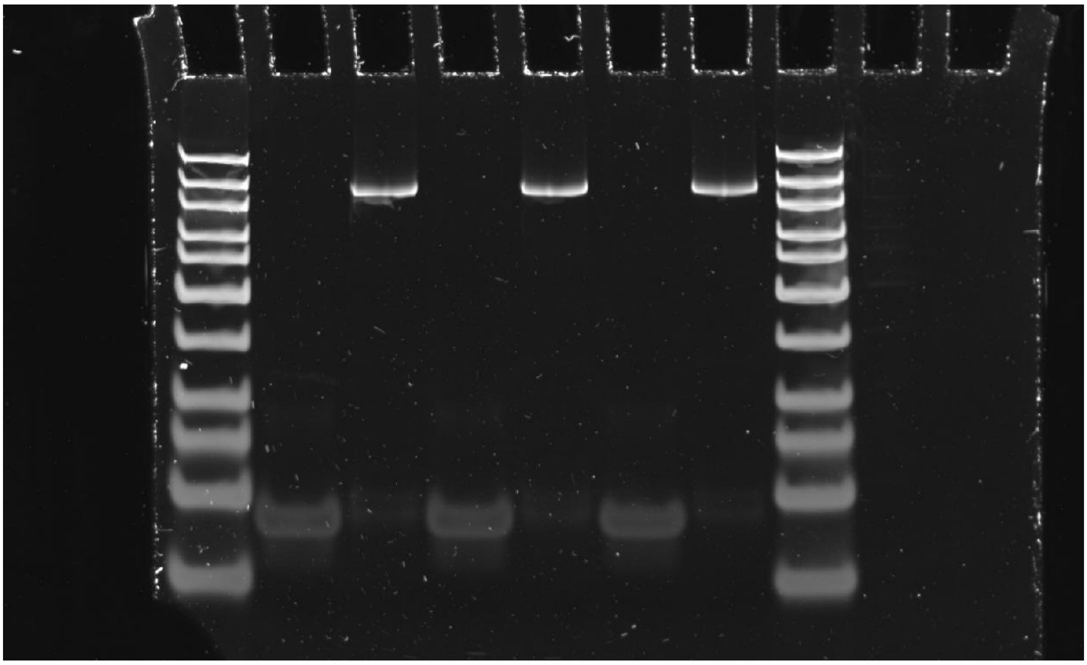

From left to right:

1) Ladder
2) Reference (14mer 7dATC product **7dATC-5a**)
3) Sample 1 (ligated 14mer 7dATC product **7dATC-5a** Ugi-4CR [178 bp])
4) Reference (14mer 7dATC product **7dATC-8c**)
5) Sample 2 (ligated 14mer 7dATC product **7d ATC-8c** Ugi-Azide-4CR [178 bp])
6) Reference (14mer 7dATC product **7dATC-10c**)
7) Sample 3 (ligated 14mer 7dATC product **7dATC-10c** GBB-3CR [178 bp])
8) Ladder

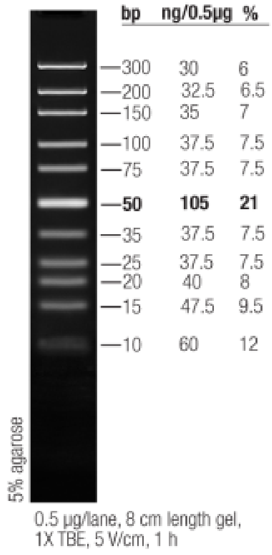

GeneRuler DNA Ladder, Ultra Low Range, Thermo Scientific

Figure S76. Agarose gel results for specified products of U-4CR, UA-4CR and GBB-3CR.

Sequencing results for product 7dATC-5a:

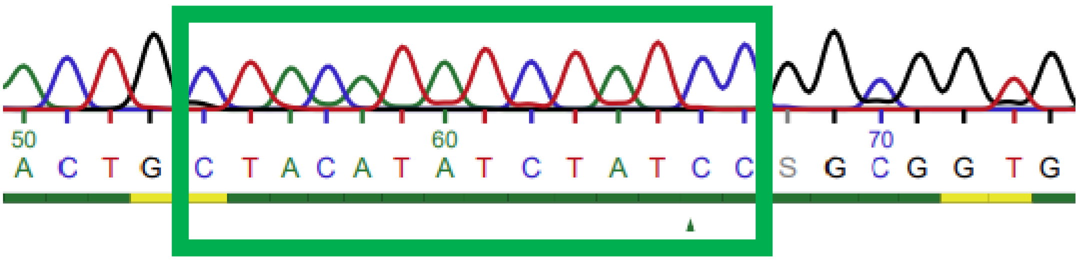

Sequencing results for product 7dATC-8c:

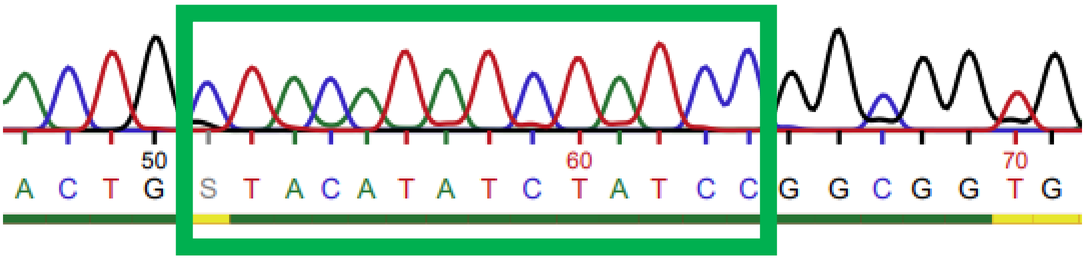

Sequencing results for product 7dATC-10c:

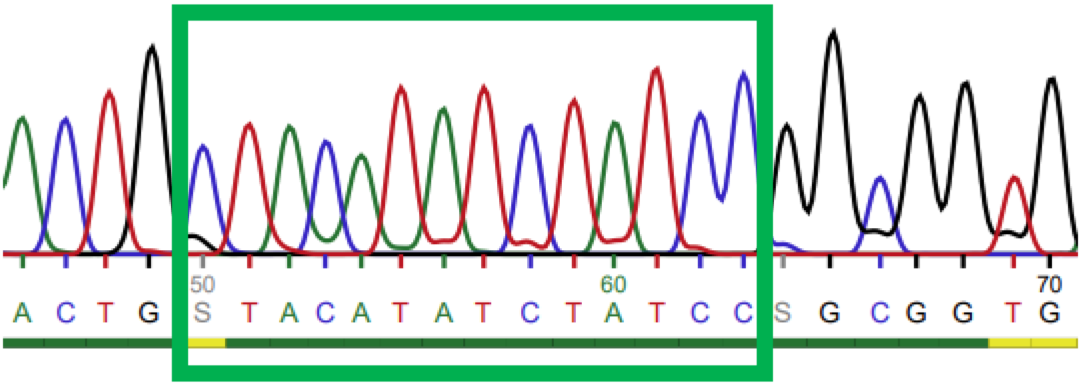

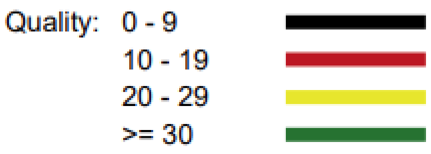

Figure S77. Sequencing results for mixtures of U-4CR (upper panel), UA-4CR (middle panel) and GBB-3CR (lower panel).
